# SPI2 T3SS effectors facilitate enterocyte apical to basolateral transmigration of *Salmonella*-containing vacuoles *in vivo*

**DOI:** 10.1080/19490976.2021.1973836

**Published:** 2021-09-20

**Authors:** Marcus Fulde, Kira van Vorst, Kaiyi Zhang, Alexander J. Westermann, Tobias Busche, Yong Chiun Huei, Katharina Welitschanski, Isabell Froh, Dennis Pägelow, Johanna Plendl, Christiane Pfarrer, Jörn Kalinowski, Jörg Vogel, Peter Valentin-Weigand, Michael Hensel, Karsten Tedin, Urska Repnik, Mathias W. Hornef

**Affiliations:** a Department of Veterinary Medicine, Freie Universität Berlin, Institute of Microbiology and Epizootics, Berlin, Germany; b Institute of Medical Microbiology, Rwth University Hospital Aachen, Aachen, Germany; c Institute of Molecular Infection Biology (IMIB), University of Würzburg, Würzburg, Germany; d Helmholtz Institute for RNA-based Infection Research (HIRI), Helmholtz Centre for Infection Research (HZI), Würzburg, Germany; e Technology Platform Genomics, Center for Biotechnology (Cebitec), Bielefeld University, Bielefeld, Germany; f Hannover Medical School, Institute for Medical Microbiology and Hospital Epidemiology, Hannover, Germany; g Department of Veterinary Medicine, Freie Universität Berlin, Institute of Veterinary Anatomy, Berlin, Germany; h Institute for Anatomy, University of Veterinary Medicine Hannover, Foundation, Hannover, Germany; i Institute of Microbiology, University of Veterinary Medicine Hannover, Foundation, Hannover, Germany; j Division of Microbiology, University of Osnabrück, Osnabrück, Germany; k Department of Biosciences, University of Oslo, Oslo, Norway

**Keywords:** *Salmonella*, *Salmonella* pathogenicity island 2 (Spi-2), enterocyte, apical to basolateral transmigration, mucosal translocation

## Abstract

*Salmonella* pathogenicity island (SPI) 2 type three secretion system (T3SS)-mediated effector molecules facilitate bacterial survival in phagocytes but their role in the intestinal epithelium *in vivo* remains ill-defined. Using our neonatal murine infection model in combination with SPI2 reporter technology and RNA-Seq of sorted primary enterocytes, we demonstrate expression of SPI2 effector molecules by intraepithelial *Salmonella* Typhimurium *(S*. Typhimurium). Contrary to expectation, immunostaining revealed that infection with SPI2 T3SS-mutants resulted in significantly enlarged intraepithelial *Salmonella*-containing vacuoles (SCV) with altered cellular positioning, suggesting impaired apical to basolateral transmigration. Also, infection with isogenic tagged *S*. Typhimurium strains revealed a reduced spread of intraepithelial SPI2 T3SS mutant *S*. Typhimurium to systemic body sites. These results suggest that SPI2 T3SS effector molecules contribute to enterocyte apical to basolateral transmigration of the SCV during the early stage of the infection.

## Introduction

Enteric and typhoid *Salmonella* colonize the gut lumen and subsequently penetrate the intestinal epithelial barrier to gain entrance to the sterile subepithelial and lymphoid tissue. The critical and non-redundant role of this early step in the course of the infection *in vivo* is illustrated by the ability of all *Salmonella* subspecies including human clinical isolates to invade non-phagocytic intestinal epithelial cells^1^. Internalization by enterocytes is facilitated by *Salmonella* pathogenicity island (SPI)1 type three secretion system (T3SS)-translocated effector molecules. Once internalized, *Salmonella* resides in a membrane-enclosed cell compartment, the *Salmonella-*containing vacuole (SCV). Cell-derived signals induce expression of a second, SPI2-encoded T3SS and set of effector molecules.^2,3^ In phagocytes, SPI2 T3SS effectors critically contribute to intracellular survival, facilitating spread to systemic organs and persistent infection.^4,5^ In non-polarized epithelial cells, SPI2 T3SS effector molecules localize the SCV to the peri-Golgi region, manipulate the endosomal compartment and ensure nutrient supply and intracellular proliferation.^6–9^ Several reports have linked an intact SPI2 T3SS with invasiveness and penetration of the mucosal tissue.^10–13^ However, few studies have analyzed the molecular mechanisms that facilitate penetration of the intestinal epithelial barrier, i.e. apical to basolateral transmigration and enterocyte egress.

We have recently shown that infection of neonate mice allows the visualization and quantitative analysis of infected enterocytes.^14^ Neonate mice exhibit reduced exfoliation of infected enterocytes.^15,16^ Using this neonate infection model, we previously characterized the requirement of individual SPI1 T3SS effector molecules for enterocyte invasion and intracellular proliferation.^17^ Here, we extended this work and analyzed the role of the SPI2 T3SS during this early step in the course of the infection.

## Results

**Figure 1.**
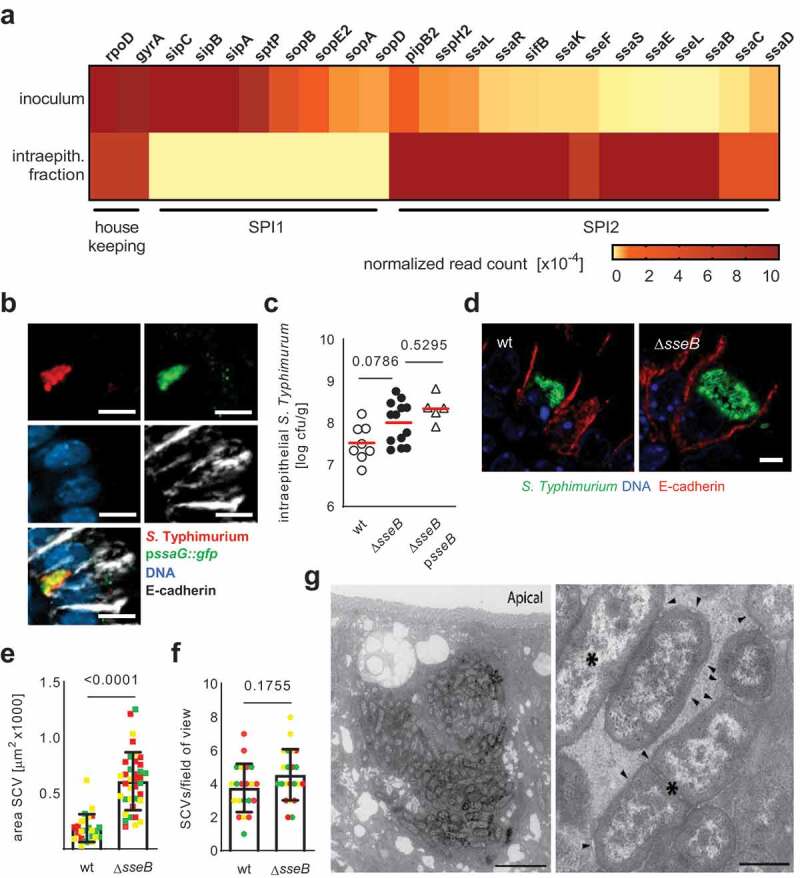
Formation of enlarged not smaller SCVs by SPI2 mutant *S*. Typhimurium. (a) normalized sequence read counts (number of reads mapped to a gene divided by the total number of bacterial reads in that library) for selected SPI1 and SPI2 associated genes obtained by RNA Seq analysis of the inoculum and RNA isolated from FACS-sorted *S*. Typhimurium-positive intestinal epithelial cells at 4 days p.i. with wt *S*. Typhimurium (for details see Experimental Procedures). (b) Immunofluorescence imaging of wt *S*. Typhimurium (red) carrying a SsaG-GFP reporter plasmid (green). counterstaining with DNA (blue) and E-cadherin (white). A representative image is shown. Bar, 5 µm. (c) The bacterial load in isolated small intestinal epithelial cells was analyzed by serial dilution and plating at day 4 p.i. with wt, Δ*sseB* SPI2 T3SS mutant or Δ*sseB* p*sseB* complemented S. Typhimurium. ANOVA with Bonferroni´s multiple post test; results from n = 6 (wt), n = 11 (Δ*sseB*) or n = 5 (Δ*sseB* p*sseB*) individual animals from one (wt and Δ*sseB* p*sseB*) or two (Δ*sseB*) litters. Shown are individual data points plus mean. *P*-values are indicated. (d) Immunostaining for wt and Δ*sseB S*. Typhimurium in small intestinal tissue sections of mice 4 days p.i. A representative image is shown. Bar, 5 µm. counterstaining with DNA (blue) and E-cadherin (red). Quantification of the size (e) and number (f) of SCVs. One data point represents one SCV (E) or one field of view (F). At least 40 SCVs or 20 fields of view from 3 different animals indicated by individual colors were analyzed. Student’s t-test. Box plots show median, quartiles and extremes. *P*-values are indicated. (g) Transmission electron microscopy of infected small intestinal epithelial cells 4 days p.i. with Δ*sseB S*. Typhimurium. Bar, 5 µm (left panel) and 500 nm (right panel). Arrowheads indicate the limiting membrane of the SCV that tightly surrounds bacteria. Asterisks label proliferating bacteria as suggested by the presence of a septum

Using transcriptomic analysis of primary *S*. Typhimurium-infected intestinal epithelial cells isolated and sorted by flow cytometry on day 4 post infection (p.i.), we observed expression of mRNAs for SPI2 T3SS components and effector molecules by intraepithelial wildtype (wt) *S*. Typhimurium *in vivo* (Figure 1(a)). SPI2 induction in intraepithelial wt *S*. Typhimurium *in vivo* was confirmed using a p*ssaG::gfp* reporter construct (Figure 1(b)). Interestingly, SPI2 T3SS effector gene expression and SCV formation by wt *S*. Typhimurium in intestinal epithelial cells did not require innate immune stimulation previously reported to mediate endosomal acidification and drive SPI2 T3SS effector gene expression in myeloid cells (Fig. S3A-D).^2,3^ SPI2 T3SS effector expression in primary enterocytes in our neonatal infection model prompted us to analyze the functional role of the SPI2 T3SS during enterocyte infection. Initially, we compared the clinical course and organ load between neonate mice infected with wt *S*. Typhimurium or isogenic mutants lacking the SPI2 T3SS translocon component SseB and unable to translocate effector molecules *via* the SPI2 T3SS (Δ*sseB*).^18^ Consistent with previous reports on the critical role of SPI2 T3SS effector molecules for survival in phagocytes and the intracellular localization of a major fraction of *S*. Typhimurium in systemic organs, Δ*sseB S*. Typhimurium exhibited a strongly attenuated phenotype compared to wt bacteria illustrated by decreased bacterial numbers in spleen and liver tissue and loss of virulence (Fig. S1A-C).

Surprisingly, the number of *S*. Typhimurium in isolated primary small intestinal epithelial cells was not reduced in the absence of a functional SPI2 T3SS (Figure 1(c)). In fact, the size of the intraepithelial SCV after Δ*sseB* SPI2 T3SS mutant *S*. Typhimurium infection *in vivo* was not reduced but rather enlarged (Figure 1(d-e)) with an unchanged number of infected cells per field of view (Figure 1(f)). Ultrastructural analysis of SPI2 T3SS mutant *S*. Typhimurium infected enterocytes revealed large SCVs covering a major part of the epithelial cell cytosol, enclosed by an intact membrane and filled with actively dividing bacteria (Figure 1(g)). Despite extensive electron microscopy analysis, no endosomal rupture or cytosolic bacteria and maximal one SCV per cell were observed for Δ*sseB S*. Typhimurium similar to what we have previously reported for wt *S*. Typhimurium.^14^ Also, the SCV membrane of Δ*sseB* S. Typhimurium was positive for the established SCV marker proteins LAMP-1 and LAMP-2, as shown by immunostaining and immunoelectron microscopy (Fig. S1D-F). Infection with a 1:1 ratio of GFP- and DsRed-labeled SPI2 T3SS mutant bacteria resulted in only single colored SCVs, demonstrating that SCVs emerged by clonal expansion of intraepithelial bacteria similar to what had been observed for wt *S*. Typhimurium (Fig. S1G).^14^

**Figure 2.**
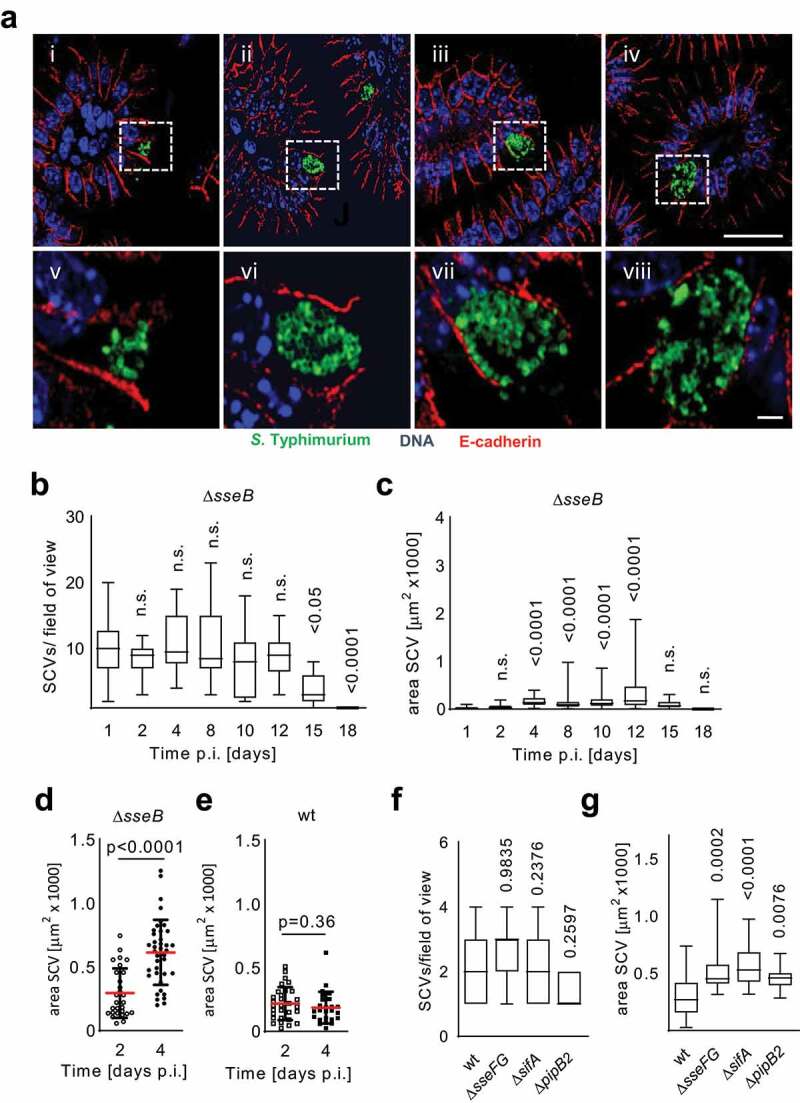
SPI2 mutant *S*. Typhimurium SCVs continuously increase in size during the course of infection. (a-c) continuous growth of SPI2 T3SS mutant SCVs. One-day old mice were orally infected with 10^2^ CFU ∆*sseB S*. Typhimurium. (a) small intestinal tissue sections were examined by immunofluorescence at 2 (i), 4 (ii), 8 (iii) and 12 (iv) days p.i. Representative images at 2, 4, 8 and 12 days p.i. (i–iv, bar, 25 µm), and enlarged views (v–viii, bar, 2.5 µm) are shown. (b and c) Small intestinal tissue sections evaluated at 1, 2, 4, 8, 10, 12, 15 and 18 days p.i. for the number (b) and size (c) of SCVs. One data point represents one field of view (B) or one SCV (C). At least 40 SCVs or 20 fields of view from 2 (day 1, 2, 8, 12, 15, 18) or 3 (day 4, 10) different from each one litter were analyzed. Significance was calculated with ordinary one-way ANOVA and Dunnett’s multiple comparison test. The indicated *p* values compare each time point to day 1 p.i. box and whiskers blots with median, lower and upper quartile and range are shown. *P*-values are indicated; n.s., not significant. (d and e) One-day-old mice were orally infected with ∆*sseB* (d) or wt (e) *S*. Typhimurium and small intestinal tissue sections were examined at day 2 and 4 p.i. by immunofluorescence for the size of SCVs. one data point represents one SCV. The SCV size was determined in at least 40 fields of view in tissue sections from each 3 individual animals from one litter per time point. significance was calculated with the Student’s t-test. Mean and SD are shown. *P*-values are indicated. (f and g) 1-day-old mice were orally infected with 10^4^ WT or isogenic Δ*sseFG*, Δ*pipB2* or Δ*sifA* mutants. SCV number (f) and SCV size (g) in intestinal epithelial cells was analyzed at 2 days p.i. At least 30 fields of view (F) or 30 SCVs (G) from 2 (wt, Δ*sseFG*, Δ*sifA*) or 3 (Δ*pipB2*) different animals from each one litter were analyzed. box and whiskers blots with median, lower and upper quartile and range are shown. Significance was tested using ANOVA and Bonferroni’s multiple posttest; the indicated *p*-values refer to each mutant in comparison to wt *S*. Typhimurium

Further in-depth analysis of the number and size of Δ*sseB* SPI2 T3SS mutant SCVs during the course of the infection revealed a stable number but steady increase in size during disease progression (Figure 2(a-c)). This increase was found after infection with Δ*sseB* SPI2 T3SS mutant but not wt *S*. Typhimurium (Figure 2(d-e)). Even SPI2 T3SS mutant *S*. Typhimurium additionally lacking uptake systems for important nutrients such as glucose, amino acids, fatty acids or peptides formed large SCVs (Fig. S3G-I). Nutrient availability of intraepithelial SPI2 T3SS mutant *S*. Typhimurium could be explained by access to luminal energy-rich material (Fig. S3E and F).

Several SPI2 effector molecules interact with the host cell cytoskeleton and motor proteins and could account for the observed phenotype.^19,20^ For example, SifA was reported to bind via the kinesin-interacting protein (SKIP) the microtubule-based motor kinesin-1 and PipB2 to promote kinesin-1 accumulation on the SCV membrane.^21,22^ Similarly, SseF and SseG was shown to contribute to the positioning of the SCV possibly also by regulating microtubule motors on the vacuole.^23^ We therefore compared the frequency and size of intraepithelial SCVs between wt, Δ*sifA*, Δ*pipB2* and Δ*sseFG* mutant *S*. Typhimurium. Indeed, Δ*sifA*, Δ*pipB2* and Δ*sseFG S*. Typhimurium mutants at 2 days p.i. exhibited an unaltered SCV frequency (Figure 2(f)) but significantly enhanced vacuolar size (Figure 2(g)).^24^ Thus, the SPI2 effector molecules SifA, PipB2, SseF and SseG may contribute to the described phenotype of SPI2 T3SS mutant *S*. Typhimurium.

**Figure 3.**
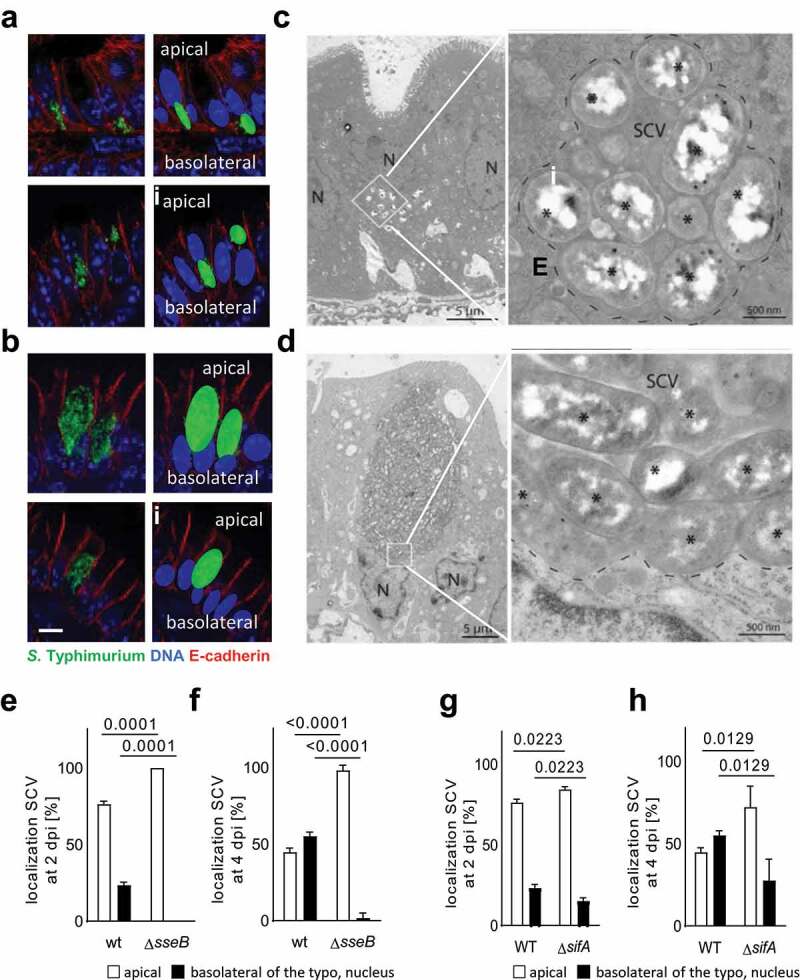
Altered positioning SPI2 mutant *S*. Typhimurium SCVs in enterocytes. (a-f) intraepithelial SCV positioning at 4 days p.i. with wt (a and c) or Δ*sseB S*. Typhimurium (green) (b and d) by immunofluorescence (a and b) or transmission electron microscopy (TEM) (c and d). (A and B) counterstaining of the immunostaining with DNA (blue) and E-cadherin (red). To better visualize the relative intracellular positioning of the SCVs in respect to the nucleus, nuclei (blue) and SCVs (green) are colored in the right panel of A and B. Representative images are shown. bar, 5 µm. (C and D) N, nucleus. White squares in the left panels indicate the area that is ten-fold enlarged in the right panels; broken lines in the right panels indicate completely membrane-enclosed SCVs; stars indicate single bacteria. Bar in the left panel of C and D, 5 µm; bar in the right panel of C and D, 500 nm. (e-h) The position of wt *versus* Δ*sseB* (e and f) and wt *versus* Δ*sifA* (g and h) SCVs apical (white) or basolateral (black) to the nucleus was categorized 2 (e and g) and 4 (f and h) days p.i. with 10^2^ CFU wt, Δ*sseB* or Δ*sifA S*. Typhimurium. At least 30 individual cells from 2 (E and F) or 3 (F and H) different animals from one litter were evaluated. Significance was tested using a 2-way ANOVA with Sidak’s multiple comparison test. Mean ±SD are shown. *P*-values are indicated

Importantly, in addition to the size, also the intraepithelial positioning of SCVs differed between wt and SPI2 T3SS mutant *S*. Typhimurium (Figure 3(a-f)). The number of wt SCVs positioned apical to the nucleus decreased during the course of the infection concomitant with an increase in the number of basolateral SCVs. In sharp contrast, the vast majority of SPI2 T3SS mutant *S*. Typhimurium SCVs remained within the apical part of the enterocyte (Figure 3(e-f)). A less pronounced but significant effect on the intraepithelial positioning was also observed for Δ*sifA* mutant *S*. Typhimurium (Figure 3(g-h)).

**Figure 4.**
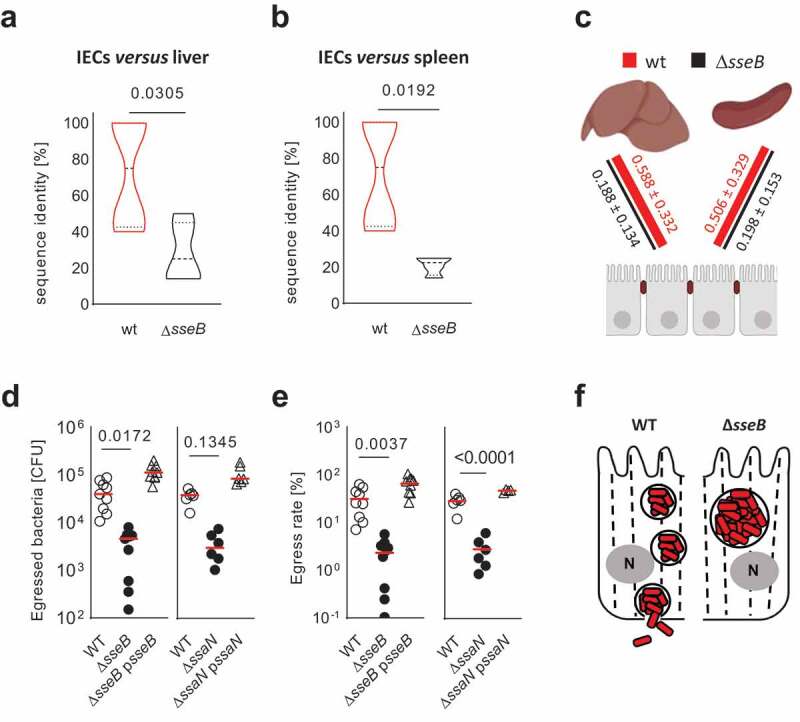
Altered population dynamic *in vivo* and enterocyte egress *in vitro* of SPI2 T3SS mutant *S*. Typhimurium. (a and b) violin plots to illustrate the tag sequence identity between intraepithelial wt and Δ*sseB S*. Typhimurium strains and total liver (a) or spleen (b) tissue from 4 (wt) or 6 (Δ*sseB*) animals from one litter. This method visualizes the presence/absence of sequence tags in each organ in respect to the total spectrum of sequence tags found in this animal at day 4 p.i. with an equal mixture of all 22 strains in a total of 10^3^ CFU bacteria. Significance was calculated with the Student’s t-test. Median (dashed line), quartile (dotted line) and *p*-values are shown. (c) Bray-Curtis Index was substracted from 1 for comparison of the similarity between the spectrum of intraepithelial *S*. Typhimurium strains and the spectrum of *S*. Typhimurium strains in liver and spleen tissue between wt and Δ*sseB S*. Typhimurium. (d) number of wt, Δ*sseB* and Δ*ssaN* as well as complemented Δ*sseB*(p*sseB*) and Δ*ssaN* (p*ssaN*) *S*. Typhimurium that egressed at the basolateral side from apically infected polarized intestinal epithelial m-IC_cl2_ cells within 30 min. Individual data, median, and *p*-values are indicated. (e) egress rate (%) calculated as total egressed bacteria/intraepithelial bacteria/x100 (see Fig. S1JK). Each data point represents one transwell insert of in total 6–9; data were generated in three independent experiments. One-way ANOVA with Dunnett’s multiple comparisons test. individual data, median, and *p*-values are indicated. (f) cartoon illustrating the observed block in the transepithelial transport of SCVs generated by SPI2 mutant *S*. Typhimurium *in vivo.*

To further substantiate our hypothesis of SPI2-dependent enterocyte apical to basolateral transmigration as part of the epithelial barrier penetration process, we generated libraries of 22 isogenic, genome-tagged strains of each our wt and our Δ*sseB S*. Typhimurium strain. This neutral barcoding method allows the quantitative evaluation of population dynamics and bottlenecks during spread between different anatomical sites.^25^ Since it does not rely on total bacterial counts, it allows the detection of impaired mucosal translocation and thus a reduced number of translocation events, independent of the reduced survival i.e. the absolute number of SPI2 T3SS mutant *versus* wt *S*. Typhimurium in tissue resident and systemic phagocytes.^4,5^ Following oral administration of the bacterial library, i.e. an equal mixture of all 22 isogenic strains of either wt or Δ*sseB S*. Typhimurium, the presence and relative abundance of individual tagged strains was analyzed in the infection inoculum as well as the total spleen tissue, the total liver tissue and the intestinal epithelial cells isolated at day 4 p.i. by plating and subsequent DNA sequencing. Population similarities between the intraepithelial bacterial population and the population in liver or spleen tissue indicated by the calculated Bray-Curtis Index were markedly reduced in the absence of a functional SPI2 T3SS compared to wt *S*. Typhimurium (Figure 4(a-c)). These findings are consistent with a role of SPI2 T3SS translocated effector molecules in enterocyte apical to basolateral transmigration with subsequent cell egress and systemic dissemination of *S*. Typhimurium in the neonate host.

In order to more specifically address the role of the SPI2 T3SS during enterocyte egress and exclude an influence of the surrounding tissue on the observed phenotype, we employed a reductionist approach using highly polarized murine small intestinal epithelial m-IC_cl2_ cells grown on transwell filter inserts. Enterocyte invasion by *S*. Typhimurium in this model is SPI1 T3SS-dependent as illustrated by significantly reduced numbers of intraepithelial SPI1 T3SS mutant Δ*invC S*. Typhimurium in the classical gentamicin kill assay (Fig. S1H and I). Consistent with the *in vivo* phenotype (Figure 1(c)), deletion of the SPI2 T3SS translocon component SseB or the ATPase SsaN required for SPI2 T3SS-mediated effector molecule translocation did not alter intraepithelial survival and growth (Fig. S1J and K). Notably, the absence of a functional SPI2 T3SS significantly reduced the bacterial egress at the basolateral plasma membrane (Figure 4(d-e)) and this difference was not explained by an altered cell viability (Fig. S1L).^18,26^ Thus, a functional SPI2 T3SS was dispensable for intraepithelial survival but required for epithelial m-IC_cl2_ cell egress. Consistently, wt *S. Typhimurium* were identified in the *lamina propria* close to the epithelial layer *in vivo* (Fig. S2).

## Discussion

The results on *S*. Typhimurium in polarized intestinal epithelial cells obtained using our neonatal murine infection model suggest significant and previously ill-defined differences to the reported situation in phagocytes with respect to intracellular survival, SCV formation, and cellular positioning. In phagocytes, SPI2 T3SS effector molecules are mainly induced upon innate immune stimulation-mediated SCV acidification and exert a critical and non-redundant role in vacuolar remodeling, intracellular bacterial survival^2,3,12^ and macrophage polarization.^27^ In contrast, intracellular bacterial survival in polarized neonatal intestinal epithelial cells *in vivo* does not require an intact SPI2 T3SS. Instead, SPI1 T3SS virulence factors, in particular SipA, SopE_2_, and SopB, contribute to SCV formation in polarized epithelial cells.^17^

Endosomal rupture, release of *S*. Typhimurium into the cytosol and rapid cytosolic proliferation associated with SPI1 effector gene expression has been described.^28,29^ In our model, intraepithelial *S*. Typhimurium exhibited low SPI1 effector gene expression and we have been unable to detect endosomal rupture or cytosolic bacteria by electron microscopy. Although we have no evidence for endosomal rupture of intraepithelial SCVs, however, we cannot exclude the occurrence in a minority of cells possibly associated with rapid exfoliation into the intestinal lumen.

In non-polarized cells, SPI2 T3SS effectors were shown to induce the formation and extension of *Salmonella*-induced filaments (Sifs), longitudinal membrane tubules that extend from the SCV and promote intravacuolar nutrition.^9,24,30^ In contrast, the intestinal epithelium represents an absorptive surface for dietary nutritional substances and intraepithelial *S*. Typhimurium *in vivo* have access to luminal energy-rich material even in the absence of a functional SPI2 T3SS, rendering their intraepithelial growth independent of important nutrient-uptake systems. The observed decrease in the number of *S*. Typhimurium-positive enterocytes at later time points is most likely due to the start of enterocyte proliferation, migration along the crypt-villus axis and exfoliation known to start at approximately two weeks after birth as part of the postnatal mucosal tissue development.^31^

Also, the role of the SPI2 T3SS for the positioning of intracellular *S*. Typhimurium seems to differ between non-polarized cells and polarized enterocytes. In non-polarized cells, SPI2 T3SS effectors were reported to mediate paranuclear localization.^6,24,30^ In contrast, our findings in highly polarized intestinal epithelial cells *in vivo* suggest that the interaction of SPI2 T3SS effector molecules with the microtubule network and trafficking machinery facilitates apical to basolateral transmigration. Importantly, this is fully consistent with the different organization of the microtubule network in polarized and non-polarized cells. In non-polarized cells, microtubules extend radially from the microtubule organizing center (MTOC) localized close to the paranuclear Golgi apparatus, whereas in polarized enterocytes they extend from the apical membrane to the basolateral side to facilitate vectoral vesicle transport.^32^ The continued presence of SPI2 T3SS mutant microcolonies mainly at the apical pole is also reminiscent of the appearance of subapical vesicles in genetic disorders of the intracellular trafficking machinery.^33^ Thus, the enhanced size and block in apical to basolateral transmigration of SPI2 T3SS mutant and somewhat less pronounced also for SifA mutant SCVs as well as the enhanced size of SseFG and PipB2 deficient *S*. Typhimurium SCVs suggest a critical role of the SPI2 T3SS and a contribution of the translocated effector molecules SifA, SseFG and PipB2 in the microtubule-mediated transport of the SCV through the epithelial cell *in vivo*.

Our results thus explain the recent finding that the SPI2 T3SS exerts a critical role during the initial course of an oral *S*. Typhimurium infection.^12^ They are also in accordance with the recent report on SPI2 T3SS-dependent transport of *Salmonella* flagellin across the epithelium,^34^ and live-imaging studies of the translocation of *S*. Typhimurium.^11^ However, in the latter study, bacterial transcytosis was observed in the cecal epithelium, occurred as SCV containing a single bacterium, and was completed within a few hours. Notably, additional alternative SPI2 T3SS-dependent and independent pathways that facilitate epithelial barrier penetration may exist, explaining the low but detectable spread of SPI2 T3SS mutant *S*. Typhimurium to systemic sites in our neonatal infection model.

Finally, the enlarged SCVs following infection with SPI2 T3SS mutant *S*. Typhimurium are reminiscent of the phenotype described for Δ*ssaV* and Δ*sseB Salmonella* in hepatic leukocytes.^35^ Although impaired progression from the apical to the basolateral side of polarized epithelial cells can explain the observed continuous intravacuolar growth, the detection of giant SPI2 T3SS mutant *Salmonella* SCVs in myeloid cells indicates an additional role of SPI2 in cell egress, both from immune cells and polarized enterocytes. Consistently, a reduced number of infectious hepatic foci was observed in the above-mentioned study after systemic SPI2 mutant *Salmonella* infection.^35^ Similarly, a recent publication reported that SPI2-deficient *Salmonella* tissue persisters were impaired in their ability to reach the gut lumen and mediate spread of a conjugative plasmid to luminal recipient bacteria.^13^ Thus, the reduced enterocyte apical to basolateral transmigration by SPI2 deficient *S*. Typhimurium could explain the critical contribution of SPI2 T3SS effector molecules to epithelial intestinal mucosal inflammation observed in murine models of *Salmonella* enterocolitis.^36,37^ Our results assign the SPI2 T3SS a new function *in vivo*. Future studies are warranted to reveal the mechanistic details of the described process and to further investigate *S*. Typhimurium enterocyte egress at the basolateral side to complete the process of epithelial transcytosis.

## Methods

### Bacterial strains and plasmids

*Salmonella enterica* subsp. *enterica* serovar Typhimurium (*S*. Typhimurium) ATCC14028 (NCTC12023) and isogenic SPI1 T3SS (Δ*invC*), SPI2 T3SS (Δ*sseB*, Δ*ssaN*) and SPI-2 T3SS effector (Δ*sifA*, Δ*sseFG*, Δ*pipB2*) mutants as well the respective complemented strains were used for *in vivo* and *in vitro* infection experiments. For visualization in tissue sections and flow cytometric sorting, bacterial strains harbored plasmids encoding constitutively expressed mCherry, DsRed or green fluorescent protein (GFP) (pGFP, AmpR, kindly provided by Brendan Cormack, Stanford, USA). The generation of directed deletion mutants of SPI2 T3SS effector genes and other mutations of *S*. Typhimurium was performed as described previously.^38^ The strains and plasmids used in this study are listed in the supplemental table 1.

## In vitro *transcytosis assay in polarized epithelial cells*

Small intestinal epithelial m-IC_cl2_ cells were cultured in a defined medium and passaged every 2–3 days as previously described.^39^ Cells were seeded on 3 µm pore size PET ThinCert transwell inserts (Greiner Bio-one, Kremsmünster, Austria) and cultured for 10–12 days with medium change every other day to obtain a covalent layer of polarized epithelial cells. Integrity of the cellular monolayer was verified by monitoring of the transepithelial electrical resistance (TEER). wt or SPI2 T3SS Δ*sseB* and Δ*ssaN* mutant *S*. Typhimurium as well as their complemented strains Δ*sseB* (p*sseB*) and Δ*ssaN* (p*ssaN*) were grown overnight at 37°C, diluted 1:10 and sub-cultivated with mild agitation at 37°C, until mid-logarithmic growth was reached (OD_600_: 0.5). Bacteria were adjusted by dilution, added at a multiplicity of infection (MOI) of 10:1 to the apical medium compartment and centrifuged at 1,500 rpm for 5 min. Following incubation for one hour, the medium in the apical and basolateral compartment was replaced with fresh medium supplemented with 100 µg/mL gentamicin and incubated for another hour. The number of intracellular bacteria at 2 h was determined by washing three times in warm PBS followed cell lysis in 0.1% Triton H_2_O and serial dilution and plating. The invasion rate was calculated as $\frac{number\;of\;intracellular\;bacteria}{number\;of\;administered\;bacteria}$ $\times 100\left[\%\right]$. To determine the number of intracellular bacteria at 22 h, infected cells were incubated in medium supplemented with 20 µg/mL gentamicin in the apical and basolateral compartment for another 20 h, washed three times in warm PBS followed cell lysis in 0.1% Triton H_2_O and serial dilution and plating. To determine the number of bacteria that egressed into the basolateral medium compartment, the infected cells were incubated in fresh medium supplemented with 20 µg/mL gentamicin for 20 h as described above, washed three times in warm medium and incubated in gentamicin-free medium. The number of bacteria in the basolateral compartment was determined after 30 min by serial dilution and plating. The egress rate was calculated as $\frac{number\;of\;evaded\;bacteria}{number\;of\;intracellular\;bacteria\;at\;22\;h}$ $\times 100\left[\%\right]$. No difference in enterocyte viability was noted between wt infected and SPI2 T3SS mutant-infected cell monolayers by visual inspection and comparative analysis of the release of the cytoplasmic enzyme lactate dehydrogenase (LDH) into the cell culture medium (Cayman).

### Ethics

All animals were handled in accordance with regulations defined by FELASA and the national animal welfare body GV-SOLAS (http://gv-solas.de). *In vivo* experiments were performed in compliance with the German animal protection law (TierSchG) and approved by the local animal welfare committee (Niedersächsisches Landesamt für Verbraucherschutz und Lebensmittelsicherheit Oldenburg, Germany; Landesamt für Natur, Umwelt und Verbraucherschutz, North Rhine Westfalia, approval 33.14.42502–04-12/0693, 81–02.04.2017.A397 and Landesamt für Gesundheit und Soziales Berlin (LaGeSo), approval 15/0293 including all approved changes. The number of animals per group was defined prior to the start of the experiments based on previous results and the expected variation of the determined read out in the application for ethical approval (see above).

### Infection experiments

Adult C57BL/6 N wild type were obtained from Charles River Laboratory (Sulzfeld, Germany) and B6.129P2(SJL)-*MyD88tm1.1Defr*/J (MyD88-/-, stock no. 009088) mice from the Jackson Laboratory (Bar Harbor, USA), respectively. Animals were bred locally under SPF conditions. Bacteria were cultured in Luria Bertani (LB) broth containing 100 µg/mL ampicillin overnight at 37°C and 200 rpm. The next day, cultures were diluted 1:10 in fresh LB medium at identical incubation conditions until an OD_600nm_ of approximately 0.5 was reached. The bacterial pellet was washed in PBS twice and cultures were adjusted to the indicated infection dose and orally applied to 1-day-old mice in a volume of 1 μL PBS containing 10^2^ CFU if not stated otherwise. Infection with Δ*sifA*, Δ*sseFG* and Δ*pipB2* mutant strains (plus the comparative wt *S*. Typhimurium strain) in Figure 2(f-g) were administered at 10^4^ CFU to allow an earlier analysis of the SCV size already at day 2 p.i. At later time points, SCVs generated by Δ*sifA*, Δ*sseFG* and Δ*pipB2* mutant *S*. Typhimurium become unstable as previously reported.^40,41^ Infections with the 22 WITS strains were performed with an inoculum of 10^3^ CFU to allow the generation of an equal mixture of all WITS strains (see below). Litters were randomly assigned to infection with wildtype or SPI2 deficient *S*. Typhimurium. At the indicated time points p.i., animals were sacrificed and liver and spleen were harvested and homogenized in sterile PBS. Viable counts were obtained by serial dilution and plating on LB agar plates supplemented with the appropriate antibiotic. For the 1:1 co-infection with *S*. Typhimurium carrying the GFP or DsRed reporter plasmid, cultures were grown separately, adjusted and mixed 1:1 prior to oral challenge.

### Isolation of primary intestinal enterocytes

Primary intestinal epithelial cells were isolated from small intestinal tissues of infected neonates as described previously.^14^ Briefly, intestinal tissues of neonate mice were cut in small pieces and incubated in 30 mM EDTA PBS at 37°C for 10 min. Epithelial cells were detached from the underlying tissue by vigorous shaking and the cell suspensions were passed through a 100 µm nylon cell strainer (VWR). Cells were washed in 10% FCS/PBS, collected by centrifugation and the purity was verified by flow cytometry (E-cadherin^+^ CD45^−^). For the cultural quantification of intraepithelial *S*. Typhimurium *in vivo*, isolated epithelial cells were incubated in gentamycin (100 µg/mL) to remove extracellular bacteria, repeatedly washed and lysed followed by serial dilution and plating as recently described.^14^ To determine the bacterial organ count, organ homogenate or cell lysate was plated on LB agar supplemented with 100 µg/mL ampicillin by replica-plating.

### Immunofluorescence staining

Tissues were fixed in paraformaldehyde, embedded in paraffin and cut in 3 µm slices. Sections were deparaffinized in xylene and rehydrated in ethanol. Antigen retrieval was conducted in 10 mM sodium citrate for 10 min and blocking with 10% serum in PBS/BSA solution prior to immunofluorescence staining. For immunostaining, a chicken anti-GFP (dilution 1:500, Abcam), a rabbit anti- *S*. Typhimurium (dilution 1:1,000, Abcam), a mouse anti-E-cadherin (dilution 1:200, BD Transduction Laboratories), a rat anti-LAMP-1 (dilution 1:500, Developmental Studies Hybridoma Bank), and a rabbit anti-mCherry (dilution 1:500, Abcam) were used in the appropriate combination with a fluorophore conjugated secondary antibody (Jackson ImmunoResearch) as indicated. Slides were mounted in Vectashield (Vector) containing DAPI and pictures were taken with an ApoTome equipped Observer Z.1 fluorescence microscope equipped with axiovision software from Zeiss (Jena, Germany) or a DMI6000B fluorescence microscope equipped with LAS X software from Leica (Wetzlar, Germany). No blinding of tissue sections prior to the visual analysis was performed. Since the visualization of the SCV membrane markers LAMP-1 and LAMP-2 by immunostaining and electron microscopy as well as the ultrastructural studies and previous work (Zhang et al., 2014 and 2018) unequivocally demonstrated an intact SCV membrane around the intraepithelial bacteria in neonatal enterocytes, we measured the size of the bacterial microcolonies as a surrogate marker for the size of the SCVs. Images for the visual quantitative analysis of the SCV number/field of view were carefully selected to ensure a similar number of epithelial cells on each field of view prior to counting. In most figure panels, data points from individual animals are indicated by colored symbols. This presentation clearly shows that the observed variation represents variation between individual SCVs and not between individual animals.

### Transmission electron microscopy

Neonate mice were sacrificed 4 days p.i. The small intestinal tissue was dissected and fixed in 1% glutaraldehyde (for the ultrastructural analysis) or 4% paraformaldehyde (for immunolabeling) in 200 mM HEPES, pH 7.4 for several days. The distal end was cut into approximately 2 mm long gut sections that were subsequently longitudinally opened at one side. For the ultrastructural analysis (Fig. 1 and 2, Fig. S2 and S3), samples were stained with 1% osmium tetroxide prepared in 1.5% potassium ferricyanide for 1 h on ice, followed by 2% aqueous uranyl acetate for 2 h, and then dehydrated with a graded ethanol series. The tissues were progressively infiltrated with epon over 2 days and then polymerized in the oven overnight. Thin, 60–80 nm sections were prepared using an Ultracut UCT ultramicrotome (Leica Microsystems) and mounted onto formvar-coated copper grids. Sections were stained with lead citrate, and imaged in a JEM-1400 TEM microscope (Jeol) equipped with a TemCam-F216 camera and the EM MENU software (both Tvips). For on-section immunolabeling (Fig. S1E and F), tissue pieces were embedded in 12% bovine gelatine and infiltrated with 2.3 M sucrose overnight. Small blocks were mounted on aluminum pins and snap frozen in liquid nitrogen. A UC7 cryo-ultramicrotome (Leica Microsystems) was used to prepare Tokuyasu sections. 80 nm thin sections were cut at −110°C, and 250 nm semi-thin sections were cut at −90°C. Using a 1 + 1 mixture of 2% methyl cellulose and 2.3 M sucrose, thin sections were transferred onto formvar-coated copper grids, while semi-thin sections were transferred onto glass coverslips. For labeling, 1% fish skin gelatine was used for blocking and for dilution of labeling probes. Grids were incubated with 5x diluted LAMP-1 (rat monoclonal Ab, clone 1D4B) and LAMP-2 (rat monoclonal Ab, clone ABL-93) hybridoma supernatant (DSHB, University of Iowa) for 30 min, followed by 250x diluted rabbit anti-rat bridging antibody (Rockland) for 15 min and 50x diluted protein A gold (CMC, University Medical Centrum Utrecht) for 30 min. Sections were embedded in 0.2% uranyl acetate in 1.8% methyl cellulose. Grids were imaged in a JEM-1400 TEM microscope equipped with a TemCam-F216 camera and the EM MENU software. Glass coverslips were incubated with 10x diluted LAMP-1 antibody for 30 min, followed by goat anti-rat Cy3-conjugated secondary Ab for 30 min, and 1 µg/mL DAPI for 5 min. Coverslips were mounted on a glass slide with Mowiol 4–88, and imaged in an inverted epifluorescence Leica DMIRBE microscope, using a 60x objective lens and a Leica application Suite (LAC) software v 3.8 (both Leica Microsystems).

### Transcriptomic analysis of intraepithelial S. Typhimurium

One-day-old mice were orally infected with 10^2^ CFU wt *S*. Typhimurium carrying a mCherry reporter plasmid. An aliquot of the bacteria used for the infection was stored for subsequent analysis (inoculum). At 4 days p.i., mice were sacrificed and primary intestinal epithelial cells were isolated from small intestinal tissues as described. E-cadherin+ CD56- cells were sorted by flow cytometry in mCherry positive (*S*. Typhimurium-infected) and mCherry negative (uninfected bystander) epithelial cells. Total RNA was isolated from the bacterial inoculum as well as *S*. Typhimurium-positive enterocytes and transcriptomic analysis was performed by RNA-seq as described in Westermann et al., 2016.^42^ Notably, due to the fragility and low number of infected enterocytes (approximately 1%)^14^ in each animal and the relatively low number of intraepithelial wildtype bacteria, the FACS sorted material of seven infected litters (with approximately 49 neonates in total) had to be pooled to obtain sufficient material for sequencing of bacterial RNA. This explains why only the most abundant *S.*Typhimurium virulence factors were detected in the intraepithelial fraction and no biological replicates could be generated. Briefly, RNA samples were treated for 45 min with DNase I (NEB) to remove genomic DNA remnants and sheared via ultra-sound sonication (4 pulses of 30 s at 4°C each) to generate ~200-400 bp average fragments. Fragments <20 nt were removed using the Agencourt RNAClean XP kit (Beckman Coulter Genomics). The samples were poly(A)-tailed using poly(A) polymerase and the 5ʹ triphosphate structures were removed using tobacco acid pyrophosphatase (TAP). An RNA adapter was ligated to the 5ʹ monophosphate of the RNA fragments. First-strand cDNA synthesis was performed using an oligo(dT)-adapter primer and the M-MLV reverse transcriptase (NEB). The resulting cDNA was PCR-amplified to about 10–20 ng/µL using a high fidelity DNA polymerase (barcode sequences for multiplexing were part of the 3ʹ primers). cDNA libraries were purified using the Agencourt AMPure XP kit (Beckman Coulter Genomics) and analyzed by capillary electrophoresis (Shimadzu MultiNA microchip electrophoresis system). For sequencing, cDNA samples were pooled in approximately equimolar amounts. The cDNA pool was size-fractionated in the size range of 150–600 bp using a differential clean-up with the Agencourt AMPure kit. Single-end sequencing (100 cycles) was performed on a HiSeq2000 platform. Illumina reads in FASTQ format were trimmed with a Phred quality score cutoff of 20 using fastq_quality_trimmer from FASTX toolkit version 0.0.13 (http://hannonlab.cshl.edu/fastx_toolkit/). Reads shorter than 20 nt after adapter- and poly(A)-trimming were discarded prior to the mapping. The mapping was performed using the READemption pipeline (version 0.3.5)^43^ and the short read mapper segemehl and its remapper lack (version 0.2.0)^44^ against the published genome sequence of *S*. Typhimurium ATCC 14028 (NCBI RefSeq accession number NC_016856) and mouse genome (GENCODE M2, GRCm38.p2) in parallel. Mapped reads with an alignment accuracy <90%, murine-mapped reads as well as cross-mapped reads (i.e. reads which could be aligned equally well to the bacterial and mouse genome) were discarded and only *S*. Typhimurium-specific reads were considered for differential expression analysis. For the data presentation, the SPI1 and −2 associated genes were selected based on the detection of sequence reads in either the inoculum or intraepithelial fraction by RNA-seq and their established role and assignment to SPI1 or SPI2. For comparison of the two fractions, the gene-specific sequence reads were normalized to the total number of aligned *S*. Typhimurium reads in that library. The resulting normalized sequence read counts for SPI1 and SPI2 genes are displayed in Figure 1(a) and the respective counts for the two established house keeping genes *rpoD* and *gyrA* are shown as control.

### Generation and use of the genome-tagged S. Typhimurium libraries

22 individual strains were barcoded with a 4 nucleotide sequence tag, introducing an artificial stop codon into the endogenous *proV* gene as described previously.^23^ Equal mixtures of each barcoded strain grown separately were orally applied to 1-day-old C57BL/6 N mice in 1 µl PBS at a final concentration of 10^3^ CFU (i.e. approximately 5 × 10^1^ per strain). At 4 days p.i., the animals were sacrificed and small intestine, liver and spleen tissue were harvested. IEC isolation was performed as described above. Total spleen and liver homogenates and total IEC lysate were plated on ampicillin containing LB agar plates. After incubation at 37°C overnight, bacteria were harvested in 4 mL PBS. Bacteria were harvested by centrifugation for 10 min at 300 rpm, followed by gDNA isolation and amplification of *proV* as recently described.^25^ Samples were sequenced on an Illumina MiSeq system using MiSeq Reagent Kits (73 cycles/323 cycles) at the Center for Biotechnology (CeBiTec) of the University of Bielefeld, Germany. From a total of 22 generated sequence tags, 21 tags were recovered from the inoculum and tissue samples. For calculation of sequence identities, only barcoded strains contributing to >1% to the overall population were considered. The calculated Bray-Curtis Index (BCI) was subtracted from 1 in order to demonstrate the degree of population identity among distinct organ compartments.

### Statistical analysis

No data were excluded from the analysis. One-way ANOVA with Bonferroni´s test for multiple comparisons was employed for the statistical evaluation of organ counts. Results from individual animals plus the median are depicted. Comparison in SCV number and size between wt and SPI2-deficient bacteria was conducted using the Student's t-test (Mann Whitney test) or one-way ANOVA (with Bonferroni´s multiple posttest) for single mutant comparison and SCV formation over time with representation of median values from 3 different animals. The cellular SCV positioning was tested using a 2-way ANOVA with Sidak’s multiple comparison test. Cell culture results show individual measurements obtained in at least three independent experiments and the median. They were analyzed by one-way ANOVA with Dunnett’s multiple comparisons test. The GraphPad Prism Software 8.0 was used for statistical evaluation. *p* values are indicated in the figures.

## Supplementary Material

Supplemental Material

## Data Availability

The authors declare that all data supporting the findings of this study are available within the paper and its supplementary information files.
