# Supplementary material for: SPI2 T3SS effectors facilitate enterocyte apical to basolateral transmigration of Salmonella-containing vacuoles in vivo
Source: Gut Microbes. 2021 Sep 20;13(1):1973836. doi: 10.1080/19490976.2021.1973836 (PMC8475570; doi:10.1080/19490976.2021.1973836)
Supplement: Supplemental Material [file KGMI_A_1973836_SM0539.zip › supp/downloadFromZipFile,.pdf]

Fulde et al., Supplemental Table 1: **Strains and plasmids used in this study**

strain name and description	reference
ATCC 14028::pFPV-25.1	Valdivia *et al.*, 1998, Zhang *et al.*, 2014
MvP643 Δ*sseB*::FRT pFPV-25.1	this study
MvP643 (p3232)	Wang and Kushner, 1991
MvP643 Δ*sseB*::FRT Δ*dpp* Δ*opp* Δ*ydgR* Δ*ygdR* Δ*yhiP* pFPV-25.1	Strugnell *et al.*, 2014
MvP643 Δ*sseB*::FRT Δ*glk* Δ*manXYZ* Δ*ptsG* Δ*uhpT* pFPV-25.1	Strugnell *et al.*, 2014
MvP643 Δ*sseB*::FRT Δ*aroP* Δ*pheP* Δ*tyrP* Δ*mtr* Δ*pheA* pFPV-25.1	Strugnell *et al.*, 2014
MvP643 Δ*sseB*::FRT Δ*fadL* pFPV-25.1	Strugnell *et al.*, 2014
MvP388 Δ*sseF* Δ*sseG* pFPV-25.1	Hansen-Wester *et al.*, 2002; Deiwick *et al.*, 2006
MvP497 Δ*sifA*::aph pFPV-25.1	Beuzón *et al.*, 2000; Noster *et al.*, 2019
MvP498 Δ*pipB2*::aph pGFP	Knodler *et al.*, 2003
MvP1604 Δ*ssaN*::aph	this study
MvP1604 (p3362)	this study
MvP818 Δ*invC*::FRT pFPV-25.1	Zhang *et al.*, 2014
MvP818 (p3545)	Zhang *et al.*, 2014
MvP643 Δ*sseG*::aphT psseFG::M45	Kuhle und Hensel, 2006
MvP643 Δ*sseB*::FRT pFPV-25.1 mCherry	Wrande *et al.*, 2016

plasmid	description	reference
pssaG::GFP	SPI2 (ssaG) reporter plasmid	Hapfelmeier *et al.*, 2005
pFPV25.1	amp P_{rpsM}::gfpmut3	Valdivia *et al.*, 1998
pWSK29	Amp lopw copy number	Wang and Kushner, 1991
3232	amp pWSK29::sseB	Hölzer and Hensel, 2010
p3545	amp P_{invF}::invC	Zhang *et al.*, 2014
pFPV-mCherry	amp P_{rpsM}::mCherry	Drecktrah *et al.*, 2008
p3362	amp pWSK29::ssaN	this study
