# Supplementary figures and images for: SPI2 T3SS effectors facilitate enterocyte apical to basolateral transmigration of Salmonella-containing vacuoles in vivo
Source: Gut Microbes. 2021 Sep 20;13(1):1973836. doi: 10.1080/19490976.2021.1973836 (PMC8475570; doi:10.1080/19490976.2021.1973836)

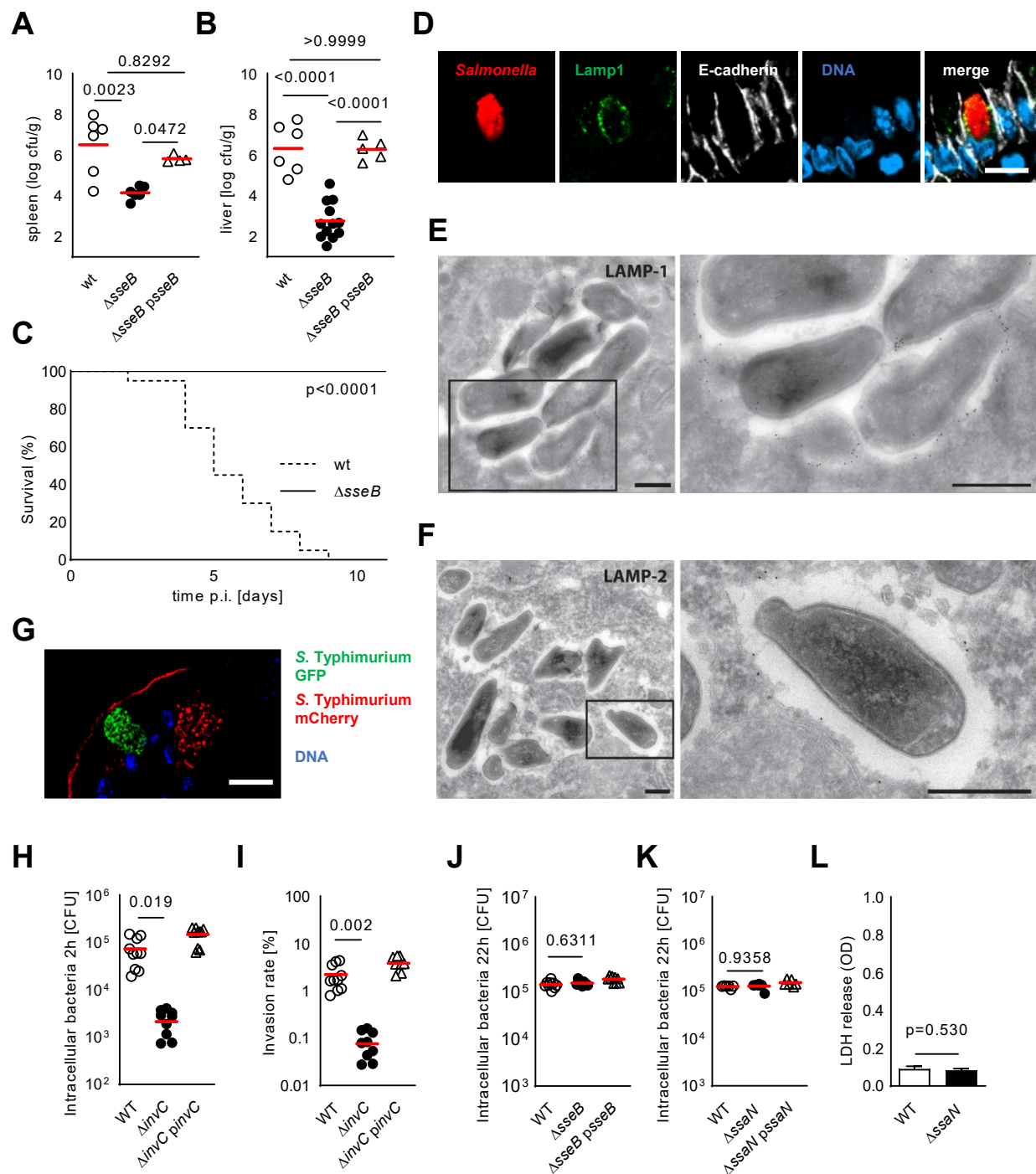

Supplement: Supplemental Material [file KGMI_A_1973836_SM0539.zip › supp/downloadFromZipFile.pdf]

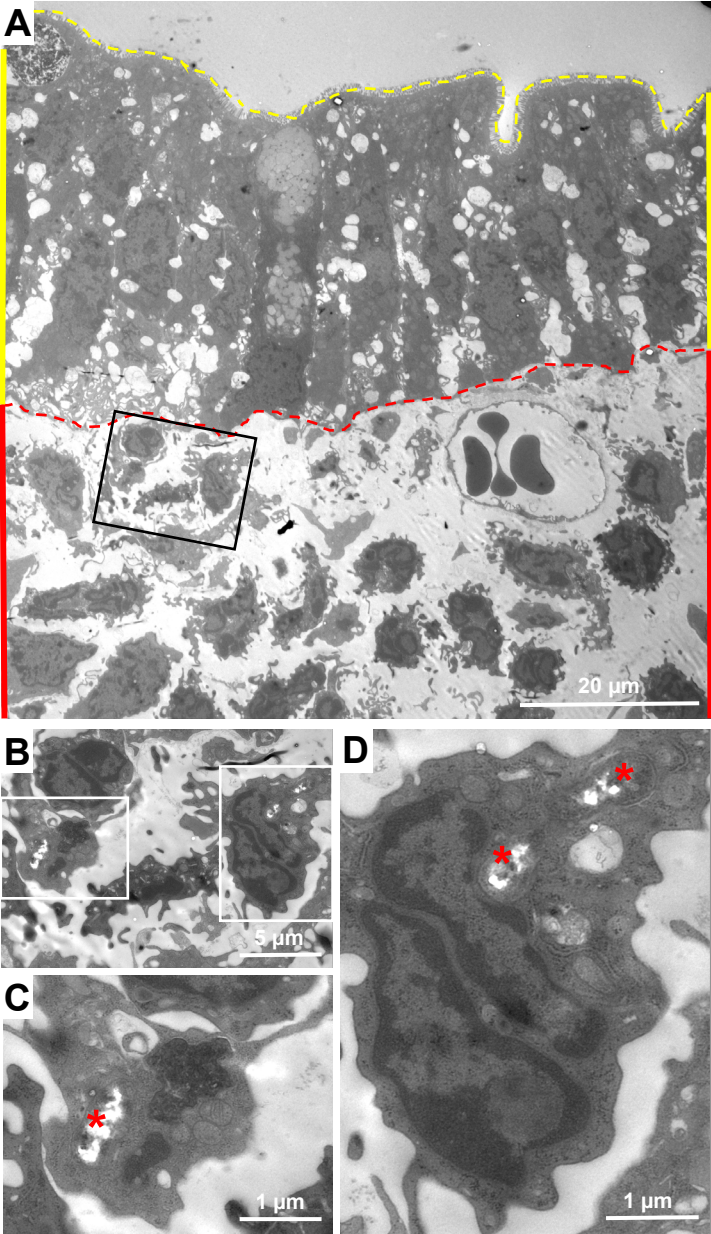

Supplement: Supplemental Material [file KGMI_A_1973836_SM0539.zip › supp/downloadFromZipFile11.pdf]

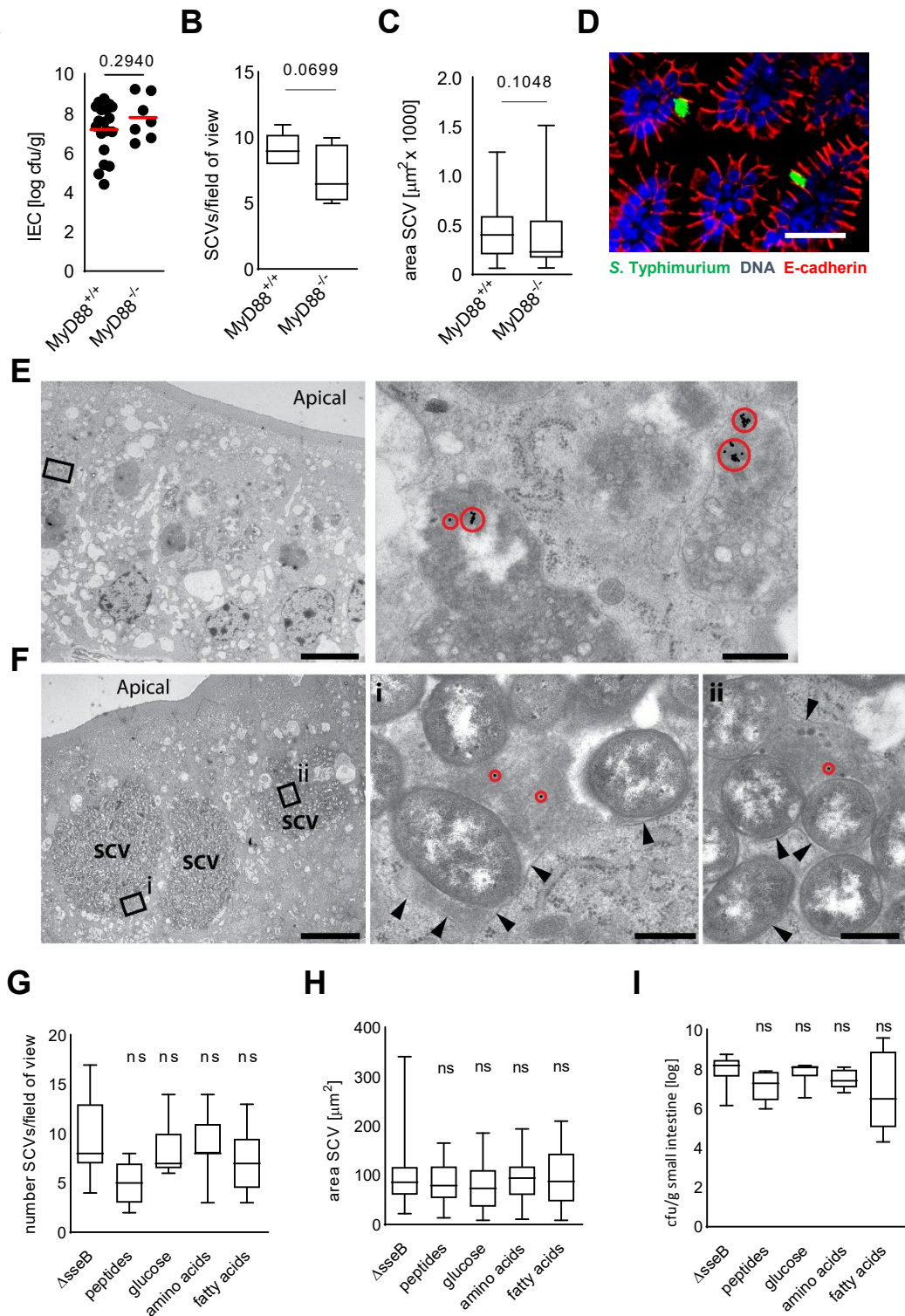

Supplement: Supplemental Material [file KGMI_A_1973836_SM0539.zip › supp/downloadFromZipFile2.pdf]
