# Supplementary material for: SPI2 T3SS effectors facilitate enterocyte apical to basolateral transmigration of Salmonella-containing vacuoles in vivo
Source: Gut Microbes. 2021 Sep 20;13(1):1973836. doi: 10.1080/19490976.2021.1973836 (PMC8475570; doi:10.1080/19490976.2021.1973836)
Supplement: Supplemental Material [file downloadFromZipFile...pdf › supp/downloadFromZipFile...pdf]

Sequence r	Source	Feature	Start	End	Strand	Frame	Attributes	Salmonella_plus IE	Salmonella_inoculum_re	Locus_tag	Gene	Product	baseMean	log2FoldChange
NC_01685!	RefSeq	CDS	94	378	+	0	ID=cds0;Na	0	6	STM14_5526	STM14_5526	putative cytoplasmic protein	0,852600993	-1,98056697
NC_01685!	RefSeq	CDS	1333	1599	+	0	ID=cds1;Na	2	68,5	STM14_5528	repC	DNA replication	13,181456	-0,982976487
NC_01685!	RefSeq	CDS	1722	1844	+	0	ID=cds2;Na	19	18,08333333	STM14_5529	repA3	DNA replication	35,98492801	2,485782757
NC_01685!	RefSeq	CDS	1849	1926	+	0	ID=cds3;Na	0	7,833333333	STM14_5530	tap	replication of plasmid	1,136801324	-2,147403465
NC_01685!	RefSeq	CDS	1907	2788	+	0	ID=cds4;Na	0	23,08333333	STM14_5531	repA	DNA replication	3,268303807	-2,432747446
NC_01685!	RefSeq	CDS	3729	3869	+	0	ID=cds5;Na	0	0	STM14_5532	STM14_5532	putative outer membrane protein	0	NA
NC_01685!	RefSeq	CDS	3966	4724	-	0	ID=cds6;Na	0	1	STM14_5533	srgC	putative bacterial regulatory helix-turn-helix protein%2C a	0,142100166	-0,623264422
NC_01685!	RefSeq	CDS	4991	5548	-	0	ID=cds7;Na	0	5	STM14_5534	rcK	resistance to complement killing	0,710500828	-1,864806942
NC_01685!	RefSeq	CDS	5638	6537	-	0	ID=cds8;Na	0	3	STM14_5535	srgB	putative outer membrane protein	0,426300497	-1,498211557
NC_01685!	RefSeq	CDS	6670	7323	-	0	ID=cds9;Na	0	0	STM14_5536	srgA	putative thiol-disulfide isomerase or thioredoxin	0	NA
NC_01685!	RefSeq	CDS	7568	7918	-	0	ID=cds10;N	0	4	STM14_5537	orf7	putative bacterial regulatory protein%2C luxR family	0,568400662	-1,710438693
NC_01685!	RefSeq	CDS	7903	8115	-	0	ID=cds11;N	0	2	STM14_5538	pefI	plasmid-encoded fimbriae%3B regulatory	0,284200331	-1,182723423
NC_01685!	RefSeq	CDS	8547	9422	-	0	ID=cds12;N	1	36	STM14_5539	orf6	putative outer membrane protein	6,874928329	-0,96718351
NC_01685!	RefSeq	CDS	9675	10232	-	0	ID=cds13;N	0	1,5	STM14_5540	orf5	putative outer membrane protein	0,284200331	-1,182723423
NC_01685!	RefSeq	CDS	10247	10939	-	0	ID=cds14;N	0	0,5	STM14_5541	pefD	plasmid-encoded fimbriae%3Bchaperone	0	NA
NC_01685!	RefSeq	CDS	10932	13340	-	0	ID=cds15;N	0	1,5	STM14_5542	pefC	plasmid-encoded fimbriae%3B usher protein	0,284200331	-1,182723423
NC_01685!	RefSeq	CDS	13568	14086	-	0	ID=cds16;N	0	3	STM14_5543	pefA	plasmid-encoded fimbriae%3B major fimbrial subunit	0,426300497	-1,498211557
NC_01685!	RefSeq	CDS	14361	14663	-	0	ID=cds17;N	0	0	STM14_5544	pefB	plasmid-encoded fimbriae%3Bregulation	0	NA
NC_01685!	RefSeq	CDS	15560	15763	+	0	ID=cds18;N	0	1,5	STM14_5545	STM14_5545	hypothetical protein	0,284200331	-1,182723423
NC_01685!	RefSeq	CDS	16635	17624	-	0	ID=cds19;N	0	40,75	STM14_5546	repA2	DNA replication	5,826106787	-2,241137745
NC_01685!	RefSeq	CDS	17368	17676	+	0	ID=cds20;N	0	0	STM14_5547	STM14_5547	hypothetical protein	0	NA
NC_01685!	RefSeq	CDS	18118	18414	-	0	ID=cds21;N	0	3	STM14_5548	STM14_5548	putative cytoplasmic protein	0,426300497	-1,498211557
NC_01685!	RefSeq	CDS	18898	19419	-	0	ID=cds22;N	1	8	STM14_5549	STM14_5549	hypothetical protein	2,896123694	0,419298367
NC_01685!	RefSeq	CDS	20095	20313	+	0	ID=cds23;N	0	14,5	STM14_5550	ccdA	toxin addiction system: antidote	1,989402318	-2,376535557
NC_01685!	RefSeq	CDS	20315	20620	+	0	ID=cds24;N	0	7	STM14_5551	ccdB	toxin addiction system: toxin	0,994701159	-2,072978628
NC_01685!	RefSeq	CDS	20622	20912	+	0	ID=cds25;N	0	4	STM14_5552	STM14_5552	putative cytoplasmic protein	0,568400662	-1,710438693
NC_01685!	RefSeq	CDS	20909	21430	+	0	ID=cds26;N	0	10,5	STM14_5553	STM14_5553	putative cytoplasmic protein	1,421001655	-2,256999172
NC_01685!	RefSeq	CDS	21465	22247	+	0	ID=cds27;N	0	9,5	STM14_5554	rsdB	resolvase	1,421001655	-2,256999172
NC_01685!	RefSeq	CDS	22256	22807	+	0	ID=cds28;N	0	0	STM14_5555	STM14_5555	diguanylate cyclase	0	NA
NC_01685!	RefSeq	CDS	23476	23961	+	0	ID=cds29;N	0	7	STM14_5556	STM14_5556	putative inner membrane protein	0,994701159	-2,072978628
NC_01685!	RefSeq	CDS	24681	25241	+	0	ID=cds30;N	0	0	STM14_5557	STM14_5557	putative adhesin	0	NA
NC_01685!	RefSeq	CDS	25308	25658	-	0	ID=cds31;N	0	71,5	STM14_5558	STM14_5558	putative transposase	10,23121192	-2,035392961
NC_01685!	RefSeq	CDS	25876	26139	+	0	ID=cds32;N	0	5	STM14_5559	STM14_5559	putative transposase%2C IS200-like protein	0,710500828	-1,864806942
NC_01685!	RefSeq	CDS	26266	26916	-	0	ID=cds33;N	0	0	STM14_5560	spvD	hydrophilic protein	0	NA
NC_01685!	RefSeq	CDS	27177	27902	-	0	ID=cds34;N	0	0	STM14_5561	spvC	hydrophilic protein	0	NA
NC_01685!	RefSeq	CDS	28183	29958	-	0	ID=cds35;N	0	1,5	STM14_5562	spvB	hydrophilic protein	0,284200331	-1,182723423
NC_01685!	RefSeq	CDS	30140	30907	-	0	ID=cds36;N	0	0	STM14_5563	spvA	outer membrane protein	0	NA
NC_01685!	RefSeq	CDS	31419	32312	-	0	ID=cds37;N	0	0	STM14_5564	spvR	regulation of spv operon%2C lysR family	0	NA
NC_01685!	RefSeq	CDS	32921	33958	+	0	ID=cds38;N	0	0,5	STM14_5565	STM14_5565	putative integrase protein	0	NA
NC_01685!	RefSeq	CDS	34147	35172	-	0	ID=cds39;N	0	61,75	STM14_5566	STM14_5566	putative phosphoribulokinase %2F uridine kinase family	8,810210263	-2,091539774
NC_01685!	RefSeq	CDS	35105	36769	-	0	ID=cds40;N	1	83,25	STM14_5567	STM14_5567	putative integrase protein	13,55363611	-1,777695363
NC_01685!	RefSeq	CDS	36753	37313	-	0	ID=cds41;N	1	42,5	STM14_5568	rlgA	putative resolvase	7,727529322	-1,109707982
NC_01685!	RefSeq	CDS	37520	38260	-	0	ID=cds42;N	1	135,5	STM14_5569	STM14_5569	putative carbonic anhydrase	21,08494488	-2,233756807
NC_01685!	RefSeq	CDS	38915	39403	-	0	ID=cds43;N	0	4	STM14_5570	STM14_5570	putative cytoplasmic protein	0,568400662	-1,710438693
NC_01685!	RefSeq	CDS	39662	40777	+	0	ID=cds44;N	0	3	STM14_5571	tlpA	alpha-helical coiled coil protein	0,426300497	-1,498211557
NC_01685!	RefSeq	CDS	40863	41279	+	0	ID=cds45;N	0	3	STM14_5572	STM14_5572	putative DNA polymerase III epsilon subunit %283%27-5%	0,426300497	-1,498211557
NC_01685!	RefSeq	CDS	40973	41299	-	0	ID=cds46;N	0	1,5	STM14_5573	STM14_5573	putative cytoplasmic protein	0,284200331	-1,182723423
NC_01685!	RefSeq	CDS	42453	43394	-	0	ID=cds47;N	0	41,5	STM14_5574	STM14_5574	putative cytoplasmic protein	5,968206953	-2,232890319
NC_01685!	RefSeq	CDS	43809	45014	+	0	ID=cds48;N	0	11	STM14_5575	parA	plasmid partition protein A	1,563101821	-2,297202414
NC_01685!	RefSeq	CDS	45011	45988	+	0	ID=cds49;N	0	22	STM14_5576	parB	plasmid partition protein B	3,126203642	-2,431590384
NC_01685!	RefSeq	CDS	46070	47344	-	0	ID=cds50;N	0	1,5	STM14_5577	samB	SamB	0,284200331	-1,182723423
NC_01685!	RefSeq	CDS	47344	47766	-	0	ID=cds51;N	1	1,5	STM14_5578	samA	SamA	2,043522701	1,824239109
NC_01685!	RefSeq	CDS	48277	48747	+	0	ID=cds52;N	0	0	STM14_5579	STM14_5579	putative cytoplasmic protein	0	NA
NC_01685!	RefSeq	CDS	48740	49096	+	0	ID=cds53;N	0	0	STM14_5580	STM14_5580	putative cytoplasmic protein	0	NA
NC_01685!	RefSeq	CDS	49478	50158	+	0	ID=cds54;N	0	0	STM14_5581	STM14_5581	putative adenine-specific DNA methylase	0	NA
NC_01685!	RefSeq	CDS	50158	50379	+	0	ID=cds55;N	0	1	STM14_5582	STM14_5582	putative cytoplasmic protein	0,142100166	-0,623264422
NC_01685!	RefSeq	CDS	50920	51684	+	0	ID=cds56;N	0	0,5	STM14_5583	STM14_5583	putative inner membrane protein	0	NA
NC_01685!	RefSeq	CDS	52180	52614	+	0	ID=cds57;N	0	0,5	STM14_5584	STM14_5584	putative cytoplasmic protein	0	NA
NC_01685!	RefSeq	CDS	52681	53115	+	0	ID=cds58;N	0	0	STM14_5585	STM14_5585	putative cytoplasmic protein	0	NA
NC_01685!	RefSeq	CDS	53351	53674	+	0	ID=cds59;N	0	1	STM14_5586	STM14_5586	putative inner membrane protein	0,142100166	-0,623264422
NC_01685!	RefSeq	CDS	54375	54893	+	0	ID=cds60;N	0	0	STM14_5587	ssbB	single-strand binding protein	0	NA
NC_01685!	RefSeq	CDS	54948	55190	+	0	ID=cds61;N	0	0	STM14_5588	STM14_5588	putative cytoplasmic protein	0	NA
NC_01685!	RefSeq	CDS	55259	57256	+	0	ID=cds62;N	0	5,5	STM14_5589	STM14_5589	putative ParB-like nuclease domain protein	0,852600993	-1,98056697
NC_01685!	RefSeq	CDS	57296	57730	+	0	ID=cds63;N	0	4	STM14_5590	psiB	Plasmid SOS inhibition	0,568400662	-1,710438693
NC_01685!	RefSeq	CDS	57727	58464	+	0	ID=cds64;N	0	0	STM14_5591	psiA	Plasmid SOS inhibition	0	NA
NC_01685!	RefSeq	CDS	60103	60573	-	0	ID=cds65;N	0	4	STM14_5592	finP	putative transglycosylase	0,568400662	-1,710438693
NC_01685!	RefSeq	CDS	60988	61368	+	0	ID=cds66;N	0	1	STM14_5593	traM	conjugative transfer: mating signal	0,142100166	-0,623264422
NC_01685!	RefSeq	ncRNA	61461	61628	-	.	ID=rna0;Pa	0	0,666666667	STM14_5594	STM14_5594	-	0,142100166	-0,623264422
NC_01685!	RefSeq	CDS	61561	62247	+	0	ID=cds67;N	0	0	STM14_5595	traJ	conjugative transfer: regulation	0	NA
NC_01685!	RefSeq	CDS	62248	62568	+	0	ID=cds68;N	0	1	STM14_5596	traY	conjugative transfer: oriT nicking	0,142100166	-0,623264422
NC_01685!	RefSeq	CDS	62610	62972	+	0	ID=cds69;N	0	5	STM14_5597	traA	conjugative transfer: fimbrial subunit	0,710500828	-1,864806942
NC_01685!	RefSeq	CDS	62987	63298	+	0	ID=cds70;N	0	1	STM14_5598	traL	conjugative transfer: assembly	0,142100166	-0,623264422
NC_01685!	RefSeq	CDS	63320	63886	+	0	ID=cds71;N	0	0,5	STM14_5599	traE	conjugative transfer: assembly	0	NA
NC_01685!	RefSeq	CDS	63873	64613	+	0	ID=cds72;N	0	0,5	STM14_5600	traK	conjugative transfer: assembly	0	NA
NC_01685!	RefSeq	CDS	64613	66034	+	0	ID=cds73;N	0	1	STM14_5601	traB	conjugative transfer: assembly	0,142100166	-0,623264422
NC_01685!	RefSeq	CDS	66024	66620	+	0	ID=cds74;N	0	1	STM14_5602	traP	conjugative transfer protein	0,142100166	-0,623264422
NC_01685!	RefSeq	CDS	66598	66843	+	0	ID=cds75;N	0	0	STM14_5603	trbD	conjugative transfer protein	0	NA
NC_01685!	RefSeq	CDS	66840	67355	+	0	ID=cds76;N	0	0	STM14_5604	traV	conjugative transfer: assembly	0	NA
NC_01685!	RefSeq	CDS	67490	67711	+	0	ID=cds77;N	0	0	STM14_5605	traR	conjugative transfer protein	0	NA
NC_01685!	RefSeq	CDS	67704	68171	+	0	ID=cds78;N	0	1	STM14_5606	STM14_5606	conjugative transfer protein	0,142100166	-0,623264422
NC_01685!	RefSeq	CDS	68567	71215	+	0	ID=cds79;N	0	4	STM14_5607	traC	conjugative transfer: assembly	0,568400662	-1,710438693
NC_01685!	RefSeq	CDS	71190	71576	+	0	ID=cds80;N	0	0,5	STM14_5608	trbI	conjugative transfer protein	0	NA
NC_01685!	RefSeq	CDS	71573	72205	+	0	ID=cds81;N	0	4	STM14_5609	traW	conjugative transfer: assembly	0,568400662	-1,710438693
NC_01685!	RefSeq	CDS	72202	73194	+	0	ID=cds82;N	0	2	STM14_5610	traU	conjugative transfer: assembly	0,284200331	-1,182723423
NC_01685!	RefSeq	CDS	73213	73521	+	0	ID=cds83;N	0	0	STM14_5611	STM14_5611	conjugative transfer protein	0	NA
NC_01685!	RefSeq	CDS	73506	74144	+	0	ID=cds84;N	0	0	STM14_5612	trbC	conjugative transfer: assembly	0	NA
NC_01685!	RefSeq	CDS	74597	76423	+	0	ID=cds85;N	0	4	STM14_5613	traN	conjugative transfer: aggregate stability	0,568400662	-1,710438693
NC_01685!	RefSeq	CDS	76434	76649	+	0	ID=cds86;N	0	0	STM14_5614	trbE	conjugative transfer protein	0	NA
NC_01685!	RefSeq	CDS	76661	77410	+	0	ID=cds87;N	0	0	STM14_5615	traF	conjugative transfer: assembly	0	NA
NC_01685!	RefSeq	CDS	77624	77917	+	0	ID=cds88;N	0	1	STM14_5616	traQ	conjugative transfer: fimbrial synthesis	0,142100166	-0,623264422
NC_01685!	RefSeq	CDS	77904	78452	+	0	ID=cds89;N	0	0	STM14_5617	trbB	conjugative transfer protein	0	NA
NC_01685!	RefSeq	CDS	78442	79824	+	0	ID=cds90;N	0	1	STM14_5618	traH	conjugative transfer: assembly	0,142100166	-0,623264422
NC_01685!	RefSeq	CDS	79821	82643	+	0	ID=cds91;N	0	21,5	STM14_5619	traG	conjugative transfer: assembly abd aggregate stability	3,126203642	-2,431590384
NC_01685!	RefSeq	CDS	82659	83153	+	0	ID=cds92;N	3	43,5	STM14_5620	traS	conjugative transfer: surface exclusion	11,53037439	-0,165957205
NC_01685!	RefSeq	CDS	83189	83920	+	0	ID=cds93;N	0	107,5	STM14_5621	traT	conjugative transfer: surface exclusion	15,34681788	-1,876042944
NC_01685!	RefSeq	CDS	84297	86459	+	0	ID=cds94;N	0	8	STM14_5622	traD	conjugative transfer: DNA transport	1,136801324	-2,147403465
NC_01685!	RefSeq	CDS	86459	87178	+	0	ID=cds95;N	0	1,5	STM14_5623	trbH	conjugative transfer protein	0,284200331	-1,182723423
NC_01685!	RefSeq	CDS	86468	86866	-	0	ID=cds96;N	0	4,333333333	STM14_5624	STM14_5624	MvpA-like protein	0,568400662	-1,710438693
NC_01685!	RefSeq	CDS	86866	87096	-	0	ID=cds97;N	0	6	STM14_5625	STM14_5625	putative cytoplasmic protein	0,852600993	-1,98056697
NC_01685!	RefSeq	CDS	87175	92433	+	0	ID=cds98;N	0	14	STM14_5626	traI	conjugative transfer: oriT nicking-unwinding	1,989402318	-2,376535557
NC_01685!	RefSeq	CDS	92453	93193	+	0	ID=cds99;N	0	2	STM14_5627	traX	conjugative transfer: fimbrial acetylation	0,284200331	-1,182723423
NC_01685!	RefSeq	CDS	93248	93811	+	0	ID=cds100;	0	8,5	STM14_5628	finO	conjugative transfer: regulation	1,136801324	-2,147403465
NC_01685(	RefSeq	CDS	190	255	+	0	ID=cds0;Na	0	9	STM14_0001	thrL	thr operon leader peptide	1,27890149	-2,207741391
NC_01685(	RefSeq	CDS	337	2799	+	0	ID=cds1;Na	0	11	STM14_0002	thrA	bifunctional aspartokinase I%2Fhomoserine dehydrogena:	1,563101821	-2,297202414
NC_01685(	RefSeq	CDS	2801	3730	+	0	ID=cds2;Na	1	10,5	STM14_0003	thrB	homoserine kinase	3,180324025	0,201203536
NC_01685(	RefSeq	CDS	3734	5020	+	0	ID=cds3;Na	0	22,5	STM14_0004	thrC	threonine synthase	3,126203642	-2,431590384
NC_01685(	RefSeq	CDS	5114	5887	-	0	ID=cds4;Na	0	11,5	STM14_0005	yaaA	hypothetical protein	1,705201986	-2,329925387
NC_01685(	RefSeq	CDS	5966	7396	-	0	ID=cds5;Na	3	12	STM14_0006	yaaJ	putative alanine%2Fglycine transport protein	6,983169096	1,056079994
NC_01685(	RefSeq	CDS	7665	8618	+	0	ID=cds6;Na	1	198,8333333	STM14_0007	talB	transaldolase B	30,03725531	-2,550932814
NC_01685(	RefSeq	CDS	8729	9319	+	0	ID=cds7;Na	0	33	STM14_0008	mogA	molybdenum cofactor biosynthesis protein	4,689305463	-2,505858154
NC_01685(	RefSeq	CDS	9376	9942	-	0	ID=cds8;Na	0	3	STM14_0009	yaaH	hypothetical protein	0,426300497	-1,498211557
NC_01685(	RefSeq	CDS	10092	10805	-	0	ID=cds9;Na	0	0	STM14_0010	htgA	hypothetical protein	0	NA
NC_01685(	RefSeq	CDS	10841	11245	-	0	ID=cds10;N	0	0	STM14_0011	yaaI	hypothetical protein	0	NA
NC_01685(	RefSeq	CDS	11257	11424	+	0	ID=cds11;N	0	0	STM14_0012	STM14_0012	hypothetical protein	0	NA
NC_01685(	RefSeq	CDS	11593	13509	+	0	ID=cds12;N	0	40,5	STM14_0013	dnaK	molecular chaperone DnaK	5,684006621	-2,24950002
NC_01685(	RefSeq	CDS	13595	14734	+	0	ID=cds13;N	5	18	STM14_0014	dnaJ	chaperone protein DnaJ	11,35441483	1,202027933
NC_01685(	RefSeq	CDS	14845	14958	+	0	ID=cds14;N	0	0	STM14_0015	STM14_0015	hypothetical protein	0	NA
NC_01685(	RefSeq	CDS	15014	15961	+	0	ID=cds15;N	0	0	STM14_0016	STM14_0016	putative transcriptional regulator	0	NA
NC_01685(	RefSeq	CDS	16088	16432	+	0	ID=cds16;N	0	0	STM14_0017	STM14_0017	putative bacteriophage protein	0	NA
NC_01685(	RefSeq	CDS	16493	17026	-	0	ID=cds17;N	0	0	STM14_0018	STM14_0018	hypothetical protein	0	NA
NC_01685(	RefSeq	CDS	17043	17486	-	0	ID=cds18;N	0	1	STM14_0019	STM14_0019	hypothetical protein	0,142100166	-0,623264422
NC_01685(	RefSeq	CDS	17613	17741	-	0	ID=cds19;N	0	0	STM14_0020	STM14_0020	hypothetical protein	0	NA
NC_01685(	RefSeq	CDS	17665	17787	+	0	ID=cds20;N	0	0	STM14_0021	STM14_0021	hypothetical protein	0	NA

NC_01685(	RefSeq	CDS	17867	19966	+	0	ID=cds21;N	0	4,5	STM14_0022	STM14_0022	putative exochitinase	0,568400662	-1,710438693
NC_01685(	RefSeq	CDS	20058	23054	+	0	ID=cds22;N	0	2	STM14_0023	STM14_0023	putative hydroxymethyltransferase	0,284200331	-1,182723423
NC_01685(	RefSeq	CDS	23091	23240	+	0	ID=cds23;N	0	0	STM14_0024	STM14_0024	hypothetical protein	0	NA
NC_01685(	RefSeq	CDS	23335	24039	+	0	ID=cds24;N	0	1	STM14_0025	STM14_0025	putative cytoplasmic protein	0,142100166	-0,623264422
NC_01685(	RefSeq	CDS	24094	24198	-	0	ID=cds25;N	0	0	STM14_0026	STM14_0026	hypothetical protein	0	NA
NC_01685(	RefSeq	CDS	24316	24426	+	0	ID=cds26;N	0	0	STM14_0027	STM14_0027	hypothetical protein	0	NA
NC_01685(	RefSeq	CDS	24469	25011	+	0	ID=cds27;N	0	0	STM14_0028	bcfA	fimbrial subunit	0	NA
NC_01685(	RefSeq	CDS	25112	25798	+	0	ID=cds28;N	0	0	STM14_0029	bcfB	fimbrial chaperone	0	NA
NC_01685(	RefSeq	CDS	25803	28424	+	0	ID=cds29;N	0	0	STM14_0030	bcfC	fimbrial usher	0	NA
NC_01685(	RefSeq	CDS	28425	29432	+	0	ID=cds30;N	0	1	STM14_0031	bcfD	fimbrial subunit	0,142100166	-0,623264422
NC_01685(	RefSeq	CDS	29433	29978	+	0	ID=cds31;N	0	0	STM14_0032	bcfE	fimbrial subunit	0	NA
NC_01685(	RefSeq	CDS	29994	30512	+	0	ID=cds32;N	0	0,5	STM14_0033	bcfF	fimbrial subunit	0	NA
NC_01685(	RefSeq	CDS	30478	31209	+	0	ID=cds33;N	0	0	STM14_0034	bcfG	fimbrial chaperone	0	NA
NC_01685(	RefSeq	CDS	31274	32119	+	0	ID=cds34;N	0	3,5	STM14_0035	bcfH	putative thiol-disulfide isomerase	0,568400662	-1,710438693
NC_01685(	RefSeq	CDS	32116	32445	+	0	ID=cds35;N	0	1,5	STM14_0036	STM14_0036	hypothetical protein	0,284200331	-1,182723423
NC_01685(	RefSeq	CDS	32545	32994	-	0	ID=cds36;N	0	0	STM14_0037	STM14_0037	putative transcriptional regulator	0	NA
NC_01685(	RefSeq	CDS	33364	34368	+	0	ID=cds37;N	0	0	STM14_0038	STM14_0038	putative transcriptional regulator	0	NA
NC_01685(	RefSeq	CDS	34376	34816	-	0	ID=cds38;N	0	0	STM14_0039	STM14_0039	putative transcriptional regulator	0	NA
NC_01685(	RefSeq	CDS	34828	34974	+	0	ID=cds39;N	0	0	STM14_0040	STM14_0040	hypothetical protein	0	NA
NC_01685(	RefSeq	CDS	35339	37057	+	0	ID=cds40;N	0	1	STM14_0041	STM14_0041	putative arylsulfatase	0,142100166	-0,623264422
NC_01685(	RefSeq	CDS	37103	38674	-	0	ID=cds41;N	0	0,5	STM14_0042	STM14_0042	putative 5%27-nucleotidase	0	NA
NC_01685(	RefSeq	CDS	38773	39534	-	0	ID=cds42;N	0	1	STM14_0043	STM14_0043	putative outer membrane%2Fexported protein	0,142100166	-0,623264422
NC_01685(	RefSeq	CDS	40131	41624	+	0	ID=cds43;N	0	7	STM14_0044	STM14_0044	putative arylsulfatase	0,994701159	-2,072978628
NC_01685(	RefSeq	CDS	41723	42913	+	0	ID=cds44;N	0	2	STM14_0045	STM14_0045	putative arylsulfatase regulator	0,284200331	-1,182723423
NC_01685(	RefSeq	CDS	42932	44185	+	0	ID=cds45;N	0	0	STM14_0046	STM14_0046	putative cytoplasmic protein	0	NA
NC_01685(	RefSeq	CDS	44312	46027	+	0	ID=cds46;N	0	1	STM14_0047	STM14_0047	putative arylsulfatase	0,142100166	-0,623264422
NC_01685(	RefSeq	CDS	46190	47356	+	0	ID=cds47;N	0	15,5	STM14_0048	nhaA	pH-dependent sodium%2Fproton antiporter	2,273602649	-2,403729808
NC_01685(	RefSeq	CDS	47418	48317	+	0	ID=cds48;N	0	10,5	STM14_0049	nhaR	transcriptional activator NhaR	1,421001655	-2,256999172
NC_01685(	RefSeq	CDS	48372	50411	-	0	ID=cds49;N	0	2	STM14_0050	STM14_0050	putative glycosyl hydrolase	0,284200331	-1,182723423
NC_01685(	RefSeq	CDS	50451	51824	-	0	ID=cds50;N	0	0	STM14_0051	STM14_0051	putative sodium galactoside symporter	0	NA
NC_01685(	RefSeq	CDS	52280	52543	-	0	ID=cds51;N	4	164,5	STM14_0052	rpsT	30S ribosomal protein S20	30,34171663	-1,188893439
NC_01685(	RefSeq	CDS	52649	52864	+	0	ID=cds52;N	0	0,5	STM14_0053	yaaY	putative cytoplasmic protein	0	NA
NC_01685(	RefSeq	CDS	52872	53810	+	0	ID=cds53;N	0	26,5	STM14_0054	ribF	bifunctional riboflavin kinase%2FFMN adenylyltransferase	3,694604304	-2,438835558
NC_01685(	RefSeq	CDS	53855	56689	+	0	ID=cds54;N	2	185,5	STM14_0055	ileS	isoleucyl-tRNA synthetase	29,94927553	-1,940103003
NC_01685(	RefSeq	CDS	56689	57189	+	0	ID=cds55;N	3	31	STM14_0056	lspA	lipoprotein signal peptidase	9,683072241	0,174550408
NC_01685(	RefSeq	CDS	57203	57334	+	0	ID=cds56;N	0	17	STM14_0057	STM14_0057	hypothetical protein	2,415702814	-2,412509737
NC_01685(	RefSeq	CDS	57344	57793	+	0	ID=cds57;N	0	6,5	STM14_0058	slpA	FKBP-type peptidyl-prolyl cis-trans isomerase	0,852600993	-1,98056697
NC_01685(	RefSeq	CDS	57796	58746	+	0	ID=cds58;N	3	28	STM14_0059	ispH	4-hydroxy-3-methylbut-2-enyl diphosphate reductase	9,256771745	0,271721137
NC_01685(	RefSeq	CDS	58946	60148	+	0	ID=cds59;N	0	7,5	STM14_0060	STM14_0060	putative nitrite reductase	1,136801324	-2,147403465
NC_01685(	RefSeq	CDS	60164	61084	+	0	ID=cds60;N	0	12,5	STM14_0061	rihC	ribonucleoside hydrolase RihC	1,705201986	-2,329925387
NC_01685(	RefSeq	CDS	61106	61792	-	0	ID=cds61;N	0	1	STM14_0062	STM14_0062	putative transcriptional regulator	0,142100166	-0,623264422
NC_01685(	RefSeq	CDS	61794	63413	-	0	ID=cds62;N	0	1,5	STM14_0063	STM14_0063	putative transcriptional regulator	0,284200331	-1,182723423
NC_01685(	RefSeq	CDS	63548	64849	-	0	ID=cds63;N	0	1,5	STM14_0064	STM14_0064	putative oxalacetate decarboxylase subunit beta	0,284200331	-1,182723423
NC_01685(	RefSeq	CDS	64862	66637	-	0	ID=cds64;N	0	0,333333333	STM14_0065	STM14_0065	pyruvate carboxylase subunit B	0	NA
NC_01685(	RefSeq	CDS	66654	66893	-	0	ID=cds65;N	0	0	STM14_0066	STM14_0066	oxaloacetate decarboxylase subunit gamma	0	NA
NC_01685(	RefSeq	CDS	67052	68392	-	0	ID=cds66;N	0	1	STM14_0067	STM14_0067	putative citrate-sodium symporter	0,142100166	-0,623264422
NC_01685(	RefSeq	CDS	68513	68671	-	0	ID=cds67;N	0	0	STM14_0068	STM14_0068	hypothetical protein	0	NA
NC_01685(	RefSeq	CDS	68637	69680	+	0	ID=cds68;N	0	0	STM14_0069	citC2	putative citrate lyase synthetase	0	NA
NC_01685(	RefSeq	CDS	69710	70003	+	0	ID=cds69;N	0	0	STM14_0070	citD2	citrate lyase subunit gamma	0	NA
NC_01685(	RefSeq	CDS	70000	70869	+	0	ID=cds70;N	0	1,5	STM14_0071	citE2	putative citrate lyase beta chain	0,284200331	-1,182723423
NC_01685(	RefSeq	CDS	70880	72400	+	0	ID=cds71;N	0	5,5	STM14_0072	citF2	putative citrate lyase alpha chain%2Fcitrate-ACP transfera	0,852600993	-1,98056697
NC_01685(	RefSeq	CDS	72400	72951	+	0	ID=cds72;N	0	1	STM14_0073	citX2	putative cytoplasmic protein	0,142100166	-0,623264422
NC_01685(	RefSeq	CDS	72929	73837	+	0	ID=cds73;N	0	0,5	STM14_0074	citG2	triphosphoribosyl-dephospho-CoA synthase	0	NA
NC_01685(	RefSeq	CDS	74017	74838	+	0	ID=cds74;N	3	6	STM14_0075	dapB	dihydrodipicolinate reductase	6,130568103	1,664704357
NC_01685(	RefSeq	CDS	74964	75239	-	0	ID=cds75;N	0	0	STM14_0076	STM14_0076	hypothetical protein	0	NA
NC_01685(	RefSeq	CDS	75881	77029	+	0	ID=cds76;N	0	1	STM14_0077	carA	carbamoyl phosphate synthase small subunit	0,142100166	-0,623264422
NC_01685(	RefSeq	CDS	77048	80275	+	0	ID=cds77;N	0	1,5	STM14_0078	carB	carbamoyl phosphate synthase large subunit	0,284200331	-1,182723423
NC_01685(	RefSeq	CDS	80354	80542	+	0	ID=cds78;N	0	1,1	STM14_0079	STM14_0079	hypothetical protein	0,142100166	-0,623264422
NC_01685(	RefSeq	CDS	80499	80957	+	0	ID=cds79;N	0	2,9	STM14_0080	tnpA_1	transposase	0,426300497	-1,498211557
NC_01685(	RefSeq	CDS	81260	81655	+	0	ID=cds80;N	0	30	STM14_0081	caiF	DNA-binding transcriptional activator CaiF	4,263004966	-2,464060063
NC_01685(	RefSeq	CDS	81732	82328	-	0	ID=cds81;N	0	1	STM14_0082	caiE	carnitine operon protein CaiE	0,142100166	-0,623264422
NC_01685(	RefSeq	CDS	82437	83222	-	0	ID=cds82;N	0	1,5	STM14_0083	caiD	carnitinyl-CoA dehydratase	0,284200331	-1,182723423
NC_01685(	RefSeq	CDS	83276	84829	-	0	ID=cds83;N	0	9	STM14_0084	caiC	putative crotonobetaine%2Fcarnitine-CoA ligase	1,27890149	-2,207741391
NC_01685(	RefSeq	CDS	84892	86109	-	0	ID=cds84;N	0	1	STM14_0085	caiB	crotonobetainyl-CoA:carnitine CoA-transferase	0,142100166	-0,623264422
NC_01685(	RefSeq	CDS	86109	86246	-	0	ID=cds85;N	0	0	STM14_0086	STM14_0086	hypothetical protein	0	NA
NC_01685(	RefSeq	CDS	86221	87363	-	0	ID=cds86;N	0	6,5	STM14_0087	caiA	crotonobetainyl-CoA dehydrogenase	0,852600993	-1,98056697
NC_01685(	RefSeq	CDS	87398	88915	-	0	ID=cds87;N	0	2,5	STM14_0088	caiT	L-carnitine%2Fgamma-butyrobetaine antiporter	0,284200331	-1,182723423
NC_01685(	RefSeq	CDS	89119	89328	+	0	ID=cds88;N	0	0	STM14_0089	STM14_0089	hypothetical protein	0	NA
NC_01685(	RefSeq	CDS	89396	90166	+	0	ID=cds89;N	0	2	STM14_0090	fixA	putative electron transfer flavoprotein FixA	0,284200331	-1,182723423
NC_01685(	RefSeq	CDS	90182	91123	+	0	ID=cds90;N	0	1,5	STM14_0091	fixB	putative electron transfer flavoprotein FixB	0,284200331	-1,182723423
NC_01685(	RefSeq	CDS	91173	92459	+	0	ID=cds91;N	0	1,5	STM14_0092	fixC	putative oxidoreductase FixC	0,284200331	-1,182723423
NC_01685(	RefSeq	CDS	92456	92743	+	0	ID=cds92;N	0	0	STM14_0093	fixX	putative ferredoxin	0	NA
NC_01685(	RefSeq	CDS	92890	94230	+	0	ID=cds93;N	0	5	STM14_0094	yaaU	putative transport protein	0,710500828	-1,864806942
NC_01685(	RefSeq	CDS	94319	94549	+	0	ID=cds94;N	0	0	STM14_0095	STM14_0095	putative outer membrane lipoprotein	0	NA
NC_01685(	RefSeq	CDS	94843	95259	+	0	ID=cds95;N	0	9,5	STM14_0096	STM14_0096	hypothetical protein	1,421001655	-2,256999172
NC_01685(	RefSeq	CDS	95263	95379	-	0	ID=cds96;N	0	0	STM14_0097	STM14_0097	hypothetical protein	0	NA
NC_01685(	RefSeq	CDS	95483	95773	-	0	ID=cds97;N	0	7	STM14_0098	STM14_0098	hypothetical protein	0,994701159	-2,072978628
NC_01685(	RefSeq	CDS	95983	96177	+	0	ID=cds98;N	0	0	STM14_0099	STM14_0099	putative inner membrane protein	0	NA
NC_01685(	RefSeq	CDS	96671	98560	+	0	ID=cds99;N	0	1,5	STM14_0100	STM14_0100	putative sulfatase	0,284200331	-1,182723423
NC_01685(	RefSeq	CDS	98644	98802	-	0	ID=cds100;	0	0	STM14_0101	STM14_0101	hypothetical protein	0	NA
NC_01685(	RefSeq	CDS	98738	99019	+	0	ID=cds101;	0	0	STM14_0102	STM14_0102	hypothetical protein	0	NA
NC_01685(	RefSeq	CDS	98967	99497	+	0	ID=cds102;	0	0	STM14_0103	yabF	glutathione-regulated potassium-efflux system ancillary p	0	NA
NC_01685(	RefSeq	CDS	99490	101352	+	0	ID=cds103;	0	2	STM14_0104	kefC	glutathione-regulated potassium-efflux system protein Ke	0,284200331	-1,182723423
NC_01685(	RefSeq	CDS	101349	101474	-	0	ID=cds104;	0	0	STM14_0105	STM14_0105	hypothetical protein	0	NA
NC_01685(	RefSeq	CDS	101551	102030	+	0	ID=cds105;	0	5,5	STM14_0106	folA	dihydrofolate reductase	0,852600993	-1,98056697
NC_01685(	RefSeq	CDS	102136	102984	-	0	ID=cds106;	0	25	STM14_0107	apaH	diadenosine tetraphosphatase	3,552504138	-2,43600272
NC_01685(	RefSeq	CDS	102995	103372	-	0	ID=cds107;	0	12,5	STM14_0108	apaG	ApaG protein	1,705201986	-2,329925387
NC_01685(	RefSeq	CDS	103375	104196	-	0	ID=cds108;	0	46,5	STM14_0109	ksgA	dimethyladenosine transferase	6,536607615	-2,201017198
NC_01685(	RefSeq	CDS	104193	105182	-	0	ID=cds109;	0	41	STM14_0110	pdxA_1	4-hydroxythreonine-4-phosphate dehydrogenase	5,826106787	-2,241137745
NC_01685(	RefSeq	CDS	105182	106468	-	0	ID=cds110;	6	93,5	STM14_0111	surA	peptidyl-prolyl cis-trans isomerase SurA	23,91334978	-0,237638
NC_01685(	RefSeq	CDS	106522	108882	-	0	ID=cds111;	0	148	STM14_0112	imp	organic solvent tolerance protein	21,0308245	-1,874746658
NC_01685(	RefSeq	CDS	108892	109053	-	0	ID=cds112;	0	9	STM14_0113	STM14_0113	hypothetical protein	1,27890149	-2,207741391
NC_01685(	RefSeq	CDS	109136	109948	+	0	ID=cds113;	0	11	STM14_0114	djlA	DNA-J like membrane chaperone protein	1,563101821	-2,297202414
NC_01685(	RefSeq	CDS	110043	110702	-	0	ID=cds114;	0	4	STM14_0115	rluA	23S rRNA%2FtRNA pseudouridine synthase A	0,568400662	-1,710438693
NC_01685(	RefSeq	CDS	110714	113620	-	0	ID=cds115;	0	18	STM14_0116	hepA	ATP-dependent helicase HepA	2,55780298	-2,419166771
NC_01685(	RefSeq	CDS	113794	116145	-	0	ID=cds116;	0	18,5	STM14_0117	polB	DNA polymerase II	2,55780298	-2,419166771
NC_01685(	RefSeq	CDS	116189	116809	-	0	ID=cds117;	0	2,5	STM14_0118	STM14_0118	hypothetical protein	0,284200331	-1,182723423
NC_01685(	RefSeq	CDS	117528	117947	+	0	ID=cds118;	0	1	STM14_0119	STM14_0119	putative cytoplasmic protein	0,142100166	-0,623264422
NC_01685(	RefSeq	CDS	117953	118648	-	0	ID=cds119;	0	0	STM14_0120	araD	L-ribulose-5-phosphate 4-epimerase	0	NA
NC_01685(	RefSeq	CDS	118789	120291	-	0	ID=cds120;	0	1	STM14_0121	araA	L-arabinose isomerase	0,142100166	-0,623264422
NC_01685(	RefSeq	CDS	120302	122011	-	0	ID=cds121;	0	1	STM14_0122	araB	ribulokinase	0,142100166	-0,623264422
NC_01685(	RefSeq	CDS	122352	123197	+	0	ID=cds122;	0	9	STM14_0123	araC	DNA-binding transcriptional regulator AraC	1,27890149	-2,207741391
NC_01685(	RefSeq	CDS	123316	124083	+	0	ID=cds123;	0	11,33333333	STM14_0124	yabI	hypothetical protein	1,563101821	-2,297202414
NC_01685(	RefSeq	CDS	124122	124829	-	0	ID=cds124;	0	1	STM14_0125	thiQ	thiamine transporter ATP-binding subunit	0,142100166	-0,623264422
NC_01685(	RefSeq	CDS	124813	126423	-	0	ID=cds125;	0	3	STM14_0126	thiP	thiamine transporter membrane protein	0,426300497	-1,498211557
NC_01685(	RefSeq	CDS	126399	127382	-	0	ID=cds126;	0	3	STM14_0127	tbpA	thiamine transporter substrate binding subunit	0,426300497	-1,498211557
NC_01685(	RefSeq	CDS	127427	127546	-	0	ID=cds127;	0	2	STM14_0128	STM14_0128	putative lipoprotein	0,284200331	-1,182723423
NC_01685(	RefSeq	CDS	127560	129218	-	0	ID=cds128;	0	2,5	STM14_0129	yabN	transcriptional regulator SgrR	0,284200331	-1,182723423
NC_01685(	RefSeq	CDS	129166	129492	-	0	ID=cds129;	0	0	STM14_0130	STM14_0130	hypothetical protein	0	NA
NC_01685(	RefSeq	CDS	129769	130374	-	0	ID=cds130;	0	0	STM14_0131	leuD	isopropylmalate isomerase small subunit	0	NA
NC_01685(	RefSeq	CDS	130385	131785	-	0	ID=cds131;	0	3	STM14_0132	leuC	isopropylmalate isomerase large subunit	0,426300497	-1,498211557
NC_01685(	RefSeq	CDS	131788	132879	-	0	ID=cds132;	0	2	STM14_0133	leuB	3-isopropylmalate dehydrogenase	0,284200331	-1,182723423
NC_01685(	RefSeq	CDS	132879	134450	-	0	ID=cds133;	0	9	STM14_0134	leuA	2-isopropylmalate synthase	1,27890149	-2,207741391
NC_01685(	RefSeq	CDS	134538	134624	-	0	ID=cds134;	2	17	STM14_0135	leuL	leu operon leader peptide	5,934347554	0,350156269
NC_01685(	RefSeq	CDS	134689	134793	-	0	ID=cds135;	0	0	STM14_0136	STM14_0136	hypothetical protein	0	NA
NC_01685(	RefSeq	CDS	135067	135210	+	0	ID=cds136;	0	0	STM14_0137	STM14_0137	hypothetical protein	0	NA
NC_01685(	RefSeq	CDS	135281	136225	+	0	ID=cds137;	0	0,5	STM14_0138	leuO	leucine transcriptional activator	0	NA
NC_01685(	RefSeq	CDS	136610	138271	+	0	ID=cds138;	0	7	STM14_0139	ilvI	acetolactate synthase 3 catalytic subunit	0,994701159	-2,072978628
NC_01685(	RefSeq	CDS	138274	138765	+	0	ID=cds139;	0	1	STM14_0140	ilvH	acetolactate synthase 3 regulatory subunit	0,142100166	-0,623264422
NC_01685(	RefSeq	CDS	138755	138877	-	0	ID=cds140;	0	1	STM14_0141	STM14_0141	hypothetical protein	0,142100166	-0,623264422
NC_01685(	RefSeq	CDS	139049	140053	+	0	ID=cds141;	0	48,5	STM14_0142	fruR	DNA-binding transcriptional regulator FruR	6,820807946	-2,185279286
NC_01685(	RefSeq	CDS	140204	140338	-	0	ID=cds142;	0	0	STM14_0143	STM14_0143	hypothetical protein	0	NA
NC_01685(	RefSeq	CDS	140496	140633	+	0	ID=cds143;	2,5	4,5	STM14_0144	STM14_0144	hypothetical protein	4,087045402	1,631390028
NC_01685(	RefSeq	CDS	140659	141117	+	0	ID=cds144;	4,5	55	STM14_0145	yabB	cell division protein MraZ	14,85279858	-0,104932734

NC_01685(	RefSeq	CDS	141119	142060	+	0	ID=cds145;	4	37,5	STM14_0146	mraW	S-adenosyl-methyltransferase MraW	12,43709577	0,262029712
NC_01685(	RefSeq	CDS	142057	142422	+	0	ID=cds146;	0	29	STM14_0147	ftsL	cell division protein FtsL	4,120904801	-2,455089955
NC_01685(	RefSeq	CDS	142438	144204	+	0	ID=cds147;	8	125,5	STM14_0148	ftsI	division specific transpeptidase	31,97919982	-0,243225152
NC_01685(	RefSeq	CDS	144191	145678	+	0	ID=cds148;	0	62	STM14_0149	murE	UDP-N-acetylmuramoylalanyl-D-glutamate--2%2C 6-diami	8,810210263	-2,091539774
NC_01685(	RefSeq	CDS	145675	147033	+	0	ID=cds149;	0	36,5	STM14_0150	murF	UDP-N-acetylmuramoyl-tripeptide--D-alanyl-D- alanine lig	5,115605959	-2,281753063
NC_01685(	RefSeq	CDS	147027	148109	+	0	ID=cds150;	0	93,5	STM14_0151	mraY	phospho-N-acetylmuramoyl-pentapeptide- transferase	13,35741556	-1,928482877
NC_01685(	RefSeq	CDS	148112	149428	+	0	ID=cds151;	4	73	STM14_0152	murD	UDP-N-acetylmuramoyl-L-alanyl-D-glutamate synthetase	17,41060156	-0,389580279
NC_01685(	RefSeq	CDS	149428	150672	+	0	ID=cds152;	0	48	STM14_0153	ftsW	cell division protein FtsW	6,820807946	-2,185729286
NC_01685(	RefSeq	CDS	150669	151736	+	0	ID=cds153;	2	34,5	STM14_0154	murG	N-acetylglucosaminyl transferase	8,350050368	-0,299869993
NC_01685(	RefSeq	CDS	151855	153330	+	0	ID=cds154;	0	57,5	STM14_0155	murC	UDP-N-acetylmuramate--L-alanine ligase	8,241809601	-2,117369507
NC_01685(	RefSeq	CDS	153323	154243	+	0	ID=cds155;	0	81,5	STM14_0156	ddl_2	D-alanine--D-alanine ligase	11,65221357	-1,983056535
NC_01685(	RefSeq	CDS	154245	155075	+	0	ID=cds156;	0	46,91666667	STM14_0157	ftsQ	cell division protein FtsQ	6,67870778	-2,193319559
NC_01685(	RefSeq	CDS	155072	156334	+	0	ID=cds157;	2	109,25	STM14_0158	ftsA	cell division protein FtsA	19,00756278	-1,447716714
NC_01685(	RefSeq	CDS	156395	157546	+	0	ID=cds158;	0	188,5	STM14_0159	ftsZ	cell division protein FtsZ	26,71483112	-1,8957776
NC_01685(	RefSeq	CDS	157647	158564	+	0	ID=cds159;	2	300	STM14_0160	lpxC	UDP-3-O-%5B3-hydroxymyristoyl%5D N-acetylglucosamin	46,1486944	-2,387587183
NC_01685(	RefSeq	CDS	158843	159340	+	0	ID=cds160;	1	33	STM14_0161	yacA	SecA regulator SecM	6,448627833	-0,888236347
NC_01685(	RefSeq	CDS	159402	162107	+	0	ID=cds161;	1	148	STM14_0162	secA	preprotein translocase subunit SecA	22,79014687	-2,305586179
NC_01685(	RefSeq	CDS	162259	162654	+	0	ID=cds162;	0	1	STM14_0163	mutT	nucleoside triphosphate pyrophosphohydrolase	0,142100166	-0,623264422
NC_01685(	RefSeq	CDS	162875	162979	+	0	ID=cds163;	0	0,5	STM14_0165	STM14_0165	LysR family transcriptional regulator	0	NA
NC_01685(	RefSeq	CDS	162976	163167	-	0	ID=cds164;	0	6	STM14_0166	yacG	zinc-binding protein	0,852600993	-1,98056697
NC_01685(	RefSeq	CDS	163177	163920	-	0	ID=cds165;	0	14,5	STM14_0167	yacF	hypothetical protein	1,989402318	-2,376535557
NC_01685(	RefSeq	CDS	163920	164540	-	0	ID=cds166;	0	4,5	STM14_0168	coaE	dephospho-CoA kinase	0,568400662	-1,710438693
NC_01685(	RefSeq	CDS	164766	165809	+	0	ID=cds167;	0	38	STM14_0169	guaC	guanosine 5%27-monophosphate oxidoreductase	5,39980629	-2,264331336
NC_01685(	RefSeq	CDS	165840	167042	-	0	ID=cds168;	0	0	STM14_0170	hofC	type IV pilin biogenesis protein	0	NA
NC_01685(	RefSeq	CDS	167032	168417	-	0	ID=cds169;	0	0	STM14_0171	hofB	hypothetical protein	0	NA
NC_01685(	RefSeq	CDS	168427	168864	-	0	ID=cds170;	0	1	STM14_0172	ppdD	putative major pilin subunit	0,142100166	-0,623264422
NC_01685(	RefSeq	CDS	168890	169072	-	0	ID=cds171;	0	0,5	STM14_0173	STM14_0173	hypothetical protein	0	NA
NC_01685(	RefSeq	CDS	169086	169979	-	0	ID=cds172;	0	6,5	STM14_0174	nadC	quinolinate phosphoribosyltransferase	0,852600993	-1,98056697
NC_01685(	RefSeq	CDS	170067	170630	+	0	ID=cds173;	0	13,5	STM14_0175	ampD	N-acetyl-anhydromuranmyl-L-alanine amidase	1,989402318	-2,376535557
NC_01685(	RefSeq	CDS	170627	171481	+	0	ID=cds174;	0	12	STM14_0176	ampE	regulatory protein AmpE	1,705201986	-2,329925387
NC_01685(	RefSeq	CDS	171573	172523	-	0	ID=cds175;	0	1	STM14_0177	STM14_0177	putative cytoplasmic protein	0,142100166	-0,623264422
NC_01685(	RefSeq	CDS	172523	173929	-	0	ID=cds176;	0	1	STM14_0178	STM14_0178	Na%2B%2Fgalactoside symporter	0,142100166	-0,623264422
NC_01685(	RefSeq	CDS	173962	174090	-	0	ID=cds177;	0	0	STM14_0179	STM14_0179	hypothetical protein	0	NA
NC_01685(	RefSeq	CDS	174093	175466	-	0	ID=cds178;	0	4	STM14_0180	aroP	aromatic amino acid transporter	0,568400662	-1,710438693
NC_01685(	RefSeq	CDS	175672	175794	+	0	ID=cds179;	0	0	STM14_0181	STM14_0181	hypothetical protein	0	NA
NC_01685(	RefSeq	CDS	176029	176793	+	0	ID=cds180;	0	9	STM14_0182	pdhR	transcriptional regulator PdhR	1,27890149	-2,207741391
NC_01685(	RefSeq	CDS	176953	179616	+	0	ID=cds181;	1	145,5	STM14_0183	aceE	pyruvate dehydrogenase subunit E1	22,50594654	-2,294118721
NC_01685(	RefSeq	CDS	179631	181520	+	0	ID=cds182;	0	108	STM14_0184	aceF	dihydrolipoamide acetyltransferase	15,34681788	-1,876042944
NC_01685(	RefSeq	CDS	181720	183144	+	0	ID=cds183;	5	190,5	STM14_0185	lpdA	dihydrolipoamide dehydrogenase	35,7956433	-1,119684401
NC_01685(	RefSeq	CDS	183222	183350	+	0	ID=cds184;	0	1	STM14_0186	STM14_0186	hypothetical protein	0,142100166	-0,623264422
NC_01685(	RefSeq	CDS	183433	183717	+	0	ID=cds185;	0	0	STM14_0187	STM14_0187	putative outer membrane protein	0	NA
NC_01685(	RefSeq	CDS	183755	184546	-	0	ID=cds186;	0	1	STM14_0188	STM14_0188	hypothetical protein	0,142100166	-0,623264422
NC_01685(	RefSeq	CDS	186562	189159	+	0	ID=cds187;	0	171	STM14_0190	acnB	bifunctional aconitate hydratase 2%2F2-methylisocitrate c	24,29912831	-1,887897921
NC_01685(	RefSeq	CDS	189220	190062	-	0	ID=cds188;	0	7,5	STM14_0191	STM14_0191	putative restriction endonuclease	1,136801324	-2,147403465
NC_01685(	RefSeq	CDS	190308	190670	+	0	ID=cds189;	2	20,5	STM14_0192	yacL	hypothetical protein	6,360648051	0,199657938
NC_01685(	RefSeq	CDS	190905	191858	+	0	ID=cds190;	0	9,666666667	STM14_0193	kdgT	2-keto-3-deoxygluconate permease	1,421001655	-2,256999172
NC_01685(	RefSeq	CDS	191855	193126	+	0	ID=cds191;	0	8	STM14_0194	STM14_0194	putative inner membrane protein	1,136801324	-2,147403465
NC_01685(	RefSeq	CDS	193116	194099	+	0	ID=cds192;	0	7,75	STM14_0195	pdxA_2	4-hydroxythreonine-4-phosphate dehydrogenase	1,136801324	-2,147403465
NC_01685(	RefSeq	CDS	194109	194876	+	0	ID=cds193;	0	13,75	STM14_0196	STM14_0196	putative transcriptional regulator	1,989402318	-2,376535557
NC_01685(	RefSeq	CDS	194906	195700	-	0	ID=cds194;	0	4	STM14_0197	speD	S-adenosylmethionine decarboxylase	0,568400662	-1,710438693
NC_01685(	RefSeq	CDS	195721	196581	-	0	ID=cds195;	0	12	STM14_0198	speE	spermidine synthase	1,705201986	-2,329925387
NC_01685(	RefSeq	CDS	196688	197035	-	0	ID=cds196;	0	8,5	STM14_0199	yacC	hypothetical protein	1,136801324	-2,147403465
NC_01685(	RefSeq	CDS	197237	198847	+	0	ID=cds197;	0	11,5	STM14_0200	cueO	multicopper oxidase	1,705201986	-2,329925387
NC_01685(	RefSeq	CDS	198925	201315	-	0	ID=cds198;	0	1	STM14_0201	gcd	glucose dehydrogenase	0,142100166	-0,623264422
NC_01685(	RefSeq	CDS	201521	202057	+	0	ID=cds199;	0	30,83333333	STM14_0202	hpt	hypoxanthine-guanine phosphoribosyltransferase	4,405105132	-2,475774913
NC_01685(	RefSeq	CDS	202115	202777	-	0	ID=cds200;	0	12	STM14_0203	yadF	carbonic anhydrase	1,705201986	-2,329925387
NC_01685(	RefSeq	CDS	202886	203812	+	0	ID=cds201;	0	9,5	STM14_0204	yadG	putative ABC-type multidrug transport system ATPase cor	1,421001655	-2,256999172
NC_01685(	RefSeq	CDS	203809	204579	+	0	ID=cds202;	0	9	STM14_0205	yadH	putative transport protein	1,27890149	-2,207741391
NC_01685(	RefSeq	CDS	204696	205775	-	0	ID=cds203;	0	1	STM14_0206	stiH	putative fimbrial protein precurosr	0,142100166	-0,623264422
NC_01685(	RefSeq	CDS	205784	208330	-	0	ID=cds204;	0	2	STM14_0207	stiC	fimbrial usher protein	0,284200331	-1,182723423
NC_01685(	RefSeq	CDS	208357	209040	-	0	ID=cds205;	0	0	STM14_0208	stiB	putative fimbrial chaperone	0	NA
NC_01685(	RefSeq	CDS	209088	209627	-	0	ID=cds206;	0	0	STM14_0209	stiA	putative fimbrial subunit	0	NA
NC_01685(	RefSeq	CDS	209796	209966	-	0	ID=cds207;	0	0	STM14_0210	STM14_0210	hypothetical protein	0	NA
NC_01685(	RefSeq	CDS	209997	210437	+	0	ID=cds208;	0	5	STM14_0211	yadI	putative PTS enzyme	0,710500828	-1,864806942
NC_01685(	RefSeq	CDS	210499	211728	+	0	ID=cds209;	0	12	STM14_0212	yadE	putative xylanase%2Fchitin deacetylase	1,705201986	-2,329925387
NC_01685(	RefSeq	CDS	211735	212115	-	0	ID=cds210;	0	7	STM14_0213	panD	aspartate alpha-decarboxylase	0,994701159	-2,072978628
NC_01685(	RefSeq	CDS	212210	213064	-	0	ID=cds211;	0	12	STM14_0214	panC	pantoate--beta-alanine ligase	1,705201986	-2,329925387
NC_01685(	RefSeq	CDS	213058	213171	-	0	ID=cds212;	0	0,5	STM14_0215	STM14_0215	hypothetical protein	0	NA
NC_01685(	RefSeq	CDS	213190	213981	-	0	ID=cds213;	0	5	STM14_0216	panB	3-methyl-2-oxobutanoate hydroxymethyltransferase	0,710500828	-1,864806942
NC_01685(	RefSeq	CDS	214102	214581	-	0	ID=cds214;	0	4	STM14_0217	folK	2-amino-4-hydroxy-6-hydroxymethyldihydropteridine pyro	0,568400662	-1,710438693
NC_01685(	RefSeq	CDS	214578	215996	-	0	ID=cds215;	0	24	STM14_0218	pcnB	poly%28A%29 polymerase I	3,410403973	-2,434086198
NC_01685(	RefSeq	CDS	216137	217033	-	0	ID=cds216;	0	10	STM14_0219	yadB	glutamyl-Q tRNA%28Asp%29 synthetase	1,421001655	-2,256999172
NC_01685(	RefSeq	CDS	217102	217557	-	0	ID=cds217;	2	46,83333333	STM14_0220	dksA	DnaK transcriptional regulator DksA	10,19735252	-0,614201102
NC_01685(	RefSeq	CDS	217734	218438	-	0	ID=cds218;	0	9	STM14_0221	sfsA	sugar fermentation stimulation protein A	1,27890149	-2,207741391
NC_01685(	RefSeq	CDS	218455	218985	-	0	ID=cds219;	0	3	STM14_0222	ligT	2%27-5%27 RNA ligase	0,426300497	-1,498211557
NC_01685(	RefSeq	CDS	219013	221487	+	0	ID=cds220;	0	15,33333333	STM14_0223	hrpB	ATP-dependent RNA helicase HrpB	2,131502483	-2,392032191
NC_01685(	RefSeq	CDS	221426	221587	+	0	ID=cds221;	0	0,333333333	STM14_0224	STM14_0224	hypothetical protein	0	NA
NC_01685(	RefSeq	CDS	221522	221629	-	0	ID=cds222;	0	0	STM14_0225	STM14_0225	hypothetical protein	0	NA
NC_01685(	RefSeq	CDS	221628	224150	+	0	ID=cds223;	0	41,5	STM14_0226	mrcB	penicillin-binding protein 1b	5,968206953	-2,232890319
NC_01685(	RefSeq	CDS	224289	224429	-	0	ID=cds224;	0	0	STM14_0227	STM14_0227	hypothetical protein	0	NA
NC_01685(	RefSeq	CDS	224443	226632	+	0	ID=cds225;	0	6	STM14_0228	fhuA	ferrichrome outer membrane transporter	0,852600993	-1,98056697
NC_01685(	RefSeq	CDS	226681	227478	+	0	ID=cds226;	2,5	0	STM14_0229	fhuC	iron-hydroxamate transporter ATP-binding subunit	3,51864474	1,925445802
NC_01685(	RefSeq	CDS	227478	228368	+	0	ID=cds227;	0	2	STM14_0230	fhuD	iron-hydroxamate transporter substrate-binding subunit	0,284200331	-1,182723423
NC_01685(	RefSeq	CDS	228365	230422	+	0	ID=cds228;	0	1	STM14_0231	fhuB	iron-hydroxamate transporter permease subunit	0,142100166	-0,623264422
NC_01685(	RefSeq	CDS	230459	230665	+	0	ID=cds229;	0	0,5	STM14_0232	STM14_0232	hypothetical protein	0	NA
NC_01685(	RefSeq	CDS	231001	231144	+	0	ID=cds230;	0	0	STM14_0233	STM14_0233	hypothetical protein	0	NA
NC_01685(	RefSeq	CDS	231361	231921	+	0	ID=cds231;	0	0	STM14_0234	stfA	putative fimbrial subunit	0	NA
NC_01685(	RefSeq	CDS	232007	234664	+	0	ID=cds232;	0	1,5	STM14_0235	stfC	putative fimbrial outer membrane usher	0,284200331	-1,182723423
NC_01685(	RefSeq	CDS	234682	235434	+	0	ID=cds233;	0	0	STM14_0236	stfD	putative periplasmic fimbrial chaperone	0	NA
NC_01685(	RefSeq	CDS	235453	235965	+	0	ID=cds234;	0	0,5	STM14_0237	stfE	putative minor fimbrial subunit	0	NA
NC_01685(	RefSeq	CDS	235962	236438	+	0	ID=cds235;	0	0	STM14_0238	stfF	putative minor fimbrial subunit	0	NA
NC_01685(	RefSeq	CDS	236438	236968	+	0	ID=cds236;	0	0	STM14_0239	stfG	putative minor fimbrial subunit	0	NA
NC_01685(	RefSeq	CDS	236968	237807	+	0	ID=cds237;	0	8,333333333	STM14_0240	STM14_0240	putative outer membrane protein	1,136801324	-2,147403465
NC_01685(	RefSeq	CDS	237915	239195	-	0	ID=cds238;	0	23,5	STM14_0241	hemL	glutamate-1-semialdehyde aminotransferase	3,410403973	-2,434086198
NC_01685(	RefSeq	CDS	239222	239365	+	0	ID=cds239;	0	0,5	STM14_0242	STM14_0242	hypothetical protein	0	NA
NC_01685(	RefSeq	CDS	239365	240786	+	0	ID=cds240;	0	14,33333333	STM14_0243	yadQ	chloride channel protein	1,989402318	-2,376535557
NC_01685(	RefSeq	CDS	240868	241212	+	0	ID=cds241;	0	18,5	STM14_0244	yadR	iron-sulfur cluster insertion protein ErpA	2,55780298	-2,419166771
NC_01685(	RefSeq	CDS	241313	241936	-	0	ID=cds242;	0	4,5	STM14_0245	yadS	hypothetical protein	0,568400662	-1,710438693
NC_01685(	RefSeq	CDS	241973	242773	-	0	ID=cds243;	0	10,5	STM14_0246	btuF	vitamin B12-transporter protein BtuF	1,421001655	-2,256999172
NC_01685(	RefSeq	CDS	242766	243464	-	0	ID=cds244;	0	16	STM14_0247	pfs	5%27-methylthioadenosine%2FS-adenosylhomocysteine r	2,273602649	-2,403729808
NC_01685(	RefSeq	CDS	243549	245066	+	0	ID=cds245;	0	16,5	STM14_0248	dgt	deoxyguanosinetriphosphate triphosphohydrolase	2,273602649	-2,403729808
NC_01685(	RefSeq	CDS	245196	246623	+	0	ID=cds246;	0	91,5	STM14_0249	htrA	serine endoprotease	13,07321523	-1,936690345
NC_01685(	RefSeq	CDS	246776	247933	+	0	ID=cds247;	1,5	20,5	STM14_0250	cdaR	carbohydrate diacid transcriptional activator CdaR	6,360648051	0,199657938
NC_01685(	RefSeq	CDS	248022	248408	-	0	ID=cds248;	0	71,5	STM14_0251	yaeH	hypothetical protein	10,23121192	-2,035392961
NC_01685(	RefSeq	CDS	249994	250818	-	0	ID=cds249;	0	26	STM14_0253	dapD	2%2C3%2C4%2C5-tetrahydropyridine-2%2C6-carboxylate	3,694604304	-2,438835558
NC_01685(	RefSeq	CDS	250848	253520	-	0	ID=cds250;	0	23,5	STM14_0254	glnD	PII uridylyl-transferase	3,410403973	-2,434086198
NC_01685(	RefSeq	CDS	253757	254551	-	0	ID=cds251;	0	46,5	STM14_0255	map	methionine aminopeptidase	6,536607615	-2,201017198
NC_01685(	RefSeq	CDS	254799	255083	+	0	ID=cds252;	2	32,75	STM14_0256	STM14_0256	30S ribosomal protein S2	8,207950203	-0,271358932
NC_01685(	RefSeq	CDS	255002	255727	+	0	ID=cds253;	3	227,25	STM14_0257	rpsB	30S ribosomal protein S2	37,53470469	-1,770746869
NC_01685(	RefSeq	CDS	255985	256836	+	0	ID=cds254;	0	177,5	STM14_0258	tsf	elongation factor Ts	25,29382947	-1,891303465
NC_01685(	RefSeq	CDS	256981	257706	+	0	ID=cds255;	0	52,5	STM14_0259	pyrH	uridylate kinase	7,389208608	-2,158807911
NC_01685(	RefSeq	CDS	257853	258410	+	0	ID=cds256;	0	62,16666667	STM14_0260	frr	ribosome recycling factor	8,810210263	-2,091539774
NC_01685(	RefSeq	CDS	258551	259747	+	0	ID=cds257;	0	27,5	STM14_0261	dxr	1-deoxy-D-xylulose 5-phosphate reductoisomerase	3,978804635	-2,448045729
NC_01685(	RefSeq	CDS	259830	260033	+	0	ID=cds258;	0	6,5	STM14_0262	STM14_0262	hypothetical protein	0,852600993	-1,98056697
NC_01685(	RefSeq	CDS	260060	260818	+	0	ID=cds259;	7	47,5	STM14_0263	uppS	undecaprenyl pyrophosphate synthase	19,13606454	0,596417255
NC_01685(	RefSeq	CDS	260831	261688	+	0	ID=cds260;	0	35,5	STM14_0264	cdsA	CDP-diglyceride synthase	5,115605959	-2,281753063
NC_01685(	RefSeq	CDS	261700	263052	+	0	ID=cds261;	0	116,5	STM14_0265	yaeL	zinc metallopeptidase	16,4836192	-1,849193185
NC_01685(	RefSeq	CDS	263084	265498	+	0	ID=cds262;	0	245	STM14_0266	yaeT	outer membrane protein assembly factor YaeT	34,81454056	-1,914927646
NC_01685(	RefSeq	CDS	265621	266106	+	0	ID=cds263;	1	104,5	STM14_0267	hlpA	periplasmic chaperone	16,53773959	-1,994540578
NC_01685(	RefSeq	CDS	266110	267135	+	0	ID=cds264;	1	124	STM14_0268	lpxD	UDP-3-O-%5B3-hydroxymyristoyl%5D glucosamine N-acyl	19,3797429	-2,153522293
NC_01685(	RefSeq	CDS	267151	267696	+	0	ID=cds265;	0	61,5	STM14_0269	fabZ	%283R%29-hydroxymyristoyl-ACP dehydratase	8,810210263	-2,091539774
NC_01685(	RefSeq	CDS	267700	268488	+	0	ID=cds266;	0	49,66666667	STM14_0270	lpxA	UDP-N-acetylglucosamine acyltransferase	7,105008277	-2,173389122
NC_01685(	RefSeq	CDS	268488	269636	+	0	ID=cds267;	0	17,5	STM14_0271	lpxB	lipid-A-disaccharide synthase	2,55780298	-2,419166771
NC_01685(	RefSeq	CDS	269633	270229	+	0	ID=cds268;	0	19	STM14_0272	rnhB	ribonuclease HII	2,699903145	-2,42419778

NC_01685(	RefSeq	CDS	270253	273735	+	0	ID=cds269;	1	110,5	STM14_0273	dnaE	DNA polymerase III subunit alpha	17,39034058	-2,046189894
NC_01685(	RefSeq	CDS	273748	274707	+	0	ID=cds270;	0	38,5	STM14_0274	accA	acetyl-CoA carboxylase carboxyltransferase subunit alpha	5,39980629	-2,264331336
NC_01685(	RefSeq	CDS	274887	276650	+	0	ID=cds271;	0	5,5	STM14_0275	STM14_0275	putative endochitinase	0,852600993	-1,98056697
NC_01685(	RefSeq	CDS	276726	278867	+	0	ID=cds272;	0	12	STM14_0276	ldcC	lysine decarboxylase 2	1,705201986	-2,329925387
NC_01685(	RefSeq	CDS	278923	279312	+	0	ID=cds273;	0	2,5	STM14_0277	yaeR	hypothetical protein	0,284200331	-1,182723423
NC_01685(	RefSeq	CDS	279375	280667	+	0	ID=cds274;	0	11	STM14_0278	tilS	tRNA%28Ile%29-lysidine synthetase	1,563101821	-2,297202414
NC_01685(	RefSeq	CDS	280751	281011	-	0	ID=cds275;	0	2	STM14_0279	rof	Rho-binding antiterminator	0,284200331	-1,182723423
NC_01685(	RefSeq	CDS	280998	281216	-	0	ID=cds276;	0	6	STM14_0280	yaeP	hypothetical protein	0,852600993	-1,98056697
NC_01685(	RefSeq	CDS	281396	281941	+	0	ID=cds277;	0	8,5	STM14_0281	yaeQ	putative cytoplasmic protein	1,136801324	-2,147403465
NC_01685(	RefSeq	CDS	281938	282360	+	0	ID=cds278;	0	2	STM14_0282	yaeJ	hypothetical protein	0,284200331	-1,182723423
NC_01685(	RefSeq	CDS	283167	284885	-	0	ID=cds279;	0	49,5	STM14_0284	proS	prolyl-tRNA synthetase	7,105008277	-2,173389122
NC_01685(	RefSeq	CDS	284996	285703	-	0	ID=cds280;	0	10,5	STM14_0285	yaeB	putative regulatory protein	1,421001655	-2,256999172
NC_01685(	RefSeq	CDS	285700	286104	-	0	ID=cds281;	0	12,5	STM14_0286	rcsF	outer membrane lipoprotein	1,705201986	-2,329925387
NC_01685(	RefSeq	CDS	286223	287038	-	0	ID=cds282;	0	28	STM14_0287	metQ	DL-methionine transporter substrate-binding subunit	3,978804635	-2,448045729
NC_01685(	RefSeq	CDS	287077	287730	-	0	ID=cds283;	0	3	STM14_0288	yaeE	DL-methionine transporter permease	0,426300497	-1,498211557
NC_01685(	RefSeq	CDS	287723	288754	-	0	ID=cds284;	0	1	STM14_0289	metN	DL-methionine transporter ATP-binding subunit	0,142100166	-0,623264422
NC_01685(	RefSeq	CDS	288944	289510	+	0	ID=cds285;	0	11	STM14_0290	yaeD	D%2CD-heptose 1%2C7-bisphosphate phosphatase	1,563101821	-2,297202414
NC_01685(	RefSeq	CDS	289581	289703	-	0	ID=cds286;	0	1	STM14_0291	STM14_0291	hypothetical protein	0,142100166	-0,623264422
NC_01685(	RefSeq	CDS	289616	289819	-	0	ID=cds287;	0	0	STM14_0292	STM14_0292	hypothetical protein	0	NA
NC_01685(	RefSeq	CDS	291760	291876	+	0	ID=cds288;	5,873015873	170,184127	STM14_0296	STM14_0296	hypothetical protein	34,71296236	-0,831201942
NC_01685(	RefSeq	CDS	295769	296572	+	0	ID=cds289;	0	2	STM14_0300	dkgB	2%2C5-diketo-D-gluconate reductase B	0,284200331	-1,182723423
NC_01685(	RefSeq	CDS	296593	297507	-	0	ID=cds290;	0	1	STM14_0301	yafC	putative transcriptional regulator	0,142100166	-0,623264422
NC_01685(	RefSeq	CDS	297612	298787	+	0	ID=cds291;	0	2	STM14_0302	STM14_0302	putative drug efflux protein	0,284200331	-1,182723423
NC_01685(	RefSeq	CDS	298940	299719	+	0	ID=cds292;	0	12,5	STM14_0303	yafD	hypothetical protein	1,705201986	-2,329925387
NC_01685(	RefSeq	CDS	299797	300567	+	0	ID=cds293;	0	7,5	STM14_0304	yafE	putative methyltransferase	1,136801324	-2,147403465
NC_01685(	RefSeq	CDS	300623	301990	-	0	ID=cds294;	11	174	STM14_0305	dniR	membrane-bound lytic murein transglycosylase D	44,07797487	-0,249814204
NC_01685(	RefSeq	CDS	302062	302817	-	0	ID=cds295;	0	11	STM14_0306	gloB	hydroxyacylglutathione hydrolase	1,563101821	-2,297202414
NC_01685(	RefSeq	CDS	302852	303574	+	0	ID=cds296;	0	5,5	STM14_0307	yafS	putative SAM-dependent methyltransferase	0,852600993	-1,98056697
NC_01685(	RefSeq	CDS	303571	304038	-	0	ID=cds297;	0	10	STM14_0308	rnhA	ribonuclease H	1,421001655	-2,256999172
NC_01685(	RefSeq	CDS	304102	304833	+	0	ID=cds298;	0	7	STM14_0309	dnaQ	DNA polymerase III subunit epsilon	0,994701159	-2,072978628
NC_01685(	RefSeq	CDS	304859	304966	-	0	ID=cds299;	0	0	STM14_0310	STM14_0310	hypothetical protein	0	NA
NC_01685(	RefSeq	CDS	305167	305274	+	0	ID=cds300;	0	1	STM14_0312	STM14_0312	hypothetical protein	0,142100166	-0,623264422
NC_01685(	RefSeq	CDS	305365	306420	-	0	ID=cds301;	0	2	STM14_0313	STM14_0313	putative cytoplasmic protein	0,284200331	-1,182723423
NC_01685(	RefSeq	CDS	306431	307426	-	0	ID=cds302;	0	0,5	STM14_0314	STM14_0314	putative cytoplasmic protein	0	NA
NC_01685(	RefSeq	CDS	307423	309306	-	0	ID=cds303;	0	1,5	STM14_0315	STM14_0315	putative cytoplasmic protein	0,284200331	-1,182723423
NC_01685(	RefSeq	CDS	309322	309816	-	0	ID=cds304;	0	0	STM14_0316	STM14_0316	putative cytoplasmic protein	0	NA
NC_01685(	RefSeq	CDS	309813	310637	-	0	ID=cds305;	0	1	STM14_0317	STM14_0317	putative cytoplasmic protein	0,142100166	-0,623264422
NC_01685(	RefSeq	CDS	310624	311526	-	0	ID=cds306;	0	1,5	STM14_0318	STM14_0318	putative cytoplasmic protein	0,284200331	-1,182723423
NC_01685(	RefSeq	CDS	311894	314533	+	0	ID=cds307;	0	35,66666667	STM14_0319	STM14_0319	putative chaperone ATPase	5,115605959	-2,281753063
NC_01685(	RefSeq	CDS	314633	315175	+	0	ID=cds308;	0	2	STM14_0320	STM14_0320	putative cytoplasmic protein	0,284200331	-1,182723423
NC_01685(	RefSeq	CDS	315199	316692	+	0	ID=cds309;	0	1	STM14_0321	STM14_0321	putative cytoplasmic protein	0,142100166	-0,623264422
NC_01685(	RefSeq	CDS	316755	316865	+	0	ID=cds310;	0	0	STM14_0322	STM14_0322	invasol SirA	0	NA
NC_01685(	RefSeq	CDS	316878	317324	+	0	ID=cds311;	0	0	STM14_0323	STM14_0323	putative cytoplasmic protein	0	NA
NC_01685(	RefSeq	CDS	317578	318063	+	0	ID=cds312;	0	0,5	STM14_0324	STM14_0324	putative cytoplasmic protein	0	NA
NC_01685(	RefSeq	CDS	318368	318853	+	0	ID=cds313;	0	17,33333333	STM14_0325	STM14_0325	putative cytoplasmic protein	2,415702814	-2,412509737
NC_01685(	RefSeq	CDS	318838	319221	+	0	ID=cds314;	0	2,5	STM14_0326	STM14_0326	hypothetical protein	0,284200331	-1,182723423
NC_01685(	RefSeq	CDS	319364	319849	+	0	ID=cds315;	0	0,5	STM14_0327	STM14_0327	putative cytoplasmic protein	0	NA
NC_01685(	RefSeq	CDS	319916	320452	+	0	ID=cds316;	0	1,5	STM14_0328	STM14_0328	putative outer membrane lipoprotein	0,284200331	-1,182723423
NC_01685(	RefSeq	CDS	320456	321799	+	0	ID=cds317;	0	2,5	STM14_0329	STM14_0329	putative cytoplasmic protein	0,284200331	-1,182723423
NC_01685(	RefSeq	CDS	321796	323100	+	0	ID=cds318;	0	2	STM14_0330	STM14_0330	hypothetical protein	0,284200331	-1,182723423
NC_01685(	RefSeq	CDS	323105	323878	+	0	ID=cds319;	0	0	STM14_0331	STM14_0331	putative inner membrane protein	0	NA
NC_01685(	RefSeq	CDS	324081	324512	+	0	ID=cds320;	0	1	STM14_0332	STM14_0332	putative Shiga-like toxin A subunit	0,142100166	-0,623264422
NC_01685(	RefSeq	CDS	324546	328415	+	0	ID=cds321;	0	16,83333333	STM14_0333	STM14_0333	putative inner membrane protein	2,415702814	-2,412509737
NC_01685(	RefSeq	CDS	328415	329203	+	0	ID=cds322;	0	1,5	STM14_0334	STM14_0334	putative cytoplasmic protein	0,284200331	-1,182723423
NC_01685(	RefSeq	CDS	329200	329616	+	0	ID=cds323;	0	2	STM14_0335	STM14_0335	hypothetical protein	0,284200331	-1,182723423
NC_01685(	RefSeq	CDS	329640	330161	+	0	ID=cds324;	0	1,5	STM14_0336	STM14_0336	putative cytoplasmic protein	0,284200331	-1,182723423
NC_01685(	RefSeq	CDS	330303	330512	+	0	ID=cds325;	0	0	STM14_0337	STM14_0337	hypothetical protein	0	NA
NC_01685(	RefSeq	CDS	330558	332747	+	0	ID=cds326;	0	5,5	STM14_0338	STM14_0338	putative cytoplasmic protein	0,852600993	-1,98056697
NC_01685(	RefSeq	CDS	332771	333217	+	0	ID=cds327;	0	2	STM14_0339	STM14_0339	putative cytoplasmic protein	0,284200331	-1,182723423
NC_01685(	RefSeq	CDS	333236	337330	+	0	ID=cds328;	1	23,33333333	STM14_0340	STM14_0340	putative RHS-like protein	5,027626177	-0,555407513
NC_01685(	RefSeq	CDS	337687	338520	+	0	ID=cds329;	8	2,5	STM14_0341	STM14_0341	putative RHS-like protein	14,35877929	3,455111456
NC_01685(	RefSeq	CDS	338514	338960	+	0	ID=cds330;	0	4	STM14_0342	STM14_0342	putative cytoplasmic protein	0,568400662	-1,710438693
NC_01685(	RefSeq	CDS	339144	339275	+	0	ID=cds331;	0	0	STM14_0343	STM14_0343	hypothetical protein	0	NA
NC_01685(	RefSeq	CDS	339345	339485	+	0	ID=cds332;	0	7,333333333	STM14_0344	STM14_0344	hypothetical protein	0,994701159	-2,072978628
NC_01685(	RefSeq	CDS	339523	339960	+	0	ID=cds333;	0	2	STM14_0345	STM14_0345	putative cytoplasmic protein	0,284200331	-1,182723423
NC_01685(	RefSeq	CDS	340013	340288	-	0	ID=cds334;	0	0	STM14_0346	STM14_0346	hypothetical protein	0	NA
NC_01685(	RefSeq	CDS	340729	341037	+	0	ID=cds335;	0	0	STM14_0348	STM14_0348	putative cytoplasmic protein	0	NA
NC_01685(	RefSeq	CDS	341440	341637	+	0	ID=cds336;	0	0	STM14_0350	STM14_0350	putative transposase	0	NA
NC_01685(	RefSeq	CDS	341863	342144	+	0	ID=cds337;	0	0	STM14_0351	STM14_0351	hypothetical protein	0	NA
NC_01685(	RefSeq	CDS	342894	343406	+	0	ID=cds338;	1	2	STM14_0352	safA	putative outer membrane protein	2,043522701	1,824239109
NC_01685(	RefSeq	CDS	343514	344227	+	0	ID=cds339;	0	1	STM14_0353	safB	putative fimbrial assembly chaperone	0,142100166	-0,623264422
NC_01685(	RefSeq	CDS	344251	346761	+	0	ID=cds340;	0	7,5	STM14_0354	safC	putative fimbrial usher	1,136801324	-2,147403465
NC_01685(	RefSeq	CDS	346783	347253	+	0	ID=cds341;	0	2	STM14_0355	safD	putative fimbrial subunit	0,284200331	-1,182723423
NC_01685(	RefSeq	CDS	347659	348480	+	0	ID=cds342;	0	2,333333333	STM14_0356	ybeJ	putative xylanase%2Fchitin deacetylase	0,284200331	-1,182723423
NC_01685(	RefSeq	CDS	348560	348820	+	0	ID=cds343;	0	0	STM14_0357	STM14_0357	hypothetical protein	0	NA
NC_01685(	RefSeq	CDS	349295	350242	+	0	ID=cds344;	0	7	STM14_0358	sinR	transcriptional regulator	0,994701159	-2,072978628
NC_01685(	RefSeq	CDS	350359	350574	+	0	ID=cds345;	0	0,5	STM14_0359	STM14_0359	putative cytoplasmic protein	0	NA
NC_01685(	RefSeq	CDS	350578	351297	-	0	ID=cds346;	3	2,5	STM14_0360	STM14_0360	SapA-like protein	5,562167441	2,509670145
NC_01685(	RefSeq	CDS	351322	351450	-	0	ID=cds347;	0	0	STM14_0361	STM14_0361	hypothetical protein	0	NA
NC_01685(	RefSeq	CDS	351628	352035	-	0	ID=cds348;	0	3	STM14_0362	STM14_0362	VirG-like protein	0,426300497	-1,498211557
NC_01685(	RefSeq	CDS	352120	352227	-	0	ID=cds349;	0	0,285714286	STM14_0363	STM14_0363	hypothetical protein	0	NA
NC_01685(	RefSeq	CDS	352406	353173	-	0	ID=cds350;	0	10	STM14_0364	yafV	hypothetical protein	1,421001655	-2,256999172
NC_01685(	RefSeq	CDS	353282	355726	-	0	ID=cds351;	0	2	STM14_0365	fadE	acyl-CoA dehydrogenase	0,284200331	-1,182723423
NC_01685(	RefSeq	CDS	355966	356544	+	0	ID=cds352;	0	21,5	STM14_0366	gmhA	phosphoheptose isomerase	3,126203642	-2,431590384
NC_01685(	RefSeq	CDS	356757	357524	+	0	ID=cds353;	0	23	STM14_0367	yafJ	putative glutamine amidotransferase	3,268303807	-2,432747446
NC_01685(	RefSeq	CDS	357495	358235	-	0	ID=cds354;	0	3,5	STM14_0368	yafK	hypothetical protein	0,568400662	-1,710438693
NC_01685(	RefSeq	CDS	358485	359540	+	0	ID=cds355;	0	10,5	STM14_0369	dinP	DNA polymerase IV	1,421001655	-2,256999172
NC_01685(	RefSeq	CDS	360888	361502	+	0	ID=cds356;	0	4	STM14_0371	prfH	peptide chain release factor-like protein	0,568400662	-1,710438693
NC_01685(	RefSeq	CDS	361658	363115	-	0	ID=cds357;	0	96,5	STM14_0372	pepD	aminoacyl-histidine dipeptidase	13,64161589	-1,920025083
NC_01685(	RefSeq	CDS	363364	363822	+	0	ID=cds358;	0	8	STM14_0373	gpt	xanthine-guanine phosphoribosyltransferase	1,136801324	-2,147403465
NC_01685(	RefSeq	CDS	363911	365155	+	0	ID=cds359;	0	18	STM14_0374	frsA	fermentation%2Frespiration switch protein	2,55780298	-2,419166771
NC_01685(	RefSeq	CDS	365213	365614	+	0	ID=cds360;	0	15,5	STM14_0375	crl	DNA-binding transcriptional regulator Crl	2,273602649	-2,403729808
NC_01685(	RefSeq	CDS	365665	366717	-	0	ID=cds361;	0	0	STM14_0376	phoE	outer membrane phosphoporin protein E	0	NA
NC_01685(	RefSeq	CDS	366783	366935	-	0	ID=cds362;	0	0	STM14_0377	STM14_0377	hypothetical protein	0	NA
NC_01685(	RefSeq	CDS	367000	368103	+	0	ID=cds363;	0	56	STM14_0378	proB	gamma-glutamyl kinase	7,95760927	-2,130811198
NC_01685(	RefSeq	CDS	368115	369365	+	0	ID=cds364;	1	39,16666667	STM14_0379	proA	gamma-glutamyl phosphate reductase	7,301228826	-1,040766944
NC_01685(	RefSeq	CDS	369882	370682	-	0	ID=cds365;	0	0,5	STM14_0381	STM14_0381	IS3 transposase	0	NA
NC_01685(	RefSeq	CDS	371424	371747	+	0	ID=cds366;	0	20,5	STM14_0383	STM14_0383	putative cytoplasmic protein	2,842003311	-2,427810709
NC_01685(	RefSeq	CDS	372094	373326	+	0	ID=cds367;	0	3	STM14_0384	STM14_0384	putative permease	0,426300497	-1,498211557
NC_01685(	RefSeq	CDS	373458	374879	+	0	ID=cds368;	0	2,5	STM14_0385	STM14_0385	isopropylmalate isomerase large subunit	0,284200331	-1,182723423
NC_01685(	RefSeq	CDS	374881	375507	+	0	ID=cds369;	0	1,5	STM14_0386	STM14_0386	putative 3-isopropylmalate isomerase	0,284200331	-1,182723423
NC_01685(	RefSeq	CDS	375522	376400	+	0	ID=cds370;	0	3,25	STM14_0387	STM14_0387	putative fumarylacetoacetate hydrolase	0,426300497	-1,498211557
NC_01685(	RefSeq	CDS	376400	377314	+	0	ID=cds371;	0	1,75	STM14_0388	STM14_0388	putative hydrolase%2Facyltransferase	0,284200331	-1,182723423
NC_01685(	RefSeq	CDS	377348	378313	+	0	ID=cds372;	0	3	STM14_0389	STM14_0389	putative transcriptional regulator	0,426300497	-1,498211557
NC_01685(	RefSeq	CDS	378258	378530	-	0	ID=cds373;	0	0	STM14_0390	STM14_0390	putative cytoplasmic protein	0	NA
NC_01685(	RefSeq	CDS	379221	379619	+	0	ID=cds374;	0	1,5	STM14_0391	STM14_0391	putative outer membrane protein	0,284200331	-1,182723423
NC_01685(	RefSeq	CDS	379666	380424	-	0	ID=cds375;	0	0	STM14_0392	stbE	putative fimbrial chaperone	0	NA
NC_01685(	RefSeq	CDS	380390	381715	-	0	ID=cds376;	0	0,5	STM14_0393	stbD	putative fimbrial usher	0	NA
NC_01685(	RefSeq	CDS	381720	384287	-	0	ID=cds377;	1	2	STM14_0394	stbC	putative fimbrial usher	2,043522701	1,824239109
NC_01685(	RefSeq	CDS	384271	385032	-	0	ID=cds378;	0	0	STM14_0395	stbB	putative fimbrial chaperone	0	NA
NC_01685(	RefSeq	CDS	385094	385630	-	0	ID=cds379;	0	0	STM14_0396	stbA	putative fimbrial major subunit	0	NA
NC_01685(	RefSeq	CDS	385733	385843	-	0	ID=cds380;	0	0	STM14_0397	STM14_0397	hypothetical protein	0	NA
NC_01685(	RefSeq	CDS	386264	387028	+	0	ID=cds381;	0	4	STM14_0398	STM14_0398	putative inner membrane protein	0,568400662	-1,710438693
NC_01685(	RefSeq	CDS	387001	387501	+	0	ID=cds382;	0	3	STM14_0399	STM14_0399	hypothetical protein	0,426300497	-1,498211557
NC_01685(	RefSeq	CDS	387801	389375	+	0	ID=cds383;	0	4,5	STM14_0400	STM14_0400	hypothetical protein	0,568400662	-1,710438693
NC_01685(	RefSeq	CDS	389475	390218	+	0	ID=cds384;	0	10	STM14_0401	STM14_0401	putative response regulator	1,421001655	-2,256999172
NC_01685(	RefSeq	CDS	390191	390679	+	0	ID=cds385;	0	2	STM14_0402	STM14_0402	putative inner membrane protein	0,284200331	-1,182723423
NC_01685(	RefSeq	CDS	390802	390939	-	0	ID=cds386;	0	0	STM14_0403	STM14_0403	hypothetical protein	0	NA
NC_01685(	RefSeq	CDS	391081	391605	+	0	ID=cds387;	0	0,5	STM14_0404	STM14_0404	putative outer membrane protein	0	NA
NC_01685(	RefSeq	CDS	391749	392390	+	0	ID=cds388;	0	1	STM14_0405	STM14_0405	putative response regulator	0,142100166	-0,623264422
NC_01685(	RefSeq	CDS	392401	392625	+	0	ID=cds389;	0	0	STM14_0406	STM14_0406	putative inner membrane protein	0	NA
NC_01685(	RefSeq	CDS	392683	393042	+	0	ID=cds390;	0	0,333333333	STM14_0407	STM14_0407	putative outer membrane lipoprotein	0	NA
NC_01685(	RefSeq	CDS	393112	394569	-	0	ID=cds391;	0	9	STM14_0408	STM14_0408	outer membrane efflux-like protein	1,27890149	-2,207741391
NC_01685(	RefSeq	CDS	394586	397753	-	0	ID=cds392;	0	0	STM14_0409	STM14_0409	putative cation efflux system protein	0	NA

NC_01685(	RefSeq	CDS	397750	398955	-	0	ID=cds393;	0	0	STM14_0410	STM14_0410	putative cation efflux pump	0	NA
NC_01685(	RefSeq	CDS	399120	399233	+	0	ID=cds394;	0	0	STM14_0411	STM14_0411	hypothetical protein	0	NA
NC_01685(	RefSeq	CDS	399253	401541	+	0	ID=cds395;	0	3	STM14_0412	STM14_0412	putative cation transport ATPase	0,426300497	-1,498211557
NC_01685(	RefSeq	CDS	401553	402017	+	0	ID=cds396;	0	2,5	STM14_0413	STM14_0413	putative transcriptional regulator	0,284200331	-1,182723423
NC_01685(	RefSeq	CDS	402095	402289	+	0	ID=cds397;	0	1,75	STM14_0414	STM14_0414	putative copper chaperone	0,284200331	-1,182723423
NC_01685(	RefSeq	CDS	402243	402422	+	0	ID=cds398;	0	0,25	STM14_0415	STM14_0415	putative metal-binding transport protein	0	NA
NC_01685(	RefSeq	CDS	402582	403835	+	0	ID=cds399;	0	2,5	STM14_0416	STM14_0416	putative inner membrane protein	0,284200331	-1,182723423
NC_01685(	RefSeq	CDS	404024	405982	+	0	ID=cds400;	2	31,91666667	STM14_0417	mod	DNA methylase	8,065850037	-0,242041354
NC_01685(	RefSeq	CDS	405989	408964	+	0	ID=cds401;	0	26,25	STM14_0418	res	DNA restriction enzyme	3,694604304	-2,438835558
NC_01685(	RefSeq	CDS	409084	409218	+	0	ID=cds402;	0	0	STM14_0419	STM14_0419	hypothetical protein	0	NA
NC_01685(	RefSeq	CDS	409118	409237	-	0	ID=cds403;	0	0	STM14_0420	STM14_0420	hypothetical protein	0	NA
NC_01685(	RefSeq	CDS	409241	409342	+	0	ID=cds404;	0	0,833333333	STM14_0421	STM14_0421	putative cytoplasmic protein	0,142100166	-0,623264422
NC_01685(	RefSeq	CDS	409459	410862	+	0	ID=cds405;	0	3	STM14_0422	STM14_0422	cytochrome BD2 subunit I	0,426300497	-1,498211557
NC_01685(	RefSeq	CDS	410859	411869	+	0	ID=cds406;	0	3,5	STM14_0423	STM14_0423	cytochrome BD2 subunit II	0,568400662	-1,710438693
NC_01685(	RefSeq	CDS	411886	412110	+	0	ID=cds407;	0	3,5	STM14_0424	STM14_0424	putative cytoplasmic protein	0,568400662	-1,710438693
NC_01685(	RefSeq	CDS	412206	413210	+	0	ID=cds408;	0	4	STM14_0425	STM14_0425	putative transcriptional regulator	0,568400662	-1,710438693
NC_01685(	RefSeq	CDS	413356	415464	+	0	ID=cds409;	0	3,5	STM14_0426	foxA	ferrioxamine receptor	0,568400662	-1,710438693
NC_01685(	RefSeq	CDS	415505	416137	-	0	ID=cds410;	0	4	STM14_0427	yahN	putative transport protein	0,568400662	-1,710438693
NC_01685(	RefSeq	CDS	416369	416644	+	0	ID=cds411;	0	0	STM14_0428	yahO	hypothetical protein	0	NA
NC_01685(	RefSeq	CDS	416780	418405	-	0	ID=cds412;	0	1	STM14_0429	prpR	prp operon regulator	0,142100166	-0,623264422
NC_01685(	RefSeq	CDS	418467	418640	+	0	ID=cds413;	0	0	STM14_0430	STM14_0430	hypothetical protein	0	NA
NC_01685(	RefSeq	CDS	418670	419557	+	0	ID=cds414;	0	34,66666667	STM14_0431	prpB	2-methylisocitrate lyase	4,973505794	-2,290637485
NC_01685(	RefSeq	CDS	419680	420849	+	0	ID=cds415;	0	2,5	STM14_0432	prpC	methylcitrate synthase	0,284200331	-1,182723423
NC_01685(	RefSeq	CDS	420889	422340	+	0	ID=cds416;	0	4	STM14_0433	prpD	2-methylcitrate dehydratase	0,568400662	-1,710438693
NC_01685(	RefSeq	CDS	422381	424267	+	0	ID=cds417;	0	11	STM14_0434	prpE	propionyl-CoA synthetase	1,563101821	-2,297202414
NC_01685(	RefSeq	CDS	424371	425345	-	0	ID=cds418;	3	50,5	STM14_0435	hemB	delta-aminolevulinic acid dehydratase	12,38297539	-0,292614157
NC_01685(	RefSeq	CDS	425565	425684	+	0	ID=cds419;	0	0	STM14_0436	STM14_0436	hypothetical protein	0	NA
NC_01685(	RefSeq	CDS	425675	425827	-	0	ID=cds420;	0	0	STM14_0437	STM14_0437	hypothetical protein	0	NA
NC_01685(	RefSeq	CDS	425858	428794	+	0	ID=cds421;	0	21,5	STM14_0438	yaiU	flagellar protein	3,126203642	-2,431590384
NC_01685(	RefSeq	CDS	428880	429503	+	0	ID=cds422;	0	2	STM14_0439	yaiV	putative DNA-binding transcriptional regulator	0,284200331	-1,182723423
NC_01685(	RefSeq	CDS	429548	430678	-	0	ID=cds423;	0	4,5	STM14_0440	ampH	beta-lactamase%2FD-alanine carboxypeptidase	0,568400662	-1,710438693
NC_01685(	RefSeq	CDS	430751	430909	-	0	ID=cds424;	0	0	STM14_0441	STM14_0441	hypothetical protein	0	NA
NC_01685(	RefSeq	CDS	430925	431059	+	0	ID=cds425;	0	1	STM14_0442	STM14_0442	hypothetical protein	0,142100166	-0,623264422
NC_01685(	RefSeq	CDS	431052	432272	+	0	ID=cds426;	0	29,5	STM14_0443	sbmA	transporter	4,263004966	-2,464060063
NC_01685(	RefSeq	CDS	432287	433381	+	0	ID=cds427;	3	71,33333333	STM14_0444	yaiW	putative outer membrane lipoprotein	15,36707886	-0,644051664
NC_01685(	RefSeq	CDS	433419	433727	-	0	ID=cds428;	0	4	STM14_0445	yaiY	putative inner membrane protein	0,568400662	-1,710438693
NC_01685(	RefSeq	CDS	433714	433827	+	0	ID=cds429;	0	0,25	STM14_0446	STM14_0446	hypothetical protein	0	NA
NC_01685(	RefSeq	CDS	433995	434210	+	0	ID=cds430;	1	32	STM14_0447	yaiZ	putative inner membrane protein	6,306527667	-0,86024894
NC_01685(	RefSeq	CDS	434236	435330	-	0	ID=cds431;	0	17,5	STM14_0448	ddl_1	D-alanyl-alanine synthetase A	2,55780298	-2,419166771
NC_01685(	RefSeq	CDS	435408	436121	+	0	ID=cds432;	0	2	STM14_0449	STM14_0449	putative inner membrane protein	0,284200331	-1,182723423
NC_01685(	RefSeq	CDS	436290	437501	+	0	ID=cds433;	0	4	STM14_0450	STM14_0450	putative permease	0,568400662	-1,710438693
NC_01685(	RefSeq	CDS	437620	437742	+	0	ID=cds434;	0	5	STM14_0451	STM14_0451	hypothetical protein	0,710500828	-1,864806942
NC_01685(	RefSeq	CDS	437792	438058	+	0	ID=cds435;	0	7,5	STM14_0452	yaiB	hypothetical protein	1,136801324	-2,147403465
NC_01685(	RefSeq	CDS	438082	438243	-	0	ID=cds436;	0	0	STM14_0453	STM14_0453	hypothetical protein	0	NA
NC_01685(	RefSeq	CDS	438410	438730	+	0	ID=cds437;	0	1	STM14_0454	psiF	hypothetical protein	0,142100166	-0,623264422
NC_01685(	RefSeq	CDS	438822	439934	+	0	ID=cds438;	0	0,5	STM14_0455	yaiC	diguanylate cyclase AdrA	0	NA
NC_01685(	RefSeq	CDS	439949	440758	-	0	ID=cds439;	0	22	STM14_0456	proC	pyrroline-5-carboxylate reductase	3,126203642	-2,431590384
NC_01685(	RefSeq	CDS	440792	440941	+	0	ID=cds440;	0	0	STM14_0457	STM14_0457	hypothetical protein	0	NA
NC_01685(	RefSeq	CDS	440898	441353	+	0	ID=cds441;	0	2,5	STM14_0458	yaiI	hypothetical protein	0,284200331	-1,182723423
NC_01685(	RefSeq	CDS	441538	442083	+	0	ID=cds442;	0	18	STM14_0459	aroL	shikimate kinase	2,55780298	-2,419166771
NC_01685(	RefSeq	CDS	442110	442301	+	0	ID=cds443;	0	9	STM14_0460	yaiA	hypothetical protein	1,27890149	-2,207741391
NC_01685(	RefSeq	CDS	442552	443229	+	0	ID=cds444;	0	13,5	STM14_0461	aroM	hypothetical protein	1,989402318	-2,376535557
NC_01685(	RefSeq	CDS	443300	443584	+	0	ID=cds445;	0	2	STM14_0462	yaiE	hypothetical protein	0,284200331	-1,182723423
NC_01685(	RefSeq	CDS	443622	444533	-	0	ID=cds446;	0	6	STM14_0463	rdgC	recombination associated protein	0,852600993	-1,98056697
NC_01685(	RefSeq	CDS	444556	444687	-	0	ID=cds447;	0	0	STM14_0464	STM14_0464	hypothetical protein	0	NA
NC_01685(	RefSeq	CDS	444659	445567	+	0	ID=cds448;	0	4,333333333	STM14_0465	yajF	fructokinase	0,568400662	-1,710438693
NC_01685(	RefSeq	CDS	445589	446761	-	0	ID=cds449;	0	5	STM14_0466	araJ	protein AraJ	0,710500828	-1,864806942
NC_01685(	RefSeq	CDS	446934	450074	-	0	ID=cds450;	0	12,5	STM14_0467	sbcC	exonuclease subunit SbcC	1,705201986	-2,329925387
NC_01685(	RefSeq	CDS	450071	451273	-	0	ID=cds451;	0	6,5	STM14_0468	sbcD	exonuclease subunit SbcD	0,852600993	-1,98056697
NC_01685(	RefSeq	CDS	451315	451446	-	0	ID=cds452;	0	0	STM14_0469	STM14_0469	hypothetical protein	0	NA
NC_01685(	RefSeq	CDS	451488	452177	+	0	ID=cds453;	1	21	STM14_0470	phoB	transcriptional regulator PhoB	4,743425846	-0,473101207
NC_01685(	RefSeq	CDS	452247	453542	+	0	ID=cds454;	0	15,5	STM14_0471	phoR	phosphate regulon sensor protein	2,273602649	-2,403729808
NC_01685(	RefSeq	CDS	453755	453904	-	0	ID=cds455;	0	0	STM14_0472	STM14_0472	hypothetical protein	0	NA
NC_01685(	RefSeq	CDS	453951	455270	+	0	ID=cds456;	0	78,66666667	STM14_0473	brnQ	branched-chain amino acid transporter	11,22591308	-1,998083373
NC_01685(	RefSeq	CDS	455345	456715	+	0	ID=cds457;	0	16,5	STM14_0474	proY	putative proline-specific permease	2,273602649	-2,403729808
NC_01685(	RefSeq	CDS	456877	458694	+	0	ID=cds458;	0	11,5	STM14_0475	malZ	maltodextrin glucosidase	1,705201986	-2,329925387
NC_01685(	RefSeq	CDS	458766	459368	-	0	ID=cds459;	0	73	STM14_0476	STM14_0476	putative thiol-alkyl hydroperoxide reductase	10,37331208	-2,029860184
NC_01685(	RefSeq	CDS	459578	460159	-	0	ID=cds460;	0	16	STM14_0477	yajB	acyl carrier protein phosphodiesterase	2,273602649	-2,403729808
NC_01685(	RefSeq	CDS	460253	461317	+	0	ID=cds461;	0	6	STM14_0478	queA	S-adenosylmethionine--tRNA ribosyltransferase-isomeras(	0,852600993	-1,98056697
NC_01685(	RefSeq	CDS	461340	461450	+	0	ID=cds462;	0	3,5	STM14_0479	STM14_0479	DNA metabolism protein	0,568400662	-1,710438693
NC_01685(	RefSeq	CDS	461514	462641	+	0	ID=cds463;	4,5	34,5	STM14_0480	tgt	queuine tRNA-ribosyltransferase	11,86869511	0,370542806
NC_01685(	RefSeq	CDS	462664	462996	+	0	ID=cds464;	1	34	STM14_0481	yajC	preprotein translocase subunit YajC	6,590727998	-0,915216377
NC_01685(	RefSeq	CDS	463024	464871	+	0	ID=cds465;	0	80	STM14_0482	secD	preprotein translocase subunit SecD	11,36801324	-1,993013165
NC_01685(	RefSeq	CDS	464882	465853	+	0	ID=cds466;	0	49,5	STM14_0483	secF	preprotein translocase subunit SecF	7,105008277	-2,173389122
NC_01685(	RefSeq	CDS	466001	466366	+	0	ID=cds467;	0	3	STM14_0484	STM14_0484	hypothetical protein	0,426300497	-1,498211557
NC_01685(	RefSeq	CDS	466392	467084	+	0	ID=cds468;	0	6,5	STM14_0485	STM14_0485	putative regulatory protein	0,852600993	-1,98056697
NC_01685(	RefSeq	CDS	467254	467601	+	0	ID=cds469;	0	10	STM14_0486	yajD	hypothetical protein	1,421001655	-2,256999172
NC_01685(	RefSeq	CDS	467631	467846	+	0	ID=cds470;	0	6	STM14_0487	STM14_0487	hypothetical protein	0,852600993	-1,98056697
NC_01685(	RefSeq	CDS	468230	469198	-	0	ID=cds471;	0	41	STM14_0489	tsx	nucleoside channel	5,826106787	-2,241137745
NC_01685(	RefSeq	CDS	469393	469932	-	0	ID=cds472;	0	2	STM14_0490	yajI	hypothetical protein	0,284200331	-1,182723423
NC_01685(	RefSeq	CDS	469965	470111	+	0	ID=cds473;	0	0,75	STM14_0491	STM14_0491	hypothetical protein	0,142100166	-0,623264422
NC_01685(	RefSeq	CDS	470084	470533	+	0	ID=cds474;	0	16,25	STM14_0492	ybaD	transcriptional regulator NrdR	2,273602649	-2,403729808
NC_01685(	RefSeq	CDS	470537	471640	+	0	ID=cds475;	0	26	STM14_0493	ribD	bifunctional diaminohydroxyphosphoribosylaminopyrimid	3,694604304	-2,438835558
NC_01685(	RefSeq	CDS	471729	472199	+	0	ID=cds476;	1	66	STM14_0494	ribH	riboflavin synthase subunit beta	11,1379333	-1,549301265
NC_01685(	RefSeq	CDS	472220	472639	+	0	ID=cds477;	0	33	STM14_0495	nusB	transcription antitermination protein NusB	4,689305463	-2,505858154
NC_01685(	RefSeq	CDS	472718	473695	+	0	ID=cds478;	0	25,25	STM14_0496	thiL	thiamine monophosphate kinase	3,552504138	-2,43600272
NC_01685(	RefSeq	CDS	473673	474188	+	0	ID=cds479;	0	5,25	STM14_0497	pgpA	phosphatidylglycerophosphatase A	0,710500828	-1,864806942
NC_01685(	RefSeq	CDS	473976	474284	-	0	ID=cds480;	0	0,5	STM14_0498	STM14_0498	hypothetical protein	0	NA
NC_01685(	RefSeq	CDS	474292	475266	-	0	ID=cds481;	2	19,5	STM14_0499	yajO	putative oxidoreductase	6,360648051	0,199657938
NC_01685(	RefSeq	CDS	475323	477185	-	0	ID=cds482;	0	68,5	STM14_0500	dxs	1-deoxy-D-xylulose-5-phosphate synthase	9,662811256	-2,058244678
NC_01685(	RefSeq	CDS	477209	478108	-	0	ID=cds483;	0	43,83333333	STM14_0501	ispA	geranyltranstransferase	6,252407284	-2,216733793
NC_01685(	RefSeq	CDS	478109	478351	-	0	ID=cds484;	0	16	STM14_0502	xseB	exodeoxyribonuclease VII small subunit	2,273602649	-2,403729808
NC_01685(	RefSeq	CDS	478561	480009	+	0	ID=cds485;	0	25,33333333	STM14_0503	thiI	thiamine biosynthesis protein ThiI	3,552504138	-2,43600272
NC_01685(	RefSeq	CDS	480053	480850	-	0	ID=cds486;	0	1	STM14_0504	phnV	2-aminoethylphosphonate transporter	0,142100166	-0,623264422
NC_01685(	RefSeq	CDS	480853	481713	-	0	ID=cds487;	0	0	STM14_0505	phnU	2-aminoethylphosphonate transporter	0	NA
NC_01685(	RefSeq	CDS	481716	482825	-	0	ID=cds488;	0	1	STM14_0506	phnT	2-aminoethylphosphonate transporter	0,142100166	-0,623264422
NC_01685(	RefSeq	CDS	482831	483844	-	0	ID=cds489;	0	1	STM14_0507	phnS	2-aminoethylphosphonate transporter	0,142100166	-0,623264422
NC_01685(	RefSeq	CDS	484042	484761	-	0	ID=cds490;	0	0	STM14_0508	phnR	2-aminoethylphosphonate transport protein	0	NA
NC_01685(	RefSeq	CDS	484792	484902	-	0	ID=cds491;	0	0	STM14_0509	STM14_0509	hypothetical protein	0	NA
NC_01685(	RefSeq	CDS	484901	486004	+	0	ID=cds492;	0	0	STM14_0510	phnW	2-aminoethylphosphonate--pyruvate transaminase	0	NA
NC_01685(	RefSeq	CDS	486011	486823	+	0	ID=cds493;	2	0,5	STM14_0511	phnX	phosphonoacetaldehyde hydrolase	3,51864474	1,925445802
NC_01685(	RefSeq	CDS	486920	487510	-	0	ID=cds494;	0	0,5	STM14_0512	thiJ	hypothetical protein	0	NA
NC_01685(	RefSeq	CDS	487473	488384	-	0	ID=cds495;	0	10	STM14_0513	apbA	2-dehydropantoate 2-reductase	1,421001655	-2,256999172
NC_01685(	RefSeq	CDS	488510	489001	+	0	ID=cds496;	0	12,5	STM14_0514	yajQ	nucleotide-binding protein	1,705201986	-2,329925387
NC_01685(	RefSeq	CDS	489048	490412	-	0	ID=cds497;	0	13,5	STM14_0515	yajR	putative transport protein	1,989402318	-2,376535557
NC_01685(	RefSeq	CDS	490949	492460	+	0	ID=cds498;	0	2	STM14_0517	STM14_0517	hypothetical protein	0,284200331	-1,182723423
NC_01685(	RefSeq	CDS	492472	493965	+	0	ID=cds499;	0	2	STM14_0518	STM14_0518	hypothetical protein	0,284200331	-1,182723423
NC_01685(	RefSeq	CDS	494047	494937	-	0	ID=cds500;	0	5	STM14_0519	cyoE	protoheme IX farnesyltransferase	0,710500828	-1,864806942
NC_01685(	RefSeq	CDS	494949	495278	-	0	ID=cds501;	0	1	STM14_0520	cyoD	cytochrome o ubiquinol oxidase subunit IV	0,142100166	-0,623264422
NC_01685(	RefSeq	CDS	495278	495892	-	0	ID=cds502;	0	5	STM14_0521	cyoC	cytochrome o ubiquinol oxidase subunit III	0,710500828	-1,864806942
NC_01685(	RefSeq	CDS	495882	497873	-	0	ID=cds503;	3	10	STM14_0522	cyoB	cytochrome o ubiquinol oxidase subunit I	6,698968765	1,219842116
NC_01685(	RefSeq	CDS	497884	498840	-	0	ID=cds504;	6	2	STM14_0523	cyoA	cytochrome o ubiquinol oxidase subunit II	10,84013455	3,166533115
NC_01685(	RefSeq	CDS	499293	500768	-	0	ID=cds505;	0	12,5	STM14_0524	ampG	muropeptide transporter	1,705201986	-2,329925387
NC_01685(	RefSeq	CDS	500812	501390	-	0	ID=cds506;	0	6,5	STM14_0525	yajG	hypothetical protein	0,852600993	-1,98056697
NC_01685(	RefSeq	CDS	501468	501653	+	0	ID=cds507;	0	0,5	STM14_0526	STM14_0526	hypothetical protein	0	NA
NC_01685(	RefSeq	CDS	501694	502011	+	0	ID=cds508;	0	3	STM14_0527	bolA	transcriptional regulator BolA	0,426300497	-1,498211557
NC_01685(	RefSeq	CDS	502053	502166	-	0	ID=cds509;	0	0	STM14_0528	STM14_0528	hypothetical protein	0	NA
NC_01685(	RefSeq	CDS	502358	503656	+	0	ID=cds510;	1	129,5	STM14_0529	tig	trigger factor	20,23234389	-2,19481852
NC_01685(	RefSeq	CDS	503903	504526	+	0	ID=cds511;	0	79,33333333	STM14_0530	clpP	ATP-dependent Clp protease proteolytic subunit	11,22591308	-1,998083373
NC_01685(	RefSeq	CDS	504670	504774	+	0	ID=cds512;	0	16	STM14_0531	STM14_0531	hypothetical protein	2,273602649	-2,403729808
NC_01685(	RefSeq	CDS	504778	506049	+	0	ID=cds513;	0	111	STM14_0532	clpX	ATP-dependent protease ATP-binding subunit	15,77311837	-1,865726942
NC_01685(	RefSeq	CDS	506235	508589	+	0	ID=cds514;	0	80,5	STM14_0533	lon	DNA-binding ATP-dependent protease La	11,36801324	-1,993013165
NC_01685(	RefSeq	CDS	508798	509070	+	0	ID=cds515;	2	370	STM14_0534	hupB	transcriptional regulator HU subunit beta	56,09570599	-2,586743437
NC_01685(	RefSeq	CDS	509361	511232	+	0	ID=cds516;	0	49	STM14_0535	cypD	peptidyl-prolyl cis-trans isomerase	6,962908111	-2,178239812

NC_01685(	RefSeq	CDS	511028	511390	-	0	ID=cds517;	0	0	STM14_0536	STM14_0536	hypothetical protein	0	NA
NC_01685(	RefSeq	CDS	511382	511756	+	0	ID=cds518;	0	1	STM14_0537	ybaV	putative DNA uptake protein	0,142100166	-0,623264422
NC_01685(	RefSeq	CDS	511860	512258	+	0	ID=cds519;	0	3	STM14_0538	ybaW	putative esterase	0,426300497	-1,498211557
NC_01685(	RefSeq	CDS	512364	513059	-	0	ID=cds520;	0	12,5	STM14_0539	ybaX	queuosine biosynthesis protein QueC	1,705201986	-2,329925387
NC_01685(	RefSeq	CDS	513124	514824	-	0	ID=cds521;	0	3	STM14_0540	ybaE	putative ABC transporter periplasmic binding protein	0,426300497	-1,498211557
NC_01685(	RefSeq	CDS	514925	515743	+	0	ID=cds522;	0	26,5	STM14_0541	cof	putative hydrolase	3,694604304	-2,438835558
NC_01685(	RefSeq	CDS	515792	516847	-	0	ID=cds523;	0	2,5	STM14_0542	STM14_0542	putative cysteine synthase%2Fcystathionine beta-synthas	0,284200331	-1,182723423
NC_01685(	RefSeq	CDS	516834	517418	+	0	ID=cds524;	0	4	STM14_0543	ybaO	putative transcriptional regulator	0,568400662	-1,710438693
NC_01685(	RefSeq	CDS	517459	519231	+	0	ID=cds525;	0	4,25	STM14_0544	mdlA	putative multidrug transporter membrane%5CATP-binding	0,568400662	-1,710438693
NC_01685(	RefSeq	CDS	519224	521005	+	0	ID=cds526;	0	19,75	STM14_0545	mdlB	putative multidrug transporter membrane%5CATP-binding	2,842003311	-2,427810709
NC_01685(	RefSeq	CDS	521218	521556	+	0	ID=cds527;	0	0	STM14_0546	glnK	nitrogen regulatory protein P-II 2	0	NA
NC_01685(	RefSeq	CDS	521588	522874	+	0	ID=cds528;	0	3	STM14_0547	amtB	ammonium transporter	0,426300497	-1,498211557
NC_01685(	RefSeq	CDS	522974	523834	-	0	ID=cds529;	0	3,5	STM14_0548	tesB	acyl-CoA thioesterase II	0,568400662	-1,710438693
NC_01685(	RefSeq	CDS	524049	524618	+	0	ID=cds530;	0	2,5	STM14_0549	ybaY	hypothetical protein	0,284200331	-1,182723423
NC_01685(	RefSeq	CDS	524651	525040	-	0	ID=cds531;	0	1,5	STM14_0550	ybaZ	putative methyltransferase	0,284200331	-1,182723423
NC_01685(	RefSeq	ncRNA	525118	525233	+	.	ID=rna8;Pa	4,5	20,75	STM14_0551	ffs	4.5S ribosomal RNA	10,02139296	0,829530494
NC_01685(	RefSeq	CDS	525271	526821	-	0	ID=cds532;	0	3,833333333	STM14_0552	ylaB	hypothetical protein	0,568400662	-1,710438693
NC_01685(	RefSeq	CDS	527046	527306	+	0	ID=cds533;	0	0	STM14_0553	rpmE2	50S ribosomal protein L31	0	NA
NC_01685(	RefSeq	CDS	527312	527452	+	0	ID=cds534;	0	2	STM14_0554	rpmJ_1	50S ribosomal protein L36	0,284200331	-1,182723423
NC_01685(	RefSeq	CDS	527508	527978	-	0	ID=cds535;	0	7,5	STM14_0555	ylaC	putative inner membrane protein	1,136801324	-2,147403465
NC_01685(	RefSeq	CDS	528095	528646	-	0	ID=cds536;	0	12	STM14_0556	maa	maltose O-acetyltransferase	1,705201986	-2,329925387
NC_01685(	RefSeq	CDS	528825	529043	-	0	ID=cds537;	0	20	STM14_0557	hha	hemolysin expression-modulating protein	2,842003311	-2,427810709
NC_01685(	RefSeq	CDS	529071	529445	-	0	ID=cds538;	0	12,5	STM14_0558	ybaJ	hypothetical protein	1,705201986	-2,329925387
NC_01685(	RefSeq	CDS	529941	533090	-	0	ID=cds539;	0	93,5	STM14_0559	acrB	acridine efflux pump	13,35741556	-1,928482877
NC_01685(	RefSeq	CDS	533113	534306	-	0	ID=cds540;	0	41,5	STM14_0560	acrA	acridine efflux pump	5,968206953	-2,232890319
NC_01685(	RefSeq	CDS	534448	535101	+	0	ID=cds541;	0	7	STM14_0561	acrR	DNA-binding transcriptional repressor AcrR	0,994701159	-2,072978628
NC_01685(	RefSeq	CDS	535085	535210	-	0	ID=cds542;	0	0	STM14_0562	STM14_0562	hypothetical protein	0	NA
NC_01685(	RefSeq	CDS	535220	538582	+	0	ID=cds543;	2	46,5	STM14_0563	aefA	potassium efflux protein KefA	10,05525235	-0,5930277
NC_01685(	RefSeq	CDS	538624	539559	-	0	ID=cds544;	1	12	STM14_0564	STM14_0564	putative transposase	3,464524356	0,028939145
NC_01685(	RefSeq	CDS	539629	539796	-	0	ID=cds545;	1	0	STM14_0565	ybaM	hypothetical protein	1,75932237	1,868512432
NC_01685(	RefSeq	CDS	539810	540325	-	0	ID=cds546;	0	0,5	STM14_0566	priC	primosomal replication protein N%27%27	0	NA
NC_01685(	RefSeq	CDS	540406	540783	+	0	ID=cds547;	0	2	STM14_0567	ybaN	hypothetical protein	0,284200331	-1,182723423
NC_01685(	RefSeq	CDS	540936	541487	+	0	ID=cds548;	0	4	STM14_0568	apt	adenine phosphoribosyltransferase	0,568400662	-1,710438693
NC_01685(	RefSeq	CDS	541601	543529	+	0	ID=cds549;	0	53	STM14_0569	dnaX	DNA polymerase III subunits gamma and tau	7,531308773	-2,151665531
NC_01685(	RefSeq	CDS	543575	543904	+	0	ID=cds550;	0	22	STM14_0570	ybaB	hypothetical protein	3,126203642	-2,431590384
NC_01685(	RefSeq	CDS	543904	544509	+	0	ID=cds551;	0	9	STM14_0571	recR	recombination protein RecR	1,27890149	-2,207741391
NC_01685(	RefSeq	CDS	544596	546494	+	0	ID=cds552;	0	26	STM14_0572	htpG	heat shock protein 90	3,694604304	-2,438835558
NC_01685(	RefSeq	CDS	546597	546818	-	0	ID=cds553;	0	0	STM14_0573	STM14_0573	hypothetical protein	0	NA
NC_01685(	RefSeq	CDS	546735	547379	+	0	ID=cds554;	0	40	STM14_0574	adk	adenylate kinase	5,684006621	-2,24950002
NC_01685(	RefSeq	CDS	547392	547598	+	0	ID=cds555;	1	2,5	STM14_0575	STM14_0575	hypothetical protein	2,043522701	1,824239109
NC_01685(	RefSeq	CDS	547608	548570	+	0	ID=cds556;	1	6	STM14_0576	hemH	ferrochelatase	2,611923363	0,709005733
NC_01685(	RefSeq	CDS	548567	549538	-	0	ID=cds557;	0	2	STM14_0577	aes	acetyl esterase	0,284200331	-1,182723423
NC_01685(	RefSeq	CDS	549691	550995	+	0	ID=cds558;	0	8	STM14_0578	gsk	inosine-guanosine kinase	1,136801324	-2,147403465
NC_01685(	RefSeq	CDS	551044	552720	-	0	ID=cds559;	0	14	STM14_0579	ybaL	putative cation:proton antiport protein	1,989402318	-2,376535557
NC_01685(	RefSeq	CDS	552798	552923	+	0	ID=cds560;	0	0	STM14_0580	STM14_0580	hypothetical protein	0	NA
NC_01685(	RefSeq	CDS	552935	554155	-	0	ID=cds561;	0	1	STM14_0581	fsr	putative transport protein	0,142100166	-0,623264422
NC_01685(	RefSeq	CDS	554328	555980	+	0	ID=cds562;	0	23,5	STM14_0582	ushA	bifunctional UDP-sugar hydrolase%2F5%27-nucleotidase p	3,410403973	-2,434086198
NC_01685(	RefSeq	CDS	556097	556576	-	0	ID=cds563;	0	11,5	STM14_0583	ybaK	hypothetical protein	1,705201986	-2,329925387
NC_01685(	RefSeq	CDS	556778	557572	-	0	ID=cds564;	0	3	STM14_0584	ybaP	putative cytoplasmic protein	0,426300497	-1,498211557
NC_01685(	RefSeq	CDS	557628	558485	-	0	ID=cds565;	0	2	STM14_0585	STM14_0585	hypothetical protein	0,284200331	-1,182723423
NC_01685(	RefSeq	CDS	558639	561140	-	0	ID=cds566;	0	29,5	STM14_0586	copA	copper exporting ATPase	4,263004966	-2,464060063
NC_01685(	RefSeq	CDS	561250	561666	+	0	ID=cds567;	0	9,5	STM14_0587	cueR	DNA-binding transcriptional regulator CueR	1,421001655	-2,256999172
NC_01685(	RefSeq	CDS	561667	562119	-	0	ID=cds568;	0	0	STM14_0588	ybbJ	hypothetical protein	0	NA
NC_01685(	RefSeq	CDS	562116	563033	-	0	ID=cds569;	0	8	STM14_0589	ybbK	putative inner membrane protein	1,136801324	-2,147403465
NC_01685(	RefSeq	CDS	563180	563857	+	0	ID=cds570;	0	1	STM14_0590	ybbL	putative ABC transporter ATP-binding protein YbbL	0,142100166	-0,623264422
NC_01685(	RefSeq	CDS	563844	564623	+	0	ID=cds571;	0	3	STM14_0591	ybbM	putative transport protein	0,426300497	-1,498211557
NC_01685(	RefSeq	CDS	564709	565563	-	0	ID=cds572;	0	13,5	STM14_0592	ybbN	putative thioredoxin protein	1,989402318	-2,376535557
NC_01685(	RefSeq	CDS	565623	566393	-	0	ID=cds573;	0	11,5	STM14_0593	ybbO	short chain dehydrogenase	1,705201986	-2,329925387
NC_01685(	RefSeq	CDS	566548	567162	-	0	ID=cds574;	0	3,5	STM14_0594	tesA	multifunctional acyl-CoA thioesterase I%2Fprotease I%2Fl	0,568400662	-1,710438693
NC_01685(	RefSeq	CDS	567133	567819	+	0	ID=cds575;	0	1,5	STM14_0595	ybbA	putative ABC transporter ATP-binding protein YbbA	0,284200331	-1,182723423
NC_01685(	RefSeq	CDS	567816	570230	+	0	ID=cds576;	1	17,5	STM14_0596	ybbP	putative inner membrane protein	4,31712535	-0,335890347
NC_01685(	RefSeq	CDS	570227	570331	+	0	ID=cds577;	0	0	STM14_0597	STM14_0597	hypothetical protein	0	NA
NC_01685(	RefSeq	CDS	570416	571549	+	0	ID=cds578;	0	3	STM14_0598	STM14_0598	putative outer membrane protein	0,426300497	-1,498211557
NC_01685(	RefSeq	CDS	571546	571650	-	0	ID=cds579;	0	0	STM14_0599	STM14_0599	hypothetical protein	0	NA
NC_01685(	RefSeq	CDS	571806	572636	+	0	ID=cds580;	0	1	STM14_0600	sfbA	ABC transporter ATP-binding protein	0,142100166	-0,623264422
NC_01685(	RefSeq	CDS	572673	573689	+	0	ID=cds581;	0	0	STM14_0601	sfbB	ABC transporter ATP-binding protein	0	NA
NC_01685(	RefSeq	CDS	573682	574341	+	0	ID=cds582;	0	2	STM14_0602	sfbC	putative ABC transporter permease	0,284200331	-1,182723423
NC_01685(	RefSeq	CDS	574380	575474	-	0	ID=cds583;	0	32,5	STM14_0603	ybbB	tRNA 2-selenouridine synthase	4,547205297	-2,489878815
NC_01685(	RefSeq	CDS	575544	576470	-	0	ID=cds584;	0	1	STM14_0604	ybbS	DNA-binding transcriptional activator AllS	0,142100166	-0,623264422
NC_01685(	RefSeq	CDS	576697	577179	+	0	ID=cds585;	0	2	STM14_0605	allA	ureidoglycolate hydrolase	0,284200331	-1,182723423
NC_01685(	RefSeq	CDS	577258	578076	+	0	ID=cds586;	0	13	STM14_0606	allR	DNA-binding transcriptional repressor AllR	1,847302152	-2,356210635
NC_01685(	RefSeq	CDS	578161	579942	+	0	ID=cds587;	0	2	STM14_0607	gcl	glyoxylate carboligase	0,284200331	-1,182723423
NC_01685(	RefSeq	CDS	579955	580731	+	0	ID=cds588;	0	0	STM14_0608	gip	hydroxypyruvate isomerase	0	NA
NC_01685(	RefSeq	CDS	580832	581710	+	0	ID=cds589;	0	1,5	STM14_0609	glxR	tartronic semialdehyde reductase	0,284200331	-1,182723423
NC_01685(	RefSeq	CDS	581776	583023	+	0	ID=cds590;	0	0	STM14_0610	STM14_0610	putative permease	0	NA
NC_01685(	RefSeq	CDS	583099	583353	+	0	ID=cds591;	0	0	STM14_0611	ybbV	putative cytoplasmic protein	0	NA
NC_01685(	RefSeq	CDS	583119	584573	+	0	ID=cds592;	0	0	STM14_0612	allP	allantoin permease	0	NA
NC_01685(	RefSeq	CDS	584652	586013	+	0	ID=cds593;	0	1,5	STM14_0613	allB	allantoinase	0,284200331	-1,182723423
NC_01685(	RefSeq	CDS	586072	587370	+	0	ID=cds594;	0	0	STM14_0614	ybbY	putative purine permease YbbY	0	NA
NC_01685(	RefSeq	CDS	587393	588541	+	0	ID=cds595;	0	0	STM14_0615	glxK	glycerate kinase	0	NA
NC_01685(	RefSeq	CDS	588621	589406	-	0	ID=cds596;	0	7,5	STM14_0616	ylbA	hypothetical protein	1,136801324	-2,147403465
NC_01685(	RefSeq	CDS	589417	590652	-	0	ID=cds597;	0	5,5	STM14_0617	allC	allantoate amidohydrolase	0,852600993	-1,98056697
NC_01685(	RefSeq	CDS	590674	591723	-	0	ID=cds598;	0	1	STM14_0618	allD	ureidoglycolate dehydrogenase	0,142100166	-0,623264422
NC_01685(	RefSeq	CDS	592054	593718	+	0	ID=cds599;	0	1	STM14_0619	fdrA	membrane protein FdrA	0,142100166	-0,623264422
NC_01685(	RefSeq	CDS	593728	594987	+	0	ID=cds600;	0	1	STM14_0620	ylbE	putative cytoplasmic protein	0,142100166	-0,623264422
NC_01685(	RefSeq	CDS	594999	595808	+	0	ID=cds601;	0	0	STM14_0621	ylbF	putative cytoplasmic protein	0	NA
NC_01685(	RefSeq	CDS	595812	596705	+	0	ID=cds602;	0	0	STM14_0622	arcC	carbamate kinase	0	NA
NC_01685(	RefSeq	CDS	596746	597813	-	0	ID=cds603;	0	7	STM14_0623	purK	phosphoribosylaminoimidazole carboxylase ATPase subur	0,994701159	-2,072978628
NC_01685(	RefSeq	CDS	597810	598319	-	0	ID=cds604;	0	3,5	STM14_0624	purE	phosphoribosylaminoimidazole carboxylase catalytic subu	0,568400662	-1,710438693
NC_01685(	RefSeq	CDS	598437	599159	-	0	ID=cds605;	0	8	STM14_0625	lpxH	UDP-2%2C3-diacylglucosamine hydrolase	1,136801324	-2,147403465
NC_01685(	RefSeq	CDS	599162	599656	-	0	ID=cds606;	0	16	STM14_0626	ppiB	peptidyl-prolyl cis-trans isomerase B	2,273602649	-2,403729808
NC_01685(	RefSeq	CDS	599829	601214	+	0	ID=cds607;	0	43,5	STM14_0627	cysS	cysteinyl-tRNA synthetase	6,252407284	-2,216733793
NC_01685(	RefSeq	CDS	601258	602082	-	0	ID=cds608;	0	2	STM14_0628	STM14_0628	putative outer membrane protein	0,284200331	-1,182723423
NC_01685(	RefSeq	CDS	602079	602516	-	0	ID=cds609;	0	1	STM14_0629	STM14_0629	putative inner membrane protein	0,142100166	-0,623264422
NC_01685(	RefSeq	CDS	602509	603054	-	0	ID=cds610;	0	0,5	STM14_0630	ybcI	putative membrane-bound metal-dependent hydrolase	0	NA
NC_01685(	RefSeq	CDS	603182	603394	-	0	ID=cds611;	0	6	STM14_0631	ybcJ	hypothetical protein	0,852600993	-1,98056697
NC_01685(	RefSeq	CDS	603396	604262	-	0	ID=cds612;	0	27	STM14_0632	folD	bifunctional 5%2C10-methylene-tetrahydrofolate dehydro	3,836704469	-2,44262576
NC_01685(	RefSeq	CDS	604316	604444	-	0	ID=cds613;	0	1	STM14_0633	STM14_0633	hypothetical protein	0,142100166	-0,623264422
NC_01685(	RefSeq	CDS	604400	604555	+	0	ID=cds614;	0	0	STM14_0634	STM14_0634	hypothetical protein	0	NA
NC_01685(	RefSeq	CDS	604824	605366	+	0	ID=cds615;	0	412,5	STM14_0635	fimA	fimbrin	58,5452682	-1,942259938
NC_01685(	RefSeq	CDS	605442	605975	+	0	ID=cds616;	0	71,5	STM14_0636	fimI	fimbrial protein	10,23121192	-2,035392961
NC_01685(	RefSeq	CDS	606019	606711	+	0	ID=cds617;	0	67	STM14_0637	fimC	periplasmic chaperone	9,520711091	-2,064144167
NC_01685(	RefSeq	CDS	606742	609354	+	0	ID=cds618;	0	132	STM14_0638	fimD	outer membrane usher protein	18,75722185	-1,863295025
NC_01685(	RefSeq	CDS	609369	610376	+	0	ID=cds619;	0	36,5	STM14_0639	fimH	minor fimbrial subunit	5,115605959	-2,281753063
NC_01685(	RefSeq	CDS	610386	610904	+	0	ID=cds620;	0	23,5	STM14_0640	fimF	putative fimbrial protein	3,410403973	-2,434086198
NC_01685(	RefSeq	CDS	610950	611582	-	0	ID=cds621;	0	9,5	STM14_0641	fimZ	transcriptional regulator FimZ	1,421001655	-2,256999172
NC_01685(	RefSeq	CDS	612186	612908	-	0	ID=cds622;	0	0	STM14_0642	fimY	putative regulatory protein	0	NA
NC_01685(	RefSeq	CDS	612927	613238	-	0	ID=cds623;	0	1	STM14_0643	STM14_0643	hypothetical protein	0,142100166	-0,623264422
NC_01685(	RefSeq	CDS	613400	613996	-	0	ID=cds624;	0	1	STM14_0644	fimW	putative fimbrial protein	0,142100166	-0,623264422
NC_01685(	RefSeq	CDS	614019	614189	+	0	ID=cds625;	0	0	STM14_0645	STM14_0645	hypothetical protein	0	NA
NC_01685(	RefSeq	CDS	614287	614475	-	0	ID=cds626;	0	1,333333333	STM14_0647	STM14_0647	integrase	0,142100166	-0,623264422
NC_01685(	RefSeq	CDS	615283	615441	+	0	ID=cds627;	0	0	STM14_0649	STM14_0649	hypothetical protein	0	NA
NC_01685(	RefSeq	CDS	615498	617150	-	0	ID=cds628;	0	25	STM14_0650	STM14_0650	putative inner membrane protein	3,552504138	-2,43600272
NC_01685(	RefSeq	CDS	617131	618057	-	0	ID=cds629;	0	6	STM14_0651	yfdH	putative glycosyltransferase	0,852600993	-1,98056697
NC_01685(	RefSeq	CDS	618054	618416	-	0	ID=cds630;	0	11,5	STM14_0652	gtrA	putative glycosyl translocase	1,705201986	-2,329925387
NC_01685(	RefSeq	CDS	619284	619613	-	0	ID=cds631;	0	0	STM14_0655	STM14_0655	sensor kinase	0	NA
NC_01685(	RefSeq	CDS	619737	619901	+	0	ID=cds632;	0	1	STM14_05870	STM14_05870	hypothetical protein	0,142100166	-0,623264422
NC_01685(	RefSeq	CDS	619952	620806	-	0	ID=cds633;	0	0	STM14_0657	STM14_0657	putative transcriptional regulator	0	NA
NC_01685(	RefSeq	CDS	621022	622347	+	0	ID=cds634;	0	1,5	STM14_0658	STM14_0658	pyridine nucleotide-disulfide oxidoreductase	0,284200331	-1,182723423
NC_01685(	RefSeq	CDS	622266	622520	-	0	ID=cds635;	0	0	STM14_0659	STM14_0659	hypothetical protein	0	NA
NC_01685(	RefSeq	CDS	622504	622740	+	0	ID=cds636;	0	0	STM14_0660	STM14_0660	hypothetical protein	0	NA
NC_01685(	RefSeq	CDS	622753	623313	+	0	ID=cds637;	0	0,5	STM14_0661	STM14_0661	putative inner membrane protein	0	NA
NC_01685(	RefSeq	CDS	623392	624567	-	0	ID=cds638;	0	10	STM14_0662	STM14_0662	putative DNA repair ATPase	1,421001655	-2,256999172
NC_01685(	RefSeq	CDS	624893	626287	+	0	ID=cds639;	0	14	STM14_0663	pheP	phenylalanine transporter	1,989402318	-2,376535557

NC_01685(	RefSeq	CDS	626329	627576	-	0	ID=cds640;	0	12	STM14_0664	ybdG	hypothetical protein	1,705201986	-2,329925387
NC_01685(	RefSeq	CDS	627603	627788	+	0	ID=cds641;	0	0	STM14_0665	STM14_0665	hypothetical protein	0	NA
NC_01685(	RefSeq	CDS	627887	629857	+	0	ID=cds642;	0	6	STM14_0666	apeE	outer membrane esterase	0,852600993	-1,98056697
NC_01685(	RefSeq	CDS	630024	632753	+	0	ID=cds643;	0	17,5	STM14_0667	STM14_0667	putative inner membrane protein	2,55780298	-2,419166771
NC_01685(	RefSeq	CDS	632881	633867	-	0	ID=cds644;	0	0	STM14_0668	STM14_0668	putative phosphosugar isomerase	0	NA
NC_01685(	RefSeq	CDS	633934	634977	-	0	ID=cds645;	0	2	STM14_0669	STM14_0669	putative inner membrane protein	0,284200331	-1,182723423
NC_01685(	RefSeq	CDS	635010	635864	-	0	ID=cds646;	0	1	STM14_0670	STM14_0670	putative PTS system mannose-specific enzyme IID	0,142100166	-0,623264422
NC_01685(	RefSeq	CDS	635867	636610	-	0	ID=cds647;	0	0	STM14_0671	STM14_0671	putative inner membrane protein	0	NA
NC_01685(	RefSeq	CDS	636625	637095	-	0	ID=cds648;	0	0	STM14_0672	STM14_0672	putative PTS system mannose-specific enzyme IIAB	0	NA
NC_01685(	RefSeq	CDS	637073	637522	-	0	ID=cds649;	0	0	STM14_0673	STM14_0673	putative PTS system mannose-specific enzyme IIAB	0	NA
NC_01685(	RefSeq	CDS	637722	638375	-	0	ID=cds650;	0	11,5	STM14_0674	nfnB	dihydropteridine reductase	1,705201986	-2,329925387
NC_01685(	RefSeq	CDS	638472	638840	-	0	ID=cds651;	0	5,5	STM14_0675	ybdF	hypothetical protein	0,852600993	-1,98056697
NC_01685(	RefSeq	CDS	638840	639421	-	0	ID=cds652;	0	4,5	STM14_0676	STM14_0676	putative regulatory protein	0,568400662	-1,710438693
NC_01685(	RefSeq	CDS	639577	639684	+	0	ID=cds653;	0	0,5	STM14_0677	STM14_0677	hypothetical protein	0	NA
NC_01685(	RefSeq	CDS	639710	640051	+	0	ID=cds654;	0	0,5	STM14_0678	STM14_0678	putative regulatory protein	0	NA
NC_01685(	RefSeq	CDS	640057	640305	-	0	ID=cds655;	0	2	STM14_0679	ybdJ	putative inner membrane protein	0,284200331	-1,182723423
NC_01685(	RefSeq	CDS	640372	641490	-	0	ID=cds656;	0	0,5	STM14_0680	ybdK	carboxylate-amine ligase	0	NA
NC_01685(	RefSeq	CDS	641502	642206	-	0	ID=cds657;	0	0	STM14_0681	entD	phosphopantetheinyltransferase component of enterobac	0	NA
NC_01685(	RefSeq	CDS	642252	644507	-	0	ID=cds658;	1	1	STM14_0682	fepA	outer membrane receptor FepA	1,901422535	2,462014545
NC_01685(	RefSeq	CDS	644504	644635	-	0	ID=cds659;	1	0	STM14_0683	STM14_0683	hypothetical protein	1,75932237	1,868512432
NC_01685(	RefSeq	CDS	644696	645910	+	0	ID=cds660;	2	0,5	STM14_0684	fes	enterobactin%2Fferric enterobactin esterase	3,51864474	1,925445802
NC_01685(	RefSeq	CDS	645941	646159	+	0	ID=cds661;	0	0,5	STM14_0685	ybdZ	putative cytoplasmic protein	0	NA
NC_01685(	RefSeq	CDS	646156	650040	+	0	ID=cds662;	0	4,5	STM14_0686	entF	enterobactin synthase subunit F	0,568400662	-1,710438693
NC_01685(	RefSeq	CDS	650290	651426	+	0	ID=cds663;	2	10,5	STM14_0687	fepE	ferric enterobactin transport protein FepE	4,939646395	0,828530271
NC_01685(	RefSeq	CDS	651479	652273	-	0	ID=cds664;	0	0	STM14_0688	fepC	iron-enterobactin transporter ATP-binding protein	0	NA
NC_01685(	RefSeq	CDS	652270	653259	-	0	ID=cds665;	0	0	STM14_0689	fepG	iron-enterobactin transporter permease	0	NA
NC_01685(	RefSeq	CDS	653259	654266	-	0	ID=cds666;	0	1	STM14_0690	fepD	iron-enterobactin transporter membrane protein	0,142100166	-0,623264422
NC_01685(	RefSeq	CDS	654377	655621	+	0	ID=cds667;	0	1,5	STM14_0691	ybdA	enterobactin exporter EntS	0,284200331	-1,182723423
NC_01685(	RefSeq	CDS	655684	656640	-	0	ID=cds668;	0	0	STM14_0692	fepB	iron-enterobactin transporter periplasmic binding protein	0	NA
NC_01685(	RefSeq	CDS	656949	658124	+	0	ID=cds669;	0	0	STM14_0693	entC	isochorismate synthase	0	NA
NC_01685(	RefSeq	CDS	658134	659744	+	0	ID=cds670;	0	0	STM14_0694	entE	enterobactin synthase subunit E	0	NA
NC_01685(	RefSeq	CDS	659758	660615	+	0	ID=cds671;	0	0	STM14_0695	entB	2%2C3-dihydro-2%2C3-dihydroxybenzoate synthetase	0	NA
NC_01685(	RefSeq	CDS	660615	661370	+	0	ID=cds672;	0	0,5	STM14_0696	entA	2%2C3-dihydroxybenzoate-2%2C3-dehydrogenase	0	NA
NC_01685(	RefSeq	CDS	661373	661786	+	0	ID=cds673;	0	1,5	STM14_0697	ybdB	hypothetical protein	0,284200331	-1,182723423
NC_01685(	RefSeq	CDS	661861	661971	+	0	ID=cds674;	0	2,5	STM14_0698	STM14_0698	hypothetical protein	0,284200331	-1,182723423
NC_01685(	RefSeq	CDS	661968	664073	+	0	ID=cds675;	4	43	STM14_0699	cstA	carbon starvation protein	13,1475966	0,140334179
NC_01685(	RefSeq	CDS	664137	664334	+	0	ID=cds676;	0	13	STM14_0700	ybdD	putative cytoplasmic protein	1,847302152	-2,356210635
NC_01685(	RefSeq	CDS	664370	665458	-	0	ID=cds677;	0	4	STM14_0701	ybdH	hypothetical protein	0,568400662	-1,710438693
NC_01685(	RefSeq	CDS	665585	666745	+	0	ID=cds678;	2	1,5	STM14_0702	ybdL	putative aminotransferase	3,802845071	2,200856121
NC_01685(	RefSeq	CDS	666746	667363	-	0	ID=cds679;	0	3,75	STM14_0703	ybdM	putative transcriptional regulator	0,568400662	-1,710438693
NC_01685(	RefSeq	CDS	667336	668571	-	0	ID=cds680;	0	4,25	STM14_0704	ybdN	putative 3%27-phosphoadenosine 5%27-phosphosulfate s	0,568400662	-1,710438693
NC_01685(	RefSeq	CDS	668737	669639	-	0	ID=cds681;	0	7	STM14_0705	ybdO	putative transcriptional regulator	0,994701159	-2,072978628
NC_01685(	RefSeq	CDS	669956	670702	-	0	ID=cds682;	0	7	STM14_0706	dsbG	disulfide isomerase%2Fthiol-disulfide oxidase	0,994701159	-2,072978628
NC_01685(	RefSeq	CDS	671143	671706	+	0	ID=cds683;	3	54,5	STM14_0707	ahpC	alkyl hydroperoxide reductase subunit C	12,95137605	-0,369382621
NC_01685(	RefSeq	CDS	671831	673513	+	0	ID=cds684;	0	19	STM14_0708	ahpF	alkyl hydroperoxide reductase F52a subunit	2,699903145	-2,42419778
NC_01685(	RefSeq	CDS	673845	674405	+	0	ID=cds685;	0	0	STM14_0709	STM14_0709	putative anaerobic dehydrogenase component	0	NA
NC_01685(	RefSeq	CDS	674398	676677	+	0	ID=cds686;	0	2,5	STM14_0710	STM14_0710	putative oxidoreductase protein	0,284200331	-1,182723423
NC_01685(	RefSeq	CDS	676674	677033	+	0	ID=cds687;	0	1	STM14_0711	STM14_0711	molybdopterin-containing oxidoreductase iron-sulfur subu	0,142100166	-0,623264422
NC_01685(	RefSeq	CDS	677033	677800	+	0	ID=cds688;	0	1	STM14_0712	STM14_0712	putative hydrogenase protein	0,142100166	-0,623264422
NC_01685(	RefSeq	CDS	677868	678296	-	0	ID=cds689;	0	25	STM14_0713	ybdQ	putative universal stress protein	3,552504138	-2,43600272
NC_01685(	RefSeq	CDS	678228	678434	+	0	ID=cds690;	0	0	STM14_0714	STM14_0714	hypothetical protein	0	NA
NC_01685(	RefSeq	CDS	678399	678539	-	0	ID=cds691;	0	0	STM14_0715	STM14_0715	hypothetical protein	0	NA
NC_01685(	RefSeq	CDS	678519	679757	+	0	ID=cds692;	0	1	STM14_0716	ybdR	putative dehydrogenase	0,142100166	-0,623264422
NC_01685(	RefSeq	CDS	679840	680250	-	0	ID=cds693;	0	20,5	STM14_0717	rnk	nucleoside diphosphate kinase regulator	2,842003311	-2,427810709
NC_01685(	RefSeq	CDS	680485	681291	-	0	ID=cds694;	2	41,83333333	STM14_0718	rna	ribonuclease I	9,486851692	-0,503856957
NC_01685(	RefSeq	CDS	681401	682864	-	0	ID=cds695;	0	38,83333333	STM14_0719	citT	citrate%2Fsuccinate transport antiport protein	5,541906456	-2,25579729
NC_01685(	RefSeq	CDS	682902	683798	-	0	ID=cds696;	0	29	STM14_0720	citG	triphosphoribosyl-dephospho-CoA synthase	4,120904801	-2,455089955
NC_01685(	RefSeq	CDS	683770	684321	-	0	ID=cds697;	0	35,25	STM14_0721	citX	2%27-%285%27%27-triphosphoribosyl%29-3%27-dephosp	4,973505794	-2,290637485
NC_01685(	RefSeq	CDS	684325	685854	-	0	ID=cds698;	0	71,75	STM14_0722	citF	citrate lyase subunit alpha%2Fcitrate-ACP transferase	10,23121192	-2,035392961
NC_01685(	RefSeq	CDS	685864	686772	-	0	ID=cds699;	0	35	STM14_0723	citE	citrate lyase subunit beta	4,973505794	-2,290637485
NC_01685(	RefSeq	CDS	686769	687065	-	0	ID=cds700;	0	33,5	STM14_0724	citD	citrate lyase subunit gamma	4,831405628	-2,523251848
NC_01685(	RefSeq	CDS	687062	688138	-	0	ID=cds701;	0	17	STM14_0725	citC	citrate lyase synthetase	2,415702814	-2,412509737
NC_01685(	RefSeq	CDS	688331	688513	+	0	ID=cds702;	0	0,5	STM14_0726	STM14_0726	hypothetical protein	0	NA
NC_01685(	RefSeq	CDS	688523	690184	+	0	ID=cds703;	0	23	STM14_0727	dpiB	sensory histidine kinase	3,268303807	-2,432747446
NC_01685(	RefSeq	CDS	690153	690833	+	0	ID=cds704;	0	10,5	STM14_0728	dpiA	two-component response regulator DpiA	1,421001655	-2,256999172
NC_01685(	RefSeq	CDS	690887	692272	-	0	ID=cds705;	0	19	STM14_0729	dcuC	C4-dicarboxylate transporter DcuC	2,699903145	-2,42419778
NC_01685(	RefSeq	CDS	692333	692443	+	0	ID=cds706;	0	1	STM14_0730	STM14_0730	hypothetical protein	0,142100166	-0,623264422
NC_01685(	RefSeq	CDS	692651	693223	+	0	ID=cds707;	0	22,5	STM14_0731	pagP	palmitoyl transferase for Lipid A	3,126203642	-2,431590384
NC_01685(	RefSeq	CDS	693411	693620	+	0	ID=cds708;	2	435,5	STM14_0732	cspE	cold shock protein CspE	65,47431691	-2,741246762
NC_01685(	RefSeq	CDS	693678	694061	-	0	ID=cds709;	3	2,5	STM14_0733	ccrB	camphor resistance protein CrcB	5,562167441	2,509670145
NC_01685(	RefSeq	CDS	694152	694940	+	0	ID=cds710;	0	4	STM14_0734	ybeM	putative hydrolase	0,568400662	-1,710438693
NC_01685(	RefSeq	CDS	694969	695076	+	0	ID=cds711;	0	1,5	STM14_0735	STM14_0735	hypothetical protein	0,284200331	-1,182723423
NC_01685(	RefSeq	CDS	695069	695272	+	0	ID=cds712;	0	13,5	STM14_0736	tatE	twin arginine translocase protein E	1,989402318	-2,376535557
NC_01685(	RefSeq	CDS	695358	696323	-	0	ID=cds713;	0,5	6	STM14_0737	lipA	lipoyl synthase	0,852600993	-1,98056697
NC_01685(	RefSeq	CDS	696364	696498	-	0	ID=cds714;	1,5	0	STM14_0738	STM14_0738	hypothetical protein	3,51864474	1,925445802
NC_01685(	RefSeq	CDS	696529	697482	-	0	ID=cds715;	0	0	STM14_0739	ybeF	putative DNA-binding transcriptional regulator	0	NA
NC_01685(	RefSeq	CDS	697584	697727	-	0	ID=cds716;	0	0	STM14_0740	STM14_0740	hypothetical protein	0	NA
NC_01685(	RefSeq	CDS	697784	698425	-	0	ID=cds717;	0	6	STM14_0741	lipB	lipoyltransferase	0,852600993	-1,98056697
NC_01685(	RefSeq	CDS	698354	698719	+	0	ID=cds718;	0	0	STM14_0742	STM14_0742	hypothetical protein	0	NA
NC_01685(	RefSeq	CDS	698525	698788	-	0	ID=cds719;	0	6	STM14_0743	ybeD	hypothetical protein	0,852600993	-1,98056697
NC_01685(	RefSeq	CDS	698897	700108	-	0	ID=cds720;	0	55	STM14_0744	dacA	D-alanyl-D-alanine carboxypeptidase	7,815509104	-2,137668811
NC_01685(	RefSeq	CDS	700128	700277	+	0	ID=cds721;	0	0	STM14_0745	STM14_0745	hypothetical protein	0	NA
NC_01685(	RefSeq	CDS	700248	701393	-	0	ID=cds722;	0	29	STM14_0746	rlpA	rare lipoprotein A	4,120904801	-2,455089955
NC_01685(	RefSeq	CDS	701404	702516	-	0	ID=cds723;	0	11,5	STM14_0747	mrdB	cell wall shape-determining protein	1,705201986	-2,329925387
NC_01685(	RefSeq	CDS	702519	704420	-	0	ID=cds724;	0	16	STM14_0748	mrdA	penicillin-binding protein 2	2,273602649	-2,403729808
NC_01685(	RefSeq	CDS	704451	704918	-	0	ID=cds725;	0	16	STM14_0749	ybeA	SPOUT methyltransferase superfamily protein	2,273602649	-2,403729808
NC_01685(	RefSeq	CDS	704922	705239	-	0	ID=cds726;	0	15,5	STM14_0750	ybeB	hypothetical protein	2,273602649	-2,403729808
NC_01685(	RefSeq	CDS	705568	706176	-	0	ID=cds727;	0	6	STM14_0751	cobC	alpha ribazole-5%27-P phosphatase	0,852600993	-1,98056697
NC_01685(	RefSeq	CDS	706273	707367	+	0	ID=cds728;	0	16,5	STM14_0752	cobD	threonine-phosphate decarboxylase	2,273602649	-2,403729808
NC_01685(	RefSeq	CDS	707342	707983	-	0	ID=cds729;	0	10	STM14_0753	nadD	nicotinic acid mononucleotide adenylyltransferase	1,421001655	-2,256999172
NC_01685(	RefSeq	CDS	707985	709016	-	0	ID=cds730;	0	29	STM14_0754	holA	DNA polymerase III subunit delta	4,120904801	-2,455089955
NC_01685(	RefSeq	CDS	709016	709606	-	0	ID=cds731;	0	24,5	STM14_0755	rlpB	LPS-assembly lipoprotein RplB	3,410403973	-2,434086198
NC_01685(	RefSeq	CDS	709621	712203	-	0	ID=cds732;	0	120,5	STM14_0756	STM14_0756	hypothetical protein	17,05201986	-1,853016647
NC_01685(	RefSeq	CDS	712491	712781	+	0	ID=cds733;	0	14,83333333	STM14_0757	STM14_0757	putative hydrolase	2,131502483	-2,392032191
NC_01685(	RefSeq	CDS	712798	713970	+	0	ID=cds734;	0	17,5	STM14_0758	STM14_0758	putative hydrolase	2,55780298	-2,419166771
NC_01685(	RefSeq	CDS	714020	714973	+	0	ID=cds735;	0	9,5	STM14_0759	STM14_0759	2-keto-3-deoxygluconate permease	1,421001655	-2,256999172
NC_01685(	RefSeq	CDS	715041	716969	+	0	ID=cds736;	0	23	STM14_0760	STM14_0760	putative sigma-54 dependent transcriptional regulator	3,268303807	-2,432747446
NC_01685(	RefSeq	CDS	717062	717535	+	0	ID=cds737;	0	22,5	STM14_0761	ybeL	hypothetical protein	3,126203642	-2,431590384
NC_01685(	RefSeq	CDS	717574	718569	-	0	ID=cds738;	0	1,5	STM14_0762	ybeQ	hypothetical protein	0,284200331	-1,182723423
NC_01685(	RefSeq	CDS	718694	719401	+	0	ID=cds739;	0	0	STM14_0763	ybeR	putative cytoplasmic protein	0	NA
NC_01685(	RefSeq	CDS	719398	720831	+	0	ID=cds740;	0	0	STM14_0764	ybeS	putative molecular chaperone	0	NA
NC_01685(	RefSeq	region	720843	721539	+	.	ID=id22;Nc	0	1	-	-	-	0,142100166	-0,623264422
NC_01685(	RefSeq	CDS	721536	723008	+	0	ID=cds741;	0	8	STM14_0766	ybeV	putative molecular chaperone	1,136801324	-2,147403465
NC_01685(	RefSeq	CDS	723030	724709	-	0	ID=cds742;	0	7	STM14_0767	hscC	putative heatshock protein	0,994701159	-2,072978628
NC_01685(	RefSeq	CDS	724785	726023	-	0	ID=cds743;	0	15,5	STM14_0768	STM14_0768	putative cytoplasmic protein	2,273602649	-2,403729808
NC_01685(	RefSeq	CDS	726055	726990	-	0	ID=cds744;	0	8,5	STM14_0769	rihA	ribonucleoside hydrolase 1	1,136801324	-2,147403465
NC_01685(	RefSeq	CDS	727107	727832	-	0	ID=cds745;	0	1	STM14_0770	gltL	glutamate%2Faspartate transporter	0,142100166	-0,623264422
NC_01685(	RefSeq	CDS	727832	728506	-	0	ID=cds746;	0	1	STM14_0771	gltK	glutamate%2Faspartate transporter	0,142100166	-0,623264422
NC_01685(	RefSeq	CDS	728506	729246	-	0	ID=cds747;	0	0	STM14_0772	gltJ	glutamate%2Faspartate transporter	0	NA
NC_01685(	RefSeq	CDS	729406	730332	-	0	ID=cds748;	0	35,5	STM14_0773	gltI	glutamate and aspartate transporter subunit	5,115605959	-2,281753063
NC_01685(	RefSeq	CDS	730309	730596	+	0	ID=cds749;	0	0	STM14_0774	STM14_0774	hypothetical protein	0	NA
NC_01685(	RefSeq	CDS	730669	732207	-	0	ID=cds750;	0	38	STM14_0775	lnt	apolipoprotein N-acyltransferase	5,39980629	-2,264331336
NC_01685(	RefSeq	CDS	732227	733105	-	0	ID=cds751;	0	38	STM14_0776	ybeX	putative transport protein	5,39980629	-2,264331336
NC_01685(	RefSeq	CDS	733102	733251	-	0	ID=cds752;	0	4,5	STM14_0777	STM14_0777	hypothetical protein	0,568400662	-1,710438693
NC_01685(	RefSeq	CDS	733263	733736	-	0	ID=cds753;	0	7	STM14_0778	ybeY	putative metalloprotease	0,994701159	-2,072978628
NC_01685(	RefSeq	CDS	733733	734818	-	0	ID=cds754;	0	23,5	STM14_0779	phoL	putative phosphate starvation-inducible protein	3,410403973	-2,434086198
NC_01685(	RefSeq	CDS	734838	734957	-	0	ID=cds755;	0	2	STM14_0780	STM14_0780	hypothetical protein	0,284200331	-1,182723423
NC_01685(	RefSeq	CDS	734984	736408	-	0	ID=cds756;	0	27,33333333	STM14_0781	miaB	rRNA modification protein	3,836704469	-2,44262576
NC_01685(	RefSeq	CDS	736552	737727	+	0	ID=cds757;	0	13	STM14_0782	ubiF	2-octaprenyl-3-methyl-6-methoxy-1%2C4-benzoquinol hy	1,847302152	-2,356210635
NC_01685(	RefSeq	CDS	737794	738369	-	0	ID=cds758;	0	1	STM14_0783	STM14_0783	putative inner membrane protein	0,142100166	-0,623264422
NC_01685(	RefSeq	CDS	739310	739477	+	0	ID=cds759;	0	0	STM14_0791	STM14_0791	hypothetical protein	0	NA
NC_01685(	RefSeq	CDS	739436	741100	-	0	ID=cds760;	0	11,5	STM14_0792	asnB	asparagine synthetase B	1,705201986	-2,329925387
NC_01685(	RefSeq	CDS	741390	742142	-	0	ID=cds761;	2	14,5	STM14_0793	nagD	UMP phosphatase	5,508047057	0,527345695
NC_01685(	RefSeq	CDS	742189	743409	-	0	ID=cds762;	0	24,83333333	STM14_0794	nagC	N-acetylglucosamine operon transcriptional repressor	3,552504138	-2,43600272

NC_01685(	RefSeq	CDS	743414	744568	-	0	ID=cds763;	0	22,5	STM14_0795	nagA	N-acetylglucosamine-6-phosphate deacetylase	3,126203642	-2,431590384
NC_01685(	RefSeq	CDS	744628	745428	-	0	ID=cds764;	3	3,5	STM14_0796	nagB	glucosamine-6-phosphate deaminase	5,846367772	1,996213194
NC_01685(	RefSeq	CDS	745470	745595	-	0	ID=cds765;	0	0	STM14_0797	STM14_0797	hypothetical protein	0	NA
NC_01685(	RefSeq	CDS	745755	747707	+	0	ID=cds766;	0	34	STM14_0798	nagE	N-acetyl glucosamine specific PTS system components IIA	4,831405628	-2,523251848
NC_01685(	RefSeq	CDS	747918	749585	+	0	ID=cds767;	0	90,5	STM14_0799	glnS	glutaminyl-tRNA synthetase	12,7890149	-1,945533971
NC_01685(	RefSeq	CDS	749621	749734	+	0	ID=cds768;	0	0	STM14_0800	STM14_0800	hypothetical protein	0	NA
NC_01685(	RefSeq	CDS	749864	750154	+	0	ID=cds769;	0	2,5	STM14_0801	STM14_0801	hypothetical protein	0,284200331	-1,182723423
NC_01685(	RefSeq	CDS	750031	751437	+	0	ID=cds770;	0	8,5	STM14_0802	ybfM	putative outer membrane protein	1,136801324	-2,147403465
NC_01685(	RefSeq	CDS	751487	751819	+	0	ID=cds771;	0	1,5	STM14_0803	ybfN	putative lipoprotein	0,284200331	-1,182723423
NC_01685(	RefSeq	CDS	751868	753172	-	0	ID=cds772;	0	2	STM14_0804	citA	citrate-proton symporter	0,284200331	-1,182723423
NC_01685(	RefSeq	CDS	753223	754362	-	0	ID=cds773;	0	1	STM14_0805	citB	citrate utilization protein b	0,142100166	-0,623264422
NC_01685(	RefSeq	CDS	754349	755752	-	0	ID=cds774;	0	0	STM14_0806	STM14_0806	tricarballylate dehydrogenase	0	NA
NC_01685(	RefSeq	CDS	755850	756776	-	0	ID=cds775;	0	5,5	STM14_0807	STM14_0807	putative transcriptional regulator	0,852600993	-1,98056697
NC_01685(	RefSeq	CDS	756891	757343	-	0	ID=cds776;	0	23,5	STM14_0808	fur	ferric uptake regulator	3,410403973	-2,434086198
NC_01685(	RefSeq	CDS	757442	757591	+	0	ID=cds777;	0	0	STM14_0809	STM14_0809	hypothetical protein	0	NA
NC_01685(	RefSeq	CDS	757625	758254	-	0	ID=cds778;	5	23	STM14_0810	fldA	flavodoxin FldA	12,06491566	0,972680854
NC_01685(	RefSeq	CDS	758318	758599	-	0	ID=cds779;	0,5	5	STM14_0811	ybfE	LexA regulated protein	0,710500828	-1,864806942
NC_01685(	RefSeq	CDS	758607	758720	+	0	ID=cds780;	0	0	STM14_0812	STM14_0812	hypothetical protein	0	NA
NC_01685(	RefSeq	CDS	758733	759503	-	0	ID=cds781;	0	1	STM14_0813	ybfF	hypothetical protein	0,142100166	-0,623264422
NC_01685(	RefSeq	CDS	759655	760230	+	0	ID=cds782;	0	58,75	STM14_0814	seqA	replication initiation regulator SeqA	8,383909767	-2,110782333
NC_01685(	RefSeq	CDS	760255	761895	+	0	ID=cds783;	0	121,25	STM14_0815	pgm	phosphoglucomutase	17,19412003	-1,853939051
NC_01685(	RefSeq	CDS	762010	762495	-	0	ID=cds784;	0	332,5	STM14_0816	STM14_0816	putative cytoplasmic protein	47,17725496	-1,93235414
NC_01685(	RefSeq	CDS	762560	763879	-	0	ID=cds785;	0	1144,166667	STM14_0817	potE	putrescine transporter	162,5625894	-1,970015346
NC_01685(	RefSeq	CDS	763876	766074	-	0	ID=cds786;	0	2450,833333	STM14_0818	speF	ornithine decarboxylase	348,2875057	-1,978802999
NC_01685(	RefSeq	CDS	766193	766318	-	0	ID=cds787;	0	310,8333333	STM14_0819	STM14_0819	hypothetical protein	44,19315148	-1,928973636
NC_01685(	RefSeq	CDS	766472	766585	+	0	ID=cds788;	0	0	STM14_0820	STM14_0820	hypothetical protein	0	NA
NC_01685(	RefSeq	CDS	766836	767513	-	0	ID=cds789;	0	5,5	STM14_0821	kdpE	DNA-binding transcriptional activator KdpE	0,852600993	-1,98056697
NC_01685(	RefSeq	CDS	767510	770194	-	0	ID=cds790;	0	9,5	STM14_0822	kdpD	sensor protein KdpD	1,421001655	-2,256999172
NC_01685(	RefSeq	CDS	770191	770775	-	0	ID=cds791;	0	0,5	STM14_0823	kdpC	potassium-transporting ATPase subunit C	0	NA
NC_01685(	RefSeq	CDS	770784	772832	-	0	ID=cds792;	0	0,5	STM14_0824	kdpB	potassium-transporting ATPase subunit B	0	NA
NC_01685(	RefSeq	CDS	772853	774532	-	0	ID=cds793;	0	0	STM14_0825	kdpA	potassium-transporting ATPase subunit A	0	NA
NC_01685(	RefSeq	CDS	774532	774729	-	0	ID=cds794;	0	0	STM14_0826	STM14_0826	K%2B-transporting ATPase subunit F	0	NA
NC_01685(	RefSeq	CDS	774958	775164	+	0	ID=cds795;	2	12	STM14_0827	ybfA	hypothetical protein	5,223846726	0,666065355
NC_01685(	RefSeq	CDS	775275	776696	+	0	ID=cds796;	0	0	STM14_0828	phrB	deoxyribodipyrimidine photolyase	0	NA
NC_01685(	RefSeq	CDS	776735	778216	-	0	ID=cds797;	0	3,5	STM14_0829	ybgH	POT family transport protein	0,568400662	-1,710438693
NC_01685(	RefSeq	CDS	778545	779288	+	0	ID=cds798;	0	31,5	STM14_0830	ybgI	putative hydrolase-oxidase	4,547205297	-2,489878815
NC_01685(	RefSeq	CDS	779304	779960	+	0	ID=cds799;	0	8,25	STM14_0831	ybgJ	putative carboxylase	1,136801324	-2,147403465
NC_01685(	RefSeq	CDS	779954	780886	+	0	ID=cds800;	0	10,75	STM14_0832	ybgK	putative carboxylase	1,563101821	-2,297202414
NC_01685(	RefSeq	CDS	780876	781610	+	0	ID=cds801;	0	3	STM14_0833	ybgL	hypothetical protein	0,426300497	-1,498211557
NC_01685(	RefSeq	CDS	781934	782065	+	0	ID=cds802;	0	0	STM14_0834	STM14_0834	putative cytoplasmic protein	0	NA
NC_01685(	RefSeq	CDS	782125	782691	+	0	ID=cds803;	0	3	STM14_0835	STM14_0835	putative phage integrase	0,426300497	-1,498211557
NC_01685(	RefSeq	CDS	783151	783486	+	0	ID=cds804;	0	1	STM14_0836	STM14_0836	putative inner membrane protein	0,142100166	-0,623264422
NC_01685(	RefSeq	CDS	783488	784228	+	0	ID=cds805;	0	1	STM14_0837	STM14_0837	putative cytoplasmic protein	0,142100166	-0,623264422
NC_01685(	RefSeq	CDS	784514	785665	+	0	ID=cds806;	0	0	STM14_0838	STM14_0838	putative UDP-galactopyranose mutase	0	NA
NC_01685(	RefSeq	CDS	785662	786558	+	0	ID=cds807;	0	1	STM14_0839	STM14_0839	putative glycosyl transferase	0,142100166	-0,623264422
NC_01685(	RefSeq	CDS	786571	787704	+	0	ID=cds808;	0	1,5	STM14_0840	STM14_0840	putative glycosyl transferase	0,284200331	-1,182723423
NC_01685(	RefSeq	CDS	787833	788603	+	0	ID=cds809;	0	1	STM14_0841	STM14_0841	ABC transporter permease	0,142100166	-0,623264422
NC_01685(	RefSeq	CDS	788607	789317	+	0	ID=cds810;	0	0	STM14_0842	STM14_0842	putative ABC-type polysaccharide%2Fpolyol phosphate tra	0	NA
NC_01685(	RefSeq	CDS	789411	791189	+	0	ID=cds811;	0	0	STM14_0843	STM14_0843	putative glycosyltransferase	0	NA
NC_01685(	RefSeq	CDS	791382	792215	+	0	ID=cds812;	0	0	STM14_0844	STM14_0844	putative glycosyltransferase	0	NA
NC_01685(	RefSeq	CDS	792273	794168	+	0	ID=cds813;	0	0	STM14_0845	STM14_0845	putative glycosyl transferase	0	NA
NC_01685(	RefSeq	CDS	794236	794373	+	0	ID=cds814;	0	6,5	STM14_0846	STM14_0846	putative cytoplasmic protein	0,852600993	-1,98056697
NC_01685(	RefSeq	CDS	794422	795213	+	0	ID=cds815;	0	11,5	STM14_0847	nei	endonuclease VIII	1,705201986	-2,329925387
NC_01685(	RefSeq	CDS	795268	796314	-	0	ID=cds816;	1	0	STM14_0848	abrB	putative transport protein	1,75932237	1,868512432
NC_01685(	RefSeq	CDS	796451	797734	-	0	ID=cds817;	0	106,5	STM14_0849	gltA	type II citrate synthase	15,06261755	-1,883514636
NC_01685(	RefSeq	CDS	797694	798080	+	0	ID=cds818;	0	0	STM14_0850	STM14_0850	putative inner membrane protein	0	NA
NC_01685(	RefSeq	CDS	798489	798878	+	0	ID=cds819;	0	20	STM14_0851	sdhC	succinate dehydrogenase cytochrome b556 large membra	2,842003311	-2,427810709
NC_01685(	RefSeq	CDS	798872	799219	+	0	ID=cds820;	0	18,25	STM14_0852	sdhD	succinate dehydrogenase cytochrome b556 small membra	2,55780298	-2,419166771
NC_01685(	RefSeq	CDS	799219	800985	+	0	ID=cds821;	4	59,75	STM14_0853	sdhA	succinate dehydrogenase flavoprotein subunit	15,56329941	-0,192302343
NC_01685(	RefSeq	CDS	800999	801718	+	0	ID=cds822;	0	44	STM14_0854	sdhB	succinate dehydrogenase iron-sulfur subunit	6,252407284	-2,216733793
NC_01685(	RefSeq	CDS	802242	805043	+	0	ID=cds823;	0	342,5	STM14_0855	kgd	alpha-ketoglutarate decarboxylase	48,59825661	-1,933828099
NC_01685(	RefSeq	CDS	805058	806266	+	0	ID=cds824;	0	282	STM14_0856	sucB	dihydrolipoamide acetyltransferase	40,07224668	-1,923545725
NC_01685(	RefSeq	CDS	806408	807574	+	0	ID=cds825;	1	215	STM14_0857	sucC	succinyl-CoA synthetase subunit beta	32,31085796	-2,615488743
NC_01685(	RefSeq	CDS	807574	808443	+	0	ID=cds826;	0	119,25	STM14_0858	sucD	succinyl-CoA synthetase subunit alpha	16,9099197	-1,852081147
NC_01685(	RefSeq	CDS	808472	808597	+	0	ID=cds827;	0	2,25	STM14_0859	STM14_0859	hypothetical protein	0,284200331	-1,182723423
NC_01685(	RefSeq	CDS	809819	809962	-	0	ID=cds828;	0	0	STM14_0860	STM14_0860	hypothetical protein	0	NA
NC_01685(	RefSeq	CDS	810026	811594	+	0	ID=cds829;	0	540	STM14_0861	cydA	cytochrome d terminal oxidase polypeptide subunit I	76,73408939	-1,952286828
NC_01685(	RefSeq	CDS	811610	812749	+	0	ID=cds830;	0	396	STM14_0862	cydB	cytochrome d terminal oxidase polypeptide subunit II	56,27166555	-1,940579381
NC_01685(	RefSeq	CDS	812764	812877	+	0	ID=cds831;	0	46,5	STM14_0863	ybgT	putative outer membrane lipoprotein	6,536607615	-2,201017198
NC_01685(	RefSeq	CDS	812877	813017	+	0	ID=cds832;	0	32	STM14_0864	STM14_0864	hypothetical protein	4,547205297	-2,489878815
NC_01685(	RefSeq	CDS	813014	813295	+	0	ID=cds833;	0	59,5	STM14_0865	ybgE	hypothetical protein	8,526009932	-2,104284262
NC_01685(	RefSeq	CDS	813524	813928	+	0	ID=cds834;	0	9,5	STM14_0866	ybgC	acyl-CoA thioester hydrolase YbgC	1,421001655	-2,256999172
NC_01685(	RefSeq	CDS	813925	814617	+	0	ID=cds835;	0	12	STM14_0867	tolQ	colicin uptake protein TolQ	1,705201986	-2,329925387
NC_01685(	RefSeq	CDS	814621	815049	+	0	ID=cds836;	0	18,5	STM14_0868	tolR	colicin uptake protein TolR	2,55780298	-2,419166771
NC_01685(	RefSeq	CDS	815114	816337	+	0	ID=cds837;	0	11,5	STM14_0869	tolA	cell envelope integrity inner membrane protein TolA	1,705201986	-2,329925387
NC_01685(	RefSeq	CDS	816461	817753	+	0	ID=cds838;	0	46,5	STM14_0870	tolB	translocation protein TolB	6,536607615	-2,201017198
NC_01685(	RefSeq	CDS	817788	818312	+	0	ID=cds839;	0	77,5	STM14_0871	pal	peptidoglycan-associated outer membrane lipoprotein	11,08381291	-2,003216272
NC_01685(	RefSeq	CDS	818322	819110	+	0	ID=cds840;	0	30,75	STM14_0872	ybgF	hypothetical protein	4,405105132	-2,475774913
NC_01685(	RefSeq	CDS	818715	819254	-	0	ID=cds841;	0	0,5	STM14_0873	STM14_0873	hypothetical protein	0	NA
NC_01685(	RefSeq	CDS	820227	820322	-	0	ID=cds842;	0	0	STM14_0879	STM14_0879	hypothetical protein	0	NA
NC_01685(	RefSeq	CDS	820575	821618	+	0	ID=cds843;	0	0,5	STM14_0880	nadA	quinolinate synthetase	0	NA
NC_01685(	RefSeq	CDS	821643	822362	+	0	ID=cds844;	0	2,5	STM14_0881	pnuC	nucleoside%2Fpurine%2Fpyrimidine transporter	0,284200331	-1,182723423
NC_01685(	RefSeq	CDS	822359	823297	-	0	ID=cds845;	0	0	STM14_0882	ybgR	zinc transporter ZitB	0	NA
NC_01685(	RefSeq	CDS	823408	823794	-	0	ID=cds846;	0	0	STM14_0883	ybgS	hypothetical protein	0	NA
NC_01685(	RefSeq	CDS	824114	825166	+	0	ID=cds847;	0	39	STM14_0884	aroG	phospho-2-dehydro-3-deoxyheptonate aldolase	5,541906456	-2,25579729
NC_01685(	RefSeq	CDS	825260	825805	-	0	ID=cds848;	0	5	STM14_0885	STM14_0885	fumarate hydratase	0,710500828	-1,864806942
NC_01685(	RefSeq	CDS	825820	826665	-	0	ID=cds849;	0	1	STM14_0886	STM14_0886	fumarate hydratase	0,142100166	-0,623264422
NC_01685(	RefSeq	CDS	826795	827685	+	0	ID=cds850;	0	6,5	STM14_0887	STM14_0887	transcriptional regulator	0,852600993	-1,98056697
NC_01685(	RefSeq	CDS	827686	828669	-	0	ID=cds851;	0	4	STM14_0888	STM14_0888	transcriptional regulator	0,568400662	-1,710438693
NC_01685(	RefSeq	CDS	828913	830160	+	0	ID=cds852;	0	0	STM14_0889	STM14_0889	putative cation transporter	0	NA
NC_01685(	RefSeq	CDS	830210	830455	+	0	ID=cds853;	0	0	STM14_0890	dcoC	oxaloacetate decarboxylase subunit gamma	0	NA
NC_01685(	RefSeq	CDS	832259	833560	+	0	ID=cds854;	0	0,5	STM14_0892	dcoB	oxaloacetate decarboxylase subunit beta	0	NA
NC_01685(	RefSeq	CDS	833679	834377	+	0	ID=cds855;	0	2	STM14_0893	STM14_0893	putative cytoplasmic protein	0,284200331	-1,182723423
NC_01685(	RefSeq	CDS	834757	835836	+	0	ID=cds856;	0	0	STM14_0894	STM14_0894	putative ABC transport protein	0	NA
NC_01685(	RefSeq	CDS	835836	836612	+	0	ID=cds857;	0	1	STM14_0895	STM14_0895	putative ABC-type cobalamin%2FFe3%2B-siderophore tra	0,142100166	-0,623264422
NC_01685(	RefSeq	CDS	836703	837455	-	0	ID=cds858;	0,5	42	STM14_0896	gpmA	phosphoglyceromutase	5,968206953	-2,232890319
NC_01685(	RefSeq	CDS	837473	837664	-	0	ID=cds859;	1,5	5	STM14_0897	STM14_0897	hypothetical protein	4,229145568	1,438201827
NC_01685(	RefSeq	CDS	837679	838719	-	0	ID=cds860;	0	33	STM14_0898	galM	aldose 1-epimerase	4,689305463	-2,505858154
NC_01685(	RefSeq	CDS	838713	839861	-	0	ID=cds861;	0	9,5	STM14_0899	galK	galactokinase	1,421001655	-2,256999172
NC_01685(	RefSeq	CDS	839864	840910	-	0	ID=cds862;	0	15,5	STM14_0900	galT	galactose-1-phosphate uridylyltransferase	2,273602649	-2,403729808
NC_01685(	RefSeq	CDS	840921	841937	-	0	ID=cds863;	0	9	STM14_0901	galE	UDP-galactose-4-epimerase	1,27890149	-2,207741391
NC_01685(	RefSeq	CDS	842160	843068	-	0	ID=cds864;	0	0	STM14_0902	STM14_0902	putative inner membrane protein	0	NA
NC_01685(	RefSeq	CDS	843123	843230	+	0	ID=cds865;	0	0	STM14_0903	STM14_0903	hypothetical protein	0	NA
NC_01685(	RefSeq	CDS	843239	844714	-	0	ID=cds866;	0	13,5	STM14_0904	modF	putative molybdenum transport ATP-binding protein Moc	1,989402318	-2,376535557
NC_01685(	RefSeq	CDS	844782	845570	-	0	ID=cds867;	0	4	STM14_0905	modE	DNA-binding transcriptional regulator ModE	0,568400662	-1,710438693
NC_01685(	RefSeq	CDS	845597	845848	+	0	ID=cds868;	1	15,5	STM14_0906	STM14_0906	hypothetical protein	4,032925019	-0,231745061
NC_01685(	RefSeq	CDS	846015	846788	+	0	ID=cds869;	0	12	STM14_0907	modA	molybdate transporter periplasmic protein	1,705201986	-2,329925387
NC_01685(	RefSeq	CDS	846788	847477	+	0	ID=cds870;	0	10	STM14_0908	modB	molybdate ABC transporter permease	1,421001655	-2,256999172
NC_01685(	RefSeq	CDS	847480	848538	+	0	ID=cds871;	0	14,5	STM14_0909	modC	molybdate transporter ATP-binding protein	1,989402318	-2,376535557
NC_01685(	RefSeq	CDS	848539	849357	-	0	ID=cds872;	0	5,5	STM14_0910	ybhA	phosphotransferase	0,852600993	-1,98056697
NC_01685(	RefSeq	CDS	849521	850516	+	0	ID=cds873;	0	18	STM14_0911	ybhE	6-phosphogluconolactonase	2,55780298	-2,419166771
NC_01685(	RefSeq	CDS	850660	851943	-	0	ID=cds874;	0	19	STM14_0912	ybhC	putative pectinesterase	2,699903145	-2,42419778
NC_01685(	RefSeq	CDS	852181	853404	+	0	ID=cds875;	0	7,5	STM14_0913	hutI	imidazolonepropionase	1,136801324	-2,147403465
NC_01685(	RefSeq	CDS	853401	854342	+	0	ID=cds876;	0	2,5	STM14_0914	hutG	formimidoylglutamase	0,284200331	-1,182723423
NC_01685(	RefSeq	CDS	854387	855112	+	0	ID=cds877;	0	9,5	STM14_0915	hutC	histidine utilization repressor	1,421001655	-2,256999172
NC_01685(	RefSeq	CDS	856996	858516	+	0	ID=cds878;	0	9,5	STM14_0917	hutH	histidine ammonia-lyase	1,421001655	-2,256999172
NC_01685(	RefSeq	CDS	858601	859077	-	0	ID=cds879;	0	3	STM14_0918	ybhB	putative kinase inhibitor protein	0,426300497	-1,498211557
NC_01685(	RefSeq	CDS	859135	860424	-	0	ID=cds880;	0	1	STM14_0919	bioA	adenosylmethionine-8-amino-7-oxononanoate aminotran	0,142100166	-0,623264422
NC_01685(	RefSeq	CDS	860511	861551	+	0	ID=cds881;	0	0	STM14_0920	bioB	biotin synthetase	0	NA
NC_01685(	RefSeq	CDS	861548	862705	+	0	ID=cds882;	0	0	STM14_0921	bioF	8-amino-7-oxononanoate synthase	0	NA
NC_01685(	RefSeq	CDS	862689	863444	+	0	ID=cds883;	0	0	STM14_0922	bioC	biotin biosynthesis protein BioC	0	NA
NC_01685(	RefSeq	CDS	863437	864123	+	0	ID=cds884;	0	0	STM14_0923	bioD_1	dithiobiotin synthetase	0	NA
NC_01685(	RefSeq	CDS	864209	864493	+	0	ID=cds885;	0	0	STM14_0924	STM14_0924	hypothetical protein	0	NA
NC_01685(	RefSeq	CDS	864550	864777	+	0	ID=cds886;	0	0,75	STM14_0925	STM14_0925	hypothetical protein	0,142100166	-0,623264422

NC_01685(	RefSeq	CDS	864774	866795	+	0	ID=cds887;	1	133,75	STM14_0926	uvrB	excinuclease ABC subunit B	20,80074455	-2,22102224
NC_01685(	RefSeq	CDS	867104	867277	+	0	ID=cds888;	0	1,25	STM14_927	STM14_927	putative cytoplasmic protein	0,142100166	-0,623264422
NC_01685(	RefSeq	CDS	867285	869582	+	0	ID=cds889;	0	37,75	STM14_928	slrP	hypothetical protein	5,39980629	-2,264331336
NC_01685(	RefSeq	CDS	869674	870582	-	0	ID=cds890;	0	9	STM14_929	ybhK	putative cytoplasmic protein	1,27890149	-2,207741391
NC_01685(	RefSeq	CDS	870979	871968	+	0	ID=cds891;	0,5	25,5	STM14_930	moaA	molybdenum cofactor biosynthesis protein A	3,694604304	-2,438835558
NC_01685(	RefSeq	CDS	871990	872502	+	0	ID=cds892;	0,5	9,25	STM14_931	moaB	molybdopterin biosynthetic protein B	1,27890149	-2,207741391
NC_01685(	RefSeq	CDS	872505	872990	+	0	ID=cds893;	0	4,25	STM14_932	moaC	molybdenum cofactor biosynthesis protein C	0,568400662	-1,710438693
NC_01685(	RefSeq	CDS	872983	873228	+	0	ID=cds894;	0	6	STM14_933	moaD	molybdopterin synthase small subunit	0,852600993	-1,98056697
NC_01685(	RefSeq	CDS	873230	873682	+	0	ID=cds895;	0	6	STM14_934	moaE	molybdopterin synthase large subunit	0,852600993	-1,98056697
NC_01685(	RefSeq	CDS	873730	874434	+	0	ID=cds896;	0	37	STM14_935	ybhL	putative permease	5,257706125	-2,272984196
NC_01685(	RefSeq	CDS	874479	874667	+	0	ID=cds897;	0	1,1	STM14_936	STM14_936	hypothetical protein	0,142100166	-0,623264422
NC_01685(	RefSeq	CDS	874624	875082	+	0	ID=cds898;	0	2,9	STM14_937	tnpA_2	transposase	0,426300497	-1,498211557
NC_01685(	RefSeq	CDS	875292	875999	+	0	ID=cds899;	0	0	STM14_938	ybhM	putative integral membrane protein	0	NA
NC_01685(	RefSeq	CDS	876023	876631	+	0	ID=cds900;	0	0	STM14_939	STM14_939	putative inner membrane protein	0	NA
NC_01685(	RefSeq	CDS	876639	877199	+	0	ID=cds901;	0	0	STM14_940	STM14_940	putative inner membrane protein	0	NA
NC_01685(	RefSeq	CDS	877202	878164	-	0	ID=cds902;	0	3	STM14_941	ybhN	hypothetical protein	0,426300497	-1,498211557
NC_01685(	RefSeq	CDS	878164	879405	-	0	ID=cds903;	0	0	STM14_942	ybhO	cardiolipin synthase 2	0	NA
NC_01685(	RefSeq	CDS	879402	880160	-	0	ID=cds904;	0	0	STM14_943	ybhP	putative cytoplasmic protein	0	NA
NC_01685(	RefSeq	CDS	880293	880703	+	0	ID=cds905;	0	52	STM14_944	ybhQ	putative inner membrane protein	7,389208608	-2,158807911
NC_01685(	RefSeq	CDS	880665	881771	-	0	ID=cds906;	0	13,75	STM14_945	ybhR	putative transport protein	1,989402318	-2,376535557
NC_01685(	RefSeq	CDS	881747	881884	-	0	ID=cds907;	0	6,25	STM14_946	STM14_946	hypothetical protein	0,852600993	-1,98056697
NC_01685(	RefSeq	CDS	881887	883017	-	0	ID=cds908;	0	19,5	STM14_947	ybhS	putative transport protein	2,842003311	-2,427810709
NC_01685(	RefSeq	CDS	883010	884746	-	0	ID=cds909;	2	17,5	STM14_948	ybhF	putative ABC-type multidrug transport system ATPase cor	6,07644772	0,297448745
NC_01685(	RefSeq	CDS	884739	885734	-	0	ID=cds910;	0	10,5	STM14_949	STM14_949	hypothetical protein	1,421001655	-2,256999172
NC_01685(	RefSeq	CDS	885734	886408	-	0	ID=cds911;	0	6,5	STM14_950	ybiH	putative DNA-binding transcriptional regulator	0,852600993	-1,98056697
NC_01685(	RefSeq	CDS	886638	888002	+	0	ID=cds912;	0	8,75	STM14_951	rhlE	ATP-dependent RNA helicase RhlE	1,27890149	-2,207741391
NC_01685(	RefSeq	CDS	888006	888110	-	0	ID=cds913;	0	0	STM14_952	STM14_952	hypothetical protein	0	NA
NC_01685(	RefSeq	CDS	888210	890354	+	0	ID=cds914;	0	39	STM14_953	dinG	ATP-dependent DNA helicase DinG	5,541906456	-2,25579729
NC_01685(	RefSeq	CDS	890384	891358	+	0	ID=cds915;	0	13	STM14_954	ybiB	hypothetical protein	1,847302152	-2,356210635
NC_01685(	RefSeq	CDS	891514	891774	-	0	ID=cds916;	0	3	STM14_955	ybiJ	hypothetical protein	0,426300497	-1,498211557
NC_01685(	RefSeq	CDS	891948	892067	+	0	ID=cds917;	0	0,5	STM14_956	STM14_956	hypothetical protein	0	NA
NC_01685(	RefSeq	CDS	892059	892325	-	0	ID=cds918;	0	1,5	STM14_957	ybiI	hypothetical protein	0,284200331	-1,182723423
NC_01685(	RefSeq	CDS	892325	892444	-	0	ID=cds919;	0	1,5	STM14_958	STM14_958	hypothetical protein	0,284200331	-1,182723423
NC_01685(	RefSeq	CDS	892530	893456	+	0	ID=cds920;	0	3	STM14_959	ybiN	putative SAM-dependent methyltransferase	0,426300497	-1,498211557
NC_01685(	RefSeq	CDS	893453	895675	-	0	ID=cds921;	0	2	STM14_960	ybiO	hypothetical protein	0,284200331	-1,182723423
NC_01685(	RefSeq	CDS	895674	895811	+	0	ID=cds922;	0	1	STM14_961	STM14_961	hypothetical protein	0,142100166	-0,623264422
NC_01685(	RefSeq	CDS	895794	896516	-	0	ID=cds923;	0	6	STM14_962	glnQ	glutamine ABC transporter ATP-binding protein	0,852600993	-1,98056697
NC_01685(	RefSeq	CDS	896513	897172	-	0	ID=cds924;	0	6,5	STM14_963	glnP	glutamine ABC transporter permease	0,852600993	-1,98056697
NC_01685(	RefSeq	CDS	897316	898062	-	0	ID=cds925;	0,5	16	STM14_964	glnH	glutamine ABC transporter periplasmic protein	2,273602649	-2,403729808
NC_01685(	RefSeq	CDS	898059	898181	-	0	ID=cds926;	0,5	0	STM14_965	STM14_965	hypothetical protein	0	NA
NC_01685(	RefSeq	CDS	898539	899042	-	0	ID=cds927;	0	25	STM14_966	dps	DNA starvation%2Fstationary phase protection protein Dp	3,552504138	-2,43600272
NC_01685(	RefSeq	CDS	899158	899316	-	0	ID=cds928;	0	0	STM14_967	STM14_967	hypothetical protein	0	NA
NC_01685(	RefSeq	CDS	899345	900232	-	0	ID=cds929;	0	4	STM14_968	ybiF	threonine and homoserine efflux system	0,568400662	-1,710438693
NC_01685(	RefSeq	CDS	900254	900415	-	0	ID=cds930;	0	0	STM14_969	STM14_969	hypothetical protein	0	NA
NC_01685(	RefSeq	CDS	900375	900506	-	0	ID=cds931;	0	0	STM14_970	STM14_970	hypothetical protein	0	NA
NC_01685(	RefSeq	CDS	900586	901101	+	0	ID=cds932;	0	68	STM14_971	ompX	outer membrane protein X	9,662811256	-2,058244678
NC_01685(	RefSeq	CDS	901164	902744	-	0	ID=cds933;	0	5	STM14_972	ybiP	putative integral membrane protein	0,710500828	-1,864806942
NC_01685(	RefSeq	CDS	902910	903080	+	0	ID=cds934;	0	0	STM14_973	STM14_973	hypothetical protein	0	NA
NC_01685(	RefSeq	CDS	903009	903137	-	0	ID=cds935;	5	10,5	STM14_974	STM14_974	hypothetical protein	10,21761351	1,731360567
NC_01685(	RefSeq	CDS	903323	903796	+	0	ID=cds936;	0	6	STM14_975	STM14_975	manganese transport regulator MntR	0,852600993	-1,98056697
NC_01685(	RefSeq	CDS	903793	904905	+	0	ID=cds937;	0	7,5	STM14_976	ybiR	putative transporter	1,136801324	-2,147403465
NC_01685(	RefSeq	CDS	904949	905869	-	0	ID=cds938;	0	11	STM14_977	ybiS	hypothetical protein	1,563101821	-2,297202414
NC_01685(	RefSeq	CDS	906088	907680	+	0	ID=cds939;	0	35,5	STM14_978	ybiT	putative ABC transporter ATPase component	5,115605959	-2,281753063
NC_01685(	RefSeq	CDS	908136	909032	+	0	ID=cds940;	0	2	STM14_979	STM14_979	putative inner membrane protein	0,284200331	-1,182723423
NC_01685(	RefSeq	CDS	909061	909780	-	0	ID=cds941;	0	0	STM14_980	ybiV%282%29	putative hydrolase	0	NA
NC_01685(	RefSeq	CDS	909985	911250	-	0	ID=cds942;	0	2	STM14_981	ybiU	putative cytoplasmic protein	0,284200331	-1,182723423
NC_01685(	RefSeq	CDS	911396	911509	+	0	ID=cds943;	0	0	STM14_982	STM14_982	hypothetical protein	0	NA
NC_01685(	RefSeq	CDS	911512	912321	+	0	ID=cds944;	0	4	STM14_983	ybiV%281%29	putative hydrolase	0,568400662	-1,710438693
NC_01685(	RefSeq	CDS	912399	914831	-	0	ID=cds945;	0	4	STM14_984	pflF	putative pyruvate formate lyase	0,568400662	-1,710438693
NC_01685(	RefSeq	CDS	914837	915736	-	0	ID=cds946;	0	2,333333333	STM14_985	pflE	putative pyruvate formate lyase activating enzyme	0,284200331	-1,182723423
NC_01685(	RefSeq	CDS	915878	916033	-	0	ID=cds947;	0	0	STM14_986	STM14_986	hypothetical protein	0	NA
NC_01685(	RefSeq	CDS	916014	916763	-	0	ID=cds948;	0	6	STM14_987	moeB	molybdopterin biosynthesis protein MoeB	0,852600993	-1,98056697
NC_01685(	RefSeq	CDS	916763	918004	-	0	ID=cds949;	0	11,5	STM14_988	moeA	molybdopterin biosynthesis protein MoeA	1,705201986	-2,329925387
NC_01685(	RefSeq	CDS	917997	918137	+	0	ID=cds950;	0	0	STM14_989	STM14_989	hypothetical protein	0	NA
NC_01685(	RefSeq	CDS	918201	919142	+	0	ID=cds951;	0	6	STM14_990	ybiK	L-asparaginase	0,852600993	-1,98056697
NC_01685(	RefSeq	CDS	919153	921024	+	0	ID=cds952;	1	12,5	STM14_991	yliA	glutathione transporter ATP-binding protein	3,464524356	0,028939145
NC_01685(	RefSeq	CDS	921057	922595	+	0	ID=cds953;	0	10	STM14_992	yliB	putative ABC transporter periplasmic binding protein	1,421001655	-2,256999172
NC_01685(	RefSeq	CDS	922656	923576	+	0	ID=cds954;	2	4	STM14_993	yliC	putative ABC transporter periplasmic binding protein	4,087045402	1,631390028
NC_01685(	RefSeq	CDS	923579	924490	+	0	ID=cds955;	0	6	STM14_994	yliD	putative ABC transporter inner membrane component	0,852600993	-1,98056697
NC_01685(	RefSeq	CDS	924480	924599	-	0	ID=cds956;	0	0	STM14_995	STM14_995	hypothetical protein	0	NA
NC_01685(	RefSeq	CDS	924581	925906	-	0	ID=cds957;	0	7	STM14_996	yliG	putative FeS oxidoreductase	0,994701159	-2,072978628
NC_01685(	RefSeq	CDS	925933	926067	+	0	ID=cds958;	0	2	STM14_997	STM14_997	hypothetical protein	0,284200331	-1,182723423
NC_01685(	RefSeq	CDS	926137	926520	+	0	ID=cds959;	0	11,5	STM14_998	bssR	biofilm formation regulatory protein BssR	1,705201986	-2,329925387
NC_01685(	RefSeq	CDS	926622	926762	+	0	ID=cds960;	0	0	STM14_999	STM14_999	hypothetical protein	0	NA
NC_01685(	RefSeq	CDS	926970	927134	+	0	ID=cds961;	0	0	STM14_1000	STM14_1000	hypothetical protein	0	NA
NC_01685(	RefSeq	CDS	927233	927748	+	0	ID=cds962;	0	0,5	STM14_1001	STM14_1001	putative cytoplasmic protein	0	NA
NC_01685(	RefSeq	CDS	927666	928520	+	0	ID=cds963;	0	0,5	STM14_1002	STM14_1002	putative electron transfer protein beta subunit	0	NA
NC_01685(	RefSeq	CDS	928531	929478	+	0	ID=cds964;	0	0	STM14_1003	STM14_1003	putative electron transfer protein alpha subunit	0	NA
NC_01685(	RefSeq	CDS	929772	930935	+	0	ID=cds965;	0	0	STM14_1005	STM14_1005	putative acyl-CoA dehydrogenase	0	NA
NC_01685(	RefSeq	CDS	931103	931243	-	0	ID=cds966;	0	0	STM14_1006	STM14_1006	hypothetical protein	0	NA
NC_01685(	RefSeq	CDS	931384	933069	+	0	ID=cds967;	0	3,5	STM14_1007	STM14_1007	putative dehydrogenase	0,568400662	-1,710438693
NC_01685(	RefSeq	CDS	933075	933965	-	0	ID=cds968;	0	10	STM14_1008	STM14_1008	putative transcriptional regulator	1,421001655	-2,256999172
NC_01685(	RefSeq	CDS	934230	934655	-	0	ID=cds969;	0	2,333333333	STM14_1009	STM14_1009	putative inner membrane protein	0,284200331	-1,182723423
NC_01685(	RefSeq	CDS	934780	934917	+	0	ID=cds970;	0	0	STM14_1010	STM14_1010	hypothetical protein	0	NA
NC_01685(	RefSeq	CDS	935002	935682	+	0	ID=cds971;	0	0	STM14_1011	STM14_1011	hypothetical protein	0	NA
NC_01685(	RefSeq	CDS	935679	936305	-	0	ID=cds972;	0	11	STM14_1012	yliJ	putative glutathione S-transferase	1,563101821	-2,297202414
NC_01685(	RefSeq	CDS	936549	937751	+	0	ID=cds973;	1	10,5	STM14_1013	dacC	D-alanyl-D-alanine carboxypeptidase	3,180324025	0,201203536
NC_01685(	RefSeq	CDS	937796	938554	-	0	ID=cds974;	0	30	STM14_1014	deoR	DNA-binding transcriptional repressor DeoR	4,263004966	-2,464060063
NC_01685(	RefSeq	CDS	938626	939234	-	0	ID=cds975;	0	32	STM14_1015	ybjG	undecaprenyl pyrophosphate phosphatase	4,547205297	-2,489878815
NC_01685(	RefSeq	CDS	939547	940779	+	0	ID=cds976;	2	4,5	STM14_1016	mdfA	multidrug translocase	4,087045402	1,631390028
NC_01685(	RefSeq	CDS	940833	941648	-	0	ID=cds977;	0	6	STM14_1017	STM14_1017	putative hydrolase	0,852600993	-1,98056697
NC_01685(	RefSeq	CDS	941645	942856	-	0	ID=cds978;	0	11,5	STM14_1018	STM14_1018	putative transport protein%2Fregulator	1,705201986	-2,329925387
NC_01685(	RefSeq	CDS	942902	943027	+	0	ID=cds979;	0	0	STM14_1019	STM14_1019	hypothetical protein	0	NA
NC_01685(	RefSeq	CDS	943024	943644	+	0	ID=cds980;	0	0	STM14_1020	STM14_1020	putative regulatory protein	0	NA
NC_01685(	RefSeq	CDS	943761	945446	-	0	ID=cds981;	0	27,5	STM14_1021	STM14_1021	hypothetical protein	3,978804635	-2,448045729
NC_01685(	RefSeq	CDS	945717	946094	+	0	ID=cds982;	0	17	STM14_1022	ybjM	putative inner membrane protein	2,415702814	-2,412509737
NC_01685(	RefSeq	CDS	946126	946389	-	0	ID=cds983;	0	43,5	STM14_1023	grxA	glutaredoxin	6,252407284	-2,216733793
NC_01685(	RefSeq	CDS	946559	946849	+	0	ID=cds984;	0	2,25	STM14_1024	ybjC	hypothetical protein	0,284200331	-1,182723423
NC_01685(	RefSeq	CDS	946833	947555	+	0	ID=cds985;	0	7,75	STM14_1025	mdaA	nitroreductase A	1,136801324	-2,147403465
NC_01685(	RefSeq	CDS	947613	948515	+	0	ID=cds986;	0	8	STM14_1026	rimK	ribosomal protein S6 modification protein	1,136801324	-2,147403465
NC_01685(	RefSeq	CDS	948612	949088	+	0	ID=cds987;	0	5	STM14_1027	ybjN	putative cytoplasmic protein	0,710500828	-1,864806942
NC_01685(	RefSeq	CDS	949437	950549	+	0	ID=cds988;	0	1	STM14_1028	potF	putrescine ABC transporter periplasmic substrate-binding	0,142100166	-0,623264422
NC_01685(	RefSeq	CDS	950637	951770	+	0	ID=cds989;	0	1	STM14_1029	potG	putrescine ABC transporter ATP-binding protein	0,142100166	-0,623264422
NC_01685(	RefSeq	CDS	951780	952733	+	0	ID=cds990;	0	0,5	STM14_1030	potH	putrescine ABC transporter membrane protein	0	NA
NC_01685(	RefSeq	CDS	952730	953575	+	0	ID=cds991;	0	3	STM14_1031	potI	putrescine ABC transporter membrane protein	0,426300497	-1,498211557
NC_01685(	RefSeq	CDS	953649	954122	+	0	ID=cds992;	0	3	STM14_1032	ybjO	putative inner membrane protein	0,426300497	-1,498211557
NC_01685(	RefSeq	CDS	954165	955295	+	0	ID=cds993;	0	5	STM14_1033	rumB	23S rRNA methyluridine methyltransferase	0,710500828	-1,864806942
NC_01685(	RefSeq	CDS	955378	955569	+	0	ID=cds994;	0	0	STM14_1034	STM14_1034	hypothetical protein	0	NA
NC_01685(	RefSeq	CDS	955578	956921	+	0	ID=cds995;	0	0,5	STM14_1035	ulaA_1	PTS system ascorbate-specific transporter subunit IIC	0	NA
NC_01685(	RefSeq	CDS	956951	957271	+	0	ID=cds996;	0	1	STM14_1036	STM14_1036	putative inner membrane protein	0,142100166	-0,623264422
NC_01685(	RefSeq	CDS	957281	958768	+	0	ID=cds997;	0	1	STM14_1037	STM14_1037	putative sulfatase	0,142100166	-0,623264422
NC_01685(	RefSeq	CDS	958765	958965	-	0	ID=cds998;	0	0,5	STM14_1038	STM14_1038	hypothetical protein	0	NA
NC_01685(	RefSeq	CDS	958987	959718	-	0	ID=cds999;	0	4	STM14_1039	artJ	arginine transport system component	0,568400662	-1,710438693
NC_01685(	RefSeq	CDS	959955	960623	-	0	ID=cds100(	0	10,5	STM14_1040	artM	arginine transporter permease subunit ArtM	1,421001655	-2,256999172
NC_01685(	RefSeq	CDS	960623	961339	-	0	ID=cds100:	0	16	STM14_1041	artQ	arginine transporter permease subunit ArtQ	2,273602649	-2,403729808
NC_01685(	RefSeq	CDS	961346	962077	-	0	ID=cds100:	0	12	STM14_1042	artI	arginine transport system	1,705201986	-2,329925387
NC_01685(	RefSeq	CDS	962095	962823	-	0	ID=cds100:	0	13	STM14_1043	artP	arginine transporter ATP-binding subunit	1,847302152	-2,356210635
NC_01685(	RefSeq	CDS	963053	963568	-	0	ID=cds100	0	8,5	STM14_1044	ybjP	putative lipoprotein	1,136801324	-2,147403465
NC_01685(	RefSeq	CDS	963696	964019	+	0	ID=cds100!	0	13	STM14_1045	orfB	hypothetical protein	1,847302152	-2,356210635
NC_01685(	RefSeq	CDS	964016	964846	+	0	ID=cds100(	2	4	STM14_1046	ybjR	putative aminidase	4,087045402	1,631390028
NC_01685(	RefSeq	CDS	964843	965856	-	0	ID=cds100	0	19,5	STM14_1047	STM14_1047	putative nucleoside-diphosphate-sugar epimerase	2,842003311	-2,427810709
NC_01685(	RefSeq	CDS	965952	967385	-	0	ID=cds100!	0	3,5	STM14_1048	ybjT	putative nucleoside-diphosphate-sugar epimerase	0,568400662	-1,710438693
NC_01685(	RefSeq	CDS	967396	968397	-	0	ID=cds100!	0	10,5	STM14_1049	ltaA	L-threonine aldolase	1,421001655	-2,256999172
NC_01685(	RefSeq	CDS	968436	970154	-	0	ID=cds101(	0	2,5	STM14_1050	poxB	pyruvate dehydrogenase	0,284200331	-1,182723423

NC_016856	RefSeq	CDS	970312	971283	-	0	ID=cds1011	0	1	STM14_1051	hcr	HCP oxidoreductase	0,142100166	-0,623264422
NC_016856	RefSeq	CDS	971292	972944	-	0	ID=cds1012	0	6	STM14_1052	hcp	hydroxylamine reductase	0,852600993	-1,98056697
NC_016856	RefSeq	CDS	972908	973111	+	0	ID=cds1013	0	0	STM14_1053	STM14_1053	hypothetical protein	0	NA
NC_016856	RefSeq	CDS	973088	973987	-	0	ID=cds1014	0	8,5	STM14_1054	ybjE	putative inner membrane protein	1,136801324	-2,147403465
NC_016856	RefSeq	CDS	973986	974153	+	0	ID=cds1015	0	0,5	STM14_1055	STM14_1055	hypothetical protein	0	NA
NC_016856	RefSeq	CDS	974189	975847	+	0	ID=cds1016	0	9,5	STM14_1056	ybjD	hypothetical protein	1,421001655	-2,256999172
NC_016856	RefSeq	CDS	975844	976812	-	0	ID=cds1017	7	87	STM14_1057	ybjX	VirK-like protein	24,67797099	-0,003889347
NC_016856	RefSeq	CDS	976940	978058	+	0	ID=cds1018	0	13,5	STM14_1058	ybjY	macrolide transporter subunit MacA	1,989402318	-2,376535557
NC_016856	RefSeq	CDS	978055	980001	+	0	ID=cds1019	0	19,5	STM14_1059	ybjZ	macrolide transporter ATP-binding %2Fpermease	2,842003311	-2,427810709
NC_016856	RefSeq	CDS	980013	980138	-	0	ID=cds1020	0	0,333333333	STM14_1060	STM14_1060	hypothetical protein	0	NA
NC_016856	RefSeq	CDS	980033	980176	+	0	ID=cds1021	0	1,333333333	STM14_1061	STM14_1061	hypothetical protein	0,142100166	-0,623264422
NC_016856	RefSeq	CDS	980131	980352	-	0	ID=cds1022	0	15,33333333	STM14_1062	cspD	stress response protein	2,131502483	-2,392032191
NC_016856	RefSeq	CDS	980676	980996	+	0	ID=cds1023	0	33,5	STM14_1063	clpS	ATP-dependent Clp protease adaptor protein ClpS	4,831405628	-2,523251848
NC_016856	RefSeq	CDS	981027	983303	+	0	ID=cds1024	0	129	STM14_1064	clpA	ATP-dependent Clp protease ATP-binding subunit	18,33092135	-1,860878454
NC_016856	RefSeq	CDS	983350	983538	+	0	ID=cds1025	0	1,6	STM14_1065	STM14_1065	hypothetical protein	0,284200331	-1,182723423
NC_016856	RefSeq	CDS	983495	983953	+	0	ID=cds1026	0	2,9	STM14_1066	tnpA_3	transposase	0,426300497	-1,498211557
NC_016856	RefSeq	CDS	984062	984343	-	0	ID=cds1027	0	0,5	STM14_1067	STM14_1067	hypothetical protein	0	NA
NC_016856	RefSeq	CDS	984416	985099	-	0	ID=cds1028	0	0	STM14_1068	STM14_1068	putative integrase	0	NA
NC_016856	RefSeq	CDS	985102	985260	-	0	ID=cds1029	0	2	STM14_1069	STM14_1069	hypothetical protein	0,284200331	-1,182723423
NC_016856	RefSeq	CDS	985270	985671	-	0	ID=cds1030	0	1	STM14_1070	STM14_1070	putative cytoplasmic protein	0,142100166	-0,623264422
NC_016856	RefSeq	CDS	986008	986685	-	0	ID=cds1031	0	3,5	STM14_1072	STM14_1072	SlsA	0,568400662	-1,710438693
NC_016856	RefSeq	CDS	986705	987565	-	0	ID=cds1032	0	7,333333333	STM14_1073	STM14_1073	putative cytoplasmic protein	0,994701159	-2,072978628
NC_016856	RefSeq	CDS	987674	988585	+	0	ID=cds1033	0	2	STM14_1074	STM14_1074	putative transcriptional regulator	0,284200331	-1,182723423
NC_016856	RefSeq	CDS	988676	988894	-	0	ID=cds1034	2	36,75	STM14_1075	infA	translation initiation factor IF-1	8,776350865	-0,381024791
NC_016856	RefSeq	CDS	988822	989205	-	0	ID=cds1035	1	22,25	STM14_1076	STM14_1076	putative inner membrane protein	4,885526012	-0,515045314
NC_016856	RefSeq	CDS	989206	989910	-	0	ID=cds1036	0	10,5	STM14_1077	aat	leucyl%2Fphenylalanyl-tRNA--protein transferase	1,421001655	-2,256999172
NC_016856	RefSeq	CDS	989955	991676	-	0	ID=cds1037	0	22,5	STM14_1078	cydC	cysteine%2Fglutathione ABC transporter membrane%2FAT	3,126203642	-2,431590384
NC_016856	RefSeq	CDS	991677	993443	-	0	ID=cds1038	0	25,5	STM14_1079	cydD	cysteine%2Fglutathione ABC transporter membrane%2FAT	3,694604304	-2,438835558
NC_016856	RefSeq	CDS	993557	994525	-	0	ID=cds1039	0	35,83333333	STM14_1080	trxB	thioredoxin reductase	5,115605959	-2,281753063
NC_016856	RefSeq	CDS	994689	994946	-	0	ID=cds1040	0	2	STM14_1081	STM14_1081	hypothetical protein	0,284200331	-1,182723423
NC_016856	RefSeq	CDS	994867	995142	-	0	ID=cds1041	0	0	STM14_1082	STM14_1082	hypothetical protein	0	NA
NC_016856	RefSeq	CDS	995071	995565	+	0	ID=cds1042	0	50,25	STM14_1083	lrp	leucine-responsive transcriptional regulator	7,105008277	-2,173389122
NC_016856	RefSeq	CDS	995700	999785	+	0	ID=cds1043	0	128	STM14_1084	ftsK	DNA translocase FtsK	18,18882119	-1,860051483
NC_016856	RefSeq	CDS	999925	1000539	+	0	ID=cds1044	0	58,25	STM14_1085	lolA	outer-membrane lipoprotein carrier protein	8,241809601	-2,117369507
NC_016856	RefSeq	CDS	1000549	1001892	+	0	ID=cds1045	1,5	31,75	STM14_1086	ycaJ	recombination factor protein RarA	8,065850037	-0,242041354
NC_016856	RefSeq	CDS	1001941	1002111	+	0	ID=cds1046	0	24,5	STM14_1087	STM14_1087	hypothetical protein	3,410403973	-2,434086198
NC_016856	RefSeq	CDS	1002151	1003443	+	0	ID=cds1047	0	164	STM14_1088	serS	seryl-tRNA synthetase	23,30442715	-1,884236327
NC_016856	RefSeq	CDS	1003680	1006124	+	0	ID=cds1048	1,5	352	STM14_1089	dmsA	anaerobic dimethyl sulfoxide reductase subunit A	53,53790301	-2,539486341
NC_016856	RefSeq	CDS	1006135	1006752	+	0	ID=cds1049	0	58	STM14_1090	dmsB	anaerobic dimethyl sulfoxide reductase subunit B	8,241809601	-2,117369507
NC_016856	RefSeq	CDS	1006754	1007617	+	0	ID=cds1050	0	89,5	STM14_1091	dmsC	anaerobic dimethyl sulfoxide reductase subunit C	12,7890149	-1,945533971
NC_016856	RefSeq	CDS	1007647	1007754	+	0	ID=cds1051	0	19,5	STM14_1092	STM14_1092	hypothetical protein	2,842003311	-2,427810709
NC_016856	RefSeq	CDS	1007837	1007968	-	0	ID=cds1052	0	0	STM14_1093	STM14_1093	hypothetical protein	0	NA
NC_016856	RefSeq	CDS	1007967	1009115	+	0	ID=cds1053	0	19	STM14_1094	ycaD	MFS family transporter	2,699903145	-2,42419778
NC_016856	RefSeq	CDS	1009333	1010754	+	0	ID=cds1054	0	8	STM14_1095	ycaM	putative amino-acid transporter	1,136801324	-2,147403465
NC_016856	RefSeq	CDS	1011047	1011844	-	0	ID=cds1055	0	35	STM14_1096	pflA	pyruvate formate lyase-activating enzyme 1	4,973505794	-2,290637485
NC_016856	RefSeq	CDS	1011963	1012289	-	0	ID=cds1056	3	10,5	STM14_1097	STM14_1097	putative cytoplasmic protein	6,698968765	1,219842116
NC_016856	RefSeq	CDS	1012420	1013379	+	0	ID=cds1057	1	13,5	STM14_1098	STM14_1098	SopD-like protein	3,748724687	-0,112256387
NC_016856	RefSeq	CDS	1013455	1015737	-	0	ID=cds1058	1	823,1666667	STM14_1099	pflB	pyruvate formate lyase I	118,7077586	-3,747156433
NC_016856	RefSeq	CDS	1015797	1016612	-	0	ID=cds1059	0	64	STM14_1100	focA	formate transporter	9,094410594	-2,079502768
NC_016856	RefSeq	CDS	1017059	1018819	-	0	ID=cds1060	2,5	17,5	STM14_1101	ycaO	putative cytoplasmic protein	6,07644772	0,297448745
NC_016856	RefSeq	CDS	1018831	1018959	+	0	ID=cds1061	0	0	STM14_1102	STM14_1102	hypothetical protein	0	NA
NC_016856	RefSeq	CDS	1018956	1019648	+	0	ID=cds1062	0	0	STM14_1103	ycaP	putative inner membrane protein	0	NA
NC_016856	RefSeq	CDS	1019641	1019754	+	0	ID=cds1063	0	0	STM14_1104	STM14_1104	hypothetical protein	0	NA
NC_016856	RefSeq	CDS	1019834	1020922	+	0	ID=cds1064	0	20,5	STM14_1105	serC	phosphoserine aminotransferase	2,842003311	-2,427810709
NC_016856	RefSeq	CDS	1020993	1022276	+	0	ID=cds1065	0	7,5	STM14_1106	aroA	3-phosphoshikimate 1-carboxyvinyltransferase	1,136801324	-2,147403465
NC_016856	RefSeq	CDS	1022419	1023180	+	0	ID=cds1066	0	2,5	STM14_1107	ycaL	putative Zn-dependent protease	0,284200331	-1,182723423
NC_016856	RefSeq	CDS	1023127	1023381	-	0	ID=cds1067	0	0	STM14_1108	STM14_1108	hypothetical protein	0	NA
NC_016856	RefSeq	CDS	1023353	1024036	+	0	ID=cds1068	0	53	STM14_1109	cmk	cytidylate kinase	7,531308773	-2,151665531
NC_016856	RefSeq	CDS	1024150	1025823	+	0	ID=cds1069	0	815,6666667	STM14_1110	rpsA	30S ribosomal protein S1	115,9537351	-1,963539109
NC_016856	RefSeq	CDS	1025979	1026263	+	0	ID=cds1070	0	17	STM14_1111	ihfB	integration host factor subunit beta	2,415702814	-2,412509737
NC_016856	RefSeq	CDS	1026487	1028757	+	0	ID=cds1071	0	3,5	STM14_1112	ycaI	hypothetical protein	0,568400662	-1,710438693
NC_016856	RefSeq	CDS	1028794	1030542	+	0	ID=cds1072	0	35,5	STM14_1113	msbA	lipid transporter ATP-binding%2Fpermease	5,115605959	-2,281753063
NC_016856	RefSeq	CDS	1030539	1031516	+	0	ID=cds1073	0	12,5	STM14_1114	lpxK	tetraacyldisaccharide 4%27-kinase	1,705201986	-2,329925387
NC_016856	RefSeq	CDS	1031560	1032792	+	0	ID=cds1074	0	9	STM14_1115	ycaQ	putative cytoplasmic protein	1,27890149	-2,207741391
NC_016856	RefSeq	CDS	1032844	1033026	+	0	ID=cds1075	0	17,25	STM14_1116	ycaR	hypothetical protein	2,415702814	-2,412509737
NC_016856	RefSeq	CDS	1033023	1033769	+	0	ID=cds1076	0	24,25	STM14_1117	kdsB	3-deoxy-manno-octulosonate cytidylyltransferase	3,410403973	-2,434086198
NC_016856	RefSeq	CDS	1033792	1033944	-	0	ID=cds1077	0	0	STM14_1118	STM14_1118	hypothetical protein	0	NA
NC_016856	RefSeq	CDS	1033980	1034873	+	0	ID=cds1078	0	28,5	STM14_1119	STM14_1119	hypothetical protein	3,978804635	-2,448045729
NC_016856	RefSeq	CDS	1034853	1035629	-	0	ID=cds1079	0	0	STM14_1120	ycbC	hypothetical protein	0	NA
NC_016856	RefSeq	CDS	1035765	1036568	+	0	ID=cds1080	0	14	STM14_1121	smtA	putative metallothionein SmtA	1,989402318	-2,376535557
NC_016856	RefSeq	CDS	1036561	1037883	+	0	ID=cds1081	0	13	STM14_1122	mukF	condesin subunit F	1,847302152	-2,356210635
NC_016856	RefSeq	CDS	1037864	1038568	+	0	ID=cds1082	0	11,5	STM14_1123	mukE	condesin subunit E	1,705201986	-2,329925387
NC_016856	RefSeq	CDS	1038568	1043034	+	0	ID=cds1083	0	119,8333333	STM14_1124	mukB	cell division protein MukB	17,05201986	-1,853016647
NC_016856	RefSeq	CDS	1043379	1045220	+	0	ID=cds1084	0	21,5	STM14_1125	ycbB	hypothetical protein	3,126203642	-2,431590384
NC_016856	RefSeq	CDS	1045480	1046028	+	0	ID=cds1085	0	91	STM14_1126	ycbK	putative outer membrane protein	12,93111506	-1,941086641
NC_016856	RefSeq	CDS	1046056	1046703	+	0	ID=cds1086	0	75	STM14_1127	ycbL	putative metallo-beta-lactamase	10,65751242	-2,019002164
NC_016856	RefSeq	CDS	1046765	1047955	-	0	ID=cds1087	0	61,5	STM14_1128	aspC	aromatic amino acid aminotransferase	8,810210263	-2,091539774
NC_016856	RefSeq	CDS	1047995	1048126	-	0	ID=cds1088	0	0	STM14_1129	STM14_1129	hypothetical protein	0	NA
NC_016856	RefSeq	CDS	1048140	1049231	-	0	ID=cds1089	0	2,5	STM14_1130	ompF	outer membrane protein F	0,284200331	-1,182723423
NC_016856	RefSeq	CDS	1049521	1049673	+	0	ID=cds1090	0	1	STM14_1131	STM14_1131	hypothetical protein	0,142100166	-0,623264422
NC_016856	RefSeq	CDS	1049838	1051238	-	0	ID=cds1091	0	103	STM14_1132	asnS	asparaginyl-tRNA synthetase	14,63631705	-1,894252559
NC_016856	RefSeq	CDS	1051307	1051426	-	0	ID=cds1092	0	0,5	STM14_1133	STM14_1133	hypothetical protein	0	NA
NC_016856	RefSeq	CDS	1051439	1051900	-	0	ID=cds1093	0	4	STM14_1134	STM14_1134	putative leucine response regulator	0,568400662	-1,710438693
NC_016856	RefSeq	CDS	1051897	1052118	-	0	ID=cds1094	0	1,5	STM14_1135	STM14_1135	hypothetical protein	0,284200331	-1,182723423
NC_016856	RefSeq	CDS	1052217	1053431	+	0	ID=cds1095	0	0	STM14_1136	STM14_1136	diaminopropionate ammonia-lyase	0	NA
NC_016856	RefSeq	CDS	1053504	1053662	+	0	ID=cds1096	0	0	STM14_1137	STM14_1137	hypothetical protein	0	NA
NC_016856	RefSeq	CDS	1053676	1055112	+	0	ID=cds1097	0	1	STM14_1138	STM14_1138	putative transcriptional regulator	0,142100166	-0,623264422
NC_016856	RefSeq	CDS	1055190	1056392	-	0	ID=cds1098	0	8	STM14_1139	pncB	nicotinate phosphoribosyltransferase	1,136801324	-2,147403465
NC_016856	RefSeq	CDS	1056587	1057879	-	0	ID=cds1099	0	21,5	STM14_1140	STM14_1140	integrase	3,126203642	-2,431590384
NC_016856	RefSeq	CDS	1057924	1058172	-	0	ID=cds1100	0	0,5	STM14_1141	STM14_1141	excisionase	0	NA
NC_016856	RefSeq	CDS	1058213	1058452	-	0	ID=cds1101	0	0,5	STM14_1142	STM14_1142	hypothetical protein	0	NA
NC_016856	RefSeq	CDS	1058495	1059604	-	0	ID=cds1102	0	1,5	STM14_1143	STM14_1143	hypothetical protein	0,284200331	-1,182723423
NC_016856	RefSeq	CDS	1059615	1062500	-	0	ID=cds1103	0	0,5	STM14_1144	STM14_1144	exodeoxyribonuclease	0	NA
NC_016856	RefSeq	CDS	1062627	1062926	-	0	ID=cds1104	0	0	STM14_1145	STM14_1145	hypothetical protein	0	NA
NC_016856	RefSeq	CDS	1062948	1063106	-	0	ID=cds1105	0	0	STM14_1146	STM14_1146	hypothetical protein	0	NA
NC_016856	RefSeq	CDS	1063099	1063359	-	0	ID=cds1106	0	0	STM14_1147	STM14_1147	hypothetical protein	0	NA
NC_016856	RefSeq	CDS	1063409	1063819	-	0	ID=cds1107	5	13,5	STM14_1148	STM14_1148	regulatory protein	10,78601417	1,432042884
NC_016856	RefSeq	CDS	1063939	1064178	+	0	ID=cds1108	0	0	STM14_1149	STM14_1149	hypothetical protein	0	NA
NC_016856	RefSeq	CDS	1064144	1064518	+	0	ID=cds1109	0	1	STM14_1150	STM14_1150	regulatory protein	0,142100166	-0,623264422
NC_016856	RefSeq	CDS	1064603	1065586	+	0	ID=cds1110	0	0	STM14_1151	STM14_1151	regulatory protein	0	NA
NC_016856	RefSeq	CDS	1065589	1066338	+	0	ID=cds1111	0	0	STM14_1152	STM14_1152	putative replication protein	0	NA
NC_016856	RefSeq	CDS	1066349	1066696	+	0	ID=cds1112	0	0	STM14_1153	STM14_1153	hypothetical protein	0	NA
NC_016856	RefSeq	CDS	1066693	1067004	+	0	ID=cds1113	0	0	STM14_1154	STM14_1154	hypothetical protein	0	NA
NC_016856	RefSeq	CDS	1067082	1067372	+	0	ID=cds1114	0	13	STM14_1155	STM14_1155	hypothetical protein	1,847302152	-2,356210635
NC_016856	RefSeq	CDS	1067664	1067897	+	0	ID=cds1115	0	2,5	STM14_1156	STM14_1156	hypothetical protein	0,284200331	-1,182723423
NC_016856	RefSeq	CDS	1068009	1068230	+	0	ID=cds1116	0	0	STM14_1157	STM14_1157	hypothetical protein	0	NA
NC_016856	RefSeq	CDS	1068313	1068915	+	0	ID=cds1117	0	1	STM14_1158	STM14_1158	hypothetical protein	0,142100166	-0,623264422
NC_016856	RefSeq	CDS	1068921	1069121	+	0	ID=cds1118	0	0	STM14_1159	STM14_1159	hypothetical protein	0	NA
NC_016856	RefSeq	CDS	1069124	1069735	+	0	ID=cds1119	0	0,5	STM14_1160	STM14_1160	hypothetical protein	0	NA
NC_016856	RefSeq	CDS	1069732	1069878	+	0	ID=cds1120	0	0	STM14_1161	STM14_1161	hypothetical protein	0	NA
NC_016856	RefSeq	CDS	1069868	1070665	+	0	ID=cds1121	0	0	STM14_1162	STM14_1162	putative molecular chaperone	0	NA
NC_016856	RefSeq	CDS	1070732	1071049	+	0	ID=cds1122	0	3,5	STM14_1163	STM14_1163	hypothetical protein	0,568400662	-1,710438693
NC_016856	RefSeq	CDS	1071223	1071348	+	0	ID=cds1123	0	0	STM14_1164	STM14_1164	hypothetical protein	0	NA
NC_016856	RefSeq	CDS	1071484	1071933	-	0	ID=cds1124	0	3	STM14_1165	STM14_1165	hypothetical protein	0,426300497	-1,498211557
NC_016856	RefSeq	CDS	1072294	1072980	-	0	ID=cds1125	0	1	STM14_1166	gtgA	bacteriophage virulence determinant	0,142100166	-0,623264422
NC_016856	RefSeq	CDS	1073250	1073585	+	0	ID=cds1126	0	0	STM14_1167	STM14_1167	hypothetical protein	0	NA
NC_016856	RefSeq	CDS	1073569	1074021	+	0	ID=cds1127	0	0	STM14_1168	STM14_1168	lysozyme	0	NA
NC_016856	RefSeq	CDS	1074039	1074518	+	0	ID=cds1128	0	1	STM14_1169	STM14_1169	hypothetical protein	0,142100166	-0,623264422
NC_016856	RefSeq	CDS	1074726	1075259	+	0	ID=cds1129	0	0	STM14_1170	STM14_1170	hypothetical protein	0	NA
NC_016856	RefSeq	CDS	1075216	1077354	+	0	ID=cds1130	0	3	STM14_1171	STM14_1171	hypothetical protein	0,426300497	-1,498211557
NC_016856	RefSeq	CDS	1077554	1079101	+	0	ID=cds1131	0	2	STM14_1172	STM14_1172	phage portal protein%2C lambda family	0,284200331	-1,182723423
NC_016856	RefSeq	CDS	1079016	1081106	+	0	ID=cds1132	0	1	STM14_1173	STM14_1173	Clp protease-like protein	0,142100166	-0,623264422
NC_016856	RefSeq	CDS	1081197	1081520	+	0	ID=cds1133	0	0	STM14_1174	STM14_1174	putative RecA%2FRadA recombinase	0	NA
NC_016856	RefSeq	CDS	1081513	1081812	+	0	ID=cds1134	0	0	STM14_1175	STM14_1175	ATP-binding sugar transporter-like protein	0	NA

NC_016856	RefSeq	CDS	1081793	1082359	+	0	ID=cds1135	0	0	STM14_1176	STM14_1176	minor tail protein	0	NA
NC_016856	RefSeq	CDS	1082356	1082757	+	0	ID=cds1136	0	0	STM14_1177	STM14_1177	minor tail protein	0	NA
NC_016856	RefSeq	CDS	1082769	1083518	+	0	ID=cds1137	0	1,5	STM14_1178	STM14_1178	major tail protein	0,284200331	-1,182723423
NC_016856	RefSeq	CDS	1083564	1083962	+	0	ID=cds1138	0	0	STM14_1179	STM14_1179	minor tail protein	0	NA
NC_016856	RefSeq	CDS	1083959	1084288	+	0	ID=cds1139	0	1	STM14_1180	STM14_1180	minor tail protein	0,142100166	-0,623264422
NC_016856	RefSeq	CDS	1084368	1087355	+	0	ID=cds1140	0	4	STM14_1181	STM14_1181	minor tail protein	0,568400662	-1,710438693
NC_016856	RefSeq	CDS	1087352	1087684	+	0	ID=cds1141	0	0	STM14_1182	STM14_1182	minor tail protein	0	NA
NC_016856	RefSeq	CDS	1087783	1088280	+	0	ID=cds1142	0	4	STM14_1183	STM14_1183	attachment%2Finvasion protein	0,568400662	-1,710438693
NC_016856	RefSeq	CDS	1088397	1088930	-	0	ID=cds1143	0	37,5	STM14_1184	sodC_1	superoxide dismutase precursor	5,39980629	-2,264331336
NC_016856	RefSeq	CDS	1089020	1089715	+	0	ID=cds1144	0	3	STM14_1185	STM14_1185	minor tail protein	0,426300497	-1,498211557
NC_016856	RefSeq	CDS	1089725	1090462	+	0	ID=cds1145	0	1	STM14_1186	STM14_1186	tail assembly protein	0,142100166	-0,623264422
NC_016856	RefSeq	CDS	1090360	1091064	+	0	ID=cds1146	0	2	STM14_1187	STM14_1187	tail assembly protein	0,284200331	-1,182723423
NC_016856	RefSeq	CDS	1091136	1094486	+	0	ID=cds1147	0	4	STM14_1188	STM14_1188	host specificity protein J	0,568400662	-1,710438693
NC_016856	RefSeq	CDS	1094525	1094767	+	0	ID=cds1148	0	0	STM14_1189	STM14_1189	hypothetical protein	0	NA
NC_016856	RefSeq	CDS	1094821	1097259	+	0	ID=cds1149	0	1	STM14_1190	STM14_1190	tail fiber protein	0,142100166	-0,623264422
NC_016856	RefSeq	CDS	1097259	1097840	+	0	ID=cds1150	0	0	STM14_1191	STM14_1191	tail fiber assembly like-protein	0	NA
NC_016856	RefSeq	CDS	1097844	1097951	-	0	ID=cds1151	0	0	STM14_1192	STM14_1192	tail fiber assembly protein	0	NA
NC_016856	RefSeq	CDS	1098316	1099284	+	0	ID=cds1152	0	3,5	STM14_1193	sseI	secreted effector protein	0,568400662	-1,710438693
NC_016856	RefSeq	CDS	1099932	1100558	-	0	ID=cds1153	0	0	STM14_1195	STM14_1195	hypothetical protein	0	NA
NC_016856	RefSeq	CDS	1100627	1100926	-	0	ID=cds1154	0	0,833333333	STM14_1196	STM14_1196	hypothetical protein	0,142100166	-0,623264422
NC_016856	RefSeq	CDS	1100911	1101597	-	0	ID=cds1155	0	1,5	STM14_1197	STM14_1197	hypothetical protein	0,284200331	-1,182723423
NC_016856	RefSeq	CDS	1101868	1102059	-	0	ID=cds1156	0	0	STM14_1198	STM14_1198	MsgA-like protein	0	NA
NC_016856	RefSeq	CDS	1102486	1105098	+	0	ID=cds1157	0	33	STM14_1199	pepN	aminopeptidase N	4,689305463	-2,505858154
NC_016856	RefSeq	CDS	1105306	1106316	+	0	ID=cds1158	5	9	STM14_1200	pyrD	dihydroorotate dehydrogenase 2	10,07551334	1,822921095
NC_016856	RefSeq	CDS	1106320	1106445	-	0	ID=cds1159	0	0	STM14_1201	STM14_1201	hypothetical protein	0	NA
NC_016856	RefSeq	CDS	1106482	1107024	+	0	ID=cds1160	0	15	STM14_1202	ycbW	putative cytoplasmic protein	2,131502483	-2,392032191
NC_016856	RefSeq	CDS	1107021	1108130	-	0	ID=cds1161	0	4,5	STM14_1203	STM14_1203	putative iron-sulfur protein	0,568400662	-1,710438693
NC_016856	RefSeq	CDS	1108229	1110337	+	0	ID=cds1162	0	21,5	STM14_1204	ycbY	23S rRNA m%282%29G2445 methyltransferase	3,126203642	-2,431590384
NC_016856	RefSeq	CDS	1110350	1112257	+	0	ID=cds1163	0	23	STM14_1205	uup	ABC transporter ATPase	3,268303807	-2,432747446
NC_016856	RefSeq	CDS	1112272	1113525	+	0	ID=cds1164	0	4,5	STM14_1206	pqiA	paraquat-inducible protein A	0,568400662	-1,710438693
NC_016856	RefSeq	CDS	1113530	1115170	+	0	ID=cds1165	0	12	STM14_1207	pqiB	paraquat-inducible protein B	1,705201986	-2,329925387
NC_016856	RefSeq	CDS	1115167	1115730	+	0	ID=cds1166	0	6,5	STM14_1208	ymbA	putative outer membrane protein	0,852600993	-1,98056697
NC_016856	RefSeq	CDS	1115818	1116021	-	0	ID=cds1167	0	0	STM14_1209	STM14_1209	hypothetical protein	0	NA
NC_016856	RefSeq	CDS	1115985	1116152	+	0	ID=cds1168	0	94	STM14_1210	rmf	ribosome modulation factor	13,35741556	-1,928482877
NC_016856	RefSeq	CDS	1116252	1116770	-	0	ID=cds1169	0	37	STM14_1211	fabA	3-hydroxydecanoyl-ACP dehydratase	5,257706125	-2,272984196
NC_016856	RefSeq	CDS	1116839	1118599	-	0	ID=cds1170	0	54	STM14_1212	lonH	putative protease	7,673408939	-2,144619715
NC_016856	RefSeq	CDS	1118785	1119237	+	0	ID=cds1171	0	10	STM14_1213	ycbG	hypothetical protein	1,421001655	-2,256999172
NC_016856	RefSeq	CDS	1119309	1120361	-	0	ID=cds1172	0	335,3333333	STM14_1214	ompA	outer membrane protein A	47,60355545	-1,932804913
NC_016856	RefSeq	CDS	1120718	1121227	-	0	ID=cds1173	0	10	STM14_1215	sulA	SOS cell division inhibitor	1,421001655	-2,256999172
NC_016856	RefSeq	CDS	1121248	1121352	-	0	ID=cds1174	0	1,5	STM14_1216	STM14_1216	hypothetical protein	0,284200331	-1,182723423
NC_016856	RefSeq	CDS	1121444	1122049	+	0	ID=cds1175	0	6	STM14_1217	yccR	putative DNA transformation protein	0,852600993	-1,98056697
NC_016856	RefSeq	CDS	1122036	1124189	-	0	ID=cds1176	0	12	STM14_1218	yccS	putative transporter	1,705201986	-2,329925387
NC_016856	RefSeq	CDS	1124208	1124654	-	0	ID=cds1177	0	3	STM14_1219	yccF	hypothetical protein	0,426300497	-1,498211557
NC_016856	RefSeq	CDS	1124778	1126832	+	0	ID=cds1178	0	24,5	STM14_1220	helD	DNA helicase IV	3,410403973	-2,434086198
NC_016856	RefSeq	CDS	1126868	1127326	-	0	ID=cds1179	0	23	STM14_1221	mgsA	methylglyoxal synthase	3,268303807	-2,432747446
NC_016856	RefSeq	CDS	1127421	1128083	-	0	ID=cds1180	0	0	STM14_1222	yccT	hypothetical protein	0	NA
NC_016856	RefSeq	CDS	1128254	1128670	+	0	ID=cds1181	0	8	STM14_1223	STM14_1223	putative cytoplasmic protein	1,136801324	-2,147403465
NC_016856	RefSeq	CDS	1128715	1129032	-	0	ID=cds1182	0	7,5	STM14_1224	yccV	putative inner membrane protein	1,136801324	-2,147403465
NC_016856	RefSeq	CDS	1129090	1130301	-	0	ID=cds1183	0	9	STM14_1225	yccW	putative SAM-dependent methyltransferase	1,27890149	-2,207741391
NC_016856	RefSeq	CDS	1130516	1131064	+	0	ID=cds1184	0	2	STM14_1226	STM14_1226	putative outer membrane protein	0,284200331	-1,182723423
NC_016856	RefSeq	CDS	1131090	1131869	+	0	ID=cds1185	0	6,5	STM14_1227	STM14_1227	regulatory protein	0,852600993	-1,98056697
NC_016856	RefSeq	CDS	1131918	1132199	+	0	ID=cds1186	0	6	STM14_1228	yccX	putative phosphohydrolase	0,852600993	-1,98056697
NC_016856	RefSeq	CDS	1132196	1132525	-	0	ID=cds1187	1	7	STM14_1229	yccK	sulfurtransferase TusE	2,754023529	0,552839346
NC_016856	RefSeq	CDS	1132612	1133271	-	0	ID=cds1188	2	43,5	STM14_1230	yccA	hypothetical protein	9,771052023	-0,549311495
NC_016856	RefSeq	CDS	1133892	1134395	-	0	ID=cds1189	0	5	STM14_1232	pipA	pathogenicity island-encoded protein A	0,710500828	-1,864806942
NC_016856	RefSeq	CDS	1134794	1135669	-	0	ID=cds1190	0	6	STM14_1233	pipB	secreted effector protein	0,852600993	-1,98056697
NC_016856	RefSeq	CDS	1135671	1135796	+	0	ID=cds1191	0	0	STM14_1234	STM14_1234	hypothetical protein	0	NA
NC_016856	RefSeq	CDS	1135887	1136207	+	0	ID=cds1192	0	0	STM14_1235	STM14_1235	putative inner membrane protein	0	NA
NC_016856	RefSeq	CDS	1136217	1136558	-	0	ID=cds1193	0	21,08333333	STM14_1236	pipC	pathogenicity island-encoded protein C	2,984103476	-2,430148754
NC_016856	RefSeq	CDS	1136575	1138260	-	0	ID=cds1194	0	45,25	STM14_1237	sopB	secreted effector protein	6,394507449	-2,208821363
NC_016856	RefSeq	CDS	1138592	1138762	+	0	ID=cds1195	0	34,75	STM14_1238	orfX	putative cytoplasmic protein	4,973505794	-2,290637485
NC_016856	RefSeq	CDS	1138763	1138873	-	0	ID=cds1196	0	0,5	STM14_1239	STM14_1239	putative cytoplasmic protein	0	NA
NC_016856	RefSeq	CDS	1138982	1140544	-	0	ID=cds1197	0	23,83333333	STM14_1240	pipD	pathogenicity island-encoded protein D	3,410403973	-2,434086198
NC_016856	RefSeq	CDS	1140624	1141988	-	0	ID=cds1198	0	4,5	STM14_1241	copS	copper resistance protein	0,568400662	-1,710438693
NC_016856	RefSeq	CDS	1141981	1142661	-	0	ID=cds1199	0	5,833333333	STM14_1242	copR	transcriptional regulatory protein YedW	0,852600993	-1,98056697
NC_016856	RefSeq	CDS	1142797	1143207	+	0	ID=cds1200	0	6,5	STM14_1243	STM14_1243	hypothetical protein	0,852600993	-1,98056697
NC_016856	RefSeq	CDS	1143230	1143337	-	0	ID=cds1201	0	0,142857143	STM14_1244	STM14_1244	hypothetical protein	0	NA
NC_016856	RefSeq	CDS	1143361	1143543	+	0	ID=cds1202	0	0,892857143	STM14_1245	STM14_1245	hypothetical protein	0,142100166	-0,623264422
NC_016856	RefSeq	CDS	1143540	1144052	-	0	ID=cds1203	0	1	STM14_1246	hpaC	4-hydroxyphenylacetate catabolism	0,142100166	-0,623264422
NC_016856	RefSeq	CDS	1144070	1145632	-	0	ID=cds1204	0	1,5	STM14_1247	hpaB	4-hydroxyphenylacetate catabolism	0,284200331	-1,182723423
NC_016856	RefSeq	CDS	1145850	1146290	-	0	ID=cds1205	0	3	STM14_1248	hpaR	4-hydroxyphenylacetate catabolism	0,426300497	-1,498211557
NC_016856	RefSeq	CDS	1146275	1146388	-	0	ID=cds1206	0	1	STM14_1249	STM14_1249	hypothetical protein	0,142100166	-0,623264422
NC_016856	RefSeq	CDS	1146565	1147854	+	0	ID=cds1207	0	1	STM14_1250	hpaG	4-hydroxyphenylacetate catabolism	0,142100166	-0,623264422
NC_016856	RefSeq	CDS	1147851	1149317	+	0	ID=cds1208	0	1,5	STM14_1251	hpaE	4-hydroxyphenylacetate catabolism	0,284200331	-1,182723423
NC_016856	RefSeq	CDS	1149319	1150170	+	0	ID=cds1209	0	0	STM14_1252	hpaD	4-hydroxyphenylacetate catabolism	0	NA
NC_016856	RefSeq	CDS	1150180	1150560	+	0	ID=cds1210	0	0	STM14_1253	hpaF	4-hydroxyphenylacetate catabolism	0	NA
NC_016856	RefSeq	CDS	1150549	1150701	-	0	ID=cds1211	0	0	STM14_1254	STM14_1254	hypothetical protein	0	NA
NC_016856	RefSeq	CDS	1150703	1151506	+	0	ID=cds1212	0	1	STM14_1255	hpaH	4-hydroxyphenylacetate catabolism	0,142100166	-0,623264422
NC_016856	RefSeq	CDS	1151517	1152308	+	0	ID=cds1213	0	1	STM14_1256	hpaI	4-hydroxyphenylacetate catabolism	0,142100166	-0,623264422
NC_016856	RefSeq	CDS	1152380	1153756	+	0	ID=cds1214	0	1	STM14_1257	hpaX	4-hydroxyphenylacetate catabolism	0,142100166	-0,623264422
NC_016856	RefSeq	CDS	1153766	1154662	+	0	ID=cds1215	0	0	STM14_1258	hpaA	4-hydroxyphenylacetate catabolism	0	NA
NC_016856	RefSeq	CDS	1154676	1155614	+	0	ID=cds1216	0	0	STM14_1259	STM14_1259	hypothetical protein	0	NA
NC_016856	RefSeq	CDS	1155618	1155782	-	0	ID=cds1217	0	0,5	STM14_1260	STM14_1260	hypothetical protein	0	NA
NC_016856	RefSeq	CDS	1156022	1156384	-	0	ID=cds1218	0	0,5	STM14_1261	STM14_1261	hypothetical protein	0	NA
NC_016856	RefSeq	CDS	1156832	1156987	+	0	ID=cds1219	0	1,5	STM14_1262	STM14_1262	hypothetical protein	0,284200331	-1,182723423
NC_016856	RefSeq	CDS	1157038	1157343	-	0	ID=cds1220	0	5,25	STM14_1263	yccD	chaperone-modulator protein CbpM	0,710500828	-1,864806942
NC_016856	RefSeq	CDS	1157343	1158263	-	0	ID=cds1221	0	16,25	STM14_1264	cbpA	curved DNA-binding protein CbpA	2,273602649	-2,403729808
NC_016856	RefSeq	CDS	1158383	1158577	-	0	ID=cds1222	0	0	STM14_1265	STM14_1265	hypothetical protein	0	NA
NC_016856	RefSeq	CDS	1158498	1158860	+	0	ID=cds1223	0,5	2,25	STM14_1266	scsA	suppression of copper sensitivity protein A	0,284200331	-1,182723423
NC_016856	RefSeq	CDS	1158909	1160795	+	0	ID=cds1224	2	2	STM14_1267	scsB	suppression of copper sensitivity protein	3,802845071	2,200856121
NC_016856	RefSeq	CDS	1160792	1161415	+	0	ID=cds1225	0	0	STM14_1268	scsC	suppression of copper sensitivity protein	0	NA
NC_016856	RefSeq	CDS	1161405	1161911	+	0	ID=cds1226	0	0	STM14_1269	scsD	suppression of copper sensitivity protein	0	NA
NC_016856	RefSeq	CDS	1162044	1163285	+	0	ID=cds1227	0	31,66666667	STM14_1270	agp	glucose-1-phosphatase%2Finositol phosphatase	4,547205297	-2,489878815
NC_016856	RefSeq	CDS	1163319	1163546	-	0	ID=cds1228	0	24	STM14_1271	yccJ	hypothetical protein	3,410403973	-2,434086198
NC_016856	RefSeq	CDS	1163567	1164163	-	0	ID=cds1229	2	31	STM14_1272	wraB	TrpR binding protein WrbA	7,923749872	-0,2118646
NC_016856	RefSeq	CDS	1164294	1164521	+	0	ID=cds1230	0	1	STM14_1273	STM14_1273	hypothetical protein	0,142100166	-0,623264422
NC_016856	RefSeq	CDS	1164548	1164715	+	0	ID=cds1231	0	3,5	STM14_1275	ymdF	putative cytoplasmic protein	0,568400662	-1,710438693
NC_016856	RefSeq	CDS	1164852	1165490	+	0	ID=cds1232	0	3	STM14_1276	ycdC	putative transcriptional repressor	0,426300497	-1,498211557
NC_016856	RefSeq	CDS	1165487	1165882	-	0	ID=cds1233	0	4,5	STM14_1277	STM14_1277	hypothetical protein	0,568400662	-1,710438693
NC_016856	RefSeq	CDS	1165941	1169903	-	0	ID=cds1234	0	32,5	STM14_1278	putA	bifunctional proline dehydrogenase%2Fpyrroline-5-carbox	4,547205297	-2,489878815
NC_016856	RefSeq	CDS	1170030	1170278	-	0	ID=cds1235	0	0	STM14_1279	STM14_1279	hypothetical protein	0	NA
NC_016856	RefSeq	CDS	1170167	1170286	-	0	ID=cds1236	0	0	STM14_1280	STM14_1280	hypothetical protein	0	NA
NC_016856	RefSeq	CDS	1170325	1171833	+	0	ID=cds1237	0	4,333333333	STM14_1281	putP	major sodium%2Fproline symporter	0,568400662	-1,710438693
NC_016856	RefSeq	CDS	1172347	1172457	+	0	ID=cds1238	1	7	STM14_1282	STM14_1282	hypothetical protein	2,754023529	0,552839346
NC_016856	RefSeq	CDS	1172451	1172747	+	0	ID=cds1239	1	4	STM14_1283	STM14_1283	hypothetical protein	2,327723032	1,125268706
NC_016856	RefSeq	CDS	1173620	1174501	-	0	ID=cds1240	0	14,5	STM14_1285	STM14_1285	putative transcriptional regulator	1,989402318	-2,376535557
NC_016856	RefSeq	CDS	1174679	1174783	+	0	ID=cds1241	0	0	STM14_1286	STM14_1286	hypothetical protein	0	NA
NC_016856	RefSeq	CDS	1174786	1176282	-	0	ID=cds1242	0	13,5	STM14_1287	STM14_1287	putative sodium%2Fglucose cotransporter	1,989402318	-2,376535557
NC_016856	RefSeq	CDS	1176314	1176421	+	0	ID=cds1243	0	0	STM14_1288	STM14_1288	hypothetical protein	0	NA
NC_016856	RefSeq	CDS	1176396	1176605	-	0	ID=cds1244	0	1,5	STM14_1289	STM14_1289	hypothetical protein	0,284200331	-1,182723423
NC_016856	RefSeq	CDS	1176619	1177299	-	0	ID=cds1245	0	1,5	STM14_1290	STM14_1290	N-acetylmannosamine-6-phosphate 2-epimerase	0,284200331	-1,182723423
NC_016856	RefSeq	CDS	1177413	1177520	-	0	ID=cds1246	0	0	STM14_1291	STM14_1291	hypothetical protein	0	NA
NC_016856	RefSeq	CDS	1177573	1177704	+	0	ID=cds1247	0	0	STM14_1292	STM14_1292	hypothetical protein	0	NA
NC_016856	RefSeq	CDS	1177805	1178965	+	0	ID=cds1248	0	3	STM14_1293	STM14_1293	putative inner membrane protein	0,426300497	-1,498211557
NC_016856	RefSeq	CDS	1179011	1179703	+	0	ID=cds1249	0	2	STM14_1294	STM14_1294	putative outer membrane protein	0,284200331	-1,182723423
NC_016856	RefSeq	CDS	1179986	1181266	+	0	ID=cds1250	0	0	STM14_1295	STM14_1295	putative sialic acid transporter	0	NA
NC_016856	RefSeq	CDS	1181280	1182383	+	0	ID=cds1251	0	6	STM14_1296	STM14_1296	putative dehydrogenase	0,852600993	-1,98056697
NC_016856	RefSeq	CDS	1183064	1183228	-	0	ID=cds1252	0	0	STM14_1298	STM14_1298	hypothetical protein	0	NA
NC_016856	RefSeq	CDS	1183227	1184165	+	0	ID=cds1253	0	8	STM14_1299	ycdW	putative oxidoreductase	1,136801324	-2,147403465
NC_016856	RefSeq	CDS	1184250	1184987	+	0	ID=cds1254	0	20	STM14_1300	ycdX	putative hydrolase	2,842003311	-2,427810709
NC_016856	RefSeq	CDS	1185011	1185565	+	0	ID=cds1255	0	6	STM14_1301	ycdY	putative oxidoreductase	0,852600993	-1,98056697
NC_016856	RefSeq	CDS	1185618	1186148	+	0	ID=cds1256	0	4	STM14_1302	ycdZ	putative inner membrane protein	0,568400662	-1,710438693
NC_016856	RefSeq	CDS	1186187	1187020	-	0	ID=cds1257	0	0	STM14_1303	csgG	putative curli operon transcriptional regulator	0	NA
NC_016856	RefSeq	CDS	1187047	1187463	-	0	ID=cds1258	0	0	STM14_1304	csgF	curli assembly protein CsgF	0	NA

NC_016856	RefSeq	CDS	1187490	1187885	-	0	ID=cds1259	0	0	STM14_1305	csgE	curli assembly protein CsgE	0	NA
NC_016856	RefSeq	CDS	1187890	1188540	-	0	ID=cds1260	1	0	STM14_1306	csgD	DNA-binding transcriptional regulator CsgD	1,75932237	1,868512432
NC_016856	RefSeq	CDS	1188559	1188696	-	0	ID=cds1261	0	0	STM14_1307	STM14_1307	hypothetical protein	0	NA
NC_016856	RefSeq	CDS	1188894	1189040	-	0	ID=cds1262	0	0	STM14_1308	STM14_1308	hypothetical protein	0	NA
NC_016856	RefSeq	CDS	1189295	1189750	+	0	ID=cds1263	0	0	STM14_1309	csgB	curlin minor subunit	0	NA
NC_016856	RefSeq	CDS	1189792	1190247	+	0	ID=cds1264	0	1	STM14_1310	csgA	cryptic curlin major subunit	0,142100166	-0,623264422
NC_016856	RefSeq	CDS	1190309	1190635	+	0	ID=cds1265	0	2	STM14_1311	csgC	putative autoagglutination protein	0,284200331	-1,182723423
NC_016856	RefSeq	CDS	1190766	1191086	+	0	ID=cds1266	0	1,5	STM14_1312	ymdA	hypothetical protein	0,284200331	-1,182723423
NC_016856	RefSeq	CDS	1191175	1191714	+	0	ID=cds1267	0	10,25	STM14_1313	STM14_1313	hypothetical protein	1,421001655	-2,256999172
NC_016856	RefSeq	CDS	1191650	1193137	+	0	ID=cds1268	0	30,25	STM14_1314	ymdC	putative phospholipase	4,263004966	-2,464060063
NC_016856	RefSeq	CDS	1193154	1194308	-	0	ID=cds1269	0	10	STM14_1315	mdoC	glucans biosynthesis protein	1,421001655	-2,256999172
NC_016856	RefSeq	CDS	1194339	1194509	-	0	ID=cds1270	0	4	STM14_1316	STM14_1316	hypothetical protein	0,568400662	-1,710438693
NC_016856	RefSeq	CDS	1194580	1196115	+	0	ID=cds1271	0	56	STM14_1317	mdoG	glucan biosynthesis protein G	7,95760927	-2,130811198
NC_016856	RefSeq	CDS	1196108	1198651	+	0	ID=cds1272	3	85	STM14_1318	mdoH	glucosyltransferase MdoH	17,35648118	-0,82408297
NC_016856	RefSeq	CDS	1198725	1198952	+	0	ID=cds1273	0	1	STM14_1319	yceK	hypothetical protein	0,142100166	-0,623264422
NC_016856	RefSeq	CDS	1198953	1199327	-	0	ID=cds1274	0	1	STM14_1320	msyB	hypothetical protein	0,142100166	-0,623264422
NC_016856	RefSeq	CDS	1199409	1200623	-	0	ID=cds1275	0	4	STM14_1321	yceE	drug efflux system protein MdtG	0,568400662	-1,710438693
NC_016856	RefSeq	CDS	1200601	1201062	+	0	ID=cds1276	0	1,5	STM14_1322	STM14_1322	hypothetical protein	0,284200331	-1,182723423
NC_016856	RefSeq	CDS	1200778	1201698	-	0	ID=cds1277	0	13,5	STM14_1323	htrB	lipid A biosynthesis lauroyl acyltransferase	1,989402318	-2,376535557
NC_016856	RefSeq	CDS	1201918	1202970	+	0	ID=cds1278	0	1	STM14_1324	yceA	hypothetical protein	0,142100166	-0,623264422
NC_016856	RefSeq	CDS	1203022	1203597	-	0	ID=cds1279	0	1	STM14_1325	yceI	hypothetical protein	0,142100166	-0,623264422
NC_016856	RefSeq	CDS	1203594	1204166	-	0	ID=cds1280	0	0	STM14_1326	STM14_1326	putative inner membrane protein	0	NA
NC_016856	RefSeq	CDS	1204177	1204380	+	0	ID=cds1281	0	0	STM14_1327	STM14_1327	hypothetical protein	0	NA
NC_016856	RefSeq	CDS	1204429	1204542	-	0	ID=cds1282	0	0	STM14_1328	yceO	putative inner membrane protein	0	NA
NC_016856	RefSeq	CDS	1204583	1205701	-	0	ID=cds1283	0	7	STM14_1329	solA	N-methyltryptophan oxidase	0,994701159	-2,072978628
NC_016856	RefSeq	CDS	1205814	1206068	-	0	ID=cds1284	1	14	STM14_1330	bssS	biofilm formation regulatory protein BssS	3,748724687	-0,112256387
NC_016856	RefSeq	CDS	1206358	1206603	-	0	ID=cds1285	0	3,5	STM14_1331	dinI	DNA damage-inducible protein I	0,568400662	-1,710438693
NC_016856	RefSeq	CDS	1206677	1207723	-	0	ID=cds1286	0	12,5	STM14_1332	pyrC	dihydroorotase	1,705201986	-2,329925387
NC_016856	RefSeq	CDS	1207828	1208388	-	0	ID=cds1287	0	21	STM14_1333	yceB	hypothetical protein	2,984103476	-2,430148754
NC_016856	RefSeq	CDS	1208513	1209160	-	0	ID=cds1288	0	18,5	STM14_1334	grxB	glutaredoxin	2,55780298	-2,419166771
NC_016856	RefSeq	CDS	1209224	1210432	-	0	ID=cds1289	0	5	STM14_1335	yceL	multidrug resistance protein MdtH	0,710500828	-1,864806942
NC_016856	RefSeq	CDS	1210669	1211253	+	0	ID=cds1290	0	34	STM14_1336	rimJ	ribosomal-protein-S5-alanine N-acetyltransferase	4,831405628	-2,523251848
NC_016856	RefSeq	CDS	1211289	1211936	+	0	ID=cds1291	0	34,5	STM14_1337	yceH	hypothetical protein	4,831405628	-2,523251848
NC_016856	RefSeq	CDS	1211938	1212861	+	0	ID=cds1292	0	28	STM14_1338	mviM	putative virulence protein	3,978804635	-2,448045729
NC_016856	RefSeq	CDS	1212983	1213159	-	0	ID=cds1293	0	0	STM14_1339	STM14_1339	hypothetical protein	0	NA
NC_016856	RefSeq	CDS	1213126	1214700	+	0	ID=cds1294	0	14	STM14_1340	mviN	putative virulence protein	1,989402318	-2,376535557
NC_016856	RefSeq	CDS	1214782	1215204	-	0	ID=cds1295	0	10,5	STM14_1341	flgN	putative FlgK%2FFlgL export chaperone	1,421001655	-2,256999172
NC_016856	RefSeq	CDS	1215209	1215502	-	0	ID=cds1296	0	4,5	STM14_1342	flgM	anti-sigma-28 factor FlgM	0,568400662	-1,710438693
NC_016856	RefSeq	CDS	1215594	1216253	-	0	ID=cds1297	0	2	STM14_1343	flgA	flagellar basal body P-ring biosynthesis protein FlgA	0,284200331	-1,182723423
NC_016856	RefSeq	CDS	1216241	1216396	-	0	ID=cds1298	0	0	STM14_1344	STM14_1344	flagellar basal body P-ring biosynthesis protein FlgA	0	NA
NC_016856	RefSeq	CDS	1216410	1216826	+	0	ID=cds1299	0	7	STM14_1345	flgB	flagellar basal body rod protein FlgB	0,994701159	-2,072978628
NC_016856	RefSeq	CDS	1216830	1217234	+	0	ID=cds1300	0	3,5	STM14_1346	flgC	flagellar basal body rod protein FlgC	0,568400662	-1,710438693
NC_016856	RefSeq	CDS	1217246	1217944	+	0	ID=cds1301	0	17	STM14_1347	flgD	flagellar basal body rod modification protein	2,415702814	-2,412509737
NC_016856	RefSeq	CDS	1217971	1219182	+	0	ID=cds1302	0	27	STM14_1348	flgE	flagellar hook protein FlgE	3,836704469	-2,44262576
NC_016856	RefSeq	CDS	1219203	1219958	+	0	ID=cds1303	0	2,5	STM14_1349	flgF	flagellar basal body rod protein FlgF	0,284200331	-1,182723423
NC_016856	RefSeq	CDS	1219972	1220754	+	0	ID=cds1304	1	13	STM14_1350	flgG	flagellar basal body rod protein FlgG	3,606624522	-0,044862546
NC_016856	RefSeq	CDS	1220809	1221507	+	0	ID=cds1305	0	9	STM14_1351	flgH	flagellar basal body L-ring protein	1,27890149	-2,207741391
NC_016856	RefSeq	CDS	1221519	1222616	+	0	ID=cds1306	0	15	STM14_1352	flgI	flagellar basal body P-ring protein	2,131502483	-2,392032191
NC_016856	RefSeq	CDS	1222616	1223566	+	0	ID=cds1307	0	10,5	STM14_1353	flgJ	peptidoglycan hydrolase	1,421001655	-2,256999172
NC_016856	RefSeq	CDS	1223631	1225292	+	0	ID=cds1308	0	45,5	STM14_1354	flgK	flagellar hook-associated protein FlgK	6,536607615	-2,201017198
NC_016856	RefSeq	CDS	1225307	1226260	+	0	ID=cds1309	0	22	STM14_1355	flgL	flagellar hook-associated protein FlgL	3,126203642	-2,431590384
NC_016856	RefSeq	CDS	1226280	1226474	-	0	ID=cds1310	0	0	STM14_1356	STM14_1356	hypothetical protein	0	NA
NC_016856	RefSeq	CDS	1226517	1229720	-	0	ID=cds1311	1	139	STM14_1357	rne	ribonuclease E	21,51124538	-2,252425995
NC_016856	RefSeq	CDS	1230294	1231253	+	0	ID=cds1312	0	12	STM14_1359	rluC	23S rRNA pseudouridylate synthase C	1,705201986	-2,329925387
NC_016856	RefSeq	CDS	1231409	1232152	+	0	ID=cds1313	0	11,5	STM14_1360	STM14_1360	putative inner membrane lipoprotein	1,705201986	-2,329925387
NC_016856	RefSeq	CDS	1232358	1232582	+	0	ID=cds1314	0	3	STM14_1361	STM14_1361	hypothetical protein	0,426300497	-1,498211557
NC_016856	RefSeq	CDS	1232654	1233238	-	0	ID=cds1315	0	3	STM14_1362	maf_1	Maf-like protein	0,426300497	-1,498211557
NC_016856	RefSeq	CDS	1233436	1233957	+	0	ID=cds1316	2	94,5	STM14_1363	yceD	hypothetical protein	16,8760603	-1,30493241
NC_016856	RefSeq	CDS	1234009	1234182	+	0	ID=cds1317	0	122	STM14_1364	rpmF	50S ribosomal protein L32	17,3362202	-1,854848636
NC_016856	RefSeq	CDS	1234316	1235395	+	0	ID=cds1318	0	40	STM14_1365	plsX	putative glycerol-3-phosphate acyltransferase PlsX	5,684006621	-2,24950002
NC_016856	RefSeq	CDS	1235475	1236428	+	0	ID=cds1319	0	63	STM14_1366	fabH	3-oxoacyl-ACP synthase	8,952310429	-2,085292348
NC_016856	RefSeq	CDS	1236444	1237373	+	0	ID=cds1320	1	57	STM14_1367	fabD	acyl carrier protein S-malonyltransferase	9,859031805	-1,403762833
NC_016856	RefSeq	CDS	1237386	1238120	+	0	ID=cds1321	2	88	STM14_1368	fabG	3-ketoacyl-ACP reductase	16,02345931	-1,240278832
NC_016856	RefSeq	CDS	1238276	1238512	+	0	ID=cds1322	1	276	STM14_1369	acpP	acyl carrier protein	40,97896806	-2,833621411
NC_016856	RefSeq	CDS	1238598	1239839	+	0	ID=cds1323	0	195	STM14_1370	fabF	3-oxoacyl-%28acyl carrier protein%29 synthase II	27,70953228	-1,898666075
NC_016856	RefSeq	CDS	1239963	1240772	+	0	ID=cds1324	0	6	STM14_1371	pabC	4-amino-4-deoxychorismate lyase	0,852600993	-1,98056697
NC_016856	RefSeq	CDS	1240775	1241797	+	0	ID=cds1325	0	9,5	STM14_1372	yceG	hypothetical protein	1,421001655	-2,256999172
NC_016856	RefSeq	CDS	1241787	1242428	+	0	ID=cds1326	0	7	STM14_1373	tdk_1	thymidylate kinase	0,994701159	-2,072978628
NC_016856	RefSeq	CDS	1242425	1243429	+	0	ID=cds1327	0	18,5	STM14_1374	holB	DNA polymerase III subunit delta%27	2,55780298	-2,419166771
NC_016856	RefSeq	CDS	1243440	1244237	+	0	ID=cds1328	0	21,5	STM14_1375	ycfH	putative metallodependent hydrolase	3,126203642	-2,431590384
NC_016856	RefSeq	CDS	1244261	1244398	-	0	ID=cds1329	0	0	STM14_1376	STM14_1376	hypothetical protein	0	NA
NC_016856	RefSeq	CDS	1244532	1245965	+	0	ID=cds1330	0	408	STM14_1377	ptsG	glucose-specific PTS system IIBC components	57,97686754	-1,941851417
NC_016856	RefSeq	CDS	1246050	1248224	-	0	ID=cds1331	0	0,5	STM14_1378	fhuE	ferric-rhodotorulic acid outer membrane transporter	0	NA
NC_016856	RefSeq	CDS	1248566	1248925	+	0	ID=cds1332	0	17	STM14_1379	ycfF	purine nucleoside phosphoramidase	2,415702814	-2,412509737
NC_016856	RefSeq	CDS	1248928	1249302	+	0	ID=cds1333	0	10	STM14_1380	ycfL	putative outer membrane lipoprotein	1,421001655	-2,256999172
NC_016856	RefSeq	CDS	1249316	1249954	+	0	ID=cds1334	4	9	STM14_1381	ycfM	putative outer membrane lipoprotein	8,31619097	1,593202513
NC_016856	RefSeq	CDS	1249935	1250759	+	0	ID=cds1335	0	7	STM14_1382	thiK	thiamine kinase	0,994701159	-2,072978628
NC_016856	RefSeq	CDS	1250770	1251795	+	0	ID=cds1336	0	13,5	STM14_1383	nagZ	beta-hexosaminidase	1,989402318	-2,376535557
NC_016856	RefSeq	CDS	1251820	1252362	+	0	ID=cds1337	4	24	STM14_1384	ycfP	hypothetical protein	10,44769345	0,704047479
NC_016856	RefSeq	CDS	1252617	1253921	+	0	ID=cds1338	0	7	STM14_1385	ndh	respiratory NADH dehydrogenase 2	0,994701159	-2,072978628
NC_016856	RefSeq	CDS	1254100	1254639	+	0	ID=cds1339	0	5,5	STM14_1386	ycfJ	hypothetical protein	0,852600993	-1,98056697
NC_016856	RefSeq	CDS	1254714	1255349	-	0	ID=cds1340	0	16	STM14_1387	ycfQ	putative transcriptional repressor	2,273602649	-2,403729808
NC_016856	RefSeq	CDS	1255591	1255848	+	0	ID=cds1341	3	3	STM14_1388	ycfR	putative outer membrane protein	5,704267606	2,218275778
NC_016856	RefSeq	CDS	1255945	1256910	-	0	ID=cds1342	0	3	STM14_1389	ycfS	hypothetical protein	0,426300497	-1,498211557
NC_016856	RefSeq	CDS	1256910	1257065	-	0	ID=cds1343	0	1	STM14_1390	STM14_1390	hypothetical protein	0,142100166	-0,623264422
NC_016856	RefSeq	CDS	1257058	1260504	-	0	ID=cds1344	0	27,5	STM14_1391	mfd	transcription-repair coupling factor	3,978804635	-2,448045729
NC_016856	RefSeq	CDS	1260842	1262041	+	0	ID=cds1345	0	19,83333333	STM14_1392	ycfU	outer membrane-specific lipoprotein transporter subunit	2,842003311	-2,427810709
NC_016856	RefSeq	CDS	1262034	1262735	+	0	ID=cds1346	0	8	STM14_1393	lolD	lipoprotein transporter ATP-binding subunit	1,136801324	-2,147403465
NC_016856	RefSeq	CDS	1262735	1263979	+	0	ID=cds1347	0	24,5	STM14_1394	ycfW	outer membrane-specific lipoprotein transporter subunit	3,410403973	-2,434086198
NC_016856	RefSeq	CDS	1264008	1264919	+	0	ID=cds1348	0	36,5	STM14_1395	ycfX	N-acetyl-D-glucosamine kinase	5,115605959	-2,281753063
NC_016856	RefSeq	CDS	1264938	1265759	+	0	ID=cds1349	0	33	STM14_1396	cobB	NAD-dependent deacetylase	4,689305463	-2,505858154
NC_016856	RefSeq	CDS	1265841	1266887	-	0	ID=cds1350	0	32	STM14_1397	potD	spermidine%2Fputrescine ABC transporter substrate-bind	4,547205297	-2,489878815
NC_016856	RefSeq	CDS	1266912	1267691	-	0	ID=cds1351	0	9,5	STM14_1398	potC	spermidine%2Fputrescine ABC transporter membrane prc	1,421001655	-2,256999172
NC_016856	RefSeq	CDS	1267794	1267916	-	0	ID=cds1352	0	8	STM14_1399	STM14_1399	hypothetical protein	1,136801324	-2,147403465
NC_016856	RefSeq	CDS	1268007	1269017	-	0	ID=cds1353	0	3	STM14_1400	sifA	secreted effector protein	0,426300497	-1,498211557
NC_016856	RefSeq	CDS	1269076	1269240	-	0	ID=cds1354	0	0	STM14_1401	STM14_1401	hypothetical protein	0	NA
NC_016856	RefSeq	CDS	1269346	1270209	-	0	ID=cds1355	0	1	STM14_1402	potB	spermidine%2Fputrescine ABC transporter membrane prc	0,142100166	-0,623264422
NC_016856	RefSeq	CDS	1270193	1271329	-	0	ID=cds1356	0	11	STM14_1403	potA	putrescine%2Fspermidine ABC transporter ATPase	1,563101821	-2,297202414
NC_016856	RefSeq	CDS	1271580	1272809	+	0	ID=cds1357	0	65	STM14_1404	pepT	peptidase T	9,23651076	-2,074135889
NC_016856	RefSeq	CDS	1273020	1273478	+	0	ID=cds1358	0	3,3	STM14_1406	tnpA_4	transposase	0,426300497	-1,498211557
NC_016856	RefSeq	CDS	1274898	1276019	-	0	ID=cds1359	0	27,5	STM14_1407	ycfD	putative cytoplasmic protein	3,978804635	-2,448045729
NC_016856	RefSeq	CDS	1276100	1277563	-	0	ID=cds1360	5	98,25	STM14_1408	phoQ	sensor protein PhoQ	22,72242807	-0,462527671
NC_016856	RefSeq	CDS	1277563	1278237	-	0	ID=cds1361	0	63,08333333	STM14_1409	phoP	DNA-binding transcriptional regulator PhoP	8,952310429	-2,085292348
NC_016856	RefSeq	CDS	1278361	1279731	-	0	ID=cds1362	0	29,5	STM14_1410	purB	adenylosuccinate lyase	4,263004966	-2,464060063
NC_016856	RefSeq	CDS	1279735	1280382	-	0	ID=cds1363	0	14,5	STM14_1411	ycfC	hypothetical protein	1,989402318	-2,376535557
NC_016856	RefSeq	CDS	1280463	1281569	-	0	ID=cds1364	0	73,5	STM14_1412	trmU	tRNA %285-methyl aminomethyl-2-thiouridylate%29-met	10,51541225	-2,024396986
NC_016856	RefSeq	CDS	1281623	1282084	-	0	ID=cds1365	0	9	STM14_1413	ymfB	putative MutT-like protein	1,27890149	-2,207741391
NC_016856	RefSeq	CDS	1282096	1282425	-	0	ID=cds1366	0	6,5	STM14_1414	STM14_1414	hypothetical protein	0,852600993	-1,98056697
NC_016856	RefSeq	CDS	1282422	1283087	-	0	ID=cds1367	0	8,5	STM14_1415	ymfC	putative ribosomal large subunit pseudouridine synthase	1,136801324	-2,147403465
NC_016856	RefSeq	CDS	1283259	1284509	+	0	ID=cds1368	0	204,3333333	STM14_1416	icdA	isocitrate dehydrogenase	28,98843377	-1,90211909
NC_016856	RefSeq	CDS	1284622	1285764	-	0	ID=cds1369	0	1	STM14_1417	STM14_1417	prophage lambda integrase	0,142100166	-0,623264422
NC_016856	RefSeq	CDS	1285754	1285990	-	0	ID=cds1370	0	0	STM14_1418	STM14_1418	excisionase	0	NA
NC_016856	RefSeq	CDS	1286040	1286591	-	0	ID=cds1371	0	1	STM14_1419	STM14_1419	Eaa protein	0,142100166	-0,623264422
NC_016856	RefSeq	CDS	1286588	1286920	-	0	ID=cds1372	0	0	STM14_1420	STM14_1420	hypothetical protein	0	NA
NC_016856	RefSeq	CDS	1286913	1287233	-	0	ID=cds1373	0	0	STM14_1421	STM14_1421	hypothetical protein	0	NA
NC_016856	RefSeq	CDS	1287269	1288099	-	0	ID=cds1374	0	1,5	STM14_1422	STM14_1422	recombination and repair protein RecT	0,284200331	-1,182723423
NC_016856	RefSeq	CDS	1290945	1291115	-	0	ID=cds1375	0	0	STM14_1425	STM14_1425	hypothetical protein	0	NA
NC_016856	RefSeq	CDS	1291108	1291224	-	0	ID=cds1376	0	0	STM14_1426	STM14_1426	prophage protein	0	NA
NC_016856	RefSeq	CDS	1291515	1291721	+	0	ID=cds1377	0	3	STM14_1427	STM14_1427	hypothetical protein	0,426300497	-1,498211557
NC_016856	RefSeq	CDS	1291829	1292914	-	0	ID=cds1378	0	81	STM14_1428	STM14_1428	hypothetical protein	11,51011341	-1,988004571
NC_016856	RefSeq	CDS	1293066	1293533	-	0	ID=cds1379	0	24	STM14_1429	STM14_1429	putative regulator	3,410403973	-2,434086198
NC_016856	RefSeq	CDS	1293547	1293774	+	0	ID=cds1380	0	0	STM14_1430	STM14_1430	hypothetical protein	0	NA
NC_016856	RefSeq	CDS	1293794	1294114	+	0	ID=cds1381	0	0	STM14_1431	STM14_1431	prophage cI protein	0	NA
NC_016856	RefSeq	CDS	1294206	1295111	+	0	ID=cds1382	0	0	STM14_1432	STM14_1432	phage replication protein O	0	NA

NC_016856	RefSeq	CDS	1295108	1295800	+	0	ID=cds1383	0	0	STM14_1433	STM14_1433	phage replication protein P	0	NA
NC_016856	RefSeq	CDS	1295815	1296480	+	0	ID=cds1384	0	0	STM14_1434	STM14_1434	Ead-like protein	0	NA
NC_016856	RefSeq	CDS	1296482	1296952	+	0	ID=cds1385	0	2,25	STM14_1435	STM14_1435	DNA N-4 cytosine methyltransferase	0,284200331	-1,182723423
NC_016856	RefSeq	CDS	1296955	1297608	+	0	ID=cds1386	0	0,75	STM14_1436	STM14_1436	Eaa-like protein	0,142100166	-0,623264422
NC_016856	RefSeq	CDS	1297601	1297882	+	0	ID=cds1387	0	0	STM14_1437	STM14_1437	hypothetical protein	0	NA
NC_016856	RefSeq	CDS	1297994	1298185	+	0	ID=cds1388	0	4	STM14_1438	STM14_1438	hypothetical protein	0,568400662	-1,710438693
NC_016856	RefSeq	CDS	1298444	1298677	+	0	ID=cds1389	0	0,5	STM14_1439	STM14_1439	prophage DinI	0	NA
NC_016856	RefSeq	CDS	1298794	1299042	+	0	ID=cds1390	0	2	STM14_1440	STM14_1440	putative bacteriophage protein	0,284200331	-1,182723423
NC_016856	RefSeq	CDS	1299077	1299676	+	0	ID=cds1391	0	14,5	STM14_1441	STM14_1441	IrsA	1,989402318	-2,376535557
NC_016856	RefSeq	CDS	1299673	1299867	+	0	ID=cds1392	0	0	STM14_1442	STM14_1442	hypothetical protein	0	NA
NC_016856	RefSeq	CDS	1299849	1300145	+	0	ID=cds1393	0	0	STM14_1443	STM14_1443	hypothetical protein	0	NA
NC_016856	RefSeq	CDS	1300142	1300696	+	0	ID=cds1394	0	0	STM14_1444	STM14_1444	putative bacteriophage protein	0	NA
NC_016856	RefSeq	CDS	1300693	1300881	+	0	ID=cds1395	0	3	STM14_1445	STM14_1445	hypothetical protein	0,426300497	-1,498211557
NC_016856	RefSeq	CDS	1300963	1301523	+	0	ID=cds1396	0	2	STM14_1446	STM14_1446	putative bacteriophage protein	0,284200331	-1,182723423
NC_016856	RefSeq	CDS	1301766	1301954	+	0	ID=cds1397	0	1	STM14_1447	STM14_1447	hypothetical protein	0,142100166	-0,623264422
NC_016856	RefSeq	CDS	1302108	1302326	+	0	ID=cds1398	0	0	STM14_1448	STM14_1448	hypothetical protein	0	NA
NC_016856	RefSeq	CDS	1302338	1302877	-	0	ID=cds1399	0	14,5	STM14_1449	STM14_1449	hypothetical protein	1,989402318	-2,376535557
NC_016856	RefSeq	CDS	1303055	1303357	+	0	ID=cds1400	0	0	STM14_1450	STM14_1450	phage-holin analog protein	0	NA
NC_016856	RefSeq	CDS	1303335	1303874	+	0	ID=cds1401	0	0	STM14_1451	STM14_1451	lysozyme	0	NA
NC_016856	RefSeq	CDS	1304044	1304181	+	0	ID=cds1402	0	0	STM14_1452	STM14_1452	putative bacteriophage protein	0	NA
NC_016856	RefSeq	CDS	1304183	1304647	+	0	ID=cds1403	0	0,5	STM14_1453	STM14_1453	bacteriophage lysis protein	0	NA
NC_016856	RefSeq	CDS	1304874	1305275	-	0	ID=cds1404	0	1,5	STM14_1454	STM14_1454	hypothetical protein	0,284200331	-1,182723423
NC_016856	RefSeq	CDS	1305561	1306106	+	0	ID=cds1405	0	0	STM14_1455	STM14_1455	prophage DNA packaging protein	0	NA
NC_016856	RefSeq	CDS	1306078	1308009	+	0	ID=cds1406	0	1,5	STM14_1456	STM14_1456	terminase-like large protein	0,284200331	-1,182723423
NC_016856	RefSeq	CDS	1307993	1308196	+	0	ID=cds1407	0	0	STM14_1457	STM14_1457	head-to-tail joining-like protein	0	NA
NC_016856	RefSeq	CDS	1308193	1309773	+	0	ID=cds1408	0	1	STM14_1458	STM14_1458	phage portal protein%2C lambda family	0,142100166	-0,623264422
NC_016856	RefSeq	CDS	1309763	1311259	+	0	ID=cds1409	0	0	STM14_1459	STM14_1459	prophage head-tail preconnector	0	NA
NC_016856	RefSeq	CDS	1311272	1311619	+	0	ID=cds1410	0	0,5	STM14_1460	STM14_1460	phage head-like protein	0	NA
NC_016856	RefSeq	CDS	1311674	1312702	+	0	ID=cds1411	4	1,5	STM14_1461	STM14_1461	phage head-like protein	7,321489811	2,765358925
NC_016856	RefSeq	CDS	1313135	1313518	+	0	ID=cds1412	0	2,75	STM14_1463	STM14_1463	minor capsid protein FII	0,426300497	-1,498211557
NC_016856	RefSeq	CDS	1313546	1314124	+	0	ID=cds1413	0	0,25	STM14_1464	STM14_1464	prophage minor tail protein Z	0	NA
NC_016856	RefSeq	CDS	1314173	1315423	-	0	ID=cds1414	0	16,75	STM14_1465	STM14_1465	Peyer%27s patch-specific virulence factor GipA	2,415702814	-2,412509737
NC_016856	RefSeq	CDS	1315412	1315813	+	0	ID=cds1415	0	0	STM14_1466	STM14_1466	phage minor tail protein U	0	NA
NC_016856	RefSeq	CDS	1315824	1316567	+	0	ID=cds1416	0	2	STM14_1467	STM14_1467	major tail-like protein	0,284200331	-1,182723423
NC_016856	RefSeq	CDS	1316618	1317013	+	0	ID=cds1417	0	0	STM14_1468	STM14_1468	phage minor tail protein G	0	NA
NC_016856	RefSeq	CDS	1317034	1317348	+	0	ID=cds1418	0	0	STM14_1469	STM14_1469	minor tail-like protein	0	NA
NC_016856	RefSeq	CDS	1317320	1320415	+	0	ID=cds1419	0	4	STM14_1470	STM14_1470	phage tail component H-like protein	0,568400662	-1,710438693
NC_016856	RefSeq	CDS	1320418	1320747	+	0	ID=cds1420	0	0,5	STM14_1471	STM14_1471	phage tail component M-like protein	0	NA
NC_016856	RefSeq	CDS	1320757	1321455	+	0	ID=cds1421	0	3,5	STM14_1472	STM14_1472	phage tail component L-like protein	0,568400662	-1,710438693
NC_016856	RefSeq	CDS	1321447	1322052	-	0	ID=cds1422	0	0	STM14_1473	STM14_1473	hypothetical protein	0	NA
NC_016856	RefSeq	CDS	1322136	1322744	+	0	ID=cds1423	0	0	STM14_1474	STM14_1474	tail assembly protein I-like protein	0	NA
NC_016856	RefSeq	CDS	1322806	1326168	+	0	ID=cds1424	0	0	STM14_1475	STM14_1475	host specificity protein	0	NA
NC_016856	RefSeq	CDS	1326207	1326449	+	0	ID=cds1425	0	0	STM14_1476	STM14_1476	hypothetical protein	0	NA
NC_016856	RefSeq	CDS	1326457	1326636	+	0	ID=cds1426	0	0	STM14_1477	STM14_1477	hypothetical protein	0	NA
NC_016856	RefSeq	CDS	1326503	1328944	+	0	ID=cds1427	0	1	STM14_1478	STM14_1478	side tail fiber protein	0,142100166	-0,623264422
NC_016856	RefSeq	CDS	1328944	1329528	+	0	ID=cds1428	0	0	STM14_1479	STM14_1479	phage tail assembly-like protein	0	NA
NC_016856	RefSeq	CDS	1329626	1329826	-	0	ID=cds1429	1	6,5	STM14_1480	STM14_1480	PagJ	2,611923363	0,709005733
NC_016856	RefSeq	CDS	1330854	1331156	+	0	ID=cds1430	0	0	STM14_1481	STM14_1481	hypothetical protein	0	NA
NC_016856	RefSeq	CDS	1332051	1334153	+	0	ID=cds1431	0	7,5	STM14_1483	STM14_1483	SspH1	1,136801324	-2,147403465
NC_016856	RefSeq	CDS	1334473	1335144	-	0	ID=cds1432	0	0	STM14_1484	STM14_1484	hypothetical protein	0	NA
NC_016856	RefSeq	CDS	1335446	1335610	+	0	ID=cds1433	0	1,5	STM14_1485	STM14_1485	isocitrate dehydrogenase	0,284200331	-1,182723423
NC_016856	RefSeq	CDS	1336058	1337182	+	0	ID=cds1434	0	4,5	STM14_1486	STM14_1486	putative cytoplasmic protein	0,568400662	-1,710438693
NC_016856	RefSeq	CDS	1337214	1337372	-	0	ID=cds1435	0	0	STM14_1487	STM14_1487	hypothetical protein	0	NA
NC_016856	RefSeq	CDS	1337312	1337452	+	0	ID=cds1436	0	0,333333333	STM14_1488	STM14_1488	hypothetical protein	0	NA
NC_016856	RefSeq	CDS	1337337	1337585	+	0	ID=cds1437	0	0,333333333	STM14_1489	STM14_1489	hypothetical protein	0	NA
NC_016856	RefSeq	CDS	1338164	1338952	-	0	ID=cds1438	0	0	STM14_1490	envF	putative envelope lipoprotein	0	NA
NC_016856	RefSeq	CDS	1339441	1339680	-	0	ID=cds1439	0	2,5	STM14_1492	msgA	macrophage survival protein	0,284200331	-1,182723423
NC_016856	RefSeq	CDS	1339871	1340392	-	0	ID=cds1440	0	2	STM14_1493	envE	putative envelope protein	0,284200331	-1,182723423
NC_016856	RefSeq	CDS	1340405	1340575	-	0	ID=cds1441	0	2	STM14_1494	STM14_1494	hypothetical protein	0,284200331	-1,182723423
NC_016856	RefSeq	CDS	1340807	1341019	-	0	ID=cds1442	0	8	STM14_1495	STM14_1495	cold shock-like protein	1,136801324	-2,147403465
NC_016856	RefSeq	CDS	1341060	1341188	-	0	ID=cds1443	0,5	0	STM14_1496	STM14_1496	hypothetical protein	0	NA
NC_016856	RefSeq	CDS	1341151	1341414	-	0	ID=cds1444	0,5	1	STM14_1497	pagD	virulence protein PAGD precursor	0,142100166	-0,623264422
NC_016856	RefSeq	CDS	1341718	1341825	+	0	ID=cds1445	0	2	STM14_1499	STM14_1499	hypothetical protein	0,284200331	-1,182723423
NC_016856	RefSeq	CDS	1342043	1342192	-	0	ID=cds1446	0,5	0	STM14_1500	STM14_1500	hypothetical protein	0	NA
NC_016856	RefSeq	CDS	1342226	1342783	+	0	ID=cds1447	5	35	STM14_1501	pagC	virulence membrane protein PAGC precursor	13,77011764	0,56833502
NC_016856	RefSeq	CDS	1342962	1343096	-	0	ID=cds1448	0	0	STM14_1502	STM14_1502	hypothetical protein	0	NA
NC_016856	RefSeq	CDS	1344020	1344364	-	0	ID=cds1449	0	29	STM14_1505	pliC	lysozyme inhibitor	4,120904801	-2,455089955
NC_016856	RefSeq	CDS	1344493	1344636	-	0	ID=cds1450	0	1	STM14_1506	STM14_1506	hypothetical protein	0,142100166	-0,623264422
NC_016856	RefSeq	CDS	1344742	1344867	+	0	ID=cds1451	4	16	STM14_1507	STM14_1507	putative lipoprotein	9,310892128	1,080610291
NC_016856	RefSeq	CDS	1345067	1345339	+	0	ID=cds1452	0	7	STM14_1508	STM14_1508	putative cytoplasmic protein	0,994701159	-2,072978628
NC_016856	RefSeq	CDS	1345491	1345958	+	0	ID=cds1453	0	10,5	STM14_1509	STM14_1509	putative molecular chaperone	1,421001655	-2,256999172
NC_016856	RefSeq	CDS	1346067	1346216	-	0	ID=cds1454	0	0	STM14_1510	STM14_1510	putative inner membrane protein	0	NA
NC_016856	RefSeq	CDS	1346297	1347340	+	0	ID=cds1455	0	5	STM14_1511	STM14_1511	putative cytoplasmic protein	0,710500828	-1,864806942
NC_016856	RefSeq	CDS	1347423	1347953	-	0	ID=cds1456	0	22	STM14_1512	STM14_1512	putative inner membrane protein	3,126203642	-2,431590384
NC_016856	RefSeq	CDS	1348102	1348416	-	0	ID=cds1457	0	41	STM14_1513	STM14_1513	putative outer membrane lipoprotein	5,826106787	-2,241137745
NC_016856	RefSeq	CDS	1348508	1348666	-	0	ID=cds1458	0	30	STM14_1514	STM14_1514	hypothetical protein	4,263004966	-2,464060063
NC_016856	RefSeq	CDS	1349098	1350732	+	0	ID=cds1459	0	19	STM14_1515	STM14_1515	putative ABC transporter periplasmic binding protein	2,699903145	-2,42419778
NC_016856	RefSeq	CDS	1350732	1351706	+	0	ID=cds1460	0	3,5	STM14_1516	STM14_1516	putative ABC transporter protein	0,568400662	-1,710438693
NC_016856	RefSeq	CDS	1351696	1352508	+	0	ID=cds1461	0	2,5	STM14_1517	STM14_1517	putative ABC transporter protein	0,284200331	-1,182723423
NC_016856	RefSeq	CDS	1352502	1353299	+	0	ID=cds1462	0	2,5	STM14_1518	STM14_1518	ABC transporter ATP-binding protein	0,284200331	-1,182723423
NC_016856	RefSeq	CDS	1353293	1353883	+	0	ID=cds1463	0	1,5	STM14_1519	STM14_1519	ABC transporter ATP-binding protein	0,284200331	-1,182723423
NC_016856	RefSeq	CDS	1353965	1355098	-	0	ID=cds1464	0	1	STM14_1520	STM14_1520	putative inner membrane protein	0,142100166	-0,623264422
NC_016856	RefSeq	CDS	1355292	1355618	+	0	ID=cds1465	0	1,166666667	STM14_1521	STM14_1521	putative cytoplasmic protein	0,142100166	-0,623264422
NC_016856	RefSeq	CDS	1355815	1356462	+	0	ID=cds1466	0	2	STM14_1523	STM14_1523	hypothetical protein	0,284200331	-1,182723423
NC_016856	RefSeq	CDS	1356451	1356600	-	0	ID=cds1467	0	0	STM14_1524	STM14_1524	hypothetical protein	0	NA
NC_016856	RefSeq	CDS	1356571	1357359	+	0	ID=cds1468	0	1	STM14_1525	aadA	aminoglycoside resistance protein	0,142100166	-0,623264422
NC_016856	RefSeq	CDS	1357504	1358097	+	0	ID=cds1469	0	0	STM14_1526	STM14_1526	putative response regulator	0	NA
NC_016856	RefSeq	CDS	1358166	1359014	+	0	ID=cds1470	2	26,5	STM14_1527	STM14_1527	putative transcriptional regulator	7,213249044	-0,045799729
NC_016856	RefSeq	CDS	1358977	1359201	+	0	ID=cds1471	0	1	STM14_1528	STM14_1528	hypothetical protein	0,142100166	-0,623264422
NC_016856	RefSeq	CDS	1359269	1359517	-	0	ID=cds1472	0	0	STM14_1529	STM14_1529	putative cytoplasmic protein	0	NA
NC_016856	RefSeq	CDS	1359596	1359745	-	0	ID=cds1473	0	0	STM14_1530	STM14_1530	putative cytoplasmic protein	0	NA
NC_016856	RefSeq	CDS	1359828	1360373	+	0	ID=cds1474	0	3	STM14_1531	STM14_1531	chorismate mutase	0,426300497	-1,498211557
NC_016856	RefSeq	CDS	1360570	1361208	+	0	ID=cds1475	1	36	STM14_1532	yeaS	leucine export protein LeuE	6,874928329	-0,96718351
NC_016856	RefSeq	CDS	1361225	1361380	-	0	ID=cds1476	0	0	STM14_1533	STM14_1533	hypothetical protein	0	NA
NC_016856	RefSeq	CDS	1361381	1361743	+	0	ID=cds1477	0	0	STM14_1534	yeaR	putative cytoplasmic protein	0	NA
NC_016856	RefSeq	CDS	1361746	1361928	+	0	ID=cds1478	0	1	STM14_1535	yoaG	putative cytoplasmic protein	0,142100166	-0,623264422
NC_016856	RefSeq	CDS	1362034	1362159	-	0	ID=cds1479	0	0	STM14_1536	STM14_1536	hypothetical protein	0	NA
NC_016856	RefSeq	CDS	1362158	1362799	+	0	ID=cds1480	0	2	STM14_1537	STM14_1537	putative nitric oxide reductase	0,284200331	-1,182723423
NC_016856	RefSeq	CDS	1362921	1363106	+	0	ID=cds1481	1	1	STM14_1538	STM14_1538	hypothetical protein	1,901422535	2,462014545
NC_016856	RefSeq	CDS	1363114	1363362	+	0	ID=cds1482	3	2	STM14_1539	yeaQ	hypothetical protein	5,562167441	2,509670145
NC_016856	RefSeq	CDS	1363413	1363529	-	0	ID=cds1483	0	0	STM14_1540	STM14_1540	hypothetical protein	0	NA
NC_016856	RefSeq	CDS	1363501	1363599	-	0	ID=cds1484	0	0	STM14_1541	STM14_1541	hypothetical protein	0	NA
NC_016856	RefSeq	CDS	1363795	1364046	+	0	ID=cds1485	0	2	STM14_1542	yaoF	putative hemolysin	0,284200331	-1,182723423
NC_016856	RefSeq	CDS	1364115	1364426	+	0	ID=cds1486	0	9	STM14_1543	STM14_1543	hypothetical protein	1,27890149	-2,207741391
NC_016856	RefSeq	CDS	1364412	1364759	-	0	ID=cds1487	0	0,5	STM14_1544	yeaO	putative cytoplasmic protein	0	NA
NC_016856	RefSeq	CDS	1364743	1364856	-	0	ID=cds1488	0	0	STM14_1545	STM14_1545	hypothetical protein	0	NA
NC_016856	RefSeq	CDS	1364774	1364911	+	0	ID=cds1489	0	0,333333333	STM14_1546	STM14_1546	hypothetical protein	0	NA
NC_016856	RefSeq	CDS	1364878	1366062	-	0	ID=cds1490	0	3	STM14_1547	yeaN	putative amino acid%2Famine transport protein	0,426300497	-1,498211557
NC_016856	RefSeq	CDS	1366163	1366957	+	0	ID=cds1491	0	1	STM14_1548	yeaM	putative regulatory protein	0,142100166	-0,623264422
NC_016856	RefSeq	CDS	1366937	1367383	-	0	ID=cds1492	0	6	STM14_1549	yeaL	putative inner membrane protein	0,852600993	-1,98056697
NC_016856	RefSeq	CDS	1367385	1367522	+	0	ID=cds1493	0	0,5	STM14_1550	STM14_1550	hypothetical protein	0	NA
NC_016856	RefSeq	CDS	1367589	1367696	+	0	ID=cds1494	0	0	STM14_1551	STM14_1551	putative inner membrane protein	0	NA
NC_016856	RefSeq	CDS	1367693	1368211	-	0	ID=cds1495	0	11	STM14_1552	yeaK	putative cytoplasmic protein	1,563101821	-2,297202414
NC_016856	RefSeq	CDS	1368261	1369754	-	0	ID=cds1496	0	20,5	STM14_1553	yeaJ	putative methyl-accepting chemotaxis protein	2,842003311	-2,427810709
NC_016856	RefSeq	CDS	1370048	1370245	+	0	ID=cds1497	0	35	STM14_1554	STM14_1554	hypothetical protein	4,973505794	-2,290637485
NC_016856	RefSeq	CDS	1370136	1370267	+	0	ID=cds1498	0	7	STM14_1555	STM14_1555	salivary secreted protein	0,994701159	-2,072978628
NC_016856	RefSeq	CDS	1370395	1371681	-	0	ID=cds1499	1	2	STM14_1556	yeaH	hypothetical protein	2,043522701	1,824239109
NC_016856	RefSeq	CDS	1371682	1371801	+	0	ID=cds1500	0	0	STM14_1557	STM14_1557	hypothetical protein	0	NA
NC_016856	RefSeq	CDS	1371804	1373738	-	0	ID=cds1501	0	4	STM14_1558	yeaG	putative serine protein kinase	0,568400662	-1,710438693
NC_016856	RefSeq	CDS	1373854	1374027	-	0	ID=cds1502	0	0	STM14_1559	STM14_1559	hypothetical protein	0	NA
NC_016856	RefSeq	CDS	1374166	1374912	+	0	ID=cds1503	0	208,3333333	STM14_1560	mipA	MltA-interacting protein A	29,55683443	-1,903567772
NC_016856	RefSeq	CDS	1375202	1376398	+	0	ID=cds1504	0	0,5	STM14_1561	STM14_1561	arylsulfatase regulator	0	NA
NC_016856	RefSeq	CDS	1376495	1377352	+	0	ID=cds1505	0	5	STM14_1562	STM14_1562	putative aldehyde reductase	0,710500828	-1,864806942
NC_016856	RefSeq	CDS	1377451	1378335	-	0	ID=cds1506	0	9,75	STM14_1563	yeaD	aldose 1-epimerase	1,421001655	-2,256999172

NC_01685(	RefSeq	CDS	1378335	1378511	-	0	ID=cds1507	0	2,25	STM14_1564	STM14_1564	hypothetical protein	0,284200331	-1,182723423
NC_01685(	RefSeq	CDS	1378670	1379665	-	0	ID=cds1508	2	317,0833333	STM14_1565	gapA	glyceraldehyde-3-phosphate dehydrogenase	48,56439721	-2,440005361
NC_01685(	RefSeq	CDS	1379665	1379795	+	0	ID=cds1509	0	0	STM14_1566	STM14_1566	hypothetical protein	0	NA
NC_01685(	RefSeq	CDS	1379977	1380420	+	0	ID=cds1510	0	1	STM14_1567	yeaA	methionine sulfoxide reductase B	0,142100166	-0,623264422
NC_01685(	RefSeq	CDS	1380462	1380740	+	0	ID=cds1511	0	7	STM14_1568	yeaC	putative cytoplasmic protein	0,994701159	-2,072978628
NC_01685(	RefSeq	CDS	1380898	1381002	+	0	ID=cds1512	0	0	STM14_1569	STM14_1569	hypothetical protein	0	NA
NC_01685(	RefSeq	CDS	1381091	1381747	-	0	ID=cds1513	0	8	STM14_1570	pncA	nicotinamidase%2Fpyrazinamidase	1,136801324	-2,147403465
NC_01685(	RefSeq	CDS	1381774	1382790	-	0	ID=cds1514	0	4,25	STM14_1571	ansA	asparaginase	0,568400662	-1,710438693
NC_01685(	RefSeq	CDS	1382785	1382889	+	0	ID=cds1515	0	0	STM14_1572	STM14_1572	hypothetical protein	0	NA
NC_01685(	RefSeq	CDS	1382886	1384742	-	0	ID=cds1516	0	11	STM14_1573	sppA	protease 4	1,563101821	-2,297202414
NC_01685(	RefSeq	CDS	1384916	1385467	+	0	ID=cds1517	0	9	STM14_1574	ydjA	hypothetical protein	1,27890149	-2,207741391
NC_01685(	RefSeq	CDS	1385585	1386628	+	0	ID=cds1518	0	15,5	STM14_1575	selD	selenophosphate synthetase	2,273602649	-2,403729808
NC_01685(	RefSeq	CDS	1386633	1388582	+	0	ID=cds1519	0	24,5	STM14_1576	topB	DNA topoisomerase III	3,410403973	-2,434086198
NC_01685(	RefSeq	CDS	1388612	1389955	-	0	ID=cds1520	0	21	STM14_1577	gdhA	glutamate dehydrogenase	2,984103476	-2,430148754
NC_01685(	RefSeq	CDS	1390194	1390469	+	0	ID=cds1521	0	2,5	STM14_1578	STM14_1578	hypothetical protein	0,284200331	-1,182723423
NC_01685(	RefSeq	CDS	1390429	1390845	-	0	ID=cds1522	0	2	STM14_1579	STM14_1579	pyrimidine %28deoxy%29nucleoside triphosphate pyroph	0,284200331	-1,182723423
NC_01685(	RefSeq	CDS	1390918	1391724	-	0	ID=cds1523	0	8	STM14_1580	xthA	exonuclease III	1,136801324	-2,147403465
NC_01685(	RefSeq	CDS	1391859	1392038	+	0	ID=cds1524	0	0	STM14_1581	STM14_1581	hypothetical protein	0	NA
NC_01685(	RefSeq	CDS	1392170	1393396	+	0	ID=cds1525	0	3	STM14_1582	argD_1	bifunctional succinylornithine transaminase%2Facetylorni	0,426300497	-1,498211557
NC_01685(	RefSeq	CDS	1393387	1394421	+	0	ID=cds1526	0	2	STM14_1583	astA	arginine succinyltransferase	0,284200331	-1,182723423
NC_01685(	RefSeq	CDS	1394418	1395896	+	0	ID=cds1527	0	0	STM14_1584	astD	succinylglutamic semialdehyde dehydrogenase	0	NA
NC_01685(	RefSeq	CDS	1395893	1397236	+	0	ID=cds1528	0	0	STM14_1585	astB	succinylarginine dihydrolase	0	NA
NC_01685(	RefSeq	CDS	1397229	1398197	+	0	ID=cds1529	0	2	STM14_1586	astE	succinylglutamate desuccinylase	0,284200331	-1,182723423
NC_01685(	RefSeq	CDS	1398234	1398449	+	0	ID=cds1530	0	0	STM14_1587	STM14_1587	hypothetical protein	0	NA
NC_01685(	RefSeq	CDS	1398533	1399018	+	0	ID=cds1531	0	6	STM14_1588	spy	periplasmic protein	0,852600993	-1,98056697
NC_01685(	RefSeq	CDS	1399093	1400001	-	0	ID=cds1532	3	12	STM14_1589	STM14_1589	nucleotide excision repair endonuclease	6,983169096	1,056079994
NC_01685(	RefSeq	CDS	1400095	1400922	-	0	ID=cds1533	0	13	STM14_1590	nadE	NAD synthetase	1,847302152	-2,356210635
NC_01685(	RefSeq	CDS	1400923	1401129	-	0	ID=cds1534	0	0,5	STM14_1591	STM14_1591	hypothetical protein	0	NA
NC_01685(	RefSeq	CDS	1401141	1401482	+	0	ID=cds1535	0	21	STM14_1592	osmE	DNA-binding transcriptional activator OsmE	2,984103476	-2,430148754
NC_01685(	RefSeq	CDS	1401610	1401732	+	0	ID=cds1536	0	0	STM14_1593	STM14_1593	hypothetical protein	0	NA
NC_01685(	RefSeq	CDS	1401783	1402103	+	0	ID=cds1537	0	7,5	STM14_1594	celA	PTS system N%2CN%27-diacetylchitobiose-specific transp	1,136801324	-2,147403465
NC_01685(	RefSeq	CDS	1402186	1403544	+	0	ID=cds1538	0	5,5	STM14_1595	celB	PTS system N%2CN%27-diacetylchitobiose-specific transp	0,852600993	-1,98056697
NC_01685(	RefSeq	CDS	1403595	1403942	+	0	ID=cds1539	0	8	STM14_1596	celC	PTS system N%2CN%27-diacetylchitobiose-specific transp	1,136801324	-2,147403465
NC_01685(	RefSeq	CDS	1403953	1404795	+	0	ID=cds1540	0	3	STM14_1597	celD	DNA-binding transcriptional regulator ChbR	0,426300497	-1,498211557
NC_01685(	RefSeq	CDS	1404920	1406275	+	0	ID=cds1541	0	3,5	STM14_1598	celF	phospho-beta-glucosidase%2Fcellobiose-6-phosphate hyc	0,568400662	-1,710438693
NC_01685(	RefSeq	CDS	1406288	1407046	+	0	ID=cds1542	0	6	STM14_1599	celG	hypothetical protein	0,852600993	-1,98056697
NC_01685(	RefSeq	CDS	1407090	1409342	-	0	ID=cds1543	0	14	STM14_1600	katE	hydroperoxidase II	1,989402318	-2,376535557
NC_01685(	RefSeq	CDS	1409372	1409509	+	0	ID=cds1544	0	0	STM14_1601	STM14_1601	hypothetical protein	0	NA
NC_01685(	RefSeq	CDS	1409399	1409647	-	0	ID=cds1545	0	0	STM14_1602	STM14_1602	hypothetical protein	0	NA
NC_01685(	RefSeq	CDS	1409707	1409949	+	0	ID=cds1546	0	0	STM14_1603	cedA	cell division modulator	0	NA
NC_01685(	RefSeq	CDS	1410051	1411442	-	0	ID=cds1547	0	7	STM14_1604	ydjN	kinase%2Ftransporter-like protein	0,994701159	-2,072978628
NC_01685(	RefSeq	CDS	1411579	1412166	-	0	ID=cds1548	0	2,5	STM14_1605	ydjM	hypothetical protein	0,284200331	-1,182723423
NC_01685(	RefSeq	CDS	1412316	1412984	-	0	ID=cds1549	0	8,5	STM14_1606	yniC	2-deoxyglucose-6-phosphatase	1,136801324	-2,147403465
NC_01685(	RefSeq	CDS	1413133	1413669	+	0	ID=cds1550	0	2,5	STM14_1607	yniB	putative regulatory protein	0,284200331	-1,182723423
NC_01685(	RefSeq	CDS	1413712	1414572	-	0	ID=cds1551	0	25,5	STM14_1608	STM14_1608	putative cytoplasmic protein	3,694604304	-2,438835558
NC_01685(	RefSeq	CDS	1414679	1414969	-	0	ID=cds1552	0	4	STM14_1609	ydiZ	putative cytoplasmic protein	0,568400662	-1,710438693
NC_01685(	RefSeq	CDS	1415066	1415998	-	0	ID=cds1553	0	13,5	STM14_1610	pfkB	6-phosphofructokinase	1,989402318	-2,376535557
NC_01685(	RefSeq	CDS	1416287	1417045	+	0	ID=cds1554	0	8,5	STM14_1611	ydiY	putative outer membrane protein	1,136801324	-2,147403465
NC_01685(	RefSeq	CDS	1417094	1418053	-	0	ID=cds1555	0	38,5	STM14_1612	STM14_1612	putative outer membrane protein	5,39980629	-2,264331336
NC_01685(	RefSeq	CDS	1418196	1418510	+	0	ID=cds1556	0	3	STM14_1613	STM14_1613	putative inner membrane protein	0,426300497	-1,498211557
NC_01685(	RefSeq	CDS	1418507	1419361	+	0	ID=cds1557	0	12	STM14_1614	STM14_1614	putative DNA%2FRNA non-specific endonuclease	1,705201986	-2,329925387
NC_01685(	RefSeq	CDS	1419534	1419740	+	0	ID=cds1558	0	2	STM14_1615	STM14_1615	hypothetical protein	0,284200331	-1,182723423
NC_01685(	RefSeq	CDS	1420063	1421286	-	0	ID=cds1559	0	9	STM14_1616	rfc	O-antigen polymerase	1,27890149	-2,207741391
NC_01685(	RefSeq	CDS	1421769	1421954	+	0	ID=cds1560	0	1,5	STM14_1617	STM14_1617	hypothetical protein	0,284200331	-1,182723423
NC_01685(	RefSeq	CDS	1421956	1422090	+	0	ID=cds1561	0	8	STM14_1618	STM14_1618	hypothetical protein	1,136801324	-2,147403465
NC_01685(	RefSeq	CDS	1422178	1424106	+	0	ID=cds1562	1	337,25	STM14_1619	thrS	threonyl-tRNA synthetase	49,64707816	-3,011752306
NC_01685(	RefSeq	CDS	1424110	1424652	+	0	ID=cds1563	10	348,4166667	STM14_1620	infC	translation initiation factor IF-3	67,04408131	-1,058016746
NC_01685(	RefSeq	CDS	1424748	1424945	+	0	ID=cds1564	0	122,5	STM14_1621	rpmI	50S ribosomal protein L35	17,3362202	-1,854848636
NC_01685(	RefSeq	CDS	1424996	1425352	+	0	ID=cds1565	0	324	STM14_1622	rplT	50S ribosomal protein L20	46,04045363	-1,931114242
NC_01685(	RefSeq	CDS	1425398	1425562	-	0	ID=cds1566	0	0	STM14_1623	STM14_1623	hypothetical protein	0	NA
NC_01685(	RefSeq	CDS	1425654	1426637	+	0	ID=cds1567	0	21,5	STM14_1624	pheS	phenylalanyl-tRNA synthetase subunit alpha	3,126203642	-2,431590384
NC_01685(	RefSeq	CDS	1426653	1429040	+	0	ID=cds1568	0	128,5	STM14_1625	pheT	phenylalanyl-tRNA synthetase subunit beta	18,18882119	-1,860051483
NC_01685(	RefSeq	CDS	1429045	1429344	+	0	ID=cds1569	0	45,5	STM14_1626	ihfA	integration host factor subunit alpha	6,536607615	-2,201017198
NC_01685(	RefSeq	CDS	1429547	1430527	+	0	ID=cds1570	0	7	STM14_1627	btuC	vitamin B12-transporter permease	0,994701159	-2,072978628
NC_01685(	RefSeq	CDS	1430619	1431170	+	0	ID=cds1571	0	3,5	STM14_1628	btuE	putative glutathione peroxidase	0,568400662	-1,710438693
NC_01685(	RefSeq	CDS	1431170	1431919	+	0	ID=cds1572	0	7,5	STM14_1629	btuD	vitamin B12-transporter ATPase	1,136801324	-2,147403465
NC_01685(	RefSeq	CDS	1431996	1432460	+	0	ID=cds1573	0	4,5	STM14_1630	nlpC	lipoprotein	0,568400662	-1,710438693
NC_01685(	RefSeq	CDS	1432577	1432696	-	0	ID=cds1574	0	0	STM14_1631	STM14_1631	hypothetical protein	0	NA
NC_01685(	RefSeq	CDS	1432774	1433487	+	0	ID=cds1575	0	13	STM14_1632	ydiV	hypothetical protein	1,847302152	-2,356210635
NC_01685(	RefSeq	CDS	1433549	1434991	+	0	ID=cds1576	0	8	STM14_1633	ydiU	hypothetical protein	1,136801324	-2,147403465
NC_01685(	RefSeq	CDS	1435026	1435217	-	0	ID=cds1577	0	0	STM14_1634	ydiE	hypothetical protein	0	NA
NC_01685(	RefSeq	CDS	1435374	1436420	-	0	ID=cds1578	0	5	STM14_1635	aroH	phospho-2-dehydro-3-deoxyheptonate aldolase	0,710500828	-1,864806942
NC_01685(	RefSeq	CDS	1436467	1436571	+	0	ID=cds1579	0	0	STM14_1636	STM14_1636	hypothetical protein	0	NA
NC_01685(	RefSeq	CDS	1436576	1437409	-	0	ID=cds1580	0	12,5	STM14_1637	ydiA	hypothetical protein	1,705201986	-2,329925387
NC_01685(	RefSeq	CDS	1437586	1437699	-	0	ID=cds1581	0	0	STM14_1638	STM14_1638	hypothetical protein	0	NA
NC_01685(	RefSeq	CDS	1437746	1440124	+	0	ID=cds1582	4	262,25	STM14_1639	pps	phosphoenolpyruvate synthase	44,26753285	-1,650284865
NC_01685(	RefSeq	CDS	1440166	1441806	-	0	ID=cds1583	0	7	STM14_1640	ydiD	short chain acyl-CoA synthetase	0,994701159	-2,072978628
NC_01685(	RefSeq	CDS	1441896	1442189	-	0	ID=cds1584	0	0	STM14_1641	ydiT	putative ferredoxin	0	NA
NC_01685(	RefSeq	CDS	1442186	1443472	-	0	ID=cds1585	0	0,5	STM14_1642	ydiS	hypothetical protein	0	NA
NC_01685(	RefSeq	CDS	1443529	1444464	-	0	ID=cds1586	0	6	STM14_1643	ydiR	electron transfer flavoprotein subunit YdiR	0,852600993	-1,98056697
NC_01685(	RefSeq	CDS	1444486	1445250	-	0	ID=cds1587	0	0	STM14_1644	ydiQ	putative electron transfer flavoprotein YdiQ	0	NA
NC_01685(	RefSeq	CDS	1445506	1445658	-	0	ID=cds1588	0	0	STM14_1645	STM14_1645	hypothetical protein	0	NA
NC_01685(	RefSeq	CDS	1445713	1446603	+	0	ID=cds1589	0	0	STM14_1646	ydiP	putative transcriptional regulator	0	NA
NC_01685(	RefSeq	CDS	1446679	1447830	-	0	ID=cds1590	0	2	STM14_1647	ydiO	putative acyl-CoA dehydrogenase	0,284200331	-1,182723423
NC_01685(	RefSeq	CDS	1447844	1449439	-	0	ID=cds1591	0	0	STM14_1648	ydiF	putative acetyl-CoA%2Facetoacetyl-CoA transferase beta	0	NA
NC_01685(	RefSeq	CDS	1449593	1450351	-	0	ID=cds1592	0	4	STM14_1649	aroD	3-dehydroquinate dehydratase	0,568400662	-1,710438693
NC_01685(	RefSeq	CDS	1450396	1451262	-	0	ID=cds1593	0	2	STM14_1650	aroE_1	quinate%2Fshikimate dehydrogenase	0,284200331	-1,182723423
NC_01685(	RefSeq	CDS	1451329	1452564	-	0	ID=cds1594	0	1	STM14_1651	ydiN	putative transport protein	0,142100166	-0,623264422
NC_01685(	RefSeq	CDS	1452598	1452744	+	0	ID=cds1595	0	0	STM14_1652	STM14_1652	hypothetical protein	0	NA
NC_01685(	RefSeq	CDS	1452728	1452838	-	0	ID=cds1596	0	0	STM14_1653	STM14_1653	hypothetical protein	0	NA
NC_01685(	RefSeq	CDS	1452928	1454139	-	0	ID=cds1597	0	0	STM14_1654	ydiM	putative transport protein	0	NA
NC_01685(	RefSeq	CDS	1454280	1454639	-	0	ID=cds1598	0	1	STM14_1655	ydiL	putative cytoplasmic protein	0,142100166	-0,623264422
NC_01685(	RefSeq	ncRNA	1454807	1454913	-	.	ID=rna28;P	1	6,25	STM14_1656	rprA	regulatory RNA	2,611923363	0,709005733
NC_01685(	RefSeq	CDS	1455097	1456215	-	0	ID=cds1599	0	5	STM14_1657	ydiK	putative inner membrane protein	0,710500828	-1,864806942
NC_01685(	RefSeq	CDS	1456308	1456475	-	0	ID=cds1600	0	0,5	STM14_1658	STM14_1658	hypothetical protein	0	NA
NC_01685(	RefSeq	CDS	1456480	1459536	+	0	ID=cds1601	0	37,5	STM14_1659	ydiJ	putative oxidase	5,39980629	-2,264331336
NC_01685(	RefSeq	CDS	1459533	1459943	+	0	ID=cds1602	0	3,5	STM14_1660	STM14_1660	hypothetical protein	0,568400662	-1,710438693
NC_01685(	RefSeq	CDS	1460003	1460230	+	0	ID=cds1603	2	54	STM14_1661	ydiH	putative cytoplasmic protein	11,19205368	-0,751843812
NC_01685(	RefSeq	CDS	1460518	1461864	-	0	ID=cds1604	0	5	STM14_1662	STM14_1662	putative Na%2B-dicarboxylate symporter	0,710500828	-1,864806942
NC_01685(	RefSeq	CDS	1462291	1462659	+	0	ID=cds1605	0	0,5	STM14_1663	sufA	iron-sulfur cluster assembly scaffold protein	0	NA
NC_01685(	RefSeq	CDS	1462668	1464155	+	0	ID=cds1606	0	1	STM14_1664	sufB	cysteine desulfurase activator complex subunit SufB	0,142100166	-0,623264422
NC_01685(	RefSeq	CDS	1464172	1464918	+	0	ID=cds1607	0	1	STM14_1665	sufC	cysteine desulfurase	0,142100166	-0,623264422
NC_01685(	RefSeq	CDS	1464893	1466164	+	0	ID=cds1608	0	4	STM14_1666	sufD	cysteine desulfurase activator complex subunit SufD	0,568400662	-1,710438693
NC_01685(	RefSeq	CDS	1466161	1467381	+	0	ID=cds1609	0	2	STM14_1667	sufS	selenocysteine lyase	0,284200331	-1,182723423
NC_01685(	RefSeq	CDS	1467394	1467810	+	0	ID=cds1610	0	0,5	STM14_1668	ynhA	cysteine desufuration protein SufE	0	NA
NC_01685(	RefSeq	CDS	1467965	1468966	+	0	ID=cds1611	0	2	STM14_1669	ynhG	hypothetical protein	0,284200331	-1,182723423
NC_01685(	RefSeq	CDS	1469033	1469272	-	0	ID=cds1612	0	5,5	STM14_1670	lppB	putative methyl-accepting chemotaxis protein	0,852600993	-1,98056697
NC_01685(	RefSeq	CDS	1469355	1469591	-	0	ID=cds1613	4	1845,166667	STM14_1671	lpp	murein lipoprotein	269,2120949	-3,269668649
NC_01685(	RefSeq	CDS	1469902	1471314	-	0	ID=cds1614	0	69,83333333	STM14_1672	pykF	pyruvate kinase	9,947011588	-2,04667217
NC_01685(	RefSeq	CDS	1471716	1473059	-	0	ID=cds1615	0	13,5	STM14_1673	orf48	putative amino acid permease	1,989402318	-2,376535557
NC_01685(	RefSeq	CDS	1473081	1473974	-	0	ID=cds1616	0	13	STM14_1674	orf32	putative proline iminopeptidase	1,847302152	-2,356210635
NC_01685(	RefSeq	CDS	1474033	1474770	-	0	ID=cds1617	0	9	STM14_1675	orf245	putative cytoplasmic protein	1,27890149	-2,207741391
NC_01685(	RefSeq	CDS	1474782	1476008	-	0	ID=cds1618	0	2	STM14_1676	orf408	putative regulatory protein	0,284200331	-1,182723423
NC_01685(	RefSeq	CDS	1476331	1479393	-	0	ID=cds1619	0	4,5	STM14_1677	ttrA	tetrathionate reductase complex subunit A	0,568400662	-1,710438693
NC_01685(	RefSeq	CDS	1479386	1480408	-	0	ID=cds1620	0	1,5	STM14_1678	ttrC	tetrathionate reductase complex subunit C	0,284200331	-1,182723423
NC_01685(	RefSeq	CDS	1480409	1481161	-	0	ID=cds1621	0	0	STM14_1679	ttrB	tetrathionate reductase complex subunit B	0	NA
NC_01685(	RefSeq	CDS	1481325	1483103	+	0	ID=cds1622	0	86	STM14_1680	ttrS	sensory histidine kinase	12,22061424	-1,963849549
NC_01685(	RefSeq	CDS	1483060	1483698	+	0	ID=cds1623	0	17	STM14_1681	ttrR	response regulator	2,415702814	-2,412509737
NC_01685(	RefSeq	CDS	1483796	1484008	+	0	ID=cds1624	0	6	STM14_1682	orf70	hypothetical protein	0,852600993	-1,98056697
NC_01685(	RefSeq	CDS	1484104	1485063	-	0	ID=cds1625	2	12,5	STM14_1683	orf319	putative inner membrane protein	5,223846726	0,666065355
NC_01685(	RefSeq	CDS	1485236	1485964	-	0	ID=cds1626	0	3,5	STM14_1684	orf242	putative regulatory protein	0,568400662	-1,710438693
NC_01685(	RefSeq	CDS	1486016	1486129	-	0	ID=cds1627	0	0	STM14_1685	STM14_1685	hypothetical protein	0	NA
NC_01685(	RefSeq	CDS	1486143	1486781	-	0	ID=cds1628	0	4	STM14_1686	ssrB	transcriptional activator	0,568400662	-1,710438693
NC_01685(	RefSeq	CDS	1486812	1489574	-	0	ID=cds1629	0	11,5	STM14_1687	ssrA	sensor kinase	1,705201986	-2,329925387

NC_016856	RefSeq	CDS	1489975	1490376	+	0	ID=cds1630	6	0	STM14_1688	ssaB	secreted effector protein	10,55593422	1,9656886
NC_016856	RefSeq	CDS	1490378	1491871	+	0	ID=cds1631	0,5	2	STM14_1689	ssaC	outer membrane secretin precursor	0,284200331	-1,182723423
NC_016856	RefSeq	CDS	1491852	1493063	+	0	ID=cds1632	0,5	5,5	STM14_1690	ssaD	virulence protein	0,852600993	-1,98056697
NC_016856	RefSeq	CDS	1493071	1493313	+	0	ID=cds1633	3	0,5	STM14_1691	ssaE	secretion system effector	5,27796711	1,945313184
NC_016856	RefSeq	CDS	1493410	1493517	+	0	ID=cds1634	0	0	STM14_1692	STM14_1692	hypothetical protein	0	NA
NC_016856	RefSeq	CDS	1493577	1493903	+	0	ID=cds1635	0	5	STM14_1693	STM14_1693	secretion system chaperone protein	0,710500828	-1,864806942
NC_016856	RefSeq	CDS	1493910	1494500	+	0	ID=cds1636	4	4,5	STM14_1694	STM14_1694	translocation machinery component	7,605690142	2,262948588
NC_016856	RefSeq	CDS	1494497	1494970	+	0	ID=cds1637	0	3,5	STM14_1695	sscA	secretion system chaperone	0,568400662	-1,710438693
NC_016856	RefSeq	CDS	1494973	1496427	+	0	ID=cds1638	16	13,5	STM14_1696	sseC	translocation machinery component	30,13856024	2,547924529
NC_016856	RefSeq	CDS	1496443	1497030	+	0	ID=cds1639	7	5,5	STM14_1697	sseD	translocation machinery component	13,16785758	2,518671465
NC_016856	RefSeq	CDS	1497033	1497449	+	0	ID=cds1640	0	3	STM14_1698	sseE	secreted effector protein	0,426300497	-1,498211557
NC_016856	RefSeq	CDS	1497501	1497935	+	0	ID=cds1641	0	2,5	STM14_1699	sscB	secretion system chaperone	0,284200331	-1,182723423
NC_016856	RefSeq	CDS	1497951	1498733	+	0	ID=cds1642	1	3	STM14_1700	sseF	secreted effector protein	2,185622867	1,419750352
NC_016856	RefSeq	CDS	1498730	1499419	+	0	ID=cds1643	0	2	STM14_1701	sseG	secreted effector protein	0,284200331	-1,182723423
NC_016856	RefSeq	CDS	1499513	1499728	+	0	ID=cds1644	0	2	STM14_1702	ssaG	type III secretion system apparatus protein	0,284200331	-1,182723423
NC_016856	RefSeq	CDS	1499769	1499996	+	0	ID=cds1645	0	4	STM14_1703	ssaH	type III secretion system apparatus protein	0,568400662	-1,710438693
NC_016856	RefSeq	CDS	1500008	1500256	+	0	ID=cds1646	0	0	STM14_1704	ssaI	type III secretion system apparatus protein	0	NA
NC_016856	RefSeq	CDS	1500253	1501002	+	0	ID=cds1647	0	6,5	STM14_1705	ssaJ	needle complex inner membrane lipoprotein	0,852600993	-1,98056697
NC_016856	RefSeq	CDS	1501020	1501568	+	0	ID=cds1648	1	3	STM14_1706	STM14_1706	putative cytoplasmic protein	2,185622867	1,419750352
NC_016856	RefSeq	CDS	1501565	1502239	+	0	ID=cds1649	2	3	STM14_1707	ssaK	type III secretion system apparatus protein	3,944945236	1,874572685
NC_016856	RefSeq	CDS	1502205	1503221	+	0	ID=cds1650	2	8,5	STM14_1708	ssaL	type III secretion system apparatus protein	4,655446064	1,025807982
NC_016856	RefSeq	CDS	1503279	1503647	+	0	ID=cds1651	0	2	STM14_1709	ssaM	type III secretion system apparatus protein	0,284200331	-1,182723423
NC_016856	RefSeq	CDS	1503632	1505677	+	0	ID=cds1652	0	3	STM14_1710	ssaV	secretion system apparatus protein SsaV	0,426300497	-1,498211557
NC_016856	RefSeq	CDS	1505667	1506968	+	0	ID=cds1653	0	6,25	STM14_1711	ssaN	type III secretion system ATPase	0,852600993	-1,98056697
NC_016856	RefSeq	CDS	1506971	1507348	+	0	ID=cds1654	0	0,25	STM14_1712	ssaO	type III secretion system apparatus protein	0	NA
NC_016856	RefSeq	CDS	1507329	1507703	+	0	ID=cds1655	0	0	STM14_1713	ssaP	type III secretion system apparatus protein	0	NA
NC_016856	RefSeq	CDS	1507684	1508652	+	0	ID=cds1656	0	1	STM14_1714	ssaQ	type III secretion system protein	0,142100166	-0,623264422
NC_016856	RefSeq	CDS	1508720	1509367	+	0	ID=cds1657	4	4	STM14_1715	ssaR	type III secretion system protein	7,605690142	2,262948588
NC_016856	RefSeq	CDS	1509364	1509630	+	0	ID=cds1658	2	1	STM14_1716	ssaS	type III secretion system apparatus protein	3,660744905	2,693976715
NC_016856	RefSeq	CDS	1509631	1510410	+	0	ID=cds1659	0	3	STM14_1717	ssaT	type III secretion system apparatus protein	0,426300497	-1,498211557
NC_016856	RefSeq	CDS	1510407	1511465	+	0	ID=cds1660	2	1,5	STM14_1718	ssaU	secretion system apparatus protein SsaU	3,802845071	2,200856121
NC_016856	RefSeq	CDS	1511477	1511602	+	0	ID=cds1661	0	0,333333333	STM14_1719	STM14_1719	hypothetical protein	0	NA
NC_016856	RefSeq	CDS	1511938	1513311	-	0	ID=cds1662	0	6	STM14_1722	ydhE	multidrug efflux protein	0,852600993	-1,98056697
NC_016856	RefSeq	CDS	1513528	1514169	+	0	ID=cds1663	0	14,16666667	STM14_1723	ribE	riboflavin synthase subunit alpha	1,989402318	-2,376535557
NC_016856	RefSeq	CDS	1514211	1515359	-	0	ID=cds1664	0	21	STM14_1724	cfa	cyclopropane-fatty-acyl-phospholipid synthase	2,984103476	-2,430148754
NC_016856	RefSeq	CDS	1515649	1516854	-	0	ID=cds1665	0	3	STM14_1725	ydhC	inner membrane transport protein YdhC	0,426300497	-1,498211557
NC_016856	RefSeq	CDS	1516967	1517899	+	0	ID=cds1666	0	0	STM14_1726	ydhB	putative DNA-binding transcriptional regulator	0	NA
NC_016856	RefSeq	CDS	1517896	1518921	-	0	ID=cds1667	0	8,5	STM14_1727	purR	DNA-binding transcriptional repressor PurR	1,136801324	-2,147403465
NC_016856	RefSeq	CDS	1519171	1519452	+	0	ID=cds1668	0	23	STM14_1728	STM14_1728	hypothetical protein	3,268303807	-2,432747446
NC_016856	RefSeq	CDS	1519389	1519970	-	0	ID=cds1669	0	198,5	STM14_1729	sodB	superoxide dismutase	28,13583278	-1,899848195
NC_016856	RefSeq	CDS	1520097	1520951	-	0	ID=cds1670	0	11,5	STM14_1730	ydhO	putative cell wall-associated hydrolase	1,705201986	-2,329925387
NC_016856	RefSeq	CDS	1521253	1521600	+	0	ID=cds1671	0	28,5	STM14_1731	ydhD	hypothetical protein	3,978804635	-2,448045729
NC_016856	RefSeq	CDS	1521678	1522325	-	0	ID=cds1672	0	18,5	STM14_1732	rnt	ribonuclease T	2,55780298	-2,419166771
NC_016856	RefSeq	CDS	1522426	1522833	-	0	ID=cds1673	0	16	STM14_1733	gloA	glyoxalase I	2,273602649	-2,403729808
NC_016856	RefSeq	CDS	1522902	1523999	-	0	ID=cds1674	0	10	STM14_1734	nemA	N-ethylmaleimide reductase	1,421001655	-2,256999172
NC_016856	RefSeq	CDS	1524058	1524657	-	0	ID=cds1675	0	1	STM14_1735	ydhM	putative transcriptional repressor	0,142100166	-0,623264422
NC_016856	RefSeq	CDS	1524760	1524999	+	0	ID=cds1676	0	0	STM14_1736	ydhL	putative oxidoreductase	0	NA
NC_016856	RefSeq	CDS	1525050	1525946	+	0	ID=cds1677	0	11	STM14_1737	ydhF	putative aldo%2Fketo reductase	1,563101821	-2,297202414
NC_016856	RefSeq	CDS	1526026	1526547	+	0	ID=cds1678	0	1,5	STM14_1738	sodC_2	superoxide dismutase	0,284200331	-1,182723423
NC_016856	RefSeq	CDS	1526523	1528562	-	0	ID=cds1679	0	5,5	STM14_1739	STM14_1739	putative inner membrane protein	0,852600993	-1,98056697
NC_016856	RefSeq	CDS	1528562	1529458	-	0	ID=cds1680	0	2,5	STM14_1740	ydhJ	putative multidrug resistance efflux pump	0,284200331	-1,182723423
NC_016856	RefSeq	CDS	1529425	1529661	-	0	ID=cds1681	0	0	STM14_1741	ydhI	putative inner membrane protein	0	NA
NC_016856	RefSeq	CDS	1529862	1530296	+	0	ID=cds1682	0	47,5	STM14_1742	slyA	transcriptional regulator SlyA	6,820807946	-2,185729286
NC_016856	RefSeq	CDS	1530344	1530811	-	0	ID=cds1683	0	102,3333333	STM14_1743	slyB	putative outer membrane lipoprotein	14,49421688	-1,89737012
NC_016856	RefSeq	CDS	1531083	1532204	+	0	ID=cds1684	0	3	STM14_1744	anmK	anhydro-N-acetylmuramic acid kinase	0,426300497	-1,498211557
NC_016856	RefSeq	CDS	1532292	1532621	+	0	ID=cds1685	0	10,5	STM14_1745	mliC	lysozyme inhibitor	1,421001655	-2,256999172
NC_016856	RefSeq	CDS	1532680	1533336	+	0	ID=cds1686	0	17	STM14_1746	pdxH	pyridoxamine 5%27-phosphate oxidase	2,415702814	-2,412509737
NC_016856	RefSeq	CDS	1533463	1534737	+	0	ID=cds1687	0	70	STM14_1747	tyrS	tyrosyl-tRNA synthetase	9,947011588	-2,04667217
NC_016856	RefSeq	CDS	1534797	1535657	+	0	ID=cds1688	0	13,5	STM14_1748	pdxY	pyridoxamine kinase	1,989402318	-2,376535557
NC_016856	RefSeq	CDS	1535716	1536321	-	0	ID=cds1689	0	27,5	STM14_1749	gst	glutathionine S-transferase	3,978804635	-2,448045729
NC_016856	RefSeq	CDS	1536426	1537931	-	0	ID=cds1690	0	39	STM14_1750	tppB	putative tripeptide transporter permease	5,541906456	-2,25579729
NC_016856	RefSeq	CDS	1538110	1538277	+	0	ID=cds1691	0	0	STM14_1751	STM14_1751	hypothetical protein	0	NA
NC_016856	RefSeq	CDS	1538533	1539168	-	0	ID=cds1692	0	3	STM14_1752	nth	endonuclease III	0,426300497	-1,498211557
NC_016856	RefSeq	CDS	1539168	1539860	-	0	ID=cds1693	0	5	STM14_1753	ydgQ	SoxR-reducing system protein RsxE	0,710500828	-1,864806942
NC_016856	RefSeq	CDS	1539863	1540483	-	0	ID=cds1694	0	7,5	STM14_1754	ydgP	electron transport complex protein RnfG	1,136801324	-2,147403465
NC_016856	RefSeq	CDS	1540487	1541545	-	0	ID=cds1695	0	8,5	STM14_1755	rnfD	electron transport complex protein RnfD	1,136801324	-2,147403465
NC_016856	RefSeq	CDS	1541546	1543753	-	0	ID=cds1696	1	28,5	STM14_1756	STM14_1756	electron transport complex protein RnfC	5,738127005	-0,73683832
NC_016856	RefSeq	CDS	1543746	1544324	-	0	ID=cds1697	0	7	STM14_1757	ydgM	electron transport complex protein RnfB	0,994701159	-2,072978628
NC_016856	RefSeq	CDS	1544324	1544905	-	0	ID=cds1698	0	18,5	STM14_1758	STM14_1758	Na%28%2B%29-translocating NADH-quinone reductase su	2,55780298	-2,419166771
NC_016856	RefSeq	CDS	1544982	1545422	-	0	ID=cds1699	0	20	STM14_1759	ydgK	putative inner membrane protein	2,842003311	-2,427810709
NC_016856	RefSeq	CDS	1545511	1545741	-	0	ID=cds1700	0	22	STM14_1760	ydgT	oriC-binding nucleoid-associated protein	3,126203642	-2,431590384
NC_016856	RefSeq	CDS	1546024	1546179	-	0	ID=cds1701	0	6	STM14_1761	STM14_1761	hypothetical protein	0,852600993	-1,98056697
NC_016856	RefSeq	CDS	1546357	1547397	+	0	ID=cds1702	0	5	STM14_1762	ydgJ	putative oxidoreductase	0,710500828	-1,864806942
NC_016856	RefSeq	CDS	1547472	1548473	-	0	ID=cds1703	0	18,5	STM14_1763	add	adenosine deaminase	2,55780298	-2,419166771
NC_016856	RefSeq	CDS	1548438	1548683	+	0	ID=cds1704	0	0	STM14_1764	STM14_1764	hypothetical protein	0	NA
NC_016856	RefSeq	CDS	1549052	1549171	-	0	ID=cds1705	0	1,5	STM14_1767	STM14_1767	hypothetical protein	0,284200331	-1,182723423
NC_016856	RefSeq	CDS	1549177	1550685	-	0	ID=cds1706	1	35,33333333	STM14_1768	ydgA	hypothetical protein	6,732828164	-0,94151442
NC_016856	RefSeq	CDS	1550788	1551963	-	0	ID=cds1707	0	15,5	STM14_1769	manA	mannose-6-phosphate isomerase	2,273602649	-2,403729808
NC_016856	RefSeq	CDS	1552163	1553809	+	0	ID=cds1708	0	105,5	STM14_1770	fumA	fumarase A	15,06261755	-1,883514636
NC_016856	RefSeq	CDS	1553745	1553906	+	0	ID=cds1709	0	3,5	STM14_1771	STM14_1771	fumarase A	0,568400662	-1,710438693
NC_016856	RefSeq	CDS	1553977	1555380	+	0	ID=cds1710	0	20,5	STM14_1772	fumC	fumarate hydratase	2,842003311	-2,427810709
NC_016856	RefSeq	CDS	1555377	1556306	-	0	ID=cds1711	0	5	STM14_1773	tus	DNA replication terminus site-binding protein	0,710500828	-1,864806942
NC_016856	RefSeq	CDS	1556382	1557683	-	0	ID=cds1712	0	0	STM14_1774	rstB	sensor protein RstB	0	NA
NC_016856	RefSeq	CDS	1557794	1559500	-	0	ID=cds1713	0	0	STM14_1775	STM14_1775	hypothetical protein	0	NA
NC_016856	RefSeq	CDS	1559385	1559690	-	0	ID=cds1714	0	0	STM14_1776	STM14_1776	outer membrane protein	0	NA
NC_016856	RefSeq	CDS	1559653	1560786	-	0	ID=cds1715	0	1	STM14_1777	ompN	outer membrane protein N	0,142100166	-0,623264422
NC_016856	RefSeq	CDS	1561266	1561997	-	0	ID=cds1716	1	54	STM14_1779	rstA	DNA-binding transcriptional regulator RstA	9,432731309	-1,350661723
NC_016856	RefSeq	CDS	1562124	1562459	+	0	ID=cds1717	0	0	STM14_1780	ydgC	putative inner membrane protein	0	NA
NC_016856	RefSeq	CDS	1562521	1563903	-	0	ID=cds1718	0	1,333333333	STM14_1781	ydgI	putative amino acid transporter	0,142100166	-0,623264422
NC_016856	RefSeq	CDS	1563882	1564016	-	0	ID=cds1719	0	0,833333333	STM14_1782	STM14_1782	putative amino acid permease	0,142100166	-0,623264422
NC_016856	RefSeq	CDS	1563914	1564087	-	0	ID=cds1720	0	0,833333333	STM14_1783	STM14_1783	hypothetical protein	0,142100166	-0,623264422
NC_016856	RefSeq	CDS	1564099	1564224	+	0	ID=cds1721	0	0	STM14_1784	STM14_1784	hypothetical protein	0	NA
NC_016856	RefSeq	CDS	1564202	1565146	-	0	ID=cds1722	0	55	STM14_1785	ydgH	hypothetical protein	7,815509104	-2,137668811
NC_016856	RefSeq	CDS	1565234	1565359	+	0	ID=cds1723	0	0	STM14_1786	STM14_1786	hypothetical protein	0	NA
NC_016856	RefSeq	CDS	1565673	1567202	+	0	ID=cds1724	0	67,5	STM14_1787	pntA	NAD%28P%29 transhydrogenase subunit alpha	9,662811256	-2,058244678
NC_016856	RefSeq	CDS	1567213	1568601	+	0	ID=cds1725	1	81,5	STM14_1788	pntB	pyridine nucleotide transhydrogenase	13,41153594	-1,765713511
NC_016856	RefSeq	CDS	1568776	1569810	-	0	ID=cds1726	0	10,5	STM14_1789	tqsA	putative transport protein	1,421001655	-2,256999172
NC_016856	RefSeq	CDS	1569946	1570116	+	0	ID=cds1727	3	30,66666667	STM14_1790	STM14_1790	hypothetical protein	9,683072241	0,174550408
NC_016856	RefSeq	CDS	1570228	1570590	+	0	ID=cds1728	0	66	STM14_1791	ydgF	multidrug efflux system protein MdtJ	9,378610925	-2,069476383
NC_016856	RefSeq	CDS	1570577	1570906	+	0	ID=cds1729	0	27	STM14_1792	ydgE	multidrug efflux system protein MdtI	3,836704469	-2,44262576
NC_016856	RefSeq	CDS	1570946	1571767	-	0	ID=cds1730	0	1,5	STM14_1793	STM14_1793	putative protease	0,284200331	-1,182723423
NC_016856	RefSeq	CDS	1571734	1572027	-	0	ID=cds1731	1	1	STM14_1794	STM14_1794	hypothetical protein	1,901422535	2,462014545
NC_016856	RefSeq	CDS	1572036	1572284	-	0	ID=cds1732	0	1,5	STM14_1795	STM14_1795	acid shock protein	0,284200331	-1,182723423
NC_016856	RefSeq	CDS	1572462	1572653	-	0	ID=cds1733	0	0,5	STM14_1796	STM14_1796	hypothetical protein	0	NA
NC_016856	RefSeq	CDS	1572679	1573932	-	0	ID=cds1734	0	17,33333333	STM14_1797	ynfM	putative transport protein	2,415702814	-2,412509737
NC_016856	RefSeq	CDS	1574052	1574951	+	0	ID=cds1735	0	1	STM14_1798	ynfL	putative transcriptional regulator	0,142100166	-0,623264422
NC_016856	RefSeq	CDS	1575079	1576299	+	0	ID=cds1736	0	23,5	STM14_1799	mlc	pts operon transcriptional repressor	3,410403973	-2,434086198
NC_016856	RefSeq	CDS	1576425	1577120	+	0	ID=cds1737	0	40	STM14_1800	bioD_2	putative dithiobiotin synthetase	5,684006621	-2,24950002
NC_016856	RefSeq	CDS	1577076	1578329	-	0	ID=cds1738	0	3	STM14_1801	STM14_1801	putative voltage-gated ClC-type chloride channel ClcB	0,426300497	-1,498211557
NC_016856	RefSeq	CDS	1578501	1579649	-	0	ID=cds1739	0	3,5	STM14_1802	STM14_1802	proline%2Fglycine betaine transport system	0,568400662	-1,710438693
NC_016856	RefSeq	CDS	1579649	1580296	-	0	ID=cds1740	0	4,5	STM14_1803	STM14_1803	putative ABC transporter permease	0,568400662	-1,710438693
NC_016856	RefSeq	CDS	1580306	1581208	-	0	ID=cds1741	0	3	STM14_1804	STM14_1804	putative ABC transporter periplasmic component	0,426300497	-1,498211557
NC_016856	RefSeq	CDS	1581237	1581947	-	0	ID=cds1742	0	0	STM14_1805	STM14_1805	putative transport system permease component	0	NA
NC_016856	RefSeq	CDS	1582252	1582866	-	0	ID=cds1743	0	24	STM14_1806	ynfI	twin-argninine leader-binding protein DmsD	3,410403973	-2,434086198
NC_016856	RefSeq	CDS	1582909	1583766	-	0	ID=cds1744	1	12,5	STM14_1807	STM14_1807	putative dimethylsulfoxide reductase	3,464524356	0,028939145
NC_016856	RefSeq	CDS	1583768	1584385	-	0	ID=cds1745	0	6,5	STM14_1808	STM14_1808	putative dimethyl sulfoxide reductase	0,852600993	-1,98056697
NC_016856	RefSeq	CDS	1584396	1586831	-	0	ID=cds1746	0	30	STM14_1809	STM14_1809	putative dimethyl sulfoxide reductase	4,263004966	-2,464060063
NC_016856	RefSeq	CDS	1586812	1587033	-	0	ID=cds1747	0	2	STM14_1810	STM14_1810	putative dimethyl sulfoxide reductase subunit A	0,284200331	-1,182723423
NC_016856	RefSeq	CDS	1586930	1589371	-	0	ID=cds1748	0	42,5	STM14_1811	STM14_1811	putative dimethyl sulfoxide reductase chain A1	5,968206953	-2,232890319
NC_016856	RefSeq	CDS	1589525	1589833	-	0	ID=cds1749	0	6	STM14_1812	ynfD	putative outer membrane protein	0,852600993	-1,98056697
NC_016856	RefSeq	CDS	1589937	1590647	+	0	ID=cds1750	0	5	STM14_1813	ynfC	hypothetical protein	0,710500828	-1,864806942
NC_016856	RefSeq	CDS	1590688	1591248	-	0	ID=cds1751	0	26,5	STM14_1814	speG	spermidine N1-acetyltransferase	3,694604304	-2,438835558
NC_016856	RefSeq	CDS	1591284	1591625	-	0	ID=cds1752	0	23,5	STM14_1815	ynfB	hypothetical protein	3,410403973	-2,434086198
NC_016856	RefSeq	CDS	1591641	1591757	-	0	ID=cds1753	0	3,5	STM14_1816	STM14_1816	hypothetical protein	0,568400662	-1,710438693

NC_01685(	RefSeq	CDS	1591778	1592104	+	0	ID=cds1754	0	1	STM14_1817	ynfA	hypothetical protein	0,142100166	-0,623264422
NC_01685(	RefSeq	CDS	1592226	1593440	+	0	ID=cds1755	0	0	STM14_1818	rspA	putative dehydratase	0	NA
NC_01685(	RefSeq	CDS	1593451	1594470	+	0	ID=cds1756	0	1	STM14_1819	rspB	putative dehydrogenase	0,142100166	-0,623264422
NC_01685(	RefSeq	CDS	1595845	1596036	+	0	ID=cds1757	0	0	STM14_1821	STM14_1821	putative membrane transport protein	0	NA
NC_01685(	RefSeq	CDS	1596047	1597513	+	0	ID=cds1758	2	7,5	STM14_1822	ydfI	putative mannitol dehydrogenase	4,655446064	1,025807982
NC_01685(	RefSeq	CDS	1597580	1597783	-	0	ID=cds1759	0	7	STM14_1823	ydfZ	putative cytoplasmic protein	0,994701159	-2,072978628
NC_01685(	RefSeq	CDS	1597964	1598650	-	0	ID=cds1760	0	5	STM14_1824	ydfH	putative regulatory protein	0,710500828	-1,864806942
NC_01685(	RefSeq	CDS	1598780	1599541	-	0	ID=cds1761	2	45	STM14_1825	ydfG	3-hydroxy acid dehydrogenase	9,913152189	-0,571432525
NC_01685(	RefSeq	CDS	1599665	1601707	+	0	ID=cds1762	0	11,5	STM14_1826	dcp	dipeptidyl carboxypeptidase II	1,705201986	-2,329925387
NC_01685(	RefSeq	CDS	1601704	1601919	-	0	ID=cds1763	0	0	STM14_1827	STM14_1827	hypothetical protein	0	NA
NC_01685(	RefSeq	CDS	1601786	1601923	+	0	ID=cds1764	0	0	STM14_1828	STM14_1828	peptidyl-dipeptidase dcp	0	NA
NC_01685(	RefSeq	CDS	1602052	1602234	+	0	ID=cds1765	0	0	STM14_1829	STM14_1829	putative cytoplasmic protein	0	NA
NC_01685(	RefSeq	CDS	1602354	1602881	-	0	ID=cds1766	0	0	STM14_1830	ydeJ	competence damage-inducible protein A	0	NA
NC_01685(	RefSeq	CDS	1602970	1603068	-	0	ID=cds1767	0	0	STM14_1831	STM14_1831	hypothetical protein	0	NA
NC_01685(	RefSeq	CDS	1603308	1603700	+	0	ID=cds1768	0	2	STM14_1832	ydeI	hypothetical protein	0,284200331	-1,182723423
NC_01685(	RefSeq	CDS	1603865	1604014	-	0	ID=cds1769	0	1	STM14_1833	STM14_1833	hypothetical protein	0,142100166	-0,623264422
NC_01685(	RefSeq	CDS	1604391	1605578	-	0	ID=cds1770	0	6,5	STM14_1834	ydeE	putative MFS-type transporter YdeE	0,852600993	-1,98056697
NC_01685(	RefSeq	CDS	1605806	1606708	+	0	ID=cds1771	1	6	STM14_1835	ydeD	O-acetylserine%2Fcysteine export protein	2,611923363	0,709005733
NC_01685(	RefSeq	CDS	1606827	1607042	-	0	ID=cds1772	0	5,5	STM14_1836	marB	hypothetical protein	0,852600993	-1,98056697
NC_01685(	RefSeq	CDS	1607071	1607454	-	0	ID=cds1773	0	2,5	STM14_1837	marA	DNA-binding transcriptional activator MarA	0,284200331	-1,182723423
NC_01685(	RefSeq	CDS	1607474	1607908	-	0	ID=cds1774	0	3	STM14_1838	marR	DNA-binding transcriptional repressor MarR	0,426300497	-1,498211557
NC_01685(	RefSeq	CDS	1608167	1608832	+	0	ID=cds1775	0	3	STM14_1839	marC	multiple drug resistance protein MarC	0,426300497	-1,498211557
NC_01685(	RefSeq	CDS	1608879	1610069	-	0	ID=cds1776	0	3	STM14_1840	ydeA	sugar efflux transporter	0,426300497	-1,498211557
NC_01685(	RefSeq	CDS	1610185	1611057	-	0	ID=cds1777	0	0	STM14_1841	yneJ	putative transcriptional regulator	0	NA
NC_01685(	RefSeq	CDS	1611160	1612548	+	0	ID=cds1778	0	3	STM14_1842	yneI	putative succinate-semialdehyde dehydrogenase	0,426300497	-1,498211557
NC_01685(	RefSeq	CDS	1612621	1613547	+	0	ID=cds1779	1	3	STM14_1843	yneH	glutaminase	2,185622867	1,419750352
NC_01685(	RefSeq	CDS	1613547	1613906	+	0	ID=cds1780	0	1	STM14_1844	yneG	putative cytoplasmic protein	0,142100166	-0,623264422
NC_01685(	RefSeq	CDS	1614130	1615077	+	0	ID=cds1781	0	3	STM14_1845	STM14_1845	putative inner membrane protein	0,426300497	-1,498211557
NC_01685(	RefSeq	CDS	1615133	1615675	-	0	ID=cds1782	0	0	STM14_1846	STM14_1846	putative outer membrane protein	0	NA
NC_01685(	RefSeq	CDS	1615524	1615850	+	0	ID=cds1783	0	0	STM14_1847	STM14_1847	putative inner membrane protein	0	NA
NC_01685(	RefSeq	CDS	1616392	1617510	+	0	ID=cds1784	0	3	STM14_1848	STM14_1848	putative outer membrane protein	0,426300497	-1,498211557
NC_01685(	RefSeq	CDS	1617651	1617992	-	0	ID=cds1785	0	0,5	STM14_1849	STM14_1849	putative hydrogenase	0	NA
NC_01685(	RefSeq	CDS	1618005	1618892	-	0	ID=cds1786	0	1,5	STM14_1850	STM14_1850	dehydrogenase	0,284200331	-1,182723423
NC_01685(	RefSeq	CDS	1618880	1619941	-	0	ID=cds1787	0	6	STM14_1851	STM14_1851	putative hydrogenase	0,852600993	-1,98056697
NC_01685(	RefSeq	CDS	1619959	1620369	-	0	ID=cds1788	0	4	STM14_1852	STM14_1852	putative hydrogenase	0,568400662	-1,710438693
NC_01685(	RefSeq	CDS	1620366	1620665	-	0	ID=cds1789	0	4,5	STM14_1853	STM14_1853	putative hydrogenase protein	0,568400662	-1,710438693
NC_01685(	RefSeq	CDS	1620665	1621273	-	0	ID=cds1790	0	7,5	STM14_1854	STM14_1854	putative hydrogenase maturation protease	1,136801324	-2,147403465
NC_01685(	RefSeq	CDS	1621279	1622022	-	0	ID=cds1791	0	4	STM14_1855	STM14_1855	putative Ni%2FFe hydrogenase 1 b-type cytochrome subu	0,568400662	-1,710438693
NC_01685(	RefSeq	CDS	1621973	1623775	-	0	ID=cds1792	0	4,5	STM14_1856	STM14_1856	putative hydrogenase-1 large subunit	0,568400662	-1,710438693
NC_01685(	RefSeq	CDS	1623778	1624881	-	0	ID=cds1793	0	4,5	STM14_1857	STM14_1857	putative hydrogenase-1 small subunit	0,568400662	-1,710438693
NC_01685(	RefSeq	CDS	1625009	1625161	-	0	ID=cds1794	0	0	STM14_1858	STM14_1858	hypothetical protein	0	NA
NC_01685(	RefSeq	CDS	1625310	1626401	+	0	ID=cds1795	0	9	STM14_1859	STM14_1859	putative hydrolase	1,27890149	-2,207741391
NC_01685(	RefSeq	CDS	1626567	1627238	+	0	ID=cds1796	0	12	STM14_1860	STM14_1860	putative regulatory protein	1,705201986	-2,329925387
NC_01685(	RefSeq	CDS	1627297	1628322	-	0	ID=cds1797	0	3,5	STM14_1861	STM14_1861	putative zinc-binding dehydrogenase	0,568400662	-1,710438693
NC_01685(	RefSeq	CDS	1628297	1629583	-	0	ID=cds1798	0	3	STM14_1862	STM14_1862	putative transport protein	0,426300497	-1,498211557
NC_01685(	RefSeq	CDS	1629760	1631250	-	0	ID=cds1799	0	2,5	STM14_1863	pqaA	PhoPQ-regulated protein	0,284200331	-1,182723423
NC_01685(	RefSeq	CDS	1631386	1632627	-	0	ID=cds1800	0	1	STM14_1864	STM14_1864	putative multidrug efflux protein	0,142100166	-0,623264422
NC_01685(	RefSeq	CDS	1632617	1634125	-	0	ID=cds1801	0	1	STM14_1865	STM14_1865	hypothetical protein	0,142100166	-0,623264422
NC_01685(	RefSeq	CDS	1634170	1634658	+	0	ID=cds1802	0	0	STM14_1866	STM14_1866	putative transcriptional regulator	0	NA
NC_01685(	RefSeq	CDS	1634718	1634879	-	0	ID=cds1803	0	0	STM14_1867	STM14_1867	hypothetical protein	0	NA
NC_01685(	RefSeq	CDS	1634851	1635930	+	0	ID=cds1804	0	4,5	STM14_1868	STM14_1868	putative S-adenosylmethionine%2FtRNA-ribosyltransferas	0,568400662	-1,710438693
NC_01685(	RefSeq	CDS	1635982	1636371	-	0	ID=cds1805	0	2	STM14_1869	STM14_1869	putative translation initiation inhibitor	0,284200331	-1,182723423
NC_01685(	RefSeq	CDS	1636542	1636826	-	0	ID=cds1806	0	0,5	STM14_1870	STM14_1870	putative cytoplasmic protein	0	NA
NC_01685(	RefSeq	CDS	1636816	1637064	-	0	ID=cds1807	1	4	STM14_1871	STM14_1871	putative cytoplasmic protein	2,327723032	1,125268706
NC_01685(	RefSeq	CDS	1637216	1637431	+	0	ID=cds1808	0	0	STM14_1872	STM14_1872	putative phage-related secreted protein	0	NA
NC_01685(	RefSeq	CDS	1637421	1637753	+	0	ID=cds1809	0	0	STM14_1873	STM14_1873	hypothetical protein	0	NA
NC_01685(	RefSeq	CDS	1637896	1638804	+	0	ID=cds1810	0	0	STM14_1874	STM14_1874	putative cytoplasmic protein	0	NA
NC_01685(	RefSeq	CDS	1639903	1640112	-	0	ID=cds1811	0	2	STM14_1876	STM14_1876	hypothetical protein	0,284200331	-1,182723423
NC_01685(	RefSeq	CDS	1640382	1641848	-	0	ID=cds1812	0	8,5	STM14_1877	STM14_1877	putative coiled-coil protein	1,136801324	-2,147403465
NC_01685(	RefSeq	CDS	1642107	1643114	+	0	ID=cds1813	0	9	STM14_1878	STM14_1878	putative transcriptional regulator	1,27890149	-2,207741391
NC_01685(	RefSeq	CDS	1643258	1644709	+	0	ID=cds1814	0	5,5	STM14_1879	STM14_1879	putative Na%2B%2FH%2B antiporter	0,852600993	-1,98056697
NC_01685(	RefSeq	CDS	1644722	1645924	+	0	ID=cds1815	0	5,5	STM14_1880	STM14_1880	putative aminotransferase	0,852600993	-1,98056697
NC_01685(	RefSeq	CDS	1646037	1648112	-	0	ID=cds1816	0	8	STM14_1881	STM14_1881	putative glycosyl hydrolase	1,136801324	-2,147403465
NC_01685(	RefSeq	CDS	1648169	1650697	-	0	ID=cds1817	0	0	STM14_1882	STM14_1882	putative glycosyl hydrolase	0	NA
NC_01685(	RefSeq	CDS	1650694	1652478	-	0	ID=cds1818	0	0	STM14_1883	STM14_1883	putative alpha amylase	0	NA
NC_01685(	RefSeq	CDS	1652607	1653392	-	0	ID=cds1819	0	0	STM14_1884	STM14_1884	putative lipoprotein	0	NA
NC_01685(	RefSeq	CDS	1653535	1653864	-	0	ID=cds1820	0	21,5	STM14_1885	hdeB	acid-resistance protein	3,126203642	-2,431590384
NC_01685(	RefSeq	CDS	1654214	1654645	-	0	ID=cds1821	0	2,5	STM14_1886	osmC	putative envelope protein	0,284200331	-1,182723423
NC_01685(	RefSeq	CDS	1655158	1655373	+	0	ID=cds1822	0	1	STM14_1887	yddX	biofilm-dependent modulation protein	0,142100166	-0,623264422
NC_01685(	RefSeq	CDS	1655468	1655611	+	0	ID=cds1823	0	8,5	STM14_1888	rpsV	30S ribosomal subunit S22	1,136801324	-2,147403465
NC_01685(	RefSeq	CDS	1655788	1657485	+	0	ID=cds1824	0	73,5	STM14_1889	sfcA	malate dehydrogenase	10,51541225	-2,024396986
NC_01685(	RefSeq	CDS	1657503	1657634	+	0	ID=cds1825	0	1,5	STM14_1890	STM14_1890	hypothetical protein	0,284200331	-1,182723423
NC_01685(	RefSeq	CDS	1657771	1658781	+	0	ID=cds1826	0	17	STM14_1891	adhP	alcohol dehydrogenase	2,415702814	-2,412509737
NC_01685(	RefSeq	CDS	1658872	1659528	-	0	ID=cds1827	0	20	STM14_1892	fdnI	formate dehydrogenase-N subunit gamma	2,842003311	-2,427810709
NC_01685(	RefSeq	CDS	1659521	1660405	-	0	ID=cds1828	0	53	STM14_1893	fdnH	formate dehydrogenase-N beta subunit	7,531308773	-2,151665531
NC_01685(	RefSeq	CDS	1660419	1663466	-	0	ID=cds1829	0	98	STM14_1894	fdnG	formate dehydrogenase-N subunit alpha	13,92581622	-1,91259592
NC_01685(	RefSeq	CDS	1663489	1663641	+	0	ID=cds1830	0	0	STM14_1895	STM14_1895	hypothetical protein	0	NA
NC_01685(	RefSeq	CDS	1663744	1664625	+	0	ID=cds1831	0	4	STM14_1896	yddG	hypothetical protein	0,568400662	-1,710438693
NC_01685(	RefSeq	CDS	1664885	1665067	-	0	ID=cds1832	0	1	STM14_1897	STM14_1897	hypothetical protein	0,142100166	-0,623264422
NC_01685(	RefSeq	CDS	1665150	1666238	+	0	ID=cds1833	0	2013,833333	STM14_1898	nmpC	putative outer membrane porin	286,1897334	-1,977115868
NC_01685(	RefSeq	CDS	1666322	1666762	+	0	ID=cds1834	0	20	STM14_1899	STM14_1899	putative cytoplasmic protein	2,842003311	-2,427810709
NC_01685(	RefSeq	CDS	1666787	1668274	-	0	ID=cds1835	7	6	STM14_1900	smvA	methyl viologen resistance	13,16785758	2,518671465
NC_01685(	RefSeq	CDS	1668391	1668969	+	0	ID=cds1836	0	5,5	STM14_1901	STM14_1901	putative transcriptional regulator	0,852600993	-1,98056697
NC_01685(	RefSeq	CDS	1668962	1669093	+	0	ID=cds1837	0	0	STM14_1902	STM14_1902	hypothetical protein	0	NA
NC_01685(	RefSeq	CDS	1669184	1670572	+	0	ID=cds1838	0	0	STM14_1903	narU	nitrate extrusion protein	0	NA
NC_01685(	RefSeq	CDS	1670559	1670702	-	0	ID=cds1839	0	0	STM14_1904	STM14_1904	hypothetical protein	0	NA
NC_01685(	RefSeq	CDS	1670664	1674404	+	0	ID=cds1840	0	2,5	STM14_1905	narZ	nitrate reductase 2 alpha subunit	0,284200331	-1,182723423
NC_01685(	RefSeq	CDS	1674401	1675945	+	0	ID=cds1841	0	0	STM14_1906	narY	nitrate reductase 2 beta subunit	0	NA
NC_01685(	RefSeq	CDS	1675945	1676640	+	0	ID=cds1842	0	0	STM14_1907	narW	nitrate reductase 2 delta subunit	0	NA
NC_01685(	RefSeq	CDS	1676637	1677317	+	0	ID=cds1843	0	0	STM14_1908	narV	nitrate reductase 2 gamma subunit	0	NA
NC_01685(	RefSeq	CDS	1677423	1678316	+	0	ID=cds1844	0	8	STM14_1909	yddE	hypothetical protein	1,136801324	-2,147403465
NC_01685(	RefSeq	CDS	1678352	1679197	-	0	ID=cds1845	0	6	STM14_1910	nhoA	putative arylamine N-acetyltransferase	0,852600993	-1,98056697
NC_01685(	RefSeq	CDS	1679194	1679457	-	0	ID=cds1846	0	2	STM14_1911	STM14_1911	hypothetical protein	0,284200331	-1,182723423
NC_01685(	RefSeq	CDS	1679481	1680113	-	0	ID=cds1847	0	50	STM14_1912	STM14_1912	putative cytoplasmic protein	7,105008277	-2,173389122
NC_01685(	RefSeq	CDS	1680178	1680390	+	0	ID=cds1848	0	0	STM14_1913	STM14_1913	hypothetical protein	0	NA
NC_01685(	RefSeq	CDS	1680335	1680472	-	0	ID=cds1849	0	0	STM14_1914	STM14_1914	hypothetical protein	0	NA
NC_01685(	RefSeq	CDS	1680342	1680476	+	0	ID=cds1850	0	0	STM14_1915	STM14_1915	hypothetical protein	0	NA
NC_01685(	RefSeq	CDS	1680626	1682119	+	0	ID=cds1851	0	0	STM14_1916	ansP	L-asparagine transport protein	0	NA
NC_01685(	RefSeq	CDS	1682196	1682417	-	0	ID=cds1852	0	1	STM14_1917	STM14_1917	putative outer membrane lipoprotein	0,142100166	-0,623264422
NC_01685(	RefSeq	CDS	1682562	1683623	-	0	ID=cds1853	0	9	STM14_1918	STM14_1918	hypothetical protein	1,27890149	-2,207741391
NC_01685(	RefSeq	CDS	1683664	1683783	-	0	ID=cds1854	0	1	STM14_1919	STM14_1919	hypothetical protein	0,142100166	-0,623264422
NC_01685(	RefSeq	CDS	1683887	1686007	+	0	ID=cds1855	0	3	STM14_1920	yncD	putative outer membrane receptor	0,426300497	-1,498211557
NC_01685(	RefSeq	CDS	1686074	1686739	-	0	ID=cds1856	0	2	STM14_1921	yncC	putative DNA-binding transcriptional regulator	0,284200331	-1,182723423
NC_01685(	RefSeq	CDS	1686850	1686969	+	0	ID=cds1857	0	0	STM14_1922	STM14_1922	hypothetical protein	0	NA
NC_01685(	RefSeq	CDS	1686958	1687080	-	0	ID=cds1858	0	0	STM14_1923	STM14_1923	hypothetical protein	0	NA
NC_01685(	RefSeq	CDS	1687073	1688143	-	0	ID=cds1859	0	7,5	STM14_1924	yncB	putative NADP-dependent oxidoreductase	1,136801324	-2,147403465
NC_01685(	RefSeq	CDS	1688322	1688837	+	0	ID=cds1860	0	5,25	STM14_1925	yncA	putative acyltransferase	0,710500828	-1,864806942
NC_01685(	RefSeq	CDS	1688837	1689286	+	0	ID=cds1861	0	0,75	STM14_1926	ydcZ	putative inner membrane protein	0,142100166	-0,623264422
NC_01685(	RefSeq	CDS	1689291	1689524	-	0	ID=cds1862	0	2	STM14_1927	ydcY	putative cytoplasmic protein	0,284200331	-1,182723423
NC_01685(	RefSeq	CDS	1689748	1691067	+	0	ID=cds1863	0	4	STM14_1928	srfA	putative virulence protein	0,568400662	-1,710438693
NC_01685(	RefSeq	CDS	1691072	1694053	+	0	ID=cds1864	0	7,5	STM14_1929	srfB	putative virulence protein	1,136801324	-2,147403465
NC_01685(	RefSeq	CDS	1694050	1696194	+	0	ID=cds1865	0	4	STM14_1930	srfC	putative virulence protein	0,568400662	-1,710438693
NC_01685(	RefSeq	CDS	1696239	1696421	-	0	ID=cds1866	0	2,5	STM14_1931	ydcX	putative inner membrane protein	0,284200331	-1,182723423
NC_01685(	RefSeq	CDS	1696611	1696706	+	0	ID=cds1867	0	1,5	STM14_1932	STM14_1932	hypothetical protein	0,284200331	-1,182723423
NC_01685(	RefSeq	CDS	1696703	1696807	+	0	ID=cds1868	0	0,5	STM14_1933	STM14_1933	hypothetical protein	0	NA
NC_01685(	RefSeq	CDS	1696811	1698235	-	0	ID=cds1869	2	11	STM14_1934	ydcW	gamma-aminobutyraldehyde dehydrogenase	5,081746561	0,743777882
NC_01685(	RefSeq	CDS	1698371	1699795	-	0	ID=cds1870	0	7,5	STM14_1935	ydcR	putative regulatory protein	1,136801324	-2,147403465
NC_01685(	RefSeq	CDS	1699931	1700701	-	0	ID=cds1871	2	3	STM14_1936	pdgL	periplasmic dipeptidase precursor	3,944945236	1,874572685
NC_01685(	RefSeq	CDS	1700960	1701190	-	0	ID=cds1872	2	1,5	STM14_1937	STM14_1937	putative inner membrane protein	3,802845071	2,200856121
NC_01685(	RefSeq	CDS	1701223	1701621	-	0	ID=cds1873	0	0,5	STM14_1938	ugtL	hypothetical protein	0	NA
NC_01685(	RefSeq	CDS	1701681	1701785	-	0	ID=cds1874	0	2,5	STM14_1939	STM14_1939	hypothetical protein	0,284200331	-1,182723423
NC_01685(	RefSeq	CDS	1702158	1703108	+	0	ID=cds1875	6	3,5	STM14_1940	sifB	secreted effector protein	11,12433488	2,678125737
NC_01685(	RefSeq	CDS	1703382	1703612	+	0	ID=cds1876	1	1	STM14_1941	yncJ	hypothetical protein	1,901422535	2,462014545
NC_01685(	RefSeq	CDS	1703613	1705577	-	0	ID=cds1877	0	14,5	STM14_1942	ydcP	putative collagenase	1,989402318	-2,376535557

NC_01685(	RefSeq	CDS	1705656	1706192	-	0	ID=cds1878	0	9	STM14_1943	ydcN	putative repressor	1,27890149	-2,207741391
NC_01685(	RefSeq	CDS	1706283	1707461	+	0	ID=cds1879	0	0,5	STM14_1944	STM14_1944	putative benzoate membrane transport protein	0	NA
NC_01685(	RefSeq	CDS	1707515	1708183	-	0	ID=cds1880	0	23	STM14_1945	STM14_1945	putative outer membrane lipoprotein	3,268303807	-2,432747446
NC_01685(	RefSeq	CDS	1708339	1708935	-	0	ID=cds1881	0	14,5	STM14_1946	tehB	tellurite resistance protein TehB	1,989402318	-2,376535557
NC_01685(	RefSeq	CDS	1708922	1709935	-	0	ID=cds1882	0	9,5	STM14_1947	tehA	potassium-tellurite ethidium and proflavin transporter	1,421001655	-2,256999172
NC_01685(	RefSeq	CDS	1710042	1711022	+	0	ID=cds1883	0	0	STM14_1948	ydcK	putative nucleoside-diphosphate-sugar pyrophosphorylas	0	NA
NC_01685(	RefSeq	CDS	1711019	1711558	-	0	ID=cds1884	0	1,5	STM14_1949	rimL	ribosomal-protein-L7%2FL12-serine acetyltransferase	0,284200331	-1,182723423
NC_01685(	RefSeq	CDS	1711642	1711749	+	0	ID=cds1885	0	0	STM14_1950	STM14_1950	hypothetical protein	0	NA
NC_01685(	RefSeq	CDS	1711775	1712893	+	0	ID=cds1886	0	3,5	STM14_1951	STM14_1951	putative cellulase protein	0,568400662	-1,710438693
NC_01685(	RefSeq	CDS	1712890	1713171	+	0	ID=cds1887	0	1	STM14_1952	STM14_1952	putative PTS system enzymeIIB component	0,142100166	-0,623264422
NC_01685(	RefSeq	CDS	1713182	1714495	+	0	ID=cds1888	0	7	STM14_1953	STM14_1953	putative PTS system enzyme IIC component	0,994701159	-2,072978628
NC_01685(	RefSeq	CDS	1714509	1715315	+	0	ID=cds1889	0	0,5	STM14_1954	STM14_1954	putative nucleoside triphosphatase	0	NA
NC_01685(	RefSeq	CDS	1715447	1715884	+	0	ID=cds1890	0	1,5	STM14_1955	STM14_1955	putative sugar-specific PTS enzyme II	0,284200331	-1,182723423
NC_01685(	RefSeq	CDS	1715896	1716528	+	0	ID=cds1891	0	2	STM14_1956	STM14_1956	ribulose-phosphate 3-epimerase	0,284200331	-1,182723423
NC_01685(	RefSeq	CDS	1716544	1717335	+	0	ID=cds1892	0	9	STM14_1957	STM14_1957	putative transcriptional repressor of sgc operon	1,27890149	-2,207741391
NC_01685(	RefSeq	CDS	1717335	1717772	+	0	ID=cds1893	0	9	STM14_1958	STM14_1958	cryptic aminoglycoside resistance protein	1,27890149	-2,207741391
NC_01685(	RefSeq	CDS	1717819	1719021	-	0	ID=cds1894	0	2,5	STM14_1959	STM14_1959	putative oxidase	0,284200331	-1,182723423
NC_01685(	RefSeq	CDS	1719033	1719608	-	0	ID=cds1895	0	1,5	STM14_1960	STM14_1960	hypothetical protein	0,284200331	-1,182723423
NC_01685(	RefSeq	CDS	1719701	1719814	+	0	ID=cds1896	0	0	STM14_1961	STM14_1961	hypothetical protein	0	NA
NC_01685(	RefSeq	CDS	1719804	1721459	-	0	ID=cds1897	1	18,33333333	STM14_1962	mdoD	glucan biosynthesis protein D	4,31712535	-0,335890347
NC_01685(	RefSeq	CDS	1721679	1723187	-	0	ID=cds1898	0	7	STM14_1963	STM14_1963	putative carboxylesterase	0,994701159	-2,072978628
NC_01685(	RefSeq	CDS	1723236	1724579	-	0	ID=cds1899	0	8,5	STM14_1964	STM14_1964	putative cytoplasmic protein	1,136801324	-2,147403465
NC_01685(	RefSeq	CDS	1724872	1725795	+	0	ID=cds1900	0	0,5	STM14_1965	ydcI	putative transcriptional regulator	0	NA
NC_01685(	RefSeq	CDS	1725856	1727481	-	0	ID=cds1901	0	14,75	STM14_1966	trg	methyl-accepting chemotaxis protein III	2,131502483	-2,392032191
NC_01685(	RefSeq	CDS	1727480	1727695	+	0	ID=cds1902	0	0	STM14_1967	STM14_1967	hypothetical protein	0	NA
NC_01685(	RefSeq	CDS	1727650	1728768	-	0	ID=cds1903	0	4	STM14_1968	STM14_1968	alcohol dehydrogenase class III	0,568400662	-1,710438693
NC_01685(	RefSeq	CDS	1728800	1729075	-	0	ID=cds1904	0	0	STM14_1969	STM14_1969	putative cytoplasmic protein	0	NA
NC_01685(	RefSeq	CDS	1729598	1729999	+	0	ID=cds1905	0	0	STM14_1970	STM14_1970	putative dipicolinate reductase	0	NA
NC_01685(	RefSeq	CDS	1730128	1730844	-	0	ID=cds1906	0	0	STM14_1971	STM14_1971	putative inner membrane protein	0	NA
NC_01685(	RefSeq	CDS	1731028	1731132	-	0	ID=cds1907	0	0	STM14_1972	STM14_1972	hypothetical protein	0	NA
NC_01685(	RefSeq	CDS	1731115	1731225	+	0	ID=cds1908	0	0	STM14_1973	STM14_1973	hypothetical protein	0	NA
NC_01685(	RefSeq	CDS	1731270	1732496	+	0	ID=cds1909	0	0	STM14_1974	sseJ	secreted effector protein	0	NA
NC_01685(	RefSeq	CDS	1732617	1732961	-	0	ID=cds1910	0	0	STM14_1975	STM14_1975	putative inner membrane protein	0	NA
NC_01685(	RefSeq	CDS	1733662	1733814	-	0	ID=cds1911	0	0	STM14_1976	STM14_1976	hypothetical protein	0	NA
NC_01685(	RefSeq	CDS	1733868	1734629	+	0	ID=cds1912	0	0	STM14_1977	STM14_1977	putative periplasmic binding protein	0	NA
NC_01685(	RefSeq	CDS	1734636	1735283	+	0	ID=cds1913	0	0	STM14_1978	STM14_1978	putative ABC transporter permease	0	NA
NC_01685(	RefSeq	CDS	1735308	1736039	+	0	ID=cds1914	0	0	STM14_1979	STM14_1979	putative ABC-type polar amino acid transport system ATP	0	NA
NC_01685(	RefSeq	CDS	1736036	1736716	+	0	ID=cds1915	0	0,5	STM14_1980	STM14_1980	putative ABC-type transport system membrane componer	0	NA
NC_01685(	RefSeq	CDS	1736853	1738403	+	0	ID=cds1916	0	2	STM14_1981	STM14_1981	putative inner membrane protein	0,284200331	-1,182723423
NC_01685(	RefSeq	CDS	1738867	1739529	+	0	ID=cds1917	0	9	STM14_1982	STM14_1982	putative SAM-dependent methyltransferase	1,27890149	-2,207741391
NC_01685(	RefSeq	CDS	1739800	1740330	-	0	ID=cds1918	0	8	STM14_1983	cybB	cytochrome b561	1,136801324	-2,147403465
NC_01685(	RefSeq	CDS	1740447	1741247	-	0	ID=cds1919	0	14	STM14_1984	ydcF	putative inner membrane protein	1,989402318	-2,376535557
NC_01685(	RefSeq	CDS	1741311	1745156	-	0	ID=cds1920	0	80,5	STM14_1985	hrpA	ATP-dependent RNA helicase HrpA	11,36801324	-1,993013165
NC_01685(	RefSeq	CDS	1745414	1746019	+	0	ID=cds1921	0	1	STM14_1986	acpD	azoreductase	0,142100166	-0,623264422
NC_01685(	RefSeq	CDS	1746016	1746465	-	0	ID=cds1922	0	6	STM14_1987	STM14_1987	putative inner membrane protein	0,852600993	-1,98056697
NC_01685(	RefSeq	CDS	1746608	1746931	-	0	ID=cds1923	0	3	STM14_1988	ydbL	hypothetical protein	0,426300497	-1,498211557
NC_01685(	RefSeq	CDS	1746939	1747130	-	0	ID=cds1924	0	1	STM14_1989	ynbE	putative outer membrane lipoprotein	0,142100166	-0,623264422
NC_01685(	RefSeq	CDS	1747130	1749766	-	0	ID=cds1925	0	19	STM14_1990	ydbH	hypothetical protein	2,699903145	-2,42419778
NC_01685(	RefSeq	CDS	1750006	1750995	+	0	ID=cds1926	0	6	STM14_1991	ldhA	D-lactate dehydrogenase	0,852600993	-1,98056697
NC_01685(	RefSeq	CDS	1751106	1751516	+	0	ID=cds1927	0,5	3	STM14_1992	hslJ	heat-inducible protein	0,426300497	-1,498211557
NC_01685(	RefSeq	CDS	1751560	1751826	-	0	ID=cds1928	0	4,75	STM14_1993	STM14_1993	putative cytoplasmic protein	0,710500828	-1,864806942
NC_01685(	RefSeq	CDS	1751742	1752041	+	0	ID=cds1929	0	1	STM14_1994	STM14_1994	hypothetical protein	0,142100166	-0,623264422
NC_01685(	RefSeq	CDS	1752102	1755626	+	0	ID=cds1930	3	19	STM14_1995	nifJ	putative pyruvate-flavodoxin oxidoreductase	7,977870255	0,635561419
NC_01685(	RefSeq	CDS	1755685	1755894	-	0	ID=cds1931	0	0	STM14_1996	STM14_1996	hypothetical protein	0	NA
NC_01685(	RefSeq	CDS	1756108	1756542	+	0	ID=cds1932	0	104	STM14_1997	ynaF	putative universal stress protein	14,77841722	-1,890380653
NC_01685(	RefSeq	CDS	1756592	1756930	-	0	ID=cds1933	0	5	STM14_1998	STM14_1998	putative membrane transporter of cations	0,710500828	-1,864806942
NC_01685(	RefSeq	CDS	1757094	1757234	+	0	ID=cds1934	0	6	STM14_1999	STM14_1999	Rac prophage%3B integrase	0,852600993	-1,98056697
NC_01685(	RefSeq	CDS	1757286	1758221	+	0	ID=cds1935	0	16,5	STM14_2000	ydaO	C32 tRNA thiolase	2,273602649	-2,403729808
NC_01685(	RefSeq	CDS	1758265	1759638	-	0	ID=cds1936	0	3,5	STM14_2001	dbpA	ATP-dependent RNA helicase DbpA	0,568400662	-1,710438693
NC_01685(	RefSeq	CDS	1760125	1761108	-	0	ID=cds1937	0	11	STM14_2002	zntB	zinc transporter	1,563101821	-2,297202414
NC_01685(	RefSeq	CDS	1761254	1762408	-	0	ID=cds1938	0	0,5	STM14_2003	STM14_2003	putative methyl-accepting chemotaxis protein	0	NA
NC_01685(	RefSeq	CDS	1762419	1762580	-	0	ID=cds1939	0	0	STM14_2004	STM14_2004	hypothetical protein	0	NA
NC_01685(	RefSeq	CDS	1762819	1763382	-	0	ID=cds1940	0	4	STM14_2005	ydaL	hypothetical protein	0,568400662	-1,710438693
NC_01685(	RefSeq	CDS	1763640	1764155	+	0	ID=cds1941	0	20,5	STM14_2006	ogt	O-6-alkylguanine-DNA:cysteine-protein methyltransferase	2,842003311	-2,427810709
NC_01685(	RefSeq	CDS	1764351	1765103	+	0	ID=cds1942	1	45,5	STM14_2007	fnr	fumarate%2Fnitrate reduction transcriptional regulator	8,295929985	-1,195723789
NC_01685(	RefSeq	CDS	1765254	1766201	+	0	ID=cds1943	0	58,5	STM14_2008	ydaA	universal stress protein UspE	8,241809601	-2,117369507
NC_01685(	RefSeq	CDS	1766256	1766510	-	0	ID=cds1944	0	7	STM14_2009	ynaJ	putative inner membrane protein	0,994701159	-2,072978628
NC_01685(	RefSeq	CDS	1766749	1767780	+	0	ID=cds1945	0	3	STM14_2010	ynaI	putative integral membrane protein	0,426300497	-1,498211557
NC_01685(	RefSeq	CDS	1767866	1768468	-	0	ID=cds1946	0	3	STM14_2011	STM14_2011	putative transcriptional regulator	0,426300497	-1,498211557
NC_01685(	RefSeq	CDS	1768512	1769198	+	0	ID=cds1947	0	1	STM14_2012	STM14_2012	putative cytoplasmic protein	0,142100166	-0,623264422
NC_01685(	RefSeq	CDS	1769670	1770227	-	0	ID=cds1948	0	0	STM14_2014	STM14_2014	putative thiol peroxidase	0	NA
NC_01685(	RefSeq	CDS	1770326	1771156	-	0	ID=cds1949	0	1	STM14_2015	STM14_2015	hypothetical protein	0,142100166	-0,623264422
NC_01685(	RefSeq	CDS	1771177	1773159	-	0	ID=cds1950	0	1,5	STM14_2016	STM14_2016	invasin-like protein	0,284200331	-1,182723423
NC_01685(	RefSeq	CDS	1773234	1774079	-	0	ID=cds1951	0	0	STM14_2017	STM14_2017	putative lipoprotein	0	NA
NC_01685(	RefSeq	CDS	1775622	1776731	+	0	ID=cds1952	2	39	STM14_2019	STM14_2019	hypothetical protein	9,060551196	-0,431862456
NC_01685(	RefSeq	CDS	1776945	1777181	-	0	ID=cds1953	0	1	STM14_2020	STM14_2020	putative outer membrane lipoprotein	0,142100166	-0,623264422
NC_01685(	RefSeq	CDS	1777310	1777900	-	0	ID=cds1954	0	2	STM14_2021	STM14_2021	putative regulatory protein	0,284200331	-1,182723423
NC_01685(	RefSeq	CDS	1777978	1778691	+	0	ID=cds1955	0	3	STM14_2022	STM14_2022	oxidoreductase	0,426300497	-1,498211557
NC_01685(	RefSeq	CDS	1778782	1779651	-	0	ID=cds1956	0	1	STM14_2023	STM14_2023	putative aldo%2Fketo reductase	0,142100166	-0,623264422
NC_01685(	RefSeq	CDS	1779789	1779902	-	0	ID=cds1957	0	0,5	STM14_2024	STM14_2024	hypothetical protein	0	NA
NC_01685(	RefSeq	CDS	1779925	1780830	-	0	ID=cds1958	0	2,5	STM14_2025	STM14_2025	putative transcriptional regulator	0,284200331	-1,182723423
NC_01685(	RefSeq	CDS	1780949	1781878	+	0	ID=cds1959	0	0	STM14_2026	STM14_2026	putative 2%27-hydroxyisoflavone reductase	0	NA
NC_01685(	RefSeq	CDS	1781975	1783588	-	0	ID=cds1960	0	36	STM14_2027	mppA	periplasmic murein tripeptide transport protein	5,115605959	-2,281753063
NC_01685(	RefSeq	CDS	1783778	1784506	+	0	ID=cds1961	0	5	STM14_2028	ycjI	murein peptide amidase A	0,710500828	-1,864806942
NC_01685(	RefSeq	CDS	1784481	1785446	-	0	ID=cds1962	5	6,5	STM14_2029	ycjG	putative chloromuconate cycloisomerase	9,649212843	2,164558341
NC_01685(	RefSeq	CDS	1785562	1786068	+	0	ID=cds1963	0	30	STM14_2030	tpx	thiol peroxidase	4,263004966	-2,464060063
NC_01685(	RefSeq	CDS	1786154	1786342	+	0	ID=cds1964	0	1,1	STM14_2031	STM14_2031	hypothetical protein	0,142100166	-0,623264422
NC_01685(	RefSeq	CDS	1786299	1786757	+	0	ID=cds1965	0	2,9	STM14_2032	tnpA_5	transposase	0,426300497	-1,498211557
NC_01685(	RefSeq	CDS	1786870	1788411	-	0	ID=cds1966	0	18,5	STM14_2033	tyrR	DNA-binding transcriptional regulator TyrR	2,55780298	-2,419166771
NC_01685(	RefSeq	CDS	1788559	1789620	-	0	ID=cds1967	0	24,83333333	STM14_2034	ycjF	hypothetical protein	3,552504138	-2,43600272
NC_01685(	RefSeq	CDS	1789617	1791020	-	0	ID=cds1968	0	28,5	STM14_2035	ycjX	putative ATPase	3,978804635	-2,448045729
NC_01685(	RefSeq	CDS	1791163	1791477	-	0	ID=cds1969	0	19,5	STM14_2036	pspE	thiosulfate:cyanide sulfurtransferase	2,842003311	-2,427810709
NC_01685(	RefSeq	CDS	1791553	1791771	-	0	ID=cds1970	0	0	STM14_2037	pspD	peripheral inner membrane phage-shock protein	0	NA
NC_01685(	RefSeq	CDS	1791794	1792153	-	0	ID=cds1971	0	2	STM14_2038	pspC	DNA-binding transcriptional activator PspC	0,284200331	-1,182723423
NC_01685(	RefSeq	CDS	1792153	1792377	-	0	ID=cds1972	0	0	STM14_2039	pspB	phage shock protein B	0	NA
NC_01685(	RefSeq	CDS	1792437	1793105	-	0	ID=cds1973	1	4	STM14_2040	pspA	phage shock protein PspA	2,327723032	1,125268706
NC_01685(	RefSeq	CDS	1793276	1794256	+	0	ID=cds1974	0	1	STM14_2041	pspF	phage shock protein operon transcriptional activator	0,142100166	-0,623264422
NC_01685(	RefSeq	CDS	1794368	1796017	+	0	ID=cds1975	1	22,75	STM14_2042	sapA	peptide transport protein	5,027626177	-0,555407513
NC_01685(	RefSeq	CDS	1796014	1796979	+	0	ID=cds1976	0	8,25	STM14_2043	sapB	peptide transport protein	1,136801324	-2,147403465
NC_01685(	RefSeq	CDS	1796966	1797856	+	0	ID=cds1977	0	10	STM14_2044	sapC	peptide transport protein	1,421001655	-2,256999172
NC_01685(	RefSeq	CDS	1797856	1798848	+	0	ID=cds1978	0	18	STM14_2045	sapD	peptide transport protein	2,55780298	-2,419166771
NC_01685(	RefSeq	CDS	1798850	1799656	+	0	ID=cds1979	0	6	STM14_2046	sapF	peptide transport protein	0,852600993	-1,98056697
NC_01685(	RefSeq	CDS	1799672	1800379	-	0	ID=cds1980	0	18,5	STM14_2047	STM14_2047	hypothetical protein	2,55780298	-2,419166771
NC_01685(	RefSeq	CDS	1800491	1800637	-	0	ID=cds1981	0	0,5	STM14_2048	STM14_2048	hypothetical protein	0	NA
NC_01685(	RefSeq	CDS	1800647	1800769	+	0	ID=cds1982	0	0	STM14_2049	STM14_2049	hypothetical protein	0	NA
NC_01685(	RefSeq	CDS	1800846	1802219	-	0	ID=cds1983	0	7	STM14_2050	STM14_2050	putative inner membrane protein	0,994701159	-2,072978628
NC_01685(	RefSeq	CDS	1802254	1802433	+	0	ID=cds1984	0	0	STM14_2051	STM14_2051	hypothetical protein	0	NA
NC_01685(	RefSeq	CDS	1802408	1802707	+	0	ID=cds1985	0	2	STM14_2052	ycjE	putative cytoplasmic protein	0,284200331	-1,182723423
NC_01685(	RefSeq	CDS	1802825	1803613	+	0	ID=cds1986	0	72,5	STM14_2053	fabI	enoyl-ACP reductase	10,23121192	-2,035392961
NC_01685(	RefSeq	CDS	1803682	1803798	-	0	ID=cds1987	0	0	STM14_2054	STM14_2054	hypothetical protein	0	NA
NC_01685(	RefSeq	CDS	1803813	1804946	+	0	ID=cds1988	0	4,5	STM14_2055	yciW	putative cytoplasmic protein	0,568400662	-1,710438693
NC_01685(	RefSeq	CDS	1805035	1806969	+	0	ID=cds1989	0	38	STM14_2056	rnb	exoribonuclease II	5,39980629	-2,264331336
NC_01685(	RefSeq	CDS	1807079	1807216	+	0	ID=cds1990	0	1	STM14_2057	STM14_2057	hypothetical protein	0,142100166	-0,623264422
NC_01685(	RefSeq	CDS	1807231	1809213	+	0	ID=cds1991	0	3,5	STM14_2058	yciR	RNase II stability modulator	0,568400662	-1,710438693
NC_01685(	RefSeq	CDS	1809302	1809514	+	0	ID=cds1992	0	6	STM14_2059	STM14_2059	hypothetical protein	0,852600993	-1,98056697
NC_01685(	RefSeq	CDS	1809601	1810353	+	0	ID=cds1993	0	17,5	STM14_2060	yciT	putative regulatory protein	2,55780298	-2,419166771
NC_01685(	RefSeq	CDS	1810467	1810628	-	0	ID=cds1994	0	0	STM14_2061	STM14_2061	hypothetical protein	0	NA
NC_01685(	RefSeq	CDS	1810612	1810830	+	0	ID=cds1995	0	2	STM14_2062	osmB	lipoprotein	0,284200331	-1,182723423
NC_01685(	RefSeq	CDS	1810950	1811276	-	0	ID=cds1996	0	2	STM14_2063	yciH	translation initiation factor Sui1	0,284200331	-1,182723423
NC_01685(	RefSeq	CDS	1811276	1812013	-	0	ID=cds1997	0	0,5	STM14_2064	pyrF	orotidine 5%27-phosphate decarboxylase	0	NA
NC_01685(	RefSeq	CDS	1812029	1812142	+	0	ID=cds1998	0	0	STM14_2065	STM14_2065	hypothetical protein	0	NA
NC_01685(	RefSeq	CDS	1812204	1813373	-	0	ID=cds1999	0	17,5	STM14_2066	yciM	hypothetical protein	2,55780298	-2,419166771
NC_01685(	RefSeq	CDS	1813380	1813688	-	0	ID=cds2000	0	22	STM14_2067	yciS	putative inner membrane protein	3,126203642	-2,431590384
NC_01685(	RefSeq	CDS	1813838	1814602	-	0	ID=cds2001	0	7,5	STM14_2068	pgpB	phosphatidylglycerophosphatase B	1,136801324	-2,147403465

NC_01685(	RefSeq	CDS	1814798	1815388	+	0	ID=cds2002	0	20	STM14_2069	ribA	GTP cyclohydrolase II	2,842003311	-2,427810709
NC_01685(	RefSeq	CDS	1815444	1818119	-	0	ID=cds2003	1	21,83333333	STM14_2070	acnA	aconitate hydratase	4,885526012	-0,515045314
NC_01685(	RefSeq	CDS	1818504	1818674	-	0	ID=cds2004	1	6,5	STM14_2071	STM14_2071	hypothetical protein	2,611923363	0,709005733
NC_01685(	RefSeq	CDS	1819096	1820070	-	0	ID=cds2005	1	10,5	STM14_2072	cysB	transcriptional regulator CysB	3,180324025	0,201203536
NC_01685(	RefSeq	CDS	1820026	1820190	+	0	ID=cds2006	0	0	STM14_2073	STM14_2073	hypothetical protein	0	NA
NC_01685(	RefSeq	CDS	1820225	1820401	-	0	ID=cds2007	0	0	STM14_2074	STM14_2074	hypothetical protein	0	NA
NC_01685(	RefSeq	CDS	1820481	1823078	-	0	ID=cds2008	0	55	STM14_2075	topA	DNA topoisomerase I	7,815509104	-2,137668811
NC_01685(	RefSeq	CDS	1823233	1823364	-	0	ID=cds2009	0	9	STM14_2076	STM14_2076	hypothetical protein	1,27890149	-2,207741391
NC_01685(	RefSeq	CDS	1823481	1823732	+	0	ID=cds2010	0	21,5	STM14_2077	yciN	hypothetical protein	3,126203642	-2,431590384
NC_01685(	RefSeq	CDS	1823774	1824820	-	0	ID=cds2011	0	24	STM14_2078	sohB	putative periplasmic protease	3,410403973	-2,434086198
NC_01685(	RefSeq	CDS	1824904	1825014	+	0	ID=cds2012	0	0	STM14_2079	STM14_2079	hypothetical protein	0	NA
NC_01685(	RefSeq	CDS	1825058	1825819	+	0	ID=cds2013	0	4	STM14_2080	yciK	short chain dehydrogenase	0,568400662	-1,710438693
NC_01685(	RefSeq	CDS	1825816	1826406	+	0	ID=cds2014	0	9	STM14_2081	btuR	cob%28I%29yrinic acid a%2Cc-diamide adenosyltransferas	1,27890149	-2,207741391
NC_01685(	RefSeq	CDS	1826493	1827368	-	0	ID=cds2015	0	22	STM14_2082	yciL	23S rRNA pseudouridylate synthase B	3,126203642	-2,431590384
NC_01685(	RefSeq	CDS	1828097	1828978	-	0	ID=cds2016	0	31	STM14_2084	trpH	hypothetical protein	4,405105132	-2,475774913
NC_01685(	RefSeq	CDS	1829100	1829144	+	0	ID=cds2017	0	0	STM14_2085	trpL	trp operon leader peptide	0	NA
NC_01685(	RefSeq	CDS	1829239	1830801	+	0	ID=cds2018	0	0	STM14_2086	trpE	anthranilate synthase component I	0	NA
NC_01685(	RefSeq	CDS	1830801	1832396	+	0	ID=cds2019	0	2,5	STM14_2087	trpD	bifunctional anthranilate synthase%2Fanthranilate phospl	0,284200331	-1,182723423
NC_01685(	RefSeq	CDS	1832400	1833758	+	0	ID=cds2020	0	7,5	STM14_2088	trpC	bifunctional indole-3-glycerol phosphate synthase%2Fphc	1,136801324	-2,147403465
NC_01685(	RefSeq	CDS	1833768	1834961	+	0	ID=cds2021	0	1	STM14_2089	trpB	tryptophan synthase subunit beta	0,142100166	-0,623264422
NC_01685(	RefSeq	CDS	1834961	1835767	+	0	ID=cds2022	0	3,5	STM14_2090	trpA	tryptophan synthase subunit alpha	0,568400662	-1,710438693
NC_01685(	RefSeq	CDS	1836247	1836429	+	0	ID=cds2023	0	0	STM14_2091	yciG	putative cytoplasmic protein	0	NA
NC_01685(	RefSeq	CDS	1836519	1837022	+	0	ID=cds2024	0	0	STM14_2092	yciF	putative cytoplasmic protein	0	NA
NC_01685(	RefSeq	CDS	1837096	1837602	+	0	ID=cds2025	0	2	STM14_2093	yciE	putative cytoplasmic protein	0,284200331	-1,182723423
NC_01685(	RefSeq	CDS	1837622	1838500	+	0	ID=cds2026	0	0	STM14_2094	STM14_2094	putative catalase	0	NA
NC_01685(	RefSeq	CDS	1838582	1839220	-	0	ID=cds2027	0	725,5	STM14_2095	ompW	outer membrane protein W	103,1647202	-1,96077624
NC_01685(	RefSeq	CDS	1839558	1839962	+	0	ID=cds2028	0	4	STM14_2096	STM14_2096	putative ferredoxin	0,568400662	-1,710438693
NC_01685(	RefSeq	CDS	1839988	1840731	+	0	ID=cds2029	2	35	STM14_2097	yciC	hypothetical protein	8,492150534	-0,327624837
NC_01685(	RefSeq	CDS	1840788	1841327	+	0	ID=cds2030	0	26,5	STM14_2098	yciB	intracellular septation protein A	3,694604304	-2,438835558
NC_01685(	RefSeq	CDS	1841488	1841889	+	0	ID=cds2031	0	3	STM14_2099	yciA	acyl-CoA thioester hydrolase	0,426300497	-1,498211557
NC_01685(	RefSeq	CDS	1841949	1842686	-	0	ID=cds2032	0	4	STM14_2100	tonB	transporter	0,568400662	-1,710438693
NC_01685(	RefSeq	CDS	1842901	1843197	+	0	ID=cds2033	0	41,5	STM14_2101	ycil	YciI-like protein	5,968206953	-2,232890319
NC_01685(	RefSeq	CDS	1843241	1843414	-	0	ID=cds2034	3	25	STM14_2102	STM14_2102	hypothetical protein	8,830471248	0,379046434
NC_01685(	RefSeq	CDS	1843557	1845017	+	0	ID=cds2035	0	3	STM14_2103	cls	cardiolipin synthetase	0,426300497	-1,498211557
NC_01685(	RefSeq	CDS	1845051	1845380	+	0	ID=cds2036	0	19	STM14_2104	yciU	dsDNA-mimic protein	2,699903145	-2,42419778
NC_01685(	RefSeq	CDS	1845428	1846264	-	0	ID=cds2037	0	2	STM14_2105	STM14_2105	putative voltage-gated potassium channel	0,284200331	-1,182723423
NC_01685(	RefSeq	CDS	1846312	1847316	-	0	ID=cds2038	0	28,5	STM14_2106	oppF	oligopeptide transport protein	3,978804635	-2,448045729
NC_01685(	RefSeq	CDS	1847313	1848320	-	0	ID=cds2039	0	39,5	STM14_2107	oppD	oligopeptide ABC transporter ATP-binding protein	5,684006621	-2,24950002
NC_01685(	RefSeq	CDS	1848332	1849240	-	0	ID=cds2040	0	23	STM14_2108	oppC	oligopeptide transport protein	3,268303807	-2,432747446
NC_01685(	RefSeq	CDS	1849255	1850175	-	0	ID=cds2041	0	9,5	STM14_2109	oppB	oligopeptide transporter permease	1,421001655	-2,256999172
NC_01685(	RefSeq	CDS	1850297	1851928	-	0	ID=cds2042	0	36,5	STM14_2110	oppA	oligopeptide transport protein	5,115605959	-2,281753063
NC_01685(	RefSeq	CDS	1851996	1852310	+	0	ID=cds2043	0	1	STM14_2111	STM14_2111	putative inner membrane protein	0,142100166	-0,623264422
NC_01685(	RefSeq	CDS	1852693	1853340	-	0	ID=cds2044	0	2	STM14_2112	ychE	hypothetical protein	0,284200331	-1,182723423
NC_01685(	RefSeq	CDS	1853817	1856495	+	0	ID=cds2045	1	118	STM14_2113	adhE	bifunctional acetaldehyde-CoA%2Falcohol dehydrogenase	18,5271419	-2,10956331
NC_01685(	RefSeq	CDS	1856692	1857309	-	0	ID=cds2046	0	10	STM14_2114	tdk_2	thymidine kinase	1,421001655	-2,256999172
NC_01685(	RefSeq	CDS	1857497	1857622	+	0	ID=cds2047	0	0	STM14_2115	STM14_2115	hypothetical protein	0	NA
NC_01685(	RefSeq	CDS	1857972	1858385	+	0	ID=cds2048	4,5	213,5	STM14_2116	hns	global DNA-binding transcriptional dual regulator H-NS	37,4467249	-1,449417711
NC_01685(	RefSeq	CDS	1858398	1858580	+	0	ID=cds2049	0,5	0	STM14_2117	STM14_2117	hypothetical protein	0	NA
NC_01685(	RefSeq	CDS	1858517	1859425	-	0	ID=cds2050	0	44,5	STM14_2118	galU	UTP--glucose-1-phosphate uridylyltransferase subunit Gal	6,252407284	-2,216733793
NC_01685(	RefSeq	CDS	1859628	1860641	-	0	ID=cds2051	0	26,5	STM14_2119	hnr	response regulator of RpoS	3,694604304	-2,438835558
NC_01685(	RefSeq	CDS	1860732	1861637	-	0	ID=cds2052	0	26,5	STM14_2120	ychK	hypothetical protein	3,694604304	-2,438835558
NC_01685(	RefSeq	CDS	1861748	1862206	+	0	ID=cds2053	0	4	STM14_2121	ychJ	hypothetical protein	0,568400662	-1,710438693
NC_01685(	RefSeq	CDS	1862257	1863099	+	0	ID=cds2054	0	30	STM14_2122	purU	formyltetrahydrofolate deformylase	4,263004966	-2,464060063
NC_01685(	RefSeq	CDS	1863160	1863279	-	0	ID=cds2055	0	0	STM14_2123	STM14_2123	hypothetical protein	0	NA
NC_01685(	RefSeq	ncRNA	1863372	1863514	+	.	ID=rna32;F	0	3,5	STM14_2125	rtT	regulatory RNA	0,568400662	-1,710438693
NC_01685(	RefSeq	CDS	1864415	1865944	-	0	ID=cds2056	0	4	STM14_2127	STM14_2127	hypothetical protein	0,568400662	-1,710438693
NC_01685(	RefSeq	CDS	1865980	1866144	+	0	ID=cds2057	0	0,5	STM14_2128	STM14_2128	hypothetical protein	0	NA
NC_01685(	RefSeq	CDS	1866186	1866863	-	0	ID=cds2058	0	2	STM14_2129	narI	nitrate reductase 1 subunit gamma	0,284200331	-1,182723423
NC_01685(	RefSeq	CDS	1866863	1867573	-	0	ID=cds2059	0	3,5	STM14_2130	narJ	nitrate reductase 1 subunit delta	0,568400662	-1,710438693
NC_01685(	RefSeq	CDS	1867570	1869105	-	0	ID=cds2060	0	4,5	STM14_2131	narH	nitrate reductase 1 subunit beta	0,568400662	-1,710438693
NC_01685(	RefSeq	CDS	1869102	1872845	-	0	ID=cds2061	0	10,5	STM14_2132	narG	nitrate reductase 1 subunit alpha	1,421001655	-2,256999172
NC_01685(	RefSeq	CDS	1872924	1873043	+	0	ID=cds2062	0	0	STM14_2133	STM14_2133	hypothetical protein	0	NA
NC_01685(	RefSeq	CDS	1873233	1874630	-	0	ID=cds2063	1	1	STM14_2134	narK	nitrite extrusion protein	1,901422535	2,462014545
NC_01685(	RefSeq	CDS	1874718	1874903	-	0	ID=cds2064	0	0	STM14_2135	STM14_2135	hypothetical protein	0	NA
NC_01685(	RefSeq	CDS	1874971	1876767	+	0	ID=cds2065	0	8,5	STM14_2136	narX	nitrate%2Fnitrite sensor protein NarX	1,136801324	-2,147403465
NC_01685(	RefSeq	CDS	1876760	1877410	+	0	ID=cds2066	0	2	STM14_2137	narL	transcriptional regulator NarL	0,284200331	-1,182723423
NC_01685(	RefSeq	CDS	1877411	1878835	-	0	ID=cds2067	0	20,5	STM14_2138	ychP	hypothetical protein	2,842003311	-2,427810709
NC_01685(	RefSeq	CDS	1879012	1879365	+	0	ID=cds2068	0	8	STM14_2139	ychN	putative sulfur reduction protein	1,136801324	-2,147403465
NC_01685(	RefSeq	CDS	1879445	1879675	-	0	ID=cds2069	0	1	STM14_2140	chaB	cation transport regulator	0,142100166	-0,623264422
NC_01685(	RefSeq	CDS	1879942	1881042	+	0	ID=cds2070	0	6	STM14_2141	chaA	calcium%2Fsodium:proton antiporter	0,852600993	-1,98056697
NC_01685(	RefSeq	CDS	1881096	1881950	-	0	ID=cds2071	0	29,5	STM14_2142	kdsA	2-dehydro-3-deoxyphosphooctonate aldolase	4,263004966	-2,464060063
NC_01685(	RefSeq	CDS	1881988	1882797	-	0	ID=cds2072	0	29,5	STM14_2143	ychA	putative transcriptional regulator	4,263004966	-2,464060063
NC_01685(	RefSeq	CDS	1882801	1883190	-	0	ID=cds2073	0	9,5	STM14_2144	sirC	putative transcriptional regulator	1,421001655	-2,256999172
NC_01685(	RefSeq	CDS	1883187	1884020	-	0	ID=cds2074	0	22	STM14_2145	hemK	N5-glutamine S-adenosyl-L-methionine-dependent methy	3,126203642	-2,431590384
NC_01685(	RefSeq	CDS	1884020	1885102	-	0	ID=cds2075	0	52	STM14_2146	prfA	peptide chain release factor 1	7,389208608	-2,158807911
NC_01685(	RefSeq	CDS	1885143	1886399	-	0	ID=cds2076	0	72	STM14_2147	hemA	glutamyl-tRNA reductase	10,23121192	-2,035392961
NC_01685(	RefSeq	CDS	1886567	1886695	-	0	ID=cds2077	0	0	STM14_2148	STM14_2148	hypothetical protein	0	NA
NC_01685(	RefSeq	CDS	1886713	1887336	+	0	ID=cds2078	0	17,83333333	STM14_2149	lolB	outer membrane lipoprotein LolB	2,55780298	-2,419166771
NC_01685(	RefSeq	CDS	1887333	1888184	+	0	ID=cds2079	1	31,75	STM14_2150	ipk	4-diphosphocytidyl-2-C-methyl-D-erythritol kinase	6,306527667	-0,86024894
NC_01685(	RefSeq	CDS	1888181	1888351	-	0	ID=cds2080	0	0	STM14_2151	STM14_2151	hypothetical protein	0	NA
NC_01685(	RefSeq	CDS	1888329	1888433	-	0	ID=cds2081	0	0	STM14_2152	STM14_2152	hypothetical protein	0	NA
NC_01685(	RefSeq	CDS	1888450	1889397	+	0	ID=cds2082	0	164	STM14_2153	prsA	ribose-phosphate pyrophosphokinase	23,30442715	-1,884236327
NC_01685(	RefSeq	CDS	1889522	1891207	+	0	ID=cds2083	1	17	STM14_2154	ychM	putative sulfate transporter YchM	4,175025184	-0,285403406
NC_01685(	RefSeq	CDS	1891252	1891530	-	0	ID=cds2084	0	44,5	STM14_2155	ychH	hypothetical protein	6,252407284	-2,216733793
NC_01685(	RefSeq	CDS	1891806	1892390	+	0	ID=cds2085	0	7,5	STM14_2156	pth	peptidyl-tRNA hydrolase	1,136801324	-2,147403465
NC_01685(	RefSeq	CDS	1892507	1893598	+	0	ID=cds2086	0	30	STM14_2157	ychF	translation-associated GTPase	4,263004966	-2,464060063
NC_01685(	RefSeq	CDS	1893588	1893701	-	0	ID=cds2087	0	0	STM14_2158	STM14_2158	hypothetical protein	0	NA
NC_01685(	RefSeq	CDS	1893757	1895022	-	0	ID=cds2088	0	19,5	STM14_2159	STM14_2159	putative cytoplasmic protein	2,842003311	-2,427810709
NC_01685(	RefSeq	CDS	1895293	1895421	-	0	ID=cds2089	0	0	STM14_2160	STM14_2160	hypothetical protein	0	NA
NC_01685(	RefSeq	CDS	1895517	1896635	+	0	ID=cds2090	0	2	STM14_2161	STM14_2161	hydrogenase-1 small subunit	0,284200331	-1,182723423
NC_01685(	RefSeq	CDS	1896632	1898425	+	0	ID=cds2091	0	17	STM14_2162	STM14_2162	hydrogenase 1 large subunit	2,415702814	-2,412509737
NC_01685(	RefSeq	CDS	1898444	1899175	+	0	ID=cds2092	0	5,5	STM14_2163	STM14_2163	hydrogenase 1 b-type cytochrome subunit	0,852600993	-1,98056697
NC_01685(	RefSeq	CDS	1899172	1899768	+	0	ID=cds2093	0	2,5	STM14_2164	STM14_2164	hydrogenase 1 maturation protease	0,284200331	-1,182723423
NC_01685(	RefSeq	CDS	1899758	1900162	+	0	ID=cds2094	0	1	STM14_2165	STM14_2165	hydrogenase-1 operon protein HyaE	0,142100166	-0,623264422
NC_01685(	RefSeq	CDS	1900159	1901007	+	0	ID=cds2095	0	2	STM14_2166	STM14_2166	putative hydrogenase-1 protein	0,284200331	-1,182723423
NC_01685(	RefSeq	CDS	1901082	1902626	+	0	ID=cds2096	0	4,5	STM14_2167	STM14_2167	putative cytochrome oxidase subunit I	0,568400662	-1,710438693
NC_01685(	RefSeq	CDS	1902638	1903774	+	0	ID=cds2097	0	1,5	STM14_2168	STM14_2168	putative cytochrome oxidase subunit II	0,284200331	-1,182723423
NC_01685(	RefSeq	CDS	1903787	1903876	+	0	ID=cds2098	0	1	STM14_2169	STM14_2169	hypothetical protein	0,142100166	-0,623264422
NC_01685(	RefSeq	CDS	1904220	1905545	+	0	ID=cds2099	1	34,5	STM14_2170	STM14_2170	glutamate dehydrogenase-like protein	6,590727998	-0,915216377
NC_01685(	RefSeq	CDS	1905555	1905662	-	0	ID=cds2100	0	0	STM14_2171	STM14_2171	hypothetical protein	0	NA
NC_01685(	RefSeq	CDS	1905759	1907471	+	0	ID=cds2101	0	5	STM14_2172	treA	trehalase	0,710500828	-1,864806942
NC_01685(	RefSeq	CDS	1907534	1907788	-	0	ID=cds2102	0	0	STM14_2173	ymgE	putative transglycosylase-associated protein	0	NA
NC_01685(	RefSeq	CDS	1907957	1908691	+	0	ID=cds2103	0	1,5	STM14_2174	ycgR	putative inner membrane protein	0,284200331	-1,182723423
NC_01685(	RefSeq	CDS	1908705	1909316	-	0	ID=cds2104	0	4,5	STM14_2175	emtA	membrane-bound lytic murein transglycosylase E	0,568400662	-1,710438693
NC_01685(	RefSeq	CDS	1909487	1910401	+	0	ID=cds2105	0	4,5	STM14_2176	ldcA	L%2CD-carboxypeptidase A	0,568400662	-1,710438693
NC_01685(	RefSeq	CDS	1910498	1912231	+	0	ID=cds2106	0	38	STM14_2177	ycgO	potassium%2Fproton antiporter	5,39980629	-2,264331336
NC_01685(	RefSeq	CDS	1912294	1913364	-	0	ID=cds2107	0	0	STM14_2178	dadX	alanine racemase	0	NA
NC_01685(	RefSeq	CDS	1913378	1914676	-	0	ID=cds2108	0	0	STM14_2179	dadA	D-amino acid dehydrogenase small subunit	0	NA
NC_01685(	RefSeq	CDS	1914824	1914934	-	0	ID=cds2109	0	0,5	STM14_2180	STM14_2180	hypothetical protein	0	NA
NC_01685(	RefSeq	CDS	1915005	1916531	+	0	ID=cds2110	0	7	STM14_2181	ycgB	hypothetical protein	0,994701159	-2,072978628
NC_01685(	RefSeq	CDS	1916578	1917297	-	0	ID=cds2111	0	49,5	STM14_2182	fadR	fatty acid metabolism regulator	7,105008277	-2,173389122
NC_01685(	RefSeq	CDS	1917517	1919061	+	0	ID=cds2112	0	16	STM14_2183	nhaB	sodium%2Fproton antiporter	2,273602649	-2,403729808
NC_01685(	RefSeq	CDS	1919203	1919733	+	0	ID=cds2113	0	18,5	STM14_2184	dsbB	disulfide bond formation protein B	2,55780298	-2,419166771
NC_01685(	RefSeq	CDS	1919842	1920183	+	0	ID=cds2114	0	0	STM14_2185	STM14_2185	putative cytoplasmic protein	0	NA
NC_01685(	RefSeq	CDS	1920255	1920428	-	0	ID=cds2115	0	4	STM14_2186	STM14_2186	putative cytoplasmic protein	0,568400662	-1,710438693
NC_01685(	RefSeq	CDS	1920441	1920557	+	0	ID=cds2116	0	0	STM14_2187	STM14_2187	hypothetical protein	0	NA
NC_01685(	RefSeq	CDS	1920620	1920757	-	0	ID=cds2117	0	2	STM14_2188	STM14_2188	putative cytoplasmic protein	0,284200331	-1,182723423
NC_01685(	RefSeq	CDS	1920801	1920899	-	0	ID=cds2118	0	0	STM14_2189	STM14_2189	hypothetical protein	0	NA
NC_01685(	RefSeq	CDS	1921109	1921555	-	0	ID=cds2119	0	20	STM14_2190	ycgN	hypothetical protein	2,842003311	-2,427810709
NC_01685(	RefSeq	CDS	1921648	1922307	-	0	ID=cds2120	0	25	STM14_2191	ycgM	hypothetical protein	3,552504138	-2,43600272
NC_01685(	RefSeq	CDS	1922357	1922689	-	0	ID=cds2121	0	18,5	STM14_2192	ycgL	putative cytoplasmic protein	2,55780298	-2,419166771
NC_01685(	RefSeq	CDS	1922776	1923483	+	0	ID=cds2122	0	63	STM14_2193	minC	septum formation inhibitor	8,952310429	-2,085292348
NC_01685(	RefSeq	CDS	1923507	1924319	+	0	ID=cds2123	0	88,5	STM14_2194	minD	cell division inhibitor MinD	12,50481457	-1,954585237
NC_01685(	RefSeq	CDS	1924323	1924589	+	0	ID=cds2124	0	11	STM14_2195	minE	cell division topological specificity factor MinE	1,563101821	-2,297202414

NC_01685(	RefSeq	CDS	1924711	1925838	-	0	ID=cds2125	0	5	STM14_2196	rnd	ribonuclease D	0,710500828	-1,864806942
NC_01685(	RefSeq	CDS	1925975	1926433	-	0	ID=cds2126	0	3,4	STM14_2197	tnpA_6	transposase	0,426300497	-1,498211557
NC_01685(	RefSeq	CDS	1926390	1926578	-	0	ID=cds2127	0	1,1	STM14_2198	STM14_2198	hypothetical protein	0,142100166	-0,623264422
NC_01685(	RefSeq	CDS	1926624	1928309	-	0	ID=cds2128	0	18,5	STM14_2199	fadD	long-chain-fatty-acid--CoA ligase	2,55780298	-2,419166771
NC_01685(	RefSeq	CDS	1928329	1928535	+	0	ID=cds2129	0	0	STM14_2200	STM14_2200	hypothetical protein	0	NA
NC_01685(	RefSeq	CDS	1928514	1929095	-	0	ID=cds2130	3	10,5	STM14_2201	slp	putative outer membrane protein	6,698968765	1,219842116
NC_01685(	RefSeq	CDS	1929167	1929862	-	0	ID=cds2131	0	35,5	STM14_2202	yeaZ	putative molecular chaperone	5,115605959	-2,281753063
NC_01685(	RefSeq	CDS	1929920	1931830	-	0	ID=cds2132	0	18,5	STM14_2203	yoaA	putative DNA helicase	2,55780298	-2,419166771
NC_01685(	RefSeq	CDS	1931961	1932305	+	0	ID=cds2133	0	41	STM14_2204	yoaB	putative translation initiation inhibitor	5,826106787	-2,241137745
NC_01685(	RefSeq	CDS	1932311	1932490	-	0	ID=cds2134	0	3,5	STM14_2205	yoaH	hypothetical protein	0,568400662	-1,710438693
NC_01685(	RefSeq	CDS	1932571	1933935	+	0	ID=cds2135	0	21,25	STM14_2206	pabB	para-aminobenzoate synthase component I	2,984103476	-2,430148754
NC_01685(	RefSeq	CDS	1933939	1934517	+	0	ID=cds2136	0	2,75	STM14_2207	yeaB	hypothetical protein	0,426300497	-1,498211557
NC_01685(	RefSeq	CDS	1934781	1936145	+	0	ID=cds2137	0	54	STM14_2208	sdaA	L-serine deaminase I%2FL-threonine deaminase I	7,673408939	-2,144619715
NC_01685(	RefSeq	CDS	1936283	1937884	+	0	ID=cds2138	2	3	STM14_2209	STM14_2209	diguanylate cyclase	3,944945236	1,874572685
NC_01685(	RefSeq	CDS	1937906	1939465	-	0	ID=cds2139	0	4	STM14_2210	yoaE	putative inner membrane protein	0,568400662	-1,710438693
NC_01685(	RefSeq	CDS	1939453	1939788	-	0	ID=cds2140	0	0,5	STM14_2211	STM14_2211	putative cytoplasmic protein	0	NA
NC_01685(	RefSeq	CDS	1939938	1940906	+	0	ID=cds2141	0	125,5	STM14_2212	manX	mannose-specific enzyme IIAB	17,90462086	-1,85836405
NC_01685(	RefSeq	CDS	1940959	1941759	+	0	ID=cds2142	0	151,5	STM14_2213	manY	mannose-specific enzyme IIC	21,59922516	-1,877281771
NC_01685(	RefSeq	CDS	1941763	1942623	+	0	ID=cds2143	0	297	STM14_2214	manZ	mannose-specific PTS system protein IID	42,20374916	-1,926475259
NC_01685(	RefSeq	CDS	1942681	1943139	+	0	ID=cds2144	0	22,5	STM14_2215	STM14_2215	hypothetical protein	3,126203642	-2,431590384
NC_01685(	RefSeq	CDS	1943495	1944115	+	0	ID=cds2145	0	8	STM14_2216	yebN	hypothetical protein	1,136801324	-2,147403465
NC_01685(	RefSeq	CDS	1944112	1944921	-	0	ID=cds2146	1	5	STM14_2217	rrmA	23S rRNA methyltransferase A	2,469823198	0,89567218
NC_01685(	RefSeq	CDS	1944987	1946732	-	0	ID=cds2147	0	23	STM14_2218	STM14_2218	putative penicillin-binding protein 3	3,268303807	-2,432747446
NC_01685(	RefSeq	CDS	1946749	1946859	-	0	ID=cds2148	0	2,5	STM14_2219	STM14_2219	hypothetical protein	0,284200331	-1,182723423
NC_01685(	RefSeq	CDS	1946952	1947161	-	0	ID=cds2149	0	91,08333333	STM14_2220	cspC	cold shock-like protein CspC	12,93111506	-1,941086641
NC_01685(	RefSeq	CDS	1947174	1947317	-	0	ID=cds2150	0	108,25	STM14_2221	yobF	putative cytoplasmic protein	15,34681788	-1,876042944
NC_01685(	RefSeq	CDS	1947434	1947541	+	0	ID=cds2151	0	0	STM14_2222	STM14_2222	hypothetical protein	0	NA
NC_01685(	RefSeq	CDS	1947556	1947696	+	0	ID=cds2152	0	0	STM14_2223	STM14_2223	hypothetical protein	0	NA
NC_01685(	RefSeq	CDS	1947624	1947740	-	0	ID=cds2153	0	3	STM14_2224	STM14_2224	hypothetical protein	0,426300497	-1,498211557
NC_01685(	RefSeq	CDS	1947966	1948253	-	0	ID=cds2154	4	33	STM14_2225	STM14_2225	hypothetical protein	11,72659494	0,399502809
NC_01685(	RefSeq	CDS	1948324	1948467	-	0	ID=cds2155	2	43,5	STM14_2226	yobG	hypothetical protein	9,771052023	-0,549311495
NC_01685(	RefSeq	CDS	1948625	1948864	+	0	ID=cds2156	0	0	STM14_2227	STM14_2227	hypothetical protein	0	NA
NC_01685(	RefSeq	CDS	1949076	1949867	-	0	ID=cds2157	0	15,5	STM14_2228	kdgR	putative transcriptional repressor	2,273602649	-2,403729808
NC_01685(	RefSeq	CDS	1949871	1949978	-	0	ID=cds2158	0	0,5	STM14_2229	STM14_2229	hypothetical protein	0	NA
NC_01685(	RefSeq	CDS	1950043	1951416	+	0	ID=cds2159	0	3,5	STM14_2230	STM14_2230	putative transport protein	0,568400662	-1,710438693
NC_01685(	RefSeq	CDS	1951464	1952345	-	0	ID=cds2160	0	17,5	STM14_2231	htpX	heat shock protein HtpX	2,55780298	-2,419166771
NC_01685(	RefSeq	CDS	1952539	1954587	-	0	ID=cds2161	0	116	STM14_2232	prc	carboxy-terminal protease	16,4836192	-1,849193185
NC_01685(	RefSeq	CDS	1954607	1955293	-	0	ID=cds2162	1	49	STM14_2233	proQ	putative solute%2FDNA competence effector	8,722230481	-1,256284002
NC_01685(	RefSeq	CDS	1955391	1955975	-	0	ID=cds2163	1	15	STM14_2234	yebR	putative nucleotide-binding protein	3,890824853	-0,174265561
NC_01685(	RefSeq	CDS	1956017	1957300	+	0	ID=cds2164	0	12,33333333	STM14_2235	yebS	putative inner membrane protein	1,705201986	-2,329925387
NC_01685(	RefSeq	CDS	1957263	1959902	+	0	ID=cds2165	0	11	STM14_2236	STM14_2236	putative inner membrane protein	1,563101821	-2,297202414
NC_01685(	RefSeq	CDS	1959980	1961419	+	0	ID=cds2166	0	6,5	STM14_2237	yebU	rRNA %28cytosine-C%285%29-%29-methyltransferase Rsr	0,852600993	-1,98056697
NC_01685(	RefSeq	CDS	1961391	1961522	+	0	ID=cds2167	0	0	STM14_2238	STM14_2238	hypothetical protein	0	NA
NC_01685(	RefSeq	CDS	1961534	1961773	+	0	ID=cds2168	0	1	STM14_2239	STM14_2239	putative cytoplasmic protein	0,142100166	-0,623264422
NC_01685(	RefSeq	CDS	1961884	1962075	+	0	ID=cds2169	0	0	STM14_2240	yebW	putative inner membrane lipoprotein	0	NA
NC_01685(	RefSeq	CDS	1962094	1962762	-	0	ID=cds2170	0	0,5	STM14_2241	pphA	serine%2Fthreonine protein phosphatase 1	0	NA
NC_01685(	RefSeq	CDS	1962968	1963132	-	0	ID=cds2171	3,5	2	STM14_2242	STM14_2242	putative inner membrane protein	7,321489811	2,765358925
NC_01685(	RefSeq	CDS	1963183	1963311	+	0	ID=cds2172	0	0	STM14_2243	STM14_2243	hypothetical protein	0	NA
NC_01685(	RefSeq	CDS	1963417	1964139	-	0	ID=cds2173	0	26	STM14_2244	sopE2	type III-secreted effector protein	3,694604304	-2,438835558
NC_01685(	RefSeq	CDS	1964429	1964551	+	0	ID=cds2174	0	0	STM14_2245	STM14_2245	hypothetical protein	0	NA
NC_01685(	RefSeq	CDS	1964644	1964787	-	0	ID=cds2175	0	0	STM14_2246	STM14_2246	hypothetical protein	0	NA
NC_01685(	RefSeq	CDS	1964823	1965218	+	0	ID=cds2176	0	3	STM14_2247	STM14_2247	putative cytoplasmic protein	0,426300497	-1,498211557
NC_01685(	RefSeq	CDS	1965548	1966024	+	0	ID=cds2177	0	9	STM14_2248	STM14_2248	hypothetical protein	1,27890149	-2,207741391
NC_01685(	RefSeq	CDS	1966412	1966831	-	0	ID=cds2178	0	0,5	STM14_2249	STM14_2249	putative acetyltransferase	0	NA
NC_01685(	RefSeq	CDS	1966960	1967154	-	0	ID=cds2179	0	0	STM14_2250	STM14_2250	hypothetical protein	0	NA
NC_01685(	RefSeq	CDS	1967201	1967470	+	0	ID=cds2180	0	0	STM14_2251	STM14_2251	putative cytoplasmic protein	0	NA
NC_01685(	RefSeq	CDS	1967603	1967776	+	0	ID=cds2181	0	0	STM14_2252	STM14_2252	putative cytoplasmic protein	0	NA
NC_01685(	RefSeq	CDS	1968259	1969116	-	0	ID=cds2182	0	0	STM14_06230	STM14_06230	hypothetical protein	0	NA
NC_01685(	RefSeq	CDS	1969279	1969608	+	0	ID=cds2183	0	1	STM14_2254	STM14_2254	hypothetical protein	0,142100166	-0,623264422
NC_01685(	RefSeq	CDS	1970094	1970294	-	0	ID=cds2184	0	0	STM14_2255	STM14_2255	hypothetical protein	0	NA
NC_01685(	RefSeq	CDS	1970912	1971826	+	0	ID=cds2185	0	1,5	STM14_2256	pagO	integral membrane protein	0,284200331	-1,182723423
NC_01685(	RefSeq	CDS	1971959	1972117	+	0	ID=cds2186	0	0,5	STM14_2257	STM14_2257	putative inner membrane protein	0	NA
NC_01685(	RefSeq	CDS	1972127	1972741	+	0	ID=cds2187	0	0	STM14_2258	STM14_2258	putative inner membrane protein	0	NA
NC_01685(	RefSeq	CDS	1973190	1973366	-	0	ID=cds2188	0	0	STM14_2260	STM14_2260	hypothetical protein	0	NA
NC_01685(	RefSeq	CDS	1973229	1973375	+	0	ID=cds2189	0	0	STM14_2261	STM14_2261	hypothetical protein	0	NA
NC_01685(	RefSeq	CDS	1973981	1974151	-	0	ID=cds2190	0	0	STM14_2263	STM14_2263	hypothetical protein	0	NA
NC_01685(	RefSeq	CDS	1974277	1974393	+	0	ID=cds2191	0	0	STM14_2264	STM14_2264	tail fiber assembly protein	0	NA
NC_01685(	RefSeq	CDS	1974584	1974784	+	0	ID=cds2192	3	6,5	STM14_2265	pagK	PagK	6,130568103	1,664704357
NC_01685(	RefSeq	CDS	1974881	1975939	-	0	ID=cds2193	0	0	STM14_2266	mig-3	phage side tail fiber assembly protein	0	NA
NC_01685(	RefSeq	CDS	1975917	1976180	-	0	ID=cds2194	0	1	STM14_2267	STM14_2267	hypothetical protein	0,142100166	-0,623264422
NC_01685(	RefSeq	CDS	1976235	1976423	+	0	ID=cds2195	0	0	STM14_2268	STM14_2268	hypothetical protein	0	NA
NC_01685(	RefSeq	CDS	1976488	1976655	+	0	ID=cds2196	0	0	STM14_2269	STM14_2269	lytic enzyme	0	NA
NC_01685(	RefSeq	CDS	1976705	1976878	+	0	ID=cds2197	0	0,833333333	STM14_2270	STM14_2270	hypothetical protein	0,142100166	-0,623264422
NC_01685(	RefSeq	CDS	1976808	1976927	+	0	ID=cds2198	0	0,833333333	STM14_2271	STM14_2271	hypothetical protein	0,142100166	-0,623264422
NC_01685(	RefSeq	CDS	1976912	1977445	-	0	ID=cds2199	0	1	STM14_2272	STM14_2272	phage-tail assembly-like protein	0,142100166	-0,623264422
NC_01685(	RefSeq	CDS	1977499	1977786	-	0	ID=cds2200	0	0	STM14_2273	STM14_2273	hypothetical protein	0	NA
NC_01685(	RefSeq	CDS	1977760	1978413	+	0	ID=cds2201	0	0,5	STM14_2274	STM14_2274	RecE-like protein	0	NA
NC_01685(	RefSeq	CDS	1978431	1979327	+	0	ID=cds2202	0	0	STM14_2275	STM14_2275	integrase	0	NA
NC_01685(	RefSeq	CDS	1979312	1979440	-	0	ID=cds2203	0	0	STM14_2276	STM14_2276	hypothetical protein	0	NA
NC_01685(	RefSeq	CDS	1979524	1979643	-	0	ID=cds2204	2	8,5	STM14_2277	STM14_2277	putative cytoplasmic protein	4,655446064	1,025807982
NC_01685(	RefSeq	CDS	1979701	1980054	-	0	ID=cds2205	0	5	STM14_2278	STM14_2278	hypothetical protein	0,710500828	-1,864806942
NC_01685(	RefSeq	CDS	1980071	1980946	-	0	ID=cds2206	0	21	STM14_2279	STM14_2279	putative inner membrane protein	2,984103476	-2,430148754
NC_01685(	RefSeq	CDS	1980947	1981321	-	0	ID=cds2207	0	19,5	STM14_2280	yobA	hypothetical protein	2,842003311	-2,427810709
NC_01685(	RefSeq	CDS	1981459	1981689	+	0	ID=cds2208	0	5	STM14_2281	holE	DNA polymerase III subunit theta	0,710500828	-1,864806942
NC_01685(	RefSeq	CDS	1981797	1982453	+	0	ID=cds2209	0	5,5	STM14_2282	STM14_2282	putative amidohydrolase	0,852600993	-1,98056697
NC_01685(	RefSeq	CDS	1982477	1983175	+	0	ID=cds2210	0	11	STM14_2283	exoX	exodeoxyribonuclease X	1,563101821	-2,297202414
NC_01685(	RefSeq	CDS	1983210	1985261	-	0	ID=cds2211	0	9,333333333	STM14_2284	ptrB	protease 2	1,27890149	-2,207741391
NC_01685(	RefSeq	CDS	1985474	1986133	-	0	ID=cds2212	4	12	STM14_2285	yebE	putative inner membrane protein	8,742491466	1,340204343
NC_01685(	RefSeq	CDS	1986227	1986580	-	0	ID=cds2213	0	11,5	STM14_2286	yebF	hypothetical protein	1,705201986	-2,329925387
NC_01685(	RefSeq	CDS	1986648	1986938	-	0	ID=cds2214	0	4	STM14_2287	yebG	DNA damage-inducible protein YebG	0,568400662	-1,710438693
NC_01685(	RefSeq	CDS	1987069	1988247	+	0	ID=cds2215	0	4,5	STM14_2288	purT	phosphoribosylglycinamide formyltransferase 2	0,568400662	-1,710438693
NC_01685(	RefSeq	CDS	1988347	1988988	-	0	ID=cds2216	0	15	STM14_2289	eda	keto-hydroxyglutarate-aldolase%2Fketo-deoxy- phosphog	2,131502483	-2,392032191
NC_01685(	RefSeq	CDS	1989026	1990837	-	0	ID=cds2217	0	13,5	STM14_2290	edd	phosphogluconate dehydratase	1,989402318	-2,376535557
NC_01685(	RefSeq	CDS	1990827	1990985	-	0	ID=cds2218	0	0	STM14_2291	STM14_2291	hypothetical protein	0	NA
NC_01685(	RefSeq	CDS	1991072	1992547	-	0	ID=cds2219	0	23	STM14_2292	zwf	glucose-6-phosphate 1-dehydrogenase	3,268303807	-2,432747446
NC_01685(	RefSeq	CDS	1992662	1992787	+	0	ID=cds2220	0	0	STM14_2293	STM14_2293	hypothetical protein	0	NA
NC_01685(	RefSeq	CDS	1992755	1992874	-	0	ID=cds2221	0	0	STM14_2294	STM14_2294	hypothetical protein	0	NA
NC_01685(	RefSeq	CDS	1992889	1993758	+	0	ID=cds2222	0	9	STM14_2295	yebK	DNA-binding transcriptional regulator HexR	1,27890149	-2,207741391
NC_01685(	RefSeq	CDS	1993882	1995324	+	0	ID=cds2223	0	115,5	STM14_2296	pykA	pyruvate kinase	16,4836192	-1,849193185
NC_01685(	RefSeq	CDS	1995397	1996368	-	0	ID=cds2224	0	23	STM14_2297	msbB	lipid A biosynthesis %28KDO%292-%28lauroyl%29-lipid IV	3,268303807	-2,432747446
NC_01685(	RefSeq	CDS	1996367	1996594	+	0	ID=cds2225	0	0	STM14_2298	STM14_2298	hypothetical protein	0	NA
NC_01685(	RefSeq	CDS	1996485	1997804	-	0	ID=cds2226	0	59	STM14_2299	yebA	hypothetical protein	8,383909767	-2,110782333
NC_01685(	RefSeq	CDS	1997820	1998764	-	0	ID=cds2227	0	33	STM14_2300	znuA	high-affinity zinc transporter periplasmic protein	4,689305463	-2,505858154
NC_01685(	RefSeq	CDS	1998843	1999598	+	0	ID=cds2228	0	2	STM14_2301	znuC	high-affinity zinc transporter ATPase	0,284200331	-1,182723423
NC_01685(	RefSeq	CDS	1999595	2000380	+	0	ID=cds2229	0	12,33333333	STM14_2302	znuB	high-affinity zinc transporter membrane protein	1,705201986	-2,329925387
NC_01685(	RefSeq	CDS	2000459	2001469	-	0	ID=cds2230	0	20	STM14_2303	ruvB	Holliday junction DNA helicase B	2,842003311	-2,427810709
NC_01685(	RefSeq	CDS	2001478	2002089	-	0	ID=cds2231	2	16	STM14_2304	ruvA	Holliday junction DNA helicase motor protein	5,792247388	0,405774795
NC_01685(	RefSeq	CDS	2002147	2002269	+	0	ID=cds2232	0	0	STM14_2305	STM14_2305	hypothetical protein	0	NA
NC_01685(	RefSeq	CDS	2002264	2002389	-	0	ID=cds2233	0	0	STM14_2306	STM14_2306	hypothetical protein	0	NA
NC_01685(	RefSeq	CDS	2004031	2004630	+	0	ID=cds2234	0	0	STM14_2308	yebB	hypothetical protein	0	NA
NC_01685(	RefSeq	CDS	2004632	2005153	-	0	ID=cds2235	1	14	STM14_2309	ruvC	Holliday junction resolvase	3,748724687	-0,112256387
NC_01685(	RefSeq	CDS	2005190	2005930	-	0	ID=cds2236	1	42,5	STM14_2310	yebC	hypothetical protein	7,727529322	-1,109707982
NC_01685(	RefSeq	CDS	2005960	2006412	-	0	ID=cds2237	0	9,5	STM14_2311	ntpA	dATP pyrophosphohydrolase	1,421001655	-2,256999172
NC_01685(	RefSeq	CDS	2006515	2008287	-	0	ID=cds2238	3	98,5	STM14_2312	aspS	aspartyl-tRNA synthetase	19,20378333	-0,964875477
NC_01685(	RefSeq	CDS	2008446	2008562	+	0	ID=cds2239	0	0	STM14_2313	STM14_2313	hypothetical protein	0	NA
NC_01685(	RefSeq	CDS	2008612	2009178	+	0	ID=cds2240	0	3	STM14_2314	yecD	hypothetical protein	0,426300497	-1,498211557
NC_01685(	RefSeq	CDS	2009175	2009993	+	0	ID=cds2241	0	3	STM14_2315	yecE	hypothetical protein	0,426300497	-1,498211557
NC_01685(	RefSeq	CDS	2010046	2010441	+	0	ID=cds2242	0	2	STM14_2316	yecN	putative inner membrane protein	0,284200331	-1,182723423
NC_01685(	RefSeq	CDS	2010482	2011225	+	0	ID=cds2243	0	23	STM14_2317	yecO	putative SAM-dependent methyltransferase	3,268303807	-2,432747446
NC_01685(	RefSeq	CDS	2011222	2012193	+	0	ID=cds2244	0	8,5	STM14_2318	yecP	hypothetical protein	1,136801324	-2,147403465
NC_01685(	RefSeq	CDS	2012429	2013175	-	0	ID=cds2245	4	23	STM14_2319	cutC	copper homeostasis protein CutC	10,30559329	0,74414677
NC_01685(	RefSeq	CDS	2013195	2013764	-	0	ID=cds2246	0	2	STM14_2320	yecM	hypothetical protein	0,284200331	-1,182723423
NC_01685(	RefSeq	CDS	2013869	2013985	+	0	ID=cds2247	0	0	STM14_2321	STM14_2321	hypothetical protein	0	NA
NC_01685(	RefSeq	CDS	2014001	2015734	+	0	ID=cds2248	0	58	STM14_2322	argS	arginyl-tRNA synthetase	8,241809601	-2,117369507

NC_016856	RefSeq	CDS	2015793	2015897	+		0	ID=cds2249	0	2,5	STM14_2323	STM14_2323	hypothetical protein	0,284200331	-1,182723423
NC_016856	RefSeq	CDS	2015923	2017794	+		0	ID=cds2250	0	6	STM14_2324	STM14_2324	putative penicillin-binding protein	0,852600993	-1,98056697
NC_016856	RefSeq	CDS	2017848	2018987	-		0	ID=cds2251	0	4	STM14_2325	STM14_2325	putative cytoplasmic protein	0,568400662	-1,710438693
NC_016856	RefSeq	CDS	2019224	2019616	-		0	ID=cds2252	0	3	STM14_2326	flhE	flagellar protein	0,426300497	-1,498211557
NC_016856	RefSeq	CDS	2019616	2021694	-		0	ID=cds2253	0	7,5	STM14_2327	flhA	flagellar biosynthesis protein FlhA	1,136801324	-2,147403465
NC_016856	RefSeq	CDS	2021687	2022838	-		0	ID=cds2254	0	2	STM14_2328	flhB	flagellar biosynthesis protein FlhB	0,284200331	-1,182723423
NC_016856	RefSeq	CDS	2022930	2023187	+		0	ID=cds2255	0	0	STM14_2329	STM14_2329	hypothetical protein	0	NA
NC_016856	RefSeq	CDS	2023032	2023676	-		0	ID=cds2256	0,5	16,25	STM14_2330	cheZ	chemotaxis regulator CheZ	2,273602649	-2,403729808
NC_016856	RefSeq	CDS	2023687	2024076	-		0	ID=cds2257	0	24,25	STM14_2331	cheY	chemotaxis regulatory protein CheY	3,410403973	-2,434086198
NC_016856	RefSeq	CDS	2024094	2025143	-		0	ID=cds2258	0	17,75	STM14_2332	cheB	chemotaxis-specific methylesterase	2,55780298	-2,419166771
NC_016856	RefSeq	CDS	2025140	2026006	-		0	ID=cds2259	0	26	STM14_2333	cheR	chemotaxis methyltransferase CheR	3,694604304	-2,438835558
NC_016856	RefSeq	CDS	2026160	2027821	-		0	ID=cds2260	0	35,5	STM14_2334	cheM	methyl accepting chemotaxis protein II	5,115605959	-2,281753063
NC_016856	RefSeq	CDS	2028058	2028561	-		0	ID=cds2261	0	22,5	STM14_2335	cheW	purine-binding chemotaxis protein	3,126203642	-2,431590384
NC_016856	RefSeq	CDS	2028582	2030597	-		0	ID=cds2262	0	80,5	STM14_2336	cheA	chemotaxis protein CheA	11,36801324	-1,993013165
NC_016856	RefSeq	CDS	2030602	2031531	-		0	ID=cds2263	0	27,5	STM14_2337	motB	flagellar motor protein MotB	3,978804635	-2,448045729
NC_016856	RefSeq	CDS	2031528	2032415	-		0	ID=cds2264	0	23,5	STM14_2338	motA	flagellar motor protein MotA	3,410403973	-2,434086198
NC_016856	RefSeq	CDS	2032400	2032531	-		0	ID=cds2265	0	4,5	STM14_2339	STM14_2339	hypothetical protein	0,568400662	-1,710438693
NC_016856	RefSeq	CDS	2032540	2033118	-		0	ID=cds2266	0	39,5	STM14_2340	flhC	transcriptional activator FlhC	5,684006621	-2,24950002
NC_016856	RefSeq	CDS	2033121	2033471	-		0	ID=cds2267	0	28,33333333	STM14_2341	flhD	transcriptional activator FlhD	3,978804635	-2,448045729
NC_016856	RefSeq	CDS	2033684	2033827	-		0	ID=cds2268	0	36,5	STM14_2342	STM14_2342	hypothetical protein	5,115605959	-2,281753063
NC_016856	RefSeq	CDS	2034145	2034261	+		0	ID=cds2269	0	1	STM14_2343	STM14_2343	hypothetical protein	0,142100166	-0,623264422
NC_016856	RefSeq	CDS	2034258	2034686	+		0	ID=cds2270	0	10	STM14_2344	yecG	universal stress protein UspC	1,421001655	-2,256999172
NC_016856	RefSeq	CDS	2034704	2036125	-		0	ID=cds2271	0	5,5	STM14_2345	otsA	trehalose-6-phosphate synthase	0,852600993	-1,98056697
NC_016856	RefSeq	CDS	2036100	2036903	-		0	ID=cds2272	0	0	STM14_2346	otsB	trehalose-6-phosphate phosphatase	0	NA
NC_016856	RefSeq	CDS	2037537	2038094	-		0	ID=cds2273	0	4,5	STM14_2348	araH	putative intracellular protease%2Famidase	0,568400662	-1,710438693
NC_016856	RefSeq	CDS	2038156	2038305	+		0	ID=cds2274	0	0	STM14_2349	STM14_2349	putative cytoplasmic protein	0	NA
NC_016856	RefSeq	CDS	2038645	2039148	+		0	ID=cds2275	0	2	STM14_2350	ftnB	ferritin-like protein	0,284200331	-1,182723423
NC_016856	RefSeq	CDS	2039327	2039965	-		0	ID=cds2276	0	3	STM14_2351	STM14_2351	hypothetical protein	0,426300497	-1,498211557
NC_016856	RefSeq	CDS	2040221	2040556	+		0	ID=cds2277	0	3	STM14_2352	STM14_2352	putative outer membrane lipoprotein	0,426300497	-1,498211557
NC_016856	RefSeq	CDS	2040796	2041293	+		0	ID=cds2278	0	18	STM14_2353	ftn	ferritin	2,55780298	-2,419166771
NC_016856	RefSeq	CDS	2041339	2041578	-		0	ID=cds2279	0	7	STM14_2354	yecH	putative cytoplasmic protein	0,994701159	-2,072978628
NC_016856	RefSeq	CDS	2041805	2043016	+		0	ID=cds2280	0	6	STM14_2355	tyrP	tyrosine-specific transport protein	0,852600993	-1,98056697
NC_016856	RefSeq	CDS	2043091	2043756	-		0	ID=cds2281	0	57,5	STM14_2356	yecA	hypothetical protein	8,241809601	-2,117369507
NC_016856	RefSeq	CDS	2043994	2044320	-		0	ID=cds2282	0	9,5	STM14_2357	STM14_2357	putative glucose-6-phosphate dehydrogenase	1,421001655	-2,256999172
NC_016856	RefSeq	CDS	2044533	2045972	-		0	ID=cds2283	0	14,5	STM14_2358	STM14_2358	putative cell wall-associated hydrolase	1,989402318	-2,376535557
NC_016856	RefSeq	CDS	2046185	2046517	-		0	ID=cds2284	0	2	STM14_2359	STM14_2359	putative inner membrane protein	0,284200331	-1,182723423
NC_016856	RefSeq	CDS	2047218	2047766	-		0	ID=cds2285	0	15	STM14_2363	pgsA	phosphatidylglycerophosphate synthetase	2,131502483	-2,392032191
NC_016856	RefSeq	CDS	2047823	2049655	-		0	ID=cds2286	3	47,5	STM14_2364	uvrC	excinuclease ABC subunit C	12,09877506	-0,252043845
NC_016856	RefSeq	CDS	2049652	2050308	-		0	ID=cds2287	0	24,5	STM14_2365	uvrY	response regulator	3,410403973	-2,434086198
NC_016856	RefSeq	CDS	2050620	2050775	+		0	ID=cds2288	0	0,5	STM14_2366	STM14_2366	putative inner membrane protein	0	NA
NC_016856	RefSeq	CDS	2050772	2050996	+		0	ID=cds2289	0	1,5	STM14_2367	yecF	hypothetical protein	0,284200331	-1,182723423
NC_016856	RefSeq	CDS	2051063	2051785	-		0	ID=cds2290	0	20	STM14_2368	sdiA	DNA-binding transcriptional activator SdiA	2,842003311	-2,427810709
NC_016856	RefSeq	CDS	2052017	2052769	-		0	ID=cds2291	0	9,5	STM14_2369	yecC	putative amino-acid ABC transporter ATP-binding protein	1,421001655	-2,256999172
NC_016856	RefSeq	CDS	2052766	2053434	-		0	ID=cds2292	0	6	STM14_2370	yecS	putative ABC-type amino acid transporter permease comp	0,852600993	-1,98056697
NC_016856	RefSeq	CDS	2053455	2054441	-		0	ID=cds2293	0	9,5	STM14_2371	yedO	D-cysteine desulfhydrase	1,421001655	-2,256999172
NC_016856	RefSeq	CDS	2054595	2055395	-		0	ID=cds2294	0	21,5	STM14_2372	fliY	cystine transporter subunit	3,126203642	-2,431590384
NC_016856	RefSeq	CDS	2055542	2056093	-		0	ID=cds2295	0	18	STM14_2373	fliZ	protein FliZ	2,55780298	-2,419166771
NC_016856	RefSeq	CDS	2056152	2056871	-		0	ID=cds2296	0	35	STM14_2374	fliA	flagellar biosynthesis sigma factor	4,973505794	-2,290637485
NC_016856	RefSeq	CDS	2057140	2057649	-		0	ID=cds2297	0	3,25	STM14_2375	tnpA_7	transposase for IS200	0,426300497	-1,498211557
NC_016856	RefSeq	CDS	2057555	2057743	-		0	ID=cds2298	0	0,75	STM14_2376	STM14_2376	hypothetical protein	0,142100166	-0,623264422
NC_016856	RefSeq	CDS	2057779	2058984	-		0	ID=cds2299	0	17,5	STM14_2377	fliB	lysine-N-methylase	2,55780298	-2,419166771
NC_016856	RefSeq	CDS	2059063	2060550	-		0	ID=cds2300	2	571,5	STM14_2378	fliC	flagellin	84,79993943	-2,990117206
NC_016856	RefSeq	CDS	2060568	2060705	-		0	ID=cds2301	0	8	STM14_2379	STM14_2379	hypothetical protein	1,136801324	-2,147403465
NC_016856	RefSeq	CDS	2060807	2062210	+		0	ID=cds2302	0	38,5	STM14_2380	fliD	flagellar capping protein	5,39980629	-2,264331336
NC_016856	RefSeq	CDS	2062225	2062632	+		0	ID=cds2303	0	6,5	STM14_2381	fliS	flagellar protein FliS	0,852600993	-1,98056697
NC_016856	RefSeq	CDS	2062632	2063000	+		0	ID=cds2304	0	20	STM14_2382	fliT	flagellar biosynthesis protein FliT	2,842003311	-2,427810709
NC_016856	RefSeq	CDS	2063072	2064556	+		0	ID=cds2305	0	5	STM14_2383	amyA	alpha-amylase	0,710500828	-1,864806942
NC_016856	RefSeq	CDS	2064596	2065021	-		0	ID=cds2306	6	8	STM14_2384	yedD	hypothetical protein	11,69273554	2,11628868
NC_016856	RefSeq	CDS	2065147	2066412	+		0	ID=cds2307	0	10,5	STM14_2385	yedE	putative inner membrane protein	1,421001655	-2,256999172
NC_016856	RefSeq	CDS	2066409	2066642	+		0	ID=cds2308	0	2	STM14_2386	yedF	hypothetical protein	0,284200331	-1,182723423
NC_016856	RefSeq	CDS	2066907	2067293	+		0	ID=cds2309	0	2	STM14_2387	STM14_2387	putative 50S ribosomal protein	0,284200331	-1,182723423
NC_016856	RefSeq	CDS	2067413	2067727	-		0	ID=cds2310	0	2,5	STM14_2388	fliE	flagellar hook-basal body protein FliE	0,284200331	-1,182723423
NC_016856	RefSeq	CDS	2067774	2067902	-		0	ID=cds2311	0	0	STM14_2389	STM14_2389	hypothetical protein	0	NA
NC_016856	RefSeq	CDS	2067944	2069626	+		0	ID=cds2312	0	40,83333333	STM14_2390	fliF	flagellar MS-ring protein	5,826106787	-2,241137745
NC_016856	RefSeq	CDS	2069619	2070614	+		0	ID=cds2313	0	14	STM14_2391	fliG	flagellar motor switch protein G	1,989402318	-2,376535557
NC_016856	RefSeq	CDS	2070607	2071314	+		0	ID=cds2314	0	7,5	STM14_2392	fliH	flagellar assembly protein H	1,136801324	-2,147403465
NC_016856	RefSeq	CDS	2071314	2072684	+		0	ID=cds2315	0	13,5	STM14_2393	fliI	flagellum-specific ATP synthase	1,989402318	-2,376535557
NC_016856	RefSeq	CDS	2072706	2073149	+		0	ID=cds2316	0	4,5	STM14_2394	fliJ	flagellar biosynthesis chaperone	0,568400662	-1,710438693
NC_016856	RefSeq	CDS	2073146	2074363	+		0	ID=cds2317	0	6	STM14_2395	fliK	flagellar hook-length control protein	0,852600993	-1,98056697
NC_016856	RefSeq	CDS	2074468	2074935	+		0	ID=cds2318	0	4,5	STM14_2396	fliL	flagellar basal body-associated protein FliL	0,568400662	-1,710438693
NC_016856	RefSeq	CDS	2074940	2075944	+		0	ID=cds2319	0	13	STM14_2397	fliM	flagellar motor switch protein FliM	1,847302152	-2,356210635
NC_016856	RefSeq	CDS	2075941	2076354	+		0	ID=cds2320	0	7,5	STM14_2398	fliN	flagellar motor switch protein FliN	1,136801324	-2,147403465
NC_016856	RefSeq	CDS	2076354	2076731	+		0	ID=cds2321	0	1,75	STM14_2399	fliO	flagellar biosynthesis protein FliO	0,284200331	-1,182723423
NC_016856	RefSeq	CDS	2076731	2077468	+		0	ID=cds2322	0	4,25	STM14_2400	fliP	flagellar biosynthesis protein FliP	0,568400662	-1,710438693
NC_016856	RefSeq	CDS	2077478	2077747	+		0	ID=cds2323	0	1	STM14_2401	fliQ	flagellar biosynthesis protein FliQ	0,142100166	-0,623264422
NC_016856	RefSeq	CDS	2077756	2078550	+		0	ID=cds2324	0	0	STM14_2402	fliR	flagellar biosynthesis protein FliR	0	NA
NC_016856	RefSeq	CDS	2078832	2079455	+		0	ID=cds2325	0	1	STM14_2403	rcsA	colanic acid capsular biosynthesis activation protein A	0,142100166	-0,623264422
NC_016856	RefSeq	CDS	2079494	2079688	-		0	ID=cds2326	0	6,5	STM14_2404	dsrB	hypothetical protein	0,852600993	-1,98056697
NC_016856	RefSeq	CDS	2079817	2080044	+		0	ID=cds2327	0	0	STM14_2405	yodD	putative cytoplasmic protein	0	NA
NC_016856	RefSeq	ncRNA	2080055	2080144	-	.		ID=rna37;P	0	0	STM14_2406	dsrA	small regulatory RNA DsrA	0	NA
NC_016856	RefSeq	CDS	2080354	2081169	+		0	ID=cds2328	0	0,5	STM14_2407	yedP	mannosyl-3-phosphoglycerate phosphatase	0	NA
NC_016856	RefSeq	CDS	2081148	2082860	-		0	ID=cds2329	0	3	STM14_2408	STM14_2408	putative inner membrane protein	0,426300497	-1,498211557
NC_016856	RefSeq	CDS	2083025	2083210	-		0	ID=cds2330	0	4	STM14_2409	STM14_2409	putative cytoplasmic protein	0,568400662	-1,710438693
NC_016856	RefSeq	CDS	2083287	2084198	-		0	ID=cds2331	0	6	STM14_2410	yedI	hypothetical protein	0,852600993	-1,98056697
NC_016856	RefSeq	CDS	2084253	2084468	+		0	ID=cds2332	0	0	STM14_2411	STM14_2411	hypothetical protein	0	NA
NC_016856	RefSeq	CDS	2084374	2085294	+		0	ID=cds2333	0	4,5	STM14_2412	yedA	hypothetical protein	0,568400662	-1,710438693
NC_016856	RefSeq	CDS	2085283	2085753	-		0	ID=cds2334	0	3	STM14_2413	vsr	DNA mismatch endonuclease	0,426300497	-1,498211557
NC_016856	RefSeq	CDS	2085734	2087164	-		0	ID=cds2335	0	10	STM14_2414	dcm	DNA cytosine methylase	1,421001655	-2,256999172
NC_016856	RefSeq	CDS	2087238	2087933	-		0	ID=cds2336	0	2	STM14_2415	yedJ	hypothetical protein	0,284200331	-1,182723423
NC_016856	RefSeq	CDS	2088025	2088324	-		0	ID=cds2337	0	0	STM14_2416	STM14_2416	putative inner membrane protein	0	NA
NC_016856	RefSeq	CDS	2088843	2088962	+		0	ID=cds2338	0	0	STM14_2417	STM14_2417	hypothetical protein	0	NA
NC_016856	RefSeq	CDS	2088974	2090170	+		0	ID=cds2339	0	0	STM14_2418	ompS	putative porin	0	NA
NC_016856	RefSeq	CDS	2090431	2090643	-		0	ID=cds2340	0	65,58333333	STM14_2419	STM14_2419	putative cold-shock protein	9,378610925	-2,069476383
NC_016856	RefSeq	CDS	2090630	2090842	-		0	ID=cds2341	0	130,25	STM14_2420	cspB	putative cold-shock protein	18,47302152	-1,861694559
NC_016856	RefSeq	CDS	2091089	2091208	-		0	ID=cds2342	0	0	STM14_2421	STM14_2421	hypothetical protein	0	NA
NC_016856	RefSeq	CDS	2091297	2092565	-		0	ID=cds2343	0	3,5	STM14_2422	umuC	DNA polymerase V subunit UmuC	0,568400662	-1,710438693
NC_016856	RefSeq	CDS	2092568	2092987	-		0	ID=cds2344	0	1	STM14_2423	umuD	DNA polymerase V subunit UmuD	0,142100166	-0,623264422
NC_016856	RefSeq	CDS	2093114	2093275	-		0	ID=cds2345	0	0	STM14_2424	STM14_2424	putative cytoplasmic protein	0	NA
NC_016856	RefSeq	CDS	2093253	2093495	-		0	ID=cds2346	2	1	STM14_2425	STM14_2425	hypothetical protein	3,660744905	2,693976715
NC_016856	RefSeq	CDS	2093906	2094127	-		0	ID=cds2347	0	7,5	STM14_2427	STM14_2427	hypothetical protein	1,136801324	-2,147403465
NC_016856	RefSeq	CDS	2094340	2095347	+		0	ID=cds2348	10	7,5	STM14_2428	STM14_2428	hypothetical protein	18,73002502	2,621762365
NC_016856	RefSeq	CDS	2095348	2095497	-		0	ID=cds2349	0	0	STM14_2429	STM14_2429	hypothetical protein	0	NA
NC_016856	RefSeq	CDS	2095632	2096201	-		0	ID=cds2350	0	0,5	STM14_2430	STM14_2430	putative tail fiber assembly protein	0	NA
NC_016856	RefSeq	CDS	2096201	2097763	-		0	ID=cds2351	0	2,5	STM14_2431	STM14_2431	hypothetical protein	0,284200331	-1,182723423
NC_016856	RefSeq	CDS	2097750	2098337	-		0	ID=cds2352	0	0	STM14_2432	STM14_2432	hypothetical protein	0	NA
NC_016856	RefSeq	CDS	2099413	2099826	-		0	ID=cds2353	0	0	STM14_2434	STM14_2434	hypothetical protein	0	NA
NC_016856	RefSeq	CDS	2099831	2100364	-		0	ID=cds2354	0	0	STM14_2435	STM14_2435	putative base plate assembly protein	0	NA
NC_016856	RefSeq	CDS	2100364	2101422	-		0	ID=cds2355	0	1,5	STM14_2436	STM14_2436	tail protein	0,284200331	-1,182723423
NC_016856	RefSeq	CDS	2101419	2102759	-		0	ID=cds2356	0	3,5	STM14_2437	STM14_2437	hypothetical protein	0,568400662	-1,710438693
NC_016856	RefSeq	CDS	2102793	2104721	-		0	ID=cds2357	0	3	STM14_2438	STM14_2438	phage related tail protein	0,426300497	-1,498211557
NC_016856	RefSeq	CDS	2104733	2105140	-		0	ID=cds2358	0	1	STM14_2439	STM14_2439	hypothetical protein	0,142100166	-0,623264422
NC_016856	RefSeq	CDS	2105128	2105484	-		0	ID=cds2359	0	3	STM14_2440	STM14_2440	tail tube protein	0,426300497	-1,498211557
NC_016856	RefSeq	CDS	2105484	2106980	-		0	ID=cds2360	0	2	STM14_2441	STM14_2441	tail sheat protein	0,284200331	-1,182723423
NC_016856	RefSeq	CDS	2106970	2107134	-		0	ID=cds2361	0	1	STM14_2442	STM14_2442	hypothetical protein	0,142100166	-0,623264422
NC_016856	RefSeq	CDS	2107138	2107698	-		0	ID=cds2362	0	1	STM14_2443	STM14_2443	hypothetical protein	0,142100166	-0,623264422
NC_016856	RefSeq	CDS	2107695	2108207	-		0	ID=cds2363	0	1	STM14_2444	STM14_2444	hypothetical protein	0,142100166	-0,623264422
NC_016856	RefSeq	CDS	2108179	2108583	-		0	ID=cds2364	0	1	STM14_2445	STM14_2445	hypothetical protein	0,142100166	-0,623264422
NC_016856	RefSeq	CDS	2108580	2108903	-		0	ID=cds2365	0	1	STM14_2446	STM14_2446	hypothetical protein	0,142100166	-0,623264422
NC_016856	RefSeq	CDS	2108906	2109106	-		0	ID=cds2366	0	0	STM14_2447	STM14_2447	hypothetical protein	0	NA
NC_016856	RefSeq	CDS	2109157	2110362	-		0	ID=cds2367	0	0	STM14_2448	STM14_2448	major capsid protein	0	NA
NC_016856	RefSeq	CDS	2110377	2111063	-		0	ID=cds2368	0	1	STM14_2449	STM14_2449	pro-head protease	0,142100166	-0,623264422
NC_016856	RefSeq	CDS	2111005	2112249	-		0	ID=cds2369	0	2	STM14_2450	STM14_2450	portal protein	0,284200331	-1,182723423
NC_016856	RefSeq	CDS	2112246	2112428	-		0	ID=cds2370	0	0	STM14_2451	STM14_2451	hypothetical protein	0	NA
NC_016856	RefSeq	CDS	2112440	2114173	-		0	ID=cds2371	0	6,5	STM14_2452	STM14_2452	terminase large subunit	0,852600993	-1,98056697

NC_01685(	RefSeq	CDS	2114170	2114664	-	0	ID=cds2372	0	2	STM14_2453	STM14_2453	hypothetical protein	0,284200331	-1,182723423
NC_01685(	RefSeq	CDS	2114790	2115140	-	0	ID=cds2373	0	2	STM14_2454	STM14_2454	hypothetical protein	0,284200331	-1,182723423
NC_01685(	RefSeq	CDS	2115201	2115503	-	0	ID=cds2374	0	7	STM14_2455	STM14_2455	hypothetical protein	0,994701159	-2,072978628
NC_01685(	RefSeq	CDS	2115612	2115710	-	0	ID=cds2375	0	0	STM14_2456	STM14_2456	hypothetical protein	0	NA
NC_01685(	RefSeq	CDS	2115723	2116142	-	0	ID=cds2376	0	0	STM14_2457	STM14_2457	hypothetical protein	0	NA
NC_01685(	RefSeq	CDS	2116355	2116840	-	0	ID=cds2377	0	1,5	STM14_2458	STM14_2458	lysis protein	0,284200331	-1,182723423
NC_01685(	RefSeq	CDS	2116837	2117451	-	0	ID=cds2378	0	1,5	STM14_2459	STM14_2459	lytic enzyme %28putative glycohydrolase%29	0,284200331	-1,182723423
NC_01685(	RefSeq	CDS	2117454	2117798	-	0	ID=cds2379	0	0	STM14_2460	STM14_2460	lysis protein %28holin%29	0	NA
NC_01685(	RefSeq	CDS	2117839	2117967	+	0	ID=cds2380	0	2,5	STM14_2461	STM14_2461	hypothetical protein	0,284200331	-1,182723423
NC_01685(	RefSeq	CDS	2118324	2118683	-	0	ID=cds2381	0	0	STM14_2462	STM14_2462	hypothetical protein	0	NA
NC_01685(	RefSeq	CDS	2118714	2119337	-	0	ID=cds2382	0	1,5	STM14_2463	STM14_2463	hypothetical protein	0,284200331	-1,182723423
NC_01685(	RefSeq	CDS	2119348	2120301	-	0	ID=cds2383	0	2,25	STM14_2464	STM14_2464	hypothetical protein	0,284200331	-1,182723423
NC_01685(	RefSeq	CDS	2120345	2121205	-	0	ID=cds2384	0	4,25	STM14_2465	STM14_2465	hypothetical protein	0,568400662	-1,710438693
NC_01685(	RefSeq	CDS	2121222	2121611	-	0	ID=cds2385	0	1	STM14_2466	STM14_2466	crossover junction protein	0,142100166	-0,623264422
NC_01685(	RefSeq	CDS	2121608	2122501	-	0	ID=cds2386	0	0	STM14_2467	STM14_2467	putative DNA methyl transferase	0	NA
NC_01685(	RefSeq	CDS	2122501	2123025	-	0	ID=cds2387	0	0	STM14_2468	STM14_2468	putative transcriptional activator	0	NA
NC_01685(	RefSeq	CDS	2122985	2123803	-	0	ID=cds2388	0	0	STM14_2469	STM14_2469	putative replication protein	0	NA
NC_01685(	RefSeq	CDS	2123800	2124024	-	0	ID=cds2389	0	0	STM14_2470	STM14_2470	hypothetical protein	0	NA
NC_01685(	RefSeq	CDS	2124021	2125178	-	0	ID=cds2390	0	4	STM14_2471	STM14_2471	hypothetical protein	0,568400662	-1,710438693
NC_01685(	RefSeq	CDS	2125175	2125729	-	0	ID=cds2391	0	1,5	STM14_2472	STM14_2472	putative phage-encoded DNA-binding protein	0,284200331	-1,182723423
NC_01685(	RefSeq	CDS	2125758	2125982	-	0	ID=cds2392	0	1,5	STM14_2473	STM14_2473	DNA binding protein	0,284200331	-1,182723423
NC_01685(	RefSeq	CDS	2126080	2126775	+	0	ID=cds2393	0	23	STM14_2474	STM14_2474	regulatory protein	3,268303807	-2,432747446
NC_01685(	RefSeq	CDS	2127590	2127961	+	0	ID=cds2394	0	4	STM14_2475	STM14_2475	hypothetical protein	0,568400662	-1,710438693
NC_01685(	RefSeq	CDS	2128019	2128846	+	0	ID=cds2395	0	5,5	STM14_2476	STM14_2476	hypothetical protein	0,852600993	-1,98056697
NC_01685(	RefSeq	CDS	2128983	2129522	+	0	ID=cds2396	0	0	STM14_2477	STM14_2477	hypothetical protein	0	NA
NC_01685(	RefSeq	CDS	2129593	2129823	+	0	ID=cds2397	0	0	STM14_2478	STM14_2478	hypothetical protein	0	NA
NC_01685(	RefSeq	CDS	2129820	2130335	+	0	ID=cds2398	0	0,5	STM14_2479	STM14_2479	hypothetical protein	0	NA
NC_01685(	RefSeq	CDS	2130332	2130949	+	0	ID=cds2399	0	1,75	STM14_2480	STM14_2480	hypothetical protein	0,284200331	-1,182723423
NC_01685(	RefSeq	CDS	2130946	2131779	+	0	ID=cds2400	0	1,75	STM14_2481	STM14_2481	hypothetical protein	0,284200331	-1,182723423
NC_01685(	RefSeq	CDS	2131783	2132352	+	0	ID=cds2401	0	0,5	STM14_2482	STM14_2482	endodeoxyribonuclease	0	NA
NC_01685(	RefSeq	CDS	2132377	2132619	+	0	ID=cds2402	0	1,5	STM14_2483	STM14_2483	hypothetical protein	0,284200331	-1,182723423
NC_01685(	RefSeq	CDS	2132621	2133610	+	0	ID=cds2403	0	1	STM14_2484	STM14_2484	integrase protein	0,142100166	-0,623264422
NC_01685(	RefSeq	CDS	2133902	2134699	+	0	ID=cds2404	0	32	STM14_2485	yeeI	putative inner membrane protein	4,547205297	-2,489878815
NC_01685(	RefSeq	CDS	2136009	2136482	+	0	ID=cds2405	0	1,5	STM14_2489	STM14_2489	putative endoprotease	0,284200331	-1,182723423
NC_01685(	RefSeq	CDS	2136957	2137655	+	0	ID=cds2406	0	1	STM14_2490	STM14_2490	putative DNA-binding protein	0,142100166	-0,623264422
NC_01685(	RefSeq	CDS	2137998	2140166	+	0	ID=cds2407	0	0	STM14_2491	STM14_2491	hypothetical protein	0	NA
NC_01685(	RefSeq	CDS	2140223	2141953	+	0	ID=cds2408	0	1	STM14_2492	STM14_2492	hypothetical protein	0,142100166	-0,623264422
NC_01685(	RefSeq	CDS	2142163	2142336	+	0	ID=cds2409	0	1,5	STM14_2493	STM14_2493	hypothetical protein	0,284200331	-1,182723423
NC_01685(	RefSeq	CDS	2142487	2142741	-	0	ID=cds2410	0	2	STM14_2494	STM14_2494	hypothetical protein	0,284200331	-1,182723423
NC_01685(	RefSeq	CDS	2142858	2144312	-	0	ID=cds2411	0	11,5	STM14_2495	amn	AMP nucleosidase	1,705201986	-2,329925387
NC_01685(	RefSeq	CDS	2144508	2144834	-	0	ID=cds2412	0	7	STM14_2496	STM14_2496	putative cytoplasmic protein	0,994701159	-2,072978628
NC_01685(	RefSeq	CDS	2144828	2145214	-	0	ID=cds2413	0	7	STM14_2497	STM14_2497	hypothetical protein	0,994701159	-2,072978628
NC_01685(	RefSeq	CDS	2145540	2145707	+	0	ID=cds2414	0	4,166666667	STM14_2498	STM14_2498	hypothetical protein	0,568400662	-1,710438693
NC_01685(	RefSeq	CDS	2145928	2147385	-	0	ID=cds2415	0	6,5	STM14_2500	yeeO	hypothetical protein	0,852600993	-1,98056697
NC_01685(	RefSeq	CDS	2147387	2147539	-	0	ID=cds2416	0	0	STM14_2501	STM14_2501	hypothetical protein	0	NA
NC_01685(	RefSeq	CDS	2147847	2148776	-	0	ID=cds2417	0	16	STM14_2503	erfK	hypothetical protein	2,273602649	-2,403729808
NC_01685(	RefSeq	CDS	2148854	2149924	-	0	ID=cds2418	0	23	STM14_2504	cobT	nicotinate-nucleotide--dimethylbenzimidazole phosphoril	3,268303807	-2,432747446
NC_01685(	RefSeq	CDS	2149951	2150694	-	0	ID=cds2419	0	30	STM14_2505	cobS	cobalamin synthase	4,263004966	-2,464060063
NC_01685(	RefSeq	CDS	2150691	2151236	-	0	ID=cds2420	0	17,5	STM14_2506	cobU	adenosylcobinamide kinase%2Fadenosylcobinamide-phos	2,55780298	-2,419166771
NC_01685(	RefSeq	CDS	2151233	2152753	-	0	ID=cds2421	0	28	STM14_2507	cbiP	cobyric acid synthase	3,978804635	-2,448045729
NC_01685(	RefSeq	CDS	2152750	2153565	-	0	ID=cds2422	0	24,5	STM14_2508	cbiO	cobalt transporter ATP-binding subunit	3,410403973	-2,434086198
NC_01685(	RefSeq	CDS	2153574	2154251	-	0	ID=cds2423	0	37,75	STM14_2509	cbiQ	vitamin B12 biosynthetic protein	5,39980629	-2,264331336
NC_01685(	RefSeq	CDS	2154238	2154519	-	0	ID=cds2424	0	2,25	STM14_2510	cbiN	cobalt transport protein CbiN	0,284200331	-1,182723423
NC_01685(	RefSeq	CDS	2154521	2155168	-	0	ID=cds2425	0	19,5	STM14_2511	cbiM	cobalt transport protein CbiM	2,842003311	-2,427810709
NC_01685(	RefSeq	CDS	2155255	2155968	-	0	ID=cds2426	0	33,5	STM14_2512	cbiL	cobalt-precorrin-2 C%2820%29-methyltransferase	4,831405628	-2,523251848
NC_01685(	RefSeq	CDS	2155965	2156759	-	0	ID=cds2427	0	42	STM14_2513	cbiK	vitamin B12 biosynthetic protein	5,968206953	-2,232890319
NC_01685(	RefSeq	CDS	2156762	2157553	-	0	ID=cds2428	0	17,5	STM14_2514	cbiJ	cobalt-precorrin-6x reductase	2,55780298	-2,419166771
NC_01685(	RefSeq	CDS	2157550	2158275	-	0	ID=cds2429	0	28	STM14_2515	cbiH	precorrin-3B C%2817%29-methyltransferase	3,978804635	-2,448045729
NC_01685(	RefSeq	CDS	2158275	2159330	-	0	ID=cds2430	0	25,08333333	STM14_2516	cbiG	cobalamin biosynthesis protein CbiG	3,552504138	-2,43600272
NC_01685(	RefSeq	CDS	2159311	2160084	-	0	ID=cds2431	0	29,25	STM14_2517	cbiF	vitamin B12 biosynthetic protein	4,120904801	-2,455089955
NC_01685(	RefSeq	CDS	2160068	2160646	-	0	ID=cds2432	0	29	STM14_2518	cbiT	cobalt-precorrin-6Y C%2815%29-methyltransferase	4,120904801	-2,455089955
NC_01685(	RefSeq	CDS	2160636	2161241	-	0	ID=cds2433	0	28,5	STM14_2519	cbiE	cobalt-precorrin-6Y C%285%29-methyltransferase	3,978804635	-2,448045729
NC_01685(	RefSeq	CDS	2161235	2162374	-	0	ID=cds2434	0	55,5	STM14_2520	cbiD	cobalt-precorrin-6A synthase	7,95760927	-2,130811198
NC_01685(	RefSeq	CDS	2162374	2163006	-	0	ID=cds2435	0	33	STM14_2521	cbiC	precorrin-8X methylmutase	4,689305463	-2,505858154
NC_01685(	RefSeq	CDS	2163017	2163976	-	0	ID=cds2436	0	41,5	STM14_2522	cbiB	protein CbiB	5,968206953	-2,232890319
NC_01685(	RefSeq	CDS	2163973	2165352	-	0	ID=cds2437	0	36,83333333	STM14_2523	cbiA	cobyrinic acid a%2Cc-diamide synthase	5,257706125	-2,272984196
NC_01685(	RefSeq	CDS	2165668	2165799	-	0	ID=cds2438	0	27	STM14_2524	STM14_2524	hypothetical protein	3,836704469	-2,44262576
NC_01685(	RefSeq	CDS	2165950	2166861	-	0	ID=cds2439	0	54,83333333	STM14_2525	pocR	transcriptional regulator	7,815509104	-2,137668811
NC_01685(	RefSeq	CDS	2167078	2167872	-	0	ID=cds2440	0	24	STM14_2526	pduF	propanediol diffusion facilitator	3,410403973	-2,434086198
NC_01685(	RefSeq	CDS	2168398	2168682	+	0	ID=cds2441	0	44	STM14_2527	pduA	polyhedral body protein	6,252407284	-2,216733793
NC_01685(	RefSeq	CDS	2168679	2169491	+	0	ID=cds2442	0	88	STM14_2528	pudB	polyhedral body protein	12,50481457	-1,954585237
NC_01685(	RefSeq	CDS	2169510	2171174	+	0	ID=cds2443	0	164,75	STM14_2529	pduC	propanediol dehydratase large subunit	23,44652731	-1,884775826
NC_01685(	RefSeq	CDS	2171185	2171859	+	0	ID=cds2444	0	44,25	STM14_2530	pduD	propanediol dehydratase medium subunit	6,252407284	-2,216733793
NC_01685(	RefSeq	CDS	2171874	2172395	+	0	ID=cds2445	0	55,5	STM14_2531	pduE	propanediol dehydratase small subunit	7,95760927	-2,130811198
NC_01685(	RefSeq	CDS	2172405	2174237	+	0	ID=cds2446	0	57	STM14_2532	pduG	propanediol dehydratase reactivation protein	8,099709436	-2,124045284
NC_01685(	RefSeq	CDS	2174227	2174577	+	0	ID=cds2447	0	9,5	STM14_2533	pduH	propanediol dehydratase reactivation protein	1,421001655	-2,256999172
NC_01685(	RefSeq	CDS	2174596	2174871	+	0	ID=cds2448	0	11,5	STM14_2534	pduJ	polyhedral body protein	1,705201986	-2,329925387
NC_01685(	RefSeq	CDS	2174875	2175357	+	0	ID=cds2449	0	31	STM14_2535	pduK	polyhedral body protein	4,405105132	-2,475774913
NC_01685(	RefSeq	CDS	2175357	2175989	+	0	ID=cds2450	0	33,5	STM14_2536	pduL	propanediol utilization protein	4,831405628	-2,523251848
NC_01685(	RefSeq	CDS	2175986	2176477	+	0	ID=cds2451	0	7	STM14_2537	pduM	propanediol utilization protein	0,994701159	-2,072978628
NC_01685(	RefSeq	CDS	2176481	2176756	+	0	ID=cds2452	0	4,75	STM14_2538	pduN	polyhedral body protein	0,710500828	-1,864806942
NC_01685(	RefSeq	CDS	2176766	2177776	+	0	ID=cds2453	0	17,75	STM14_2539	pduO	propanediol utilization protein	2,55780298	-2,419166771
NC_01685(	RefSeq	CDS	2177773	2179167	+	0	ID=cds2454	0	12	STM14_2540	pduP	CoA-dependent propionaldehyde dehydrogenase	1,705201986	-2,329925387
NC_01685(	RefSeq	CDS	2179179	2180291	+	0	ID=cds2455	0	6	STM14_2541	pduQ	propanol dehydrogenase	0,852600993	-1,98056697
NC_01685(	RefSeq	CDS	2180288	2181643	+	0	ID=cds2456	0	17,75	STM14_2542	pduS	polyhedral body protein	2,55780298	-2,419166771
NC_01685(	RefSeq	CDS	2181646	2182200	+	0	ID=cds2457	0	6,75	STM14_2543	pduT	polyhedral body protein	0,994701159	-2,072978628
NC_01685(	RefSeq	CDS	2182200	2182550	+	0	ID=cds2458	0	1,75	STM14_2544	pduU	polyhedral body protein	0,284200331	-1,182723423
NC_01685(	RefSeq	CDS	2182555	2183007	+	0	ID=cds2459	0	1,25	STM14_2545	pduV	propanediol utilization protein	0,142100166	-0,623264422
NC_01685(	RefSeq	CDS	2182992	2184206	+	0	ID=cds2460	0	9,5	STM14_2546	pduW	propionate kinase	1,421001655	-2,256999172
NC_01685(	RefSeq	CDS	2184264	2185166	+	0	ID=cds2461	0	11	STM14_2547	pduX	propanediol utilization protein	1,563101821	-2,297202414
NC_01685(	RefSeq	CDS	2185200	2185535	-	0	ID=cds2462	1	31	STM14_2548	yeeX	hypothetical protein	6,164427502	-0,831122204
NC_01685(	RefSeq	CDS	2185570	2185701	-	0	ID=cds2463	2	10,5	STM14_2549	STM14_2549	hypothetical protein	4,939646395	0,828530271
NC_01685(	RefSeq	CDS	2185780	2186838	-	0	ID=cds2464	0	16,5	STM14_2550	yeeA	putative inner membrane protein	2,273602649	-2,403729808
NC_01685(	RefSeq	CDS	2186957	2187424	-	0	ID=cds2465	0	47,5	STM14_2551	sbmC	DNA gyrase inhibitor	6,820807946	-2,185729286
NC_01685(	RefSeq	CDS	2187577	2188749	-	0	ID=cds2466	0	30,5	STM14_2552	dacD	D-alanyl-D-alanine carboxypeptidase	4,263004966	-2,464060063
NC_01685(	RefSeq	CDS	2188873	2189637	-	0	ID=cds2467	0	89,5	STM14_2553	phsC	thiosulfate reductase cytochrome B subunit	12,7890149	-1,945533971
NC_01685(	RefSeq	CDS	2189634	2190212	-	0	ID=cds2468	0	39,25	STM14_2554	phsB	thiosulfate reductase electron transport protein	5,541906456	-2,25579729
NC_01685(	RefSeq	CDS	2190227	2192503	-	0	ID=cds2469	2	143,75	STM14_2555	phsA	thiosulfate reductase	23,98106858	-1,707574221
NC_01685(	RefSeq	CDS	2192950	2193219	-	0	ID=cds2470	0	0	STM14_2556	STM14_2556	hypothetical protein	0	NA
NC_01685(	RefSeq	CDS	2193122	2195470	+	0	ID=cds2471	0	9	STM14_2557	sopA	secreted effector protein	1,27890149	-2,207741391
NC_01685(	RefSeq	CDS	2195674	2195796	-	0	ID=cds2472	0	4,5	STM14_2558	STM14_2558	hypothetical protein	0,568400662	-1,710438693
NC_01685(	RefSeq	CDS	2195813	2197243	+	0	ID=cds2473	0	16	STM14_2559	sbcB	exonuclease I	2,273602649	-2,403729808
NC_01685(	RefSeq	CDS	2197379	2198743	-	0	ID=cds2474	0	12,33333333	STM14_2560	yeeF	putative amino acid transport protein	1,705201986	-2,329925387
NC_01685(	RefSeq	CDS	2198729	2198896	+	0	ID=cds2475	0	0	STM14_2561	STM14_2561	hypothetical protein	0	NA
NC_01685(	RefSeq	CDS	2198740	2198937	-	0	ID=cds2476	0	0,5	STM14_2562	STM14_2562	hypothetical protein	0	NA
NC_01685(	RefSeq	CDS	2199023	2199937	-	0	ID=cds2477	0	3,5	STM14_2563	yeeY	putative transcriptional regulator	0,568400662	-1,710438693
NC_01685(	RefSeq	CDS	2199983	2200807	-	0	ID=cds2478	0	10	STM14_2564	yeeZ	putative dehydratase	1,421001655	-2,256999172
NC_01685(	RefSeq	CDS	2201167	2202066	+	0	ID=cds2479	0	6	STM14_2565	hisG	ATP phosphoribosyltransferase	0,852600993	-1,98056697
NC_01685(	RefSeq	CDS	2202169	2203473	+	0	ID=cds2480	0	6,5	STM14_2566	hisD	histidinol dehydrogenase	0,852600993	-1,98056697
NC_01685(	RefSeq	CDS	2203470	2204549	+	0	ID=cds2481	0	2,5	STM14_2567	hisC	histidinol-phosphate aminotransferase	0,284200331	-1,182723423
NC_01685(	RefSeq	CDS	2204546	2205613	+	0	ID=cds2482	0	7	STM14_2568	hisB	imidazole glycerol-phosphate dehydratase%2Fhistidinol p	0,994701159	-2,072978628
NC_01685(	RefSeq	CDS	2205613	2206203	+	0	ID=cds2483	0	8	STM14_2569	hisH	imidazole glycerol phosphate synthase subunit HisH	1,136801324	-2,147403465
NC_01685(	RefSeq	CDS	2206203	2206940	+	0	ID=cds2484	0	5	STM14_2570	hisA	1-%285-phosphoribosyl%29-5-%5B%285-phosphoribosyla	0,710500828	-1,864806942
NC_01685(	RefSeq	CDS	2206922	2207698	+	0	ID=cds2485	0	8,5	STM14_2571	hisF	imidazole glycerol phosphate synthase subunit HisF	1,136801324	-2,147403465
NC_01685(	RefSeq	CDS	2207692	2208303	+	0	ID=cds2486	0	5,5	STM14_2572	hisI	bifunctional phosphoribosyl-AMP cyclohydrolase%2Fphos	0,852600993	-1,98056697
NC_01685(	RefSeq	CDS	2208384	2209367	-	0	ID=cds2487	0	47,5	STM14_2573	wzzB	lipopolysaccharide O-antigen chain length regulator	6,820807946	-2,185729286
NC_01685(	RefSeq	CDS	2209510	2210676	-	0	ID=cds2488	0	47,5	STM14_2574	udg	UDP-glucose%2FGDP-mannose dehydrogenase	6,820807946	-2,185729286
NC_01685(	RefSeq	CDS	2210913	2212319	-	0	ID=cds2489	0	45,5	STM14_2575	gnd	6-phosphogluconate dehydrogenase	6,536607615	-2,201017198
NC_01685(	RefSeq	CDS	2212483	2213913	-	0	ID=cds2490	0	36	STM14_2576	rfbP	undecaprenol-phosphate galactosephosphotransferase%2	5,115605959	-2,281753063
NC_01685(	RefSeq	CDS	2213985	2215418	-	0	ID=cds2491	0	42,25	STM14_2577	rfbK	phosphomannomutase	5,968206953	-2,232890319
NC_01685(	RefSeq	CDS	2215405	2216844	-	0	ID=cds2492	0	49,25	STM14_2578	rfbM	mannose-1-phosphate guanylyltransferase	6,962908111	-2,178239812
NC_01685(	RefSeq	CDS	2216845	2217789	-	0	ID=cds2493	0	24	STM14_2579	rfbN	rhamnosyl transferase	3,410403973	-2,434086198
NC_01685(	RefSeq	CDS	2217790	2218851	-	0	ID=cds2494	0	33	STM14_2580	rfbU	mannosyl transferase	4,689305463	-2,505858154
NC_01685(	RefSeq	CDS	2219171	2220172	-	0	ID=cds2495	0	22	STM14_2581	rfbV	abequosyltransferase	3,126203642	-2,431590384

NC_01685	RefSeq	CDS	2220177	2221469	-	0	ID=cds249	0	18,5	STM14_2582	rfbX	putative O-antigen transferase	2,55780298	-2,419166771
NC_01685	RefSeq	CDS	2221551	2222450	-	0	ID=cds249	0	16,33333333	STM14_2583	rfbJ	CDP-abequose synthase	2,273602649	-2,403729808
NC_01685	RefSeq	CDS	2222478	2223791	-	0	ID=cds249	0	101,8333333	STM14_2584	rfbH	CDP-6-deoxy-D-xylo-4-hexulose-3-dehydrase	14,49421688	-1,89737012
NC_01685	RefSeq	CDS	2223818	2224897	-	0	ID=cds249	0	58	STM14_2585	rfbG	CDP glucose 4%2C6-dehydratase	8,241809601	-2,117369507
NC_01685	RefSeq	CDS	2224902	2225675	-	0	ID=cds250	1	35,5	STM14_2586	rfbF	glucose-1-phosphate cytidylyltransferase	6,874928329	-0,96718351
NC_01685	RefSeq	CDS	2225672	2226664	-	0	ID=cds250	0	45	STM14_2587	rfbI	CDP-6-deoxy-delta-3%2C4-glucoseen reductase	6,394507449	-2,208821363
NC_01685	RefSeq	CDS	2226670	2227221	-	0	ID=cds250	0	15,5	STM14_2588	rfbC	dTDP-4-deoxyrhamnose 3%2C5 epimerase	2,273602649	-2,403729808
NC_01685	RefSeq	CDS	2227222	2228100	-	0	ID=cds250	0	15,5	STM14_2589	rfbA	dTDP-glucose pyrophosphorylase	2,273602649	-2,403729808
NC_01685	RefSeq	CDS	2228148	2229047	-	0	ID=cds250	0	25	STM14_2590	rfbD	dTDP-4-dehydrorhamnose reductase	3,552504138	-2,43600272
NC_01685	RefSeq	CDS	2229047	2230132	-	0	ID=cds250	1	55,5	STM14_2591	rfbB	dTDP-glucose-4%2C6-dehydratase	9,71693164	-1,386337946
NC_01685	RefSeq	CDS	2230509	2231402	-	0	ID=cds250	0	42,5	STM14_2592	galF	UTP--glucose-1-phosphate uridylyltransferase subunit Gal	5,968206953	-2,232890319
NC_01685	RefSeq	CDS	2231580	2232983	-	0	ID=cds250	0	1	STM14_2593	wcaM	putative colanic acid biosynthesis protein	0,142100166	-0,623264422
NC_01685	RefSeq	CDS	2232994	2234214	-	0	ID=cds250	0	0	STM14_2594	wcaL	putative glycosyl transferase	0	NA
NC_01685	RefSeq	CDS	2234211	2235491	-	0	ID=cds250	0	1	STM14_2595	wcaK	putative pyruvyl transferase	0,142100166	-0,623264422
NC_01685	RefSeq	CDS	2235513	2236991	-	0	ID=cds251	0	2	STM14_2596	wzxC	colanic acid exporter	0,284200331	-1,182723423
NC_01685	RefSeq	CDS	2237124	2238518	-	0	ID=cds251	0	0	STM14_2597	wcaJ	putative UDP-glucose lipid carrier transferase	0	NA
NC_01685	RefSeq	CDS	2238572	2239942	-	0	ID=cds251	0	1	STM14_2598	cpsG	phosphomannomutase	0,142100166	-0,623264422
NC_01685	RefSeq	CDS	2239941	2240216	+	0	ID=cds251	0	0	STM14_2599	STM14_2599	hypothetical protein	0	NA
NC_01685	RefSeq	CDS	2240053	2241489	-	0	ID=cds251	0	0	STM14_2600	manC	mannose-1-phosphate guanylyltransferase	0	NA
NC_01685	RefSeq	CDS	2241492	2242715	-	0	ID=cds251	0	0	STM14_2601	wcaI	glycosyl transferase family protein	0	NA
NC_01685	RefSeq	CDS	2242712	2243185	-	0	ID=cds251	0	0	STM14_2602	wcaH	GDP-mannose mannosyl hydrolase	0	NA
NC_01685	RefSeq	CDS	2243188	2244153	-	0	ID=cds251	0	2	STM14_2603	wcaG	GDP-fucose synthetase	0,284200331	-1,182723423
NC_01685	RefSeq	CDS	2244156	2245277	-	0	ID=cds251	0	0	STM14_2604	gmd	GDP-D-mannose dehydratase	0	NA
NC_01685	RefSeq	CDS	2245301	2245855	-	0	ID=cds251	0	0	STM14_2605	wcaF	putative colanic acid biosynthesis acetyltransferase WcaF	0	NA
NC_01685	RefSeq	CDS	2245871	2246617	-	0	ID=cds252	0	0	STM14_2606	wcaE	glycosyl transferase family protein	0	NA
NC_01685	RefSeq	CDS	2246630	2247844	-	0	ID=cds252	0	0	STM14_2607	wcaD	putative colanic acid biosynthesis protein	0	NA
NC_01685	RefSeq	CDS	2247819	2249036	-	0	ID=cds252	0	0	STM14_2608	wcaC	glycosyl transferase family protein	0	NA
NC_01685	RefSeq	CDS	2249033	2249521	-	0	ID=cds252	0	0	STM14_2609	wcaB	putative colanic acid biosynthesis acetyltransferase WcaB	0	NA
NC_01685	RefSeq	CDS	2249524	2250366	-	0	ID=cds252	0	0,5	STM14_2610	wcaA	glycosyl transferase family protein	0	NA
NC_01685	RefSeq	CDS	2250453	2252612	-	0	ID=cds252	0	0	STM14_2611	wzc	tyrosine kinase	0	NA
NC_01685	RefSeq	CDS	2252609	2253058	-	0	ID=cds252	0	0	STM14_2612	wzb	tyrosine phosphatase	0	NA
NC_01685	RefSeq	CDS	2253064	2254203	-	0	ID=cds252	0	0	STM14_2613	wza	putative outer membrane polysaccharide export protein	0	NA
NC_01685	RefSeq	CDS	2254277	2254522	+	0	ID=cds252	0	0	STM14_2614	STM14_2614	hypothetical protein	0	NA
NC_01685	RefSeq	CDS	2254878	2256458	+	0	ID=cds252	0	22	STM14_2615	yegH	putative inner membrane protein	3,126203642	-2,431590384
NC_01685	RefSeq	CDS	2256544	2258400	-	0	ID=cds253	0	34,33333333	STM14_2616	asmA	putative assembly protein	4,831405628	-2,523251848
NC_01685	RefSeq	CDS	2258439	2259020	-	0	ID=cds253	0	7,833333333	STM14_2617	dcd	deoxycytidine triphosphate deaminase	1,136801324	-2,147403465
NC_01685	RefSeq	CDS	2259111	2259752	-	0	ID=cds253	0	12,5	STM14_2618	udk	uridine kinase	1,705201986	-2,329925387
NC_01685	RefSeq	CDS	2259828	2259998	-	0	ID=cds253	0	4	STM14_2619	STM14_2619	hypothetical protein	0,568400662	-1,710438693
NC_01685	RefSeq	CDS	2260083	2263073	+	0	ID=cds253	0	20,5	STM14_2620	yegE	diguanylate cyclase	2,842003311	-2,427810709
NC_01685	RefSeq	CDS	2263041	2263910	-	0	ID=cds253	0	0	STM14_2621	alkA	3-methyladenine DNA glycosylase	0	NA
NC_01685	RefSeq	CDS	2264044	2265396	+	0	ID=cds253	0	1	STM14_2622	yegD	putative chaperone	0,142100166	-0,623264422
NC_01685	RefSeq	CDS	2265633	2265749	-	0	ID=cds253	0	0	STM14_2623	STM14_2623	hypothetical protein	0	NA
NC_01685	RefSeq	CDS	2265837	2267078	+	0	ID=cds253	0	5,5	STM14_2624	STM14_2624	multidrug efflux system subunit MdtA	0,852600993	-1,98056697
NC_01685	RefSeq	CDS	2267078	2270200	+	0	ID=cds253	0	3,5	STM14_2625	yegN	multidrug efflux system subunit MdtB	0,568400662	-1,710438693
NC_01685	RefSeq	CDS	2270201	2273281	+	0	ID=cds254	0	3	STM14_2626	yegO	multidrug efflux system subunit MdtC	0,426300497	-1,498211557
NC_01685	RefSeq	CDS	2273278	2274690	+	0	ID=cds254	0	2,5	STM14_2627	yegB	multidrug efflux system protein MdtE	0,284200331	-1,182723423
NC_01685	RefSeq	CDS	2274690	2276093	+	0	ID=cds254	0	3,5	STM14_2628	baeS	signal transduction histidine-protein kinase BaeS	0,568400662	-1,710438693
NC_01685	RefSeq	CDS	2276090	2276812	+	0	ID=cds254	0	3	STM14_2629	baeR	DNA-binding transcriptional regulator BaeR	0,426300497	-1,498211557
NC_01685	RefSeq	CDS	2277399	2278283	+	0	ID=cds254	0	0	STM14_2631	STM14_2631	putative cytoplasmic protein	0	NA
NC_01685	RefSeq	CDS	2278283	2278999	+	0	ID=cds254	0	0	STM14_2632	STM14_2632	putative inner membrane protein	0	NA
NC_01685	RefSeq	CDS	2279010	2281121	+	0	ID=cds254	0	2	STM14_2633	STM14_2633	putative inner membrane protein	0,284200331	-1,182723423
NC_01685	RefSeq	CDS	2281237	2282598	+	0	ID=cds254	0	7,5	STM14_2634	yegQ	putative protease	1,136801324	-2,147403465
NC_01685	RefSeq	CDS	2282708	2282821	-	0	ID=cds254	0	0	STM14_2635	STM14_2635	hypothetical protein	0	NA
NC_01685	RefSeq	CDS	2284131	2284547	-	0	ID=cds254	0	1	STM14_2637	STM14_2637	putative cytoplasmic protein	0,142100166	-0,623264422
NC_01685	RefSeq	CDS	2284669	2285004	-	0	ID=cds255	0	2	STM14_2638	STM14_2638	putative inner membrane protein	0,284200331	-1,182723423
NC_01685	RefSeq	CDS	2285114	2285233	+	0	ID=cds255	0	0	STM14_2639	STM14_2639	hypothetical protein	0	NA
NC_01685	RefSeq	CDS	2285366	2285482	-	0	ID=cds255	0	0	STM14_2640	STM14_2640	hypothetical protein	0	NA
NC_01685	RefSeq	CDS	2285566	2286465	+	0	ID=cds255	0	0	STM14_2641	yegS	lipid kinase	0	NA
NC_01685	RefSeq	CDS	2286518	2287570	-	0	ID=cds255	0	2,5	STM14_2642	fbaB	fructose-bisphosphate aldolase	0,284200331	-1,182723423
NC_01685	RefSeq	CDS	2287824	2289095	+	0	ID=cds255	0	1	STM14_2643	yegT	putative transport protein	0,142100166	-0,623264422
NC_01685	RefSeq	CDS	2289092	2290096	+	0	ID=cds255	0	1,5	STM14_2644	yegU	putative glycohydrolase	0,284200331	-1,182723423
NC_01685	RefSeq	CDS	2290093	2291058	+	0	ID=cds255	0	0,5	STM14_2645	yegV	putative sugar kinase	0	NA
NC_01685	RefSeq	CDS	2291032	2291778	-	0	ID=cds255	0	12	STM14_2646	yegW	putative regulatory protein	1,705201986	-2,329925387
NC_01685	RefSeq	CDS	2291814	2292614	-	0	ID=cds255	0	2	STM14_2647	thiD	phosphomethylpyrimidine kinase	0,284200331	-1,182723423
NC_01685	RefSeq	CDS	2292601	2293398	-	0	ID=cds256	0	4	STM14_2648	thiM	hydroxyethylthiazole kinase	0,568400662	-1,710438693
NC_01685	RefSeq	CDS	2293497	2293682	-	0	ID=cds256	0	19	STM14_2649	STM14_2649	hypothetical protein	2,699903145	-2,42419778
NC_01685	RefSeq	CDS	2293808	2294122	+	0	ID=cds256	0	1	STM14_2650	STM14_2650	hypothetical protein	0,142100166	-0,623264422
NC_01685	RefSeq	CDS	2294185	2294343	-	0	ID=cds256	0	0	STM14_2651	STM14_2651	hypothetical protein	0	NA
NC_01685	RefSeq	CDS	2294385	2295392	-	0	ID=cds256	0	0	STM14_2652	stcD	putative outer membrane lipoprotein	0	NA
NC_01685	RefSeq	CDS	2295408	2297897	-	0	ID=cds256	0	0	STM14_2653	stcC	putative outer membrane protein	0	NA
NC_01685	RefSeq	CDS	2297911	2298594	-	0	ID=cds256	0	0	STM14_2654	stcB	putative periplasmic chaperone protein	0	NA
NC_01685	RefSeq	CDS	2298650	2299180	-	0	ID=cds256	0	4	STM14_2655	stcA	putative fimbrial-like protein	0,568400662	-1,710438693
NC_01685	RefSeq	CDS	2299459	2299740	-	0	ID=cds256	0	0	STM14_2656	yehE	putative outer membrane protein	0	NA
NC_01685	RefSeq	CDS	2300018	2301127	-	0	ID=cds256	0	46	STM14_2657	mrp	putative ATPase	6,536607615	-2,201017198
NC_01685	RefSeq	CDS	2301292	2303325	+	0	ID=cds257	0	39,5	STM14_2658	metG	methionyl-tRNA synthetase	5,684006621	-2,24950002
NC_01685	RefSeq	CDS	2303566	2304024	+	0	ID=cds257	0	1	STM14_2659	yehR	putative lipoprotein	0,142100166	-0,623264422
NC_01685	RefSeq	CDS	2304196	2304726	+	0	ID=cds257	0	0	STM14_2660	STM14_2660	hypothetical protein	0	NA
NC_01685	RefSeq	CDS	2304783	2305250	-	0	ID=cds257	0	11,5	STM14_2661	yehS	putative cytoplasmic protein	1,705201986	-2,329925387
NC_01685	RefSeq	CDS	2305297	2306016	-	0	ID=cds257	0	4,5	STM14_2662	yehT	putative two-component response-regulatory protein Yeh	0,568400662	-1,710438693
NC_01685	RefSeq	CDS	2306013	2307800	-	0	ID=cds257	0	15,5	STM14_2663	yehU	putative sensor kinase	2,273602649	-2,403729808
NC_01685	RefSeq	CDS	2307921	2308652	+	0	ID=cds257	0	1	STM14_2664	yehV	putative transcriptional repressor	0,142100166	-0,623264422
NC_01685	RefSeq	CDS	2308712	2308819	+	0	ID=cds257	0	4,25	STM14_2665	STM14_2665	hypothetical protein	0,568400662	-1,710438693
NC_01685	RefSeq	CDS	2308800	2309531	-	0	ID=cds257	0	3,75	STM14_2666	yehW	putative ABC-type proline%2Fglycine betaine transport sy	0,568400662	-1,710438693
NC_01685	RefSeq	CDS	2309515	2310462	-	0	ID=cds257	0	1,5	STM14_2667	yehX	putative ABC-type proline%2Fglycine betaine transport sy	0,284200331	-1,182723423
NC_01685	RefSeq	CDS	2310455	2311624	-	0	ID=cds258	0	4,5	STM14_2668	yehY	putative ABC-type proline%2Fglycine betaine transport sy	0,568400662	-1,710438693
NC_01685	RefSeq	CDS	2311628	2312545	-	0	ID=cds258	0	1	STM14_2669	yehZ	putative transport protein	0,142100166	-0,623264422
NC_01685	RefSeq	CDS	2312726	2315023	-	0	ID=cds258	0	21,5	STM14_2670	bglX	periplasmic beta-D-glucoside glucohydrolase	3,126203642	-2,431590384
NC_01685	RefSeq	CDS	2315124	2315237	-	0	ID=cds258	0	0	STM14_2671	STM14_2671	hypothetical protein	0	NA
NC_01685	RefSeq	CDS	2315290	2317020	+	0	ID=cds258	0	17,5	STM14_2672	dld	D-lactate dehydrogenase	2,55780298	-2,419166771
NC_01685	RefSeq	CDS	2317078	2318025	-	0	ID=cds258	1	8,083333333	STM14_2673	pbpG	D-alanyl-D-alanine endopeptidase	2,896123694	0,419298367
NC_01685	RefSeq	CDS	2317916	2318191	-	0	ID=cds258	1	0,25	STM14_2674	STM14_2674	hypothetical protein	1,75932237	1,868512432
NC_01685	RefSeq	CDS	2318191	2318778	-	0	ID=cds258	0	1	STM14_2675	yohC	putative transport protein	0,142100166	-0,623264422
NC_01685	RefSeq	CDS	2318810	2318917	+	0	ID=cds258	0	0	STM14_2676	STM14_2676	hypothetical protein	0	NA
NC_01685	RefSeq	CDS	2318910	2319506	+	0	ID=cds258	0	2	STM14_2677	yohD	hypothetical protein	0,284200331	-1,182723423
NC_01685	RefSeq	CDS	2319555	2320316	-	0	ID=cds259	0	1,5	STM14_2678	yohF	acetoin dehydrogenase	0,284200331	-1,182723423
NC_01685	RefSeq	CDS	2320382	2321818	-	0	ID=cds259	0	0	STM14_2679	yohG	multidrug resistance outer membrane protein MdtQ	0	NA
NC_01685	RefSeq	CDS	2322009	2322206	+	0	ID=cds259	0	0	STM14_2680	STM14_2680	hypothetical protein	0	NA
NC_01685	RefSeq	CDS	2322277	2323215	-	0	ID=cds259	0	3	STM14_2681	yohI	tRNA-dihydrouridine synthase C	0,426300497	-1,498211557
NC_01685	RefSeq	CDS	2323324	2324517	-	0	ID=cds259	0	0	STM14_2682	STM14_2682	salicylate hydroxylase	0	NA
NC_01685	RefSeq	CDS	2324532	2325176	-	0	ID=cds259	0	2,5	STM14_2683	STM14_2683	putative glutathione S-transferase	0,284200331	-1,182723423
NC_01685	RefSeq	CDS	2325185	2325886	-	0	ID=cds259	0	0,5	STM14_2684	STM14_2684	putative flutathione S-transferase	0	NA
NC_01685	RefSeq	CDS	2325902	2326939	-	0	ID=cds259	0	1	STM14_2685	STM14_2685	putative 1%2C2-dioxygenase	0,142100166	-0,623264422
NC_01685	RefSeq	CDS	2326951	2328309	-	0	ID=cds259	0	1	STM14_2686	STM14_2686	putative sugar transporter	0,142100166	-0,623264422
NC_01685	RefSeq	CDS	2328435	2329343	+	0	ID=cds259	0	2,5	STM14_2687	STM14_2687	putative transcriptional regulator	0,284200331	-1,182723423
NC_01685	RefSeq	CDS	2329475	2329873	+	0	ID=cds260	0	1	STM14_2688	yohJ	hypothetical protein	0,142100166	-0,623264422
NC_01685	RefSeq	CDS	2329870	2330565	+	0	ID=cds260	0	2	STM14_2689	yohK	hypothetical protein	0,284200331	-1,182723423
NC_01685	RefSeq	CDS	2330719	2331603	+	0	ID=cds260	0	3	STM14_2690	cdd	cytidine deaminase	0,426300497	-1,498211557
NC_01685	RefSeq	CDS	2331780	2332499	+	0	ID=cds260	1	13	STM14_2691	sanA	hypothetical protein	3,606624522	-0,044862546
NC_01685	RefSeq	CDS	2332496	2332741	+	0	ID=cds260	0	2	STM14_2692	STM14_2692	putative inner membrane protein	0,284200331	-1,182723423
NC_01685	RefSeq	CDS	2332946	2334187	+	0	ID=cds260	0	28,25	STM14_2693	STM14_2693	putative oxidoreductase	3,978804635	-2,448045729
NC_01685	RefSeq	CDS	2334181	2335416	+	0	ID=cds260	0	20,25	STM14_2694	yeiA	dihydropyrimidine dehydrogenase	2,842003311	-2,427810709
NC_01685	RefSeq	CDS	2335491	2336501	-	0	ID=cds260	0	5	STM14_2695	mglC	beta-methylgalactoside transporter inner membrane prot	0,710500828	-1,864806942
NC_01685	RefSeq	CDS	2336517	2338037	-	0	ID=cds260	0	9,5	STM14_2696	mglA	galactose%2Fmethyl galaxtoside transporter ATP-binding	1,421001655	-2,256999172
NC_01685	RefSeq	CDS	2338065	2338184	+	0	ID=cds260	0	0	STM14_2697	STM14_2697	hypothetical protein	0	NA
NC_01685	RefSeq	CDS	2338171	2339169	-	0	ID=cds261	0	21	STM14_2698	mglB	galactose transport protein	2,984103476	-2,430148754
NC_01685	RefSeq	CDS	2339344	2339451	+	0	ID=cds261	0	0,285714286	STM14_2699	STM14_2699	hypothetical protein	0	NA
NC_01685	RefSeq	CDS	2339668	2340690	-	0	ID=cds261	0	9,5	STM14_2700	galS	DNA-binding transcriptional regulator GalS	1,421001655	-2,256999172
NC_01685	RefSeq	CDS	2340683	2340847	+	0	ID=cds261	0	0	STM14_2701	STM14_2701	hypothetical protein	0	NA
NC_01685	RefSeq	CDS	2340840	2341982	-	0	ID=cds261	0	5,5	STM14_2702	yeiB	hypothetical protein	0,852600993	-1,98056697
NC_01685	RefSeq	CDS	2341997	2342665	-	0	ID=cds261	0	5,5	STM14_2703	folE	GTP cyclohydrolase I	0,852600993	-1,98056697
NC_01685	RefSeq	CDS	2342649	2342762	-	0	ID=cds261	0	1	STM14_2704	STM14_2704	hypothetical protein	0,142100166	-0,623264422
NC_01685	RefSeq	CDS	2342720	2342848	-	0	ID=cds261	0	0,5	STM14_2705	STM14_2705	hypothetical protein	0	NA
NC_01685	RefSeq	CDS	2342834	2343067	+	0	ID=cds261	1	0,75	STM14_2706	STM14_2706	hypothetical protein	1,901422535	2,462014545
NC_01685	RefSeq	CDS	2342995	2343852	+	0	ID=cds261	1	13,25	STM14_2707	fghA	S-formylglutathione hydrolase	3,606624522	-0,044862546

NC_016856	RefSeq	CDS	2343841	2344230	-	0	ID=cds2620	0	4,5	STM14_2708	STM14_2708	putative transcriptional regulator	0,568400662	-1,710438693
NC_016856	RefSeq	CDS	2344235	2345602	-	0	ID=cds2621	0	4	STM14_2709	STM14_2709	putative L-serine dehydratase	0,568400662	-1,710438693
NC_016856	RefSeq	CDS	2345635	2345787	-	0	ID=cds2622	0	0	STM14_2710	STM14_2710	hypothetical protein	0	NA
NC_016856	RefSeq	CDS	2345819	2346706	+	0	ID=cds2623	0	1	STM14_2711	STM14_2711	putative phosphoserine phosphatase	0,142100166	-0,623264422
NC_016856	RefSeq	CDS	2346739	2348061	+	0	ID=cds2624	0	2,5	STM14_2712	STM14_2712	putative regulatory protein	0,284200331	-1,182723423
NC_016856	RefSeq	CDS	2348105	2350096	-	0	ID=cds2625	0	2,5	STM14_2713	cirA	colicin I receptor	0,284200331	-1,182723423
NC_016856	RefSeq	CDS	2349999	2350304	+	0	ID=cds2626	0	0	STM14_2714	STM14_2714	hypothetical protein	0	NA
NC_016856	RefSeq	CDS	2350441	2351910	-	0	ID=cds2627	0	28,5	STM14_2715	lysP	lysine transporter	3,978804635	-2,448045729
NC_016856	RefSeq	CDS	2351963	2352097	-	0	ID=cds2628	0	5,5	STM14_2716	STM14_2716	hypothetical protein	0,852600993	-1,98056697
NC_016856	RefSeq	CDS	2352100	2352963	-	0	ID=cds2629	0	16,5	STM14_2717	yeiE	putative DNA-binding transcriptional regulator	2,273602649	-2,403729808
NC_016856	RefSeq	CDS	2352991	2353110	-	0	ID=cds2630	0	1	STM14_2718	STM14_2718	hypothetical protein	0,142100166	-0,623264422
NC_016856	RefSeq	CDS	2353084	2354133	+	0	ID=cds2631	0	3	STM14_2719	yeiH	putative inner membrane protein	0,426300497	-1,498211557
NC_016856	RefSeq	CDS	2354212	2355069	+	0	ID=cds2632	0	16,5	STM14_2720	nfo	endonuclease IV	2,273602649	-2,403729808
NC_016856	RefSeq	CDS	2355134	2356822	-	0	ID=cds2633	0	1	STM14_2721	fruA	PTS system fructose-specific transporter subunit IIBC	0,142100166	-0,623264422
NC_016856	RefSeq	CDS	2356839	2357777	-	0	ID=cds2634	0	1	STM14_2722	fruK	1-phosphofructokinase	0,142100166	-0,623264422
NC_016856	RefSeq	CDS	2357777	2358907	-	0	ID=cds2635	3	2	STM14_2723	fruF	bifunctional fructose-specific PTS IIA%2FHPr protein	5,562167441	2,509670145
NC_016856	RefSeq	CDS	2358928	2359059	+	0	ID=cds2636	0	0	STM14_2724	STM14_2724	hypothetical protein	0	NA
NC_016856	RefSeq	CDS	2359144	2359266	+	0	ID=cds2637	0	0	STM14_2725	STM14_2725	hypothetical protein	0	NA
NC_016856	RefSeq	CDS	2359149	2359277	-	0	ID=cds2638	0	0	STM14_2726	STM14_2726	hypothetical protein	0	NA
NC_016856	RefSeq	CDS	2359276	2360457	+	0	ID=cds2639	0	1	STM14_2727	setB	proton efflux pump	0,142100166	-0,623264422
NC_016856	RefSeq	CDS	2360522	2361187	-	0	ID=cds2640	0	1	STM14_2728	STM14_2728	putative inner membrane protein	0,142100166	-0,623264422
NC_016856	RefSeq	CDS	2361189	2361311	-	0	ID=cds2641	0	0	STM14_2729	STM14_2729	putative inner membrane protein	0	NA
NC_016856	RefSeq	CDS	2361472	2361576	-	0	ID=cds2642	0	0	STM14_2730	STM14_2730	hypothetical protein	0	NA
NC_016856	RefSeq	CDS	2361699	2361953	-	0	ID=cds2643	0	1	STM14_2731	STM14_2731	hypothetical protein	0,142100166	-0,623264422
NC_016856	RefSeq	CDS	2362277	2362849	+	0	ID=cds2644	3	4,5	STM14_2733	yeiP	elongation factor P	5,846367772	1,996213194
NC_016856	RefSeq	CDS	2363062	2364048	+	0	ID=cds2645	0	11	STM14_2734	yeiR	putative cobalamin biosynthetic protein	1,563101821	-2,297202414
NC_016856	RefSeq	CDS	2364078	2364797	+	0	ID=cds2646	0	13,5	STM14_2735	yeiU	putative permease	1,989402318	-2,376535557
NC_016856	RefSeq	CDS	2364975	2365205	-	0	ID=cds2647	0	0	STM14_2736	STM14_2736	hypothetical protein	0	NA
NC_016856	RefSeq	CDS	2365211	2365783	+	0	ID=cds2648	0,5	135,5	STM14_2737	spr	putative outer membrane lipoprotein	19,32562251	-1,866374658
NC_016856	RefSeq	CDS	2365897	2366040	+	0	ID=cds2649	0	5,5	STM14_2738	STM14_2738	hypothetical protein	0,852600993	-1,98056697
NC_016856	RefSeq	CDS	2366109	2367665	+	0	ID=cds2650	0	14	STM14_2739	rtn	hypothetical protein	1,989402318	-2,376535557
NC_016856	RefSeq	CDS	2367772	2369577	+	0	ID=cds2651	0	14,5	STM14_2740	yejA	putative ABC transporter periplasmic binding protein	1,989402318	-2,376535557
NC_016856	RefSeq	CDS	2369587	2370681	+	0	ID=cds2652	0	8,5	STM14_2741	yejB	putative ABC-type dipeptide%2Foligopeptide%2Fnickel tra	1,136801324	-2,147403465
NC_016856	RefSeq	CDS	2370681	2371706	+	0	ID=cds2653	0	4,5	STM14_2742	yejE	putative ABC-type dipeptide%2Foligopeptide%2Fnickel tra	0,568400662	-1,710438693
NC_016856	RefSeq	CDS	2371708	2373297	+	0	ID=cds2654	2	10	STM14_2743	yejF	ABC transporter ATP-binding protein	4,939646395	0,828530271
NC_016856	RefSeq	CDS	2373301	2373645	-	0	ID=cds2655	4	2	STM14_2744	yejG	hypothetical protein	7,321489811	2,765358925
NC_016856	RefSeq	CDS	2373810	2374100	-	0	ID=cds2656	0	2,5	STM14_2745	STM14_2745	bicyclomycin%2Fmultidrug efflux system protein	0,284200331	-1,182723423
NC_016856	RefSeq	CDS	2374036	2375226	-	0	ID=cds2657	0	4,833333333	STM14_2746	bcr	bicyclomycin%2Fmultidrug efflux system protein	0,710500828	-1,864806942
NC_016856	RefSeq	CDS	2375254	2375949	-	0	ID=cds2658	0	6	STM14_2747	rsuA	16S rRNA pseudouridylate synthase A	0,852600993	-1,98056697
NC_016856	RefSeq	CDS	2376101	2377861	+	0	ID=cds2659	0	13,5	STM14_2748	yejH	putative ATP-dependent helicase	1,989402318	-2,376535557
NC_016856	RefSeq	CDS	2377986	2378270	+	0	ID=cds2660	2	35,5	STM14_2749	rplY	50S ribosomal protein L25	8,634250699	-0,354662722
NC_016856	RefSeq	CDS	2378246	2378392	-	0	ID=cds2661	0	0,5	STM14_2750	STM14_2750	hypothetical protein	0	NA
NC_016856	RefSeq	CDS	2378379	2378999	-	0	ID=cds2662	0	7,5	STM14_2751	STM14_2751	putative inner membrane protein	1,136801324	-2,147403465
NC_016856	RefSeq	CDS	2379027	2380034	-	0	ID=cds2663	0	19,5	STM14_2752	yejK	nucleoid-associated protein NdpA	2,842003311	-2,427810709
NC_016856	RefSeq	CDS	2380214	2380441	+	0	ID=cds2664	0	15	STM14_2753	yejL	hypothetical protein	2,131502483	-2,392032191
NC_016856	RefSeq	CDS	2380473	2382233	+	0	ID=cds2665	0	45,5	STM14_2754	yejM	putative hydrolase	6,536607615	-2,201017198
NC_016856	RefSeq	CDS	2382514	2382984	-	0	ID=cds2666	0	0	STM14_2756	STM14_2756	DNA polymerase V subunit	0	NA
NC_016856	RefSeq	CDS	2383045	2383335	+	0	ID=cds2667	0	0	STM14_2757	STM14_2757	virulence protein	0	NA
NC_016856	RefSeq	CDS	2383683	2385512	+	0	ID=cds2668	0	21	STM14_2758	oafA	O-antigen acetylase	2,984103476	-2,430148754
NC_016856	RefSeq	CDS	2385566	2386009	-	0	ID=cds2669	0	0	STM14_2759	STM14_2759	putative cytoplasmic protein	0	NA
NC_016856	RefSeq	CDS	2386387	2386914	-	0	ID=cds2670	0	0	STM14_2760	STM14_2760	putative phage tail fiber assembly protein	0	NA
NC_016856	RefSeq	CDS	2386917	2388158	-	0	ID=cds2671	0	0	STM14_2761	STM14_2761	hypothetical protein	0	NA
NC_016856	RefSeq	CDS	2388219	2388737	-	0	ID=cds2672	0	0	STM14_2762	STM14_2762	hypothetical protein	0	NA
NC_016856	RefSeq	CDS	2388751	2389080	-	0	ID=cds2673	0	0	STM14_2763	STM14_2763	putative inner membrane protein	0	NA
NC_016856	RefSeq	CDS	2389094	2389207	-	0	ID=cds2674	0	0	STM14_2764	STM14_2764	hypothetical protein	0	NA
NC_016856	RefSeq	CDS	2389377	2390708	+	0	ID=cds2675	0	19,5	STM14_2765	STM14_2765	hypothetical protein	2,842003311	-2,427810709
NC_016856	RefSeq	CDS	2390737	2391102	-	0	ID=cds2676	0	0	STM14_2766	STM14_2766	putative phage antiterminator	0	NA
NC_016856	RefSeq	CDS	2391120	2392109	-	0	ID=cds2677	0	2	STM14_2767	STM14_2767	putative cytoplasmic protein	0,284200331	-1,182723423
NC_016856	RefSeq	CDS	2392117	2392347	-	0	ID=cds2678	0	1	STM14_2768	STM14_2768	KilA-N domain protein	0,142100166	-0,623264422
NC_016856	RefSeq	CDS	2392438	2394804	-	0	ID=cds2679	3	9	STM14_2769	sspH2	hypothetical protein	6,5568686	1,313600801
NC_016856	RefSeq	CDS	2394973	2395176	-	0	ID=cds2680	0	0	STM14_2770	STM14_2770	putative phage tail fiber protein	0	NA
NC_016856	RefSeq	CDS	2395473	2396264	-	0	ID=cds2681	0	0	STM14_2771	STM14_2771	putative tail fiber protein of phage	0	NA
NC_016856	RefSeq	CDS	2396453	2396770	+	0	ID=cds2682	0	0	STM14_2772	STM14_2772	virulence protein	0	NA
NC_016856	RefSeq	CDS	2396962	2397525	-	0	ID=cds2683	0	0	STM14_2773	STM14_2773	putative outer membrane protein	0	NA
NC_016856	RefSeq	CDS	2397715	2397861	+	0	ID=cds2684	0	0	STM14_2774	STM14_2774	hypothetical protein	0	NA
NC_016856	RefSeq	CDS	2398255	2398902	+	0	ID=cds2685	0	17,5	STM14_2775	narP	transcriptional regulator NarP	2,55780298	-2,419166771
NC_016856	RefSeq	CDS	2398946	2399989	-	0	ID=cds2686	0	4,75	STM14_2776	ccmH_1	putative heme lyase subunit	0,710500828	-1,864806942
NC_016856	RefSeq	CDS	2399986	2400543	-	0	ID=cds2687	0	4,75	STM14_2777	ccmG_1	heme lyase%2Fdisulfide oxidoreductase	0,710500828	-1,864806942
NC_016856	RefSeq	CDS	2400540	2402471	-	0	ID=cds2688	0	3,5	STM14_2778	ccmF_1	cytochrome c-type biogenesis protein	0,568400662	-1,710438693
NC_016856	RefSeq	CDS	2402468	2402947	-	0	ID=cds2689	0	1,5	STM14_2779	ccmE_1	cytochrome c-type biogenesis protein CcmE	0,284200331	-1,182723423
NC_016856	RefSeq	CDS	2402944	2403156	-	0	ID=cds2690	0	1,25	STM14_2780	ccmD_1	heme exporter protein C	0,142100166	-0,623264422
NC_016856	RefSeq	CDS	2403153	2403890	-	0	ID=cds2691	0	3,5	STM14_2781	ccmC_1	heme exporter protein	0,568400662	-1,710438693
NC_016856	RefSeq	CDS	2403942	2404601	-	0	ID=cds2692	0	0,75	STM14_2782	ccmB_1	heme exporter protein	0,142100166	-0,623264422
NC_016856	RefSeq	CDS	2404598	2405215	-	0	ID=cds2693	0	1	STM14_2783	ccmA_1	cytochrome c biogenesis protein CcmA	0,142100166	-0,623264422
NC_016856	RefSeq	CDS	2405236	2405838	-	0	ID=cds2694	0	4,5	STM14_2784	napC	cytochrome c-type protein NapC	0,568400662	-1,710438693
NC_016856	RefSeq	CDS	2405848	2406297	-	0	ID=cds2695	0	1	STM14_2785	napB	diheme cytochrome c550	0,142100166	-0,623264422
NC_016856	RefSeq	CDS	2406413	2407282	-	0	ID=cds2696	0	2,5	STM14_2786	napH	quinol dehydrogenase membrane component	0,284200331	-1,182723423
NC_016856	RefSeq	CDS	2407269	2407964	-	0	ID=cds2697	0	2,5	STM14_2787	napG	quinol dehydrogenase periplasmic component	0,284200331	-1,182723423
NC_016856	RefSeq	CDS	2407971	2410457	-	0	ID=cds2698	0	14	STM14_2788	napA	nitrate reductase	1,989402318	-2,376535557
NC_016856	RefSeq	CDS	2410454	2410717	-	0	ID=cds2699	0	0,5	STM14_2789	napD	assembly protein for periplasmic nitrate reductase	0	NA
NC_016856	RefSeq	CDS	2410707	2411198	-	0	ID=cds2700	0	0,5	STM14_2790	napF	ferredoxin-type protein	0	NA
NC_016856	RefSeq	CDS	2411310	2411441	+	0	ID=cds2701	0	0	STM14_2791	STM14_2791	hypothetical protein	0	NA
NC_016856	RefSeq	CDS	2411612	2412106	+	0	ID=cds2702	0	38	STM14_2792	eco	ecotin	5,39980629	-2,264331336
NC_016856	RefSeq	CDS	2412315	2413958	-	0	ID=cds2703	0	5	STM14_2793	yojI	multidrug transporter membrane protein%2FATP-binding	0,710500828	-1,864806942
NC_016856	RefSeq	CDS	2414034	2414684	-	0	ID=cds2704	0	2	STM14_2794	alkB	DNA repair system protein	0,284200331	-1,182723423
NC_016856	RefSeq	CDS	2414687	2415748	-	0	ID=cds2705	0	1,5	STM14_2795	ada	O6-methylguanine-DNA methyltransferase%2Ftranscriptio	0,284200331	-1,182723423
NC_016856	RefSeq	CDS	2415829	2416881	-	0	ID=cds2706	0	4	STM14_2796	apbE	thiamine biosynthesis lipoprotein ApbE	0,568400662	-1,710438693
NC_016856	RefSeq	CDS	2416996	2418132	-	0	ID=cds2707	0	446,8333333	STM14_2797	ompC	outer membrane porin protein C	63,51877399	-1,945541056
NC_016856	RefSeq	CDS	2418131	2418256	+	0	ID=cds2708	0	0	STM14_2798	STM14_2798	hypothetical protein	0	NA
NC_016856	RefSeq	ncRNA	2418383	2418563	-	.	ID=rna44;P	0	0,5	STM14_2799	micF	regulatory RNA	0	NA
NC_016856	RefSeq	CDS	2418861	2421530	+	0	ID=cds2709	2	35	STM14_2800	yojN	phosphotransfer intermediate protein in two-component	8,492150534	-0,327624837
NC_016856	RefSeq	CDS	2421547	2422197	+	0	ID=cds2710	1	16	STM14_2801	rcsB	transcriptional regulator RcsB	4,032925019	-0,231745061
NC_016856	RefSeq	CDS	2422300	2425146	-	0	ID=cds2711	3	23	STM14_2802	rcsC	hybrid sensory kinase in two-component regulatory syster	8,546270917	0,457464926
NC_016856	RefSeq	CDS	2425164	2425280	-	0	ID=cds2712	0	2	STM14_2803	STM14_2803	hypothetical protein	0,284200331	-1,182723423
NC_016856	RefSeq	CDS	2425264	2427900	-	0	ID=cds2713	1	129,5	STM14_2804	gyrA	DNA gyrase subunit A	20,23234389	-2,19481852
NC_016856	RefSeq	CDS	2427980	2428210	+	0	ID=cds2714	0	0	STM14_2805	STM14_2805	hypothetical protein	0	NA
NC_016856	RefSeq	CDS	2428153	2428293	+	0	ID=cds2715	0	0	STM14_2806	STM14_2806	hypothetical protein	0	NA
NC_016856	RefSeq	CDS	2428310	2429512	-	0	ID=cds2716	0	3	STM14_2807	STM14_2807	putative dehydratase	0,426300497	-1,498211557
NC_016856	RefSeq	CDS	2429527	2430852	-	0	ID=cds2717	0	0	STM14_2808	STM14_2808	putative permease	0	NA
NC_016856	RefSeq	CDS	2430902	2431786	+	0	ID=cds2718	0	0	STM14_2809	STM14_2809	putative regulatory protein	0	NA
NC_016856	RefSeq	CDS	2431869	2432597	+	0	ID=cds2719	1	25	STM14_2810	ubiG	3-demethylubiquinone-9 3-methyltransferase	5,311826508	-0,631843652
NC_016856	RefSeq	CDS	2432657	2432773	+	0	ID=cds2720	0	2	STM14_2811	STM14_2811	hypothetical protein	0,284200331	-1,182723423
NC_016856	RefSeq	CDS	2432953	2435238	+	0	ID=cds2721	3	198,5	STM14_2812	nrdA	ribonucleotide-diphosphate reductase subunit alpha	33,41379989	-1,638814669
NC_016856	RefSeq	CDS	2435351	2436481	+	0	ID=cds2722	0	70	STM14_2813	nrdB	ribonucleotide-diphosphate reductase subunit beta	9,947011588	-2,04667217
NC_016856	RefSeq	CDS	2436481	2436735	+	0	ID=cds2723	0	1,5	STM14_2814	yfaE	2Fe-2S ferredoxin	0,284200331	-1,182723423
NC_016856	RefSeq	CDS	2436737	2437927	-	0	ID=cds2724	0	3	STM14_2815	STM14_2815	putative permease	0,426300497	-1,498211557
NC_016856	RefSeq	CDS	2438089	2438967	+	0	ID=cds2725	0	5,5	STM14_2816	STM14_2816	putative transcriptional regulator	0,852600993	-1,98056697
NC_016856	RefSeq	CDS	2439083	2440153	-	0	ID=cds2726	1	28,5	STM14_2817	glpQ	glycerophosphodiester phosphodiesterase	5,738127005	-0,73683832
NC_016856	RefSeq	CDS	2440158	2441516	-	0	ID=cds2727	0	33,5	STM14_2818	glpT	sn-glycerol-3-phosphate transporter	4,831405628	-2,523251848
NC_016856	RefSeq	CDS	2441789	2443417	+	0	ID=cds2728	0	0	STM14_2819	glpA	sn-glycerol-3-phosphate dehydrogenase subunit A	0	NA
NC_016856	RefSeq	CDS	2443407	2444666	+	0	ID=cds2729	0	3	STM14_2820	glpB	anaerobic glycerol-3-phosphate dehydrogenase subunit B	0,426300497	-1,498211557
NC_016856	RefSeq	CDS	2444663	2445853	+	0	ID=cds2730	0	1,5	STM14_2821	glpC	sn-glycerol-3-phosphate dehydrogenase subunit C	0,284200331	-1,182723423
NC_016856	RefSeq	CDS	2445948	2446052	+	0	ID=cds2731	0	1	STM14_2822	STM14_2822	hypothetical protein	0,142100166	-0,623264422
NC_016856	RefSeq	CDS	2446155	2446358	+	0	ID=cds2732	2,5	0	STM14_2823	STM14_2823	hypothetical protein	3,51864474	1,925445802
NC_016856	RefSeq	CDS	2446348	2447301	+	0	ID=cds2733	2,5	0	STM14_2824	sseL	deubiquitinase	3,51864474	1,925445802
NC_016856	RefSeq	CDS	2447411	2447587	-	0	ID=cds2734	0	3	STM14_2825	STM14_2825	putative cytoplasmic protein	0,426300497	-1,498211557
NC_016856	RefSeq	CDS	2447593	2448396	-	0	ID=cds2735	0	4	STM14_2826	STM14_2826	putative aldolase	0,568400662	-1,710438693
NC_016856	RefSeq	CDS	2448422	2449711	-	0	ID=cds2736	0	3	STM14_2827	yfaV	putative transport protein	0,426300497	-1,498211557
NC_016856	RefSeq	CDS	2449767	2450984	-	0	ID=cds2737	0	10,5	STM14_2828	yfaW	putative galactonate dehydratase	1,421001655	-2,256999172
NC_016856	RefSeq	CDS	2450986	2451768	-	0	ID=cds2738	0	0	STM14_2829	yfaX	putative transcriptional regulator	0	NA
NC_016856	RefSeq	CDS	2452018	2453214	-	0	ID=cds2739	0	18	STM14_2830	STM14_2830	competence damage-inducible protein A	2,55780298	-2,419166771
NC_016856	RefSeq	CDS	2453329	2453976	-	0	ID=cds2740	0	4	STM14_2831	yfaZ	putative inner membrane protein	0,568400662	-1,710438693
NC_016856	RefSeq	CDS	2454150	2454575	+	0	ID=cds2741	0	10,5	STM14_2832	yfaO	putative NTP pyrophosphohydrolase	1,421001655	-2,256999172
NC_016856	RefSeq	CDS	2454620	2455225	-	0	ID=cds2742	0	57	STM14_2833	ais	aluminum-inducible protein	8,099709436	-2,124045284

NC_01685(	RefSeq	CDS	2455277	2455405	+	0	ID=cds2743	0	0	STM14_2834	STM14_2834	hypothetical protein	0	NA
NC_01685(	RefSeq	CDS	2455524	2456663	+	0	ID=cds2744	0	85	STM14_2835	yfbE	UDP-4-amino-4-deoxy-L-arabinose--oxoglutarate aminotra	12,07851407	-1,968565518
NC_01685(	RefSeq	CDS	2456666	2457649	+	0	ID=cds2745	3	50,5	STM14_2836	pmrF	undecaprenyl phosphate 4-deoxy-4-formamido-L-arabino:	12,38297539	-0,292614157
NC_01685(	RefSeq	CDS	2457646	2459628	+	0	ID=cds2746	0	116	STM14_2837	yfbG	bifunctional UDP-glucuronic acid decarboxylase%2FUDP-4	16,4836192	-1,849193185
NC_01685(	RefSeq	CDS	2459625	2460524	+	0	ID=cds2747	0	30	STM14_2838	STM14_2838	putative cytoplasmic protein	4,263004966	-2,464060063
NC_01685(	RefSeq	CDS	2460521	2462167	+	0	ID=cds2748	3	37,33333333	STM14_2839	arnT	4-amino-4-deoxy-L-arabinose transferase	10,53567323	0,003740066
NC_01685(	RefSeq	CDS	2462164	2462499	+	0	ID=cds2749	0	6	STM14_2840	STM14_2840	putative inner membrane protein	0,852600993	-1,98056697
NC_01685(	RefSeq	CDS	2462499	2462876	+	0	ID=cds2750	0	4,5	STM14_2841	STM14_2841	hypothetical protein	0,568400662	-1,710438693
NC_01685(	RefSeq	CDS	2462871	2463128	-	0	ID=cds2751	8	23,5	STM14_2842	pmrD	polymyxin resistance protein B	17,48498293	1,412223411
NC_01685(	RefSeq	CDS	2463226	2464593	-	0	ID=cds2752	0	39	STM14_2843	menE	O-succinylbenzoic acid--CoA ligase	5,541906456	-2,25579729
NC_01685(	RefSeq	CDS	2464590	2465552	-	0	ID=cds2753	0	44	STM14_2844	menC	O-succinylbenzoate synthase	6,252407284	-2,216733793
NC_01685(	RefSeq	CDS	2465552	2466409	-	0	ID=cds2754	0	50,75	STM14_2845	menB	naphthoate synthase	7,247108442	-2,166048537
NC_01685(	RefSeq	CDS	2466424	2467182	-	0	ID=cds2755	0	39,75	STM14_2846	yfbB	acyl-CoA thioester hydrolase YfbB	5,684006621	-2,24950002
NC_01685(	RefSeq	CDS	2467179	2468849	-	0	ID=cds2756	0	77	STM14_2847	menD	2-succinyl-6-hydroxy-2%2C 4-cyclohexadiene-1-carboxylic	10,94171275	-2,008412955
NC_01685(	RefSeq	CDS	2468935	2470230	-	0	ID=cds2757	0	20,5	STM14_2848	menF	menaquinone-specific isochorismate synthase	2,842003311	-2,427810709
NC_01685(	RefSeq	CDS	2470331	2470642	-	0	ID=cds2758	0	12,5	STM14_2849	elaB	hypothetical protein	1,705201986	-2,329925387
NC_01685(	RefSeq	CDS	2470691	2471152	-	0	ID=cds2759	0	4	STM14_2850	elaA	hypothetical protein	0,568400662	-1,710438693
NC_01685(	RefSeq	region	2471105	2472129	+	.	ID=id89;Nc	0	1	-	-	-	0,142100166	-0,623264422
NC_01685(	RefSeq	CDS	2472195	2473196	+	0	ID=cds2760	0	16	STM14_2852	STM14_2852	putative chemotaxis signal transduction protein	2,273602649	-2,403729808
NC_01685(	RefSeq	CDS	2473237	2475018	-	0	ID=cds2761	0	6	STM14_2853	yfbK	hypothetical protein	0,852600993	-1,98056697
NC_01685(	RefSeq	CDS	2475407	2475529	-	0	ID=cds2762	0	1,5	STM14_2854	STM14_2854	hypothetical protein	0,284200331	-1,182723423
NC_01685(	RefSeq	CDS	2475538	2475645	+	0	ID=cds2763	0	0	STM14_2855	STM14_2855	hypothetical protein	0	NA
NC_01685(	RefSeq	CDS	2475654	2475836	+	0	ID=cds2764	0	0	STM14_2856	STM14_2856	hypothetical protein	0	NA
NC_01685(	RefSeq	CDS	2475906	2477363	-	0	ID=cds2765	0	72,75	STM14_2857	nuoN	NADH dehydrogenase subunit N	10,37331208	-2,029860184
NC_01685(	RefSeq	CDS	2477370	2478899	-	0	ID=cds2766	0	100,75	STM14_2858	nuoM	NADH dehydrogenase subunit M	14,35211672	-1,901319866
NC_01685(	RefSeq	CDS	2479213	2481054	-	0	ID=cds2767	0	189	STM14_2859	nuoL	NADH dehydrogenase subunit L	26,85693129	-1,896201874
NC_01685(	RefSeq	CDS	2481051	2481353	-	0	ID=cds2768	0	28,25	STM14_2860	nuoK	NADH dehydrogenase subunit K	3,978804635	-2,448045729
NC_01685(	RefSeq	CDS	2481350	2481904	-	0	ID=cds2769	0	22,25	STM14_2861	nuoJ	NADH dehydrogenase subunit J	3,126203642	-2,431590384
NC_01685(	RefSeq	CDS	2481915	2482457	-	0	ID=cds2770	0	55,75	STM14_2862	nuoI	NADH dehydrogenase subunit I	7,95760927	-2,130811198
NC_01685(	RefSeq	CDS	2482472	2483449	-	0	ID=cds2771	0	58,25	STM14_2863	nuoH	NADH dehydrogenase subunit H	8,241809601	-2,117369507
NC_01685(	RefSeq	CDS	2483446	2486172	-	0	ID=cds2772	0	141	STM14_2864	nuoG	NADH dehydrogenase subunit G	20,03612334	-1,870012233
NC_01685(	RefSeq	CDS	2486203	2487540	-	0	ID=cds2773	1	37,5	STM14_2865	nuoF	NADH dehydrogenase I subunit F	7,15912866	-1,016778355
NC_01685(	RefSeq	CDS	2487537	2488037	-	0	ID=cds2774	0	12,5	STM14_2866	nuoE	NADH dehydrogenase subunit E	1,705201986	-2,329925387
NC_01685(	RefSeq	CDS	2488040	2489842	-	0	ID=cds2775	0	52,5	STM14_2867	nuoC	bifunctional NADH:ubiquinone oxidoreductase subunit C%	7,389208608	-2,158807911
NC_01685(	RefSeq	CDS	2489929	2490591	-	0	ID=cds2776	0	36	STM14_2868	nuoB	NADH dehydrogenase subunit B	5,115605959	-2,281753063
NC_01685(	RefSeq	CDS	2490607	2491050	-	0	ID=cds2777	0	24,75	STM14_2869	nuoA	NADH dehydrogenase subunit A	3,552504138	-2,43600272
NC_01685(	RefSeq	CDS	2491031	2491153	+	0	ID=cds2778	0	0	STM14_2870	STM14_2870	hypothetical protein	0	NA
NC_01685(	RefSeq	CDS	2491241	2491357	-	0	ID=cds2779	0	8	STM14_2871	STM14_2871	hypothetical protein	1,136801324	-2,147403465
NC_01685(	RefSeq	CDS	2491445	2491597	-	0	ID=cds2780	0	0	STM14_2872	STM14_2872	putative cytoplasmic protein	0	NA
NC_01685(	RefSeq	CDS	2491677	2492615	-	0	ID=cds2781	0	37	STM14_2873	lrhA	NADH dehydrogenase transcriptional repressor	5,257706125	-2,272984196
NC_01685(	RefSeq	CDS	2493547	2494761	+	0	ID=cds2782	2	16	STM14_2874	yfbQ	aminotransferase AlaT	5,792247388	0,405774795
NC_01685(	RefSeq	CDS	2494855	2495454	+	0	ID=cds2783	0	3	STM14_2875	STM14_2875	hypothetical protein	0,426300497	-1,498211557
NC_01685(	RefSeq	CDS	2495546	2497372	-	0	ID=cds2784	0	32,5	STM14_2876	yfbS	putative response regulator	4,547205297	-2,489878815
NC_01685(	RefSeq	CDS	2497449	2498108	-	0	ID=cds2785	0	23,75	STM14_2877	yfbT	putative phosphatase	3,410403973	-2,434086198
NC_01685(	RefSeq	CDS	2498119	2498613	-	0	ID=cds2786	0	20,75	STM14_2878	yfbU	hypothetical protein	2,984103476	-2,430148754
NC_01685(	RefSeq	CDS	2498696	2499151	-	0	ID=cds2787	0	6	STM14_2879	STM14_2879	hypothetical protein	0,852600993	-1,98056697
NC_01685(	RefSeq	CDS	2499179	2499430	-	0	ID=cds2788	0	2	STM14_2880	STM14_2880	hypothetical protein	0,284200331	-1,182723423
NC_01685(	RefSeq	CDS	2499320	2499454	+	0	ID=cds2789	1	3	STM14_2881	STM14_2881	hypothetical protein	2,185622867	1,419750352
NC_01685(	RefSeq	CDS	2499491	2500693	+	0	ID=cds2790	0	162,5	STM14_2882	ackA	acetate kinase	23,02022682	-1,883139852
NC_01685(	RefSeq	CDS	2500770	2502914	+	0	ID=cds2791	1	167	STM14_2883	pta	phosphate acetyltransferase	25,49005001	-2,406240177
NC_01685(	RefSeq	CDS	2503132	2504652	+	0	ID=cds2792	0	7,5	STM14_2884	yfcC	hypothetical protein	1,136801324	-2,147403465
NC_01685(	RefSeq	CDS	2504701	2505654	-	0	ID=cds2793	0	14	STM14_2885	STM14_2885	putative transketolase	1,989402318	-2,376535557
NC_01685(	RefSeq	CDS	2505647	2506477	-	0	ID=cds2794	0	15	STM14_2886	STM14_2886	putative transketolase	2,131502483	-2,392032191
NC_01685(	RefSeq	CDS	2506474	2507871	-	0	ID=cds2795	0	9,5	STM14_2887	ulaA_2	PTS system ascorbate-specific transporter subunit IIC	1,421001655	-2,256999172
NC_01685(	RefSeq	CDS	2507886	2508158	-	0	ID=cds2796	0	5	STM14_2888	STM14_2888	putative cytoplasmic protein	0,710500828	-1,864806942
NC_01685(	RefSeq	CDS	2508239	2508682	-	0	ID=cds2797	0	3,5	STM14_2889	STM14_2889	putative PTS system transporter subunit A component	0,568400662	-1,710438693
NC_01685(	RefSeq	CDS	2508681	2508854	+	0	ID=cds2798	0	0	STM14_2890	STM14_2890	hypothetical protein	0	NA
NC_01685(	RefSeq	CDS	2508935	2509954	+	0	ID=cds2799	0	13	STM14_2891	STM14_2891	putative transcriptional regulator	1,847302152	-2,356210635
NC_01685(	RefSeq	CDS	2509960	2510514	-	0	ID=cds2800	0	8	STM14_2892	STM14_2892	putative NTP pyrophosphohydrolase	1,136801324	-2,147403465
NC_01685(	RefSeq	CDS	2510571	2511122	-	0	ID=cds2801	0	18,5	STM14_2893	yfcE	phosphodiesterase	2,55780298	-2,419166771
NC_01685(	RefSeq	CDS	2511178	2511822	-	0	ID=cds2802	0	4,5	STM14_2894	yfcF	putative glutathione S-transferase	0,568400662	-1,710438693
NC_01685(	RefSeq	CDS	2511965	2512612	+	0	ID=cds2803	0	0	STM14_2895	yfcG	putative glutathione S-transferase	0	NA
NC_01685(	RefSeq	CDS	2512738	2513631	+	0	ID=cds2804	0	8,5	STM14_2896	yfcH	putative sugar nucleotide epimerase	1,136801324	-2,147403465
NC_01685(	RefSeq	CDS	2513683	2514459	-	0	ID=cds2805	0	5	STM14_2897	hisP	histidine%2Flysine%2Farginine%2Fornithine transporter s	0,710500828	-1,864806942
NC_01685(	RefSeq	CDS	2514470	2515177	-	0	ID=cds2806	0	7	STM14_2898	hisM	histidine%2Flysine%2Farginine%2Fornithine transport pro	0,994701159	-2,072978628
NC_01685(	RefSeq	CDS	2515174	2515860	-	0	ID=cds2807	0	5,5	STM14_2899	hisQ	histidine%2Flysine%2Farginine%2Fornithine transport pro	0,852600993	-1,98056697
NC_01685(	RefSeq	CDS	2516043	2516825	-	0	ID=cds2808	0	16	STM14_2900	hisJ	histidine transport protein	2,273602649	-2,403729808
NC_01685(	RefSeq	CDS	2517063	2517845	-	0	ID=cds2809	0	1	STM14_2901	argT	lysine%2Farginine%2Fornithine transport protein	0,142100166	-0,623264422
NC_01685(	RefSeq	CDS	2517875	2518045	+	0	ID=cds2810	0	0	STM14_2902	STM14_2902	hypothetical protein	0	NA
NC_01685(	RefSeq	CDS	2518134	2518703	-	0	ID=cds2811	0	4	STM14_2903	ubiX	3-octaprenyl-4-hydroxybenzoate carboxy-lyase	0,568400662	-1,710438693
NC_01685(	RefSeq	CDS	2518730	2520136	-	0	ID=cds2812	0	12	STM14_2904	STM14_2904	putative amino acid transporter	1,705201986	-2,329925387
NC_01685(	RefSeq	CDS	2520200	2521303	-	0	ID=cds2813	0	6,5	STM14_2905	STM14_2905	putative cytoplasmic protein	0,852600993	-1,98056697
NC_01685(	RefSeq	CDS	2521305	2522726	-	0	ID=cds2814	0	0,5	STM14_2906	STM14_2906	putative amino acid transporter	0	NA
NC_01685(	RefSeq	CDS	2522817	2524214	-	0	ID=cds2815	0	0	STM14_2907	STM14_2907	putative diaminopimelate decarboxylase	0	NA
NC_01685(	RefSeq	CDS	2524425	2525852	+	0	ID=cds2816	0	3	STM14_2908	STM14_2908	putative regulatory protein	0,426300497	-1,498211557
NC_01685(	RefSeq	CDS	2525888	2527405	-	0	ID=cds2817	2	10	STM14_2909	purF	amidophosphoribosyltransferase	4,939646395	0,828530271
NC_01685(	RefSeq	CDS	2527443	2527931	-	0	ID=cds2818	2	12,5	STM14_2910	cvpA	colicin V production protein	5,223846726	0,666065355
NC_01685(	RefSeq	CDS	2527947	2528102	-	0	ID=cds2819	0	7,5	STM14_2911	STM14_2911	hypothetical protein	1,136801324	-2,147403465
NC_01685(	RefSeq	CDS	2528242	2528916	-	0	ID=cds2820	0	10,5	STM14_2912	dedD	hypothetical protein	1,421001655	-2,256999172
NC_01685(	RefSeq	CDS	2528906	2530174	-	0	ID=cds2821	0	22,5	STM14_2913	folC	bifunctional folylpolyglutamate synthase%2Fdihydrofolate	3,126203642	-2,431590384
NC_01685(	RefSeq	CDS	2530242	2531156	-	0	ID=cds2822	1	43,5	STM14_2914	accD	acetyl-CoA carboxylase subunit beta	8,011729654	-1,15351741
NC_01685(	RefSeq	CDS	2531306	2531965	-	0	ID=cds2823	1	17,5	STM14_2915	dedA	hypothetical protein	4,31712535	-0,335890347
NC_01685(	RefSeq	CDS	2532014	2532826	-	0	ID=cds2824	1	16,5	STM14_2916	truA	tRNA pseudouridine synthase A	4,032925019	-0,231745061
NC_01685(	RefSeq	CDS	2532826	2533839	-	0	ID=cds2825	0	24,5	STM14_2917	usg	putative semialdehyde dehydrogenase	3,410403973	-2,434086198
NC_01685(	RefSeq	CDS	2533907	2535043	-	0	ID=cds2826	0	24	STM14_2918	pdxB	erythronate-4-phosphate dehydrogenase	3,410403973	-2,434086198
NC_01685(	RefSeq	CDS	2535147	2536148	+	0	ID=cds2827	0	2	STM14_2919	flk	flagella biosynthesis regulator	0,284200331	-1,182723423
NC_01685(	RefSeq	CDS	2536145	2537323	-	0	ID=cds2828	0	5,5	STM14_2920	STM14_2920	hypothetical protein	0,852600993	-1,98056697
NC_01685(	RefSeq	CDS	2537322	2537438	+	0	ID=cds2829	0	0	STM14_2921	STM14_2921	hypothetical protein	0	NA
NC_01685(	RefSeq	CDS	2537503	2537877	-	0	ID=cds2830	0	0	STM14_2922	STM14_2922	putative cytoplasmic protein	0	NA
NC_01685(	RefSeq	CDS	2538050	2538298	+	0	ID=cds2831	0	0	STM14_2923	STM14_2923	putative regulatory protein	0	NA
NC_01685(	RefSeq	CDS	2538467	2538835	+	0	ID=cds2832	0	0	STM14_2924	STM14_2924	putative cytoplasmic protein	0	NA
NC_01685(	RefSeq	CDS	2538835	2539353	+	0	ID=cds2833	0	1	STM14_2925	STM14_2925	hypothetical protein	0,142100166	-0,623264422
NC_01685(	RefSeq	CDS	2539420	2540076	-	0	ID=cds2834	1	4	STM14_2926	STM14_2926	putative inner membrane protein	2,327723032	1,125268706
NC_01685(	RefSeq	CDS	2540174	2541388	-	0	ID=cds2835	0	196	STM14_2927	fabB	3-oxoacyl-ACP synthase	27,85163245	-1,899063699
NC_01685(	RefSeq	CDS	2541548	2543548	+	0	ID=cds2836	0	21	STM14_2928	mnmC	5-methylaminomethyl-2-thiouridine methyltransferase	2,984103476	-2,430148754
NC_01685(	RefSeq	CDS	2543600	2543875	-	0	ID=cds2837	0	3,5	STM14_2929	yfcL	putative cytoplasmic protein	0,568400662	-1,710438693
NC_01685(	RefSeq	CDS	2543908	2544456	-	0	ID=cds2838	0	8	STM14_2930	yfcM	putative cytoplasmic protein	1,136801324	-2,147403465
NC_01685(	RefSeq	CDS	2544456	2545265	-	0	ID=cds2839	0	11	STM14_2931	yfcA	hypothetical protein	1,563101821	-2,297202414
NC_01685(	RefSeq	CDS	2545265	2546089	-	0	ID=cds2840	0	6,833333333	STM14_2932	mepA	penicillin-insensitive murein endopeptidase	0,994701159	-2,072978628
NC_01685(	RefSeq	CDS	2546093	2547178	-	0	ID=cds2841	0	18,5	STM14_2933	aroC	chorismate synthase	2,55780298	-2,419166771
NC_01685(	RefSeq	CDS	2547214	2548146	-	0	ID=cds2842	0	4,5	STM14_2934	yfcB	N5-glutamine S-adenosyl-L-methionine-dependent methy	0,568400662	-1,710438693
NC_01685(	RefSeq	CDS	2548312	2548863	+	0	ID=cds2843	0	3,5	STM14_2935	yfcN	hypothetical protein	0,568400662	-1,710438693
NC_01685(	RefSeq	CDS	2548963	2549448	-	0	ID=cds2844	1	36	STM14_2936	sixA	phosphohistidine phosphatase	6,874928329	-0,96718351
NC_01685(	RefSeq	CDS	2549657	2551804	-	0	ID=cds2845	0	12	STM14_2937	fadJ	multifunctional fatty acid oxidation complex subunit alpha	1,705201986	-2,329925387
NC_01685(	RefSeq	CDS	2551804	2553114	-	0	ID=cds2846	0	1,5	STM14_2938	fadI	3-ketoacyl-CoA thiolase	0,284200331	-1,182723423
NC_01685(	RefSeq	CDS	2553292	2553576	-	0	ID=cds2847	0	85	STM14_2939	yfcZ	putative cytoplasmic protein	12,07851407	-1,968565518
NC_01685(	RefSeq	CDS	2553683	2553823	+	0	ID=cds2848	0	1	STM14_2940	STM14_2940	hypothetical protein	0,142100166	-0,623264422
NC_01685(	RefSeq	CDS	2553820	2553948	-	0	ID=cds2849	0	0	STM14_2941	STM14_2941	hypothetical protein	0	NA
NC_01685(	RefSeq	CDS	2553941	2555254	+	0	ID=cds2850	0	5	STM14_2942	fadL	long-chain fatty acid outer membrane transporter	0,710500828	-1,864806942
NC_01685(	RefSeq	CDS	2555315	2556166	-	0	ID=cds2851	0	21,5	STM14_2943	STM14_2943	hypothetical protein	3,126203642	-2,431590384
NC_01685(	RefSeq	CDS	2556359	2557300	+	0	ID=cds2852	0	0	STM14_2944	yfdC	hypothetical protein	0	NA
NC_01685(	RefSeq	CDS	2557607	2558545	-	0	ID=cds2853	0	6	STM14_2946	pgtE	outer membrane protease	0,852600993	-1,98056697
NC_01685(	RefSeq	CDS	2558813	2560060	-	0	ID=cds2854	0	16,75	STM14_2947	pgtA	activator	2,415702814	-2,412509737
NC_01685(	RefSeq	CDS	2560050	2562056	-	0	ID=cds2855	0	17,75	STM14_2948	pgtB	phosphoglycerate transport system sensor protein	2,55780298	-2,419166771
NC_01685(	RefSeq	CDS	2562053	2563363	-	0	ID=cds2856	0	16	STM14_2949	pgtC	phosphoglycerate transport regulatory protein precursor	2,273602649	-2,403729808
NC_01685(	RefSeq	CDS	2563682	2565073	+	0	ID=cds2857	0	1	STM14_2950	pgtP	transporter	0,142100166	-0,623264422
NC_01685(	RefSeq	CDS	2565349	2565591	-	0	ID=cds2858	0	4,5	STM14_2951	STM14_2951	putative inner membrane protein	0,568400662	-1,710438693
NC_01685(	RefSeq	CDS	2565752	2565865	-	0	ID=cds2859	0	0	STM14_2952	STM14_2952	hypothetical protein	0	NA
NC_01685(	RefSeq	CDS	2566113	2567033	+	0	ID=cds2860	0	90	STM14_2953	ddg	lipid A biosynthesis palmitoleoyl acyltransferase	12,7890149	-1,945533971
NC_01685(	RefSeq	CDS	2567557	2568795	-	0	ID=cds2861	0	13	STM14_2954	yfdZ	aminotransferase	1,847302152	-2,356210635
NC_01685(	RefSeq	CDS	2569137	2569283	-	0	ID=cds2862	0	0,5	STM14_2955	STM14_2955	hypothetical protein	0	NA
NC_01685(	RefSeq	CDS	2569516	2570481	-	0	ID=cds2863	0	24	STM14_2956	glk	glucokinase	3,410403973	-2,434086198
NC_01685(	RefSeq	CDS	2570685	2571920	+	0	ID=cds2864	0	5,5	STM14_2957	STM14_2957	hypothetical protein	0,852600993	-1,98056697
NC_01685(	RefSeq	CDS	2571940	2573592	-	0	ID=cds2865	0	1	STM14_2958	STM14_2958	indolepyruvate decarboxylase	0,142100166	-0,623264422

NC_01685	RefSeq	CDS	2573587	2573775	+	0	ID=cds286	0	0	STM14_2959	STM14_2959	hypothetical protein	0	NA
NC_01685	RefSeq	CDS	2573772	2574770	+	0	ID=cds286	0	6,5	STM14_2960	STM14_2960	putative oxidoreductase	0,852600993	-1,98056697
NC_01685	RefSeq	CDS	2574884	2575210	+	0	ID=cds286	0	4,5	STM14_2961	ypeC	hypothetical protein	0,568400662	-1,710438693
NC_01685	RefSeq	CDS	2575271	2576512	-	0	ID=cds286	0	4	STM14_2962	mntH	manganese transport protein MntH	0,568400662	-1,710438693
NC_01685	RefSeq	CDS	2576855	2578057	+	0	ID=cds287	0	11,5	STM14_2963	nupC	nucleoside transport	1,705201986	-2,329925387
NC_01685	RefSeq	CDS	2578110	2580299	-	0	ID=cds287	0	18,5	STM14_2964	yfeA	hypothetical protein	2,55780298	-2,419166771
NC_01685	RefSeq	CDS	2580905	2581267	+	0	ID=cds287	0	19	STM14_2967	yfeC	putative negative regulator	2,699903145	-2,42419778
NC_01685	RefSeq	CDS	2581269	2581661	+	0	ID=cds287	0	32	STM14_2968	yfeD	putative negative regulator	4,547205297	-2,489878815
NC_01685	RefSeq	CDS	2581715	2583130	-	0	ID=cds287	0	54,5	STM14_2969	gltX	glutamyl-tRNA synthetase	7,673408939	-2,144619715
NC_01685	RefSeq	CDS	2583946	2584830	-	0	ID=cds287	0	1	STM14_2974	xapR	DNA-binding transcriptional activator XapR	0,142100166	-0,623264422
NC_01685	RefSeq	CDS	2584882	2585040	-	0	ID=cds287	0	0	STM14_2975	STM14_2975	hypothetical protein	0	NA
NC_01685	RefSeq	CDS	2585099	2586355	-	0	ID=cds287	0	2	STM14_2976	xapB	xanthosine permease	0,284200331	-1,182723423
NC_01685	RefSeq	CDS	2586410	2587333	-	0	ID=cds287	0	0	STM14_2977	STM14_2977	xanthosine phosphorylase	0	NA
NC_01685	RefSeq	CDS	2587499	2588260	+	0	ID=cds287	0	0	STM14_2978	yfeN	hypothetical protein	0	NA
NC_01685	RefSeq	CDS	2588322	2589248	-	0	ID=cds288	0	0	STM14_2979	yfeR	putative transcriptional regulator	0	NA
NC_01685	RefSeq	CDS	2589338	2590336	+	0	ID=cds288	0	3	STM14_2980	yfeH	putative Na%2B-dependent transporter	0,426300497	-1,498211557
NC_01685	RefSeq	CDS	2590333	2590560	-	0	ID=cds288	0	1,5	STM14_2981	STM14_2981	putative cytoplasmic protein	0,284200331	-1,182723423
NC_01685	RefSeq	CDS	2590553	2592568	-	0	ID=cds288	0	30	STM14_2982	ligA	NAD-dependent DNA ligase LigA	4,263004966	-2,464060063
NC_01685	RefSeq	CDS	2592640	2593626	-	0	ID=cds288	1	25	STM14_2983	zipA	cell division protein ZipA	5,311826508	-0,631843652
NC_01685	RefSeq	CDS	2593747	2594619	+	0	ID=cds288	0	7	STM14_2984	cysZ	putative sulfate transport protein CysZ	0,994701159	-2,072978628
NC_01685	RefSeq	CDS	2594783	2595754	+	0	ID=cds288	0	14,5	STM14_2985	cysK	cysteine synthase A	1,989402318	-2,376535557
NC_01685	RefSeq	CDS	2595762	2595923	+	0	ID=cds288	0	5,583333333	STM14_2986	STM14_2986	hypothetical protein	0,852600993	-1,98056697
NC_01685	RefSeq	CDS	2595873	2595998	-	0	ID=cds288	0	0	STM14_2987	STM14_2987	hypothetical protein	0	NA
NC_01685	RefSeq	CDS	2595966	2596094	+	0	ID=cds288	0	2,583333333	STM14_2988	STM14_2988	hypothetical protein	0,426300497	-1,498211557
NC_01685	RefSeq	CDS	2596138	2596395	+	0	ID=cds289	0	76	STM14_2989	ptsH	phosphohistidinoprotein-hexose phosphotransferase com	10,79961258	-2,01367454
NC_01685	RefSeq	CDS	2596444	2598171	+	0	ID=cds289	2	267,75	STM14_2990	ptsI	phosphoenolpyruvate-protein phosphotransferase	41,6014891	-2,280359379
NC_01685	RefSeq	CDS	2598212	2598721	+	0	ID=cds289	0	142,25	STM14_2991	crr	glucose-specific PTS system component	20,17822351	-1,870713222
NC_01685	RefSeq	CDS	2598870	2599109	-	0	ID=cds289	4	0	STM14_2992	STM14_2992	putative cytoplasmic protein	7,03728948	1,955434289
NC_01685	RefSeq	CDS	2599106	2599972	-	0	ID=cds289	0	3	STM14_2993	pdxK	pyridoxal kinase	0,426300497	-1,498211557
NC_01685	RefSeq	CDS	2600055	2601347	+	0	ID=cds289	0	1	STM14_2994	ptsJ	putative regulatory protein	0,142100166	-0,623264422
NC_01685	RefSeq	CDS	2601362	2602081	+	0	ID=cds289	0	1,5	STM14_2995	yfeJ	glutamine amidotransferase	0,284200331	-1,182723423
NC_01685	RefSeq	CDS	2602140	2602520	+	0	ID=cds289	0	2	STM14_2996	yfeK	hypothetical protein	0,284200331	-1,182723423
NC_01685	RefSeq	CDS	2602533	2603072	+	0	ID=cds289	0	4,5	STM14_2997	yfeL	putative membrane carboxypeptidase	0,568400662	-1,710438693
NC_01685	RefSeq	CDS	2603203	2604114	-	0	ID=cds289	0	6	STM14_2998	cysM	cysteine synthase B	0,852600993	-1,98056697
NC_01685	RefSeq	CDS	2604182	2605279	-	0	ID=cds290	0	0,25	STM14_2999	cysA	sulfate%2Fthiosulfate transporter subunit	0	NA
NC_01685	RefSeq	CDS	2605269	2606144	-	0	ID=cds290	0	0,25	STM14_3000	cysW	sulfate%2Fthiosulfate transporter permease subunit	0	NA
NC_01685	RefSeq	CDS	2606144	2606977	-	0	ID=cds290	0	1	STM14_3001	cysU	sulfate%2Fthiosulfate transporter subunit	0,142100166	-0,623264422
NC_01685	RefSeq	CDS	2606977	2607993	-	0	ID=cds290	0	2,5	STM14_3002	cysP	thiosulfate transporter subunit	0,284200331	-1,182723423
NC_01685	RefSeq	CDS	2608151	2608942	-	0	ID=cds290	0	107,5	STM14_3003	ucpA	short chain dehydrogenase	15,34681788	-1,876042944
NC_01685	RefSeq	CDS	2609180	2610079	-	0	ID=cds290	4	68,5	STM14_3004	STM14_3004	putative iron-dependent peroxidase	16,70010074	-0,318210415
NC_01685	RefSeq	CDS	2610174	2610749	-	0	ID=cds290	0	23,5	STM14_3005	STM14_3005	hypothetical protein	3,410403973	-2,434086198
NC_01685	RefSeq	CDS	2610811	2611260	-	0	ID=cds290	0	7	STM14_3006	yfeZ	putative inner membrane protein	0,994701159	-2,072978628
NC_01685	RefSeq	CDS	2611247	2611672	-	0	ID=cds290	0	5	STM14_3007	STM14_3007	putative acetyltransferase	0,710500828	-1,864806942
NC_01685	RefSeq	CDS	2611885	2612754	+	0	ID=cds290	0	11	STM14_3008	amiA	N-acetylmuramoyl-l-alanine amidase I	1,563101821	-2,297202414
NC_01685	RefSeq	CDS	2612757	2613656	+	0	ID=cds291	0	13	STM14_3009	hemF	coproporphyrinogen III oxidase	1,847302152	-2,356210635
NC_01685	RefSeq	CDS	2613677	2614006	+	0	ID=cds291	0	3,5	STM14_3010	STM14_3010	putative inner membrane protein	0,568400662	-1,710438693
NC_01685	RefSeq	CDS	2614135	2615412	+	0	ID=cds291	0	4	STM14_3011	STM14_3011	putative cytoplasmic protein	0,568400662	-1,710438693
NC_01685	RefSeq	CDS	2615612	2616664	-	0	ID=cds291	0	11	STM14_3012	eutR	transcriptional regulator EutR	1,563101821	-2,297202414
NC_01685	RefSeq	CDS	2616712	2617206	-	0	ID=cds291	0	14	STM14_3013	eutK	putative carboxysome structural protein	1,989402318	-2,376535557
NC_01685	RefSeq	CDS	2617219	2617878	-	0	ID=cds291	0	12,25	STM14_3014	eutL	putative carboxysome structural protein	1,705201986	-2,329925387
NC_01685	RefSeq	CDS	2617888	2618784	-	0	ID=cds291	0	19,25	STM14_3015	eutC	ethanolamine ammonia-lyase small subunit	2,699903145	-2,42419778
NC_01685	RefSeq	CDS	2618803	2620164	-	0	ID=cds291	0	21	STM14_3016	eutB	ethanolamine ammonia-lyase heavy chain	2,984103476	-2,430148754
NC_01685	RefSeq	CDS	2620176	2621579	-	0	ID=cds291	0	16	STM14_3017	eutA	reactivating factor for ethanolamine ammonia lyase	2,273602649	-2,403729808
NC_01685	RefSeq	CDS	2621576	2622802	-	0	ID=cds291	0	16,83333333	STM14_3018	eutH	putative transport protein	2,415702814	-2,412509737
NC_01685	RefSeq	CDS	2622922	2624109	-	0	ID=cds292	0	14	STM14_3019	eutG	putative transport protein	1,989402318	-2,376535557
NC_01685	RefSeq	CDS	2624099	2624938	-	0	ID=cds292	0	8,5	STM14_3020	eutJ	ethanolamine utilization protein	1,136801324	-2,147403465
NC_01685	RefSeq	CDS	2624949	2626352	-	0	ID=cds292	0	34	STM14_3021	eutE	putative aldehyde oxidoreductase	4,831405628	-2,523251848
NC_01685	RefSeq	CDS	2626364	2626651	-	0	ID=cds292	0	26,5	STM14_3022	eutN	putative detox protein	3,694604304	-2,438835558
NC_01685	RefSeq	CDS	2626764	2627054	-	0	ID=cds292	0	24	STM14_3023	eutM	putative detox protein	3,410403973	-2,434086198
NC_01685	RefSeq	CDS	2627095	2628111	-	0	ID=cds292	0	36,5	STM14_3024	eutD	phosphotransacetylase	5,115605959	-2,281753063
NC_01685	RefSeq	CDS	2628108	2628911	-	0	ID=cds292	0	29,5	STM14_3025	eutT	putative cobalamin adenosyltransferase	4,263004966	-2,464060063
NC_01685	RefSeq	CDS	2628908	2629597	-	0	ID=cds292	0	22	STM14_3026	eutQ	putative ethanolamine utilization protein	3,126203642	-2,431590384
NC_01685	RefSeq	CDS	2629575	2630054	-	0	ID=cds292	0	37,5	STM14_3027	eutP	putative ethanolamine utilization protein	5,39980629	-2,264331336
NC_01685	RefSeq	CDS	2630067	2630402	-	0	ID=cds292	0	18,5	STM14_3028	eutS	putative carboxysome structural protein	2,55780298	-2,419166771
NC_01685	RefSeq	CDS	2630883	2631341	-	0	ID=cds293	0	2,9	STM14_3029	tnpA_8	transposase	0,426300497	-1,498211557
NC_01685	RefSeq	CDS	2631298	2631486	-	0	ID=cds293	0	1,85	STM14_3030	STM14_3030	hypothetical protein	0,284200331	-1,182723423
NC_01685	RefSeq	CDS	2631531	2633810	-	0	ID=cds293	0	108,75	STM14_3031	maeB	malic enzyme	15,48891804	-1,872366033
NC_01685	RefSeq	CDS	2633812	2634012	+	0	ID=cds293	0	0	STM14_3032	STM14_3032	hypothetical protein	0	NA
NC_01685	RefSeq	CDS	2634082	2635032	+	0	ID=cds293	0	2	STM14_3033	talA	transaldolase A	0,284200331	-1,182723423
NC_01685	RefSeq	CDS	2635052	2637052	+	0	ID=cds293	0	3	STM14_3034	tktB	transketolase	0,426300497	-1,498211557
NC_01685	RefSeq	CDS	2637115	2637345	-	0	ID=cds293	0	11,5	STM14_3035	STM14_3035	putative cytoplasmic protein	1,705201986	-2,329925387
NC_01685	RefSeq	CDS	2637342	2637476	-	0	ID=cds293	0	2,5	STM14_3036	STM14_3036	hypothetical protein	0,284200331	-1,182723423
NC_01685	RefSeq	CDS	2637473	2638516	-	0	ID=cds293	3	1	STM14_3037	ypfG	hypothetical protein	5,420067275	2,935327108
NC_01685	RefSeq	CDS	2638641	2639216	-	0	ID=cds293	0	10,5	STM14_3038	yffH	putative pyrophosphohydrolase	1,421001655	-2,256999172
NC_01685	RefSeq	CDS	2639420	2640718	+	0	ID=cds294	0	7	STM14_3039	STM14_3039	hypothetical protein	0,994701159	-2,072978628
NC_01685	RefSeq	CDS	2640806	2640913	+	0	ID=cds294	0	0,285714286	STM14_3040	STM14_3040	hypothetical protein	0	NA
NC_01685	RefSeq	CDS	2641167	2643128	-	0	ID=cds294	0	11	STM14_3041	aegA	putative oxidoreductase Fe-S binding subunit	1,563101821	-2,297202414
NC_01685	RefSeq	CDS	2643314	2645014	+	0	ID=cds294	0	8,5	STM14_3042	narQ	nitrate%2Fnitrite sensor protein NarQ	1,136801324	-2,147403465
NC_01685	RefSeq	CDS	2645096	2645227	-	0	ID=cds294	0	0	STM14_3043	STM14_3043	hypothetical protein	0	NA
NC_01685	RefSeq	CDS	2645199	2648312	+	0	ID=cds294	0	11	STM14_3044	acrD	aminoglycoside%2Fmultidrug efflux system	1,563101821	-2,297202414
NC_01685	RefSeq	CDS	2648886	2649242	+	0	ID=cds294	0	7	STM14_3045	yffB	hypothetical protein	0,994701159	-2,072978628
NC_01685	RefSeq	CDS	2649246	2650373	+	0	ID=cds294	0	29	STM14_3046	dapE	succinyl-diaminopimelate desuccinylase	4,120904801	-2,455089955
NC_01685	RefSeq	CDS	2650401	2650601	+	0	ID=cds294	0	5,75	STM14_3047	STM14_3047	hypothetical protein	0,852600993	-1,98056697
NC_01685	RefSeq	CDS	2650628	2652646	-	0	ID=cds294	0	11,5	STM14_3048	ypfI	putative acetyltransferase	1,705201986	-2,329925387
NC_01685	RefSeq	CDS	2652662	2653525	-	0	ID=cds295	0	13,5	STM14_3049	STM14_3049	putative inner membrane protein	1,989402318	-2,376535557
NC_01685	RefSeq	CDS	2653656	2654369	-	0	ID=cds295	0	17,5	STM14_3050	purC	phosphoribosylaminoimidazolesuccinocarboxamide synth	2,55780298	-2,419166771
NC_01685	RefSeq	CDS	2654544	2655578	-	0	ID=cds295	0	90	STM14_3051	nlpB	lipoprotein	12,7890149	-1,945533971
NC_01685	RefSeq	CDS	2655595	2656473	-	0	ID=cds295	1	73,83333333	STM14_3052	dapA	dihydrodipicolinate synthase	12,27473462	-1,663602209
NC_01685	RefSeq	CDS	2656619	2657191	+	0	ID=cds295	1	19	STM14_3053	gcvR	glycine cleavage system transcriptional repressor	4,459225515	-0,383770206
NC_01685	RefSeq	CDS	2657191	2657661	+	0	ID=cds295	3	26	STM14_3054	bcp	thioredoxin-dependent thiol peroxidase	8,972571414	0,342003337
NC_01685	RefSeq	CDS	2657708	2658061	-	0	ID=cds295	0	2	STM14_3055	STM14_3055	putative glycerate kinase	0,284200331	-1,182723423
NC_01685	RefSeq	CDS	2657883	2658191	-	0	ID=cds295	0	2,5	STM14_3056	STM14_3056	hypothetical protein	0,284200331	-1,182723423
NC_01685	RefSeq	CDS	2658248	2659315	-	0	ID=cds295	0	13,5	STM14_3057	perM	putative permease	1,989402318	-2,376535557
NC_01685	RefSeq	CDS	2659523	2660986	+	0	ID=cds295	0	18	STM14_3058	STM14_3058	hypothetical protein	2,55780298	-2,419166771
NC_01685	RefSeq	CDS	2661026	2661385	+	0	ID=cds296	0	9,5	STM14_3059	yfgD	putative arsenate reductase	1,421001655	-2,256999172
NC_01685	RefSeq	CDS	2661412	2662137	-	0	ID=cds296	0	25,83333333	STM14_3060	yfgE	DNA replication initiation factor	3,694604304	-2,438835558
NC_01685	RefSeq	CDS	2662208	2663497	-	0	ID=cds296	2	5,5	STM14_3061	uraA	uracil transporter	4,371245733	1,278786496
NC_01685	RefSeq	CDS	2663585	2664211	-	0	ID=cds296	0	14	STM14_3062	upp	uracil phosphoribosyltransferase	1,989402318	-2,376535557
NC_01685	RefSeq	CDS	2664349	2664552	-	0	ID=cds296	0	1	STM14_3063	STM14_3063	hypothetical protein	0,142100166	-0,623264422
NC_01685	RefSeq	CDS	2664610	2665662	+	0	ID=cds296	0	16,5	STM14_3064	purM	phosphoribosylaminoimidazole synthetase	2,273602649	-2,403729808
NC_01685	RefSeq	CDS	2665662	2666300	+	0	ID=cds296	1	14,5	STM14_3065	purN	phosphoribosylglycinamide formyltransferase	3,748724687	-0,112256387
NC_01685	RefSeq	CDS	2666489	2668555	+	0	ID=cds296	0	38,83333333	STM14_3066	ppk	polyphosphate kinase	5,541906456	-2,25579729
NC_01685	RefSeq	CDS	2668560	2670101	+	0	ID=cds296	0	20	STM14_3067	ppx	exopolyphosphatase	2,842003311	-2,427810709
NC_01685	RefSeq	CDS	2670137	2672350	-	0	ID=cds296	1	10	STM14_3068	STM14_3068	putative diguanylate cyclase	3,180324025	0,201203536
NC_01685	RefSeq	CDS	2672447	2672584	-	0	ID=cds297	0	0	STM14_3069	STM14_3069	putative cytoplasmic protein	0	NA
NC_01685	RefSeq	CDS	2672655	2672915	-	0	ID=cds297	0	0	STM14_3070	STM14_3070	putative inner membrane protein	0	NA
NC_01685	RefSeq	CDS	2672763	2672954	+	0	ID=cds297	0,5	4,5	STM14_3071	STM14_3071	putative inner membrane protein	0,568400662	-1,710438693
NC_01685	RefSeq	CDS	2673033	2673269	-	0	ID=cds297	0	0	STM14_3072	STM14_3072	putative inner membrane protein	0	NA
NC_01685	RefSeq	CDS	2673510	2673824	+	0	ID=cds297	0	1	STM14_3073	STM14_3073	putative cytoplasmic protein	0,142100166	-0,623264422
NC_01685	RefSeq	CDS	2673844	2674200	-	0	ID=cds297	0	0,5	STM14_3074	STM14_3074	putative transposase	0	NA
NC_01685	RefSeq	CDS	2674357	2675934	-	0	ID=cds297	2	70	STM14_3075	guaA	bifunctional GMP synthase%2Fglutamine amidotransferas	13,46565633	-1,012041739
NC_01685	RefSeq	CDS	2676004	2677554	-	0	ID=cds297	0	44	STM14_3076	guaB	inositol-5-monophosphate dehydrogenase	6,252407284	-2,216733793
NC_01685	RefSeq	CDS	2677631	2678980	+	0	ID=cds297	0	29,5	STM14_3077	xseA	exodeoxyribonuclease VII large subunit	4,263004966	-2,464060063
NC_01685	RefSeq	CDS	2679142	2685111	-	0	ID=cds297	2	27,33333333	STM14_3078	shdA	AIDA autotransporter-like protein	7,355349209	-0,081275219
NC_01685	RefSeq	CDS	2685671	2685784	+	0	ID=cds298	0	0	STM14_3079	STM14_3079	hypothetical protein	0	NA
NC_01685	RefSeq	CDS	2685675	2685857	+	0	ID=cds298	0	0	STM14_3080	STM14_3080	hypothetical protein	0	NA
NC_01685	RefSeq	CDS	2685854	2685967	-	0	ID=cds298	0	0	STM14_3081	STM14_3081	hypothetical protein	0	NA
NC_01685	RefSeq	CDS	2693427	2699024	-	0	ID=cds298	0	24,5	STM14_3083	ratA	putative outer membrane protein	3,410403973	-2,434086198
NC_01685	RefSeq	CDS	2699144	2700103	-	0	ID=cds298	0	0,5	STM14_3084	sinI	putative outer membrane protein	0	NA
NC_01685	RefSeq	CDS	2700161	2702353	-	0	ID=cds298	0	21,83333333	STM14_3085	sinH	intimin-like protein	3,126203642	-2,431590384
NC_01685	RefSeq	CDS	2702354	2702470	+	0	ID=cds298	0	0	STM14_3086	STM14_3086	hypothetical protein	0	NA
NC_01685	RefSeq	CDS	2702529	2702684	-	0	ID=cds298	0	0,5	STM14_3087	STM14_3087	hypothetical protein	0	NA
NC_01685	RefSeq	CDS	2702699	2702920	-	0	ID=cds298	0	3	STM14_3088	yfgJ	putative cytoplasmic protein	0,426300497	-1,498211557
NC_01685	RefSeq	CDS	2703009	2704481	-	0	ID=cds298	1	22,5	STM14_3089	engA	GTP-binding protein EngA	4,885526012	-0,515045314

NC_01685(	RefSeq	CDS	2704480	2704581	+	0	ID=cds2990	0	0	STM14_3090	STM14_3090	hypothetical protein	0	NA
NC_01685(	RefSeq	CDS	2704600	2705778	-	0	ID=cds2991	2	117,75	STM14_3091	yfgL	outer membrane protein assembly complex subunit YfgL	20,28646427	-1,522855417
NC_01685(	RefSeq	CDS	2705789	2706409	-	0	ID=cds2992	0	45,75	STM14_3092	yfgM	putative inner membrane protein	6,536607615	-2,201017198
NC_01685(	RefSeq	CDS	2706423	2707697	-	0	ID=cds2993	1	84	STM14_3093	hisS	histidyl-tRNA synthetase	13,69573627	-1,78951192
NC_01685(	RefSeq	CDS	2707808	2708926	-	0	ID=cds2994	1	58,5	STM14_3094	ispG	4-hydroxy-3-methylbut-2-en-1-yl diphosphate synthase	10,00113197	-1,420921355
NC_01685(	RefSeq	CDS	2708953	2709957	-	0	ID=cds2995	0	44	STM14_3095	yfgA	hypothetical protein	6,252407284	-2,216733793
NC_01685(	RefSeq	CDS	2710094	2710216	-	0	ID=cds2996	0	7	STM14_3096	STM14_3096	type IV pilus biogenesis%2Fstability protein PilW	0,994701159	-2,072978628
NC_01685(	RefSeq	CDS	2710249	2711415	-	0	ID=cds2997	0	29,5	STM14_3097	yfgB	hypothetical protein	4,263004966	-2,464060063
NC_01685(	RefSeq	CDS	2711622	2712053	-	0	ID=cds2998	0,5	7	STM14_3098	ndk	nucleoside diphosphate kinase	0,994701159	-2,072978628
NC_01685(	RefSeq	CDS	2712052	2712147	+	0	ID=cds2999	0	0	STM14_3099	STM14_3099	hypothetical protein	0	NA
NC_01685(	RefSeq	CDS	2712174	2713037	-	0	ID=cds3000	0	12,5	STM14_3100	STM14_3100	putative polyferredoxin	1,705201986	-2,329925387
NC_01685(	RefSeq	CDS	2713037	2713846	-	0	ID=cds3001	0	8,25	STM14_3101	STM14_3101	putative dimethylsulfoxide reductase	1,136801324	-2,147403465
NC_01685(	RefSeq	CDS	2713839	2714468	-	0	ID=cds3002	0	3,75	STM14_3102	STM14_3102	putative anaerobic dimethylsulfoxide reductase	0,568400662	-1,710438693
NC_01685(	RefSeq	CDS	2714465	2716870	-	0	ID=cds3003	8,5	20,83333333	STM14_3103	STM14_3103	putative anaerobic dimethylsulfoxide reductase	17,05868244	1,539731261
NC_01685(	RefSeq	CDS	2716999	2719314	-	0	ID=cds3004	0	12	STM14_3104	pbpC	penicillin-binding protein 1C	1,705201986	-2,329925387
NC_01685(	RefSeq	CDS	2719315	2724249	-	0	ID=cds3005	0	106,8333333	STM14_3105	STM14_3105	putative inner membrane lipoprotein	15,20471771	-1,879758932
NC_01685(	RefSeq	CDS	2724458	2725300	+	0	ID=cds3006	0	21	STM14_3106	sseA	3-mercaptopyruvate sulfurtransferase	2,984103476	-2,430148754
NC_01685(	RefSeq	CDS	2725280	2725504	-	0	ID=cds3007	0	0	STM14_3107	STM14_3107	hypothetical protein	0	NA
NC_01685(	RefSeq	CDS	2725547	2726215	+	0	ID=cds3008	0	0	STM14_3108	STM14_3108	putative cytoplasmic protein	0	NA
NC_01685(	RefSeq	CDS	2726253	2726387	-	0	ID=cds3009	0	5,5	STM14_3109	STM14_3109	hypothetical protein	0,852600993	-1,98056697
NC_01685(	RefSeq	CDS	2726784	2727569	-	0	ID=cds3010	0,5	20	STM14_3110	sseB	enhanced serine sensitivity protein SseB	2,842003311	-2,427810709
NC_01685(	RefSeq	CDS	2727670	2728953	-	0	ID=cds3011	0	39	STM14_3111	pepB	aminopeptidase B	5,541906456	-2,25579729
NC_01685(	RefSeq	CDS	2729200	2729400	-	0	ID=cds3012	0	12,5	STM14_3112	yfhJ	hypothetical protein	1,705201986	-2,329925387
NC_01685(	RefSeq	CDS	2729412	2729747	-	0	ID=cds3013	1,5	16	STM14_3113	fdx	electron carrer protein	5,792247388	0,405774795
NC_01685(	RefSeq	CDS	2729749	2731599	-	0	ID=cds3014	1,5	23	STM14_3114	hscA	chaperone protein HscA	6,786948547	0,069120359
NC_01685(	RefSeq	CDS	2731612	2732127	-	0	ID=cds3015	0	16,83333333	STM14_3115	hscB	co-chaperone HscB	2,415702814	-2,412509737
NC_01685(	RefSeq	CDS	2732323	2732646	-	0	ID=cds3016	9	31,33333333	STM14_3116	iscA	iron-sulfur cluster assembly protein	20,23900646	1,280699961
NC_01685(	RefSeq	CDS	2732675	2733061	-	0	ID=cds3017	0	26,5	STM14_3117	nifU	scaffold protein	3,694604304	-2,438835558
NC_01685(	RefSeq	CDS	2733089	2734303	-	0	ID=cds3018	2	64	STM14_3118	nifS	cysteine desulfurase	12,61305533	-0,922025439
NC_01685(	RefSeq	CDS	2734356	2734475	+	0	ID=cds3019	0	0	STM14_3119	STM14_3119	hypothetical protein	0	NA
NC_01685(	RefSeq	CDS	2734484	2734978	-	0	ID=cds3020	4	36	STM14_3120	yfhP	DNA-binding transcriptional regulator IscR	12,15289544	0,31489869
NC_01685(	RefSeq	CDS	2735138	2735869	-	0	ID=cds3021	1	49,08333333	STM14_3121	STM14_3121	putative rRNA methylase	8,722230481	-1,256284002
NC_01685(	RefSeq	CDS	2735856	2735978	+	0	ID=cds3022	0	2,166666667	STM14_3122	STM14_3122	hypothetical protein	0,284200331	-1,182723423
NC_01685(	RefSeq	CDS	2735883	2735993	-	0	ID=cds3023	0	17,25	STM14_3123	STM14_3123	hypothetical protein	2,415702814	-2,412509737
NC_01685(	RefSeq	CDS	2735988	2736791	+	0	ID=cds3024	0	25	STM14_3124	suhB	inositol monophosphatase	3,552504138	-2,43600272
NC_01685(	RefSeq	CDS	2736936	2737814	+	0	ID=cds3025	0	2	STM14_3125	STM14_3125	putative hydrolase	0,284200331	-1,182723423
NC_01685(	RefSeq	CDS	2737996	2739039	+	0	ID=cds3026	0	3,5	STM14_3126	asrA	anaerobic sulfide reductase	0,568400662	-1,710438693
NC_01685(	RefSeq	CDS	2739043	2739861	+	0	ID=cds3027	0	4	STM14_3127	asrB	anaerobic sulfite reductase subunit B	0,568400662	-1,710438693
NC_01685(	RefSeq	CDS	2739872	2740885	+	0	ID=cds3028	0	5	STM14_3128	asrC	anaerobic sulfide reductase	0,710500828	-1,864806942
NC_01685(	RefSeq	CDS	2740886	2741872	-	0	ID=cds3029	0	4	STM14_3129	STM14_3129	putative inner membrane protein	0,568400662	-1,710438693
NC_01685(	RefSeq	CDS	2741863	2742501	-	0	ID=cds3030	0	0	STM14_3130	STM14_3130	hypothetical protein	0	NA
NC_01685(	RefSeq	CDS	2742627	2743904	+	0	ID=cds3031	0	4,5	STM14_3131	csiE	stationary phase inducible protein CsiE	0,568400662	-1,710438693
NC_01685(	RefSeq	CDS	2743899	2745038	-	0	ID=cds3032	0	12	STM14_3132	hcaT	putative 3-phenylpropionic acid transporter	1,705201986	-2,329925387
NC_01685(	RefSeq	CDS	2745234	2746487	-	0	ID=cds3033	0	8456,666667	STM14_3133	glyA	serine hydroxymethyltransferase	1201,7471	-2,186700207
NC_01685(	RefSeq	CDS	2746625	2746771	-	0	ID=cds3034	0	4,5	STM14_3134	STM14_3134	hypothetical protein	0,568400662	-1,710438693
NC_01685(	RefSeq	CDS	2746812	2748002	+	0	ID=cds3035	0	0	STM14_3135	hmpA	nitric oxide dioxygenase	0	NA
NC_01685(	RefSeq	CDS	2748184	2749728	+	0	ID=cds3036	0	38,5	STM14_3136	cadC	DNA-binding transcriptional activator CadC	5,39980629	-2,264331336
NC_01685(	RefSeq	CDS	2750089	2751420	+	0	ID=cds3037	0	170,3333333	STM14_3137	cadB	lysine%2Fcadaverine antiporter	24,15702814	-1,887389583
NC_01685(	RefSeq	CDS	2751503	2753647	+	0	ID=cds3038	0	368,5	STM14_3138	cadA	lysine decarboxylase 1	52,29286092	-1,937309877
NC_01685(	RefSeq	CDS	2753703	2755163	+	0	ID=cds3039	0	20	STM14_3139	yjdL	putative di-%2Ftripeptide transport protein	2,842003311	-2,427810709
NC_01685(	RefSeq	CDS	2755212	2755550	-	0	ID=cds3040	0	16,25	STM14_3140	glnB	nitrogen regulatory protein P-II 1	2,273602649	-2,403729808
NC_01685(	RefSeq	CDS	2755627	2756964	-	0	ID=cds3041	0	34,5	STM14_3141	yfhA	putative transcriptional regulator	4,831405628	-2,523251848
NC_01685(	RefSeq	CDS	2756961	2757725	-	0	ID=cds3042	0	10	STM14_3142	yfhG	hypothetical protein	1,421001655	-2,256999172
NC_01685(	RefSeq	CDS	2757727	2759169	-	0	ID=cds3043	0	9	STM14_3143	yfhK	putative sensor kinase	1,27890149	-2,207741391
NC_01685(	RefSeq	CDS	2759420	2759548	+	0	ID=cds3044	0	0	STM14_3144	STM14_3144	hypothetical protein	0	NA
NC_01685(	RefSeq	CDS	2759807	2763694	-	0	ID=cds3045	0	21	STM14_3145	purG	phosphoribosylformylglycinamidine synthase	2,984103476	-2,430148754
NC_01685(	RefSeq	CDS	2763716	2763949	+	0	ID=cds3046	0	0,5	STM14_3146	STM14_3146	hypothetical protein	0	NA
NC_01685(	RefSeq	CDS	2763950	2765494	+	0	ID=cds3047	0	5,5	STM14_3147	yfhD	putative transglycosylase	0,852600993	-1,98056697
NC_01685(	RefSeq	CDS	2765545	2766096	-	0	ID=cds3048	0	5	STM14_3148	yfhC	tRNA-specific adenosine deaminase	0,710500828	-1,864806942
NC_01685(	RefSeq	CDS	2766121	2766756	-	0	ID=cds3049	0	11	STM14_3149	yfhB	hypothetical protein	1,563101821	-2,297202414
NC_01685(	RefSeq	CDS	2766760	2768121	-	0	ID=cds3050	0	11	STM14_3150	STM14_3150	putative phosphotransferase system IIB component	1,563101821	-2,297202414
NC_01685(	RefSeq	CDS	2768132	2769025	-	0	ID=cds3051	0	5,5	STM14_3151	murQ	N-acetylmuramic acid 6-phosphate etherase	0,852600993	-1,98056697
NC_01685(	RefSeq	CDS	2769141	2769989	+	0	ID=cds3052	0	2,5	STM14_3152	yfhH	putative DNA-binding transcriptional regulator	0,284200331	-1,182723423
NC_01685(	RefSeq	CDS	2770028	2770945	-	0	ID=cds3053	0	1	STM14_3153	STM14_3153	2-dehydropantoate 2-reductase	0,142100166	-0,623264422
NC_01685(	RefSeq	CDS	2770967	2772163	-	0	ID=cds3054	0	0	STM14_3154	STM14_3154	putative permease	0	NA
NC_01685(	RefSeq	CDS	2772279	2773205	+	0	ID=cds3055	0	1	STM14_3155	STM14_3155	putative transcriptional regulator	0,142100166	-0,623264422
NC_01685(	RefSeq	CDS	2773243	2773503	+	0	ID=cds3056	1	2	STM14_3156	yfhL	putative ferredoxin	2,043522701	1,824239109
NC_01685(	RefSeq	CDS	2773615	2773995	-	0	ID=cds3057	0	7,25	STM14_3157	acpS	4%27-phosphopantetheinyl transferase	0,994701159	-2,072978628
NC_01685(	RefSeq	CDS	2773995	2774726	-	0	ID=cds3058	0	3,25	STM14_3158	pdxJ	pyridoxal phosphate biosynthetic protein PdxJ	0,426300497	-1,498211557
NC_01685(	RefSeq	CDS	2774738	2775466	-	0	ID=cds3059	0	22,5	STM14_3159	recO	DNA repair protein RecO	3,126203642	-2,431590384
NC_01685(	RefSeq	CDS	2775478	2776383	-	0	ID=cds3060	0	50	STM14_3160	era	GTP-binding protein Era	7,105008277	-2,173389122
NC_01685(	RefSeq	CDS	2776380	2777060	-	0	ID=cds3061	0	19	STM14_3161	rnc	ribonuclease III	2,699903145	-2,42419778
NC_01685(	RefSeq	CDS	2777334	2778308	-	0	ID=cds3062	0	35	STM14_3162	lepB	signal peptidase I	4,973505794	-2,290637485
NC_01685(	RefSeq	CDS	2778325	2780124	-	0	ID=cds3063	1	54	STM14_3163	lepA	GTP-binding protein LepA	9,432731309	-1,350661723
NC_01685(	RefSeq	CDS	2780529	2782022	+	0	ID=cds3064	0	4	STM14_3164	gogB	hypothetical protein	0,568400662	-1,710438693
NC_01685(	RefSeq	CDS	2782226	2782354	-	0	ID=cds3065	0	0	STM14_3165	STM14_3165	hypothetical protein	0	NA
NC_01685(	RefSeq	CDS	2782885	2783430	+	0	ID=cds3066	2	4,5	STM14_3166	STM14_3166	transposase-like protein	4,087045402	1,631390028
NC_01685(	RefSeq	CDS	2784542	2784769	+	0	ID=cds3067	0	2,5	STM14_3167	STM14_3167	PagK-like protein	0,284200331	-1,182723423
NC_01685(	RefSeq	CDS	2784866	2785444	-	0	ID=cds3068	0	1	STM14_3168	STM14_3168	phage tail assembly-like protein	0,142100166	-0,623264422
NC_01685(	RefSeq	CDS	2785434	2786258	-	0	ID=cds3069	0	1	STM14_3169	STM14_3169	phage tail assembly-like protein	0,142100166	-0,623264422
NC_01685(	RefSeq	CDS	2786255	2788627	-	0	ID=cds3070	0	3	STM14_3170	STM14_3170	tail fiber-like protein	0,426300497	-1,498211557
NC_01685(	RefSeq	CDS	2788681	2788923	-	0	ID=cds3071	0	2,5	STM14_3171	STM14_3171	hypothetical protein	0,284200331	-1,182723423
NC_01685(	RefSeq	CDS	2788962	2792324	-	0	ID=cds3072	0	3,5	STM14_3172	STM14_3172	host specificity protein-J-like protein	0,568400662	-1,710438693
NC_01685(	RefSeq	CDS	2792386	2793033	-	0	ID=cds3073	0	1,25	STM14_3173	STM14_3173	tail assembly protein I-like protein	0,142100166	-0,623264422
NC_01685(	RefSeq	CDS	2792931	2793668	-	0	ID=cds3074	0	1,75	STM14_3174	STM14_3174	tail assembly protein K-like protein	0,284200331	-1,182723423
NC_01685(	RefSeq	CDS	2793675	2794373	-	0	ID=cds3075	0	4	STM14_3175	STM14_3175	phage tail component L-like protein	0,568400662	-1,710438693
NC_01685(	RefSeq	CDS	2794383	2794712	-	0	ID=cds3076	0	0,5	STM14_3176	STM14_3176	phage tail component M-like protein	0	NA
NC_01685(	RefSeq	CDS	2794715	2797810	-	0	ID=cds3077	0	14,33333333	STM14_3177	STM14_3177	phage tail component H-like protein	1,989402318	-2,376535557
NC_01685(	RefSeq	CDS	2797782	2798120	-	0	ID=cds3078	0	0	STM14_3178	STM14_3178	minor tail-like protein	0	NA
NC_01685(	RefSeq	CDS	2798117	2798512	-	0	ID=cds3079	0	0	STM14_3179	STM14_3179	minor tail-like protein	0	NA
NC_01685(	RefSeq	CDS	2798563	2799306	-	0	ID=cds3080	0	10,33333333	STM14_3180	STM14_3180	major tail-like protein	1,421001655	-2,256999172
NC_01685(	RefSeq	CDS	2799317	2799718	-	0	ID=cds3081	0	0	STM14_3181	STM14_3181	hypothetical protein	0	NA
NC_01685(	RefSeq	CDS	2799827	2800957	+	0	ID=cds3082	0	17,5	STM14_3182	STM14_3182	putative virulence protein	2,55780298	-2,419166771
NC_01685(	RefSeq	CDS	2801006	2801584	-	0	ID=cds3083	0	0,75	STM14_3183	STM14_3183	minor tail protein Z-like protein	0,142100166	-0,623264422
NC_01685(	RefSeq	CDS	2801612	2801995	-	0	ID=cds3084	0	2,75	STM14_3184	STM14_3184	minor capsid protein FII	0,426300497	-1,498211557
NC_01685(	RefSeq	CDS	2802428	2803456	-	0	ID=cds3085	4	2	STM14_3186	STM14_3186	phage head-like protein	7,321489811	2,765358925
NC_01685(	RefSeq	CDS	2803511	2803858	-	0	ID=cds3086	0	0,5	STM14_3187	STM14_3187	phage head-like protein	0	NA
NC_01685(	RefSeq	CDS	2803871	2805367	-	0	ID=cds3087	0	1	STM14_3188	STM14_3188	head-tail preconnector-like protein	0,142100166	-0,623264422
NC_01685(	RefSeq	CDS	2805357	2806937	-	0	ID=cds3088	0	4,25	STM14_3189	STM14_3189	head-tail preconnector-like protein	0,568400662	-1,710438693
NC_01685(	RefSeq	CDS	2806934	2807137	-	0	ID=cds3089	0	0,25	STM14_3190	STM14_3190	head-to-tail joining-like protein	0	NA
NC_01685(	RefSeq	CDS	2807121	2809052	-	0	ID=cds3090	0	16,5	STM14_3191	STM14_3191	terminase-like large protein	2,273602649	-2,403729808
NC_01685(	RefSeq	CDS	2809024	2809569	-	0	ID=cds3091	0	2,5	STM14_3192	STM14_3192	DNA packaging-like protein	0,284200331	-1,182723423
NC_01685(	RefSeq	CDS	2809856	2810257	+	0	ID=cds3092	0	1	STM14_3193	STM14_3193	hypothetical protein	0,142100166	-0,623264422
NC_01685(	RefSeq	CDS	2810493	2810966	-	0	ID=cds3093	0	0	STM14_3194	STM14_3194	endopeptidase-like protein	0	NA
NC_01685(	RefSeq	CDS	2810963	2811415	-	0	ID=cds3094	0	0	STM14_3195	STM14_3195	morphogenesis-like protein	0	NA
NC_01685(	RefSeq	CDS	2811399	2811728	-	0	ID=cds3095	0	0,5	STM14_3196	STM14_3196	hypothetical protein	0	NA
NC_01685(	RefSeq	CDS	2811771	2811908	+	0	ID=cds3096	0	0	STM14_3197	STM14_3197	hypothetical protein	0	NA
NC_01685(	RefSeq	CDS	2812004	2812690	+	0	ID=cds3097	0	2	STM14_3198	STM14_3198	hypothetical protein	0,284200331	-1,182723423
NC_01685(	RefSeq	CDS	2812905	2813093	-	0	ID=cds3098	0	0	STM14_3199	STM14_3199	hypothetical protein	0	NA
NC_01685(	RefSeq	CDS	2813600	2814163	-	0	ID=cds3099	0	0	STM14_3201	STM14_3201	antirepressor-like protein	0	NA
NC_01685(	RefSeq	CDS	2814160	2814285	-	0	ID=cds3100	0	1,5	STM14_3202	STM14_3202	hypothetical protein	0,284200331	-1,182723423
NC_01685(	RefSeq	CDS	2814436	2815113	-	0	ID=cds3101	0	2,5	STM14_3203	STM14_3203	antiterminator-like protein	0,284200331	-1,182723423
NC_01685(	RefSeq	CDS	2815110	2815250	-	0	ID=cds3102	0	0	STM14_3204	STM14_3204	hypothetical protein	0	NA
NC_01685(	RefSeq	CDS	2815247	2815858	-	0	ID=cds3103	0	0	STM14_3205	STM14_3205	hypothetical protein	0	NA
NC_01685(	RefSeq	CDS	2815861	2816061	-	0	ID=cds3104	0	0,5	STM14_3206	STM14_3206	hypothetical protein	0	NA
NC_01685(	RefSeq	CDS	2816067	2816669	-	0	ID=cds3105	0	7,5	STM14_3207	STM14_3207	hypothetical protein	1,136801324	-2,147403465
NC_01685(	RefSeq	CDS	2816704	2816883	-	0	ID=cds3106	0	2,5	STM14_3208	STM14_3208	putative bacteriophage protein	0,284200331	-1,182723423
NC_01685(	RefSeq	CDS	2816906	2817040	+	0	ID=cds3107	0	0	STM14_3209	STM14_3209	prophage DinI	0	NA
NC_01685(	RefSeq	CDS	2817069	2817302	-	0	ID=cds3108	0	0	STM14_3210	STM14_3210	hypothetical protein	0	NA
NC_01685(	RefSeq	CDS	2817545	2818177	-	0	ID=cds3109	0	16	STM14_3211	STM14_3211	hypothetical protein	2,273602649	-2,403729808
NC_01685(	RefSeq	CDS	2818285	2818983	-	0	ID=cds3110	0	1,5	STM14_3212	STM14_3212	Eaa1	0,284200331	-1,182723423
NC_01685(	RefSeq	CDS	2818997	2819692	-	0	ID=cds3111	0	0	STM14_3213	STM14_3213	phage replication protein P	0	NA
NC_01685(	RefSeq	CDS	2819689	2820573	-	0	ID=cds3112	0	2	STM14_3214	STM14_3214	phage replication protein O	0,284200331	-1,182723423
NC_01685(	RefSeq	CDS	2820665	2821039	-	0	ID=cds3113	0	0	STM14_3215	STM14_3215	prophage cI protein	0	NA

NC_01685(	RefSeq	CDS	2820999	2821259	-	0	ID=cds3114	0	0,5	STM14_3216	STM14_3216	hypothetical protein	0	NA
NC_01685(	RefSeq	CDS	2821341	2821736	+	0	ID=cds3115	0	29,83333333	STM14_3217	STM14_3217	putative regulator	4,263004966	-2,464060063
NC_01685(	RefSeq	CDS	2821795	2822634	+	0	ID=cds3116	0	30	STM14_3218	STM14_3218	chromosome partitioning ATPase	4,263004966	-2,464060063
NC_01685(	RefSeq	CDS	2822627	2823013	+	0	ID=cds3117	0	4	STM14_3219	STM14_3219	hypothetical protein	0,568400662	-1,710438693
NC_01685(	RefSeq	CDS	2823013	2823675	+	0	ID=cds3118	0	18,5	STM14_3220	STM14_3220	hypothetical protein	2,55780298	-2,419166771
NC_01685(	RefSeq	CDS	2824017	2824139	+	0	ID=cds3119	0	0	STM14_3221	STM14_3221	Gifsy-1 prophage protein	0	NA
NC_01685(	RefSeq	CDS	2824132	2824290	+	0	ID=cds3120	0	0	STM14_3222	STM14_3222	hypothetical protein	0	NA
NC_01685(	RefSeq	CDS	2824312	2824662	+	0	ID=cds3121	0	0	STM14_3223	STM14_3223	hypothetical protein	0	NA
NC_01685(	RefSeq	CDS	2824789	2827716	+	0	ID=cds3122	0	1	STM14_3224	STM14_3224	exodeoxyribonuclease VIII-like protein	0,142100166	-0,623264422
NC_01685(	RefSeq	CDS	2827727	2828836	+	0	ID=cds3123	0	1,5	STM14_3225	STM14_3225	enterohemolysin 1-like protein	0,284200331	-1,182723423
NC_01685(	RefSeq	CDS	2828879	2829118	+	0	ID=cds3124	0	0,5	STM14_3226	STM14_3226	putative cytoplasmic protein	0	NA
NC_01685(	RefSeq	CDS	2829159	2829443	+	0	ID=cds3125	0	1	STM14_3227	STM14_3227	excisionase-like protein	0,142100166	-0,623264422
NC_01685(	RefSeq	CDS	2829421	2830650	-	0	ID=cds3126	1	3,5	STM14_3228	STM14_3228	integrase-like protein	2,327723032	1,125268706
NC_01685(	RefSeq	CDS	2830868	2831005	+	0	ID=cds3127	0	0	STM14_3229	STM14_3229	hypothetical protein	0	NA
NC_01685(	RefSeq	CDS	2831002	2831118	-	0	ID=cds3128	0	3,75	STM14_3230	STM14_3230	hypothetical protein	0,568400662	-1,710438693
NC_01685(	RefSeq	CDS	2831148	2831627	-	0	ID=cds3129	0	20,75	STM14_3231	rseC	SoxR reducing system protein RseC	2,984103476	-2,430148754
NC_01685(	RefSeq	CDS	2831624	2832580	-	0	ID=cds3130	0	93,75	STM14_3232	rseB	periplasmic negative regulator of sigmaE	13,35741556	-1,928482877
NC_01685(	RefSeq	CDS	2832580	2833230	-	0	ID=cds3131	0	179,5	STM14_3233	rseA	anti-RNA polymerase sigma factor SigE	25,5780298	-1,892233448
NC_01685(	RefSeq	CDS	2833262	2833837	-	0	ID=cds3132	5,5	122,75	STM14_3234	rpoE	RNA polymerase sigma factor RpoE	28,03425458	-0,507636836
NC_01685(	RefSeq	CDS	2833834	2833998	-	0	ID=cds3133	5,5	28,25	STM14_3235	STM14_3235	hypothetical protein	14,53473885	0,972190407
NC_01685(	RefSeq	CDS	2833998	2834177	-	0	ID=cds3134	0	5,5	STM14_3236	STM14_3236	hypothetical protein	0,852600993	-1,98056697
NC_01685(	RefSeq	CDS	2834262	2835884	+	0	ID=cds3135	0	1,5	STM14_3237	nadB	L-aspartate oxidase	0,284200331	-1,182723423
NC_01685(	RefSeq	CDS	2835869	2836606	-	0	ID=cds3136	0	12,75	STM14_3238	yfiC	putative transferase	1,847302152	-2,356210635
NC_01685(	RefSeq	CDS	2836525	2836725	-	0	ID=cds3137	0	1,75	STM14_3239	STM14_3239	putative methyltransferase	0,284200331	-1,182723423
NC_01685(	RefSeq	CDS	2836737	2838071	+	0	ID=cds3138	0	22,5	STM14_3240	srmB	ATP-dependent RNA helicase SrmB	3,126203642	-2,431590384
NC_01685(	RefSeq	CDS	2838089	2838988	-	0	ID=cds3139	0	5	STM14_3241	yfiE	putative transcriptional regulator	0,710500828	-1,864806942
NC_01685(	RefSeq	CDS	2839091	2839678	+	0	ID=cds3140	0	0	STM14_3242	yfiK	neutral amino-acid efflux protein	0	NA
NC_01685(	RefSeq	CDS	2839740	2840123	-	0	ID=cds3141	0	122	STM14_3243	yfiD	autonomous glycyl radical cofactor GrcA	17,3362202	-1,854848636
NC_01685(	RefSeq	CDS	2840442	2841131	+	0	ID=cds3142	0	19	STM14_3244	ung	uracil-DNA glycosylase	2,699903145	-2,42419778
NC_01685(	RefSeq	CDS	2841247	2842284	-	0	ID=cds3143	0	12	STM14_3245	yfiF	putative methyltransferase	1,705201986	-2,329925387
NC_01685(	RefSeq	CDS	2842488	2842907	+	0	ID=cds3144	0	1,5	STM14_3246	trxC	thioredoxin 2	0,284200331	-1,182723423
NC_01685(	RefSeq	CDS	2842980	2843660	+	0	ID=cds3145	0	5,5	STM14_3247	yfiP	putative cytoplasmic protein	0,852600993	-1,98056697
NC_01685(	RefSeq	CDS	2843714	2846374	+	0	ID=cds3146	0	91,5	STM14_3248	yfiQ	putative acetyl-CoA synthetase	13,07321523	-1,936690345
NC_01685(	RefSeq	CDS	2846371	2846505	+	0	ID=cds3147	0	6,5	STM14_3249	STM14_3249	hypothetical protein	0,852600993	-1,98056697
NC_01685(	RefSeq	CDS	2846489	2847844	+	0	ID=cds3148	1	93,5	STM14_3250	pssA	phosphatidylserine synthase	15,11673793	-1,899073915
NC_01685(	RefSeq	CDS	2847889	2848212	+	0	ID=cds3149	0	7,5	STM14_3251	yfiM	hypothetical protein	1,136801324	-2,147403465
NC_01685(	RefSeq	CDS	2848209	2849510	-	0	ID=cds3150	0	6,5	STM14_3252	kgtP	alpha-ketoglutarate transporter	0,852600993	-1,98056697
NC_01685(	RefSeq	CDS	2849614	2850069	-	0	ID=cds3151	0	1	STM14_3253	STM14_3253	putative cytoplasmic protein	0,142100166	-0,623264422
NC_01685(	RefSeq	CDS	2855709	2855837	+	0	ID=cds3152	0	0	STM14_3259	STM14_3259	hypothetical protein	0	NA
NC_01685(	RefSeq	CDS	2855915	2856031	+	0	ID=cds3153	0	0	STM14_3260	STM14_3260	hypothetical protein	0	NA
NC_01685(	RefSeq	CDS	2856033	2858606	-	0	ID=cds3154	1	101,75	STM14_3261	clpB	protein disaggregation chaperone	16,25353925	-1,976443334
NC_01685(	RefSeq	CDS	2858736	2859467	-	0	ID=cds3155	7	21,83333333	STM14_3262	yfiH	hypothetical protein	15,44146023	1,361346995
NC_01685(	RefSeq	CDS	2859464	2860444	-	0	ID=cds3156	0	33,5	STM14_3263	rluD	23S rRNA pseudouridine synthase D	4,831405628	-2,523251848
NC_01685(	RefSeq	CDS	2860576	2861313	+	0	ID=cds3157	0	49,5	STM14_3264	yfiO	outer membrane protein assembly complex subunit YfiO	7,105008277	-2,173389122
NC_01685(	RefSeq	CDS	2861585	2861923	+	0	ID=cds3158	0	212	STM14_3266	yfiA	translation inhibitor protein RaiA	30,12523509	-1,904967369
NC_01685(	RefSeq	CDS	2861933	2862097	+	0	ID=cds3159	1	6,5	STM14_3267	STM14_3267	hypothetical protein	2,611923363	0,709005733
NC_01685(	RefSeq	CDS	2862174	2863334	+	0	ID=cds3160	0	5	STM14_3268	pheA	bifunctional chorismate mutase%2Fprephenate dehydrat	0,710500828	-1,864806942
NC_01685(	RefSeq	CDS	2863295	2864203	-	0	ID=cds3161	0	3,166666667	STM14_3269	STM14_3269	putative cytoplasmic protein	0,426300497	-1,498211557
NC_01685(	RefSeq	CDS	2864261	2865382	-	0	ID=cds3162	0	12	STM14_3270	tyrA	bifunctional chorismate mutase%2Fprephenate dehydrog	1,705201986	-2,329925387
NC_01685(	RefSeq	CDS	2865392	2866462	-	0	ID=cds3163	0	13	STM14_3271	aroF	phospho-2-dehydro-3-deoxyheptonate aldolase	1,847302152	-2,356210635
NC_01685(	RefSeq	CDS	2866577	2866735	+	0	ID=cds3164	0	0	STM14_3272	STM14_3272	hypothetical protein	0	NA
NC_01685(	RefSeq	CDS	2866637	2866750	-	0	ID=cds3165	0	0	STM14_3273	STM14_3273	hypothetical protein	0	NA
NC_01685(	RefSeq	CDS	2866902	2867420	+	0	ID=cds3166	1	15	STM14_3274	yfiR	hypothetical protein	3,890824853	-0,174265561
NC_01685(	RefSeq	CDS	2867413	2868633	+	0	ID=cds3167	0	6	STM14_3275	yfiN	hypothetical protein	0,852600993	-1,98056697
NC_01685(	RefSeq	CDS	2868790	2869137	-	0	ID=cds3168	2	61,25	STM14_3276	rplS	50S ribosomal protein L19	12,18675484	-0,873804631
NC_01685(	RefSeq	CDS	2869178	2869945	-	0	ID=cds3169	5	96,5	STM14_3277	trmD	tRNA %28guanine-N%281%29-%29-methyltransferase	22,43822774	-0,441873352
NC_01685(	RefSeq	CDS	2869990	2870538	-	0	ID=cds3170	1	104,25	STM14_3278	rimM	16S rRNA-processing protein	16,53773959	-1,994540578
NC_01685(	RefSeq	CDS	2870557	2870805	-	0	ID=cds3171	0	38,5	STM14_3279	rpsP	30S ribosomal protein S16	5,39980629	-2,264331336
NC_01685(	RefSeq	CDS	2870862	2871008	-	0	ID=cds3172	0	9,5	STM14_3280	STM14_3280	hypothetical protein	1,421001655	-2,256999172
NC_01685(	RefSeq	CDS	2871119	2872480	-	0	ID=cds3173	5	108,5833333	STM14_3281	ffh	signal recognition particle protein	24,28552989	-0,568824558
NC_01685(	RefSeq	CDS	2872506	2872700	+	0	ID=cds3174	0	2	STM14_3282	STM14_3282	putative cytochrome c-type biogenesis protein %28heme	0,284200331	-1,182723423
NC_01685(	RefSeq	CDS	2872646	2873437	+	0	ID=cds3175	0	24	STM14_3283	corE	hypothetical protein	3,410403973	-2,434086198
NC_01685(	RefSeq	CDS	2873547	2874743	+	0	ID=cds3176	0	19,5	STM14_3284	yfjD	hypothetical protein	2,842003311	-2,427810709
NC_01685(	RefSeq	CDS	2874864	2875469	+	0	ID=cds3177	0	6	STM14_3285	STM14_3285	putative cytoplasmic protein	0,852600993	-1,98056697
NC_01685(	RefSeq	CDS	2875504	2876094	-	0	ID=cds3178	0	26,83333333	STM14_3286	grpE	heat shock protein GrpE	3,836704469	-2,44262576
NC_01685(	RefSeq	CDS	2876217	2877095	+	0	ID=cds3179	0	8	STM14_3288	ppnK	inorganic polyphosphate%2FATP-NAD kinase	1,136801324	-2,147403465
NC_01685(	RefSeq	CDS	2877181	2878842	+	0	ID=cds3180	1	5,5	STM14_3289	recN	recombination and repair protein	2,611923363	0,709005733
NC_01685(	RefSeq	CDS	2878991	2879329	+	0	ID=cds3181	0	21	STM14_3290	smpA	hypothetical protein	2,984103476	-2,430148754
NC_01685(	RefSeq	CDS	2879362	2879469	-	0	ID=cds3182	0	1	STM14_3291	STM14_3291	hypothetical protein	0,142100166	-0,623264422
NC_01685(	RefSeq	CDS	2879495	2879785	-	0	ID=cds3183	0	4,5	STM14_3292	yfjF	hypothetical protein	0,568400662	-1,710438693
NC_01685(	RefSeq	CDS	2879775	2880251	-	0	ID=cds3184	0	28,5	STM14_3293	yfjG	hypothetical protein	3,978804635	-2,448045729
NC_01685(	RefSeq	CDS	2880401	2880883	+	0	ID=cds3185	0	13	STM14_3294	smpB	SsrA-binding protein	1,847302152	-2,356210635
NC_01685(	RefSeq	CDS	2881056	2881160	+	0	ID=cds3186	0	0	STM14_3295	STM14_3295	hypothetical protein	0	NA
NC_01685(	RefSeq	CDS	2881388	2881540	-	0	ID=cds3187	0	1	STM14_3296	STM14_3296	hypothetical protein	0,142100166	-0,623264422
NC_01685(	RefSeq	CDS	2893036	2894445	+	0	ID=cds3188	0	0	STM14_3298	STM14_3298	putative outer membrane efflux protein	0	NA
NC_01685(	RefSeq	CDS	2894442	2896622	+	0	ID=cds3189	0	0	STM14_3299	STM14_3299	putative ABC transporter transmembrane region	0	NA
NC_01685(	RefSeq	CDS	2896630	2897793	+	0	ID=cds3190	0	0	STM14_3300	STM14_3300	HlyD family secretion protein	0	NA
NC_01685(	RefSeq	CDS	2898012	2898236	-	0	ID=cds3191	0	0	STM14_3301	STM14_3301	hypothetical protein	0	NA
NC_01685(	RefSeq	CDS	2899686	2900279	+	0	ID=cds3192	0	9	STM14_3304	STM14_3304	hypothetical protein	1,27890149	-2,207741391
NC_01685(	RefSeq	CDS	2900669	2901862	+	0	ID=cds3193	0	16	STM14_3305	STM14_3305	putative cytoplasmic protein	2,273602649	-2,403729808
NC_01685(	RefSeq	CDS	2902197	2903024	-	0	ID=cds3194	0	8,5	STM14_3306	STM14_3306	putative cytoplasmic protein	1,136801324	-2,147403465
NC_01685(	RefSeq	CDS	2903475	2903690	+	0	ID=cds3195	0	0	STM14_3307	STM14_3307	putative cytoplasmic protein	0	NA
NC_01685(	RefSeq	CDS	2903726	2905795	+	0	ID=cds3196	0	15,5	STM14_3308	STM14_3308	putative inner membrane protein	2,273602649	-2,403729808
NC_01685(	RefSeq	CDS	2905915	2906040	+	0	ID=cds3197	0	0	STM14_3309	STM14_3309	hypothetical protein	0	NA
NC_01685(	RefSeq	CDS	2906298	2907581	+	0	ID=cds3198	0	19,5	STM14_3310	STM14_3310	putative ATPase	2,842003311	-2,427810709
NC_01685(	RefSeq	CDS	2907626	2908444	+	0	ID=cds3199	0	10	STM14_3311	STM14_3311	putative cytoplasmic protein	1,421001655	-2,256999172
NC_01685(	RefSeq	CDS	2908421	2908582	-	0	ID=cds3200	0	3	STM14_3312	STM14_3312	hypothetical protein	0,426300497	-1,498211557
NC_01685(	RefSeq	CDS	2908598	2908954	-	0	ID=cds3201	0	2	STM14_3313	STM14_3313	putative transcriptional regulator	0,284200331	-1,182723423
NC_01685(	RefSeq	CDS	2909049	2909333	+	0	ID=cds3202	0	0	STM14_3314	STM14_3314	putative cytoplasmic protein	0	NA
NC_01685(	RefSeq	CDS	2909446	2909967	+	0	ID=cds3203	0	0	STM14_3315	STM14_3315	putative PTS system glucitol%2Fsorbitol-specific enzyme I	0	NA
NC_01685(	RefSeq	CDS	2909964	2910338	+	0	ID=cds3204	0	0	STM14_3316	STM14_3316	putative glucitol-specific PTS enzyme III	0	NA
NC_01685(	RefSeq	CDS	2910335	2911315	+	0	ID=cds3205	0	1	STM14_3317	STM14_3317	putative glucitol-specific PTS enzyme III	0,142100166	-0,623264422
NC_01685(	RefSeq	CDS	2911326	2912339	+	0	ID=cds3206	0	4,166666667	STM14_3318	STM14_3318	putative dehydrogenase	0,568400662	-1,710438693
NC_01685(	RefSeq	CDS	2912634	2913836	+	0	ID=cds3207	0	3	STM14_3319	STM14_3319	putative hexulose 6 phosphate synthase	0,426300497	-1,498211557
NC_01685(	RefSeq	CDS	2913910	2914545	-	0	ID=cds3208	0	0	STM14_3320	STM14_3320	putative hexulose 6 phosphate synthase	0	NA
NC_01685(	RefSeq	CDS	2914569	2915132	-	0	ID=cds3209	0	0,5	STM14_3321	STM14_3321	putative sugar phosphate aminotransferase	0	NA
NC_01685(	RefSeq	CDS	2915132	2915974	-	0	ID=cds3210	0	0,5	STM14_3322	STM14_3322	putative cytoplasmic protein	0	NA
NC_01685(	RefSeq	CDS	2917712	2917867	-	0	ID=cds3211	0	0	STM14_3324	STM14_3324	hypothetical protein	0	NA
NC_01685(	RefSeq	CDS	2917868	2919547	+	0	ID=cds3212	0	4	STM14_3325	STM14_3325	putative dipeptide%2Foligopeptide%2Fnickel ABC-type tr	0,568400662	-1,710438693
NC_01685(	RefSeq	CDS	2919626	2919739	-	0	ID=cds3213	0	0	STM14_3326	STM14_3326	hypothetical protein	0	NA
NC_01685(	RefSeq	CDS	2920664	2921539	+	0	ID=cds3214	0	0	STM14_3327	STM14_3327	putative integrase	0	NA
NC_01685(	RefSeq	CDS	2921705	2923579	+	0	ID=cds3215	0	3	STM14_3328	STM14_3328	putative inner membrane protein	0,426300497	-1,498211557
NC_01685(	RefSeq	CDS	2923839	2925122	-	0	ID=cds3216	0	6,5	STM14_3329	STM14_3329	putative inner membrane protein	0,852600993	-1,98056697
NC_01685(	RefSeq	CDS	2925700	2926296	+	0	ID=cds3217	0	0	STM14_06470	STM14_06470	P4-type integrase	0	NA
NC_01685(	RefSeq	CDS	2926750	2927154	-	0	ID=cds3218	0	0	STM14_3331	STM14_3331	putative transposase	0	NA
NC_01685(	RefSeq	CDS	2927301	2927567	-	0	ID=cds3219	0	0	STM14_3332	STM14_3332	putative transposase	0	NA
NC_01685(	RefSeq	CDS	2928020	2928658	-	0	ID=cds3220	0	3	STM14_3333	STM14_3333	putative cytoplasmic protein	0,426300497	-1,498211557
NC_01685(	RefSeq	CDS	2928655	2930637	-	0	ID=cds3221	0	69,33333333	STM14_3334	STM14_3334	putative DNA%2FRNA helicase	9,804911422	-2,052421118
NC_01685(	RefSeq	CDS	2930916	2931215	+	0	ID=cds3222	0	1	STM14_3335	STM14_3335	putative transposase	0,142100166	-0,623264422
NC_01685(	RefSeq	CDS	2931212	2932078	+	0	ID=cds3223	0	1,5	STM14_3336	STM14_3336	putative transposase	0,284200331	-1,182723423
NC_01685(	RefSeq	CDS	2932858	2933397	-	0	ID=cds3224	0	0	STM14_3337	fljA	phase-1 flagellin repressor	0	NA
NC_01685(	RefSeq	CDS	2933465	2934985	-	0	ID=cds3225	2	85,83333333	STM14_3338	fljB	flagellin	15,73925898	-1,217602833
NC_01685(	RefSeq	CDS	2935077	2935649	+	0	ID=cds3226	0	0	STM14_3339	hin	DNA-invertase Hin	0	NA
NC_01685(	RefSeq	CDS	2935806	2935976	+	0	ID=cds3227	0	0	STM14_3340	STM14_3340	hypothetical protein	0	NA
NC_01685(	RefSeq	CDS	2936002	2936229	-	0	ID=cds3228	0	0	STM14_3341	STM14_3341	hypothetical protein	0	NA
NC_01685(	RefSeq	CDS	2936297	2936449	-	0	ID=cds3229	0	0	STM14_3342	STM14_3342	hypothetical protein	0	NA
NC_01685(	RefSeq	CDS	2936325	2936507	+	0	ID=cds3230	0	0	STM14_3343	STM14_3343	hypothetical protein	0	NA
NC_01685(	RefSeq	CDS	2936612	2937727	+	0	ID=cds3231	0	2	STM14_3344	iroB	putative glycosyl transferase	0,284200331	-1,182723423
NC_01685(	RefSeq	CDS	2937808	2941461	+	0	ID=cds3232	0	1	STM14_3345	iroC	putative ABC transporter protein	0,142100166	-0,623264422
NC_01685(	RefSeq	CDS	2941571	2942815	+	0	ID=cds3233	0	2	STM14_3346	iroD	enterochelin esterase%3D-like protein	0,284200331	-1,182723423
NC_01685(	RefSeq	CDS	2942847	2943782	+	0	ID=cds3234	0	0,5	STM14_3347	iroE	putative hydrolase	0	NA
NC_01685(	RefSeq	CDS	2943824	2946004	-	0	ID=cds3235	0	9,833333333	STM14_3348	iroN	outer membrane receptor FepA	1,421001655	-2,256999172
NC_01685(	RefSeq	CDS	2947037	2948089	-	0	ID=cds3236	16	25	STM14_3350	pipB2	secreted effector protein	31,70166206	2,031601715
NC_01685(	RefSeq	CDS	2948161	2948499	+	0	ID=cds3237	0	0	STM14_3351	STM14_3351	hypothetical protein	0	NA

NC_01685(	RefSeq	CDS	2948608	2949537	+	0	ID=cds3238	1	55,5	STM14_3352	virK	virulence protein	9,71693164	-1,386337946
NC_01685(	RefSeq	CDS	2949636	2949833	+	0	ID=cds3239	0	4,75	STM14_3353	STM14_3353	hypothetical protein	0,710500828	-1,864806942
NC_01685(	RefSeq	CDS	2949830	2950726	+	0	ID=cds3240	0	47,75	STM14_3354	mig-14	putative transcriptional activator	6,820807946	-2,185729286
NC_01685(	RefSeq	CDS	2950805	2950957	-	0	ID=cds3241	0	0	STM14_3355	STM14_3355	hypothetical protein	0	NA
NC_01685(	RefSeq	CDS	2951059	2951226	+	0	ID=cds3242	0	3,5	STM14_3356	STM14_3356	hypothetical protein	0,568400662	-1,710438693
NC_01685(	RefSeq	CDS	2951242	2951355	+	0	ID=cds3243	0	0	STM14_3357	STM14_3357	hypothetical protein	0	NA
NC_01685(	RefSeq	CDS	2951448	2952461	-	0	ID=cds3244	0	5,833333333	STM14_3358	nixA	putative nickel transporter	0,852600993	-1,98056697
NC_01685(	RefSeq	CDS	2952594	2954009	-	0	ID=cds3245	0	3	STM14_3359	tctE	regulatory protein	0,426300497	-1,498211557
NC_01685(	RefSeq	CDS	2953996	2954670	-	0	ID=cds3246	1	0	STM14_3360	tctD	regulatory protein	1,75932237	1,868512432
NC_01685(	RefSeq	CDS	2954825	2955802	+	0	ID=cds3247	0	2	STM14_3361	STM14_3361	tricarboxylic transport	0,284200331	-1,182723423
NC_01685(	RefSeq	CDS	2955814	2956248	+	0	ID=cds3248	0	1	STM14_3362	STM14_3362	tricarboxylic transport	0,142100166	-0,623264422
NC_01685(	RefSeq	CDS	2956259	2957773	+	0	ID=cds3249	0	5,5	STM14_3363	STM14_3363	tricarboxylic transport	0,852600993	-1,98056697
NC_01685(	RefSeq	CDS	2957937	2958050	+	0	ID=cds3250	0	0	STM14_3364	STM14_3364	hypothetical protein	0	NA
NC_01685(	RefSeq	CDS	2958076	2959053	+	0	ID=cds3251	0	1	STM14_3365	STM14_3365	hypothetical protein	0,142100166	-0,623264422
NC_01685(	RefSeq	CDS	2959079	2960347	+	0	ID=cds3252	0	0	STM14_3366	ygaF	hypothetical protein	0	NA
NC_01685(	RefSeq	CDS	2960369	2961817	+	0	ID=cds3253	0	1	STM14_3367	gabD	succinate-semialdehyde dehydrogenase I	0,142100166	-0,623264422
NC_01685(	RefSeq	CDS	2961832	2963115	+	0	ID=cds3254	0	3,5	STM14_3368	gabT	4-aminobutyrate aminotransferase	0,568400662	-1,710438693
NC_01685(	RefSeq	CDS	2963245	2964645	+	0	ID=cds3255	0	4,5	STM14_3369	gabP	gamma-aminobutyrate transporter	0,568400662	-1,710438693
NC_01685(	RefSeq	CDS	2964687	2965364	+	0	ID=cds3256	0	0	STM14_3370	ygaE	DNA-binding transcriptional regulator CsiR	0	NA
NC_01685(	RefSeq	CDS	2965386	2965835	-	0	ID=cds3257	0	2,75	STM14_3371	ygaU	hypothetical protein	0,426300497	-1,498211557
NC_01685(	RefSeq	CDS	2965822	2965938	+	0	ID=cds3258	0	0	STM14_3372	STM14_3372	hypothetical protein	0	NA
NC_01685(	RefSeq	CDS	2965935	2966093	-	0	ID=cds3259	0	0	STM14_3373	yqaE	putative transport protein	0	NA
NC_01685(	RefSeq	CDS	2966276	2966575	+	0	ID=cds3260	0	7	STM14_3374	STM14_3374	putative regulatory protein	0,994701159	-2,072978628
NC_01685(	RefSeq	CDS	2966585	2967112	+	0	ID=cds3261	2	18,5	STM14_3375	ygaP	putative rhodanese-like sulfurtransferase	6,07644772	0,297448745
NC_01685(	RefSeq	CDS	2967191	2967370	+	0	ID=cds3262	0	5	STM14_3376	STM14_3376	hypothetical protein	0,710500828	-1,864806942
NC_01685(	RefSeq	CDS	2967460	2967861	-	0	ID=cds3263	3	34,5	STM14_3377	stpA	DNA binding protein	10,10937274	0,085681013
NC_01685(	RefSeq	CDS	2968220	2968345	+	0	ID=cds3264	0	1	STM14_3378	STM14_3378	hypothetical protein	0,142100166	-0,623264422
NC_01685(	RefSeq	CDS	2968355	2968534	+	0	ID=cds3265	0	6	STM14_3379	STM14_3379	hypothetical protein	0,852600993	-1,98056697
NC_01685(	RefSeq	CDS	2968560	2969009	+	0	ID=cds3266	0	39,5	STM14_3380	STM14_3380	putative inner membrane protein	5,684006621	-2,24950002
NC_01685(	RefSeq	CDS	2969044	2969394	-	0	ID=cds3267	0	2	STM14_3381	ygaC	hypothetical protein	0,284200331	-1,182723423
NC_01685(	RefSeq	CDS	2969378	2969494	-	0	ID=cds3268	0	0,5	STM14_3382	STM14_3382	hypothetical protein	0	NA
NC_01685(	RefSeq	CDS	2969544	2969882	+	0	ID=cds3269	0	5	STM14_3383	ygaM	hypothetical protein	0,710500828	-1,864806942
NC_01685(	RefSeq	CDS	2969966	2971300	-	0	ID=cds3270	0	2	STM14_3384	STM14_3384	putative regulatory protein	0,284200331	-1,182723423
NC_01685(	RefSeq	CDS	2971389	2971820	+	0	ID=cds3271	0	0	STM14_3385	STM14_3385	putative cytoplasmic protein	0	NA
NC_01685(	RefSeq	CDS	2971817	2971993	+	0	ID=cds3272	0	0	STM14_3386	STM14_3386	hypothetical protein	0	NA
NC_01685(	RefSeq	CDS	2972092	2972337	+	0	ID=cds3273	0	0	STM14_3387	nrdH	glutaredoxin-like protein	0	NA
NC_01685(	RefSeq	CDS	2972334	2972744	+	0	ID=cds3274	0	0	STM14_3388	nrdI	ribonucleotide reductase stimulatory protein	0	NA
NC_01685(	RefSeq	CDS	2972717	2974861	+	0	ID=cds3275	0	0	STM14_3389	nrdE	ribonucleotide-diphosphate reductase subunit alpha	0	NA
NC_01685(	RefSeq	CDS	2974872	2975831	+	0	ID=cds3276	0	0	STM14_3390	nrdF	ribonucleotide-diphosphate reductase subunit beta	0	NA
NC_01685(	RefSeq	CDS	2976186	2977388	+	0	ID=cds3277	2	41	STM14_3391	proV	glycine betaine transporter ATP-binding subunit	9,344751527	-0,480390752
NC_01685(	RefSeq	CDS	2977381	2978445	+	0	ID=cds3278	0	22	STM14_3392	proW	glycine betaine transporter membrane protein	3,126203642	-2,431590384
NC_01685(	RefSeq	CDS	2978515	2979510	+	0	ID=cds3279	0	30	STM14_3393	proX	glycine betaine transporter periplasmic subunit	4,263004966	-2,464060063
NC_01685(	RefSeq	CDS	2979522	2979632	+	0	ID=cds3280	0	0	STM14_3394	STM14_3394	hypothetical protein	0	NA
NC_01685(	RefSeq	CDS	2979675	2980859	+	0	ID=cds3281	0	6	STM14_3395	STM14_3395	putative inner membrane protein	0,852600993	-1,98056697
NC_01685(	RefSeq	CDS	2981060	2981167	-	0	ID=cds3282	0	0	STM14_3396	STM14_3396	hypothetical protein	0	NA
NC_01685(	RefSeq	CDS	2981356	2981886	+	0	ID=cds3283	0	13,5	STM14_3397	emrR	transcriptional repressor MprA	1,989402318	-2,376535557
NC_01685(	RefSeq	CDS	2981900	2982022	-	0	ID=cds3284	0	0	STM14_3398	STM14_3398	hypothetical protein	0	NA
NC_01685(	RefSeq	CDS	2982013	2983185	+	0	ID=cds3285	2,5	23	STM14_3399	emrA	multidrug resistance secretion protein	6,786948547	0,069120359
NC_01685(	RefSeq	CDS	2983202	2984740	+	0	ID=cds3286	0	31,5	STM14_3400	emrB	putative multidrug transport protein	4,547205297	-2,489878815
NC_01685(	RefSeq	CDS	2984789	2986159	-	0	ID=cds3287	0	1	STM14_3401	STM14_3401	putative glycoporin	0,142100166	-0,623264422
NC_01685(	RefSeq	CDS	2986505	2987020	-	0	ID=cds3288	0	22	STM14_3402	luxS	S-ribosylhomocysteinase	3,126203642	-2,431590384
NC_01685(	RefSeq	CDS	2987170	2988726	-	0	ID=cds3289	0	49,33333333	STM14_3403	gshA	glutamate--cysteine ligase	6,962908111	-2,178239812
NC_01685(	RefSeq	CDS	2988803	2989228	-	0	ID=cds3290	0	18,25	STM14_3404	yqaA	putative inner membrane protein	2,55780298	-2,419166771
NC_01685(	RefSeq	CDS	2989225	2989791	-	0	ID=cds3291	0	65,25	STM14_3405	yqaB	fructose-1-phosphatase	9,23651076	-2,074135889
NC_01685(	RefSeq	CDS	2991063	2991173	+	0	ID=cds3292	0	1,5	STM14_3411	STM14_3411	hypothetical protein	0,284200331	-1,182723423
NC_01685(	RefSeq	CDS	2991186	2991371	-	0	ID=cds3293	0	32	STM14_3412	csrA	carbon storage regulator	4,547205297	-2,489878815
NC_01685(	RefSeq	CDS	2991606	2994236	-	0	ID=cds3294	0	148,6666667	STM14_3413	alaS	alanyl-tRNA synthetase	21,17292466	-1,8753915
NC_01685(	RefSeq	CDS	2994186	2994350	+	0	ID=cds3295	0	0	STM14_3414	STM14_3414	hypothetical protein	0	NA
NC_01685(	RefSeq	CDS	2994269	2994475	-	0	ID=cds3296	0	6,083333333	STM14_3415	STM14_3415	hypothetical protein	0,852600993	-1,98056697
NC_01685(	RefSeq	CDS	2994472	2994972	-	0	ID=cds3297	0	1,5	STM14_3416	recX	recombination regulator RecX	0,284200331	-1,182723423
NC_01685(	RefSeq	CDS	2995089	2996150	-	0	ID=cds3298	0	48	STM14_3417	recA	recombinase A	6,820807946	-2,185729286
NC_01685(	RefSeq	CDS	2996235	2996732	-	0	ID=cds3299	0	11,5	STM14_3418	ygaD	competence damage-inducible protein A	1,705201986	-2,329925387
NC_01685(	RefSeq	CDS	2996927	2997172	+	0	ID=cds3300	0	2,5	STM14_3419	STM14_3419	hypothetical protein	0,284200331	-1,182723423
NC_01685(	RefSeq	CDS	2997210	2998289	-	0	ID=cds3301	0	8	STM14_3420	mltB	murein hydrolase B	1,136801324	-2,147403465
NC_01685(	RefSeq	CDS	2998326	2998466	+	0	ID=cds3302	0	0	STM14_3421	STM14_3421	hypothetical protein	0	NA
NC_01685(	RefSeq	CDS	2998543	2999106	+	0	ID=cds3303	0	11	STM14_3422	srlA	glucitol%2Fsorbitol-specific enzyme IIC component	1,563101821	-2,297202414
NC_01685(	RefSeq	CDS	2999103	3000074	+	0	ID=cds3304	0	6,5	STM14_3423	srlE	PTS system glucitol%2Fsorbitol-specific transporter subun	0,852600993	-1,98056697
NC_01685(	RefSeq	CDS	3000086	3000448	+	0	ID=cds3305	0	5	STM14_3424	slrB	glucitol%2Fsorbitol-specific PTS system component IIA	0,710500828	-1,864806942
NC_01685(	RefSeq	CDS	3000460	3001239	+	0	ID=cds3306	0	5,5	STM14_3425	srlD	sorbitol-6-phosphate dehydrogenase	0,852600993	-1,98056697
NC_01685(	RefSeq	CDS	3001315	3001674	+	0	ID=cds3307	0	0	STM14_3426	gutM	DNA-binding transcriptional activator GutM	0	NA
NC_01685(	RefSeq	CDS	3001727	3001867	-	0	ID=cds3308	0	0	STM14_3427	STM14_3427	hypothetical protein	0	NA
NC_01685(	RefSeq	CDS	3001872	3002645	+	0	ID=cds3309	0	10,75	STM14_3428	srlR	DNA-binding transcriptional repressor SrlR	1,563101821	-2,297202414
NC_01685(	RefSeq	CDS	3002638	3003603	+	0	ID=cds3310	0	12,25	STM14_3429	gutQ	D-arabinose 5-phosphate isomerase	1,705201986	-2,329925387
NC_01685(	RefSeq	CDS	3003600	3005120	-	0	ID=cds3311	0	1,5	STM14_3430	ygaA	anaerobic nitric oxide reductase transcriptional regulator	0,284200331	-1,182723423
NC_01685(	RefSeq	CDS	3005306	3006745	+	0	ID=cds3312	0	0	STM14_3431	STM14_3431	anaerobic nitric oxide reductase flavorubredoxin	0	NA
NC_01685(	RefSeq	CDS	3006742	3007875	+	0	ID=cds3313	0	0	STM14_3432	ygbD	nitric oxide reductase	0	NA
NC_01685(	RefSeq	CDS	3007751	3007963	-	0	ID=cds3314	0	0,5	STM14_3433	STM14_3433	hydrogenase maturation protein	0	NA
NC_01685(	RefSeq	CDS	3007972	3010212	-	0	ID=cds3315	0,5	8	STM14_3434	hypF	hydrogenase maturation protein	1,136801324	-2,147403465
NC_01685(	RefSeq	CDS	3010214	3010348	-	0	ID=cds3316	0,5	0	STM14_3435	STM14_3435	hypothetical protein	0	NA
NC_01685(	RefSeq	CDS	3010358	3010903	-	0	ID=cds3317	0	0	STM14_3436	hydN	electron transport protein HydN	0	NA
NC_01685(	RefSeq	CDS	3010872	3011006	-	0	ID=cds3318	0	0	STM14_3437	STM14_3437	hypothetical protein	0	NA
NC_01685(	RefSeq	CDS	3011104	3011913	-	0	ID=cds3319	0	4	STM14_3438	STM14_3438	hypothetical protein	0,568400662	-1,710438693
NC_01685(	RefSeq	CDS	3011940	3012410	-	0	ID=cds3320	0	0,5	STM14_3439	hycI	hydrogenase 3 maturation protease	0	NA
NC_01685(	RefSeq	CDS	3012403	3012813	-	0	ID=cds3321	0	0	STM14_3440	hycH	hydrogenase 3 large subunit processing protein	0	NA
NC_01685(	RefSeq	CDS	3012810	3013577	-	0	ID=cds3322	0	0	STM14_3441	hycG	hydrogenase	0	NA
NC_01685(	RefSeq	CDS	3013577	3014119	-	0	ID=cds3323	0	0	STM14_3442	hycF	formate hydrogenlyase complex iron-sulfur subunit	0	NA
NC_01685(	RefSeq	CDS	3014129	3015838	-	0	ID=cds3324	0	1	STM14_3443	hycE	hydrogenase 3 large subunit	0,142100166	-0,623264422
NC_01685(	RefSeq	CDS	3015856	3016779	-	0	ID=cds3325	0	0	STM14_3444	hycD	hydrogenase 3 membrane subunit	0	NA
NC_01685(	RefSeq	CDS	3016782	3018608	-	0	ID=cds3326	0	1	STM14_3445	hycC	formate hydrogenlyase subunit 3	0,142100166	-0,623264422
NC_01685(	RefSeq	CDS	3018608	3019216	-	0	ID=cds3327	0	0,5	STM14_3446	hycB	hydrogenase-3 iron-sulfur subunit	0	NA
NC_01685(	RefSeq	CDS	3019238	3019342	-	0	ID=cds3328	0	0	STM14_3447	STM14_3447	hypothetical protein	0	NA
NC_01685(	RefSeq	CDS	3019362	3019823	-	0	ID=cds3329	0	0	STM14_3448	hycA	formate hydrogenlyase regulatory protein HycA	0	NA
NC_01685(	RefSeq	CDS	3019869	3020072	+	0	ID=cds3330	0	0	STM14_3449	STM14_3449	hypothetical protein	0	NA
NC_01685(	RefSeq	CDS	3020033	3020389	+	0	ID=cds3331	0	39,5	STM14_3450	hypA_1	hydrogenase nickel incorporation protein	5,684006621	-2,24950002
NC_01685(	RefSeq	CDS	3020458	3021330	+	0	ID=cds3332	0	85,5	STM14_3451	hypB	hydrogenase nickel incorporation protein HypB	12,22061424	-1,963849549
NC_01685(	RefSeq	CDS	3021321	3021593	+	0	ID=cds3333	0	29,5	STM14_3452	hypC	hydrogenase isoenzymes formation protein	4,263004966	-2,464060063
NC_01685(	RefSeq	CDS	3021593	3022714	+	0	ID=cds3334	0	89,75	STM14_3453	hypD	putative hydrogenase formation protein	12,7890149	-1,945533971
NC_01685(	RefSeq	CDS	3022711	3023721	+	0	ID=cds3335	0	53,25	STM14_3454	hypE	putative hydrogenase formation protein	7,531308773	-2,151665531
NC_01685(	RefSeq	CDS	3023955	3026018	+	0	ID=cds3336	0	60,5	STM14_3455	fhlA	formate hydrogen-lyase transcriptional activator	8,526009932	-2,104284262
NC_01685(	RefSeq	CDS	3026080	3026424	-	0	ID=cds3337	0	0	STM14_3456	ygbA	hypothetical protein	0	NA
NC_01685(	RefSeq	CDS	3026426	3026551	+	0	ID=cds3338	0	0,25	STM14_3457	STM14_3457	hypothetical protein	0	NA
NC_01685(	RefSeq	CDS	3026606	3027523	+	0	ID=cds3339	0	0	STM14_3458	sitA	putative periplasmic binding protein	0	NA
NC_01685(	RefSeq	CDS	3027520	3028341	+	0	ID=cds3340	1	3	STM14_3459	sitB	putative ATP-binding protein	2,185622867	1,419750352
NC_01685(	RefSeq	CDS	3028338	3029198	+	0	ID=cds3341	0	0,5	STM14_3460	sitC	putative permease	0	NA
NC_01685(	RefSeq	CDS	3029189	3030037	+	0	ID=cds3342	1	2,5	STM14_3461	sitD	putative permease	2,043522701	1,824239109
NC_01685(	RefSeq	CDS	3031204	3031959	-	0	ID=cds3343	0	66,5	STM14_3463	sprB	transcriptional regulator	9,378610925	-2,069476383
NC_01685(	RefSeq	CDS	3032212	3032319	+	0	ID=cds3344	0	0	STM14_3464	STM14_3464	hypothetical protein	0	NA
NC_01685(	RefSeq	CDS	3032344	3033231	-	0	ID=cds3345	0	41,75	STM14_3465	hilC	invasion regulatory protein	5,968206953	-2,232890319
NC_01685(	RefSeq	CDS	3033296	3033403	-	0	ID=cds3346	0	10	STM14_3466	STM14_3466	hypothetical protein	1,421001655	-2,256999172
NC_01685(	RefSeq	CDS	3033576	3034028	-	0	ID=cds3347	0	43	STM14_3467	orgC	putative cytoplasmic protein	6,110307118	-2,224756213
NC_01685(	RefSeq	CDS	3034025	3034705	-	0	ID=cds3348	6	69,75	STM14_3468	orgB	needle complex export protein	20,50294581	0,060067284
NC_01685(	RefSeq	CDS	3034662	3035261	-	0	ID=cds3349	0	99,75	STM14_3469	orgA	needle complex assembly protein	14,21001655	-1,905313172
NC_01685(	RefSeq	CDS	3035233	3035991	-	0	ID=cds3350	0	280,75	STM14_3470	prgK	needle complex inner membrane lipoprotein	39,93014652	-1,923340214
NC_01685(	RefSeq	CDS	3035988	3036293	-	0	ID=cds3351	0	160,25	STM14_3471	prgJ	needle complex minor subunit	22,73602649	-1,882019434
NC_01685(	RefSeq	CDS	3036312	3036554	-	0	ID=cds3352	0	97,5	STM14_3472	prgI	needle complex major subunit	13,92581622	-1,91259592
NC_01685(	RefSeq	CDS	3036579	3037757	-	0	ID=cds3353	0	168,5	STM14_3473	prgH	needle complex inner membrane protein	23,87282781	-1,886360475
NC_01685(	RefSeq	CDS	3038073	3039002	+	0	ID=cds3354	0	75	STM14_3474	hilD	invasion protein regulatory protein	10,65751242	-2,019002164
NC_01685(	RefSeq	CDS	3040093	3041754	+	0	ID=cds3355	0	50,25	STM14_3475	hilA	invasion protein regulator	7,105008277	-2,173389122
NC_01685(	RefSeq	CDS	3041772	3042254	+	0	ID=cds3356	0	12,58333333	STM14_3476	iagB	invasion protein precursor	1,847302152	-2,356210635
NC_01685(	RefSeq	CDS	3042308	3043939	-	0	ID=cds3357	0	110,25	STM14_3477	sptP	protein tyrosine phosphatase%2FGTPase activating protei	15,63101821	-1,86872757
NC_01685(	RefSeq	CDS	3043926	3044318	-	0	ID=cds3358	0	19,25	STM14_3478	sicP	secretion chaperone	2,699903145	-2,42419778
NC_01685(	RefSeq	CDS	3044345	3044605	-	0	ID=cds3359	0	15	STM14_3479	STM14_3479	putative cytoplasmic protein	2,131502483	-2,392032191
NC_01685(	RefSeq	CDS	3044649	3044897	-	0	ID=cds3360	0	24	STM14_3480	iacP	acyl carrier protein	3,410403973	-2,434086198
NC_01685(	RefSeq	CDS	3044916	3046973	-	0	ID=cds3361	0	137,3333333	STM14_3481	sipA	secreted effector protein	19,46772268	-1,867120417

NC_01685(	RefSeq	CDS	3046992	3048023	-	0	ID=cds3362	0	157,5	STM14_3482	sipD	translocation machinery component	22,45182615	-1,880874272
NC_01685(	RefSeq	CDS	3048094	3049323	-	0	ID=cds3363	0	335,8333333	STM14_3483	sipC	translocation machinery component	47,74565562	-1,932953508
NC_01685(	RefSeq	CDS	3049351	3051132	-	0	ID=cds3364	0	274,5	STM14_3484	sipB	translocation machinery component	38,93544536	-1,921863151
NC_01685(	RefSeq	CDS	3051135	3051632	-	0	ID=cds3365	0	89	STM14_3485	sicA	secretion chaperone	12,64691473	-1,950033207
NC_01685(	RefSeq	CDS	3051770	3052840	-	0	ID=cds3366	0	24	STM14_3486	spaS	surface presentation of antigens protein SpaS	3,410403973	-2,434086198
NC_01685(	RefSeq	CDS	3052827	3053618	-	0	ID=cds3367	0	13,5	STM14_3487	spaR	needle complex export protein	1,989402318	-2,376535557
NC_01685(	RefSeq	CDS	3053622	3053882	-	0	ID=cds3368	0	22	STM14_3488	spaQ	needle complex export protein	3,126203642	-2,431590384
NC_01685(	RefSeq	CDS	3053908	3054582	-	0	ID=cds3369	0	29,5	STM14_3489	spaP	surface presentation of antigens protein SpaP	4,263004966	-2,464060063
NC_01685(	RefSeq	CDS	3054572	3055483	-	0	ID=cds3370	0	100,25	STM14_3490	spaO	surface presentation of antigens protein SpaO	14,21001655	-1,905313172
NC_01685(	RefSeq	CDS	3055483	3056493	-	0	ID=cds3371	0	106,25	STM14_3491	invJ	needle length control protein	15,06261755	-1,883514636
NC_01685(	RefSeq	CDS	3056493	3056936	-	0	ID=cds3372	0	40,75	STM14_3492	invI	needle complex assembly protein	5,826106787	-2,241137745
NC_01685(	RefSeq	CDS	3056914	3058209	-	0	ID=cds3373	0	118,25	STM14_3493	invC	ATP synthase SpaL	16,76781953	-1,851132267
NC_01685(	RefSeq	CDS	3058206	3058613	-	0	ID=cds3374	0	53	STM14_3494	invB	secretion chaperone	7,531308773	-2,151665531
NC_01685(	RefSeq	CDS	3058637	3060694	-	0	ID=cds3375	0	128,5	STM14_3495	invA	needle complex export protein	18,18882119	-1,860051483
NC_01685(	RefSeq	CDS	3060719	3061837	-	0	ID=cds3376	0	58,5	STM14_3496	invE	invasion protein	8,241809601	-2,117369507
NC_01685(	RefSeq	CDS	3061834	3063522	-	0	ID=cds3377	0	156,5	STM14_3497	invG	outer membrane secretin precursor	22,16762582	-1,879703531
NC_01685(	RefSeq	CDS	3063519	3064268	-	0	ID=cds3378	1	93	STM14_3498	invF	invasion regulatory protein	14,97463776	-1,888790714
NC_01685(	RefSeq	CDS	3064626	3065069	+	0	ID=cds3379	0	25,5	STM14_3499	invH	needle complex outer membrane lipoprotein precursor	3,694604304	-2,438835558
NC_01685(	RefSeq	CDS	3065500	3065949	+	0	ID=cds3380	0	14,33333333	STM14_3500	STM14_3500	putative cytoplasmic protein	1,989402318	-2,376535557
NC_01685(	RefSeq	CDS	3065934	3066281	+	0	ID=cds3381	0	5,5	STM14_3501	STM14_3501	putative cytoplasmic protein	0,852600993	-1,98056697
NC_01685(	RefSeq	CDS	3066393	3066497	-	0	ID=cds3382	0	0	STM14_3502	STM14_3502	hypothetical protein	0	NA
NC_01685(	RefSeq	CDS	3066554	3066880	-	0	ID=cds3383	0	2	STM14_3503	STM14_3503	putative cytoplasmic protein	0,284200331	-1,182723423
NC_01685(	RefSeq	CDS	3067340	3067483	-	0	ID=cds3384	0	0	STM14_3504	STM14_3504	hypothetical protein	0	NA
NC_01685(	RefSeq	CDS	3067569	3067859	+	0	ID=cds3385	0	2,5	STM14_3505	STM14_3505	putative ABC-type transporter	0,284200331	-1,182723423
NC_01685(	RefSeq	CDS	3067856	3068383	+	0	ID=cds3386	0	3	STM14_3506	STM14_3506	putative acetyltransferase	0,426300497	-1,498211557
NC_01685(	RefSeq	CDS	3068456	3068632	-	0	ID=cds3387	0	0	STM14_3507	STM14_3507	hypothetical protein	0	NA
NC_01685(	RefSeq	CDS	3068687	3068806	+	0	ID=cds3388	0	0	STM14_3508	STM14_3508	hypothetical protein	0	NA
NC_01685(	RefSeq	CDS	3069008	3069664	+	0	ID=cds3389	0	0,5	STM14_3509	pphB	serine%2Fthreonine-specific protein phosphatase 2	0	NA
NC_01685(	RefSeq	CDS	3069836	3070357	-	0	ID=cds3390	0	0,5	STM14_3510	STM14_3510	putative cytoplasmic protein	0	NA
NC_01685(	RefSeq	CDS	3070516	3073083	+	0	ID=cds3391	0	20,5	STM14_3511	mutS	DNA mismatch repair protein	2,842003311	-2,427810709
NC_01685(	RefSeq	CDS	3073139	3073519	-	0	ID=cds3392	0	0	STM14_3512	STM14_3512	putative cytoplasmic protein	0	NA
NC_01685(	RefSeq	CDS	3073516	3074724	-	0	ID=cds3393	0	1	STM14_3513	STM14_3513	putative permease	0,142100166	-0,623264422
NC_01685(	RefSeq	CDS	3074812	3075744	+	0	ID=cds3394	0	0	STM14_3514	STM14_3514	putative transcriptional regulator	0	NA
NC_01685(	RefSeq	CDS	3075786	3077282	-	0	ID=cds3395	0	1,5	STM14_3515	STM14_3515	putative permease	0,284200331	-1,182723423
NC_01685(	RefSeq	CDS	3077279	3078229	-	0	ID=cds3396	0	0	STM14_3516	STM14_3516	putative nucleoside-diphosphate-sugar epimerase	0	NA
NC_01685(	RefSeq	CDS	3078269	3079045	-	0	ID=cds3397	0	0	STM14_3517	ygbM	hypothetical protein	0	NA
NC_01685(	RefSeq	CDS	3079050	3079688	-	0	ID=cds3398	0	0,5	STM14_3518	ygbL	putative aldolase	0	NA
NC_01685(	RefSeq	CDS	3079685	3080947	-	0	ID=cds3399	0	0	STM14_3519	ygbK	putative tRNA synthase	0	NA
NC_01685(	RefSeq	CDS	3080941	3081864	-	0	ID=cds3400	0	2	STM14_3520	ygbJ	3-hydroxyisobutyrate dehydrogenase	0,284200331	-1,182723423
NC_01685(	RefSeq	CDS	3082061	3082825	+	0	ID=cds3401	0	4,5	STM14_3521	ygbI	putative regulatory protein	0,568400662	-1,710438693
NC_01685(	RefSeq	CDS	3082844	3083248	-	0	ID=cds3402	0	1	STM14_3522	STM14_3522	putative transcriptional regulator	0,142100166	-0,623264422
NC_01685(	RefSeq	CDS	3083419	3084012	+	0	ID=cds3403	0	1	STM14_3523	STM14_3523	putative flavoprotein	0,142100166	-0,623264422
NC_01685(	RefSeq	CDS	3084012	3085439	+	0	ID=cds3404	0	1,5	STM14_3524	STM14_3524	putative 3-polyprenyl-4-hydroxybenzoate decarboxylase	0,284200331	-1,182723423
NC_01685(	RefSeq	CDS	3085450	3085686	+	0	ID=cds3405	0	0,5	STM14_3525	STM14_3525	putative cytoplasmic protein	0	NA
NC_01685(	RefSeq	CDS	3085731	3086723	-	0	ID=cds3406	3	148	STM14_3526	rpoS	RNA polymerase sigma factor RpoS	26,30879161	-1,362657983
NC_01685(	RefSeq	CDS	3086786	3087919	-	0	ID=cds3407	3	97	STM14_3527	nlpD	lipoprotein NlpD	19,06168317	-0,954784456
NC_01685(	RefSeq	CDS	3087981	3088118	+	0	ID=cds3408	0	0	STM14_3528	STM14_3528	hypothetical protein	0	NA
NC_01685(	RefSeq	CDS	3088095	3088721	-	0	ID=cds3409	0	23,75	STM14_3529	pcm	protein-L-isoaspartate O-methyltransferase	3,410403973	-2,434086198
NC_01685(	RefSeq	CDS	3088715	3089476	-	0	ID=cds3410	0	25,5	STM14_3530	surE	stationary phase survival protein SurE	3,694604304	-2,438835558
NC_01685(	RefSeq	CDS	3089457	3090506	-	0	ID=cds3411	0	28,25	STM14_3531	truD	tRNA pseudouridine synthase D	3,978804635	-2,448045729
NC_01685(	RefSeq	CDS	3090503	3090982	-	0	ID=cds3412	0	12,5	STM14_3532	ispF	2-C-methyl-D-erythritol 2%2C4-cyclo diphosphate synthas	1,705201986	-2,329925387
NC_01685(	RefSeq	CDS	3090982	3091692	-	0	ID=cds3413	0	17,5	STM14_3533	ispD	2-C-methyl-D-erythritol 4-phosphate cytidylyltransferase	2,55780298	-2,419166771
NC_01685(	RefSeq	CDS	3091711	3092022	-	0	ID=cds3414	0	9,5	STM14_3534	ftsB	cell division protein FtsB	1,421001655	-2,256999172
NC_01685(	RefSeq	CDS	3092010	3092120	-	0	ID=cds3415	1	4,5	STM14_3535	STM14_3535	hypothetical protein	2,327723032	1,125268706
NC_01685(	RefSeq	CDS	3092213	3092569	-	0	ID=cds3416	0	2,5	STM14_3536	ygbE	hypothetical protein	0,284200331	-1,182723423
NC_01685(	RefSeq	CDS	3092587	3093192	-	0	ID=cds3417	0	4,5	STM14_3537	cysC	adenylylsulfate kinase	0,568400662	-1,710438693
NC_01685(	RefSeq	CDS	3093179	3094618	-	0	ID=cds3418	0	3	STM14_3538	cysN	sulfate adenylyltransferase subunit 1	0,426300497	-1,498211557
NC_01685(	RefSeq	CDS	3094628	3095536	-	0	ID=cds3419	0	0,5	STM14_3539	cysD	sulfate adenylyltransferase subunit 2	0	NA
NC_01685(	RefSeq	CDS	3095787	3096833	+	0	ID=cds3420	0	15	STM14_3540	iap	alkaline phosphatase isozyme conversion aminopeptidase	2,131502483	-2,392032191
NC_01685(	RefSeq	CDS	3098420	3098716	-	0	ID=cds3421	0	4	STM14_3541	ygbF	hypothetical protein	0,568400662	-1,710438693
NC_01685(	RefSeq	CDS	3098713	3099633	-	0	ID=cds3422	0	7,5	STM14_3542	STM14_3542	putative cytoplasmic protein	1,136801324	-2,147403465
NC_01685(	RefSeq	CDS	3099630	3100280	-	0	ID=cds3423	0	2,5	STM14_3543	ygcH	putative cytoplasmic protein	0,284200331	-1,182723423
NC_01685(	RefSeq	CDS	3100262	3101008	-	0	ID=cds3424	0	2,5	STM14_3544	STM14_3544	putative cytoplasmic protein	0,284200331	-1,182723423
NC_01685(	RefSeq	CDS	3101019	3102077	-	0	ID=cds3425	0	11	STM14_3545	yghJ	putative cytoplasmic protein	1,563101821	-2,297202414
NC_01685(	RefSeq	CDS	3102091	3102651	-	0	ID=cds3426	0	8	STM14_3546	STM14_3546	putative transposase	1,136801324	-2,147403465
NC_01685(	RefSeq	CDS	3102648	3104204	-	0	ID=cds3427	0	5,5	STM14_3547	STM14_3547	putative cytoplasmic protein	0,852600993	-1,98056697
NC_01685(	RefSeq	CDS	3104216	3106879	-	0	ID=cds3428	0	10,5	STM14_3548	ygcB	putative helicase	1,421001655	-2,256999172
NC_01685(	RefSeq	CDS	3106978	3107112	-	0	ID=cds3429	0	0	STM14_3549	STM14_3549	hypothetical protein	0	NA
NC_01685(	RefSeq	CDS	3107324	3108277	+	0	ID=cds3430	0	8	STM14_3550	sopD	secreted effector protein	1,136801324	-2,147403465
NC_01685(	RefSeq	CDS	3108365	3109099	-	0	ID=cds3431	0	0	STM14_3551	cysH	phosphoadenosine phosphosulfate reductase	0	NA
NC_01685(	RefSeq	CDS	3109175	3110887	-	0	ID=cds3432	0	4,5	STM14_3552	cysI	sulfite reductase subunit beta	0,568400662	-1,710438693
NC_01685(	RefSeq	CDS	3110887	3112686	-	0	ID=cds3433	0	1	STM14_3553	cysJ	sulfite reductase subunit alpha	0,142100166	-0,623264422
NC_01685(	RefSeq	CDS	3113110	3113472	+	0	ID=cds3434	0	2	STM14_3554	ptpS	putative 6-pyruvoyl tetrahydrobiopterin synthase	0,284200331	-1,182723423
NC_01685(	RefSeq	CDS	3113560	3114357	-	0	ID=cds3435	0	13	STM14_3555	STM14_3555	putative metal-dependent hydrolase	1,847302152	-2,356210635
NC_01685(	RefSeq	CDS	3116367	3117038	-	0	ID=cds3436	5	9,5	STM14_3556	ygcF	hypothetical protein	10,21761351	1,731360567
NC_01685(	RefSeq	CDS	3117174	3118472	-	0	ID=cds3437	1	260,8333333	STM14_3557	eno	phosphopyruvate hydratase	38,84746557	-2,783892623
NC_01685(	RefSeq	CDS	3118555	3120192	-	0	ID=cds3438	6	152,5	STM14_3558	pyrG	CTP synthetase	32,15515938	-0,719222048
NC_01685(	RefSeq	CDS	3120420	3121220	-	0	ID=cds3439	1	8	STM14_3559	mazG	nucleoside triphosphate pyrophosphohydrolase	2,896123694	0,419298367
NC_01685(	RefSeq	CDS	3121321	3121458	+	0	ID=cds3440	0	0	STM14_3560	STM14_3560	hypothetical protein	0	NA
NC_01685(	RefSeq	CDS	3121826	3121978	+	0	ID=cds3441	0	0	STM14_3561	STM14_3561	putative fimbrial subunit	0	NA
NC_01685(	RefSeq	CDS	3121988	3122278	-	0	ID=cds3442	0	0	STM14_3562	STM14_3562	hypothetical protein	0	NA
NC_01685(	RefSeq	CDS	3122250	3122525	-	0	ID=cds3443	0	1	STM14_3563	STM14_3563	putative transcriptional regulator	0,142100166	-0,623264422
NC_01685(	RefSeq	CDS	3122690	3124924	-	0	ID=cds3444	0	90	STM14_3564	relA	GDP%2FGTP pyrophosphokinase	12,7890149	-1,945533971
NC_01685(	RefSeq	CDS	3124976	3126271	-	0	ID=cds3445	0	33,5	STM14_3565	rumA	23S rRNA 5-methyluridine methyltransferase	4,831405628	-2,523251848
NC_01685(	RefSeq	CDS	3126329	3129085	+	0	ID=cds3446	0	33,5	STM14_3566	barA	hybrid sensory histidine kinase BarA	4,831405628	-2,523251848
NC_01685(	RefSeq	CDS	3129129	3130271	-	0	ID=cds3447	0	7	STM14_3567	STM14_3567	putative glycerate kinase 2	0,994701159	-2,072978628
NC_01685(	RefSeq	CDS	3130349	3131689	-	0	ID=cds3448	0	8	STM14_3568	gudD	d-glucarate dehydratase	1,136801324	-2,147403465
NC_01685(	RefSeq	CDS	3131710	3133050	-	0	ID=cds3449	0	9	STM14_3569	ygcY	putative D-glucarate dehydratase	1,27890149	-2,207741391
NC_01685(	RefSeq	CDS	3133047	3134405	-	0	ID=cds3450	0	5,5	STM14_3570	gudT	putative D-glucarate permease	0,852600993	-1,98056697
NC_01685(	RefSeq	CDS	3134509	3134736	+	0	ID=cds3451	0	2,5	STM14_3571	STM14_3571	hypothetical protein	0,284200331	-1,182723423
NC_01685(	RefSeq	CDS	3134886	3135335	-	0	ID=cds3452	0	7	STM14_3572	STM14_3572	flavodoxin	0,994701159	-2,072978628
NC_01685(	RefSeq	CDS	3135354	3136136	-	0	ID=cds3453	0	2,5	STM14_3573	yqcB	tRNA pseudouridine synthase C	0,284200331	-1,182723423
NC_01685(	RefSeq	CDS	3136136	3136465	-	0	ID=cds3454	0	7,5	STM14_3574	yqcC	putative cytoplasmic protein	1,136801324	-2,147403465
NC_01685(	RefSeq	ncRNA	3136507	3136860	-	.	ID=rna62;P	3,5	688,4166667	STM14_3575	csrB	regulatory RNA	104,8022034	-2,589394305
NC_01685(	RefSeq	CDS	3137077	3137622	-	0	ID=cds3455	0	10	STM14_3576	syd	SecY interacting protein Syd	1,421001655	-2,256999172
NC_01685(	RefSeq	CDS	3137691	3138539	+	0	ID=cds3456	0	12	STM14_3577	queF	7-cyano-7-deazaguanine reductase	1,705201986	-2,329925387
NC_01685(	RefSeq	CDS	3138652	3140016	+	0	ID=cds3457	0	64,5	STM14_3578	ygdH	putative nucleotide binding protein	9,094410594	-2,079502768
NC_01685(	RefSeq	CDS	3140022	3140165	+	0	ID=cds3458	0	1	STM14_3579	STM14_3579	hypothetical protein	0,142100166	-0,623264422
NC_01685(	RefSeq	CDS	3140255	3140359	+	0	ID=cds3459	0	2	STM14_3580	STM14_3580	hypothetical protein	0,284200331	-1,182723423
NC_01685(	RefSeq	CDS	3140581	3141870	+	0	ID=cds3460	0	259,3333333	STM14_3581	sdaC	putative serine transport protein	36,80394287	-1,918452781
NC_01685(	RefSeq	CDS	3141929	3143296	+	0	ID=cds3461	2	209,5	STM14_3582	sdaB	L-serine dehydratase%2FL-threonine deaminase 2	33,3596795	-2,050919254
NC_01685(	RefSeq	CDS	3143293	3143478	-	0	ID=cds3462	0	0	STM14_3583	STM14_3583	hypothetical protein	0	NA
NC_01685(	RefSeq	CDS	3143407	3144222	+	0	ID=cds3463	0	8,75	STM14_3584	xni	exonuclease IX	1,27890149	-2,207741391
NC_01685(	RefSeq	CDS	3144205	3144342	-	0	ID=cds3464	0	0	STM14_3585	STM14_3585	L-1%2C2-propanediol oxidoreductase	0	NA
NC_01685(	RefSeq	CDS	3144314	3145462	-	0	ID=cds3465	0	1	STM14_3586	fucO	L-1%2C2-propanediol oxidoreductase	0,142100166	-0,623264422
NC_01685(	RefSeq	CDS	3145479	3146126	-	0	ID=cds3466	0	0	STM14_3587	fucA	L-fuculose phosphate aldolase	0	NA
NC_01685(	RefSeq	CDS	3146280	3146447	-	0	ID=cds3467	0	0	STM14_3588	STM14_3588	hypothetical protein	0	NA
NC_01685(	RefSeq	CDS	3148029	3149804	+	0	ID=cds3468	0	27	STM14_3590	fucI	L-fucose isomerase	3,836704469	-2,44262576
NC_01685(	RefSeq	CDS	3149905	3151323	+	0	ID=cds3469	0	13,5	STM14_3591	fucK	L-fuculokinase	1,989402318	-2,376535557
NC_01685(	RefSeq	CDS	3151325	3151747	+	0	ID=cds3470	0	6	STM14_3592	fucU	putative L-fucose-binding protein	0,852600993	-1,98056697
NC_01685(	RefSeq	CDS	3151805	3152515	+	0	ID=cds3471	0	9	STM14_3593	fucR	DNA-binding transcriptional activator FucR	1,27890149	-2,207741391
NC_01685(	RefSeq	CDS	3152574	3153674	-	0	ID=cds3472	1	14	STM14_3594	ygdE	putative RNA 2%27-O-ribose methyltransferase	3,748724687	-0,112256387
NC_01685(	RefSeq	CDS	3153667	3154062	-	0	ID=cds3473	0	7	STM14_3595	ygdD	hypothetical protein	0,994701159	-2,072978628
NC_01685(	RefSeq	CDS	3154081	3154998	-	0	ID=cds3474	3	13,5	STM14_3596	gcvA	DNA-binding transcriptional activator GcvA	7,267369427	0,916307618
NC_01685(	RefSeq	CDS	3155345	3155572	-	0	ID=cds3475	1	16	STM14_3597	ygdI	putative lipoprotein	4,032925019	-0,231745061
NC_01685(	RefSeq	CDS	3155765	3156970	+	0	ID=cds3476	0	13,5	STM14_3598	csdA	cysteine sulfinate desulfinase	1,989402318	-2,376535557
NC_01685(	RefSeq	CDS	3156970	3157413	+	0	ID=cds3477	0	6	STM14_3599	ygdK	putative FeS center assembly protein	0,852600993	-1,98056697
NC_01685(	RefSeq	CDS	3157563	3157685	+	0	ID=cds3478	0	11,5	STM14_3600	STM14_3600	hypothetical protein	1,705201986	-2,329925387
NC_01685(	RefSeq	CDS	3157686	3158600	+	0	ID=cds3479	1	15	STM14_3601	STM14_3601	putative integral membrane protein	3,890824853	-0,174265561
NC_01685(	RefSeq	CDS	3158652	3159458	-	0	ID=cds3480	0	3	STM14_3602	ygdL	hypothetical protein	0,426300497	-1,498211557
NC_01685(	RefSeq	CDS	3159568	3160665	-	0	ID=cds3481	2	24	STM14_3603	mltA	murein transglycosylase A	6,929048713	0,029280489
NC_01685(	RefSeq	CDS	3161165	3162418	-	0	ID=cds3482	0	17	STM14_3606	amiC	N-acetylmuramoyl-L-alanine amidase	2,415702814	-2,412509737
NC_01685(	RefSeq	CDS	3162651	3163982	+	0	ID=cds3483	1	3	STM14_3607	argA	N-acetylglutamate synthase	2,185622867	1,419750352
NC_01685(	RefSeq	CDS	3164085	3165920	-	0	ID=cds3484	0	17,5	STM14_3608	recD	exonuclease V subunit alpha	2,55780298	-2,419166771

NC_01685(	RefSeq	CDS	3165917	3169462	-	0	ID=cds3485	0	43	STM14_3609	recB	exonuclease V subunit beta	6,110307118	-2,224756213
NC_01685(	RefSeq	CDS	3169455	3172343	-	0	ID=cds3486	0	25	STM14_3610	ptr	protease III	3,552504138	-2,43600272
NC_01685(	RefSeq	CDS	3172521	3175892	-	0	ID=cds3487	0	28	STM14_3611	recC	exonuclease V subunit gamma	3,978804635	-2,448045729
NC_01685(	RefSeq	CDS	3175908	3176228	-	0	ID=cds3488	0	0,5	STM14_3612	ppdC	hypothetical protein	0	NA
NC_01685(	RefSeq	CDS	3176213	3176620	-	0	ID=cds3489	0	0	STM14_3613	ygdB	hypothetical protein	0	NA
NC_01685(	RefSeq	CDS	3176617	3177180	-	0	ID=cds3490	0	1	STM14_3614	ppdB	hypothetical protein	0,142100166	-0,623264422
NC_01685(	RefSeq	CDS	3177171	3177641	-	0	ID=cds3491	0	0	STM14_3615	ppdA	hypothetical protein	0	NA
NC_01685(	RefSeq	CDS	3177651	3177833	+	0	ID=cds3492	0	0	STM14_3616	STM14_3616	hypothetical protein	0	NA
NC_01685(	RefSeq	CDS	3177826	3178620	-	0	ID=cds3493	0	19	STM14_3617	thyA	thymidylate synthase	2,699903145	-2,42419778
NC_01685(	RefSeq	CDS	3178627	3179502	-	0	ID=cds3494	1	34	STM14_3618	lgt	prolipoprotein diacylglyceryl transferase	6,590727998	-0,915216377
NC_01685(	RefSeq	CDS	3179499	3179657	-	0	ID=cds3495	1	7	STM14_3619	STM14_3619	hypothetical protein	2,754023529	0,552839346
NC_01685(	RefSeq	CDS	3179718	3181964	-	0	ID=cds3496	3	77,5	STM14_3620	ptsP	fused phosphoenolpyruvate-protein phosphotransferase I	16,36178002	-0,73825176
NC_01685(	RefSeq	CDS	3181977	3182507	-	0	ID=cds3497	0	24,5	STM14_3621	ygdP	dinucleoside polyphosphate hydrolase	3,410403973	-2,434086198
NC_01685(	RefSeq	CDS	3182548	3182661	-	0	ID=cds3498	0	0	STM14_3622	STM14_3622	hypothetical protein	0	NA
NC_01685(	RefSeq	CDS	3182641	3182757	+	0	ID=cds3499	0	0	STM14_3623	STM14_3623	hypothetical protein	0	NA
NC_01685(	RefSeq	CDS	3182858	3183004	-	0	ID=cds3500	0	0	STM14_3624	STM14_3624	hypothetical protein	0	NA
NC_01685(	RefSeq	CDS	3183029	3183154	+	0	ID=cds3501	0	1,083333333	STM14_3625	STM14_3625	hypothetical protein	0,142100166	-0,623264422
NC_01685(	RefSeq	CDS	3183053	3183160	-	0	ID=cds3502	0	0	STM14_3626	STM14_3626	hypothetical protein	0	NA
NC_01685(	RefSeq	CDS	3183190	3183885	+	0	ID=cds3503	0	5,833333333	STM14_3627	mutH	DNA mismatch repair protein	0,852600993	-1,98056697
NC_01685(	RefSeq	CDS	3183985	3184095	+	0	ID=cds3504	0,5	1,5	STM14_3628	STM14_3628	hypothetical protein	0,284200331	-1,182723423
NC_01685(	RefSeq	CDS	3184067	3184780	+	0	ID=cds3505	0,5	10	STM14_3629	ygdQ	putative transport protein	1,421001655	-2,256999172
NC_01685(	RefSeq	CDS	3184950	3185168	+	0	ID=cds3506	0	3	STM14_3630	ygdR	putative peptide transport protein	0,426300497	-1,498211557
NC_01685(	RefSeq	CDS	3185172	3185342	+	0	ID=cds3507	0	1,5	STM14_3631	STM14_3631	hypothetical protein	0,284200331	-1,182723423
NC_01685(	RefSeq	CDS	3185359	3186399	+	0	ID=cds3508	0	6	STM14_3632	tas	putative aldo-keto reductase	0,852600993	-1,98056697
NC_01685(	RefSeq	CDS	3186486	3187688	-	0	ID=cds3509	0	10,5	STM14_3633	ygeD	hypothetical protein	1,421001655	-2,256999172
NC_01685(	RefSeq	CDS	3187681	3189840	-	0	ID=cds3510	0	16	STM14_3634	aas	bifunctional acyl-%5Bacyl carrier protein%5D synthetase%	2,273602649	-2,403729808
NC_01685(	RefSeq	CDS	3190309	3190434	-	0	ID=cds3511	0	0	STM14_3635	STM14_3635	hypothetical protein	0	NA
NC_01685(	RefSeq	CDS	3190433	3191461	+	0	ID=cds3512	0	9	STM14_3636	galR	DNA-binding transcriptional regulator GalR	1,27890149	-2,207741391
NC_01685(	RefSeq	CDS	3191472	3192494	+	0	ID=cds3513	0	10	STM14_3637	STM14_3637	putative transcriptional regulator	1,421001655	-2,256999172
NC_01685(	RefSeq	CDS	3192504	3193766	-	0	ID=cds3514	0	19	STM14_3638	lysA	diaminopimelate decarboxylase	2,699903145	-2,42419778
NC_01685(	RefSeq	CDS	3193884	3194819	+	0	ID=cds3515	0	1	STM14_3639	lysR	DNA-binding transcriptional regulator LysR	0,142100166	-0,623264422
NC_01685(	RefSeq	CDS	3194791	3195498	-	0	ID=cds3516	0	13	STM14_3640	ygeA	putative racemase	1,847302152	-2,356210635
NC_01685(	RefSeq	CDS	3195611	3197029	-	0	ID=cds3517	0	2	STM14_3641	araE	L-arabinose%2Fproton symport protein	0,284200331	-1,182723423
NC_01685(	RefSeq	CDS	3197131	3197247	-	0	ID=cds3518	0	0	STM14_3642	STM14_3642	hypothetical protein	0	NA
NC_01685(	RefSeq	CDS	3197383	3198144	-	0	ID=cds3519	0	4	STM14_3643	kduD	2-deoxy-D-gluconate 3-dehydrogenase	0,568400662	-1,710438693
NC_01685(	RefSeq	CDS	3198201	3199037	-	0	ID=cds3520	0	2	STM14_3644	kduI	5-keto-4-deoxyuronate isomerase	0,284200331	-1,182723423
NC_01685(	RefSeq	CDS	3199028	3199198	-	0	ID=cds3521	0	0	STM14_3645	STM14_3645	hypothetical protein	0	NA
NC_01685(	RefSeq	CDS	3199467	3200645	-	0	ID=cds3522	0	4,5	STM14_3646	yqeF	acetyl-CoA acetyltransferase	0,568400662	-1,710438693
NC_01685(	RefSeq	CDS	3200768	3201631	-	0	ID=cds3523	0	8	STM14_3647	STM14_3647	putative transcriptional regulator	1,136801324	-2,147403465
NC_01685(	RefSeq	CDS	3201708	3202190	+	0	ID=cds3524	0	1	STM14_3648	STM14_3648	putative inner membrane protein	0,142100166	-0,623264422
NC_01685(	RefSeq	CDS	3202193	3202315	+	0	ID=cds3525	0	0	STM14_3649	STM14_3649	hypothetical protein	0	NA
NC_01685(	RefSeq	CDS	3202332	3203561	+	0	ID=cds3526	0	9	STM14_3650	STM14_3650	putative transport protein	1,27890149	-2,207741391
NC_01685(	RefSeq	CDS	3203592	3203864	-	0	ID=cds3527	0	1	STM14_3651	yohL	putative cytoplasmic protein	0,142100166	-0,623264422
NC_01685(	RefSeq	CDS	3203986	3204894	+	0	ID=cds3528	0	1	STM14_3652	yohM	nickel%2Fcobalt efflux protein RcnA	0,142100166	-0,623264422
NC_01685(	RefSeq	CDS	3205104	3205862	-	0	ID=cds3529	0	0	STM14_3653	STM14_3653	putative cytoplasmic protein	0	NA
NC_01685(	RefSeq	CDS	3205849	3206361	-	0	ID=cds3530	0	0	STM14_3654	STM14_3654	hypothetical protein	0	NA
NC_01685(	RefSeq	CDS	3206505	3207581	-	0	ID=cds3531	0	0	STM14_3655	STM14_3655	putative outer membrane protein	0	NA
NC_01685(	RefSeq	CDS	3207578	3208321	-	0	ID=cds3532	0	0	STM14_3656	stdC	putative fimbrial chaperone	0	NA
NC_01685(	RefSeq	CDS	3208362	3210851	-	0	ID=cds3533	0	1	STM14_3657	stdB	putative outer membrane usher protein	0,142100166	-0,623264422
NC_01685(	RefSeq	CDS	3210549	3210968	+	0	ID=cds3534	0	0,5	STM14_3658	STM14_3658	hypothetical protein	0	NA
NC_01685(	RefSeq	CDS	3210971	3211555	-	0	ID=cds3535	0	0	STM14_3659	stdA	putative fimbrial-like protein	0	NA
NC_01685(	RefSeq	CDS	3212274	3212906	-	0	ID=cds3536	0	5	STM14_3660	STM14_3660	hypothetical protein	0,710500828	-1,864806942
NC_01685(	RefSeq	CDS	3212953	3213489	-	0	ID=cds3537	0	0	STM14_3661	STM14_3661	Ail%2FOmpX-like protein	0	NA
NC_01685(	RefSeq	CDS	3213534	3213719	-	0	ID=cds3538	0	0	STM14_3662	STM14_3662	hypothetical protein	0	NA
NC_01685(	RefSeq	CDS	3214295	3214693	-	0	ID=cds3539	1,5	12,75	STM14_3663	STM14_3663	plasmid maintenance protein	5,365946892	0,594220918
NC_01685(	RefSeq	CDS	3214693	3215070	-	0	ID=cds3540	1,5	5,25	STM14_3664	STM14_3664	putative virulence-associated protein	4,229145568	1,438201827
NC_01685(	RefSeq	CDS	3215088	3215348	+	0	ID=cds3541	0	0	STM14_3665	STM14_3665	endonuclease	0	NA
NC_01685(	RefSeq	CDS	3215425	3215670	-	0	ID=cds3542	0	1	STM14_3666	STM14_3666	hypothetical protein	0,142100166	-0,623264422
NC_01685(	RefSeq	CDS	3215623	3216429	+	0	ID=cds3543	0	7,5	STM14_3667	STM14_3667	putative inner membrane protein	1,136801324	-2,147403465
NC_01685(	RefSeq	CDS	3216714	3217472	-	0	ID=cds3544	0	14	STM14_3669	STM14_3669	putative metalloendopeptidase	1,989402318	-2,376535557
NC_01685(	RefSeq	CDS	3217519	3217686	+	0	ID=cds3545	0	0,5	STM14_3670	STM14_3670	hypothetical protein	0	NA
NC_01685(	RefSeq	CDS	3217737	3218282	+	0	ID=cds3546	0	12,5	STM14_3671	idi	isopentenyl-diphosphate delta-isomerase	1,705201986	-2,329925387
NC_01685(	RefSeq	CDS	3218358	3219875	-	0	ID=cds3547	0	132,5	STM14_3672	lysS	lysyl-tRNA synthetase	18,75722185	-1,863295025
NC_01685(	RefSeq	CDS	3219885	3220961	-	0	ID=cds3548	0	51,5	STM14_3673	prfB	peptide chain release factor 2	7,389208608	-2,158807911
NC_01685(	RefSeq	CDS	3221088	3222821	-	0	ID=cds3549	0	46,75	STM14_3674	recJ	ssDNA exonuclease RecJ	6,67870778	-2,193319559
NC_01685(	RefSeq	CDS	3222827	3223540	-	0	ID=cds3550	0	20,75	STM14_3675	dsbC	thiol:disulfide interchange protein DsbC	2,984103476	-2,430148754
NC_01685(	RefSeq	CDS	3223564	3224460	-	0	ID=cds3551	0	21	STM14_3676	xerD	site-specific tyrosine recombinase XerD	2,984103476	-2,430148754
NC_01685(	RefSeq	CDS	3224573	3225094	+	0	ID=cds3552	0	8,5	STM14_3677	fldB	flavodoxin FldB	1,136801324	-2,147403465
NC_01685(	RefSeq	CDS	3225147	3225560	-	0	ID=cds3553	0	43	STM14_3678	ygfX	putative inner membrane protein	6,110307118	-2,224756213
NC_01685(	RefSeq	CDS	3225541	3225807	-	0	ID=cds3554	0	15,5	STM14_3679	ygfY	hypothetical protein	2,273602649	-2,403729808
NC_01685(	RefSeq	CDS	3225806	3226042	+	0	ID=cds3555	0	1	STM14_3680	STM14_3680	hypothetical protein	0,142100166	-0,623264422
NC_01685(	RefSeq	CDS	3226057	3227037	+	0	ID=cds3556	0	11	STM14_3681	ygfZ	putative global regulator	1,563101821	-2,297202414
NC_01685(	RefSeq	CDS	3227153	3227812	-	0	ID=cds3557	5	12,5	STM14_3682	yqfA	putative hemolysin	10,50181384	1,570439755
NC_01685(	RefSeq	CDS	3227976	3228287	-	0	ID=cds3558	0	4	STM14_3683	yqfB	hypothetical protein	0,568400662	-1,710438693
NC_01685(	RefSeq	CDS	3228446	3229879	+	0	ID=cds3559	0	8	STM14_3684	bglA	6-phospho-beta-glucosidase	1,136801324	-2,147403465
NC_01685(	RefSeq	CDS	3229927	3230820	-	0	ID=cds3560	1	2	STM14_3685	STM14_3685	putative outer membrane protein	2,043522701	1,824239109
NC_01685(	RefSeq	CDS	3230904	3231050	-	0	ID=cds3561	1	0	STM14_3686	STM14_3686	immunoglobulin VH domain protein	1,75932237	1,868512432
NC_01685(	RefSeq	CDS	3231276	3234149	-	0	ID=cds3562	0	519,1666667	STM14_3687	gcvP	glycine dehydrogenase	73,74998591	-1,950964775
NC_01685(	RefSeq	CDS	3234312	3234701	-	0	ID=cds3563	0	143	STM14_3688	gcvH	glycine cleavage system protein H	20,32032367	-1,871405746
NC_01685(	RefSeq	CDS	3234727	3235821	-	0	ID=cds3564	0	195,25	STM14_3689	gcvT	glycine cleavage system aminomethyltransferase T	27,70953228	-1,898666075
NC_01685(	RefSeq	CDS	3235813	3235968	+	0	ID=cds3565	0	0,5	STM14_3690	STM14_3690	hypothetical protein	0	NA
NC_01685(	RefSeq	CDS	3236175	3236279	+	0	ID=cds3566	0	0	STM14_3691	STM14_3691	hypothetical protein	0	NA
NC_01685(	RefSeq	CDS	3236276	3237478	-	0	ID=cds3567	0	21,5	STM14_3692	visC	hypothetical protein	3,126203642	-2,431590384
NC_01685(	RefSeq	CDS	3237457	3237624	+	0	ID=cds3568	0	0	STM14_3693	STM14_3693	hypothetical protein	0	NA
NC_01685(	RefSeq	CDS	3237604	3238782	-	0	ID=cds3569	0	36,5	STM14_3694	ubiH	2-octaprenyl-6-methoxyphenyl hydroxylase	5,115605959	-2,281753063
NC_01685(	RefSeq	CDS	3238779	3240095	-	0	ID=cds3570	0	60,33333333	STM14_3695	pepP	proline aminopeptidase P II	8,526009932	-2,104284262
NC_01685(	RefSeq	CDS	3240121	3240699	-	0	ID=cds3571	0	11	STM14_3696	ygfB	hypothetical protein	1,563101821	-2,297202414
NC_01685(	RefSeq	CDS	3240866	3241195	+	0	ID=cds3572	0	44	STM14_3697	ygfE	Z-ring-associated protein	6,252407284	-2,216733793
NC_01685(	RefSeq	CDS	3241489	3242037	+	0	ID=cds3573	0	4,5	STM14_3698	ygfA	putative ligase	0,568400662	-1,710438693
NC_01685(	RefSeq	CDS	3242418	3243650	-	0	ID=cds3574	0	12,5	STM14_3699	serA	D-3-phosphoglycerate dehydrogenase	1,705201986	-2,329925387
NC_01685(	RefSeq	CDS	3243685	3243795	+	0	ID=cds3575	0	0	STM14_3700	STM14_3700	hypothetical protein	0	NA
NC_01685(	RefSeq	CDS	3243916	3244575	-	0	ID=cds3576	0	45	STM14_3701	rpiA	ribose-5-phosphate isomerase A	6,394507449	-2,208821363
NC_01685(	RefSeq	CDS	3244595	3244732	+	0	ID=cds3577	0	0	STM14_3702	STM14_3702	hypothetical protein	0	NA
NC_01685(	RefSeq	CDS	3244729	3245622	+	0	ID=cds3578	0	3,5	STM14_3703	iciA	chromosome replication initiation inhibitor protein	0,568400662	-1,710438693
NC_01685(	RefSeq	CDS	3245634	3245885	+	0	ID=cds3579	0	0,5	STM14_3704	STM14_3704	hypothetical protein	0	NA
NC_01685(	RefSeq	CDS	3245888	3246634	-	0	ID=cds3580	1	10	STM14_3705	yggE	hypothetical protein	3,180324025	0,201203536
NC_01685(	RefSeq	CDS	3246728	3247363	-	0	ID=cds3581	0	7,5	STM14_3706	yggA	arginine exporter protein	1,136801324	-2,147403465
NC_01685(	RefSeq	CDS	3247563	3248423	-	0	ID=cds3582	0	6	STM14_3707	yggB	mechanosensitive channel MscS	0,852600993	-1,98056697
NC_01685(	RefSeq	CDS	3248650	3249729	-	0	ID=cds3583	0	245,3333333	STM14_3708	fba	fructose-bisphosphate aldolase	34,81454056	-1,914927646
NC_01685(	RefSeq	CDS	3249831	3250994	-	0	ID=cds3584	1	220	STM14_3709	pgk	phosphoglycerate kinase	33,02135879	-2,634896482
NC_01685(	RefSeq	CDS	3251016	3252062	-	0	ID=cds3585	0	53,5	STM14_3710	epd	erythrose 4-phosphate dehydrogenase	7,673408939	-2,144619715
NC_01685(	RefSeq	CDS	3252233	3252433	+	0	ID=cds3586	0	1,25	STM14_3711	STM14_3711	hypothetical protein	0,142100166	-0,623264422
NC_01685(	RefSeq	CDS	3252436	3252861	+	0	ID=cds3587	0	1,75	STM14_3712	STM14_3712	putative DNA-binding protein	0,284200331	-1,182723423
NC_01685(	RefSeq	CDS	3252887	3253465	+	0	ID=cds3588	0	2,5	STM14_3713	STM14_3713	putative inner membrane protein	0,284200331	-1,182723423
NC_01685(	RefSeq	CDS	3253466	3254173	+	0	ID=cds3589	0	4,5	STM14_3714	STM14_3714	putative ABC-type cobalt transport system permease com	0,568400662	-1,710438693
NC_01685(	RefSeq	CDS	3254161	3254838	+	0	ID=cds3590	0	7	STM14_3715	STM14_3715	putative ABC-type cobalt transport system ATP-binding cc	0,994701159	-2,072978628
NC_01685(	RefSeq	CDS	3254832	3255488	+	0	ID=cds3591	0	2	STM14_3716	STM14_3716	putative ABC-type cobalt transport system ATP-binding cc	0,284200331	-1,182723423
NC_01685(	RefSeq	CDS	3255532	3257523	-	0	ID=cds3592	0	145,5	STM14_3717	tktA	transketolase	20,74662417	-1,873434052
NC_01685(	RefSeq	CDS	3257540	3257698	+	0	ID=cds3593	0	0	STM14_3718	STM14_3718	hypothetical protein	0	NA
NC_01685(	RefSeq	CDS	3257799	3258557	+	0	ID=cds3594	0	10	STM14_3719	yggG	putative Zn-dependent protease	1,421001655	-2,256999172
NC_01685(	RefSeq	CDS	3258658	3259578	-	0	ID=cds3595	0	11,5	STM14_3720	speB	agmatinase	1,705201986	-2,329925387
NC_01685(	RefSeq	CDS	3259783	3260598	-	0	ID=cds3596	0	1,5	STM14_3721	STM14_3721	putative hydrolase%2Facyltransferase	0,284200331	-1,182723423
NC_01685(	RefSeq	CDS	3260720	3260824	+	0	ID=cds3597	0	0,5	STM14_3722	STM14_3722	hypothetical protein	0	NA
NC_01685(	RefSeq	CDS	3260953	3261426	-	0	ID=cds3598	0	0	STM14_3723	STM14_3723	putative mannitol dehydrogenase	0	NA
NC_01685(	RefSeq	CDS	3261465	3262472	-	0	ID=cds3599	0	1	STM14_3724	STM14_3724	putative malate%2FL-lactate dehydrogenase	0,142100166	-0,623264422
NC_01685(	RefSeq	CDS	3262486	3263502	-	0	ID=cds3600	0	0	STM14_3725	STM14_3725	putative zinc-binding dehydrogenase	0	NA
NC_01685(	RefSeq	CDS	3263505	3264977	-	0	ID=cds3601	0	0	STM14_3726	STM14_3726	putative mannitol dehydrogenase	0	NA
NC_01685(	RefSeq	CDS	3266219	3266968	-	0	ID=cds3602	0	16,5	STM14_3727	STM14_3727	putative regulatory protein	2,273602649	-2,403729808
NC_01685(	RefSeq	CDS	3267173	3267904	-	0	ID=cds3603	0	1	STM14_3728	STM14_3728	putative outer membrane lipoprotein	0,142100166	-0,623264422
NC_01685(	RefSeq	CDS	3268048	3270024	-	0	ID=cds3604	0	31	STM14_3729	speA	arginine decarboxylase	4,405105132	-2,475774913
NC_01685(	RefSeq	CDS	3270033	3270164	-	0	ID=cds3605	0	1,5	STM14_3730	yqgB	putative inner membrane protein	0,284200331	-1,182723423
NC_01685(	RefSeq	CDS	3270459	3270758	-	0	ID=cds3606	2	5	STM14_3732	yqgD	putative inner membrane protein	4,229145568	1,438201827
NC_01685(	RefSeq	CDS	3270814	3271968	+	0	ID=cds3607	0	19	STM14_3733	metK	S-adenosylmethionine synthetase	2,699903145	-2,42419778
NC_01685(	RefSeq	CDS	3271996	3272121	-	0	ID=cds3608	0	0,5	STM14_3734	STM14_3734	hypothetical protein	0	NA

NC_01685(	RefSeq	CDS	3272461	3273855	+	0	ID=cds3609	0	12,5	STM14_3735	galP	galactose%2Fproton symporter	1,705201986	-2,329925387
NC_01685(	RefSeq	CDS	3273934	3274431	+	0	ID=cds3610	0	0	STM14_3736	sprT	hypothetical protein	0	NA
NC_01685(	RefSeq	CDS	3274526	3275233	+	0	ID=cds3611	2	2,5	STM14_3737	endA	DNA-specific endonuclease I	3,802845071	2,200856121
NC_01685(	RefSeq	CDS	3275310	3276041	+	0	ID=cds3612	0	8	STM14_3738	yggJ	hypothetical protein	1,136801324	-2,147403465
NC_01685(	RefSeq	CDS	3276061	3277008	+	0	ID=cds3613	0	24	STM14_3739	gshB	glutathione synthetase	3,410403973	-2,434086198
NC_01685(	RefSeq	CDS	3277224	3277787	+	0	ID=cds3614	0	19,5	STM14_3740	yqgE	hypothetical protein	2,842003311	-2,427810709
NC_01685(	RefSeq	CDS	3277787	3278203	+	0	ID=cds3615	0	20	STM14_3741	yqgF	Holliday junction resolvase-like protein	2,842003311	-2,427810709
NC_01685(	RefSeq	CDS	3278250	3278936	-	0	ID=cds3616	0	1,5	STM14_3742	STM14_3742	putative transcriptional regulator	0,284200331	-1,182723423
NC_01685(	RefSeq	CDS	3279068	3280048	-	0	ID=cds3617	0	0	STM14_3743	yggR	twitching motility protein	0	NA
NC_01685(	RefSeq	CDS	3280066	3280770	+	0	ID=cds3618	0	19,75	STM14_3744	yggS	hypothetical protein	2,842003311	-2,427810709
NC_01685(	RefSeq	CDS	3280789	3281355	+	0	ID=cds3619	0	19,25	STM14_3745	yggT	putative integral membrane protein	2,699903145	-2,42419778
NC_01685(	RefSeq	CDS	3281352	3281642	+	0	ID=cds3620	0	2	STM14_3746	yggU	hypothetical protein	0,284200331	-1,182723423
NC_01685(	RefSeq	CDS	3281650	3282243	+	0	ID=cds3621	0	8,5	STM14_3747	yggV	putative deoxyribonucleotide triphosphate pyrophosphat	1,136801324	-2,147403465
NC_01685(	RefSeq	CDS	3282236	3283372	+	0	ID=cds3622	0	22,5	STM14_3748	yggW	coproporphyrinogen III oxidase	3,126203642	-2,431590384
NC_01685(	RefSeq	CDS	3283463	3284470	-	0	ID=cds3623	0	27,5	STM14_3749	yggM	hypothetical protein	3,978804635	-2,448045729
NC_01685(	RefSeq	CDS	3284603	3285649	-	0	ID=cds3624	0	191	STM14_3750	ansB	L-asparaginase II	27,14113162	-1,897038569
NC_01685(	RefSeq	CDS	3285668	3285820	-	0	ID=cds3625	0	5,25	STM14_3751	STM14_3751	hypothetical protein	0,710500828	-1,864806942
NC_01685(	RefSeq	CDS	3285838	3286557	-	0	ID=cds3626	1	29,75	STM14_3752	yggN	hypothetical protein	6,022327336	-0,800851242
NC_01685(	RefSeq	CDS	3286607	3286933	-	0	ID=cds3627	0	26	STM14_3753	yggL	hypothetical protein	3,694604304	-2,438835558
NC_01685(	RefSeq	CDS	3286933	3287652	-	0	ID=cds3628	0	14	STM14_3754	trmB	tRNA %28guanine-N%287%29%29-methyltransferase	1,989402318	-2,376535557
NC_01685(	RefSeq	CDS	3287807	3288859	+	0	ID=cds3629	0	16	STM14_3755	mutY	adenine DNA glycosylase	2,273602649	-2,403729808
NC_01685(	RefSeq	CDS	3288887	3289162	+	0	ID=cds3630	0	8	STM14_3756	yggX	hypothetical protein	1,136801324	-2,147403465
NC_01685(	RefSeq	CDS	3289138	3289263	-	0	ID=cds3631	0	0	STM14_3757	STM14_3757	hypothetical protein	0	NA
NC_01685(	RefSeq	CDS	3289275	3290360	+	0	ID=cds3632	6	10,5	STM14_3758	mltC	murein transglycosylase C	11,97693588	1,923303842
NC_01685(	RefSeq	CDS	3290425	3290550	-	0	ID=cds3633	0	1	STM14_3759	STM14_3759	hypothetical protein	0,142100166	-0,623264422
NC_01685(	RefSeq	CDS	3291026	3293161	-	0	ID=cds3634	0	9	STM14_3761	speC	ornithine decarboxylase	1,27890149	-2,207741391
NC_01685(	RefSeq	CDS	3293235	3293459	-	0	ID=cds3635	0	2,5	STM14_3762	STM14_3762	hypothetical protein	0,284200331	-1,182723423
NC_01685(	RefSeq	CDS	3293413	3293547	+	0	ID=cds3636	0	0	STM14_3763	STM14_3763	hypothetical protein	0	NA
NC_01685(	RefSeq	CDS	3293586	3294293	+	0	ID=cds3637	0	0	STM14_3764	yqgA	putative inner membrane protein	0	NA
NC_01685(	RefSeq	CDS	3294633	3295067	-	0	ID=cds3638	0	0,5	STM14_3766	STM14_3766	putative lactoylglutathione lyase	0	NA
NC_01685(	RefSeq	CDS	3295078	3296403	-	0	ID=cds3639	0	2	STM14_3767	STM14_3767	putative acetyl-CoA hydrolase	0,284200331	-1,182723423
NC_01685(	RefSeq	CDS	3296428	3296955	-	0	ID=cds3640	0	3,5	STM14_3768	STM14_3768	putative monoamine oxidase	0,568400662	-1,710438693
NC_01685(	RefSeq	CDS	3296979	3297821	-	0	ID=cds3641	0	4	STM14_3769	STM14_3769	putative transcriptional regulator	0,568400662	-1,710438693
NC_01685(	RefSeq	CDS	3297913	3298791	+	0	ID=cds3642	0	5	STM14_3770	STM14_3770	putative transcriptional regulator	0,710500828	-1,864806942
NC_01685(	RefSeq	CDS	3298839	3300578	-	0	ID=cds3643	0	5	STM14_3771	STM14_3771	putative arylsulfatase	0,710500828	-1,864806942
NC_01685(	RefSeq	CDS	3300647	3301831	-	0	ID=cds3644	0	0	STM14_3772	STM14_3772	putative arylsulfatase regulator	0	NA
NC_01685(	RefSeq	CDS	3301831	3301965	-	0	ID=cds3645	0	0	STM14_3773	STM14_3773	hypothetical protein	0	NA
NC_01685(	RefSeq	CDS	3302110	3302214	+	0	ID=cds3646	0	0	STM14_3774	STM14_3774	hypothetical protein	0	NA
NC_01685(	RefSeq	CDS	3302343	3303026	+	0	ID=cds3647	0	0,5	STM14_3775	STM14_3775	putative response regulator	0	NA
NC_01685(	RefSeq	CDS	3303130	3303687	-	0	ID=cds3648	0	1	STM14_3776	STM14_3776	putative cytoplasmic protein	0,142100166	-0,623264422
NC_01685(	RefSeq	CDS	3303665	3305164	-	0	ID=cds3649	0	5	STM14_3777	STM14_3777	putative amino acid transporter	0,710500828	-1,864806942
NC_01685(	RefSeq	CDS	3305157	3305300	-	0	ID=cds3650	0	0	STM14_3778	STM14_3778	hypothetical protein	0	NA
NC_01685(	RefSeq	CDS	3305240	3305395	-	0	ID=cds3651	0	0	STM14_3779	STM14_3779	putative cytoplasmic protein	0	NA
NC_01685(	RefSeq	CDS	3305373	3305747	-	0	ID=cds3652	0	3	STM14_3780	STM14_3780	putative cytoplasmic protein	0,426300497	-1,498211557
NC_01685(	RefSeq	CDS	3305762	3307063	-	0	ID=cds3653	0	1,5	STM14_3781	STM14_3781	putative oxidoreductase	0,284200331	-1,182723423
NC_01685(	RefSeq	CDS	3307225	3308709	+	0	ID=cds3654	0	1	STM14_3782	STM14_3782	putative NAD-dependent aldehyde dehydrogenase	0,142100166	-0,623264422
NC_01685(	RefSeq	CDS	3308724	3308864	+	0	ID=cds3655	0	0	STM14_3783	STM14_3783	hypothetical protein	0	NA
NC_01685(	RefSeq	CDS	3308845	3309225	-	0	ID=cds3656	0	3,5	STM14_3784	STM14_3784	putative cytoplasmic protein	0,568400662	-1,710438693
NC_01685(	RefSeq	CDS	3309225	3309710	-	0	ID=cds3657	0	6,5	STM14_3785	STM14_3785	putative cytoplasmic protein	0,852600993	-1,98056697
NC_01685(	RefSeq	CDS	3310018	3310173	+	0	ID=cds3658	0	0	STM14_3786	STM14_3786	hypothetical protein	0	NA
NC_01685(	RefSeq	CDS	3310436	3311359	-	0	ID=cds3659	0	1	STM14_3787	STM14_3787	putative xylanase%2Fchitin deacetylase	0,142100166	-0,623264422
NC_01685(	RefSeq	CDS	3312290	3312427	+	0	ID=cds3660	0	0	STM14_3789	STM14_3789	hypothetical protein	0	NA
NC_01685(	RefSeq	CDS	3312496	3312606	-	0	ID=cds3661	0	0	STM14_3790	STM14_3790	hypothetical protein	0	NA
NC_01685(	RefSeq	CDS	3312670	3313974	-	0	ID=cds3662	0	25	STM14_3791	STM14_3791	putative permease	3,552504138	-2,43600272
NC_01685(	RefSeq	CDS	3314050	3314181	+	0	ID=cds3663	0	0	STM14_3792	STM14_3792	hypothetical protein	0	NA
NC_01685(	RefSeq	CDS	3314188	3314334	-	0	ID=cds3664	0	0	STM14_3793	STM14_3793	hypothetical protein	0	NA
NC_01685(	RefSeq	CDS	3314216	3314410	+	0	ID=cds3665	0	2,166666667	STM14_3794	STM14_3794	hypothetical protein	0,284200331	-1,182723423
NC_01685(	RefSeq	CDS	3314379	3315563	+	0	ID=cds3666	0	26,16666667	STM14_3795	STM14_3795	mannonate dehydratase	3,694604304	-2,438835558
NC_01685(	RefSeq	CDS	3315674	3317146	+	0	ID=cds3667	0	20,5	STM14_3796	STM14_3796	putative D-mannonate oxidoreductase	2,842003311	-2,427810709
NC_01685(	RefSeq	CDS	3317158	3318570	+	0	ID=cds3668	0	39	STM14_3797	STM14_3797	glucuronate isomerase	5,541906456	-2,25579729
NC_01685(	RefSeq	CDS	3318584	3318703	+	0	ID=cds3669	0	1	STM14_3798	STM14_3798	hypothetical protein	0,142100166	-0,623264422
NC_01685(	RefSeq	CDS	3318870	3319928	-	0	ID=cds3670	0	34	STM14_3799	STM14_3799	putative methyl-accepting chemotaxis protein	4,831405628	-2,523251848
NC_01685(	RefSeq	CDS	3319990	3320106	-	0	ID=cds3671	0	0	STM14_3800	STM14_3800	hypothetical protein	0	NA
NC_01685(	RefSeq	CDS	3320290	3320487	+	0	ID=cds3672	0	0	STM14_3801	STM14_3801	hypothetical protein	0	NA
NC_01685(	RefSeq	CDS	3320598	3322454	-	0	ID=cds3673	0	25	STM14_3802	gsp	bifunctional glutathionylspermidine amidase%2Fglutathio	3,552504138	-2,43600272
NC_01685(	RefSeq	CDS	3322464	3322571	-	0	ID=cds3674	0	0	STM14_3803	STM14_3803	hypothetical protein	0	NA
NC_01685(	RefSeq	CDS	3322681	3323547	+	0	ID=cds3675	0	8	STM14_3804	yghU	putative glutathione S-transferase YghU	1,136801324	-2,147403465
NC_01685(	RefSeq	CDS	3323615	3324325	-	0	ID=cds3676	0	2	STM14_3805	STM14_3805	hypothetical protein	0,284200331	-1,182723423
NC_01685(	RefSeq	CDS	3324325	3325377	-	0	ID=cds3677	0	3	STM14_3806	STM14_3806	putative periplasmic ferrichrome-binding protein	0,426300497	-1,498211557
NC_01685(	RefSeq	CDS	3325453	3325701	-	0	ID=cds3678	0	25	STM14_3807	hybG	hydrogenase 2 accessory protein HypG	3,552504138	-2,43600272
NC_01685(	RefSeq	CDS	3325729	3326070	-	0	ID=cds3679	0	26	STM14_3808	hypA_2	hydrogenase nickel incorporation protein HybF	3,694604304	-2,438835558
NC_01685(	RefSeq	CDS	3326063	3326551	-	0	ID=cds3680	0	70,75	STM14_3809	hybE	hydrogenase 2-specific chaperone	10,08911175	-2,040996541
NC_01685(	RefSeq	CDS	3326544	3327038	-	0	ID=cds3681	0	86,5	STM14_3810	hybD	hydrogenase 2 maturation endopeptidase	12,22061424	-1,963849549
NC_01685(	RefSeq	CDS	3327038	3328741	-	0	ID=cds3682	0	318,5833333	STM14_3811	hybC	hydrogenase 2 large subunit	45,32995281	-1,930310058
NC_01685(	RefSeq	CDS	3328738	3329916	-	0	ID=cds3683	0	283	STM14_3812	hybB	putative hydrogenase 2 b cytochrome subunit	40,21434685	-1,923749902
NC_01685(	RefSeq	CDS	3329906	3330892	-	0	ID=cds3684	1	226,5	STM14_3813	hybA	hydrogenase 2 protein HybA	33,87395978	-2,657777204
NC_01685(	RefSeq	CDS	3330895	3332013	-	0	ID=cds3685	0	239,3333333	STM14_3814	hypO	hydrogenase 2 small subunit	33,96193956	-1,913302279
NC_01685(	RefSeq	CDS	3332051	3332179	+	0	ID=cds3686	0	0	STM14_3815	STM14_3815	hypothetical protein	0	NA
NC_01685(	RefSeq	CDS	3332203	3332490	-	0	ID=cds3687	0	2	STM14_3816	yghW	putative cytoplasmic protein	0,284200331	-1,182723423
NC_01685(	RefSeq	CDS	3332575	3334218	-	0	ID=cds3688	1	7	STM14_3817	STM14_3817	putative methyl-accepting chemotaxis protein	2,754023529	0,552839346
NC_01685(	RefSeq	CDS	3334525	3335019	-	0	ID=cds3689	0	4	STM14_3818	yqhA	hypothetical protein	0,568400662	-1,710438693
NC_01685(	RefSeq	CDS	3335174	3335281	+	0	ID=cds3690	0	0,285714286	STM14_3819	STM14_3819	hypothetical protein	0	NA
NC_01685(	RefSeq	CDS	3335299	3335814	-	0	ID=cds3691	0	3,5	STM14_3820	STM14_3820	putative ATP-dependent RNA helicase-like protein	0,568400662	-1,710438693
NC_01685(	RefSeq	CDS	3335906	3336316	+	0	ID=cds3692	0	3	STM14_3821	STM14_3821	putative cytoplasmic protein	0,426300497	-1,498211557
NC_01685(	RefSeq	CDS	3336303	3336707	+	0	ID=cds3693	0	3	STM14_3822	STM14_3822	putative cytoplasmic protein	0,426300497	-1,498211557
NC_01685(	RefSeq	CDS	3336831	3337715	+	0	ID=cds3694	0	6,5	STM14_3823	yghA	oxidoreductase	0,852600993	-1,98056697
NC_01685(	RefSeq	CDS	3337828	3338253	-	0	ID=cds3695	3	6,5	STM14_3824	exbD	biopolymer transport protein ExbD	6,130568103	1,664704357
NC_01685(	RefSeq	CDS	3338260	3338994	-	0	ID=cds3696	4	16,5	STM14_3825	exbB	biopolymer transport protein ExbB	9,310892128	1,080610291
NC_01685(	RefSeq	CDS	3338987	3339136	-	0	ID=cds3697	0	5	STM14_3826	STM14_3826	putative inner membrane protein	0,710500828	-1,864806942
NC_01685(	RefSeq	CDS	3339111	3340433	+	0	ID=cds3698	0	4,5	STM14_3827	metC	cystathionine beta-lyase	0,568400662	-1,710438693
NC_01685(	RefSeq	CDS	3340573	3341232	+	0	ID=cds3699	0	34	STM14_3828	yghB	hypothetical protein	4,831405628	-2,523251848
NC_01685(	RefSeq	CDS	3341296	3342213	-	0	ID=cds3700	0	1	STM14_3829	yqhC	putative transcriptional regulator	0,142100166	-0,623264422
NC_01685(	RefSeq	CDS	3342234	3342434	+	0	ID=cds3701	0	1	STM14_3830	STM14_3830	hypothetical protein	0,142100166	-0,623264422
NC_01685(	RefSeq	CDS	3342388	3343551	+	0	ID=cds3702	0	8,5	STM14_3831	yqhD	putative alcohol dehydrogenase	1,136801324	-2,147403465
NC_01685(	RefSeq	CDS	3343657	3344484	+	0	ID=cds3703	0	10	STM14_3832	dkgA	2%2C5-diketo-D-gluconate reductase A	1,421001655	-2,256999172
NC_01685(	RefSeq	CDS	3344492	3344629	-	0	ID=cds3704	0	0	STM14_3833	STM14_3833	hypothetical protein	0	NA
NC_01685(	RefSeq	CDS	3344530	3344640	+	0	ID=cds3705	0	0	STM14_3834	STM14_3834	hypothetical protein	0	NA
NC_01685(	RefSeq	CDS	3344681	3346135	+	0	ID=cds3706	0	0	STM14_3835	STM14_3835	putative cation transporter	0	NA
NC_01685(	RefSeq	CDS	3346180	3346635	+	0	ID=cds3707	0	4,5	STM14_3836	STM14_3836	putative diadenosine tetraphosphatehydrolase	0,568400662	-1,710438693
NC_01685(	RefSeq	CDS	3346625	3346789	+	0	ID=cds3708	0	1,5	STM14_3837	STM14_3837	hypothetical protein	0,284200331	-1,182723423
NC_01685(	RefSeq	CDS	3346793	3346924	-	0	ID=cds3709	0	0	STM14_3838	STM14_3838	hypothetical protein	0	NA
NC_01685(	RefSeq	CDS	3347190	3349361	-	0	ID=cds3710	0	14,5	STM14_3839	ygiR	hypothetical protein	1,989402318	-2,376535557
NC_01685(	RefSeq	CDS	3349441	3349545	-	0	ID=cds3711	0	0	STM14_3840	STM14_3840	hypothetical protein	0	NA
NC_01685(	RefSeq	CDS	3349849	3350832	+	0	ID=cds3712	0	2	STM14_3841	STM14_3841	putative periplasmic dicarboxylate-binding protein	0,284200331	-1,182723423
NC_01685(	RefSeq	CDS	3350874	3351356	+	0	ID=cds3713	0	0	STM14_3842	STM14_3842	putative inner membrane protein	0	NA
NC_01685(	RefSeq	CDS	3351367	3352674	+	0	ID=cds3714	1	1	STM14_3843	ygiK	putative transporter	1,901422535	2,462014545
NC_01685(	RefSeq	CDS	3352719	3354131	-	0	ID=cds3715	1	17	STM14_3844	sufI	repressor protein for FtsI	4,175025184	-0,285403406
NC_01685(	RefSeq	CDS	3354205	3354942	-	0	ID=cds3716	0	22	STM14_3845	plsC	1-acyl-sn-glycerol-3-phosphate acyltransferase	3,126203642	-2,431590384
NC_01685(	RefSeq	CDS	3355199	3357457	-	0	ID=cds3717	0	32,5	STM14_3846	parC	DNA topoisomerase IV subunit A	4,547205297	-2,489878815
NC_01685(	RefSeq	CDS	3357568	3358434	-	0	ID=cds3718	0	16,5	STM14_3847	STM14_3847	putative regulatory protein	2,273602649	-2,403729808
NC_01685(	RefSeq	CDS	3358506	3358898	-	0	ID=cds3719	0	28,5	STM14_3848	ygiW	putative outer membrane protein	3,978804635	-2,448045729
NC_01685(	RefSeq	CDS	3359050	3359709	+	0	ID=cds3720	0	3	STM14_3849	ygiX	DNA-binding transcriptional regulator QseB	0,426300497	-1,498211557
NC_01685(	RefSeq	CDS	3359706	3361055	+	0	ID=cds3721	0	2,5	STM14_3850	ygiY	sensor protein QseC	0,284200331	-1,182723423
NC_01685(	RefSeq	CDS	3361049	3361165	+	0	ID=cds3722	0	1	STM14_3851	STM14_3851	hypothetical protein	0,142100166	-0,623264422
NC_01685(	RefSeq	CDS	3361162	3361743	+	0	ID=cds3723	0	2	STM14_3852	mdaB	NADPH-specific quinone oxidoreductase	0,284200331	-1,182723423
NC_01685(	RefSeq	CDS	3361775	3362089	+	0	ID=cds3724	0	6	STM14_3853	ygiN	putative cytoplasmic protein	0,852600993	-1,98056697
NC_01685(	RefSeq	CDS	3362214	3364106	-	0	ID=cds3725	3	110	STM14_3854	parE	DNA topoisomerase IV subunit B	20,90898532	-1,07778949
NC_01685(	RefSeq	CDS	3364134	3364715	-	0	ID=cds3726	0	44,5	STM14_3855	yqiA	esterase YqiA	6,252407284	-2,216733793
NC_01685(	RefSeq	CDS	3364715	3365542	-	0	ID=cds3727	0	64,5	STM14_3856	icc	cyclic 3%27%2C5%27-adenosine monophosphate phospho	9,094410594	-2,079502768
NC_01685(	RefSeq	CDS	3365567	3365989	-	0	ID=cds3728	0	8	STM14_3857	yqiB	hypothetical protein	1,136801324	-2,147403465
NC_01685(	RefSeq	CDS	3365986	3366618	-	0	ID=cds3729	0	4	STM14_3858	nudF	ADP-ribose pyrophosphatase NudF	0,568400662	-1,710438693
NC_01685(	RefSeq	CDS	3366825	3368294	+	0	ID=cds3730	0	53,25	STM14_3859	tolC	outer membrane channel protein	7,531308773	-2,151665531
NC_01685(	RefSeq	CDS	3368291	3368398	+	0	ID=cds3731	0	1,25	STM14_3860	STM14_3860	hypothetical protein	0,142100166	-0,623264422
NC_01685(	RefSeq	CDS	3368507	3369178	+	0	ID=cds3732	0	24	STM14_3861	ygiB	hypothetical protein	3,410403973	-2,434086198

NC_01685	RefSeq	CDS	3369184	3370347	+	0	ID=cds373	0	66	STM14_3862	ygiC	putative glutathionylspermidine synthase	9,378610925	-2,069476383
NC_01685	RefSeq	CDS	3370409	3371239	-	0	ID=cds373	0	12,5	STM14_3863	ygiD	hypothetical protein	1,705201986	-2,329925387
NC_01685	RefSeq	CDS	3371338	3372111	+	0	ID=cds373	0	4	STM14_3864	ygiE	zinc transporter ZupT	0,568400662	-1,710438693
NC_01685	RefSeq	CDS	3372178	3372354	-	0	ID=cds373	0	7,5	STM14_3865	STM14_3865	hypothetical protein	1,136801324	-2,147403465
NC_01685	RefSeq	CDS	3372754	3374550	+	0	ID=cds373	0	0,5	STM14_3866	STM14_3866	putative arylsulfate sulfotransferase	0	NA
NC_01685	RefSeq	CDS	3374570	3375241	+	0	ID=cds373	0	0	STM14_3867	STM14_3867	putative disulfide bond formation protein	0	NA
NC_01685	RefSeq	CDS	3375256	3375933	+	0	ID=cds373	0	2	STM14_3868	STM14_3868	putative disulfide oxidoreductase	0,284200331	-1,182723423
NC_01685	RefSeq	CDS	3376104	3376757	-	0	ID=cds374	0	43	STM14_3869	ribB	3%2C4-dihydroxy-2-butanone 4-phosphate synthase	6,110307118	-2,224756213
NC_01685	RefSeq	CDS	3377080	3377493	+	0	ID=cds374	0	3	STM14_3870	yqiC	putative cytoplasmic protein	0,426300497	-1,498211557
NC_01685	RefSeq	CDS	3377567	3377776	-	0	ID=cds374	0	12	STM14_3871	glgS	glycogen synthesis protein GlgS	1,705201986	-2,329925387
NC_01685	RefSeq	CDS	3377873	3378022	-	0	ID=cds374	0	0	STM14_3872	STM14_3872	hypothetical protein	0	NA
NC_01685	RefSeq	CDS	3378034	3378654	+	0	ID=cds374	0	0	STM14_3873	STM14_3873	putative inner membrane protein	0	NA
NC_01685	RefSeq	CDS	3378673	3380352	+	0	ID=cds374	0	6,5	STM14_3874	yqiK	hypothetical protein	0,852600993	-1,98056697
NC_01685	RefSeq	CDS	3380454	3380651	+	0	ID=cds374	8	0	STM14_3875	STM14_3875	hypothetical protein	14,07457896	1,970868688
NC_01685	RefSeq	CDS	3380812	3382245	-	0	ID=cds374	0	35,5	STM14_3876	rfaE	bifunctional heptose 7-phosphate kinase%2Fheptose 1-ph	5,115605959	-2,281753063
NC_01685	RefSeq	CDS	3382293	3385136	-	0	ID=cds374	0	53,33333333	STM14_3877	glnE	bifunctional glutamine-synthetase adenylyltransferase%2	7,531308773	-2,151665531
NC_01685	RefSeq	CDS	3385254	3386555	-	0	ID=cds374	0	17,5	STM14_3878	ygiF	putative cytoplasmic protein	2,55780298	-2,419166771
NC_01685	RefSeq	CDS	3386797	3387411	+	0	ID=cds375	0	129	STM14_3879	ygiM	putative signal transduction protein	18,33092135	-1,860878454
NC_01685	RefSeq	CDS	3387474	3388715	+	0	ID=cds375	0	49	STM14_3880	cca	multifunctional tRNA nucleotidyl transferase%2F2%273%2	6,962908111	-2,178239812
NC_01685	RefSeq	CDS	3388820	3389644	-	0	ID=cds375	0	10	STM14_3881	uppP	undecaprenyl pyrophosphate phosphatase	1,421001655	-2,256999172
NC_01685	RefSeq	CDS	3389739	3390101	-	0	ID=cds375	0	2	STM14_3882	folB	bifunctional dihydroneopterin aldolase%2Fdihydroneopte	0,284200331	-1,182723423
NC_01685	RefSeq	CDS	3390205	3390816	+	0	ID=cds375	0	4	STM14_3883	ygiH	putative glycerol-3-phosphate acyltransferase PlsY	0,568400662	-1,710438693
NC_01685	RefSeq	CDS	3390891	3391163	+	0	ID=cds375	0	7,5	STM14_3884	STM14_3884	hypothetical protein	1,136801324	-2,147403465
NC_01685	RefSeq	CDS	3391067	3392080	-	0	ID=cds375	0	9,5	STM14_3885	gcp	O-sialoglycoprotein endopeptidase	1,421001655	-2,256999172
NC_01685	RefSeq	CDS	3392308	3392523	+	0	ID=cds375	1	71	STM14_3886	rpsU	30S ribosomal protein S21	11,84843412	-1,622257172
NC_01685	RefSeq	CDS	3392759	3394504	+	0	ID=cds375	10	64	STM14_3887	dnaG	DNA primase	26,68763429	0,665990932
NC_01685	RefSeq	CDS	3394519	3396501	+	0	ID=cds375	1	143	STM14_3888	rpoD	RNA polymerase sigma factor RpoD	22,07964604	-2,276556465
NC_01685	RefSeq	CDS	3396625	3397131	-	0	ID=cds376	0	10,5	STM14_3889	mug	G%2FU mismatch-specific DNA glycosylase	1,421001655	-2,256999172
NC_01685	RefSeq	CDS	3397412	3398179	-	0	ID=cds376	0	5	STM14_3891	yqjH	putative transporter	0,710500828	-1,864806942
NC_01685	RefSeq	CDS	3398411	3399058	+	0	ID=cds376	0	6	STM14_3892	yqjI	putative transcriptional regulator	0,852600993	-1,98056697
NC_01685	RefSeq	CDS	3399055	3400620	-	0	ID=cds376	0	35,66666667	STM14_3893	STM14_3893	putative methyl-accepting chemotaxis protein	5,115605959	-2,281753063
NC_01685	RefSeq	CDS	3401008	3402528	-	0	ID=cds376	0	21,83333333	STM14_3894	aer	aerotaxis sensor receptor	3,126203642	-2,431590384
NC_01685	RefSeq	CDS	3402540	3402695	+	0	ID=cds376	0	0	STM14_3895	STM14_3895	hypothetical protein	0	NA
NC_01685	RefSeq	CDS	3402931	3404337	+	0	ID=cds376	4	1	STM14_3896	oat	putrescine--2-oxoglutarate aminotransferase	7,179389645	3,174770149
NC_01685	RefSeq	CDS	3404350	3404460	+	0	ID=cds376	0	0	STM14_3897	STM14_3897	hypothetical protein	0	NA
NC_01685	RefSeq	CDS	3404508	3406526	+	0	ID=cds376	0	2,5	STM14_3898	fadH	2%2C4-dienoyl-CoA reductase	0,284200331	-1,182723423
NC_01685	RefSeq	CDS	3406607	3407743	-	0	ID=cds376	3	12,5	STM14_3899	ygjO	putative methyltransferase	6,983169096	1,056079994
NC_01685	RefSeq	CDS	3407829	3408326	+	0	ID=cds377	0	2	STM14_3900	ygjP	putative metal-dependent hydrolase	0,284200331	-1,182723423
NC_01685	RefSeq	CDS	3408358	3408459	-	0	ID=cds377	0	0,5	STM14_3901	STM14_3901	hypothetical protein	0	NA
NC_01685	RefSeq	CDS	3408478	3409170	+	0	ID=cds377	0	6	STM14_3902	ygjQ	putative integral membrane protein	0,852600993	-1,98056697
NC_01685	RefSeq	CDS	3409259	3410257	+	0	ID=cds377	0	33,5	STM14_3903	ygjR	putative dehydrogenase	4,831405628	-2,523251848
NC_01685	RefSeq	CDS	3410298	3410435	+	0	ID=cds377	19	3	STM14_3904	STM14_3904	hypothetical protein	33,85342553	3,818292024
NC_01685	RefSeq	CDS	3410530	3411498	+	0	ID=cds377	0	0	STM14_3905	ygjT	putative tellurite resistance protein	0	NA
NC_01685	RefSeq	CDS	3411753	3412997	+	0	ID=cds377	0	5	STM14_3906	ygjU	serine%2Fthreonine transporter SstT	0,710500828	-1,864806942
NC_01685	RefSeq	CDS	3412994	3413194	-	0	ID=cds377	0	1	STM14_3907	STM14_3907	hypothetical protein	0,142100166	-0,623264422
NC_01685	RefSeq	CDS	3413447	3414109	+	0	ID=cds377	0	23,83333333	STM14_3908	yqjA	hypothetical protein	3,410403973	-2,434086198
NC_01685	RefSeq	CDS	3414113	3414496	+	0	ID=cds377	0	5,75	STM14_3909	yqjB	hypothetical protein	0,852600993	-1,98056697
NC_01685	RefSeq	CDS	3414641	3415009	+	0	ID=cds378	0	19	STM14_3910	yqjC	hypothetical protein	2,699903145	-2,42419778
NC_01685	RefSeq	CDS	3415051	3415356	+	0	ID=cds378	0	8	STM14_3911	yqjD	putative inner membrane protein	1,136801324	-2,147403465
NC_01685	RefSeq	CDS	3415359	3415757	+	0	ID=cds378	0	23,5	STM14_3912	yqjE	putative inner membrane protein	3,410403973	-2,434086198
NC_01685	RefSeq	CDS	3415754	3416053	+	0	ID=cds378	0	4,5	STM14_3913	yqjK	putative inner membrane protein	0,568400662	-1,710438693
NC_01685	RefSeq	CDS	3416198	3416683	+	0	ID=cds378	0	0	STM14_3914	yqjF	hypothetical protein	0	NA
NC_01685	RefSeq	CDS	3416751	3417737	+	0	ID=cds378	0	1	STM14_3915	yqjG	putative glutathione S-transferase	0,142100166	-0,623264422
NC_01685	RefSeq	CDS	3417861	3418226	+	0	ID=cds378	0	2	STM14_3916	yhaH	putative inner membrane protein	0,284200331	-1,182723423
NC_01685	RefSeq	CDS	3418265	3419161	-	0	ID=cds378	0	127,8333333	STM14_3917	yhaJ	putative transcriptional regulator	18,18882119	-1,860051483
NC_01685	RefSeq	CDS	3419266	3419967	+	0	ID=cds378	0	0	STM14_3918	yhaK	putative cytoplasmic protein	0	NA
NC_01685	RefSeq	CDS	3419991	3420155	+	0	ID=cds378	0	1	STM14_3919	yhaL	putative cytoplasmic protein	0,142100166	-0,623264422
NC_01685	RefSeq	CDS	3420268	3421578	-	0	ID=cds379	0	17,5	STM14_3920	yhaN	putative inner membrane protein	2,55780298	-2,419166771
NC_01685	RefSeq	CDS	3421604	3423043	-	0	ID=cds379	0	14	STM14_3921	yhaO	putative transport protein	1,989402318	-2,376535557
NC_01685	RefSeq	CDS	3423251	3424615	-	0	ID=cds379	0	160,8333333	STM14_3922	tdcG	L-serine deaminase	22,87812665	-1,882582685
NC_01685	RefSeq	CDS	3424685	3426979	-	0	ID=cds379	0	392,5833333	STM14_3923	tdcE	pyruvate formate-lyase 4%2F2-ketobutyrate formate-lyas	55,84536506	-1,940249986
NC_01685	RefSeq	CDS	3427013	3428221	-	0	ID=cds379	0,5	303,5833333	STM14_3924	tdcD	propionate%2Facetate kinase	43,19845032	-1,927751005
NC_01685	RefSeq	CDS	3428287	3429618	-	0	ID=cds379	0	472,5	STM14_3925	tdcC	threonine%2Fserine transporter TdcC	67,07127813	-1,94760169
NC_01685	RefSeq	CDS	3429639	3430628	-	0	ID=cds379	0	274,8333333	STM14_3926	tdcB	threonine dehydratase	39,07754552	-1,922078381
NC_01685	RefSeq	CDS	3430726	3431664	-	0	ID=cds379	0	141,1666667	STM14_3927	tdcA	DNA-binding transcriptional activator TdcA	20,03612334	-1,870012233
NC_01685	RefSeq	ncRNA	3433052	3433432	+	.	ID=rna68;P	0	0	STM14_3928	rnpB	regulatory RNA	0	NA
NC_01685	RefSeq	CDS	3433463	3434608	-	0	ID=cds379	0	15,33333333	STM14_3929	garK	glycerate kinase	2,131502483	-2,392032191
NC_01685	RefSeq	CDS	3434706	3435596	-	0	ID=cds379	0	11	STM14_3930	garR	tartronate semialdehyde reductase	1,563101821	-2,297202414
NC_01685	RefSeq	CDS	3435622	3436392	-	0	ID=cds380	0	10,33333333	STM14_3931	garL	alpha-dehydro-beta-deoxy-D-glucarate aldolase	1,421001655	-2,256999172
NC_01685	RefSeq	CDS	3436508	3436648	-	0	ID=cds380	0	0	STM14_3932	STM14_3932	hypothetical protein	0	NA
NC_01685	RefSeq	CDS	3436575	3436694	+	0	ID=cds380	0	0	STM14_3933	STM14_3933	hypothetical protein	0	NA
NC_01685	RefSeq	CDS	3436648	3436776	+	0	ID=cds380	0	0	STM14_3934	STM14_3934	hypothetical protein	0	NA
NC_01685	RefSeq	CDS	3436922	3438493	+	0	ID=cds380	0	9	STM14_3935	garD	galactarate dehydratase	1,27890149	-2,207741391
NC_01685	RefSeq	CDS	3438733	3439680	-	0	ID=cds380	0	3	STM14_3936	STM14_3936	putative sugar kinase	0,426300497	-1,498211557
NC_01685	RefSeq	CDS	3439691	3440524	-	0	ID=cds380	0	5,5	STM14_3937	agaR	AGA operon transcriptional repressor	0,852600993	-1,98056697
NC_01685	RefSeq	CDS	3440518	3440661	+	0	ID=cds380	0	0	STM14_3938	STM14_3938	hypothetical protein	0	NA
NC_01685	RefSeq	CDS	3440858	3441712	+	0	ID=cds380	0	0	STM14_3939	gatY_1	tagatose-bisphosphate aldolase	0	NA
NC_01685	RefSeq	CDS	3441723	3442637	+	0	ID=cds380	0	0	STM14_3940	STM14_3940	putative fructose-1-phosphate kinase	0	NA
NC_01685	RefSeq	CDS	3443499	3444542	+	0	ID=cds381	0	0	STM14_3943	STM14_3943	galactitol-1-phosphate dehydrogenase	0	NA
NC_01685	RefSeq	CDS	3444653	3445426	+	0	ID=cds381	0	13	STM14_3944	STM14_3944	galactitol utilization operon transcriptional repressor	1,847302152	-2,356210635
NC_01685	RefSeq	CDS	3445584	3446447	-	0	ID=cds381	0	9	STM14_3945	yraL	putative methyltransferase	1,27890149	-2,207741391
NC_01685	RefSeq	CDS	3446511	3448553	+	0	ID=cds381	0	39,5	STM14_3946	yraM	putative transglycosylase	5,684006621	-2,24950002
NC_01685	RefSeq	CDS	3448511	3448906	+	0	ID=cds381	0	25	STM14_3947	yraN	hypothetical protein	3,552504138	-2,43600272
NC_01685	RefSeq	CDS	3448928	3449518	+	0	ID=cds381	0	23	STM14_3948	yraO	DnaA initiator-associating protein DiaA	3,268303807	-2,432747446
NC_01685	RefSeq	CDS	3449528	3450103	+	0	ID=cds381	0	11	STM14_3949	yraP	hypothetical protein	1,563101821	-2,297202414
NC_01685	RefSeq	CDS	3450170	3450838	-	0	ID=cds381	0	2	STM14_3950	yraR	putative nucleoside-diphosphate-sugar epimerase	0,284200331	-1,182723423
NC_01685	RefSeq	CDS	3450936	3451454	+	0	ID=cds381	0	0	STM14_3951	yhbO	putative intracellular proteinase	0	NA
NC_01685	RefSeq	CDS	3451434	3451877	-	0	ID=cds381	0	9	STM14_3952	yhbP	hypothetical protein	1,27890149	-2,207741391
NC_01685	RefSeq	CDS	3451915	3452232	+	0	ID=cds382	1	0	STM14_3953	yhbQ	GIY-YIG nuclease superfamily protein	1,75932237	1,868512432
NC_01685	RefSeq	CDS	3452219	3452722	-	0	ID=cds382	0	39	STM14_3954	yhbS	putative transport protein	5,541906456	-2,25579729
NC_01685	RefSeq	CDS	3452716	3453240	-	0	ID=cds382	0	54,66666667	STM14_3955	yhbT	putative lipid carrier protein	7,815509104	-2,137668811
NC_01685	RefSeq	CDS	3453457	3454452	+	0	ID=cds382	0	13	STM14_3956	yhbU	putative protease	1,847302152	-2,356210635
NC_01685	RefSeq	CDS	3454461	3455339	+	0	ID=cds382	0	24,5	STM14_3957	yhbV	putative protease	3,410403973	-2,434086198
NC_01685	RefSeq	CDS	3455517	3456524	+	0	ID=cds382	0	7	STM14_3958	yhbW	hypothetical protein	0,994701159	-2,072978628
NC_01685	RefSeq	CDS	3456502	3457221	-	0	ID=cds382	0	1	STM14_3959	STM14_3959	putative inner membrane protein	0,142100166	-0,623264422
NC_01685	RefSeq	CDS	3457380	3457910	+	0	ID=cds382	0	0	STM14_3960	STM14_3960	putative cytoplasmic protein	0	NA
NC_01685	RefSeq	CDS	3457971	3459215	-	0	ID=cds382	0	1	STM14_3961	mtr	tryptophan permease	0,142100166	-0,623264422
NC_01685	RefSeq	CDS	3459369	3461258	-	0	ID=cds382	0	188,5	STM14_3962	deaD	ATP-dependent RNA helicase DeaD	26,71483112	-1,8957776
NC_01685	RefSeq	CDS	3461437	3462321	-	0	ID=cds383	4	383	STM14_3963	nlpI	lipoprotein NlpI	61,46165288	-2,027340277
NC_01685	RefSeq	CDS	3462431	3464566	-	0	ID=cds383	4	272,5	STM14_3964	pnp	polynucleotide phosphorylase%2Fpolyadenylase	45,68853451	-1,68752346
NC_01685	RefSeq	CDS	3464611	3464718	-	0	ID=cds383	0	24,33333333	STM14_3965	STM14_3965	hypothetical protein	3,410403973	-2,434086198
NC_01685	RefSeq	CDS	3464808	3465077	-	0	ID=cds383	0	58,5	STM14_3966	rpsO	30S ribosomal protein S15	8,241809601	-2,117369507
NC_01685	RefSeq	CDS	3465228	3466172	-	0	ID=cds383	0	33,5	STM14_3967	truB	tRNA pseudouridine synthase B	4,831405628	-2,523251848
NC_01685	RefSeq	CDS	3466172	3466573	-	0	ID=cds383	0	7,5	STM14_3968	rbfA	ribosome-binding factor A	1,136801324	-2,147403465
NC_01685	RefSeq	CDS	3466794	3469472	-	0	ID=cds383	2	244,1666667	STM14_3969	infB	translation initiation factor IF-2	38,19108513	-2,191347028
NC_01685	RefSeq	CDS	3469497	3470999	-	0	ID=cds383	2	204,0833333	STM14_3970	nusA	transcription elongation factor NusA	32,50707851	-2,024295291
NC_01685	RefSeq	CDS	3471027	3471479	-	0	ID=cds383	0	47,5	STM14_3971	yhbC	hypothetical protein	6,820807946	-2,185729286
NC_01685	RefSeq	CDS	3472107	3473450	+	0	ID=cds383	0	4	STM14_3973	argG	argininosuccinate synthase	0,568400662	-1,710438693
NC_01685	RefSeq	CDS	3473536	3474600	-	0	ID=cds384	0	8	STM14_3974	STM14_3974	putative cytoplasmic protein	1,136801324	-2,147403465
NC_01685	RefSeq	CDS	3475036	3475368	-	0	ID=cds384	3	48,66666667	STM14_3976	secG	preprotein translocase subunit SecG	12,24087522	-0,27252227
NC_01685	RefSeq	CDS	3475591	3476928	-	0	ID=cds384	0	37	STM14_3977	glmM	phosphoglucosamine mutase	5,257706125	-2,272984196
NC_01685	RefSeq	CDS	3476921	3477769	-	0	ID=cds384	0	21,83333333	STM14_3978	folP	dihydropteroate synthase	3,126203642	-2,431590384
NC_01685	RefSeq	CDS	3477916	3478374	-	0	ID=cds384	0	3,9	STM14_3979	tnpA_9	transposase	0,568400662	-1,710438693
NC_01685	RefSeq	CDS	3478331	3478519	-	0	ID=cds384	0	2,1	STM14_3980	STM14_3980	hypothetical protein	0,284200331	-1,182723423
NC_01685	RefSeq	CDS	3478585	3480519	-	0	ID=cds384	1	290,3333333	STM14_3981	hflB	ATP-dependent metalloprotease	42,96837038	-2,877730807
NC_01685	RefSeq	CDS	3480623	3481249	-	0	ID=cds384	0	44,5	STM14_3982	rrmJ	23S rRNA methyltransferase J	6,252407284	-2,216733793
NC_01685	RefSeq	CDS	3481376	3481669	+	0	ID=cds384	0	16	STM14_3983	yhbY	RNA-binding protein YhbY	2,273602649	-2,403729808
NC_01685	RefSeq	CDS	3481825	3482301	-	0	ID=cds384	0	42	STM14_3984	greA	transcription elongation factor GreA	5,968206953	-2,232890319
NC_01685	RefSeq	CDS	3482355	3482456	-	0	ID=cds385	0	1	STM14_3985	STM14_3985	hypothetical protein	0,142100166	-0,623264422
NC_01685	RefSeq	CDS	3482549	3483982	+	0	ID=cds385	0	12	STM14_3986	dacB	D-alanyl-D-alanine carboxypeptidase	1,705201986	-2,329925387
NC_01685	RefSeq	CDS	3483983	3484117	+	0	ID=cds385	0	0	STM14_3987	STM14_3987	hypothetical protein	0	NA
NC_01685	RefSeq	CDS	3484114	3485286	-	0	ID=cds385	0	73,66666667	STM14_3988	obgE	GTPase ObgE	10,51541225	-2,024396986
NC_01685	RefSeq	CDS	3486397	3486654	-	0	ID=cds385	0	96	STM14_3990	rpmA	50S ribosomal protein L27	13,64161589	-1,920025083
NC_01685	RefSeq	CDS	3486674	3486985	-	0	ID=cds385	1	112	STM14_3991	rplU	50S ribosomal protein L21	17,67454091	-2,062587896

NC_016856	RefSeq	CDS	3487069	3488214	+	0	ID=cds3856	0	26,5	STM14_3992	ispB	octaprenyl diphosphate synthase	3,694604304	-2,438835558
NC_016856	RefSeq	CDS	3488446	3488733	+	0	ID=cds3857	0	1	STM14_3993	nlp	DNA-binding transcriptional regulator Nlp	0,142100166	-0,623264422
NC_016856	RefSeq	CDS	3488791	3490050	-	0	ID=cds3858	3	27,83333333	STM14_3994	murA	UDP-N-acetylglucosamine 1-carboxyvinyltransferase	9,256771745	0,271721137
NC_016856	RefSeq	CDS	3490104	3490397	-	0	ID=cds3859	0	9,5	STM14_3995	yrbA	putative transcriptional regulator	1,421001655	-2,256999172
NC_016856	RefSeq	CDS	3490436	3490546	-	0	ID=cds3860	0	0,5	STM14_3996	STM14_3996	hypothetical protein	0	NA
NC_016856	RefSeq	CDS	3490546	3490842	-	0	ID=cds3861	0	10,5	STM14_3997	yrbB	hypothetical protein	1,421001655	-2,256999172
NC_016856	RefSeq	CDS	3490842	3491477	-	0	ID=cds3862	0	22,5	STM14_3998	yrbC	putative transport protein	3,126203642	-2,431590384
NC_016856	RefSeq	CDS	3491496	3492047	-	0	ID=cds3863	0	9	STM14_3999	yrbD	putative transport protein	1,27890149	-2,207741391
NC_016856	RefSeq	CDS	3492052	3492834	-	0	ID=cds3864	0	14,5	STM14_4000	yrbE	putative transport protein	1,989402318	-2,376535557
NC_016856	RefSeq	CDS	3492842	3493654	-	0	ID=cds3865	0	22,5	STM14_4001	yrbF	putative ABC transporter ATP-binding protein YrbF	3,126203642	-2,431590384
NC_016856	RefSeq	CDS	3493867	3494844	+	0	ID=cds3866	3	32,5	STM14_4002	yrbG	putative calcium%2Fsodium:proton antiporter	9,825172407	0,144080782
NC_016856	RefSeq	CDS	3494858	3495844	+	0	ID=cds3867	5	23,5	STM14_4003	yrbH	D-arabinose 5-phosphate isomerase	12,20701582	0,932342952
NC_016856	RefSeq	CDS	3495865	3496431	+	0	ID=cds3868	0	18	STM14_4004	yrbI	3-deoxy-D-manno-octulosonate 8-phosphate phosphatase	2,55780298	-2,419166771
NC_016856	RefSeq	CDS	3496428	3497003	+	0	ID=cds3869	0	30,25	STM14_4005	yrbK	hypothetical protein	4,263004966	-2,464060063
NC_016856	RefSeq	CDS	3496972	3497526	+	0	ID=cds3870	0	18,75	STM14_4006	yhbN	hypothetical protein	2,699903145	-2,42419778
NC_016856	RefSeq	CDS	3497533	3498258	+	0	ID=cds3871	0	27	STM14_4007	yhbG	putative ABC transporter ATP-binding protein YhbG	3,836704469	-2,44262576
NC_016856	RefSeq	CDS	3498306	3499739	+	0	ID=cds3872	1	101,75	STM14_4008	rpoN	RNA polymerase factor sigma-54	16,25353925	-1,976443334
NC_016856	RefSeq	CDS	3499762	3500049	+	0	ID=cds3873	4	30,25	STM14_4009	yhbH	putative sigma%2854%29 modulation protein	11,30029445	0,491490062
NC_016856	RefSeq	CDS	3500167	3500658	+	0	ID=cds3874	0	41	STM14_4010	ptsN	sugar-specific enzyme IIA component of PTS	5,826106787	-2,241137745
NC_016856	RefSeq	CDS	3500704	3501558	+	0	ID=cds3875	1	33,5	STM14_4011	yhbJ	hypothetical protein	6,590727998	-0,915216377
NC_016856	RefSeq	CDS	3501555	3501827	+	0	ID=cds3876	0	6,5	STM14_4012	ptsO	phosphohistidinoprotein-hexose phosphotransferase com	0,852600993	-1,98056697
NC_016856	RefSeq	CDS	3502076	3502708	+	0	ID=cds3877	0	0	STM14_4013	yrbL	hypothetical protein	0	NA
NC_016856	RefSeq	CDS	3502783	3503511	-	0	ID=cds3878	0	6,75	STM14_4014	mtgA	monofunctional biosynthetic peptidoglycan transglycosyla	0,994701159	-2,072978628
NC_016856	RefSeq	CDS	3503508	3504161	-	0	ID=cds3879	0	22,41666667	STM14_4015	yhbL	isoprenoid biosynthesis protein	3,126203642	-2,431590384
NC_016856	RefSeq	CDS	3504388	3506724	-	0	ID=cds3880	0	42,5	STM14_4016	arcB	aerobic respiration control sensor protein ArcB	5,968206953	-2,232890319
NC_016856	RefSeq	CDS	3506818	3507747	-	0	ID=cds3881	0	5,5	STM14_4017	yhcC	putative FeS oxidoreductase	0,852600993	-1,98056697
NC_016856	RefSeq	CDS	3508092	3508241	-	0	ID=cds3882	0	0	STM14_4018	STM14_4018	hypothetical protein	0	NA
NC_016856	RefSeq	CDS	3508281	3508475	-	0	ID=cds3883	0	0	STM14_4019	STM14_4019	hypothetical protein	0	NA
NC_016856	RefSeq	CDS	3508419	3512879	+	0	ID=cds3884	0	19,5	STM14_4020	gltB	glutamate synthase subunit alpha	2,842003311	-2,427810709
NC_016856	RefSeq	CDS	3512889	3514307	+	0	ID=cds3885	1	4,5	STM14_4021	gltD	glutamate synthase subunit beta	2,327723032	1,125268706
NC_016856	RefSeq	CDS	3514514	3515617	+	0	ID=cds3886	0	0	STM14_4022	yhcG	putative cytoplasmic protein	0	NA
NC_016856	RefSeq	CDS	3515633	3515794	+	0	ID=cds3887	0	0	STM14_4023	STM14_4023	hypothetical protein	0	NA
NC_016856	RefSeq	CDS	3515734	3516987	+	0	ID=cds3888	0	0	STM14_4024	codB	cytosine permease	0	NA
NC_016856	RefSeq	CDS	3516974	3518254	+	0	ID=cds3889	0	5,5	STM14_4025	STM14_4025	cytosine deaminase	0,852600993	-1,98056697
NC_016856	RefSeq	CDS	3518337	3518804	-	0	ID=cds3890	0	3,5	STM14_4026	yhcH	putative cytoplasmic protein	0,568400662	-1,710438693
NC_016856	RefSeq	CDS	3518801	3519676	-	0	ID=cds3891	0	3	STM14_4027	nanK	N-acetylmannosamine kinase	0,426300497	-1,498211557
NC_016856	RefSeq	CDS	3519673	3520362	-	0	ID=cds3892	0	11,5	STM14_4028	nanE	N-acetylmannosamine-6-phosphate 2-epimerase	1,705201986	-2,329925387
NC_016856	RefSeq	CDS	3520409	3521899	-	0	ID=cds3893	0	11,5	STM14_4029	nanT	putative sialic acid transporter	1,705201986	-2,329925387
NC_016856	RefSeq	CDS	3522015	3522908	-	0	ID=cds3894	0	15,66666667	STM14_4030	nanA	N-acetylneuraminate lyase	2,273602649	-2,403729808
NC_016856	RefSeq	CDS	3523043	3523834	-	0	ID=cds3895	0	21	STM14_4031	yhcK	transcriptional regulator NanR	2,984103476	-2,430148754
NC_016856	RefSeq	CDS	3523943	3524443	-	0	ID=cds3896	2	47,5	STM14_4032	sspB	ClpXP protease specificity-enhancing factor	10,33945269	-0,634968955
NC_016856	RefSeq	CDS	3524449	3525087	-	0	ID=cds3897	2	154	STM14_4033	sspA	stringent starvation protein A	25,40207023	-1,768858527
NC_016856	RefSeq	CDS	3525086	3525205	+	0	ID=cds3898	0	0	STM14_4034	STM14_4034	hypothetical protein	0	NA
NC_016856	RefSeq	CDS	3525350	3526102	-	0	ID=cds3899	0	18	STM14_4035	STM14_4035	putative cytoplasmic protein	2,55780298	-2,419166771
NC_016856	RefSeq	CDS	3526302	3526694	-	0	ID=cds3900	0	146,5	STM14_4036	rpsI	30S ribosomal protein S9	20,74662417	-1,873434052
NC_016856	RefSeq	CDS	3526710	3527138	-	0	ID=cds3901	0	143,5	STM14_4037	rplM	50S ribosomal protein L13	20,46242384	-1,87208996
NC_016856	RefSeq	CDS	3527441	3528565	-	0	ID=cds3902	0	40,5	STM14_4038	yhcM	putative ATPase	5,684006621	-2,24950002
NC_016856	RefSeq	CDS	3528752	3529156	+	0	ID=cds3903	1	19,5	STM14_4039	yhcB	cytochrome d ubiquinol oxidase subunit III	4,601325681	-0,429413798
NC_016856	RefSeq	CDS	3529313	3530680	+	0	ID=cds3904	0	34	STM14_4040	degQ	serine endoprotease	4,831405628	-2,523251848
NC_016856	RefSeq	CDS	3530773	3531843	+	0	ID=cds3905	0	10,5	STM14_4041	degS	serine endoprotease	1,421001655	-2,256999172
NC_016856	RefSeq	CDS	3531877	3532608	-	0	ID=cds3906	1	1,5	STM14_4042	STM14_4042	putative inner membrane protein	2,043522701	1,824239109
NC_016856	RefSeq	CDS	3532628	3533929	-	0	ID=cds3907	0	2,5	STM14_4043	oadB	putative sodium ion pump oxaloacetate decarboxylase be	0,284200331	-1,182723423
NC_016856	RefSeq	CDS	3533945	3535720	-	0	ID=cds3908	0	1,333333333	STM14_4044	oadA	pyruvate carboxylase subunit B	0,142100166	-0,623264422
NC_016856	RefSeq	CDS	3535736	3535990	-	0	ID=cds3909	0	0	STM14_4045	oadG	oxaloacetate decarboxylase subunit gamma	0	NA
NC_016856	RefSeq	CDS	3536146	3536763	-	0	ID=cds3910	0	1	STM14_4046	STM14_4046	L%28%2B%29-tartrate dehydratase subunit beta	0,142100166	-0,623264422
NC_016856	RefSeq	CDS	3536763	3537662	-	0	ID=cds3911	0	0	STM14_4047	STM14_4047	tartrate dehydratase subunit alpha	0	NA
NC_016856	RefSeq	CDS	3537695	3538963	-	0	ID=cds3912	0	0,5	STM14_4048	STM14_4048	putative cation transporter	0	NA
NC_016856	RefSeq	CDS	3539174	3539839	-	0	ID=cds3913	0	14,5	STM14_4049	STM14_4049	putative regulatory protein	1,989402318	-2,376535557
NC_016856	RefSeq	CDS	3539826	3540455	-	0	ID=cds3914	0	8,833333333	STM14_4050	STM14_4050	putative regulatory protein	1,27890149	-2,207741391
NC_016856	RefSeq	CDS	3540466	3540597	-	0	ID=cds3915	0	6,25	STM14_4051	STM14_4051	malate dehydrogenase	0,852600993	-1,98056697
NC_016856	RefSeq	CDS	3540575	3541513	-	0	ID=cds3916	0	85,75	STM14_4052	mdh	malate dehydrogenase	12,22061424	-1,963849549
NC_016856	RefSeq	CDS	3541795	3541923	+	0	ID=cds3917	0	4,5	STM14_4053	STM14_4053	hypothetical protein	0,568400662	-1,710438693
NC_016856	RefSeq	CDS	3541927	3542397	+	0	ID=cds3918	4	39,5	STM14_4054	argR	arginine repressor	12,7212961	0,211668787
NC_016856	RefSeq	CDS	3542762	3543025	+	0	ID=cds3919	0	5,5	STM14_4055	yhcN	putative outer membrane protein	0,852600993	-1,98056697
NC_016856	RefSeq	CDS	3543129	3543395	+	0	ID=cds3920	0	2	STM14_4056	STM14_4056	hypothetical protein	0,284200331	-1,182723423
NC_016856	RefSeq	CDS	3543455	3543727	-	0	ID=cds3921	0	1	STM14_4057	yhcO	putative cytoplasmic protein	0,142100166	-0,623264422
NC_016856	RefSeq	CDS	3543899	3545866	-	0	ID=cds3922	0	4	STM14_4058	yhcP	p-hydroxybenzoic acid efflux subunit AaeB	0,568400662	-1,710438693
NC_016856	RefSeq	CDS	3545872	3546804	-	0	ID=cds3923	0	3,5	STM14_4059	yhcQ	p-hydroxybenzoic acid efflux subunit AaeA	0,568400662	-1,710438693
NC_016856	RefSeq	CDS	3546812	3547015	-	0	ID=cds3924	0	1	STM14_4060	yhcR	hypothetical protein	0,142100166	-0,623264422
NC_016856	RefSeq	CDS	3547197	3548126	+	0	ID=cds3925	0	4	STM14_4061	yhcS	putative DNA-binding transcriptional regulator	0,568400662	-1,710438693
NC_016856	RefSeq	CDS	3548249	3549694	-	0	ID=cds3926	3	28,5	STM14_4062	tldD	protease TldD	9,256771745	0,271721137
NC_016856	RefSeq	CDS	3549839	3553639	-	0	ID=cds3927	1	79	STM14_4063	yhdP	hypothetical protein	12,98523545	-1,728751476
NC_016856	RefSeq	CDS	3553746	3555215	-	0	ID=cds3928	0	91,16666667	STM14_4064	cafA	ribonuclease G	12,93111506	-1,941086641
NC_016856	RefSeq	CDS	3555205	3555798	-	0	ID=cds3929	0	64,66666667	STM14_4065	maf_2	Maf-like protein	9,23651076	-2,074135889
NC_016856	RefSeq	CDS	3555807	3556298	-	0	ID=cds3930	0	38	STM14_4066	mreD	rod shape-determining protein MreD	5,39980629	-2,264331336
NC_016856	RefSeq	CDS	3556298	3557350	-	0	ID=cds3931	0	42,5	STM14_4067	mreC	cell wall structural complex MreBCD transmembrane com	5,968206953	-2,232890319
NC_016856	RefSeq	CDS	3557415	3558458	-	0	ID=cds3932	0	88,5	STM14_4068	mreB	rod shape-determining protein	12,50481457	-1,954585237
NC_016856	RefSeq	CDS	3558566	3558757	+	0	ID=cds3933	0	0	STM14_4069	STM14_4069	hypothetical protein	0	NA
NC_016856	RefSeq	CDS	3558766	3560706	-	0	ID=cds3934	0	1141,916667	STM14_4070	yhdA	regulatory protein CsrD	162,278389	-1,969986862
NC_016856	RefSeq	CDS	3560910	3561884	+	0	ID=cds3935	0	51,5	STM14_4071	yhdH	putative oxidoreductase	7,389208608	-2,158807911
NC_016856	RefSeq	CDS	3561997	3563001	+	0	ID=cds3936	0	12	STM14_4072	STM14_4072	putative sulfite oxidase subunit YedY	1,705201986	-2,329925387
NC_016856	RefSeq	CDS	3563002	3563601	+	0	ID=cds3937	0	6	STM14_4073	STM14_4073	putative sulfite oxidase subunit YedZ	0,852600993	-1,98056697
NC_016856	RefSeq	CDS	3563669	3563851	-	0	ID=cds3938	0	0	STM14_4074	STM14_4074	hypothetical protein	0	NA
NC_016856	RefSeq	CDS	3563867	3563971	+	0	ID=cds3939	0	4,25	STM14_4075	STM14_4075	hypothetical protein	0,568400662	-1,710438693
NC_016856	RefSeq	CDS	3563994	3564464	+	0	ID=cds3940	0	77,25	STM14_4076	accB	acetyl-CoA carboxylase biotin carboxyl carrier protein sub	10,94171275	-2,008412955
NC_016856	RefSeq	CDS	3564475	3565824	+	0	ID=cds3941	0	121,1666667	STM14_4077	accC	acetyl-CoA carboxylase biotin carboxylase subunit	17,19412003	-1,853939051
NC_016856	RefSeq	CDS	3565933	3566175	+	0	ID=cds3942	0	1	STM14_4078	yhdT	hypothetical protein	0,142100166	-0,623264422
NC_016856	RefSeq	CDS	3566165	3567616	+	0	ID=cds3943	0	5	STM14_4079	panF	sodium%2Fpanthothenate symporter	0,710500828	-1,864806942
NC_016856	RefSeq	CDS	3567628	3568509	+	0	ID=cds3944	0	14,5	STM14_4080	prmA	50S ribosomal protein L11 methyltransferase	1,989402318	-2,376535557
NC_016856	RefSeq	CDS	3568595	3568738	-	0	ID=cds3945	0	0	STM14_4081	STM14_4081	hypothetical protein	0	NA
NC_016856	RefSeq	CDS	3569170	3570135	+	0	ID=cds3946	2	62	STM14_4082	yhdG	tRNA-dihydrouridine synthase B	12,328855	-0,890129043
NC_016856	RefSeq	CDS	3570161	3570457	+	0	ID=cds3947	0	24,5	STM14_4083	fis	DNA-binding protein Fis	3,410403973	-2,434086198
NC_016856	RefSeq	CDS	3570543	3571427	+	0	ID=cds3948	0	2	STM14_4084	yhdJ	putative methyltransferase	0,284200331	-1,182723423
NC_016856	RefSeq	CDS	3571510	3571674	+	0	ID=cds3949	0	5	STM14_4085	yhdU	hypothetical protein	0,710500828	-1,864806942
NC_016856	RefSeq	CDS	3571825	3573924	+	0	ID=cds3950	0	0	STM14_4086	STM14_4086	putative signal transduction protein	0	NA
NC_016856	RefSeq	CDS	3573927	3574589	-	0	ID=cds3951	0	0,5	STM14_4087	envR	DNA-binding transcriptional regulator EnvR	0	NA
NC_016856	RefSeq	CDS	3574592	3574753	-	0	ID=cds3952	0	0	STM14_4088	STM14_4088	hypothetical protein	0	NA
NC_016856	RefSeq	CDS	3574662	3574820	+	0	ID=cds3953	0	0	STM14_4089	STM14_4089	hypothetical protein	0	NA
NC_016856	RefSeq	CDS	3575003	3576160	+	0	ID=cds3954	0	0	STM14_4090	acrE	acriflavine resistance protein E	0	NA
NC_016856	RefSeq	CDS	3576172	3579285	+	0	ID=cds3955	0	0	STM14_4091	acrF	multidrug transport protein	0	NA
NC_016856	RefSeq	CDS	3579521	3579742	+	0	ID=cds3956	0	3	STM14_4092	yhdV	putative outer membrane lipoprotein	0,426300497	-1,498211557
NC_016856	RefSeq	CDS	3579746	3579922	+	0	ID=cds3957	0	0	STM14_4093	STM14_4093	hypothetical protein	0	NA
NC_016856	RefSeq	CDS	3580182	3580316	-	0	ID=cds3958	0	1	STM14_4094	STM14_4094	hypothetical protein	0,142100166	-0,623264422
NC_016856	RefSeq	CDS	3580370	3580480	+	0	ID=cds3959	0	0	STM14_4095	STM14_4095	hypothetical protein	0	NA
NC_016856	RefSeq	CDS	3586043	3586165	+	0	ID=cds3960	0	0	STM14_4101	STM14_4101	hypothetical protein	0	NA
NC_016856	RefSeq	CDS	3586340	3586894	+	0	ID=cds3961	0	9,5	STM14_4102	yrdA	putative ferripyochelin-binding protein	1,421001655	-2,256999172
NC_016856	RefSeq	CDS	3586870	3587127	-	0	ID=cds3962	0	3,5	STM14_4103	yrdB	hypothetical protein	0,568400662	-1,710438693
NC_016856	RefSeq	CDS	3587124	3587942	-	0	ID=cds3963	0	22,5	STM14_4104	aroE_2	shikimate 5-dehydrogenase	3,126203642	-2,431590384
NC_016856	RefSeq	CDS	3587947	3588519	-	0	ID=cds3964	0	18,5	STM14_4105	yrdC	putative ribosome maturation factor	2,55780298	-2,419166771
NC_016856	RefSeq	CDS	3588524	3589066	-	0	ID=cds3965	1	26,75	STM14_4106	yrdD	putative DNA topoisomerase	5,596026839	-0,703064376
NC_016856	RefSeq	CDS	3589093	3589566	-	0	ID=cds3966	1	49,75	STM14_4107	smg	hypothetical protein	8,864330647	-1,275787415
NC_016856	RefSeq	CDS	3589538	3590662	-	0	ID=cds3967	0	44,33333333	STM14_4108	smf	hypothetical protein	6,252407284	-2,216733793
NC_016856	RefSeq	CDS	3590794	3591303	+	0	ID=cds3968	0	38,5	STM14_4109	def	peptide deformylase	5,39980629	-2,264331336
NC_016856	RefSeq	CDS	3591319	3592266	+	0	ID=cds3969	0	59	STM14_4110	fmt	methionyl-tRNA formyltransferase	8,383909767	-2,110782333
NC_016856	RefSeq	CDS	3592318	3593607	+	0	ID=cds3970	0	13	STM14_4111	sun	16S rRNA methyltransferase B	1,847302152	-2,356210635
NC_016856	RefSeq	CDS	3593629	3595005	+	0	ID=cds3971	0	11,5	STM14_4112	trkA	potassium transporter peripheral membrane protein	1,705201986	-2,329925387
NC_016856	RefSeq	CDS	3595147	3595560	+	0	ID=cds3972	0	2,5	STM14_4113	mscL	large-conductance mechanosensitive channel	0,284200331	-1,182723423
NC_016856	RefSeq	CDS	3595496	3595765	-	0	ID=cds3973	0	0	STM14_4114	STM14_4114	putative cytoplasmic protein	0	NA
NC_016856	RefSeq	CDS	3595823	3596248	-	0	ID=cds3974	1	2	STM14_4115	zntR	zinc-responsive transcriptional regulator	2,043522701	1,824239109
NC_016856	RefSeq	CDS	3596259	3596627	-	0	ID=cds3975	0	1	STM14_4116	yhdN	hypothetical protein	0,142100166	-0,623264422
NC_016856	RefSeq	CDS	3596735	3597118	-	0	ID=cds3976	7	144,3333333	STM14_4117	rplQ	50S ribosomal protein L17	32,77768043	-0,511842924
NC_016856	RefSeq	CDS	3597159	3598148	-	0	ID=cds3977	0	362	STM14_4118	rpoA	DNA-directed RNA polymerase subunit alpha	51,44025992	-1,936547842
NC_016856	RefSeq	CDS	3598174	3598794	-	0	ID=cds3978	4	412,5	STM14_4119	rpsD	30S ribosomal protein S4	65,58255768	-2,09936251
NC_016856	RefSeq	CDS	3598828	3599217	-	0	ID=cds3979	1	318,25	STM14_4120	rpsK	30S ribosomal protein S11	46,94717501	-2,959984013

NC_016856	RefSeq	CDS	3599234	3599590	-	0	ID=cds3980	86	828,4166667	STM14_4121	rpsM	30S ribosomal protein S13	268,9606609	0,252819796
NC_016856	RefSeq	CDS	3599737	3599853	-	0	ID=cds3981	0	28,5	STM14_4122	rpmJ_2	50S ribosomal protein L36	3,978804635	-2,448045729
NC_016856	RefSeq	CDS	3599885	3601216	-	0	ID=cds3982	5	624,75	STM14_4123	secY	preprotein translocase subunit SecY	97,60921531	-2,30286502
NC_016856	RefSeq	CDS	3601224	3601658	-	0	ID=cds3983	0	177,5	STM14_4124	rplO	50S ribosomal protein L15	25,29382947	-1,891303465
NC_016856	RefSeq	CDS	3601662	3601841	-	0	ID=cds3984	0	44,5	STM14_4125	rpmD	50S ribosomal protein L30	6,252407284	-2,216733793
NC_016856	RefSeq	CDS	3601845	3602348	-	0	ID=cds3985	0	184,3333333	STM14_4126	rpsE	30S ribosomal protein S5	26,14643046	-1,894039656
NC_016856	RefSeq	CDS	3602363	3602716	-	0	ID=cds3986	5	180,0833333	STM14_4127	rplR	50S ribosomal protein L18	34,37464165	-1,06589952
NC_016856	RefSeq	CDS	3602726	3603259	-	0	ID=cds3987	1	211	STM14_4128	rplF	50S ribosomal protein L6	31,7424573	-2,599718775
NC_016856	RefSeq	CDS	3603272	3603664	-	0	ID=cds3988	0	132,5	STM14_4129	rpsH	30S ribosomal protein S8	18,75722185	-1,863295025
NC_016856	RefSeq	CDS	3603698	3603988	-	0	ID=cds3989	0	198	STM14_4130	rpsN	30S ribosomal protein S14	28,13583278	-1,899848195
NC_016856	RefSeq	CDS	3604018	3604557	-	0	ID=cds3990	3	375,3333333	STM14_4131	rplE	50S ribosomal protein L5	58,56552919	-2,261087885
NC_016856	RefSeq	CDS	3604572	3604886	-	0	ID=cds3991	3	133	STM14_4132	rplX	50S ribosomal protein L24	24,17728913	-1,260856582
NC_016856	RefSeq	CDS	3604897	3605268	-	0	ID=cds3992	10	244,1666667	STM14_4133	rplN	50S ribosomal protein L14	52,26566409	-0,69272717
NC_016856	RefSeq	CDS	3605432	3605686	-	0	ID=cds3993	0	100,5	STM14_4134	rpsQ	30S ribosomal protein S17	14,21001655	-1,905313172
NC_016856	RefSeq	CDS	3605686	3605877	-	0	ID=cds3994	3	111	STM14_4135	rpmC	50S ribosomal protein L29	21,05108548	-1,086588725
NC_016856	RefSeq	CDS	3605877	3606287	-	0	ID=cds3995	2	72,08333333	STM14_4136	rplP	50S ribosomal protein L16	13,74985666	-1,040341688
NC_016856	RefSeq	CDS	3606300	3607001	-	0	ID=cds3996	1,5	171,75	STM14_4137	rpsC	30S ribosomal protein S3	27,95987321	-1,869169386
NC_016856	RefSeq	CDS	3607019	3607351	-	0	ID=cds3997	2,5	154	STM14_4138	rplV	50S ribosomal protein L22	25,40207023	-1,768858527
NC_016856	RefSeq	CDS	3607366	3607644	-	0	ID=cds3998	0	40,5	STM14_4139	rpsS	30S ribosomal protein S19	5,684006621	-2,24950002
NC_016856	RefSeq	CDS	3607661	3608482	-	0	ID=cds3999	3	112,8333333	STM14_4140	rplB	50S ribosomal protein L2	21,33528582	-1,103929478
NC_016856	RefSeq	CDS	3608500	3608802	-	0	ID=cds4000	0	116	STM14_4141	rplW	50S ribosomal protein L23	16,4836192	-1,849193185
NC_016856	RefSeq	CDS	3608799	3609404	-	0	ID=cds4001	0	225,5	STM14_4142	rplD	50S ribosomal protein L4	32,11463741	-1,909513572
NC_016856	RefSeq	CDS	3609415	3610044	-	0	ID=cds4002	1	164	STM14_4143	rplC	50S ribosomal protein L3	25,06374952	-2,391237981
NC_016856	RefSeq	CDS	3610077	3610388	-	0	ID=cds4003	3	95,5	STM14_4144	rpsJ	30S ribosomal protein S10	18,919583	-0,944579571
NC_016856	RefSeq	CDS	3610642	3611235	+	0	ID=cds4004	0	0	STM14_4145	hopD	leader peptidase HopD	0	NA
NC_016856	RefSeq	CDS	3611232	3611708	-	0	ID=cds4005	1	8,5	STM14_4146	bfr	bacterioferritin%2C iron storage and detoxification protei	2,896123694	0,419298367
NC_016856	RefSeq	CDS	3611781	3611975	-	0	ID=cds4006	1,5	0	STM14_4147	bfd	bacterioferritin-associated ferredoxin	3,51864474	1,925445802
NC_016856	RefSeq	CDS	3611988	3612179	-	0	ID=cds4007	1,5	3,25	STM14_4148	STM14_4148	elongation factor Tu	3,944945236	1,874572685
NC_016856	RefSeq	CDS	3612160	3613344	-	0	ID=cds4008	5,5	688,25	STM14_4149	tuf_1	elongation factor Tu	108,3208481	-2,22791997
NC_016856	RefSeq	CDS	3613416	3615530	-	0	ID=cds4009	0	595	STM14_4150	fusA	elongation factor G	84,54959849	-1,955326002
NC_016856	RefSeq	CDS	3615627	3616097	-	0	ID=cds4010	3	155	STM14_4151	rpsG	30S ribosomal protein S7	27,30349277	-1,406495975
NC_016856	RefSeq	CDS	3616193	3616567	-	0	ID=cds4011	0	67,83333333	STM14_4152	rpsL	30S ribosomal protein S12	9,662811256	-2,058244678
NC_016856	RefSeq	CDS	3616693	3616980	-	0	ID=cds4012	0	6,5	STM14_4153	yheL	tRNA 2-thiouridine synthesizing protein B	0,852600993	-1,98056697
NC_016856	RefSeq	CDS	3616988	3617344	-	0	ID=cds4013	0	21,5	STM14_4154	yheM	hypothetical protein	3,126203642	-2,431590384
NC_016856	RefSeq	CDS	3617344	3617730	-	0	ID=cds4014	0	23	STM14_4155	yheN	hypothetical protein	3,268303807	-2,432747446
NC_016856	RefSeq	CDS	3617730	3618452	-	0	ID=cds4015	0	46	STM14_4156	yheO	putative regulatory protein	6,536607615	-2,201017198
NC_016856	RefSeq	CDS	3618729	3619547	-	0	ID=cds4016	4	150,8333333	STM14_4157	fkpA	FKBP-type peptidyl-prolyl cis-trans isomerase	28,49441448	-1,108638258
NC_016856	RefSeq	CDS	3619767	3619985	+	0	ID=cds4017	0	1	STM14_4158	slyX	hypothetical protein	0,142100166	-0,623264422
NC_016856	RefSeq	CDS	3620173	3620763	-	0	ID=cds4018	0	61,83333333	STM14_4159	slyD	FKBP-type peptidyl-prolyl cis-trans isomerase	8,810210263	-2,091539774
NC_016856	RefSeq	CDS	3620858	3621058	-	0	ID=cds4019	2,5	0,5	STM14_4160	yheV	putative cytoplasmic protein	3,51864474	1,925445802
NC_016856	RefSeq	CDS	3621069	3622874	-	0	ID=cds4020	4,5	13,33333333	STM14_4161	kefB	glutathione-regulated potassium-efflux system protein Ke	8,884591632	1,268616464
NC_016856	RefSeq	CDS	3622874	3623425	-	0	ID=cds4021	0	0	STM14_4162	yheR	glutathione-regulated potassium-efflux system ancillary p	0	NA
NC_016856	RefSeq	CDS	3623551	3623655	+	0	ID=cds4022	0	2	STM14_4163	STM14_4163	hypothetical protein	0,284200331	-1,182723423
NC_016856	RefSeq	CDS	3623691	3625598	+	0	ID=cds4023	0	19	STM14_4164	yheS	putative ABC transporter ATP-binding protein	2,699903145	-2,42419778
NC_016856	RefSeq	CDS	3625984	3626295	-	0	ID=cds4024	0	22	STM14_4166	STM14_4166	hypothetical protein	3,126203642	-2,431590384
NC_016856	RefSeq	CDS	3626355	3626480	+	0	ID=cds4025	0	1	STM14_4167	STM14_4167	hypothetical protein	0,142100166	-0,623264422
NC_016856	RefSeq	CDS	3626493	3627560	+	0	ID=cds4026	3	19	STM14_4168	yheT	putative hydrolase	7,977870255	0,635561419
NC_016856	RefSeq	CDS	3627557	3627775	+	0	ID=cds4027	0	2	STM14_4169	yheU	hypothetical protein	0,284200331	-1,182723423
NC_016856	RefSeq	CDS	3627827	3628696	+	0	ID=cds4028	0	7	STM14_4170	prkB	putative phosphoribulokinase	0,994701159	-2,072978628
NC_016856	RefSeq	CDS	3628792	3629196	-	0	ID=cds4029	0	26	STM14_4171	yhfA	hypothetical protein	3,694604304	-2,438835558
NC_016856	RefSeq	CDS	3629162	3629344	+	0	ID=cds4030	0	0,5	STM14_4172	STM14_4172	hypothetical protein	0	NA
NC_016856	RefSeq	CDS	3629504	3630136	+	0	ID=cds4031	0	74	STM14_4173	crp	cAMP-regulatory protein	10,51541225	-2,024396986
NC_016856	RefSeq	CDS	3630185	3632272	+	0	ID=cds4032	0	45,5	STM14_4174	yhfK	putative inner membrane protein	6,536607615	-2,201017198
NC_016856	RefSeq	CDS	3632315	3633532	-	0	ID=cds4033	0	6,5	STM14_4175	argD_2	bifunctional N-succinyldiaminopimelate-aminotransferase	0,852600993	-1,98056697
NC_016856	RefSeq	CDS	3633618	3634181	-	0	ID=cds4034	0	3,5	STM14_4176	pabA	para-aminobenzoate synthase component II	0,568400662	-1,710438693
NC_016856	RefSeq	CDS	3634213	3634815	-	0	ID=cds4035	0	7,833333333	STM14_4177	fic	cell filamentation protein Fic	1,136801324	-2,147403465
NC_016856	RefSeq	CDS	3634805	3634972	-	0	ID=cds4036	0	0,5	STM14_4178	yhfG	hypothetical protein	0	NA
NC_016856	RefSeq	CDS	3635081	3635653	-	0	ID=cds4037	0	14	STM14_4179	ppiA	peptidyl-prolyl cis-trans isomerase A	1,989402318	-2,376535557
NC_016856	RefSeq	CDS	3635785	3635937	+	0	ID=cds4038	0	0	STM14_4180	STM14_4180	hypothetical protein	0	NA
NC_016856	RefSeq	CDS	3635930	3637111	+	0	ID=cds4039	0	1	STM14_4181	yhfC	hypothetical protein	0,142100166	-0,623264422
NC_016856	RefSeq	CDS	3637245	3637421	-	0	ID=cds4040	0	0,5	STM14_4182	STM14_4182	hypothetical protein	0	NA
NC_016856	RefSeq	CDS	3637374	3639917	+	0	ID=cds4041	0	3	STM14_4183	nirB	nitrite reductase large subunit	0,426300497	-1,498211557
NC_016856	RefSeq	CDS	3639914	3640240	+	0	ID=cds4042	0	0	STM14_4184	nirD	nitrite reductase small subunit	0	NA
NC_016856	RefSeq	CDS	3640241	3640396	-	0	ID=cds4043	0	0	STM14_4185	STM14_4185	hypothetical protein	0	NA
NC_016856	RefSeq	CDS	3640506	3641315	+	0	ID=cds4044	0	2	STM14_4186	nirC	nitrite transporter NirC	0,284200331	-1,182723423
NC_016856	RefSeq	CDS	3641327	3642700	+	0	ID=cds4045	2	16	STM14_4187	cysG	siroheme synthase	5,792247388	0,405774795
NC_016856	RefSeq	CDS	3643029	3648923	+	0	ID=cds4046	0	0,5	STM14_4188	bigA	putative surface-exposed virulence protein	0	NA
NC_016856	RefSeq	CDS	3649055	3649513	-	0	ID=cds4047	0	3,4	STM14_4189	tnpA_10	transposase for IS200	0,426300497	-1,498211557
NC_016856	RefSeq	CDS	3649470	3649658	-	0	ID=cds4048	0	1,6	STM14_4190	STM14_4190	hypothetical protein	0,284200331	-1,182723423
NC_016856	RefSeq	CDS	3649827	3649997	+	0	ID=cds4049	0	1	STM14_4191	yhfL	putative outer membrane lipoprotein	0,142100166	-0,623264422
NC_016856	RefSeq	CDS	3649991	3650131	-	0	ID=cds4050	0	1,833333333	STM14_4192	STM14_4192	hypothetical protein	0,284200331	-1,182723423
NC_016856	RefSeq	CDS	3650142	3651146	-	0	ID=cds4051	5,5	19,58333333	STM14_4193	trpS	tryptophanyl-tRNA synthetase	13,39793753	1,293149792
NC_016856	RefSeq	CDS	3651139	3651897	-	0	ID=cds4052	0	13,75	STM14_4194	gph	phosphoglycolate phosphatase	1,989402318	-2,376535557
NC_016856	RefSeq	CDS	3651890	3652567	-	0	ID=cds4053	0	19	STM14_4195	rpe	ribulose-phosphate 3-epimerase	2,699903145	-2,42419778
NC_016856	RefSeq	CDS	3652585	3653421	-	0	ID=cds4054	0	20	STM14_4196	dam	DNA adenine methylase	2,842003311	-2,427810709
NC_016856	RefSeq	CDS	3653602	3654879	-	0	ID=cds4055	0	101,5	STM14_4197	damX	hypothetical protein	14,49421688	-1,89737012
NC_016856	RefSeq	CDS	3654977	3656065	-	0	ID=cds4056	0	97	STM14_4198	aroB	3-dehydroquinate synthase	13,78371606	-1,916716097
NC_016856	RefSeq	CDS	3656122	3656643	-	0	ID=cds4057	0	63,83333333	STM14_4199	aroK	shikimate kinase I	9,094410594	-2,079502768
NC_016856	RefSeq	CDS	3657108	3658346	-	0	ID=cds4058	0	5	STM14_4200	hofQ	outer membrane porin HofQ	0,710500828	-1,864806942
NC_016856	RefSeq	CDS	3658261	3658662	-	0	ID=cds4059	0	1	STM14_4201	yrfA	putative inner membrane protein	0,142100166	-0,623264422
NC_016856	RefSeq	CDS	3658652	3659125	-	0	ID=cds4060	0	2,5	STM14_4202	yrfB	putative inner membrane protein	0,284200331	-1,182723423
NC_016856	RefSeq	CDS	3659109	3659648	-	0	ID=cds4061	0	0	STM14_4203	yrfC	putative inner membrane protein	0	NA
NC_016856	RefSeq	CDS	3659648	3660427	-	0	ID=cds4062	0	3,5	STM14_4204	yrfD	hypothetical protein	0,568400662	-1,710438693
NC_016856	RefSeq	CDS	3660524	3663100	+	0	ID=cds4063	0	33	STM14_4205	mrcA	peptidoglycan synthetase	4,689305463	-2,505858154
NC_016856	RefSeq	CDS	3663196	3663759	-	0	ID=cds4064	3	19,5	STM14_4206	nudE	ADP-ribose diphosphatase NudE	8,119970421	0,58795304
NC_016856	RefSeq	CDS	3663870	3663983	-	0	ID=cds4065	0	0	STM14_4207	STM14_4207	hypothetical protein	0	NA
NC_016856	RefSeq	CDS	3664082	3666214	+	0	ID=cds4066	0	33,5	STM14_4208	yrfF	putative inner membrane protein	4,831405628	-2,523251848
NC_016856	RefSeq	CDS	3666279	3666947	+	0	ID=cds4067	0	4,25	STM14_4209	yrfG	putative hydrolase	0,568400662	-1,710438693
NC_016856	RefSeq	CDS	3666958	3667359	+	0	ID=cds4068	0	6,25	STM14_4210	yrfH	ribosome-associated heat shock protein Hsp15	0,852600993	-1,98056697
NC_016856	RefSeq	CDS	3667384	3668262	+	0	ID=cds4069	0	12,5	STM14_4211	hslO	Hsp33-like chaperonin	1,705201986	-2,329925387
NC_016856	RefSeq	CDS	3668372	3670081	-	0	ID=cds4070	0	6,5	STM14_4212	yhgE	putative inner membrane protein	0,852600993	-1,98056697
NC_016856	RefSeq	CDS	3670273	3670407	-	0	ID=cds4071	0	0	STM14_4213	STM14_4213	hypothetical protein	0	NA
NC_016856	RefSeq	CDS	3670342	3670449	+	0	ID=cds4072	0,5	4,25	STM14_4214	STM14_4214	hypothetical protein	0,568400662	-1,710438693
NC_016856	RefSeq	CDS	3670461	3672080	+	0	ID=cds4073	0	191	STM14_4215	pckA	phosphoenolpyruvate carboxykinase	27,14113162	-1,897038569
NC_016856	RefSeq	CDS	3672155	3673507	-	0	ID=cds4074	0	31,33333333	STM14_4216	envZ	osmolarity sensor protein	4,405105132	-2,475774913
NC_016856	RefSeq	CDS	3673504	3674223	-	0	ID=cds4075	0	15	STM14_4217	ompR	osmolarity response regulator	2,131502483	-2,392032191
NC_016856	RefSeq	CDS	3674449	3674922	+	0	ID=cds4076	0	4	STM14_4218	greB	transcription elongation factor GreB	0,568400662	-1,710438693
NC_016856	RefSeq	CDS	3675024	3677351	+	0	ID=cds4077	0	28	STM14_4219	yhgF	putative RNase R	3,978804635	-2,448045729
NC_016856	RefSeq	CDS	3677538	3677768	+	0	ID=cds4078	0	5	STM14_4220	STM14_4220	hypothetical protein	0,710500828	-1,864806942
NC_016856	RefSeq	CDS	3677788	3678015	+	0	ID=cds4079	0	9,5	STM14_4221	feoA	ferrous iron transport protein A	1,421001655	-2,256999172
NC_016856	RefSeq	CDS	3678034	3680352	+	0	ID=cds4080	4	69	STM14_4222	feoB	ferrous iron transport protein B	16,8422009	-0,332900496
NC_016856	RefSeq	CDS	3680365	3680601	+	0	ID=cds4081	0	6	STM14_4223	yhgG	putative cytoplasmic protein	0,852600993	-1,98056697
NC_016856	RefSeq	CDS	3680653	3680766	+	0	ID=cds4082	0	1	STM14_4224	STM14_4224	hypothetical protein	0,142100166	-0,623264422
NC_016856	RefSeq	CDS	3680836	3681750	+	0	ID=cds4083	0	3	STM14_4225	STM14_4225	putative cytoplasmic protein	0,426300497	-1,498211557
NC_016856	RefSeq	CDS	3681786	3682556	-	0	ID=cds4084	0	7	STM14_4226	bioH	carboxylesterase BioH	0,994701159	-2,072978628
NC_016856	RefSeq	CDS	3682594	3683277	+	0	ID=cds4085	0	0	STM14_4227	yhgH	gluconate periplasmic binding protein	0	NA
NC_016856	RefSeq	CDS	3683336	3683911	+	0	ID=cds4086	1	7	STM14_4228	yhgI	putative DNA uptake protein	2,754023529	0,552839346
NC_016856	RefSeq	CDS	3684140	3684289	-	0	ID=cds4087	0	0	STM14_4229	STM14_4229	hypothetical protein	0	NA
NC_016856	RefSeq	CDS	3684288	3685604	+	0	ID=cds4088	0	3	STM14_4230	gntT	high-affinity gluconate permease	0,426300497	-1,498211557
NC_016856	RefSeq	CDS	3685723	3687801	-	0	ID=cds4089	0	19,5	STM14_4231	malQ	4-alpha-glucanotransferase	2,842003311	-2,427810709
NC_016856	RefSeq	CDS	3687811	3690204	-	0	ID=cds4090	0	32,83333333	STM14_4232	malP	maltodextrin phosphorylase	4,689305463	-2,505858154
NC_016856	RefSeq	CDS	3690393	3690500	-	0	ID=cds4091	0	0,5	STM14_4233	STM14_4233	hypothetical protein	0	NA
NC_016856	RefSeq	CDS	3690798	3693503	+	0	ID=cds4092	2	277	STM14_4234	malT	transcriptional regulator MalT	42,88039059	-2,311743778
NC_016856	RefSeq	CDS	3693591	3693866	-	0	ID=cds4093	0	8	STM14_4235	STM14_4235	putative cytoplasmic protein	1,136801324	-2,147403465
NC_016856	RefSeq	CDS	3693869	3694129	-	0	ID=cds4094	0	6,5	STM14_4236	STM14_4236	putative DNA-damage-inducibile protein	0,852600993	-1,98056697
NC_016856	RefSeq	CDS	3694238	3695257	-	0	ID=cds4095	0	7	STM14_4237	rtcA	RNA 3%27-terminal-phosphate cyclase	0,994701159	-2,072978628
NC_016856	RefSeq	CDS	3695261	3696478	-	0	ID=cds4096	0	18	STM14_4238	rtcB	putative cytoplasmic protein	2,55780298	-2,419166771
NC_016856	RefSeq	CDS	3696823	3698376	-	0	ID=cds4097	0	14	STM14_4239	STM14_4239	putative ribonucleoprotein related-protein	1,989402318	-2,376535557
NC_016856	RefSeq	CDS	3698369	3698491	-	0	ID=cds4098	0	2	STM14_4240	STM14_4240	hypothetical protein	0,284200331	-1,182723423
NC_016856	RefSeq	CDS	3698566	3700149	+	0	ID=cds4099	0	8	STM14_4241	rtcR	sigma N	1,136801324	-2,147403465
NC_016856	RefSeq	CDS	3700146	3700904	-	0	ID=cds4100	0	10,5	STM14_4242	glpR	DNA-binding transcriptional repressor GlpR	1,421001655	-2,256999172
NC_016856	RefSeq	CDS	3700901	3701017	-	0	ID=cds4101	0	1	STM14_4243	STM14_4243	hypothetical protein	0,142100166	-0,623264422
NC_016856	RefSeq	CDS	3700998	3701828	-	0	ID=cds4102	0	10,5	STM14_4244	glpG	intramembrane serine protease GlpG	1,421001655	-2,256999172
NC_016856	RefSeq	CDS	3701904	3702230	-	0	ID=cds4103	0	1	STM14_4245	glpE	thiosulfate sulfurtransferase	0,142100166	-0,623264422

NC_01685(	RefSeq	CDS	3702429	3703937	+	0	ID=cds4104	0	0	STM14_4246	glpD	glycerol-3-phosphate dehydrogenase	0	NA
NC_01685(	RefSeq	CDS	3703984	3704529	-	0	ID=cds4105	0	1	STM14_4247	STM14_4247	hypothetical protein	0,142100166	-0,623264422
NC_01685(	RefSeq	CDS	3704539	3706098	-	0	ID=cds4106	0	6,333333333	STM14_4248	STM14_4248	putative periplasmic phosphate-binding protein	0,852600993	-1,98056697
NC_01685(	RefSeq	CDS	3706192	3707301	-	0	ID=cds4107	0	2,333333333	STM14_4249	gldA_1	glycerol dehydrogenase	0,284200331	-1,182723423
NC_01685(	RefSeq	CDS	3708970	3710685	+	0	ID=cds4108	0	6,5	STM14_4251	STM14_4251	putative dihydroxyacid dehydratase	0,852600993	-1,98056697
NC_01685(	RefSeq	CDS	3710729	3711634	+	0	ID=cds4109	0	1	STM14_4252	STM14_4252	dihydrodipicolinate synthase	0,142100166	-0,623264422
NC_01685(	RefSeq	CDS	3711678	3712433	-	0	ID=cds4110	0	8,5	STM14_4253	STM14_4253	putative transcriptional regulator	1,136801324	-2,147403465
NC_01685(	RefSeq	CDS	3712611	3715058	-	0	ID=cds4111	0	146,3333333	STM14_4254	glgP	glycogen phosphorylase	20,74662417	-1,873434052
NC_01685(	RefSeq	CDS	3715078	3716511	-	0	ID=cds4112	0	38	STM14_4255	glgA	glycogen synthase	5,39980629	-2,264331336
NC_01685(	RefSeq	CDS	3716511	3717806	-	0	ID=cds4113	0	43,5	STM14_4256	glgC	glucose-1-phosphate adenylyltransferase	6,252407284	-2,216733793
NC_01685(	RefSeq	CDS	3717821	3719797	-	0	ID=cds4114	0	31	STM14_4257	glgX	glycogen debranching protein	4,405105132	-2,475774913
NC_01685(	RefSeq	CDS	3719794	3721980	-	0	ID=cds4115	0	41	STM14_4258	glgB	glycogen branching protein	5,826106787	-2,241137745
NC_01685(	RefSeq	CDS	3722125	3722298	-	0	ID=cds4116	0	5	STM14_4259	STM14_4259	hypothetical protein	0,710500828	-1,864806942
NC_01685(	RefSeq	CDS	3722327	3723433	-	0	ID=cds4117	0	59	STM14_4260	asd	aspartate-semialdehyde dehydrogenase	8,383909767	-2,110782333
NC_01685(	RefSeq	CDS	3723463	3723627	+	0	ID=cds4118	0	0	STM14_4261	STM14_4261	hypothetical protein	0	NA
NC_01685(	RefSeq	CDS	3723698	3723940	+	0	ID=cds4119	0	1	STM14_4263	STM14_4263	putative inner membrane protein	0,142100166	-0,623264422
NC_01685(	RefSeq	CDS	3723857	3725197	-	0	ID=cds4120	0	2,5	STM14_4264	gntU	low affinity gluconate transporter	0,284200331	-1,182723423
NC_01685(	RefSeq	CDS	3725194	3725727	-	0	ID=cds4121	0	2	STM14_4265	gntK	gluconate kinase	0,284200331	-1,182723423
NC_01685(	RefSeq	CDS	3725866	3726861	-	0	ID=cds4122	1	17	STM14_4266	gntR	gluconate operon transcriptional repressor	4,175025184	-0,285403406
NC_01685(	RefSeq	CDS	3726991	3727686	-	0	ID=cds4123	0	6	STM14_4267	yhhW	putative cytoplasmic protein	0,852600993	-1,98056697
NC_01685(	RefSeq	CDS	3727810	3728847	-	0	ID=cds4124	0	5	STM14_4268	yhhX	putative dehydrogenase	0,710500828	-1,864806942
NC_01685(	RefSeq	CDS	3728906	3729031	+	0	ID=cds4125	0	0	STM14_4269	STM14_4269	hypothetical protein	0	NA
NC_01685(	RefSeq	CDS	3729191	3729319	+	0	ID=cds4126	0	0	STM14_4270	STM14_4270	hypothetical protein	0	NA
NC_01685(	RefSeq	CDS	3729316	3729804	+	0	ID=cds4127	0	0	STM14_4271	yhhY	putative acetyltransferase YhhY	0	NA
NC_01685(	RefSeq	CDS	3730068	3731282	+	0	ID=cds4128	0	0,5	STM14_4272	STM14_4272	putative transcriptional regulator	0	NA
NC_01685(	RefSeq	CDS	3731298	3732059	+	0	ID=cds4129	0	0	STM14_4273	STM14_4273	putative cytoplasmic protein	0	NA
NC_01685(	RefSeq	CDS	3732113	3733084	+	0	ID=cds4130	0	3	STM14_4274	STM14_4274	putative inner membrane protein	0,426300497	-1,498211557
NC_01685(	RefSeq	CDS	3733081	3734115	+	0	ID=cds4131	0	2	STM14_4275	STM14_4275	putative phosphotriesterase	0,284200331	-1,182723423
NC_01685(	RefSeq	CDS	3734160	3735902	-	0	ID=cds4132	0	13,33333333	STM14_4276	ggt	gamma-glutamyltranspeptidase periplasmic precursor	1,847302152	-2,356210635
NC_01685(	RefSeq	CDS	3735927	3736031	+	0	ID=cds4133	0	0	STM14_4277	STM14_4277	hypothetical protein	0	NA
NC_01685(	RefSeq	CDS	3736028	3736444	+	0	ID=cds4134	2	1	STM14_4278	yhhA	hypothetical protein	3,660744905	2,693976715
NC_01685(	RefSeq	CDS	3736457	3737197	-	0	ID=cds4135	0	9,75	STM14_4279	ugpQ	glycerophosphodiester phosphodiesterase	1,421001655	-2,256999172
NC_01685(	RefSeq	CDS	3737194	3738264	-	0	ID=cds4136	0	9,75	STM14_4280	ugpC	glycerol-3-phosphate transporter ATP-binding subunit	1,421001655	-2,256999172
NC_01685(	RefSeq	CDS	3738266	3739111	-	0	ID=cds4137	0	0,5	STM14_4281	ugpE	glycerol-3-phosphate transporter membrane protein	0	NA
NC_01685(	RefSeq	CDS	3739108	3739995	-	0	ID=cds4138	0	5	STM14_4282	ugpA	glycerol-3-phosphate transporter permease	0,710500828	-1,864806942
NC_01685(	RefSeq	CDS	3740059	3741375	-	0	ID=cds4139	0	1	STM14_4283	ugpB	glycerol-3-phosphate transporter periplasmic binding pro	0,142100166	-0,623264422
NC_01685(	RefSeq	CDS	3741634	3742002	-	0	ID=cds4140	0	1,5	STM14_4284	STM14_4284	death-on-curing protein	0,284200331	-1,182723423
NC_01685(	RefSeq	CDS	3741999	3742226	-	0	ID=cds4141	0	0	STM14_4285	yhhV	putative cytoplasmic protein	0	NA
NC_01685(	RefSeq	CDS	3742352	3743065	-	0	ID=cds4142	0	0,833333333	STM14_4286	livF	leucine%2Fisoleucine%2Fvaline transporter ATP-binding s	0,142100166	-0,623264422
NC_01685(	RefSeq	CDS	3743067	3743834	-	0	ID=cds4143	0	0,25	STM14_4287	livG	leucine%2Fisoleucine%2Fvaline transporter ATP-binding s	0	NA
NC_01685(	RefSeq	CDS	3743831	3745108	-	0	ID=cds4144	0	0,25	STM14_4288	livM	leucine%2Fisoleucine%2Fvaline transporter permease sub	0	NA
NC_01685(	RefSeq	CDS	3745105	3746031	-	0	ID=cds4145	0	5	STM14_4289	livH	branched-chain amino acid transporter permease subunit	0,710500828	-1,864806942
NC_01685(	RefSeq	CDS	3746091	3747200	-	0	ID=cds4146	0	1,5	STM14_4290	livK	high-affinity branched-chain amino acid transporter	0,284200331	-1,182723423
NC_01685(	RefSeq	CDS	3747318	3747434	-	0	ID=cds4147	0	0	STM14_4291	STM14_4291	hypothetical protein	0	NA
NC_01685(	RefSeq	CDS	3747622	3748005	+	0	ID=cds4148	0	1,5	STM14_4292	yhhK	putative acetyltransferase	0,284200331	-1,182723423
NC_01685(	RefSeq	CDS	3747983	3748117	+	0	ID=cds4149	0	1	STM14_4293	STM14_4293	putative cytoplasmic protein	0,142100166	-0,623264422
NC_01685(	RefSeq	CDS	3748200	3749297	-	0	ID=cds4150	0	2,5	STM14_4294	livJ	high-affinity branched-chain amino acid transporter	0,284200331	-1,182723423
NC_01685(	RefSeq	CDS	3749625	3750479	-	0	ID=cds4151	6	108,5	STM14_4295	rpoH	RNA polymerase factor sigma-32	25,9027521	-0,377277903
NC_01685(	RefSeq	CDS	3750462	3750605	+	0	ID=cds4152	0	0	STM14_4296	STM14_4296	hypothetical protein	0	NA
NC_01685(	RefSeq	CDS	3750725	3751780	-	0	ID=cds4153	0	29	STM14_4297	ftsX	cell division protein FtsX	4,120904801	-2,455089955
NC_01685(	RefSeq	CDS	3751773	3752441	-	0	ID=cds4154	0	26	STM14_4298	ftsE	cell division protein FtsE	3,694604304	-2,438835558
NC_01685(	RefSeq	CDS	3752444	3753919	-	0	ID=cds4155	0	44	STM14_4299	ftsY	cell division protein FtsY	6,252407284	-2,216733793
NC_01685(	RefSeq	CDS	3754045	3754641	+	0	ID=cds4156	0	9	STM14_4300	yhhF	16S rRNA m%282%29G966-methyltransferase	1,27890149	-2,207741391
NC_01685(	RefSeq	CDS	3754628	3754900	+	0	ID=cds4157	0	3	STM14_4301	yhhL	hypothetical protein	0,426300497	-1,498211557
NC_01685(	RefSeq	CDS	3754922	3755293	-	0	ID=cds4158	0	2	STM14_4302	yhhM	putative inner membrane protein	0,284200331	-1,182723423
NC_01685(	RefSeq	CDS	3755435	3756061	+	0	ID=cds4159	0	18	STM14_4303	yhhN	putative inner membrane protein	2,55780298	-2,419166771
NC_01685(	RefSeq	CDS	3756142	3758340	+	0	ID=cds4160	0	5,5	STM14_4304	zntA	zinc%2Fcadmium%2Fmercury%2Flead-transporting ATPas	0,852600993	-1,98056697
NC_01685(	RefSeq	CDS	3758540	3760183	+	0	ID=cds4161	0	36,5	STM14_4305	tcp	methyl-accepting transmembrane citrate%2Fphenol chem	5,115605959	-2,281753063
NC_01685(	RefSeq	CDS	3760207	3760452	-	0	ID=cds4162	1	8,5	STM14_4306	yhhP	cell developmental protein SirA	2,896123694	0,419298367
NC_01685(	RefSeq	CDS	3760622	3761287	+	0	ID=cds4163	0	0,5	STM14_4307	yhhQ	hypothetical protein	0	NA
NC_01685(	RefSeq	CDS	3761360	3761917	+	0	ID=cds4164	2	18,5	STM14_4308	STM14_4308	hypothetical protein	6,07644772	0,297448745
NC_01685(	RefSeq	CDS	3761921	3763138	-	0	ID=cds4165	13,83333333	12	STM14_4309	yhhS	hypothetical protein	26,33571517	2,565412158
NC_01685(	RefSeq	CDS	3763270	3764319	+	0	ID=cds4166	0	3,5	STM14_4310	yhhT	putative permease	0,568400662	-1,710438693
NC_01685(	RefSeq	CDS	3764371	3764949	+	0	ID=cds4167	0	6	STM14_4311	acpT	holo-%28acyl carrier protein%29 synthase 2	0,852600993	-1,98056697
NC_01685(	RefSeq	CDS	3765041	3765442	+	0	ID=cds4168	0	9	STM14_4312	nikR	nickel responsive regulator	1,27890149	-2,207741391
NC_01685(	RefSeq	CDS	3765532	3766656	-	0	ID=cds4169	0	11,25	STM14_4313	yhhJ	putative ABC transport protein	1,563101821	-2,297202414
NC_01685(	RefSeq	CDS	3766656	3769397	-	0	ID=cds4170	0	24,25	STM14_4314	yhiH	putative ABC-type multidrug transport system ATPase cor	3,410403973	-2,434086198
NC_01685(	RefSeq	CDS	3769394	3770461	-	0	ID=cds4171	0	16	STM14_4315	yhiI	hypothetical protein	2,273602649	-2,403729808
NC_01685(	RefSeq	CDS	3770553	3770684	+	0	ID=cds4172	0	0	STM14_4316	STM14_4316	hypothetical protein	0	NA
NC_01685(	RefSeq	CDS	3770767	3771963	-	0	ID=cds4173	0	3,5	STM14_4317	yhiN	hypothetical protein	0,568400662	-1,710438693
NC_01685(	RefSeq	CDS	3772194	3773690	+	0	ID=cds4174	0	42,33333333	STM14_4318	pitA	low-affinity phosphate transporter	5,968206953	-2,232890319
NC_01685(	RefSeq	CDS	3773830	3774165	-	0	ID=cds4175	2	1	STM14_4319	uspB	universal stress protein UspB	3,660744905	2,693976715
NC_01685(	RefSeq	CDS	3774553	3774987	+	0	ID=cds4176	0	69,5	STM14_4320	uspA	universal stress protein A	9,947011588	-2,04667217
NC_01685(	RefSeq	CDS	3775312	3776784	+	0	ID=cds4177	0	149	STM14_4321	yhiP	inner membrane transporter YhiP	21,17292466	-1,8753915
NC_01685(	RefSeq	CDS	3776876	3777634	-	0	ID=cds4178	0	5,75	STM14_4322	yhiQ	putative methyltransferase	0,852600993	-1,98056697
NC_01685(	RefSeq	CDS	3777641	3779683	-	0	ID=cds4179	0	26,75	STM14_4323	prlC	oligopeptidase A	3,836704469	-2,44262576
NC_01685(	RefSeq	CDS	3779865	3781136	-	0	ID=cds4180	0	3	STM14_4324	STM14_4324	putative phosphatase	0,426300497	-1,498211557
NC_01685(	RefSeq	CDS	3781138	3781305	+	0	ID=cds4181	0	0	STM14_4325	STM14_4325	hypothetical protein	0	NA
NC_01685(	RefSeq	CDS	3781347	3782189	+	0	ID=cds4182	0	27,5	STM14_4326	yhiR	putative cytoplasmic protein	3,978804635	-2,448045729
NC_01685(	RefSeq	CDS	3782294	3783646	+	0	ID=cds4183	0	39,5	STM14_4327	gor	glutathione reductase	5,684006621	-2,24950002
NC_01685(	RefSeq	CDS	3783693	3784736	-	0	ID=cds4184	0	22,5	STM14_4328	STM14_4328	putative L-asparaginase	3,126203642	-2,431590384
NC_01685(	RefSeq	CDS	3784795	3786114	-	0	ID=cds4185	0	69	STM14_4329	STM14_4329	anaerobic C4-dicarboxylate transporter	9,804911422	-2,052421118
NC_01685(	RefSeq	CDS	3786168	3787013	-	0	ID=cds4186	0	60	STM14_4330	STM14_4330	putative sugar kinase	8,526009932	-2,104284262
NC_01685(	RefSeq	CDS	3787077	3788054	-	0	ID=cds4187	0	133,3333333	STM14_4331	STM14_4331	putative phosphosugar isomerase	18,89932202	-1,8640798
NC_01685(	RefSeq	CDS	3788230	3788949	-	0	ID=cds4188	0	7	STM14_4332	STM14_4332	putative regulatory protein	0,994701159	-2,072978628
NC_01685(	RefSeq	CDS	3789092	3789217	+	0	ID=cds4189	0	1	STM14_4333	STM14_4333	hypothetical protein	0,142100166	-0,623264422
NC_01685(	RefSeq	CDS	3789275	3790924	+	0	ID=cds4190	0	10,5	STM14_4334	treF	trehalase	1,421001655	-2,256999172
NC_01685(	RefSeq	CDS	3790937	3792526	-	0	ID=cds4191	0	23,5	STM14_4335	STM14_4335	putative inner membrane protein	3,410403973	-2,434086198
NC_01685(	RefSeq	CDS	3792675	3792815	-	0	ID=cds4192	0	0	STM14_4336	STM14_4336	hypothetical protein	0	NA
NC_01685(	RefSeq	CDS	3792807	3793163	+	0	ID=cds4193	0	0	STM14_4337	STM14_4337	putative phage endolysin	0	NA
NC_01685(	RefSeq	CDS	3793169	3793771	-	0	ID=cds4194	0	0	STM14_4338	yhjB	putative transcriptional regulator	0	NA
NC_01685(	RefSeq	CDS	3794036	3794179	-	0	ID=cds4195	0	0	STM14_4339	STM14_4339	hypothetical protein	0	NA
NC_01685(	RefSeq	CDS	3794140	3794265	+	0	ID=cds4196	0	0	STM14_4340	STM14_4340	hypothetical protein	0	NA
NC_01685(	RefSeq	CDS	3794402	3795301	+	0	ID=cds4197	0	1,5	STM14_4341	yhjC	putative transcriptional regulator	0,284200331	-1,182723423
NC_01685(	RefSeq	CDS	3795374	3796402	+	0	ID=cds4198	0	2	STM14_4342	yhjD	putative tRNA-processing ribonuclease	0,284200331	-1,182723423
NC_01685(	RefSeq	CDS	3796527	3796667	+	0	ID=cds4199	0	0	STM14_4343	STM14_4343	hypothetical protein	0	NA
NC_01685(	RefSeq	CDS	3796753	3798075	+	0	ID=cds4200	0	1,5	STM14_4344	yhjE	putative transport protein	0,284200331	-1,182723423
NC_01685(	RefSeq	CDS	3798115	3800175	-	0	ID=cds4201	0	9	STM14_4345	yhjG	putative inner membrane protein	1,27890149	-2,207741391
NC_01685(	RefSeq	CDS	3800285	3801052	-	0	ID=cds4202	0	9	STM14_4346	yhjH	EAL domain-containing protein	1,27890149	-2,207741391
NC_01685(	RefSeq	CDS	3801111	3801227	-	0	ID=cds4203	0	0,5	STM14_4347	STM14_4347	hypothetical protein	0	NA
NC_01685(	RefSeq	CDS	3801281	3802210	+	0	ID=cds4204	0	1	STM14_4348	kdgK	ketodeoxygluconokinase	0,142100166	-0,623264422
NC_01685(	RefSeq	CDS	3802264	3803751	-	0	ID=cds4205	0	54,75	STM14_4349	yhjJ	putative Zn-dependent peptidase	7,815509104	-2,137668811
NC_01685(	RefSeq	CDS	3803739	3803876	+	0	ID=cds4206	0	0	STM14_4350	STM14_4350	hypothetical protein	0	NA
NC_01685(	RefSeq	CDS	3803971	3805257	-	0	ID=cds4207	0	84,5	STM14_4351	dctA	C4-dicarboxylate transporter DctA	11,93641391	-1,973338016
NC_01685(	RefSeq	CDS	3805315	3805461	-	0	ID=cds4208	0	1	STM14_4352	STM14_4352	hypothetical protein	0,142100166	-0,623264422
NC_01685(	RefSeq	CDS	3805415	3807388	-	0	ID=cds4209	0	11,5	STM14_4353	yhjK	putative phosphodiesterase	1,705201986	-2,329925387
NC_01685(	RefSeq	CDS	3807400	3807591	-	0	ID=cds4210	0	0	STM14_4354	STM14_4354	hypothetical protein	0	NA
NC_01685(	RefSeq	CDS	3807595	3811137	-	0	ID=cds4211	0	25	STM14_4355	yhjL	cellulose synthase subunit BcsC	3,552504138	-2,43600272
NC_01685(	RefSeq	CDS	3811119	3812228	-	0	ID=cds4212	0	11,5	STM14_4356	STM14_4356	endo-1%2C4-D-glucanase	1,705201986	-2,329925387
NC_01685(	RefSeq	CDS	3812232	3814532	-	0	ID=cds4213	0	24	STM14_4357	yhjN	cellulose synthase regulator protein	3,410403973	-2,434086198
NC_01685(	RefSeq	CDS	3814543	3817167	-	0	ID=cds4214	0	30	STM14_4358	bcsA	cellulose synthase catalytic subunit	4,263004966	-2,464060063
NC_01685(	RefSeq	CDS	3817164	3817916	-	0	ID=cds4215	0	12	STM14_4359	yhjQ	cell division protein	1,705201986	-2,329925387
NC_01685(	RefSeq	CDS	3817917	3818120	-	0	ID=cds4216	0	3,5	STM14_4360	yhjR	putative cytoplasmic protein	0,568400662	-1,710438693
NC_01685(	RefSeq	CDS	3818231	3818344	+	0	ID=cds4217	0	0	STM14_4361	STM14_4361	hypothetical protein	0	NA
NC_01685(	RefSeq	CDS	3818365	3819936	+	0	ID=cds4218	0	8,5	STM14_4362	yhjS	putative cytoplasmic protein	1,136801324	-2,147403465
NC_01685(	RefSeq	CDS	3819933	3820124	+	0	ID=cds4219	0	4,5	STM14_4363	yhjT	putative inner membrane protein	0,568400662	-1,710438693
NC_01685(	RefSeq	CDS	3820121	3821800	+	0	ID=cds4220	0	15,5	STM14_4364	yhjU	putative inner membrane protein	2,273602649	-2,403729808
NC_01685(	RefSeq	CDS	3822632	3823930	+	0	ID=cds4221	0	3,5	STM14_4369	yhjV	putative transport protein	0,568400662	-1,710438693
NC_01685(	RefSeq	CDS	3824031	3825044	-	0	ID=cds4222	0	5,5	STM14_4370	dppF	dipeptide transporter ATP-binding subunit	0,852600993	-1,98056697
NC_01685(	RefSeq	CDS	3825041	3826024	-	0	ID=cds4223	0	2,5	STM14_4371	dppD	dipeptide transporter ATP-binding subunit	0,284200331	-1,182723423
NC_01685(	RefSeq	CDS	3826035	3826937	-	0	ID=cds4224	0	6,5	STM14_4372	dppC	dipeptide transporter	0,852600993	-1,98056697
NC_01685(	RefSeq	CDS	3826947	3827966	-	0	ID=cds4225	0	2	STM14_4373	dppB	dipeptide transporter permease DppB	0,284200331	-1,182723423
NC_01685(	RefSeq	CDS	3827982	3828101	-	0	ID=cds4226	0	1,5	STM14_4374	STM14_4374	hypothetical protein	0,284200331	-1,182723423
NC_01685(	RefSeq	CDS	3828123	3829730	-	0	ID=cds4227	0	21	STM14_4375	dppA	dipeptide transport protein	2,984103476	-2,430148754

NC_01685(	RefSeq	CDS	3830286	3831611	-	0	ID=cds4228	0	0	STM14_4376	STM14_4376	putative xanthine permease	0	NA
NC_01685(	RefSeq	CDS	3831669	3832793	-	0	ID=cds4229	0	0,25	STM14_4377	STM14_4377	hypothetical protein	0	NA
NC_01685(	RefSeq	CDS	3832781	3832954	-	0	ID=cds4230	0	0,25	STM14_4378	STM14_4378	hypothetical protein	0	NA
NC_01685(	RefSeq	CDS	3832815	3832976	+	0	ID=cds4231	0	0	STM14_4379	STM14_4379	hypothetical protein	0	NA
NC_01685(	RefSeq	CDS	3832943	3833950	+	0	ID=cds4232	0	1	STM14_4380	STM14_4380	putative regulatory protein	0,142100166	-0,623264422
NC_01685(	RefSeq	CDS	3834277	3835968	-	0	ID=cds4233	0	42	STM14_4382	yhjW	phosphoethanolamine transferase	5,968206953	-2,232890319
NC_01685(	RefSeq	CDS	3836186	3836713	-	0	ID=cds4234	0	1	STM14_4383	lpfE	long polar fimbrial minor protein	0,142100166	-0,623264422
NC_01685(	RefSeq	CDS	3837806	3840334	-	0	ID=cds4235	0	1	STM14_4385	lpfC	long polar fimbrial outer membrane usher protein	0,142100166	-0,623264422
NC_01685(	RefSeq	CDS	3840357	3841055	-	0	ID=cds4236	0	0	STM14_4386	lpfB	long polar fimbrial chaperone precursor	0	NA
NC_01685(	RefSeq	CDS	3841140	3841676	-	0	ID=cds4237	0	0	STM14_4387	lpfA	long polar fimbrial protein A precursor	0	NA
NC_01685(	RefSeq	CDS	3842019	3842180	+	0	ID=cds4238	0	0	STM14_4388	STM14_4388	hypothetical protein	0	NA
NC_01685(	RefSeq	CDS	3842182	3842886	-	0	ID=cds4239	0	0	STM14_4389	yhjY	putative lipase	0	NA
NC_01685(	RefSeq	CDS	3843043	3843624	+	0	ID=cds4240	0	5,5	STM14_4390	tag	3-methyladenine DNA glycosylase	0,852600993	-1,98056697
NC_01685(	RefSeq	CDS	3843602	3844042	+	0	ID=cds4241	0	6	STM14_4391	yiaC	hypothetical protein	0,852600993	-1,98056697
NC_01685(	RefSeq	CDS	3844011	3846344	-	0	ID=cds4242	0	16	STM14_4392	bisC	biotin sulfoxide reductase	2,273602649	-2,403729808
NC_01685(	RefSeq	CDS	3846344	3846445	+	0	ID=cds4243	0	0	STM14_4393	STM14_4393	hypothetical protein	0	NA
NC_01685(	RefSeq	CDS	3846497	3847159	+	0	ID=cds4244	0	13	STM14_4394	yiaD	putative outer membrane lipoprotein	1,847302152	-2,356210635
NC_01685(	RefSeq	CDS	3847245	3847379	-	0	ID=cds4245	0	0,5	STM14_4395	STM14_4395	hypothetical protein	0	NA
NC_01685(	RefSeq	CDS	3847378	3848352	+	0	ID=cds4246	0	12	STM14_4396	yiaE	2-hydroxyacid dehydrogenase	1,705201986	-2,329925387
NC_01685(	RefSeq	CDS	3848402	3849112	-	0	ID=cds4247	0	16	STM14_4397	yiaF	putative outer membrane lipoprotein	2,273602649	-2,403729808
NC_01685(	RefSeq	CDS	3849551	3849841	+	0	ID=cds4248	0	1	STM14_4398	yiaG	putative transcriptional regulator	0,142100166	-0,623264422
NC_01685(	RefSeq	CDS	3850130	3850342	+	0	ID=cds4249	2	958,5	STM14_4399	cspA	major cold shock protein	139,6506033	-3,40989097
NC_01685(	RefSeq	CDS	3850516	3851055	+	0	ID=cds4250	0	6,5	STM14_4400	STM14_4400	hypothetical protein	0,852600993	-1,98056697
NC_01685(	RefSeq	CDS	3851427	3851912	-	0	ID=cds4251	0	4,5	STM14_4401	STM14_4401	putative acetyltransferase	0,568400662	-1,710438693
NC_01685(	RefSeq	CDS	3851900	3852187	-	0	ID=cds4252	0	3,5	STM14_4402	STM14_4402	putative cytoplasmic protein	0,568400662	-1,710438693
NC_01685(	RefSeq	CDS	3852193	3852324	-	0	ID=cds4253	0	0	STM14_4403	STM14_4403	hypothetical protein	0	NA
NC_01685(	RefSeq	CDS	3852365	3852832	-	0	ID=cds4254	0	0	STM14_4404	STM14_4404	putative acetyltransferase	0	NA
NC_01685(	RefSeq	CDS	3853461	3855530	-	0	ID=cds4255	0	34,5	STM14_4406	glyS	glycyl-tRNA synthetase subunit beta	4,831405628	-2,523251848
NC_01685(	RefSeq	CDS	3855540	3856451	-	0	ID=cds4256	0	31,25	STM14_4407	glyQ	glycyl-tRNA synthetase subunit alpha	4,405105132	-2,475774913
NC_01685(	RefSeq	CDS	3856473	3856610	+	0	ID=cds4257	0	0	STM14_4408	STM14_4408	hypothetical protein	0	NA
NC_01685(	RefSeq	CDS	3856590	3856892	-	0	ID=cds4258	0	0,5	STM14_4409	STM14_4409	putative outer membrane lipoprotein	0	NA
NC_01685(	RefSeq	CDS	3856798	3857019	-	0	ID=cds4259	0	0,5	STM14_4410	STM14_4410	hypothetical protein	0	NA
NC_01685(	RefSeq	CDS	3857058	3858053	+	0	ID=cds4260	0	0	STM14_4411	yiaH	putative inner membrane protein	0	NA
NC_01685(	RefSeq	CDS	3858076	3858429	-	0	ID=cds4261	0	1	STM14_4412	yiaB	putative inner membrane protein	0,142100166	-0,623264422
NC_01685(	RefSeq	CDS	3858604	3860058	-	0	ID=cds4262	0	2	STM14_4413	xylB	xylulokinase	0,284200331	-1,182723423
NC_01685(	RefSeq	CDS	3860158	3861480	-	0	ID=cds4263	0	9	STM14_4414	xylA	xylose isomerase	1,27890149	-2,207741391
NC_01685(	RefSeq	CDS	3861460	3861693	+	0	ID=cds4264	0	0	STM14_4415	STM14_4415	hypothetical protein	0	NA
NC_01685(	RefSeq	CDS	3861562	3861738	-	0	ID=cds4265	0	0	STM14_4416	STM14_4416	hypothetical protein	0	NA
NC_01685(	RefSeq	CDS	3861843	3863021	+	0	ID=cds4266	0	1	STM14_4417	xylR	xylose operon regulatory protein	0,142100166	-0,623264422
NC_01685(	RefSeq	CDS	3863082	3863906	-	0	ID=cds4267	1	75	STM14_4418	bax	hypothetical protein	12,41683479	-1,676999795
NC_01685(	RefSeq	CDS	3863890	3864222	+	0	ID=cds4268	0	0	STM14_4419	STM14_4419	hypothetical protein	0	NA
NC_01685(	RefSeq	CDS	3864222	3866249	+	0	ID=cds4269	0	1	STM14_4420	malS	periplasmic alpha-amylase	0,142100166	-0,623264422
NC_01685(	RefSeq	CDS	3866423	3867673	+	0	ID=cds4270	0	7,5	STM14_4421	avtA	valine--pyruvate transaminase	1,136801324	-2,147403465
NC_01685(	RefSeq	CDS	3867710	3868183	-	0	ID=cds4271	0	9,5	STM14_4422	ysaA	putative oxidoreductase	1,421001655	-2,256999172
NC_01685(	RefSeq	CDS	3868300	3869100	-	0	ID=cds4272	0	7,5	STM14_4423	yiaJ	transcriptional repressor	1,136801324	-2,147403465
NC_01685(	RefSeq	CDS	3869165	3869311	-	0	ID=cds4273	0	0	STM14_4424	STM14_4424	hypothetical protein	0	NA
NC_01685(	RefSeq	CDS	3869330	3870328	+	0	ID=cds4274	0	1	STM14_4425	yiaK	2%2C3-diketo-L-gulonate reductase	0,142100166	-0,623264422
NC_01685(	RefSeq	CDS	3870340	3870804	+	0	ID=cds4275	0	0	STM14_4426	yiaL	putative cytoplasmic protein	0	NA
NC_01685(	RefSeq	CDS	3870828	3871760	+	0	ID=cds4276	0	0	STM14_4427	STM14_4427	putative chemotaxis protein	0	NA
NC_01685(	RefSeq	CDS	3871899	3872372	+	0	ID=cds4277	0	0	STM14_4428	yiaM	2%2C3-diketo-L-gulonate TRAP transporter small permeas	0	NA
NC_01685(	RefSeq	CDS	3872375	3873652	+	0	ID=cds4278	0	1	STM14_4429	yiaN	hypothetical protein	0,142100166	-0,623264422
NC_01685(	RefSeq	CDS	3873664	3874650	+	0	ID=cds4279	0	0	STM14_4430	yiaO	putative periplasmic dicarboxylate-binding protein	0	NA
NC_01685(	RefSeq	CDS	3874654	3876150	+	0	ID=cds4280	0	0	STM14_4431	lyxK	L-xylulose kinase	0	NA
NC_01685(	RefSeq	CDS	3876147	3876809	+	0	ID=cds4281	0	0	STM14_4432	sgbH	3-keto-L-gulonate-6-phosphate decarboxylase	0	NA
NC_01685(	RefSeq	CDS	3876802	3877662	+	0	ID=cds4282	0	0	STM14_4433	sgbU	putative L-xylulose 5-phosphate 3-epimerase	0	NA
NC_01685(	RefSeq	CDS	3877656	3878351	+	0	ID=cds4283	0	0	STM14_4434	sgbE	L-ribulose-5-phosphate 4-epimerase	0	NA
NC_01685(	RefSeq	CDS	3878512	3879327	-	0	ID=cds4284	0	1	STM14_4435	STM14_4435	putative regulatory protein	0,142100166	-0,623264422
NC_01685(	RefSeq	CDS	3879591	3881546	+	0	ID=cds4285	0	4	STM14_4436	STM14_4436	putative cytoplasmic protein	0,568400662	-1,710438693
NC_01685(	RefSeq	CDS	3881551	3881694	+	0	ID=cds4286	0	0,75	STM14_4437	STM14_4437	hypothetical protein	0,142100166	-0,623264422
NC_01685(	RefSeq	CDS	3881664	3883202	-	0	ID=cds4287	0	13,5	STM14_4438	aldB	aldehyde dehydrogenase B	1,989402318	-2,376535557
NC_01685(	RefSeq	CDS	3883371	3884252	+	0	ID=cds4288	0	3	STM14_4439	STM14_4439	putative transcriptional regulator	0,426300497	-1,498211557
NC_01685(	RefSeq	CDS	3884537	3886387	-	0	ID=cds4289	0	22,5	STM14_4440	selB	selenocysteinyl-tRNA-specific translation factor	3,126203642	-2,431590384
NC_01685(	RefSeq	CDS	3886384	3887775	-	0	ID=cds4290	0	15,5	STM14_4441	selA	selenocysteine synthase	2,273602649	-2,403729808
NC_01685(	RefSeq	CDS	3887874	3888482	-	0	ID=cds4291	2	3	STM14_4442	yibF	glutathione S-transferase	3,944945236	1,874572685
NC_01685(	RefSeq	CDS	3888958	3890874	+	0	ID=cds4292	1	19	STM14_4443	mtlA	mannitol-specific enzyme IIABC component	4,459225515	-0,383770206
NC_01685(	RefSeq	CDS	3891080	3892243	+	0	ID=cds4293	0	15,5	STM14_4444	mtlD	mannitol-1-phosphate 5-dehydrogenase	2,273602649	-2,403729808
NC_01685(	RefSeq	CDS	3892240	3892830	+	0	ID=cds4294	0	19	STM14_4445	mtlR	mannitol repressor protein	2,699903145	-2,42419778
NC_01685(	RefSeq	CDS	3892840	3893049	-	0	ID=cds4295	0	1	STM14_4446	STM14_4446	putative cytoplasmic protein	0,142100166	-0,623264422
NC_01685(	RefSeq	CDS	3893340	3893702	+	0	ID=cds4296	0	1,5	STM14_4447	yibL	hypothetical protein	0,284200331	-1,182723423
NC_01685(	RefSeq	CDS	3893735	3893932	+	0	ID=cds4297	0	0,5	STM14_4448	STM14_4448	hypothetical protein	0	NA
NC_01685(	RefSeq	CDS	3894193	3894876	+	0	ID=cds4298	0	0	STM14_4449	STM14_4449	putative inner membrane lipoprotein	0	NA
NC_01685(	RefSeq	CDS	3894920	3899305	+	0	ID=cds4299	0	3,5	STM14_4450	STM14_4450	putative inner membrane protein	0,568400662	-1,710438693
NC_01685(	RefSeq	CDS	3899604	3901259	+	0	ID=cds4300	0	0	STM14_4451	lldP	L-lactate permease	0	NA
NC_01685(	RefSeq	CDS	3901256	3902032	+	0	ID=cds4301	0	0	STM14_4452	lldR	DNA-binding transcriptional repressor LldR	0	NA
NC_01685(	RefSeq	CDS	3902029	3903219	+	0	ID=cds4302	0	3	STM14_4453	lldD	L-lactate dehydrogenase	0,426300497	-1,498211557
NC_01685(	RefSeq	CDS	3903283	3903756	+	0	ID=cds4303	0	2	STM14_4454	yibK	putative tRNA%2FrRNA methyltransferase YibK	0,284200331	-1,182723423
NC_01685(	RefSeq	CDS	3903824	3904828	-	0	ID=cds4304	0	4,5	STM14_4455	STM14_4455	putative transcriptional regulator	0,568400662	-1,710438693
NC_01685(	RefSeq	CDS	3904878	3905129	+	0	ID=cds4305	0	0	STM14_4456	STM14_4456	hypothetical protein	0	NA
NC_01685(	RefSeq	CDS	3905147	3906343	+	0	ID=cds4306	0	1	STM14_4457	STM14_4457	putative mandelate racemase	0,142100166	-0,623264422
NC_01685(	RefSeq	CDS	3906417	3907727	+	0	ID=cds4307	0	1	STM14_4458	STM14_4458	putative permease	0,142100166	-0,623264422
NC_01685(	RefSeq	CDS	3907810	3908631	-	0	ID=cds4308	0	26	STM14_4459	cysE	serine acetyltransferase	3,694604304	-2,438835558
NC_01685(	RefSeq	CDS	3908709	3909728	-	0	ID=cds4309	0	78	STM14_4460	gpsA	NAD%28P%29H-dependent glycerol-3-phosphate dehydro	11,08381291	-2,003216272
NC_01685(	RefSeq	CDS	3909728	3910195	-	0	ID=cds4310	0	46,5	STM14_4461	secB	preprotein translocase subunit SecB	6,536607615	-2,201017198
NC_01685(	RefSeq	CDS	3910239	3910490	-	0	ID=cds4311	0	21,5	STM14_4462	grxC	glutaredoxin 3	3,126203642	-2,431590384
NC_01685(	RefSeq	CDS	3910577	3911008	-	0	ID=cds4312	0	16,5	STM14_4463	yibN	putative rhodanese-like sulfurtransferase	2,273602649	-2,403729808
NC_01685(	RefSeq	CDS	3911256	3912800	+	0	ID=cds4313	0	43,5	STM14_4464	pmgI	phosphoglyceromutase	6,252407284	-2,216733793
NC_01685(	RefSeq	CDS	3912810	3914093	+	0	ID=cds4314	2	14,5	STM14_4465	yibP	hypothetical protein	5,508047057	0,527345695
NC_01685(	RefSeq	CDS	3914097	3915059	+	0	ID=cds4315	0	8,5	STM14_4466	yigQ	hypothetical protein	1,136801324	-2,147403465
NC_01685(	RefSeq	CDS	3915046	3916080	-	0	ID=cds4316	0	8,5	STM14_4467	yibD	glycosyl transferase family protein	1,136801324	-2,147403465
NC_01685(	RefSeq	CDS	3916321	3916461	-	0	ID=cds4317	0	4	STM14_4468	STM14_4468	putative inner membrane protein	0,568400662	-1,710438693
NC_01685(	RefSeq	CDS	3916574	3917599	-	0	ID=cds4318	0	78	STM14_4469	tdh	L-threonine 3-dehydrogenase	11,08381291	-2,003216272
NC_01685(	RefSeq	CDS	3917609	3918805	-	0	ID=cds4319	0	58,5	STM14_4470	kbl	2-amino-3-ketobutyrate coenzyme A ligase	8,241809601	-2,117369507
NC_01685(	RefSeq	CDS	3919008	3919940	+	0	ID=cds4320	0	75	STM14_4471	rfaD	ADP-L-glycero-D-manno-heptose-6-epimerase	10,65751242	-2,019002164
NC_01685(	RefSeq	CDS	3919943	3920989	+	0	ID=cds4321	0	39,25	STM14_4472	rfaF	ADP-heptose--LPS heptosyltransferase	5,541906456	-2,25579729
NC_01685(	RefSeq	CDS	3920989	3921942	+	0	ID=cds4322	0	20,25	STM14_4473	rfaC	ADP-heptose--LPS heptosyltransferase	2,842003311	-2,427810709
NC_01685(	RefSeq	CDS	3921982	3923196	+	0	ID=cds4323	0	25,5	STM14_4474	rfaL	O-antigen ligase	3,694604304	-2,438835558
NC_01685(	RefSeq	CDS	3923253	3924398	-	0	ID=cds4324	0	10	STM14_4475	rfaK	putative hexose transferase	1,421001655	-2,256999172
NC_01685(	RefSeq	CDS	3924499	3925308	-	0	ID=cds4325	0	20	STM14_4476	rfaZ	lipopolysaccharide core biosynthesis protein	2,842003311	-2,427810709
NC_01685(	RefSeq	CDS	3925458	3926156	-	0	ID=cds4326	0	20,5	STM14_4477	rfaY	lipopolysaccharide core biosynthesis protein	2,842003311	-2,427810709
NC_01685(	RefSeq	CDS	3926179	3927189	-	0	ID=cds4327	0	13,5	STM14_4478	rfaJ	lipopolysaccharide glucosyltransferase	1,989402318	-2,376535557
NC_01685(	RefSeq	CDS	3927207	3928220	-	0	ID=cds4328	0	8	STM14_4479	rfaI	lipopolysaccharide-alpha-1%2C 3-D-galactosyltransferase	1,136801324	-2,147403465
NC_01685(	RefSeq	CDS	3928226	3929305	-	0	ID=cds4329	0	12	STM14_4480	rfaB	UDP-D-galactose:%28glucosyl%29lipopolysaccharide-1%2(	1,705201986	-2,329925387
NC_01685(	RefSeq	CDS	3929375	3929608	-	0	ID=cds4330	0	5	STM14_4481	yibR	putative inner membrane protein	0,710500828	-1,864806942
NC_01685(	RefSeq	CDS	3929640	3930437	-	0	ID=cds4331	0	16	STM14_4482	rfaP	lipopolysaccharide core biosynthetic protein	2,273602649	-2,403729808
NC_01685(	RefSeq	CDS	3930430	3931554	-	0	ID=cds4332	0	34,5	STM14_4483	rfaG	glucosyltransferase I	4,831405628	-2,523251848
NC_01685(	RefSeq	CDS	3931551	3932585	-	0	ID=cds4333	0	21	STM14_4484	rfaQ	lipopolysaccharide core biosynthesis protein	2,984103476	-2,430148754
NC_01685(	RefSeq	CDS	3932731	3932850	-	0	ID=cds4334	0	0	STM14_4485	STM14_4485	hypothetical protein	0	NA
NC_01685(	RefSeq	CDS	3933030	3934307	+	0	ID=cds4335	1	38,75	STM14_4486	kdtA	3-deoxy-D-manno-octulosonic-acid transferase	7,301228826	-1,040766944
NC_01685(	RefSeq	CDS	3934316	3934795	+	0	ID=cds4336	0	12,25	STM14_4487	coaD	phosphopantetheine adenylyltransferase	1,705201986	-2,329925387
NC_01685(	RefSeq	CDS	3934823	3935632	-	0	ID=cds4337	0	4,5	STM14_4488	mutM	formamidopyrimidine-DNA glycosylase	0,568400662	-1,710438693
NC_01685(	RefSeq	CDS	3935730	3935897	-	0	ID=cds4338	0	11,5	STM14_4489	rpmG	50S ribosomal protein L33	1,705201986	-2,329925387
NC_01685(	RefSeq	CDS	3935918	3936154	-	0	ID=cds4339	0	53,5	STM14_4490	rpmB	50S ribosomal protein L28	7,673408939	-2,144619715
NC_01685(	RefSeq	CDS	3936372	3937211	-	0	ID=cds4340	0	8	STM14_4491	radC	DNA repair protein RadC	1,136801324	-2,147403465
NC_01685(	RefSeq	CDS	3937213	3938433	+	0	ID=cds4341	0	27,75	STM14_4492	dfp	bifunctional phosphopantothenoylcysteine decarboxylase	3,978804635	-2,448045729
NC_01685(	RefSeq	CDS	3938414	3938869	+	0	ID=cds4342	1	25,25	STM14_4493	dut	deoxyuridine 5%27-triphosphate nucleotidohydrolase	5,311826508	-0,631843652
NC_01685(	RefSeq	CDS	3938977	3939573	+	0	ID=cds4343	0	9,5	STM14_4494	slmA	nucleoid occlusion protein	1,421001655	-2,256999172
NC_01685(	RefSeq	CDS	3939651	3940292	-	0	ID=cds4344	0	3,5	STM14_4495	pyrE	orotate phosphoribosyltransferase	0,568400662	-1,710438693
NC_01685(	RefSeq	CDS	3940371	3941087	-	0	ID=cds4345	0	9,25	STM14_4496	rph	ribonuclease PH	1,27890149	-2,207741391
NC_01685(	RefSeq	CDS	3941019	3941198	+	0	ID=cds4346	0	0	STM14_4497	STM14_4497	hypothetical protein	0	NA
NC_01685(	RefSeq	CDS	3941213	3942076	+	0	ID=cds4347	0	15,5	STM14_4498	yicC	hypothetical protein	2,273602649	-2,403729808
NC_01685(	RefSeq	CDS	3942126	3943028	-	0	ID=cds4348	0	1	STM14_4499	STM14_4499	putative transcriptional regulator	0,142100166	-0,623264422
NC_01685(	RefSeq	CDS	3943102	3943983	+	0	ID=cds4349	0	0	STM14_4500	STM14_4500	putative Zn-dependent hydrolase	0	NA
NC_01685(	RefSeq	CDS	3943928	3944110	+	0	ID=cds4350	9	0	STM14_4501	STM14_4501	hypothetical protein	15,83390133	1,972603419
NC_01685(	RefSeq	CDS	3944234	3944851	+	0	ID=cds4351	0	1	STM14_4502	STM14_4502	putative inner membrane protein	0,142100166	-0,623264422

NC_016856	RefSeq	CDS	3944848	3946533	-	0	ID=cds4352	0	3	STM14_4503	ligB	NAD-dependent DNA ligase LigB	0,426300497	-1,498211557
NC_016856	RefSeq	CDS	3946552	3946725	+	0	ID=cds4353	0	0	STM14_4504	STM14_4504	hypothetical protein	0	NA
NC_016856	RefSeq	CDS	3946790	3947413	+	0	ID=cds4354	0	31	STM14_4505	gmk	guanylate kinase	4,405105132	-2,475774913
NC_016856	RefSeq	CDS	3947468	3947743	+	0	ID=cds4355	0	19	STM14_4506	rpoZ	DNA-directed RNA polymerase subunit omega	2,699903145	-2,42419778
NC_016856	RefSeq	CDS	3947762	3949873	+	0	ID=cds4356	0	83,5	STM14_4507	spoT	bifunctional %28p%29ppGpp synthetase II%2F guanosine-	11,93641391	-1,973338016
NC_016856	RefSeq	CDS	3949878	3950567	+	0	ID=cds4357	0	17,5	STM14_4508	spoU	tRNA guanosine-2%27-O-methyltransferase	2,55780298	-2,419166771
NC_016856	RefSeq	CDS	3950573	3952654	+	0	ID=cds4358	0	27,5	STM14_4509	recG	ATP-dependent DNA helicase RecG	3,978804635	-2,448045729
NC_016856	RefSeq	CDS	3952657	3953508	-	0	ID=cds4359	0	16,75	STM14_4510	STM14_4510	putative cytoplasmic protein	2,415702814	-2,412509737
NC_016856	RefSeq	CDS	3953511	3954716	-	0	ID=cds4360	0	30,75	STM14_4511	gltS	glutamate transport protein	4,405105132	-2,475774913
NC_016856	RefSeq	CDS	3954806	3954916	-	0	ID=cds4361	0	1,5	STM14_4512	STM14_4512	hypothetical protein	0,284200331	-1,182723423
NC_016856	RefSeq	CDS	3954944	3956335	+	0	ID=cds4362	1	6,5	STM14_4513	yicE	putative purine%2Fxanthine transport protein	2,611923363	0,709005733
NC_016856	RefSeq	CDS	3956456	3958165	+	0	ID=cds4363	1	8,5	STM14_4514	yicH	putative inner membrane protein	2,896123694	0,419298367
NC_016856	RefSeq	CDS	3958268	3960586	-	0	ID=cds4364	0	2,5	STM14_4515	yicI	alpha-xylosidase	0,284200331	-1,182723423
NC_016856	RefSeq	CDS	3960598	3961980	-	0	ID=cds4365	0	2	STM14_4516	yicJ	putative transporter	0,284200331	-1,182723423
NC_016856	RefSeq	CDS	3962472	3962597	+	0	ID=cds4366	0	0	STM14_4518	STM14_4518	hypothetical protein	0	NA
NC_016856	RefSeq	CDS	3962679	3963023	-	0	ID=cds4367	0	0	STM14_4519	STM14_4519	putative cytoplasmic protein	0	NA
NC_016856	RefSeq	CDS	3963213	3963356	+	0	ID=cds4368	0	0	STM14_4520	STM14_4520	hypothetical protein	0	NA
NC_016856	RefSeq	CDS	3963387	3963497	-	0	ID=cds4369	0	0	STM14_4521	STM14_4521	hypothetical protein	0	NA
NC_016856	RefSeq	CDS	3963701	3964891	+	0	ID=cds4370	0	7,5	STM14_4522	sugR	ATP-binding protein	1,136801324	-2,147403465
NC_016856	RefSeq	CDS	3964888	3965238	+	0	ID=cds4371	0	2	STM14_4523	STM14_4523	putative cytoplasmic protein	0,284200331	-1,182723423
NC_016856	RefSeq	CDS	3965425	3966462	+	0	ID=cds4372	0	7	STM14_4524	rhuM	putative cytoplasmic protein	0,994701159	-2,072978628
NC_016856	RefSeq	CDS	3967761	3968378	+	0	ID=cds4373	0	0,5	STM14_4526	rmbA	putative cytoplasmic protein	0	NA
NC_016856	RefSeq	CDS	3968453	3971320	+	0	ID=cds4374	0	3	STM14_4527	misL	putative autotransporter	0,426300497	-1,498211557
NC_016856	RefSeq	CDS	3971415	3971879	-	0	ID=cds4375	0	1	STM14_4528	fidL	putative inner membrane protein	0,142100166	-0,623264422
NC_016856	RefSeq	CDS	3971890	3972678	-	0	ID=cds4376	0	1	STM14_4529	marT	putative transcriptional regulator	0,142100166	-0,623264422
NC_016856	RefSeq	CDS	3973351	3973476	+	0	ID=cds4377	0	0	STM14_4531	STM14_4531	hypothetical protein	0	NA
NC_016856	RefSeq	CDS	3973531	3974211	+	0	ID=cds4378	0	6	STM14_4532	slsA	putative inner membrane protein	0,852600993	-1,98056697
NC_016856	RefSeq	CDS	3974252	3974359	-	0	ID=cds4379	0	0,285714286	STM14_4533	STM14_4533	hypothetical protein	0	NA
NC_016856	RefSeq	CDS	3974440	3974919	-	0	ID=cds4380	0	4,5	STM14_4534	cigR	putative inner membrane protein	0,568400662	-1,710438693
NC_016856	RefSeq	CDS	3974935	3975075	+	0	ID=cds4381	0	0	STM14_4535	STM14_4535	hypothetical protein	0	NA
NC_016856	RefSeq	CDS	3975234	3977960	-	0	ID=cds4382	25	6,5	STM14_4536	mgtB	Mg2%2B transporter	44,83566024	3,597771194
NC_016856	RefSeq	CDS	3978059	3978172	+	0	ID=cds4383	0	0	STM14_4537	STM14_4537	hypothetical protein	0	NA
NC_016856	RefSeq	CDS	3978180	3978875	-	0	ID=cds4384	0	7	STM14_4538	mgtC	Mg2%2B transport protein	0,994701159	-2,072978628
NC_016856	RefSeq	CDS	3979055	3979171	+	0	ID=cds4385	0	0	STM14_4539	STM14_4539	hypothetical protein	0	NA
NC_016856	RefSeq	CDS	3979172	3979297	-	0	ID=cds4386	0	0	STM14_4540	STM14_4540	hypothetical protein	0	NA
NC_016856	RefSeq	CDS	3979384	3980286	+	0	ID=cds4387	0	6,5	STM14_4541	yicL	putative permease	0,852600993	-1,98056697
NC_016856	RefSeq	CDS	3980329	3981270	-	0	ID=cds4388	0	1,5	STM14_4542	STM14_4542	putative cytoplasmic protein	0,284200331	-1,182723423
NC_016856	RefSeq	CDS	3981506	3982249	-	0	ID=cds4389	0	0,5	STM14_4543	STM14_4543	putative cytoplasmic protein	0	NA
NC_016856	RefSeq	CDS	3982236	3983345	-	0	ID=cds4390	0	2	STM14_4544	STM14_4544	putative selenocysteine synthase	0,284200331	-1,182723423
NC_016856	RefSeq	CDS	3983349	3984206	-	0	ID=cds4391	0	1	STM14_4545	STM14_4545	putative PTS system transporter subunit	0,142100166	-0,623264422
NC_016856	RefSeq	CDS	3984206	3984955	-	0	ID=cds4392	0	1	STM14_4546	STM14_4546	putative PTS system transporter subunitC	0,142100166	-0,623264422
NC_016856	RefSeq	CDS	3985101	3985586	-	0	ID=cds4393	0	0	STM14_4547	STM14_4547	putative PTS system transporter subunitB	0	NA
NC_016856	RefSeq	CDS	3985597	3986022	-	0	ID=cds4394	0	0	STM14_4548	STM14_4548	putative PTS system transporter subunitA	0	NA
NC_016856	RefSeq	CDS	3986024	3986146	+	0	ID=cds4395	0	0	STM14_4549	STM14_4549	hypothetical protein	0	NA
NC_016856	RefSeq	CDS	3986241	3989039	-	0	ID=cds4396	1	46,5	STM14_4550	STM14_4550	putative transcriptional regulator	8,295929985	-1,195723789
NC_016856	RefSeq	CDS	3989051	3989164	-	0	ID=cds4397	0	2	STM14_4551	STM14_4551	hypothetical protein	0,284200331	-1,182723423
NC_016856	RefSeq	CDS	3989236	3989529	+	0	ID=cds4398	0	0	STM14_4552	STM14_4552	putative inner membrane protein	0	NA
NC_016856	RefSeq	CDS	3989514	3989657	+	0	ID=cds4399	0	1,5	STM14_4553	STM14_4553	hypothetical protein	0,284200331	-1,182723423
NC_016856	RefSeq	CDS	3989635	3991017	+	0	ID=cds4400	0	11	STM14_4554	STM14_4554	putative glycosyl hydrolase	1,563101821	-2,297202414
NC_016856	RefSeq	CDS	3991077	3992270	-	0	ID=cds4401	0	2	STM14_4555	nepI	ribonucleoside transporter	0,284200331	-1,182723423
NC_016856	RefSeq	CDS	3992486	3992845	+	0	ID=cds4402	0	0,5	STM14_4556	STM14_4556	putative cytoplasmic protein	0	NA
NC_016856	RefSeq	CDS	3992829	3993152	+	0	ID=cds4403	0	1,5	STM14_4557	STM14_4557	putative DNA-binding protein	0,284200331	-1,182723423
NC_016856	RefSeq	CDS	3993243	3993512	-	0	ID=cds4404	0	3	STM14_4558	STM14_4558	putative phosphotransferase system HPr protein	0,426300497	-1,498211557
NC_016856	RefSeq	CDS	3993522	3994382	-	0	ID=cds4405	0	2	STM14_4559	gatY_2	putative fructose-1%2C6-bisphosphate aldolase	0,284200331	-1,182723423
NC_016856	RefSeq	CDS	3994445	3995929	-	0	ID=cds4406	0	4,5	STM14_4560	STM14_4560	putative sugar kinase	0,568400662	-1,710438693
NC_016856	RefSeq	CDS	3995922	3997280	-	0	ID=cds4407	0	13	STM14_4561	STM14_4561	putative PTS system galactitol-specific enzyme IIC compor	1,847302152	-2,356210635
NC_016856	RefSeq	CDS	3997356	3997643	-	0	ID=cds4408	0	4	STM14_4562	STM14_4562	hypothetical protein	0,568400662	-1,710438693
NC_016856	RefSeq	CDS	3997640	3998113	-	0	ID=cds4409	0	13	STM14_4563	STM14_4563	phosphotransferase system mannitol%2Ffructose-specific	1,847302152	-2,356210635
NC_016856	RefSeq	CDS	3998131	3998874	-	0	ID=cds4410	0	4,5	STM14_4564	STM14_4564	putative regulatory protein	0,568400662	-1,710438693
NC_016856	RefSeq	CDS	3999123	3999230	-	0	ID=cds4411	0	0,25	STM14_4565	STM14_4565	hypothetical protein	0	NA
NC_016856	RefSeq	CDS	3999227	3999679	-	0	ID=cds4412	0	3,25	STM14_4566	yicN	putative inner membrane protein	0,426300497	-1,498211557
NC_016856	RefSeq	CDS	3999764	3999871	-	0	ID=cds4413	0	0	STM14_4567	STM14_4567	hypothetical protein	0	NA
NC_016856	RefSeq	CDS	3999872	4001263	-	0	ID=cds4414	0	0	STM14_4568	uhpT	sugar phosphate antiporter	0	NA
NC_016856	RefSeq	CDS	4001406	4002734	-	0	ID=cds4415	0	2	STM14_4569	uhpC	regulatory protein UhpC	0,284200331	-1,182723423
NC_016856	RefSeq	CDS	4002744	4004246	-	0	ID=cds4416	0	1	STM14_4570	uhpB	sensory histidine kinase UhpB	0,142100166	-0,623264422
NC_016856	RefSeq	CDS	4004246	4004836	-	0	ID=cds4417	0	2	STM14_4571	uhpA	DNA-binding transcriptional activator UhpA	0,284200331	-1,182723423
NC_016856	RefSeq	CDS	4004911	4005924	-	0	ID=cds4418	0	9,5	STM14_4572	STM14_4572	putative cytoplasmic protein	1,421001655	-2,256999172
NC_016856	RefSeq	CDS	4005936	4007252	-	0	ID=cds4419	0	12	STM14_4573	STM14_4573	putative L-fucose permease	1,705201986	-2,329925387
NC_016856	RefSeq	CDS	4007283	4008203	-	0	ID=cds4420	0	9	STM14_4574	STM14_4574	putative sugar kinase	1,27890149	-2,207741391
NC_016856	RefSeq	CDS	4008528	4009313	+	0	ID=cds4421	0	2	STM14_4575	STM14_4575	putative regulatory protein	0,284200331	-1,182723423
NC_016856	RefSeq	CDS	4009315	4009605	-	0	ID=cds4422	0	0	STM14_4576	ilvN	acetolactate synthase 1 regulatory subunit	0	NA
NC_016856	RefSeq	CDS	4009609	4011297	-	0	ID=cds4423	0	6,5	STM14_4577	ilvB	acetolactate synthase catalytic subunit	0,852600993	-1,98056697
NC_016856	RefSeq	CDS	4011296	4011472	+	0	ID=cds4424	0	0	STM14_4578	STM14_4578	hypothetical protein	0	NA
NC_016856	RefSeq	CDS	4011404	4011502	-	0	ID=cds4425	0	22,75	STM14_4579	ivbL	ilvB operon leader peptide	3,268303807	-2,432747446
NC_016856	RefSeq	CDS	4011519	4011680	+	0	ID=cds4426	0	0	STM14_4580	STM14_4580	hypothetical protein	0	NA
NC_016856	RefSeq	CDS	4011613	4011768	-	0	ID=cds4427	0	0,5	STM14_4581	STM14_4581	hypothetical protein	0	NA
NC_016856	RefSeq	CDS	4011777	4011950	+	0	ID=cds4428	0	0	STM14_4582	STM14_4582	hypothetical protein	0	NA
NC_016856	RefSeq	CDS	4012345	4013178	+	0	ID=cds4429	0	3,5	STM14_4585	STM14_4585	integral membrane protein	0,568400662	-1,710438693
NC_016856	RefSeq	CDS	4013381	4014565	+	0	ID=cds4430	0	4	STM14_4586	emrD	multidrug resistance protein D	0,568400662	-1,710438693
NC_016856	RefSeq	CDS	4014492	4015577	-	0	ID=cds4431	0	1	STM14_4587	STM14_4587	hypothetical protein	0,142100166	-0,623264422
NC_016856	RefSeq	CDS	4015641	4016564	-	0	ID=cds4432	0	1	STM14_4588	dsdC	DNA-binding transcriptional regulator DsdC	0,142100166	-0,623264422
NC_016856	RefSeq	CDS	4016572	4016697	-	0	ID=cds4433	0	0	STM14_4589	STM14_4589	hypothetical protein	0	NA
NC_016856	RefSeq	CDS	4016790	4018127	+	0	ID=cds4434	0	0,5	STM14_4590	dsdX	permease DsdX	0	NA
NC_016856	RefSeq	CDS	4018145	4019467	+	0	ID=cds4435	0	15	STM14_4591	dsdA	D-serine dehydratase	2,131502483	-2,392032191
NC_016856	RefSeq	CDS	4019549	4020079	-	0	ID=cds4436	0	21,25	STM14_4592	yidF	putative cytoplasmic protein	2,984103476	-2,430148754
NC_016856	RefSeq	CDS	4020076	4020438	-	0	ID=cds4437	0	3,25	STM14_4593	yidG	putative inner membrane protein	0,426300497	-1,498211557
NC_016856	RefSeq	CDS	4020428	4020775	-	0	ID=cds4438	0	1	STM14_4594	yidH	putative inner membrane protein	0,142100166	-0,623264422
NC_016856	RefSeq	CDS	4020774	4020887	+	0	ID=cds4439	0	1	STM14_4595	STM14_4595	hypothetical protein	0,142100166	-0,623264422
NC_016856	RefSeq	CDS	4021219	4022880	-	0	ID=cds4440	0	14,5	STM14_4597	yidE	hypothetical protein	1,989402318	-2,376535557
NC_016856	RefSeq	CDS	4022921	4023073	+	0	ID=cds4441	0	0	STM14_4598	STM14_4598	hypothetical protein	0	NA
NC_016856	RefSeq	CDS	4023070	4023498	-	0	ID=cds4442	0	1	STM14_4599	ibpB	heat shock chaperone IbpB	0,142100166	-0,623264422
NC_016856	RefSeq	CDS	4023608	4024027	-	0	ID=cds4443	4	10	STM14_4600	ibpA	heat shock protein IbpA	8,458291135	1,501349535
NC_016856	RefSeq	CDS	4024130	4024306	+	0	ID=cds4444	0	0,5	STM14_4601	STM14_4601	hypothetical protein	0	NA
NC_016856	RefSeq	CDS	4024328	4024663	+	0	ID=cds4445	0	5	STM14_4602	yidQ	hypothetical protein	0,710500828	-1,864806942
NC_016856	RefSeq	CDS	4024665	4025891	-	0	ID=cds4446	0	10	STM14_4603	yidR	putative cytoplasmic protein	1,421001655	-2,256999172
NC_016856	RefSeq	CDS	4026013	4027056	-	0	ID=cds4447	0	5,25	STM14_4604	ccmH_2	putative heme lyase subunit	0,710500828	-1,864806942
NC_016856	RefSeq	CDS	4027053	4027610	-	0	ID=cds4448	0	5,25	STM14_4605	ccmG_2	heme lyase disulfide oxidoreductase	0,710500828	-1,864806942
NC_016856	RefSeq	CDS	4027607	4029538	-	0	ID=cds4449	0	4,5	STM14_4606	ccmF_2	cytochrome c-type biogenesis protein	0,568400662	-1,710438693
NC_016856	RefSeq	CDS	4029535	4030014	-	0	ID=cds4450	0	1,5	STM14_4607	ccmE_2	cytochrome c-type biogenesis protein CcmE	0,284200331	-1,182723423
NC_016856	RefSeq	CDS	4030011	4030223	-	0	ID=cds4451	0	1,25	STM14_4608	ccmD_2	heme exporter protein C	0,142100166	-0,623264422
NC_016856	RefSeq	CDS	4030220	4030957	-	0	ID=cds4452	0	3,5	STM14_4609	ccmC_2	heme exporter protein	0,568400662	-1,710438693
NC_016856	RefSeq	CDS	4031009	4031668	-	0	ID=cds4453	0	1,25	STM14_4610	ccmB_2	heme exporter protein	0,142100166	-0,623264422
NC_016856	RefSeq	CDS	4031665	4032282	-	0	ID=cds4454	0	1	STM14_4611	ccmA_2	cytochrome c biogenesis protein CcmA	0,142100166	-0,623264422
NC_016856	RefSeq	CDS	4032288	4033688	-	0	ID=cds4455	0	19,5	STM14_4612	STM14_4612	putative cytochrome c peroxidase	2,842003311	-2,427810709
NC_016856	RefSeq	CDS	4033878	4034510	-	0	ID=cds4456	0	0	STM14_4613	torD	chaperone protein TorD	0	NA
NC_016856	RefSeq	CDS	4034503	4037055	-	0	ID=cds4457	0	1	STM14_4614	torA	trimethylamine N-oxide reductase subunit	0,142100166	-0,623264422
NC_016856	RefSeq	CDS	4037045	4038229	-	0	ID=cds4458	0	0	STM14_4615	torC	trimethylamine N-oxide reductase cytochrome c-like subu	0	NA
NC_016856	RefSeq	CDS	4038359	4039051	+	0	ID=cds4459	0	9,5	STM14_4616	torR	DNA-binding transcriptional regulator TorR	1,421001655	-2,256999172
NC_016856	RefSeq	CDS	4039024	4040064	-	0	ID=cds4460	0	9,75	STM14_4617	torT	periplasmic sensory protein associated with the TorRS twe	1,421001655	-2,256999172
NC_016856	RefSeq	CDS	4040144	4042879	+	0	ID=cds4461	0	8,5	STM14_4618	torS	hybrid sensory histidine kinase TorS	1,136801324	-2,147403465
NC_016856	RefSeq	CDS	4042905	4044242	-	0	ID=cds4462	0	0,5	STM14_4619	dgoT	D-galactonate transport protein	0	NA
NC_016856	RefSeq	CDS	4044327	4045475	-	0	ID=cds4463	0	3	STM14_4620	dgoA	2-oxo-3-deoxygalactonate 6-phosphate aldolase%2Fgalact	0,426300497	-1,498211557
NC_016856	RefSeq	CDS	4046073	4046951	-	0	ID=cds4464	0	2	STM14_4622	dgoK	2-oxo-3-deoxygalactonate kinase	0,284200331	-1,182723423
NC_016856	RefSeq	CDS	4046948	4047637	-	0	ID=cds4465	0	2,5	STM14_4623	dgoR	galactonate operon transcriptional repressor	0,284200331	-1,182723423
NC_016856	RefSeq	CDS	4047898	4048743	-	0	ID=cds4466	0	9	STM14_4624	yidA	sugar phosphatase	1,27890149	-2,207741391
NC_016856	RefSeq	CDS	4049049	4050311	+	0	ID=cds4467	0	2	STM14_4625	STM14_4625	putative permease	0,284200331	-1,182723423
NC_016856	RefSeq	CDS	4050325	4051518	+	0	ID=cds4468	0	0,5	STM14_4626	STM14_4626	putative mandelate racemase	0	NA
NC_016856	RefSeq	CDS	4051633	4052529	+	0	ID=cds4469	0	13	STM14_4627	STM14_4627	putative transcriptional regulator	1,847302152	-2,356210635
NC_016856	RefSeq	CDS	4052548	4054962	-	0	ID=cds4470	0	163	STM14_4628	gyrB	DNA gyrase subunit B	23,16232698	-1,883691034
NC_016856	RefSeq	CDS	4054991	4056064	-	0	ID=cds4471	0	23	STM14_4629	recF	recombination protein F	3,268303807	-2,432747446
NC_016856	RefSeq	CDS	4056093	4056239	+	0	ID=cds4472	0	0	STM14_4630	STM14_4630	hypothetical protein	0	NA
NC_016856	RefSeq	CDS	4056212	4057312	-	0	ID=cds4473	0	47,75	STM14_4631	dnaN	DNA polymerase III subunit beta	6,820807946	-2,185729286
NC_016856	RefSeq	CDS	4057317	4058717	-	0	ID=cds4474	0	63,5	STM14_4632	dnaA	chromosomal replication initiation protein	9,094410594	-2,079502768
NC_016856	RefSeq	CDS	4059079	4059237	+	0	ID=cds4475	0	5	STM14_4633	STM14_4633	hypothetical protein	0,710500828	-1,864806942

NC_01685(	RefSeq	CDS	4059378	4059518	+	0	ID=cds4476	3	60,25	STM14_4634	rpmH	50S ribosomal protein L34	13,80397704	-0,474946798
NC_01685(	RefSeq	CDS	4059535	4059894	+	0	ID=cds4477	0	33,08333333	STM14_4635	rnpA	ribonuclease P	4,689305463	-2,505858154
NC_01685(	RefSeq	CDS	4059858	4060115	+	0	ID=cds4478	0	9	STM14_4636	STM14_4636	hypothetical protein	1,27890149	-2,207741391
NC_01685(	RefSeq	CDS	4060118	4061764	+	0	ID=cds4479	2	54,83333333	STM14_4637	yidC	putative inner membrane protein translocase component	11,33415384	-0,770146969
NC_01685(	RefSeq	CDS	4061906	4063270	+	0	ID=cds4480	0	5,5	STM14_4638	trmE	tRNA modification GTPase TrmE	0,852600993	-1,98056697
NC_01685(	RefSeq	CDS	4065071	4066033	+	0	ID=cds4481	0	18,5	STM14_4640	STM14_4640	putative inner membrane protein	2,55780298	-2,419166771
NC_01685(	RefSeq	CDS	4066033	4066968	+	0	ID=cds4482	0	11	STM14_4641	STM14_4641	putative reverse transcriptase	1,563101821	-2,297202414
NC_01685(	RefSeq	CDS	4067770	4068957	+	0	ID=cds4483	0	0	STM14_4642	yidY	multidrug efflux system protein MdtL	0	NA
NC_01685(	RefSeq	CDS	4068926	4069885	+	0	ID=cds4484	0	2,5	STM14_4643	yidZ	DNA-binding transcriptional regulator YidZ	0,284200331	-1,182723423
NC_01685(	RefSeq	CDS	4070043	4070798	+	0	ID=cds4485	0	3,5	STM14_4644	yieE	putative cytoplasmic protein	0,568400662	-1,710438693
NC_01685(	RefSeq	CDS	4070816	4071382	+	0	ID=cds4486	0	11	STM14_4645	yieF	putative oxidoreductase	1,563101821	-2,297202414
NC_01685(	RefSeq	CDS	4071427	4072764	-	0	ID=cds4487	0	10,5	STM14_4646	yieG	putative xanthine%2Furacil permease family protein	1,421001655	-2,256999172
NC_01685(	RefSeq	CDS	4072933	4073598	+	0	ID=cds4488	0	2,5	STM14_4647	yieH	6-phosphogluconate phosphatase	0,284200331	-1,182723423
NC_01685(	RefSeq	CDS	4073693	4074418	-	0	ID=cds4489	0	3,75	STM14_4648	phoU	transcriptional regulator PhoU	0,568400662	-1,710438693
NC_01685(	RefSeq	CDS	4074433	4075206	-	0	ID=cds4490	0	5,25	STM14_4649	pstB	phosphate transporter subunit	0,710500828	-1,864806942
NC_01685(	RefSeq	CDS	4075293	4076183	-	0	ID=cds4491	0	3	STM14_4650	pstA	phosphate transporter permease subunit	0,426300497	-1,498211557
NC_01685(	RefSeq	CDS	4076183	4077142	-	0	ID=cds4492	0	5	STM14_4651	pstC	phosphate transporter permease subunit	0,710500828	-1,864806942
NC_01685(	RefSeq	CDS	4077278	4078318	-	0	ID=cds4493	0	4,5	STM14_4652	pstS	phosphate transporter subunit	0,568400662	-1,710438693
NC_01685(	RefSeq	CDS	4078485	4079858	-	0	ID=cds4494	0	3	STM14_4653	STM14_4653	putative phosphotransferase system fructose-specific con	0,426300497	-1,498211557
NC_01685(	RefSeq	CDS	4079907	4080725	-	0	ID=cds4495	0	0	STM14_4654	aroE_3	shikimate 5-dehydrogenase	0	NA
NC_01685(	RefSeq	CDS	4080820	4082577	+	0	ID=cds4496	0	1	STM14_4655	STM14_4655	putative dipeptide%2Foligopeptide%2Fnickel ABC-type tra	0,142100166	-0,623264422
NC_01685(	RefSeq	CDS	4082728	4084557	-	0	ID=cds4497	0	147	STM14_4656	glmS	D-fructose-6-phosphate amidotransferase	20,88872433	-1,874094221
NC_01685(	RefSeq	CDS	4084746	4086116	-	0	ID=cds4498	0	86	STM14_4657	glmU	bifunctional N-acetylglucosamine-1-phosphate uridyltrans	12,22061424	-1,963849549
NC_01685(	RefSeq	CDS	4086316	4086504	+	0	ID=cds4499	0	0	STM14_4658	STM14_4658	hypothetical protein	0	NA
NC_01685(	RefSeq	CDS	4086436	4087134	-	0	ID=cds4500	1	3	STM14_4659	STM14_4659	putative permease	2,185622867	1,419750352
NC_01685(	RefSeq	CDS	4087364	4087783	-	0	ID=cds4501	0	47	STM14_4660	atpC	F0F1 ATP synthase subunit epsilon	6,67870778	-2,193319559
NC_01685(	RefSeq	CDS	4087804	4089186	-	0	ID=cds4502	0	254,5	STM14_4661	atpD	F0F1 ATP synthase subunit beta	36,09344205	-1,917234455
NC_01685(	RefSeq	CDS	4089213	4090076	-	0	ID=cds4503	0	148,5	STM14_4662	atpG	F0F1 ATP synthase subunit gamma	21,0308245	-1,874746658
NC_01685(	RefSeq	CDS	4090127	4091668	-	0	ID=cds4504	0	215,25	STM14_4663	atpA	F0F1 ATP synthase subunit alpha	30,55153559	-1,905986344
NC_01685(	RefSeq	CDS	4091681	4092214	-	0	ID=cds4505	0	41,58333333	STM14_4664	atpH	F0F1 ATP synthase subunit delta	5,968206953	-2,232890319
NC_01685(	RefSeq	CDS	4092229	4092699	-	0	ID=cds4506	0	86,5	STM14_4665	atpF	F0F1 ATP synthase subunit B	12,22061424	-1,963849549
NC_01685(	RefSeq	CDS	4092759	4092998	-	0	ID=cds4507	0	137	STM14_4666	atpE	F0F1 ATP synthase subunit C	19,46772268	-1,867120417
NC_01685(	RefSeq	CDS	4093045	4093860	-	0	ID=cds4508	0	105,75	STM14_4667	atpB	F0F1 ATP synthase subunit A	15,06261755	-1,883514636
NC_01685(	RefSeq	CDS	4093869	4094249	-	0	ID=cds4509	0	25,75	STM14_4668	atpI	F0F1 ATP synthase subunit I	3,694604304	-2,438835558
NC_01685(	RefSeq	CDS	4094364	4094507	-	0	ID=cds4510	0	1	STM14_4669	STM14_4669	hypothetical protein	0,142100166	-0,623264422
NC_01685(	RefSeq	CDS	4094865	4095494	-	0	ID=cds4511	0	14	STM14_4670	gidB	glucose-inhibited division protein B	1,989402318	-2,376535557
NC_01685(	RefSeq	CDS	4095591	4097480	-	0	ID=cds4512	0	43	STM14_4671	gidA	tRNA uridine 5-carboxymethylaminomethyl modification (	6,110307118	-2,224756213
NC_01685(	RefSeq	CDS	4097859	4098302	-	0	ID=cds4513	0	29	STM14_4672	mioC	flavodoxin	4,120904801	-2,455089955
NC_01685(	RefSeq	CDS	4098392	4098850	-	0	ID=cds4514	0	5	STM14_4673	asnC	DNA-binding transcriptional regulator AsnC	0,710500828	-1,864806942
NC_01685(	RefSeq	CDS	4099001	4099993	+	0	ID=cds4515	0	20,5	STM14_4674	asnA	asparagine synthetase AsnA	2,842003311	-2,427810709
NC_01685(	RefSeq	CDS	4099998	4101449	-	0	ID=cds4516	0	11,5	STM14_4675	yieM	hypothetical protein	1,705201986	-2,329925387
NC_01685(	RefSeq	CDS	4101443	4103047	-	0	ID=cds4517	0	18	STM14_4676	yieN	regulatory ATPase RavA	2,55780298	-2,419166771
NC_01685(	RefSeq	CDS	4103287	4105155	+	0	ID=cds4518	2	23	STM14_4677	trkD	potassium transport protein Kup	6,786948547	0,069120359
NC_01685(	RefSeq	CDS	4105352	4105771	+	0	ID=cds4519	5	3	STM14_4678	rbsD	D-ribose pyranase	9,222912346	2,70091526
NC_01685(	RefSeq	CDS	4105779	4107284	+	0	ID=cds4520	0	2,5	STM14_4679	rbsA	D-ribose transporter ATP-binding protein	0,284200331	-1,182723423
NC_01685(	RefSeq	CDS	4107290	4108255	+	0	ID=cds4521	0	3,75	STM14_4680	rbsC	ribose ABC transporter permease	0,568400662	-1,710438693
NC_01685(	RefSeq	CDS	4108280	4109170	+	0	ID=cds4522	0	35,75	STM14_4681	rbsB	D-ribose transporter subunit RbsB	5,115605959	-2,281753063
NC_01685(	RefSeq	CDS	4109304	4110233	+	0	ID=cds4523	0	30	STM14_4682	rbsK	ribokinase	4,263004966	-2,464060063
NC_01685(	RefSeq	CDS	4110237	4111235	+	0	ID=cds4524	0	16	STM14_4683	rbsR	transcriptional repressor RbsR	2,273602649	-2,403729808
NC_01685(	RefSeq	CDS	4111201	4112628	-	0	ID=cds4525	0	6	STM14_4684	yieO	putative tranport protein	0,852600993	-1,98056697
NC_01685(	RefSeq	CDS	4112640	4113344	-	0	ID=cds4526	3	10	STM14_4685	yieP	putative regulatory protein	6,698968765	1,219842116
NC_01685(	RefSeq	ncRNA	4118719	4118817	+	.	ID=rna82;P	13,00089286	300,9449405	STM14_4689	STM14_4689	regulatory RNA 9S	65,64334064	-0,644635914
NC_01685(	RefSeq	CDS	4119236	4120084	-	0	ID=cds4527	0	12	STM14_4693	yifA	transcriptional regulator HdfR	1,705201986	-2,329925387
NC_01685(	RefSeq	CDS	4120191	4120529	+	0	ID=cds4528	0	6	STM14_4694	yifE	hypothetical protein	0,852600993	-1,98056697
NC_01685(	RefSeq	CDS	4120554	4122074	-	0	ID=cds4529	0	2,5	STM14_4695	yifB	putative ATP-dependent protease	0,284200331	-1,182723423
NC_01685(	RefSeq	CDS	4122161	4122346	+	0	ID=cds4530	0	0,5	STM14_4696	STM14_4696	hypothetical protein	0	NA
NC_01685(	RefSeq	CDS	4122426	4122524	+	0	ID=cds4531	5	18,5	STM14_4697	ilvL	ilvG operon leader peptide	11,35441483	1,202027933
NC_01685(	RefSeq	CDS	4122664	4124310	+	0	ID=cds4532	0	20,5	STM14_4698	ilvG	acetolactate synthase 2 catalytic subunit	2,842003311	-2,427810709
NC_01685(	RefSeq	CDS	4124310	4124570	+	0	ID=cds4533	0	0	STM14_4699	ilvM	acetolactate synthase 2 regulatory subunit	0	NA
NC_01685(	RefSeq	CDS	4124588	4125517	+	0	ID=cds4534	0	3,5	STM14_4700	ilvE	branched-chain amino acid aminotransferase	0,568400662	-1,710438693
NC_01685(	RefSeq	CDS	4125678	4127528	+	0	ID=cds4535	0	10	STM14_4701	ilvD	dihydroxy-acid dehydratase	1,421001655	-2,256999172
NC_01685(	RefSeq	CDS	4127531	4129075	+	0	ID=cds4536	0	10,5	STM14_4702	ilvA	threonine dehydratase	1,421001655	-2,256999172
NC_01685(	RefSeq	CDS	4129359	4129676	+	0	ID=cds4537	0	1	STM14_4703	STM14_4703	putative cytoplasmic protein	0,142100166	-0,623264422
NC_01685(	RefSeq	CDS	4129673	4130014	+	0	ID=cds4538	0	2	STM14_4704	STM14_4704	putative cytoplasmic protein	0,284200331	-1,182723423
NC_01685(	RefSeq	CDS	4130017	4130904	-	0	ID=cds4539	0	3	STM14_4705	ilvY	DNA-binding transcriptional regulator IlvY	0,426300497	-1,498211557
NC_01685(	RefSeq	CDS	4131068	4132543	+	0	ID=cds4540	0	7,5	STM14_4706	ilvC	ketol-acid reductoisomerase	1,136801324	-2,147403465
NC_01685(	RefSeq	CDS	4132635	4132916	-	0	ID=cds4541	0	9,25	STM14_4707	ppiC	peptidyl-prolyl cis-trans isomerase C	1,27890149	-2,207741391
NC_01685(	RefSeq	CDS	4132921	4133268	+	0	ID=cds4542	0	1	STM14_4708	STM14_4708	cyclic nucleotide-binding domain-containing protein	0,142100166	-0,623264422
NC_01685(	RefSeq	CDS	4133205	4133354	+	0	ID=cds4543	0	2	STM14_4709	STM14_4709	putative inner membrane protein	0,284200331	-1,182723423
NC_01685(	RefSeq	CDS	4133355	4135478	+	0	ID=cds4544	0	15	STM14_4710	rep	ATP-dependent DNA helicase Rep	2,131502483	-2,392032191
NC_01685(	RefSeq	CDS	4135518	4136999	-	0	ID=cds4545	0	74,5	STM14_4711	gppA	guanosine pentaphosphate phosphohydrolase	10,51541225	-2,024396986
NC_01685(	RefSeq	CDS	4137118	4138383	-	0	ID=cds4546	0	104,5	STM14_4712	rhlB	ATP-dependent RNA helicase RhlB	14,77841722	-1,890380653
NC_01685(	RefSeq	CDS	4138527	4138856	+	0	ID=cds4547	0	75,33333333	STM14_4713	trxA	thioredoxin	10,65751242	-2,019002164
NC_01685(	RefSeq	CDS	4139275	4140534	+	0	ID=cds4548	0	226,8333333	STM14_4715	rho	transcription termination factor Rho	32,25673758	-1,90981892
NC_01685(	RefSeq	CDS	4140764	4141867	+	0	ID=cds4549	0	23,5	STM14_4716	rfe	undecaprenyl-phosphate N-acetylglucosaminyltransferase	3,410403973	-2,434086198
NC_01685(	RefSeq	CDS	4141879	4142925	+	0	ID=cds4550	3	30	STM14_4717	wzzE	lipopolysaccharide biosynthesis protein WzzE	9,540972076	0,205938589
NC_01685(	RefSeq	CDS	4142981	4144111	+	0	ID=cds4551	1	34,33333333	STM14_4718	wecB	UDP-N-acetyl glucosamine-2-epimerase	6,590727998	-0,915216377
NC_01685(	RefSeq	CDS	4144108	4145370	+	0	ID=cds4552	0	30	STM14_4719	wecC	UDP-N-acetyl-D-mannosamine dehydrogenase	4,263004966	-2,464060063
NC_01685(	RefSeq	CDS	4145370	4146437	+	0	ID=cds4553	0	22	STM14_4720	rffG	dTDP-glucose 4%2C6-dehydratase	3,126203642	-2,431590384
NC_01685(	RefSeq	CDS	4146470	4146694	+	0	ID=cds4554	0	3,5	STM14_4721	rffH	glucose-1-phosphate thymidylyltransferase	0,568400662	-1,710438693
NC_01685(	RefSeq	CDS	4146672	4147349	+	0	ID=cds4555	0	9,5	STM14_4722	wecD	TDP-fucosamine acetyltransferase	1,421001655	-2,256999172
NC_01685(	RefSeq	CDS	4147354	4148484	+	0	ID=cds4556	0	25,5	STM14_4723	wecE	TDP-4-oxo-6-deoxy-D-glucose transaminase	3,694604304	-2,438835558
NC_01685(	RefSeq	CDS	4148486	4149736	+	0	ID=cds4557	0	19,5	STM14_4724	wzxE	O-antigen translocase	2,842003311	-2,427810709
NC_01685(	RefSeq	CDS	4149733	4150812	+	0	ID=cds4558	0	26,33333333	STM14_4725	STM14_4725	4-alpha-L-fucosyltransferase	3,694604304	-2,438835558
NC_01685(	RefSeq	CDS	4150809	4152167	+	0	ID=cds4559	0	24,75	STM14_4726	wecF	putative entero common antigen polymerase	3,552504138	-2,43600272
NC_01685(	RefSeq	CDS	4152164	4152904	+	0	ID=cds4560	0	6,75	STM14_4727	wecG	putative UDP-N-acetyl-D-mannosaminuronic acid transfer	0,994701159	-2,072978628
NC_01685(	RefSeq	CDS	4153111	4154496	+	0	ID=cds4561	0	10	STM14_4728	yifK	putative transport protein YifK	1,421001655	-2,256999172
NC_01685(	RefSeq	CDS	4155257	4155382	+	0	ID=cds4562	0	41	STM14_4733	STM14_4733	hypothetical protein	5,826106787	-2,241137745
NC_01685(	RefSeq	CDS	4155613	4156812	-	0	ID=cds4563	0	45,5	STM14_4734	hemY	putative protoheme IX biogenesis protein	6,536607615	-2,201017198
NC_01685(	RefSeq	CDS	4156812	4157981	-	0	ID=cds4564	0	35,5	STM14_4735	hemX	putative uroporphyrinogen III C-methyltransferase	5,115605959	-2,281753063
NC_01685(	RefSeq	CDS	4158003	4158743	-	0	ID=cds4565	0	22	STM14_4736	hemD	uroporphyrinogen-III synthase	3,126203642	-2,431590384
NC_01685(	RefSeq	CDS	4158740	4159696	-	0	ID=cds4566	0	67	STM14_4737	hemC	porphobilinogen deaminase	9,520711091	-2,064144167
NC_01685(	RefSeq	CDS	4159908	4160012	-	0	ID=cds4567	0	0	STM14_4738	STM14_4738	hypothetical protein	0	NA
NC_01685(	RefSeq	CDS	4160058	4162604	+	0	ID=cds4568	1	110	STM14_4739	cyaA	adenylate cyclase	17,39034058	-2,046189894
NC_01685(	RefSeq	CDS	4162570	4162710	+	0	ID=cds4569	0	1	STM14_4740	STM14_4740	hypothetical protein	0,142100166	-0,623264422
NC_01685(	RefSeq	CDS	4162712	4163065	+	0	ID=cds4570	0	0	STM14_4741	STM14_4741	putative inner membrane protein	0	NA
NC_01685(	RefSeq	CDS	4163251	4163793	+	0	ID=cds4571	0	0	STM14_4742	STM14_4742	putative inner membrane protein	0	NA
NC_01685(	RefSeq	CDS	4163805	4164167	+	0	ID=cds4572	0	0	STM14_4743	STM14_4743	putative cytoplasmic protein	0	NA
NC_01685(	RefSeq	CDS	4164238	4164558	-	0	ID=cds4573	0	3	STM14_4744	cyaY	frataxin-like protein	0,426300497	-1,498211557
NC_01685(	RefSeq	CDS	4164628	4165017	-	0	ID=cds4574	0	0	STM14_4745	STM14_4745	putative inner membrane protein	0	NA
NC_01685(	RefSeq	CDS	4165713	4165916	+	0	ID=cds4575	0	9	STM14_4747	yifL	putative outer membrane lipoprotein	1,27890149	-2,207741391
NC_01685(	RefSeq	CDS	4165953	4166777	+	0	ID=cds4576	0	35	STM14_4748	dapF	diaminopimelate epimerase	4,973505794	-2,290637485
NC_01685(	RefSeq	CDS	4166774	4167481	+	0	ID=cds4577	0	35,5	STM14_4749	yigA	hypothetical protein	5,115605959	-2,281753063
NC_01685(	RefSeq	CDS	4167478	4168380	+	0	ID=cds4578	0,833333333	19,5	STM14_4750	xerC	site-specific tyrosine recombinase XerC	4,601325681	-0,429413798
NC_01685(	RefSeq	CDS	4168380	4169096	+	0	ID=cds4579	0	12,5	STM14_4751	yigB	flavin mononucleotide phosphatase	1,705201986	-2,329925387
NC_01685(	RefSeq	CDS	4169232	4171394	+	0	ID=cds4580	0	37,83333333	STM14_4752	uvrD	DNA-dependent helicase II	5,39980629	-2,264331336
NC_01685(	RefSeq	CDS	4171444	4171629	-	0	ID=cds4581	0	0	STM14_4753	STM14_4753	hypothetical protein	0	NA
NC_01685(	RefSeq	CDS	4171866	4172816	+	0	ID=cds4582	0	17,16666667	STM14_4754	corA	magnesium%2Fnickel%2Fcobalt transporter CorA	2,415702814	-2,412509737
NC_01685(	RefSeq	CDS	4172865	4173245	-	0	ID=cds4583	0	0	STM14_4755	yigF	putative inner membrane protein	0	NA
NC_01685(	RefSeq	CDS	4173261	4173719	-	0	ID=cds4584	0	0	STM14_4756	yigG	hypothetical protein	0	NA
NC_01685(	RefSeq	CDS	4173754	4174638	-	0	ID=cds4585	0	4	STM14_4757	rarD	chloramphenicol resistance	0,568400662	-1,710438693
NC_01685(	RefSeq	CDS	4174682	4175149	-	0	ID=cds4586	0	0	STM14_4758	yigI	hypothetical protein	0	NA
NC_01685(	RefSeq	CDS	4175211	4175348	-	0	ID=cds4587	0	0	STM14_4759	STM14_4759	hypothetical protein	0	NA
NC_01685(	RefSeq	CDS	4175314	4176183	+	0	ID=cds4588	0	21,25	STM14_4760	pldA	phospholipase A	2,984103476	-2,430148754
NC_01685(	RefSeq	CDS	4176249	4178096	+	0	ID=cds4589	0	33,5	STM14_4761	recQ	ATP-dependent DNA helicase RecQ	4,831405628	-2,523251848
NC_01685(	RefSeq	CDS	4178160	4178780	+	0	ID=cds4590	0	5	STM14_4762	rhtC	threonine efflux system	0,710500828	-1,864806942
NC_01685(	RefSeq	CDS	4178820	4179440	-	0	ID=cds4591	0	2	STM14_4763	rhtB	homoserine%2Fhomoserine lactone efflux protein	0,284200331	-1,182723423
NC_01685(	RefSeq	CDS	4179455	4180567	+	0	ID=cds4592	0	13,5	STM14_4764	pldB	lysophospholipase L2	1,989402318	-2,376535557
NC_01685(	RefSeq	CDS	4180583	4181383	+	0	ID=cds4593	0	14	STM14_4765	yigL	putative sugar phosphatase	1,989402318	-2,376535557
NC_01685(	RefSeq	CDS	4181464	4182363	+	0	ID=cds4594	0	2,5	STM14_4766	yigM	putative transport protein	0,284200331	-1,182723423
NC_01685(	RefSeq	CDS	4182251	4183204	-	0	ID=cds4595	0	1	STM14_4767	metR	metE%2FmetH regulator	0,142100166	-0,623264422
NC_01685(	RefSeq	CDS	4183187	4183360	+	0	ID=cds4596	0	0	STM14_4768	STM14_4768	hypothetical protein	0	NA
NC_01685(	RefSeq	CDS	4183453	4185717	+	0	ID=cds4597	0,5	3,5	STM14_4769	metE	5-methyltetrahydropteroyltriglutamate%2Fhomocysteine	0,568400662	-1,710438693
NC_01685(	RefSeq	CDS	4185870	4186004	-	0	ID=cds4598	0	1	STM14_4770	STM14_4770	hypothetical protein	0,142100166	-0,623264422

NC_016856	RefSeq	CDS	4185906	4186052	+		0	ID=cds4599	0	0,333333333	STM14_4771	STM14_4771	hypothetical protein	0	NA
NC_016856	RefSeq	CDS	4186061	4187404	+		0	ID=cds4600	0	5,333333333	STM14_4772	STM14_4772	putative arylsulfatase regulator	0,710500828	-1,864806942
NC_016856	RefSeq	CDS	4187484	4188296	-		0	ID=cds4601	0	7,5	STM14_4773	dlhH	putative dienelactone hydrolase	1,136801324	-2,147403465
NC_016856	RefSeq	CDS	4188555	4189316	+		0	ID=cds4602	0	23	STM14_4774	udp	uridine phosphorylase	3,268303807	-2,432747446
NC_016856	RefSeq	CDS	4189456	4190886	+		0	ID=cds4603	0	16,5	STM14_4775	yigN	DNA recombination protein RmuC	2,273602649	-2,403729808
NC_016856	RefSeq	CDS	4190982	4191737	+		0	ID=cds4604	0	32,58333333	STM14_4776	ubiE	ubiquinone%2Fmenaquinone biosynthesis methyltransfer	4,689305463	-2,505858154
NC_016856	RefSeq	CDS	4191747	4192352	+		0	ID=cds4605	0	39,75	STM14_4777	yigP	putative inner membrane protein	5,684006621	-2,24950002
NC_016856	RefSeq	CDS	4192349	4193989	+		0	ID=cds4606	0	52	STM14_4778	ubiB	putative ubiquinone biosynthesis protein UbiB	7,389208608	-2,158807911
NC_016856	RefSeq	CDS	4194195	4194449	+		0	ID=cds4607	1	27	STM14_4779	tatA	twin argininte translocase protein A	5,596026839	-0,703064376
NC_016856	RefSeq	CDS	4194453	4195001	+		0	ID=cds4608	2	36	STM14_4780	tatB	sec-independent translocase	8,634250699	-0,354662722
NC_016856	RefSeq	CDS	4195004	4195783	+		0	ID=cds4609	0	64	STM14_4781	tatC	TatABCE protein translocation system subunit	9,094410594	-2,079502768
NC_016856	RefSeq	CDS	4195813	4196607	+		0	ID=cds4610	0	11,5	STM14_4782	yigW	DNase TatD	1,705201986	-2,329925387
NC_016856	RefSeq	CDS	4196615	4197103	-		0	ID=cds4611	0	3	STM14_4783	rfaH	transcriptional activator RfaH	0,426300497	-1,498211557
NC_016856	RefSeq	CDS	4197098	4197223	+		0	ID=cds4612	0	0,5	STM14_4784	STM14_4784	hypothetical protein	0	NA
NC_016856	RefSeq	CDS	4197289	4198767	+		0	ID=cds4613	0	48,5	STM14_4785	yigC	3-octaprenyl-4-hydroxybenzoate carboxy-lyase	6,820807946	-2,185729286
NC_016856	RefSeq	CDS	4198853	4199554	+		0	ID=cds4614	0	26	STM14_4786	fre	FMN reductase	3,694604304	-2,438835558
NC_016856	RefSeq	CDS	4199808	4201631	+		0	ID=cds4615	0	20	STM14_4787	assT	arylsulfotransferase	2,842003311	-2,427810709
NC_016856	RefSeq	CDS	4201828	4202991	-		0	ID=cds4616	0	2	STM14_4788	fadA	3-ketoacyl-CoA thiolase	0,284200331	-1,182723423
NC_016856	RefSeq	CDS	4203001	4205316	-		0	ID=cds4617	0	1	STM14_4789	fadB	multifunctional fatty acid oxidation complex subunit alpha	0,142100166	-0,623264422
NC_016856	RefSeq	CDS	4205380	4206711	+		0	ID=cds4618	0	52,5	STM14_4790	pepQ	proline dipeptidase	7,389208608	-2,158807911
NC_016856	RefSeq	CDS	4206711	4207325	+		0	ID=cds4619	0	12	STM14_4791	yigZ	hypothetical protein	1,705201986	-2,329925387
NC_016856	RefSeq	CDS	4207364	4208815	+		0	ID=cds4620	0	21	STM14_4792	trkH	potassium transporter	2,984103476	-2,430148754
NC_016856	RefSeq	CDS	4208827	4209372	+		0	ID=cds4621	0	14	STM14_4793	hemG	protoporphyrinogen oxidase	1,989402318	-2,376535557
NC_016856	RefSeq	CDS	4211633	4211749	+		0	ID=cds4622	8,873015873	155,5507937	STM14_4797	STM14_4797	hypothetical protein	38,00152715	-0,341111741
NC_016856	RefSeq	CDS	4215049	4215231	-		0	ID=cds4623	0	0,833333333	STM14_4800	STM14_4800	hypothetical protein	0,142100166	-0,623264422
NC_016856	RefSeq	CDS	4215077	4215433	+		0	ID=cds4624	0	2	STM14_4801	STM14_4801	hypothetical protein	0,284200331	-1,182723423
NC_016856	RefSeq	CDS	4215268	4215783	-		0	ID=cds4625	0	3,833333333	STM14_4802	mobB	molybdopterin-guanine dinucleotide biosynthesis protein	0,568400662	-1,710438693
NC_016856	RefSeq	CDS	4215780	4216364	-		0	ID=cds4626	0	2,5	STM14_4803	mobA	molybdopterin-guanine dinucleotide biosynthesis protein	0,284200331	-1,182723423
NC_016856	RefSeq	CDS	4216434	4216703	+		0	ID=cds4627	0	15,5	STM14_4804	yihD	putative cytoplasmic protein	2,273602649	-2,403729808
NC_016856	RefSeq	CDS	4216780	4217766	+		0	ID=cds4628	1	71,5	STM14_4805	yihE	serine%2Fthreonine protein kinase	11,99053429	-1,636234148
NC_016856	RefSeq	CDS	4217783	4218406	+		0	ID=cds4629	0	38	STM14_4806	dsbA	protein disulfide isomerase I	5,39980629	-2,264331336
NC_016856	RefSeq	CDS	4218419	4219327	-		0	ID=cds4630	0	14	STM14_4807	yihG	putative endonuclease	1,989402318	-2,376535557
NC_016856	RefSeq	CDS	4219525	4219656	-		0	ID=cds4631	0	0	STM14_4808	STM14_4808	hypothetical protein	0	NA
NC_016856	RefSeq	CDS	4219715	4222501	+		0	ID=cds4632	1,333333333	66,83333333	STM14_4809	polA	DNA polymerase I	11,28003346	-1,564314751
NC_016856	RefSeq	ncRNA	4222650	4222715	+	.		ID=rna95;P	1,333333333	5,25	STM14_4810	spf	spot 42 RNA	2,469823198	0,89567218
NC_016856	RefSeq	CDS	4222708	4222851	+		0	ID=cds4633	1,333333333	3,5	STM14_4811	STM14_4811	hypothetical protein	2,327723032	1,125268706
NC_016856	RefSeq	CDS	4222836	4223468	-		0	ID=cds4634	0	18	STM14_4812	yihA	GTPase EngB	2,55780298	-2,419166771
NC_016856	RefSeq	CDS	4223708	4223932	+		0	ID=cds4635	90	511	STM14_4813	STM14_4813	putative cytoplasmic protein	230,9521979	0,790292529
NC_016856	RefSeq	CDS	4224051	4224566	+		0	ID=cds4636	0	11,5	STM14_4814	yihI	hypothetical protein	1,705201986	-2,329925387
NC_016856	RefSeq	CDS	4224755	4226128	+		0	ID=cds4637	0	23,33333333	STM14_4815	hemN	coproporphyrinogen III oxidase	3,268303807	-2,432747446
NC_016856	RefSeq	CDS	4226121	4226246	-		0	ID=cds4638	0	17	STM14_4816	STM14_4816	hypothetical protein	2,415702814	-2,412509737
NC_016856	RefSeq	CDS	4226441	4227850	-		0	ID=cds4639	2	6,5	STM14_4817	glnG	nitrogen regulation protein NR%28I%29	4,371245733	1,278786496
NC_016856	RefSeq	CDS	4227859	4228908	-		0	ID=cds4640	0	5,5	STM14_4818	glnL	nitrogen regulation protein NR%28II%29	0,852600993	-1,98056697
NC_016856	RefSeq	CDS	4228860	4229039	-		0	ID=cds4641	0	1,5	STM14_4819	STM14_4819	hypothetical protein	0,284200331	-1,182723423
NC_016856	RefSeq	CDS	4229183	4230592	-		0	ID=cds4642	16	63,5	STM14_4820	glnA	glutamine synthetase	37,24356851	1,137460543
NC_016856	RefSeq	CDS	4230571	4230990	-		0	ID=cds4643	3	4	STM14_4821	STM14_4821	putative cytoplasmic protein	5,846367772	1,996213194
NC_016856	RefSeq	CDS	4230968	4232791	+		0	ID=cds4644	0	85,5	STM14_4822	typA	GTP-binding protein	12,22061424	-1,963849549
NC_016856	RefSeq	CDS	4232846	4233580	-		0	ID=cds4645	0	2	STM14_4823	STM14_4823	putative hydrolase	0,284200331	-1,182723423
NC_016856	RefSeq	CDS	4233565	4234443	-		0	ID=cds4646	0	0	STM14_4824	STM14_4824	putative inner membrane protein	0	NA
NC_016856	RefSeq	CDS	4234440	4235681	-		0	ID=cds4647	0	1,5	STM14_4825	STM14_4825	coproporphyrinogen III oxidase	0,284200331	-1,182723423
NC_016856	RefSeq	CDS	4235683	4236546	-		0	ID=cds4648	0	0	STM14_4826	STM14_4826	putative membrane-associated metal-dependent hydrolase	0	NA
NC_016856	RefSeq	CDS	4236551	4237576	-		0	ID=cds4649	0	1	STM14_4827	STM14_4827	hypothetical protein	0,142100166	-0,623264422
NC_016856	RefSeq	CDS	4237589	4238437	-		0	ID=cds4650	0	0	STM14_4828	STM14_4828	putative cytoplasmic protein	0	NA
NC_016856	RefSeq	CDS	4238594	4239286	-		0	ID=cds4651	0	0	STM14_4829	yshA	outer membrane porin L	0	NA
NC_016856	RefSeq	CDS	4239354	4240775	-		0	ID=cds4652	0	3	STM14_4830	yihO	GPH family transport protein	0,426300497	-1,498211557
NC_016856	RefSeq	CDS	4240821	4242203	-		0	ID=cds4653	0	3	STM14_4831	yihP	GPH family transport protein	0,426300497	-1,498211557
NC_016856	RefSeq	CDS	4242249	4244285	-		0	ID=cds4654	0	5	STM14_4832	yihQ	alpha-glucosidase	0,710500828	-1,864806942
NC_016856	RefSeq	CDS	4244329	4245180	-		0	ID=cds4655	0	0	STM14_4833	yihR	putative aldose-1-epimerase	0	NA
NC_016856	RefSeq	CDS	4245190	4246431	-		0	ID=cds4656	0	2	STM14_4834	yihS	putative isomerase	0,284200331	-1,182723423
NC_016856	RefSeq	CDS	4246447	4247325	-		0	ID=cds4657	0	1	STM14_4835	yihT	putative aldolase	0,142100166	-0,623264422
NC_016856	RefSeq	CDS	4247348	4248244	-		0	ID=cds4658	0	0	STM14_4836	yihU	putative oxidoreductase	0	NA
NC_016856	RefSeq	CDS	4248232	4248351	-		0	ID=cds4659	0	0	STM14_4837	STM14_4837	hypothetical protein	0	NA
NC_016856	RefSeq	CDS	4248403	4249305	+		0	ID=cds4660	0	1	STM14_4838	yihV	putative sugar kinase	0,142100166	-0,623264422
NC_016856	RefSeq	CDS	4249339	4250142	+		0	ID=cds4661	0	7,5	STM14_4839	yihW	putative glycerol-3-phosphate regulon repressor	1,136801324	-2,147403465
NC_016856	RefSeq	CDS	4250333	4250932	+		0	ID=cds4662	3	9	STM14_4840	yihX	phosphatase	6,5568686	1,313600801
NC_016856	RefSeq	CDS	4250926	4251798	+		0	ID=cds4663	0	10,25	STM14_4841	rbn	ribonuclease BN	1,421001655	-2,256999172
NC_016856	RefSeq	CDS	4251795	4252232	+		0	ID=cds4664	0	10,75	STM14_4842	yihZ	D-tyrosyl-tRNA deacylase	1,563101821	-2,297202414
NC_016856	RefSeq	CDS	4252229	4253218	+		0	ID=cds4665	0,5	20	STM14_4843	yiiD	putative acetyltransferase	2,842003311	-2,427810709
NC_016856	RefSeq	CDS	4253233	4253661	-		0	ID=cds4666	0	1,25	STM14_4844	STM14_4844	putative cytoplasmic protein	0,142100166	-0,623264422
NC_016856	RefSeq	CDS	4253676	4253987	-		0	ID=cds4667	0	0	STM14_4845	STM14_4845	putative cytoplasmic protein	0	NA
NC_016856	RefSeq	CDS	4254073	4255002	-		0	ID=cds4668	0	5	STM14_4846	STM14_4846	putative acetyl esterase	0,710500828	-1,864806942
NC_016856	RefSeq	CDS	4255220	4255531	+		0	ID=cds4669	0	2	STM14_4847	STM14_4847	hypothetical protein	0,284200331	-1,182723423
NC_016856	RefSeq	CDS	4255532	4255822	+		0	ID=cds4670	0	0,5	STM14_4848	STM14_4848	putative regulatory protein	0	NA
NC_016856	RefSeq	CDS	4255869	4256798	-		0	ID=cds4671	0	24,5	STM14_4849	fdhE	formate dehydrogenase accessory protein FdhE	3,410403973	-2,434086198
NC_016856	RefSeq	CDS	4256795	4257430	-		0	ID=cds4672	0	25	STM14_4850	fdoI	formate dehydrogenase-O subunit gamma	3,552504138	-2,43600272
NC_016856	RefSeq	CDS	4257427	4258329	-		0	ID=cds4673	0	56,25	STM14_4851	fdoH	formate dehydrogenase-O beta subunit	7,95760927	-2,130811198
NC_016856	RefSeq	CDS	4258342	4261392	-		0	ID=cds4674	0	132,75	STM14_4852	fdoG	formate dehydrogenase alpha subunit	18,89932202	-1,8640798
NC_016856	RefSeq	CDS	4261587	4262423	+		0	ID=cds4675	0	10	STM14_4853	fdhD	formate dehydrogenase accessory protein	1,421001655	-2,256999172
NC_016856	RefSeq	CDS	4262409	4262564	-		0	ID=cds4676	0	0,5	STM14_4854	STM14_4854	hypothetical protein	0	NA
NC_016856	RefSeq	CDS	4262691	4263722	-		0	ID=cds4677	1	4	STM14_4855	STM14_4855	putative inner membrane lipoprotein	2,327723032	1,125268706
NC_016856	RefSeq	CDS	4263770	4263904	+		0	ID=cds4678	0	0	STM14_4856	STM14_4856	hypothetical protein	0	NA
NC_016856	RefSeq	CDS	4263947	4265005	+		0	ID=cds4679	0	13	STM14_4857	yiiG	putative cytoplasmic protein	1,847302152	-2,356210635
NC_016856	RefSeq	CDS	4265002	4265130	+		0	ID=cds4680	0	0	STM14_4858	STM14_4858	hypothetical protein	0	NA
NC_016856	RefSeq	CDS	4265360	4265683	-		0	ID=cds4681	0	0	STM14_4859	STM14_4859	putative inner membrane protein	0	NA
NC_016856	RefSeq	CDS	4265683	4266342	-		0	ID=cds4682	0	6,5	STM14_4860	STM14_4860	putative branched-chain amino acid permease	0,852600993	-1,98056697
NC_016856	RefSeq	CDS	4266425	4266991	+		0	ID=cds4683	0	10,5	STM14_4861	STM14_4861	hypothetical protein	1,421001655	-2,256999172
NC_016856	RefSeq	CDS	4267080	4267394	-		0	ID=cds4684	0	3,5	STM14_4862	yiiL	putative cytoplasmic protein	0,568400662	-1,710438693
NC_016856	RefSeq	CDS	4267391	4268539	-		0	ID=cds4685	0	9	STM14_4863	STM14_4863	putative alcohol dehydrogenase	1,27890149	-2,207741391
NC_016856	RefSeq	CDS	4268666	4269493	-		0	ID=cds4686	0	1,5	STM14_4864	rhaD	rhamnulose-1-phosphate aldolase	0,284200331	-1,182723423
NC_016856	RefSeq	CDS	4269636	4270895	-		0	ID=cds4687	0	0	STM14_4865	rhaA	L-rhamnose isomerase	0	NA
NC_016856	RefSeq	CDS	4270892	4272361	-		0	ID=cds4688	0	0	STM14_4866	rhaB	rhamnulokinase	0	NA
NC_016856	RefSeq	CDS	4272347	4272496	+		0	ID=cds4689	1,5	0,5	STM14_4867	STM14_4867	hypothetical protein	3,51864474	1,925445802
NC_016856	RefSeq	CDS	4272426	4272560	-		0	ID=cds4690	0	0	STM14_4868	STM14_4868	hypothetical protein	0	NA
NC_016856	RefSeq	CDS	4272649	4273485	+		0	ID=cds4691	0	1	STM14_4869	rhaS	transcriptional activator RhaS	0,142100166	-0,623264422
NC_016856	RefSeq	CDS	4273638	4274486	+		0	ID=cds4692	0	3	STM14_4870	rhaR	transcriptional activator RhaR	0,426300497	-1,498211557
NC_016856	RefSeq	CDS	4274483	4275517	-		0	ID=cds4693	0	1	STM14_4871	rhaT	rhamnose-proton symporter	0,142100166	-0,623264422
NC_016856	RefSeq	CDS	4275521	4275652	+		0	ID=cds4694	0	0	STM14_4872	STM14_4872	hypothetical protein	0	NA
NC_016856	RefSeq	CDS	4275986	4276111	+		0	ID=cds4695	0	0	STM14_4873	STM14_4873	hypothetical protein	0	NA
NC_016856	RefSeq	CDS	4276136	4276819	+		0	ID=cds4696	0	0	STM14_4874	STM14_4874	putative outer membrane protein	0	NA
NC_016856	RefSeq	CDS	4276977	4278284	-		0	ID=cds4697	0	1	STM14_4875	STM14_4875	putative C4-dicarboxylate transport system	0,142100166	-0,623264422
NC_016856	RefSeq	CDS	4278277	4278792	-		0	ID=cds4698	0	0	STM14_4876	STM14_4876	putative C4-dicarboxylate transport system	0	NA
NC_016856	RefSeq	CDS	4278811	4279794	-		0	ID=cds4699	0	0	STM14_4877	STM14_4877	putative periplasmic dicarboxylate-binding protein	0	NA
NC_016856	RefSeq	CDS	4280123	4280743	+		0	ID=cds4700	1	19,5	STM14_4878	sodA	superoxide dismutase	4,601325681	-0,429413798
NC_016856	RefSeq	CDS	4280813	4281502	+		0	ID=cds4701	0	12,5	STM14_4879	yiiM	hypothetical protein	1,705201986	-2,329925387
NC_016856	RefSeq	CDS	4281514	4281909	+		0	ID=cds4702	0	19,5	STM14_4880	STM14_4880	putative inner membrane protein	2,842003311	-2,427810709
NC_016856	RefSeq	CDS	4281960	4283333	-		0	ID=cds4703	0	13	STM14_4881	cpxA	two-component sensor protein	1,847302152	-2,356210635
NC_016856	RefSeq	CDS	4283330	4284028	-		0	ID=cds4704	0	16	STM14_4882	cpxR	DNA-binding transcriptional regulator CpxR	2,273602649	-2,403729808
NC_016856	RefSeq	CDS	4284179	4284679	+		0	ID=cds4705	29	57,5	STM14_4883	cpxP	repressor CpxP	59,26215833	1,835778412
NC_016856	RefSeq	CDS	4284827	4285729	+		0	ID=cds4706	3	23	STM14_4884	fieF	ferrous iron efflux protein F	8,546270917	0,457464926
NC_016856	RefSeq	CDS	4285914	4286876	+		0	ID=cds4707	1	32,5	STM14_4885	pfkA	6-phosphofructokinase	6,306527667	-0,86024894
NC_016856	RefSeq	CDS	4286951	4287055	-		0	ID=cds4708	0	0	STM14_4886	STM14_4886	hypothetical protein	0	NA
NC_016856	RefSeq	CDS	4287080	4288069	+		0	ID=cds4709	2	5,5	STM14_4887	sbp	sulfate transporter subunit	4,371245733	1,278786496
NC_016856	RefSeq	CDS	4288170	4288925	+		0	ID=cds4710	0	5	STM14_4888	ushB	CDP-diacylglycerol pyrophosphatase	0,710500828	-1,864806942
NC_016856	RefSeq	CDS	4289188	4290522	+		0	ID=cds4711	0	4,5	STM14_4889	STM14_4889	putative Na%2B%2Fgalactoside symporter	0,568400662	-1,710438693
NC_016856	RefSeq	CDS	4290533	4291492	+		0	ID=cds4712	0	4	STM14_4890	STM14_4890	aminoimidazole riboside kinase	0,568400662	-1,710438693
NC_016856	RefSeq	CDS	4291502	4292542	+		0	ID=cds4713	0	11,5	STM14_4891	STM14_4891	putative ADP-ribosylglycohydrolase	1,705201986	-2,329925387
NC_016856	RefSeq	CDS	4292605	4293327	+		0	ID=cds4714	0	23,83333333	STM14_4892	STM14_4892	putative regulatory protein	3,410403973	-2,434086198
NC_016856	RefSeq	CDS	4293425	4293589	+		0	ID=cds4715	0	0	STM14_4893	STM14_4893	hypothetical protein	0	NA
NC_016856	RefSeq	CDS	4293605	4293736	+		0	ID=cds4716	0	0	STM14_4894	STM14_4894	putative cytoplasmic protein	0	NA
NC_016856	RefSeq	CDS	4293826	4294176	-		0	ID=cds4717	0	4,5	STM14_4895	STM14_4895	putative mannose-6-phosphate isomerase	0,568400662	-1,710438693
NC_016856	RefSeq	CDS	4294190	4295782	-		0	ID=cds4718	0	36	STM14_4896	ydeV	autoinducer-2 %28AI-2%29 kinase	5,115605959	-2,281753063
NC_016856	RefSeq	CDS	4295871	4296830	-		0	ID=cds4719	0	29,5	STM14_4897	ydeW	putative transcriptional repressor	4,263004966	-2,464060063
NC_016856	RefSeq	CDS	4296957	4297091	-		0	ID=cds4720	0	0,5	STM14_4898	STM14_4898	hypothetical protein	0	NA
NC_016856	RefSeq	CDS	4297086	4298621	+		0	ID=cds4721	0	24	STM14_4899	ego	putative ABC-type aldose transport system ATPase compo	3,410403973	-2,434086198

NC_016856	RefSeq	CDS	4298615	4299658	+	0	ID=cds4722	0	15	STM14_4900	ydeY	putative sugar transport protein	2,131502483	-2,392032191
NC_016856	RefSeq	CDS	4299655	4300656	+	0	ID=cds4723	0	21,5	STM14_4901	ydeZ	putative sugar transport protein	3,126203642	-2,431590384
NC_016856	RefSeq	CDS	4300685	4301707	+	0	ID=cds4724	0	23,75	STM14_4902	yneA	putative sugar transport protein	3,410403973	-2,434086198
NC_016856	RefSeq	CDS	4301736	4302611	+	0	ID=cds4725	0	53,75	STM14_4903	yneB	aldolase	7,673408939	-2,144619715
NC_016856	RefSeq	CDS	4302691	4302984	+	0	ID=cds4726	0	6	STM14_4904	yneC	autoinducer-2 %28AI-2%29 modifying protein LsrG	0,852600993	-1,98056697
NC_016856	RefSeq	CDS	4302994	4303758	+	0	ID=cds4727	0	21	STM14_4905	STM14_4905	ribulose-phosphate 3-epimerase	2,984103476	-2,430148754
NC_016856	RefSeq	CDS	4303850	4304617	-	0	ID=cds4728	0	210,3333333	STM14_4906	tpiA	triosephosphate isomerase	29,84103476	-1,904273548
NC_016856	RefSeq	CDS	4304730	4305326	-	0	ID=cds4729	0	2,5	STM14_4907	yiiQ	hypothetical protein	0,284200331	-1,182723423
NC_016856	RefSeq	CDS	4305427	4305855	+	0	ID=cds4730	0	4,25	STM14_4908	yiiR	putative inner membrane protein	0,568400662	-1,710438693
NC_016856	RefSeq	CDS	4305795	4305962	-	0	ID=cds4731	0	1	STM14_4909	STM14_4909	hypothetical protein	0,142100166	-0,623264422
NC_016856	RefSeq	CDS	4305962	4306708	-	0	ID=cds4732	0	7	STM14_4910	fpr	ferredoxin-NADP reductase	0,994701159	-2,072978628
NC_016856	RefSeq	CDS	4306805	4307905	-	0	ID=cds4733	0	23	STM14_4911	glpX	fructose 1%2C6-bisphosphatase II	3,268303807	-2,432747446
NC_016856	RefSeq	CDS	4307927	4309435	-	0	ID=cds4734	0	49	STM14_4912	glpK	glycerol kinase	6,962908111	-2,178239812
NC_016856	RefSeq	CDS	4309456	4310301	-	0	ID=cds4735	0	17	STM14_4913	glpF	glycerol diffusion	2,415702814	-2,412509737
NC_016856	RefSeq	CDS	4310547	4310666	+	0	ID=cds4736	0	2	STM14_4914	STM14_4914	hypothetical protein	0,284200331	-1,182723423
NC_016856	RefSeq	CDS	4310700	4310939	+	0	ID=cds4737	0	36	STM14_4915	yiiU	putative cytoplasmic protein	5,115605959	-2,281753063
NC_016856	RefSeq	CDS	4311024	4311164	-	0	ID=cds4738	0	2	STM14_4916	STM14_4916	hypothetical protein	0,284200331	-1,182723423
NC_016856	RefSeq	CDS	4311161	4311646	-	0	ID=cds4739	0	85,5	STM14_4917	menG	ribonuclease activity regulator protein RraA	12,22061424	-1,963849549
NC_016856	RefSeq	CDS	4311739	4312668	-	0	ID=cds4740	0	22	STM14_4918	menA	1%2C4-dihydroxy-2-naphthoate octaprenyltransferase	3,126203642	-2,431590384
NC_016856	RefSeq	CDS	4312735	4314066	-	0	ID=cds4741	1	45	STM14_4919	hslU	ATP-dependent protease ATP-binding subunit	8,153829819	-1,174813059
NC_016856	RefSeq	CDS	4314076	4314606	-	0	ID=cds4742	0	5	STM14_4920	hslV	ATP-dependent protease peptidase subunit	0,710500828	-1,864806942
NC_016856	RefSeq	CDS	4314698	4315672	-	0	ID=cds4743	4	13,5	STM14_4921	ftsN	essential cell division protein	9,026691797	1,201892645
NC_016856	RefSeq	CDS	4315766	4316956	-	0	ID=cds4744	0	20,5	STM14_4922	cytR	DNA-binding transcriptional regulator CytR	2,842003311	-2,427810709
NC_016856	RefSeq	CDS	4316946	4319144	-	0	ID=cds4745	0	16	STM14_4923	priA	primosome assembly protein PriA	2,273602649	-2,403729808
NC_016856	RefSeq	CDS	4319348	4319560	+	0	ID=cds4746	0	40	STM14_4924	rpmE	50S ribosomal protein L31	5,684006621	-2,24950002
NC_016856	RefSeq	CDS	4319606	4320268	-	0	ID=cds4747	0	4,5	STM14_4925	STM14_4925	putative outer membrane lipoprotein	0,568400662	-1,710438693
NC_016856	RefSeq	CDS	4320469	4322259	-	0	ID=cds4748	0	2	STM14_4926	STM14_4926	putative arylsulfate sulfotransferase	0,284200331	-1,182723423
NC_016856	RefSeq	CDS	4322716	4323033	-	0	ID=cds4749	0	16	STM14_4927	metJ	transcriptional repressor protein MetJ	2,273602649	-2,403729808
NC_016856	RefSeq	CDS	4323157	4324458	+	0	ID=cds4750	0	10,33333333	STM14_4928	metB	cystathionine gamma-synthase	1,421001655	-2,256999172
NC_016856	RefSeq	CDS	4324461	4326893	+	0	ID=cds4751	0	35,5	STM14_4929	metL	bifunctional aspartate kinase II%2Fhomoserine dehydroge	5,115605959	-2,281753063
NC_016856	RefSeq	CDS	4327009	4327866	-	0	ID=cds4752	0	0	STM14_4930	STM14_4930	putative inner membrane protein	0	NA
NC_016856	RefSeq	CDS	4327869	4328867	-	0	ID=cds4753	0	1	STM14_4931	STM14_4931	hypothetical protein	0,142100166	-0,623264422
NC_016856	RefSeq	CDS	4329102	4330226	-	0	ID=cds4754	0	9	STM14_4932	STM14_4932	putative cytoplasmic protein	1,27890149	-2,207741391
NC_016856	RefSeq	CDS	4330362	4331918	+	0	ID=cds4755	0	3,833333333	STM14_4933	STM14_4933	putative 5%27-nucleotidase%2F2%27%2C3%27-cyclic pho	0,568400662	-1,710438693
NC_016856	RefSeq	CDS	4332008	4332127	-	0	ID=cds4756	0	0	STM14_4934	STM14_4934	hypothetical protein	0	NA
NC_016856	RefSeq	CDS	4332113	4333003	+	0	ID=cds4757	0	1,5	STM14_4935	metF	5%2C10-methylenetetrahydrofolate reductase	0,284200331	-1,182723423
NC_016856	RefSeq	CDS	4333168	4335348	+	0	ID=cds4758	1	65,5	STM14_4936	katG	hydroperoxidase	11,1379333	-1,549301265
NC_016856	RefSeq	CDS	4335406	4336029	-	0	ID=cds4759	0	2	STM14_4937	yijF	hypothetical protein	0,284200331	-1,182723423
NC_016856	RefSeq	CDS	4336017	4336178	+	0	ID=cds4760	0	0	STM14_4938	STM14_4938	hypothetical protein	0	NA
NC_016856	RefSeq	CDS	4336288	4337391	-	0	ID=cds4761	0	28	STM14_4939	gldA_2	glycerol dehydrogenase	3,978804635	-2,448045729
NC_016856	RefSeq	CDS	4337403	4338065	-	0	ID=cds4762	0	19,5	STM14_4940	talC	fructose-6-phosphate aldolase	2,842003311	-2,427810709
NC_016856	RefSeq	CDS	4338076	4340577	-	0	ID=cds4763	0	14	STM14_4941	ptsA	PEP-protein phosphotransferase	1,989402318	-2,376535557
NC_016856	RefSeq	CDS	4340772	4340927	-	0	ID=cds4764	0	0	STM14_4943	STM14_4943	hypothetical protein	0	NA
NC_016856	RefSeq	CDS	4340886	4341965	+	0	ID=cds4765	0	2,5	STM14_4944	frwC	putative fructose-like permease EIIC subunit 2	0,284200331	-1,182723423
NC_016856	RefSeq	CDS	4341980	4342300	+	0	ID=cds4766	0	5	STM14_4945	frwB	putative fructose-like phosphotransferase EIIB subunit 2	0,710500828	-1,864806942
NC_016856	RefSeq	CDS	4342399	4344696	+	0	ID=cds4767	0	19	STM14_4946	pflD	putative formate acetyltransferase 2	2,699903145	-2,42419778
NC_016856	RefSeq	CDS	4344662	4345540	+	0	ID=cds4768	0	7,5	STM14_4947	pflC	pyruvate formate lyase II activase	1,136801324	-2,147403465
NC_016856	RefSeq	CDS	4345542	4345895	+	0	ID=cds4769	0	5,5	STM14_4948	frwD	putative fructose-like phosphotransferase EIIB subunit 3	0,852600993	-1,98056697
NC_016856	RefSeq	CDS	4345882	4346733	-	0	ID=cds4770	0	10,5	STM14_4949	yijO	putative regulatory protein	1,421001655	-2,256999172
NC_016856	RefSeq	CDS	4346732	4346872	+	0	ID=cds4771	0	0	STM14_4950	STM14_4950	hypothetical protein	0	NA
NC_016856	RefSeq	CDS	4346784	4346900	+	0	ID=cds4772	0	0	STM14_4951	STM14_4951	hypothetical protein	0	NA
NC_016856	RefSeq	CDS	4346894	4348627	-	0	ID=cds4773	0	83	STM14_4952	yijP	hypothetical protein	11,79431374	-1,978168022
NC_016856	RefSeq	CDS	4348703	4348810	+	0	ID=cds4774	0	0	STM14_4953	STM14_4953	hypothetical protein	0	NA
NC_016856	RefSeq	CDS	4348838	4351489	-	0	ID=cds4775	0	53	STM14_4954	ppc	phosphoenolpyruvate carboxylase	7,531308773	-2,151665531
NC_016856	RefSeq	CDS	4351858	4353009	-	0	ID=cds4776	0	14,5	STM14_4955	argE	acetylornithine deacetylase	1,989402318	-2,376535557
NC_016856	RefSeq	CDS	4353098	4354102	+	0	ID=cds4777	0	2,5	STM14_4956	argC	N-acetyl-gamma-glutamyl-phosphate reductase	0,284200331	-1,182723423
NC_016856	RefSeq	CDS	4354113	4354886	+	0	ID=cds4778	0	4	STM14_4957	argB	acetylglutamate kinase	0,568400662	-1,710438693
NC_016856	RefSeq	CDS	4355004	4356380	+	0	ID=cds4779	0	11	STM14_4958	argH	argininosuccinate lyase	1,563101821	-2,297202414
NC_016856	RefSeq	CDS	4356665	4357582	+	0	ID=cds4780	0	24	STM14_4959	oxyR	DNA-binding transcriptional regulator OxyR	3,410403973	-2,434086198
NC_016856	RefSeq	CDS	4357565	4358965	-	0	ID=cds4781	0	10,5	STM14_4960	udhA	soluble pyridine nucleotide transhydrogenase	1,421001655	-2,256999172
NC_016856	RefSeq	CDS	4358974	4359162	-	0	ID=cds4782	0	0	STM14_4961	STM14_4961	hypothetical protein	0	NA
NC_016856	RefSeq	CDS	4359164	4359799	+	0	ID=cds4783	0	17,5	STM14_4962	yijC	DNA-binding transcriptional repressor FabR	2,55780298	-2,419166771
NC_016856	RefSeq	CDS	4359815	4360174	+	0	ID=cds4784	0	13	STM14_4963	yijD	hypothetical protein	1,847302152	-2,356210635
NC_016856	RefSeq	CDS	4360221	4361321	-	0	ID=cds4785	0	3	STM14_4964	trmA	tRNA %28uracil-5-%29-methyltransferase	0,426300497	-1,498211557
NC_016856	RefSeq	CDS	4361695	4363539	+	0	ID=cds4786	0	26,5	STM14_4965	btuB	vitamin B12%2Fcobalamin outer membrane transporter	3,694604304	-2,438835558
NC_016856	RefSeq	CDS	4363484	4364335	+	0	ID=cds4787	0	21,5	STM14_4966	murI	glutamate racemase	3,126203642	-2,431590384
NC_016856	RefSeq	CDS	4366598	4366714	+	0	ID=cds4788	8,873015873	155,5507937	STM14_4970	STM14_4970	hypothetical protein	38,00152715	-0,341111741
NC_016856	RefSeq	CDS	4370253	4371281	+	0	ID=cds4789	0	19,5	STM14_4973	murB	UDP-N-acetylenolpyruvoylglucosamine reductase	2,842003311	-2,427810709
NC_016856	RefSeq	CDS	4371278	4372240	+	0	ID=cds4790	0	13	STM14_4974	birA	biotin--protein ligase	1,847302152	-2,356210635
NC_016856	RefSeq	CDS	4372275	4373225	-	0	ID=cds4791	0	7,5	STM14_4975	coaA	pantothenate kinase	1,136801324	-2,147403465
NC_016856	RefSeq	CDS	4373434	4373607	-	0	ID=cds4792	0	0	STM14_4977	STM14_4977	putative cytoplasmic protein	0	NA
NC_016856	RefSeq	CDS	4374184	4375368	+	0	ID=cds4793	2	532,1666667	STM14_4982	tuf_2	elongation factor Tu	79,11593281	-2,924814833
NC_016856	RefSeq	CDS	4375382	4375510	+	0	ID=cds4794	0	34,5	STM14_4983	STM14_4983	hypothetical protein	4,831405628	-2,523251848
NC_016856	RefSeq	CDS	4375598	4375981	+	0	ID=cds4795	0	68,25	STM14_4984	secE	preprotein translocase subunit SecE	9,662811256	-2,058244678
NC_016856	RefSeq	CDS	4375983	4376528	+	0	ID=cds4796	7	37,25	STM14_4985	nusG	transcription antitermination protein NusG	17,57296271	0,855827501
NC_016856	RefSeq	CDS	4376686	4377114	+	0	ID=cds4797	0	110	STM14_4986	rplK	50S ribosomal protein L11	15,63101821	-1,86872757
NC_016856	RefSeq	CDS	4377118	4377822	+	0	ID=cds4798	0	180,5	STM14_4987	rplA	50S ribosomal protein L1	25,5780298	-1,892233448
NC_016856	RefSeq	CDS	4378242	4378739	+	0	ID=cds4799	0	198,8333333	STM14_4988	rplJ	50S ribosomal protein L10	28,27793294	-1,900235164
NC_016856	RefSeq	CDS	4378806	4379171	+	0	ID=cds4800	0	224,8333333	STM14_4989	rplL	50S ribosomal protein L7%2FL12	31,97253725	-1,909205776
NC_016856	RefSeq	CDS	4379489	4383517	+	0	ID=cds4801	0	556,1666667	STM14_4990	rpoB	DNA-directed RNA polymerase subunit beta	79,00769204	-1,953230131
NC_016856	RefSeq	CDS	4383594	4387817	+	0	ID=cds4802	1	389,3333333	STM14_4991	rpoC	DNA-directed RNA polymerase subunit beta%27	57,03628676	-3,13926313
NC_016856	RefSeq	CDS	4387859	4388182	+	0	ID=cds4803	0	1,5	STM14_4992	STM14_4992	putative inner membrane protein	0,284200331	-1,182723423
NC_016856	RefSeq	CDS	4388188	4388532	-	0	ID=cds4804	0	0	STM14_4993	STM14_4993	putative cytoplasmic protein	0	NA
NC_016856	RefSeq	CDS	4388486	4388623	-	0	ID=cds4805	0	0	STM14_4994	STM14_4994	hypothetical protein	0	NA
NC_016856	RefSeq	CDS	4388644	4388847	+	0	ID=cds4806	0	0	STM14_4995	STM14_4995	hypothetical protein	0	NA
NC_016856	RefSeq	CDS	4388931	4389941	+	0	ID=cds4807	0	1,5	STM14_4996	STM14_4996	putative cytoplasmic protein	0,284200331	-1,182723423
NC_016856	RefSeq	CDS	4389931	4390140	+	0	ID=cds4808	0	0,5	STM14_4997	STM14_4997	hypothetical protein	0	NA
NC_016856	RefSeq	CDS	4390264	4390452	+	0	ID=cds4809	0	0	STM14_4999	STM14_4999	putative cytoplasmic protein	0	NA
NC_016856	RefSeq	CDS	4390603	4391736	-	0	ID=cds4810	0	1,5	STM14_5000	thiH	thiamine biosynthesis protein ThiH	0,284200331	-1,182723423
NC_016856	RefSeq	CDS	4391733	4392503	-	0	ID=cds4811	0	1	STM14_5001	thiG	thiazole synthase	0,142100166	-0,623264422
NC_016856	RefSeq	CDS	4392505	4392705	-	0	ID=cds4812	0	0	STM14_5002	STM14_5002	sulfur carrier protein ThiS	0	NA
NC_016856	RefSeq	CDS	4392686	4393444	-	0	ID=cds4813	0	0	STM14_5003	thiF	thiamine biosynthesis protein ThiF	0	NA
NC_016856	RefSeq	CDS	4393437	4394072	-	0	ID=cds4814	0	0	STM14_5004	thiE	thiamine-phosphate pyrophosphorylase	0	NA
NC_016856	RefSeq	CDS	4394072	4395967	-	0	ID=cds4815	0	1	STM14_5005	thiC	thiamine biosynthesis protein ThiC	0,142100166	-0,623264422
NC_016856	RefSeq	CDS	4396331	4396819	-	0	ID=cds4816	0	1	STM14_5006	rsd	anti-RNA polymerase sigma 70 factor	0,142100166	-0,623264422
NC_016856	RefSeq	CDS	4396912	4397685	+	0	ID=cds4817	0	10,5	STM14_5007	nudC	NADH pyrophosphatase	1,421001655	-2,256999172
NC_016856	RefSeq	CDS	4397726	4398790	+	0	ID=cds4818	0	45,5	STM14_5008	hemE	uroporphyrinogen decarboxylase	6,536607615	-2,201017198
NC_016856	RefSeq	CDS	4398800	4399471	+	0	ID=cds4819	0	11,5	STM14_5009	nfi	endonuclease V	1,705201986	-2,329925387
NC_016856	RefSeq	CDS	4399513	4400103	+	0	ID=cds4820	0	39,5	STM14_5010	yjaG	putative cytoplasmic protein	5,684006621	-2,24950002
NC_016856	RefSeq	CDS	4400290	4400562	+	0	ID=cds4821	0	238,75	STM14_5011	hupA	transcriptional regulator HU subunit alpha	33,96193956	-1,913302279
NC_016856	RefSeq	CDS	4400574	4401266	+	0	ID=cds4822	0	23,08333333	STM14_5012	yjaH	putative inner membrane protein	3,268303807	-2,432747446
NC_016856	RefSeq	CDS	4401308	4401763	-	0	ID=cds4823	0	3,5	STM14_5013	zraP	zinc resistance protein	0,568400662	-1,710438693
NC_016856	RefSeq	CDS	4401933	4403414	+	0	ID=cds4824	0	14,83333333	STM14_5014	hydH	sensor protein ZraS	2,131502483	-2,392032191
NC_016856	RefSeq	CDS	4403420	4404745	+	0	ID=cds4825	0	19	STM14_5015	hydG	transcriptional regulatory protein ZraR	2,699903145	-2,42419778
NC_016856	RefSeq	CDS	4404742	4406031	-	0	ID=cds4826	0	1,5	STM14_5016	purD	phosphoribosylamine--glycine ligase	0,284200331	-1,182723423
NC_016856	RefSeq	CDS	4406043	4407632	-	0	ID=cds4827	4	10,5	STM14_5017	purH	bifunctional phosphoribosylaminoimidazolecarboxamide f	8,458291135	1,501349535
NC_016856	RefSeq	CDS	4407683	4407805	+	0	ID=cds4828	0	0	STM14_5018	STM14_5018	hypothetical protein	0	NA
NC_016856	RefSeq	CDS	4407796	4407918	-	0	ID=cds4829	0	1	STM14_5019	STM14_5019	hypothetical protein	0,142100166	-0,623264422
NC_016856	RefSeq	CDS	4407851	4407991	+	0	ID=cds4830	0,333333333	15	STM14_5020	STM14_5020	hypothetical protein	2,131502483	-2,392032191
NC_016856	RefSeq	CDS	4413499	4413630	-	0	ID=cds4831	0	1	STM14_5025	STM14_5025	hypothetical protein	0,142100166	-0,623264422
NC_016856	RefSeq	CDS	4413565	4413795	+	0	ID=cds4832	0	0	STM14_5026	STM14_5026	hypothetical protein	0	NA
NC_016856	RefSeq	CDS	4413786	4414223	-	0	ID=cds4833	0	9	STM14_5027	yjaB	hypothetical protein	1,27890149	-2,207741391
NC_016856	RefSeq	CDS	4414380	4415309	+	0	ID=cds4834	0	5,5	STM14_5028	metA	homoserine O-succinyltransferase	0,852600993	-1,98056697
NC_016856	RefSeq	CDS	4415578	4417179	+	0	ID=cds4835	0	12,5	STM14_5029	aceB	malate synthase	1,705201986	-2,329925387
NC_016856	RefSeq	CDS	4417211	4418515	+	0	ID=cds4836	0	2,5	STM14_5030	aceA	isocitrate lyase	0,284200331	-1,182723423
NC_016856	RefSeq	CDS	4418519	4418641	+	0	ID=cds4837	0	0	STM14_5031	STM14_5031	hypothetical protein	0	NA
NC_016856	RefSeq	CDS	4418617	4420368	+	0	ID=cds4838	3	5,5	STM14_5032	aceK	bifunctional isocitrate dehydrogenase kinase%2Fphospha	6,130568103	1,664704357
NC_016856	RefSeq	CDS	4420332	4420739	-	0	ID=cds4839	0	3,5	STM14_5033	STM14_5033	putative cytoplasmic protein	0,568400662	-1,710438693
NC_016856	RefSeq	CDS	4420750	4421574	-	0	ID=cds4840	0	6,5	STM14_5034	iclR	IclR family transcriptional regulator	0,852600993	-1,98056697
NC_016856	RefSeq	CDS	4421773	4425561	+	0	ID=cds4841	0	153,5	STM14_5035	metH	B12-dependent methionine synthase	21,88342549	-1,878506335
NC_016856	RefSeq	CDS	4425828	4427459	+	0	ID=cds4842	1	18	STM14_5036	yjbB	putative transport protein	4,31712535	-0,335890347
NC_016856	RefSeq	CDS	4427535	4428224	-	0	ID=cds4843	0	24,5	STM14_5037	pepE	peptidase E	3,410403973	-2,434086198
NC_016856	RefSeq	CDS	4428296	4428397	+	0	ID=cds4844	0	0	STM14_5038	STM14_5038	putative cytoplasmic protein	0	NA
NC_016856	RefSeq	CDS	4428432	4428971	+	0	ID=cds4845	0	5,333333333	STM14_5039	STM14_5039	putative cytoplasmic protein	0,710500828	-1,864806942

NC_01685(	RefSeq	CDS	4429018	4429887	+	0	ID=cds4846	0	22,5	STM14_5040	yjbC	23S rRNA pseudouridine synthase F	3,126203642	-2,431590384
NC_01685(	RefSeq	CDS	4429884	4430156	-	0	ID=cds4847	0	9,5	STM14_5041	yjbD	hypothetical protein	1,421001655	-2,256999172
NC_01685(	RefSeq	CDS	4430254	4430427	-	0	ID=cds4848	0	1	STM14_5042	STM14_5042	putative Na%2B-dependent transporter	0,142100166	-0,623264422
NC_01685(	RefSeq	CDS	4431313	4431468	+	0	ID=cds4849	0	0	STM14_5044	STM14_5044	hypothetical protein	0	NA
NC_01685(	RefSeq	CDS	4431457	4432185	-	0	ID=cds4850	0	4	STM14_5045	STM14_5045	putative cytoplasmic protein	0,568400662	-1,710438693
NC_01685(	RefSeq	CDS	4432382	4432672	-	0	ID=cds4851	0	0	STM14_5046	STM14_5046	putative inner membrane protein	0	NA
NC_01685(	RefSeq	CDS	4432921	4433376	-	0	ID=cds4852	0	0	STM14_5047	STM14_5047	putative cytoplasmic protein	0	NA
NC_01685(	RefSeq	CDS	4433373	4433978	-	0	ID=cds4853	0	0	STM14_5048	STM14_5048	putative cytoplasmic protein	0	NA
NC_01685(	RefSeq	CDS	4433983	4435728	-	0	ID=cds4854	0	0	STM14_5049	STM14_5049	putative phage tail fiber protein H	0	NA
NC_01685(	RefSeq	CDS	4435731	4436363	-	0	ID=cds4855	0	0	STM14_5050	STM14_5050	putative phage tail protein	0	NA
NC_01685(	RefSeq	CDS	4436356	4437471	-	0	ID=cds4856	0	1	STM14_5051	STM14_5051	putative phage baseplate protein	0,142100166	-0,623264422
NC_01685(	RefSeq	CDS	4437462	4437821	-	0	ID=cds4857	0	0	STM14_5052	STM14_5052	putative phage baseplate protein	0	NA
NC_01685(	RefSeq	CDS	4437882	4438004	+	0	ID=cds4858	0	0	STM14_5053	STM14_5053	hypothetical protein	0	NA
NC_01685(	RefSeq	CDS	4437985	4439532	-	0	ID=cds4859	0	5,75	STM14_5054	STM14_5054	putative inner membrane protein	0,852600993	-1,98056697
NC_01685(	RefSeq	CDS	4439532	4440461	-	0	ID=cds4860	0	5,25	STM14_5055	STM14_5055	putative phage glycosyltransferase	0,710500828	-1,864806942
NC_01685(	RefSeq	CDS	4440458	4440820	-	0	ID=cds4861	0	0	STM14_5056	STM14_5056	putative phage glucose translocase	0	NA
NC_01685(	RefSeq	CDS	4441014	4441139	-	0	ID=cds4862	0	0	STM14_5057	STM14_5057	hypothetical protein	0	NA
NC_01685(	RefSeq	CDS	4441148	4441870	-	0	ID=cds4863	0	1	STM14_5058	STM14_5058	putative phage baseplate component	0,142100166	-0,623264422
NC_01685(	RefSeq	CDS	4441880	4442923	-	0	ID=cds4864	0	1,5	STM14_5059	STM14_5059	putative cytoplasmic protein	0,284200331	-1,182723423
NC_01685(	RefSeq	CDS	4442911	4443120	-	0	ID=cds4865	0	0	STM14_5060	STM14_5060	putative inner membrane protein	0	NA
NC_01685(	RefSeq	CDS	4443120	4444073	-	0	ID=cds4866	0	1	STM14_5061	STM14_5061	putative methyl-accepting chemotaxis protein	0,142100166	-0,623264422
NC_01685(	RefSeq	CDS	4444073	4446427	-	0	ID=cds4867	0	0	STM14_5062	STM14_5062	putative phage tail protein	0	NA
NC_01685(	RefSeq	CDS	4446524	4446652	-	0	ID=cds4868	0	0,5	STM14_5063	STM14_5063	hypothetical protein	0	NA
NC_01685(	RefSeq	CDS	4446981	4447505	-	0	ID=cds4869	0	0	STM14_5065	STM14_5065	putative phage tail core protein	0	NA
NC_01685(	RefSeq	CDS	4447505	4448932	-	0	ID=cds4870	0	0	STM14_5066	STM14_5066	putative phage tail sheath protein	0	NA
NC_01685(	RefSeq	CDS	4448922	4449119	-	0	ID=cds4871	0	0	STM14_5067	STM14_5067	putative cytoplasmic protein	0	NA
NC_01685(	RefSeq	CDS	4449116	4449571	-	0	ID=cds4872	0	1	STM14_5068	STM14_5068	putative cytoplasmic protein	0,142100166	-0,623264422
NC_01685(	RefSeq	CDS	4449731	4450045	-	0	ID=cds4873	0	0	STM14_5069	STM14_5069	putative inner membrane protein	0	NA
NC_01685(	RefSeq	CDS	4450058	4450663	-	0	ID=cds4874	0	0	STM14_5070	STM14_5070	putative soluble lytic murein transglycosylase	0	NA
NC_01685(	RefSeq	CDS	4450666	4450953	-	0	ID=cds4875	0	0	STM14_5071	STM14_5071	putative inner membrane protein	0	NA
NC_01685(	RefSeq	CDS	4451435	4451575	+	0	ID=cds4876	0	0	STM14_5072	STM14_5072	hypothetical protein	0	NA
NC_01685(	RefSeq	CDS	4451529	4451876	+	0	ID=cds4877	1,5	0	STM14_5073	STM14_5073	putative cytoplasmic protein	3,51864474	1,925445802
NC_01685(	RefSeq	CDS	4452009	4453358	-	0	ID=cds4878	0	7,5	STM14_5074	lysC	aspartate kinase III	1,136801324	-2,147403465
NC_01685(	RefSeq	CDS	4453703	4455352	+	0	ID=cds4879	0	31,5	STM14_5075	pgi	glucose-6-phosphate isomerase	4,547205297	-2,489878815
NC_01685(	RefSeq	CDS	4455796	4456038	+	0	ID=cds4880	0	0	STM14_5076	yjbE	putative outer membrane protein	0	NA
NC_01685(	RefSeq	CDS	4456072	4456740	+	0	ID=cds4881	0	1	STM14_5077	yjbF	putative outer membrane lipoprotein	0,142100166	-0,623264422
NC_01685(	RefSeq	CDS	4456737	4457474	+	0	ID=cds4882	0	1	STM14_5078	yjbG	hypothetical protein	0,142100166	-0,623264422
NC_01685(	RefSeq	CDS	4457474	4459570	+	0	ID=cds4883	0	7	STM14_5079	yjbH	putative outer membrane lipoprotein	0,994701159	-2,072978628
NC_01685(	RefSeq	CDS	4459713	4460123	+	0	ID=cds4884	0	9,5	STM14_5080	yjbA	phosphate-starvation-inducible protein PsiE	1,421001655	-2,256999172
NC_01685(	RefSeq	CDS	4460289	4461179	-	0	ID=cds4885	0	10,5	STM14_5081	malG	maltose transporter permease	1,421001655	-2,256999172
NC_01685(	RefSeq	CDS	4461194	4462738	-	0	ID=cds4886	0	14,83333333	STM14_5082	malF	maltose transporter membrane protein	2,131502483	-2,392032191
NC_01685(	RefSeq	CDS	4462681	4462857	-	0	ID=cds4887	0	3,333333333	STM14_5083	STM14_5083	hypothetical protein	0,426300497	-1,498211557
NC_01685(	RefSeq	CDS	4462737	4462877	+	0	ID=cds4888	0	0	STM14_5084	STM14_5084	hypothetical protein	0	NA
NC_01685(	RefSeq	CDS	4462870	4464297	-	0	ID=cds4889	0	75,5	STM14_5085	malE	maltose ABC transporter periplasmic protein	10,79961258	-2,01367454
NC_01685(	RefSeq	CDS	4464422	4465531	+	0	ID=cds4890	0	23	STM14_5086	malK	maltose%2Fmaltodextrin transporter ATP-binding protein	3,268303807	-2,432747446
NC_01685(	RefSeq	CDS	4465620	4466978	+	0	ID=cds4891	0	75,75	STM14_5087	lamB	maltoporin	10,79961258	-2,01367454
NC_01685(	RefSeq	CDS	4466972	4467136	+	0	ID=cds4892	0	10,25	STM14_5088	STM14_5088	hypothetical protein	1,421001655	-2,256999172
NC_01685(	RefSeq	CDS	4467142	4468059	+	0	ID=cds4893	0	23,5	STM14_5089	malM	maltose regulon periplasmic protein	3,410403973	-2,434086198
NC_01685(	RefSeq	CDS	4468240	4468737	+	0	ID=cds4894	5	11	STM14_5090	ubiC	chorismate pyruvate lyase	10,35971367	1,647620804
NC_01685(	RefSeq	CDS	4468751	4469623	+	0	ID=cds4895	0	5,5	STM14_5091	ubiA	4-hydroxybenzoate octaprenyltransferase	0,852600993	-1,98056697
NC_01685(	RefSeq	CDS	4469722	4472142	-	0	ID=cds4896	0	106,5	STM14_5092	plsB	glycerol-3-phosphate acyltransferase	15,06261755	-1,883514636
NC_01685(	RefSeq	CDS	4472313	4472681	+	0	ID=cds4897	0	8	STM14_5093	dgkA	diacylglycerol kinase	1,136801324	-2,147403465
NC_01685(	RefSeq	CDS	4472790	4473398	+	0	ID=cds4898	9	106	STM14_5094	lexA	LexA repressor	30,89651888	0,050515892
NC_01685(	RefSeq	CDS	4473577	4474902	+	0	ID=cds4899	1	20,5	STM14_5095	dinF	DNA-damage-inducible SOS response protein	4,601325681	-0,429413798
NC_01685(	RefSeq	CDS	4474899	4475012	+	0	ID=cds4900	0	6,5	STM14_5096	STM14_5096	putative cytoplasmic protein	0,852600993	-1,98056697
NC_01685(	RefSeq	CDS	4475031	4475243	+	0	ID=cds4901	0	19	STM14_5097	yjbJ	putative stress-response protein	2,699903145	-2,42419778
NC_01685(	RefSeq	CDS	4475342	4475857	-	0	ID=cds4902	0	26	STM14_5098	zur	zinc uptake transcriptional repressor	3,694604304	-2,438835558
NC_01685(	RefSeq	CDS	4476104	4477414	+	0	ID=cds4903	0	4,5	STM14_5099	STM14_5099	conjugative transfer protein	0,568400662	-1,710438693
NC_01685(	RefSeq	CDS	4477502	4478500	+	0	ID=cds4904	0	14	STM14_5100	yjbN	tRNA-dihydrouridine synthase A	1,989402318	-2,376535557
NC_01685(	RefSeq	CDS	4478537	4478704	+	0	ID=cds4905	0	2,5	STM14_5101	STM14_5101	hypothetical protein	0,284200331	-1,182723423
NC_01685(	RefSeq	CDS	4478668	4478910	+	0	ID=cds4906	0	0	STM14_5102	pspG	phage shock protein G	0	NA
NC_01685(	RefSeq	CDS	4479085	4480068	-	0	ID=cds4907	0	5,5	STM14_5103	qor	quinone oxidoreductase	0,852600993	-1,98056697
NC_01685(	RefSeq	CDS	4480133	4481548	+	0	ID=cds4908	0	34,5	STM14_5104	dnaB	replicative DNA helicase	4,831405628	-2,523251848
NC_01685(	RefSeq	CDS	4481580	4482659	+	0	ID=cds4909	0	11,5	STM14_5105	alr	alanine racemase	1,705201986	-2,329925387
NC_01685(	RefSeq	CDS	4482845	4484038	+	0	ID=cds4910	0	10,33333333	STM14_5106	tyrB	aromatic amino acid aminotransferase	1,421001655	-2,256999172
NC_01685(	RefSeq	CDS	4484225	4484938	+	0	ID=cds4911	0	21,5	STM14_5107	aphA	acid phosphatase%2Fphosphotransferase	3,126203642	-2,431590384
NC_01685(	RefSeq	CDS	4485067	4485483	+	0	ID=cds4912	0	19	STM14_5108	yjbQ	putative cytoplasmic protein	2,699903145	-2,42419778
NC_01685(	RefSeq	CDS	4485486	4485842	+	0	ID=cds4913	0	9,833333333	STM14_5109	yjbR	putative cytoplasmic protein	1,421001655	-2,256999172
NC_01685(	RefSeq	CDS	4485843	4486181	-	0	ID=cds4914	0	0	STM14_5110	STM14_5110	putative inner membrane protein	0	NA
NC_01685(	RefSeq	CDS	4486168	4486623	-	0	ID=cds4915	0	0	STM14_5111	STM14_5111	putative outer membrane lipoprotein	0	NA
NC_01685(	RefSeq	CDS	4486755	4489580	-	0	ID=cds4916	1	46,83333333	STM14_5112	uvrA	excinuclease ABC subunit A	8,43803015	-1,216263872
NC_01685(	RefSeq	CDS	4489545	4489661	+	0	ID=cds4917	0	0,25	STM14_5113	STM14_5113	putative cytoplasmic protein	0	NA
NC_01685(	RefSeq	CDS	4489828	4490358	+	0	ID=cds4918	0,5	31	STM14_5114	ssb	single-strand DNA-binding protein	4,405105132	-2,475774913
NC_01685(	RefSeq	CDS	4490374	4490565	-	0	ID=cds4919	0	0	STM14_5115	STM14_5115	hypothetical protein	0	NA
NC_01685(	RefSeq	CDS	4490568	4490723	+	0	ID=cds4920	0	0	STM14_5116	STM14_5116	hypothetical protein	0	NA
NC_01685(	RefSeq	CDS	4491399	4492031	+	0	ID=cds4921	0	20,5	STM14_5117	STM14_5117	hypothetical protein	2,842003311	-2,427810709
NC_01685(	RefSeq	CDS	4492028	4493416	+	0	ID=cds4922	0	19	STM14_5118	STM14_5118	putative methyl-accepting chemotaxis protein	2,699903145	-2,42419778
NC_01685(	RefSeq	CDS	4493406	4494725	+	0	ID=cds4923	0	12	STM14_5119	STM14_5119	putative ABC exporter outer membrane component	1,705201986	-2,329925387
NC_01685(	RefSeq	CDS	4494722	4495999	+	0	ID=cds4924	0	20,5	STM14_5120	STM14_5120	cation efflux pump	2,842003311	-2,427810709
NC_01685(	RefSeq	CDS	4496016	4512695	+	0	ID=cds4925	0	491,5	STM14_5121	STM14_5121	putative inner membrane protein	69,91328144	-1,949107066
NC_01685(	RefSeq	CDS	4512735	4514801	+	0	ID=cds4926	0	21	STM14_5122	STM14_5122	putative ABC-type bacteriocin%2Flantibiotic exporter	2,984103476	-2,430148754
NC_01685(	RefSeq	CDS	4515079	4515360	-	0	ID=cds4927	0	0	STM14_5123	yjcB	putative inner membrane protein	0	NA
NC_01685(	RefSeq	CDS	4515420	4515557	+	0	ID=cds4928	0	0	STM14_5124	STM14_5124	hypothetical protein	0	NA
NC_01685(	RefSeq	CDS	4515741	4515845	-	0	ID=cds4929	0	0	STM14_5125	STM14_5125	hypothetical protein	0	NA
NC_01685(	RefSeq	CDS	4515923	4517524	+	0	ID=cds4930	0	20	STM14_5126	yjcC	diguanylate cyclase	2,842003311	-2,427810709
NC_01685(	RefSeq	CDS	4517512	4517835	-	0	ID=cds4931	0	0	STM14_5127	soxS	DNA-binding transcriptional regulator SoxS	0	NA
NC_01685(	RefSeq	CDS	4517922	4518380	+	0	ID=cds4932	0	1	STM14_5128	soxR	redox-sensing transcriptional activator	0,142100166	-0,623264422
NC_01685(	RefSeq	CDS	4518503	4518631	-	0	ID=cds4933	0	4,5	STM14_5129	STM14_5129	hypothetical protein	0,568400662	-1,710438693
NC_01685(	RefSeq	CDS	4518673	4519341	+	0	ID=cds4934	0	0	STM14_5130	STM14_5130	putative glutathione S-transferase	0	NA
NC_01685(	RefSeq	CDS	4519322	4519456	-	0	ID=cds4935	0	0	STM14_5131	STM14_5131	hypothetical protein	0	NA
NC_01685(	RefSeq	CDS	4519489	4519611	-	0	ID=cds4936	0	0	STM14_5132	STM14_5132	hypothetical protein	0	NA
NC_01685(	RefSeq	CDS	4519688	4521037	+	0	ID=cds4937	0	6	STM14_5133	yjcD	hypothetical protein	0,852600993	-1,98056697
NC_01685(	RefSeq	CDS	4521030	4521188	-	0	ID=cds4938	0	0	STM14_5134	STM14_5134	hypothetical protein	0	NA
NC_01685(	RefSeq	CDS	4521187	4522833	+	0	ID=cds4939	0	10	STM14_5135	yjcE	Na%2FH transport protein	1,421001655	-2,256999172
NC_01685(	RefSeq	CDS	4522928	4523815	-	0	ID=cds4940	0	3,5	STM14_5136	STM14_5136	putative transcriptional regulator	0,568400662	-1,710438693
NC_01685(	RefSeq	CDS	4523814	4524329	+	0	ID=cds4941	0	1	STM14_5137	STM14_5137	LrgA family protein	0,142100166	-0,623264422
NC_01685(	RefSeq	CDS	4524322	4525011	+	0	ID=cds4942	1,5	2,5	STM14_5138	STM14_5138	putative inner membrane protein	3,802845071	2,200856121
NC_01685(	RefSeq	CDS	4525050	4526699	-	0	ID=cds4943	0	1	STM14_5139	actP	acetate permease	0,142100166	-0,623264422
NC_01685(	RefSeq	CDS	4526696	4527085	-	0	ID=cds4944	0	0	STM14_5140	yjcH	putative inner membrane protein	0	NA
NC_01685(	RefSeq	CDS	4527256	4529214	-	0	ID=cds4945	0	5	STM14_5141	acs	acetyl-CoA synthetase	0,710500828	-1,864806942
NC_01685(	RefSeq	CDS	4529401	4529547	+	0	ID=cds4946	0	0,5	STM14_5142	STM14_5142	putative cytoplasmic protein	0	NA
NC_01685(	RefSeq	CDS	4529646	4531082	+	0	ID=cds4947	0	20	STM14_5143	nrfA	cytochrome c nitrite reductase	2,842003311	-2,427810709
NC_01685(	RefSeq	CDS	4531194	4531760	+	0	ID=cds4948	0	10	STM14_5144	nrfB	cytochrome c-type protein NrfB	1,421001655	-2,256999172
NC_01685(	RefSeq	CDS	4531757	4532428	+	0	ID=cds4949	0	5	STM14_5145	nrfC	putative formate-dependent nitrite reductase	0,710500828	-1,864806942
NC_01685(	RefSeq	CDS	4532425	4533381	+	0	ID=cds4950	0	13,5	STM14_5146	nrfD	putative formate-dependent nitrate reductase	1,989402318	-2,376535557
NC_01685(	RefSeq	CDS	4533374	4535596	+	0	ID=cds4951	0	12	STM14_5147	nrfE	formate-dependent nitrite reductase	1,705201986	-2,329925387
NC_01685(	RefSeq	CDS	4535593	4536213	+	0	ID=cds4952	0	2	STM14_5148	nrfG	formate-dependent nitrite reductase complex subunit Nrf	0,284200331	-1,182723423
NC_01685(	RefSeq	CDS	4536353	4536481	-	0	ID=cds4953	0	0	STM14_5149	STM14_5149	hypothetical protein	0	NA
NC_01685(	RefSeq	CDS	4536377	4536649	+	0	ID=cds4954	0	1,5	STM14_5150	STM14_5150	hypothetical protein	0,284200331	-1,182723423
NC_01685(	RefSeq	CDS	4536558	4537868	+	0	ID=cds4955	0	17	STM14_5151	gltP	glutamate%2Faspartate:proton symporter	2,415702814	-2,412509737
NC_01685(	RefSeq	CDS	4537849	4538022	+	0	ID=cds4956	0	0,5	STM14_5152	STM14_5152	hypothetical protein	0	NA
NC_01685(	RefSeq	CDS	4538026	4538715	-	0	ID=cds4957	0	8,5	STM14_5153	yjcO	hypothetical protein	1,136801324	-2,147403465
NC_01685(	RefSeq	CDS	4538728	4538883	-	0	ID=cds4958	0	1	STM14_5154	STM14_5154	hypothetical protein	0,142100166	-0,623264422
NC_01685(	RefSeq	CDS	4538892	4541039	-	0	ID=cds4959	0	27,5	STM14_5155	fdhF	formate dehydrogenase	3,978804635	-2,448045729
NC_01685(	RefSeq	CDS	4541394	4542302	-	0	ID=cds4960	0	1	STM14_5156	lpxO	putative dioxygenase	0,142100166	-0,623264422
NC_01685(	RefSeq	CDS	4542560	4543102	-	0	ID=cds4961	0	2	STM14_5157	phnO	aminoalkylphosphonic acid N-acetyltransferase	0,284200331	-1,182723423
NC_01685(	RefSeq	CDS	4543146	4543589	-	0	ID=cds4962	0	0,5	STM14_5158	phnB	hypothetical protein	0	NA
NC_01685(	RefSeq	CDS	4543709	4544044	-	0	ID=cds4963	0	17	STM14_5159	phnA	hypothetical protein	2,415702814	-2,412509737
NC_01685(	RefSeq	CDS	4544189	4544284	+	0	ID=cds4964	0	0	STM14_5160	STM14_5160	hypothetical protein	0	NA
NC_01685(	RefSeq	CDS	4544512	4546014	+	0	ID=cds4965	2	5	STM14_5161	proP	proline%2Fglycine betaine transporter	4,229145568	1,438201827
NC_01685(	RefSeq	CDS	4546069	4546173	+	0	ID=cds4966	0	12	STM14_5162	STM14_5162	hypothetical protein	1,705201986	-2,329925387
NC_01685(	RefSeq	CDS	4546181	4547251	-	0	ID=cds4967	0	10	STM14_5163	basS	sensor protein BasS%2FPmrB	1,421001655	-2,256999172
NC_01685(	RefSeq	CDS	4547261	4547929	-	0	ID=cds4968	0	25,5	STM14_5164	basR	DNA-binding transcriptional regulator BasR	3,694604304	-2,438835558
NC_01685(	RefSeq	CDS	4547926	4549569	-	0	ID=cds4969	0	49,5	STM14_5165	yjdB	putative cell division protein	7,105008277	-2,173389122

NC_01685(	RefSeq	CDS	4549703	4551040	-	0	ID=cds4970	0	7	STM14_5166	yjdE	arginine:agmatin antiporter	0,994701159	-2,072978628
NC_01685(	RefSeq	CDS	4551180	4551941	-	0	ID=cds4971	0	3,5	STM14_5167	adiY	transcriptional activator	0,568400662	-1,710438693
NC_01685(	RefSeq	CDS	4552110	4552238	+	0	ID=cds4972	0	1,25	STM14_5168	STM14_5168	hypothetical protein	0,142100166	-0,623264422
NC_01685(	RefSeq	CDS	4552239	4554509	-	0	ID=cds4973	0	4	STM14_5169	adi	catabolic arginine decarboxylase	0,568400662	-1,710438693
NC_01685(	RefSeq	CDS	4554739	4555671	-	0	ID=cds4974	0	19	STM14_5170	melR	DNA-binding transcriptional regulator MelR	2,699003145	-2,42419778
NC_01685(	RefSeq	CDS	4555940	4557295	+	0	ID=cds4975	0	73,5	STM14_5171	melA	alpha-galactosidase	10,51541225	-2,024396986
NC_01685(	RefSeq	CDS	4557379	4558809	+	0	ID=cds4976	0	28,5	STM14_5172	melB	melibiose:sodium symporter	3,978804635	-2,448045729
NC_01685(	RefSeq	CDS	4558907	4560553	-	0	ID=cds4977	0	342,1666667	STM14_5173	fumB	fumarase B	48,59825661	-1,933828099
NC_01685(	RefSeq	CDS	4560649	4561989	-	0	ID=cds4978	0	416,5	STM14_5174	dcuB	anaerobic C4-dicarboxylate transporter	59,11366886	-1,942661066
NC_01685(	RefSeq	CDS	4562136	4562402	-	0	ID=cds4979	0	44	STM14_5175	STM14_5175	putative cytoplasmic protein	6,252407284	-2,216733793
NC_01685(	RefSeq	CDS	4562719	4563438	-	0	ID=cds4980	0	9	STM14_5176	dcuR	DNA-binding transcriptional activator DcuR	1,27890149	-2,207741391
NC_01685(	RefSeq	CDS	4563435	4565066	-	0	ID=cds4981	0	7	STM14_5177	dcuS	sensory histidine kinase DcuS	0,994701159	-2,072978628
NC_01685(	RefSeq	CDS	4565422	4567851	+	0	ID=cds4982	0	93,5	STM14_5178	STM14_5178	putative anaerobic dimethylsulfoxide reductase subunit A	13,35741556	-1,928482877
NC_01685(	RefSeq	CDS	4567865	4568491	+	0	ID=cds4983	0	28	STM14_5179	STM14_5179	putative anaerobic dimethylsulfoxide reductase subunit B	3,978804635	-2,448045729
NC_01685(	RefSeq	CDS	4568484	4569257	+	0	ID=cds4984	0	11,25	STM14_5180	STM14_5180	putative anaerobic dimethylsulfoxide reductase subunit C	1,563101821	-2,297202414
NC_01685(	RefSeq	CDS	4569273	4569926	+	0	ID=cds4985	0	18,25	STM14_5181	STM14_5181	putative anaerobic dehydrogenase component	2,55780298	-2,419166771
NC_01685(	RefSeq	CDS	4570010	4571410	-	0	ID=cds4986	0	0	STM14_5182	STM14_5182	hypothetical protein	0	NA
NC_01685(	RefSeq	CDS	4571436	4571549	+	0	ID=cds4987	0	0	STM14_5183	STM14_5183	hypothetical protein	0	NA
NC_01685(	RefSeq	CDS	4571714	4572619	+	0	ID=cds4988	0	7	STM14_5184	STM14_5184	putative inner membrane protein	0,994701159	-2,072978628
NC_01685(	RefSeq	CDS	4572913	4573182	-	0	ID=cds4989	0	2,5	STM14_5185	STM14_5185	hypothetical protein	0,284200331	-1,182723423
NC_01685(	RefSeq	CDS	4573190	4573405	-	0	ID=cds4990	0	8	STM14_5186	STM14_5186	putative cytoplasmic protein	1,136801324	-2,147403465
NC_01685(	RefSeq	CDS	4573428	4573715	-	0	ID=cds4991	0	10	STM14_5187	STM14_5187	putative regulatory protein	1,421001655	-2,256999172
NC_01685(	RefSeq	CDS	4573712	4574587	-	0	ID=cds4992	0	32,5	STM14_5188	STM14_5188	putative DNA-binding protein	4,547205297	-2,489878815
NC_01685(	RefSeq	CDS	4574725	4574850	+	0	ID=cds4993	0	0	STM14_5189	STM14_5189	hypothetical protein	0	NA
NC_01685(	RefSeq	CDS	4574852	4575073	-	0	ID=cds4994	0	0	STM14_5190	STM14_5190	putative cytoplasmic protein	0	NA
NC_01685(	RefSeq	CDS	4575390	4575683	+	0	ID=cds4995	0	5	STM14_5191	STM14_5191	hypothetical protein	0,710500828	-1,864806942
NC_01685(	RefSeq	CDS	4575680	4576171	+	0	ID=cds4996	0	6	STM14_5192	STM14_5192	putative acetyltransferase	0,852600993	-1,98056697
NC_01685(	RefSeq	CDS	4576419	4577171	-	0	ID=cds4997	0	15,5	STM14_5193	phoN	non-specific acid phosphatase	2,273602649	-2,403729808
NC_01685(	RefSeq	CDS	4578381	4578758	-	0	ID=cds4998	0	0	STM14_5194	STM14_5194	ethyl tert-butyl ether degradation EthD	0	NA
NC_01685(	RefSeq	CDS	4578830	4579162	+	0	ID=cds4999	0	2,5	STM14_5195	STM14_5195	putative regulatory protein	0,284200331	-1,182723423
NC_01685(	RefSeq	CDS	4579152	4579388	-	0	ID=cds5000	0	0	STM14_5196	STM14_5196	hypothetical protein	0	NA
NC_01685(	RefSeq	CDS	4579601	4580176	-	0	ID=cds5001	0	15	STM14_5198	yjdC	putative transcriptional regulator	2,131502483	-2,392032191
NC_01685(	RefSeq	CDS	4580213	4581916	-	0	ID=cds5002	0	22	STM14_5199	dipZ	thiol:disulfide interchange protein	3,126203642	-2,431590384
NC_01685(	RefSeq	CDS	4581892	4582239	-	0	ID=cds5003	0	9,5	STM14_5200	cutA	divalent-cation tolerance protein CutA	1,421001655	-2,256999172
NC_01685(	RefSeq	CDS	4582360	4583661	-	0	ID=cds5004	0	499	STM14_5201	dcuA	anaerobic C4-dicarboxylate transporter	70,9079826	-1,94960687
NC_01685(	RefSeq	CDS	4583776	4585212	-	0	ID=cds5005	1	1979,583333	STM14_5202	aspA	aspartate ammonia-lyase	283,1176501	-4,027943758
NC_01685(	RefSeq	CDS	4585245	4585367	-	0	ID=cds5006	0	38,75	STM14_5203	STM14_5203	hypothetical protein	5,541906456	-2,25579729
NC_01685(	RefSeq	CDS	4585553	4586029	+	0	ID=cds5007	1	4,5	STM14_5204	fxsA	FxsA protein	2,327723032	1,125268706
NC_01685(	RefSeq	CDS	4586088	4587329	-	0	ID=cds5008	0	12	STM14_5205	yjeH	inner membrane protein YjeH	1,705201986	-2,329925387
NC_01685(	RefSeq	CDS	4587425	4587898	+	0	ID=cds5009	0	127,5	STM14_5206	groES	co-chaperonin GroES	18,18882119	-1,860051483
NC_01685(	RefSeq	CDS	4587942	4589588	+	0	ID=cds5010	0	256,5	STM14_5207	groEL	chaperonin GroEL	36,37764238	-1,917726992
NC_01685(	RefSeq	CDS	4589666	4589833	-	0	ID=cds5011	0	0	STM14_5208	STM14_5208	hypothetical protein	0	NA
NC_01685(	RefSeq	CDS	4589814	4590185	+	0	ID=cds5012	0,5	49,25	STM14_5209	yjeI	putative outer membrane lipoprotein	6,962908111	-2,178239812
NC_01685(	RefSeq	CDS	4590233	4591090	-	0	ID=cds5013	0	5	STM14_5210	yjeJ	putative inner membrane protein	0,710500828	-1,864806942
NC_01685(	RefSeq	CDS	4591089	4591223	+	0	ID=cds5014	0	0,5	STM14_5211	STM14_5211	hypothetical protein	0	NA
NC_01685(	RefSeq	CDS	4591232	4591378	-	0	ID=cds5015	0	2	STM14_5212	STM14_5212	hypothetical protein	0,284200331	-1,182723423
NC_01685(	RefSeq	CDS	4591372	4592400	-	0	ID=cds5016	0	12,5	STM14_5213	yjeK	putative aminomutase	1,705201986	-2,329925387
NC_01685(	RefSeq	CDS	4592441	4593007	+	0	ID=cds5017	0	33,5	STM14_5214	efp	elongation factor P	4,831405628	-2,523251848
NC_01685(	RefSeq	CDS	4593026	4593202	+	0	ID=cds5018	0	1,5	STM14_5215	ecnA	putative entericidin A precursor	0,284200331	-1,182723423
NC_01685(	RefSeq	CDS	4593310	4593456	+	0	ID=cds5019	2	10	STM14_5216	ecnB	entericidin B membrane lipoprotein	4,939646395	0,828530271
NC_01685(	RefSeq	CDS	4593487	4594074	-	0	ID=cds5020	0	1	STM14_5217	ecnR	putative regulatory protein	0,142100166	-0,623264422
NC_01685(	RefSeq	CDS	4594331	4594648	+	0	ID=cds5021	0	6	STM14_5218	sugE	quaternary ammonium compound-resistance protein Sugl	0,852600993	-1,98056697
NC_01685(	RefSeq	CDS	4594665	4595198	-	0	ID=cds5022	0	8,5	STM14_5219	blc	outer membrane lipoprotein Blc	1,136801324	-2,147403465
NC_01685(	RefSeq	CDS	4595310	4595669	-	0	ID=cds5023	0	168	STM14_5220	frdD	fumarate reductase subunit D	23,87282781	-1,886360475
NC_01685(	RefSeq	CDS	4595680	4596075	-	0	ID=cds5024	0	144,5	STM14_5221	frdC	fumarate reductase subunit C	20,46242384	-1,87208996
NC_01685(	RefSeq	CDS	4596086	4596820	-	0	ID=cds5025	0	464,75	STM14_5222	frdB	fumarate reductase iron-sulfur subunit	66,07657697	-1,947045854
NC_01685(	RefSeq	CDS	4596813	4598603	-	0	ID=cds5026	0	900,25	STM14_5223	frdA	fumarate reductase flavoprotein subunit	127,890149	-1,965633929
NC_01685(	RefSeq	CDS	4598926	4599903	+	0	ID=cds5027	1	20	STM14_5224	yjeA	lysyl-tRNA synthetase	4,601325681	-0,429413798
NC_01685(	RefSeq	CDS	4599983	4600087	+	0	ID=cds5028	0	0	STM14_5225	STM14_5225	hypothetical protein	0	NA
NC_01685(	RefSeq	CDS	4600129	4601631	+	0	ID=cds5029	0	6,5	STM14_5226	yjeM	putative amino-acid transport protein	0,852600993	-1,98056697
NC_01685(	RefSeq	CDS	4601683	4601997	+	0	ID=cds5030	0	14,5	STM14_5227	yjeO	putative inner membrane protein	1,989402318	-2,376535557
NC_01685(	RefSeq	CDS	4602064	4605390	-	0	ID=cds5031	0	17,5	STM14_5228	yjeP	hypothetical protein	2,55780298	-2,419166771
NC_01685(	RefSeq	CDS	4605409	4606377	-	0	ID=cds5032	0	23	STM14_5229	psd	phosphatidylserine decarboxylase	3,268303807	-2,432747446
NC_01685(	RefSeq	CDS	4606469	4607521	-	0	ID=cds5033	0	24	STM14_5230	yjeQ	ribosome-associated GTPase	3,410403973	-2,434086198
NC_01685(	RefSeq	CDS	4607628	4608173	+	0	ID=cds5034	0	15	STM14_5231	orn	oligoribonuclease	2,131502483	-2,392032191
NC_01685(	RefSeq	CDS	4608235	4608975	-	0	ID=cds5035	0	11	STM14_5232	STM14_5232	putative arginine-binding periplasmic protein	1,563101821	-2,297202414
NC_01685(	RefSeq	CDS	4610060	4611199	-	0	ID=cds5036	0	12,5	STM14_5236	yjeS	putative FeS protein	1,705201986	-2,329925387
NC_01685(	RefSeq	CDS	4611198	4612745	+	0	ID=cds5037	0	5,75	STM14_5237	yjeF	hypothetical protein	0,852600993	-1,98056697
NC_01685(	RefSeq	CDS	4612717	4613178	+	0	ID=cds5038	0	3	STM14_5238	yjeE	putative ATPase	0,426300497	-1,498211557
NC_01685(	RefSeq	CDS	4613195	4614514	+	0	ID=cds5039	0	16,5	STM14_5239	amiB	N-acetylmuramoyl-l-alanine amidase II	2,273602649	-2,403729808
NC_01685(	RefSeq	CDS	4614524	4616380	+	0	ID=cds5040	8	28,5	STM14_5240	mutL	DNA mismatch repair protein	18,05338359	1,263345574
NC_01685(	RefSeq	CDS	4616373	4617323	+	0	ID=cds5041	2	42,5	STM14_5241	miaA	tRNA delta%282%29-isopentenylpyrophosphate transfera	9,486851692	-0,503856957
NC_01685(	RefSeq	CDS	4617406	4617714	+	0	ID=cds5042	3	35	STM14_5242	hfq	RNA-binding protein Hfq	10,2514729	0,057654837
NC_01685(	RefSeq	CDS	4617786	4619066	+	0	ID=cds5043	2	77	STM14_5243	hflX	putative GTPase HflX	14,46035749	-1,107647426
NC_01685(	RefSeq	CDS	4619281	4620540	+	0	ID=cds5044	0	61,75	STM14_5244	hflK	FtsH protease regulator HflK	8,810210263	-2,091539774
NC_01685(	RefSeq	CDS	4620543	4621547	+	0	ID=cds5045	2	74,25	STM14_5245	hflC	FtsH protease regulator HflC	14,03405699	-1,067838496
NC_01685(	RefSeq	CDS	4621626	4621823	+	0	ID=cds5046	0	1,75	STM14_5246	yjeT	putative inner membrane protein	0,284200331	-1,182723423
NC_01685(	RefSeq	CDS	4621703	4621933	+	0	ID=cds5047	0	13,75	STM14_5247	STM14_5247	hypothetical protein	1,989402318	-2,376535557
NC_01685(	RefSeq	CDS	4621926	4623224	+	0	ID=cds5048	0	104	STM14_5248	purA	adenylosuccinate synthetase	14,77841722	-1,890380653
NC_01685(	RefSeq	CDS	4623431	4623856	+	0	ID=cds5049	7	24	STM14_5249	yjeB	transcriptional repressor NsrR	15,72566056	1,278159
NC_01685(	RefSeq	CDS	4623894	4626332	+	0	ID=cds5050	0	108,5	STM14_5250	vacB	exoribonuclease R	15,34681788	-1,876042944
NC_01685(	RefSeq	CDS	4626423	4627154	+	0	ID=cds5051	0	11	STM14_5251	yjfH	23S rRNA %28guanosine-2%27-O-%29-methyltransferase	1,563101821	-2,297202414
NC_01685(	RefSeq	CDS	4627286	4627690	+	0	ID=cds5052	0	0	STM14_5252	yjfI	putative cytoplasmic protein	0	NA
NC_01685(	RefSeq	CDS	4627705	4628403	+	0	ID=cds5053	0	0	STM14_5253	yjfJ	putative phage shock protein A	0	NA
NC_01685(	RefSeq	CDS	4628403	4629473	+	0	ID=cds5054	0	0	STM14_5254	STM14_5254	putative potassium channel	0	NA
NC_01685(	RefSeq	CDS	4629446	4630129	+	0	ID=cds5055	0	0	STM14_5255	yjfK	putative cytoplasmic protein	0	NA
NC_01685(	RefSeq	CDS	4630147	4630545	+	0	ID=cds5056	0	0	STM14_5256	yjfL	putative inner membrane protein	0	NA
NC_01685(	RefSeq	CDS	4630555	4631193	+	0	ID=cds5057	0	1	STM14_5257	yjfM	putative inner membrane protein	0,142100166	-0,623264422
NC_01685(	RefSeq	CDS	4631196	4632359	+	0	ID=cds5058	0	4,5	STM14_5258	yjfC	putative glutathionylspermidine synthase	0,568400662	-1,710438693
NC_01685(	RefSeq	CDS	4632426	4634066	+	0	ID=cds5059	0	6	STM14_5259	aidB	isovaleryl CoA dehydrogenase	0,852600993	-1,98056697
NC_01685(	RefSeq	CDS	4634111	4634431	-	0	ID=cds5060	0	88	STM14_5260	yjfN	putative inner membrane protein	12,50481457	-1,954585237
NC_01685(	RefSeq	CDS	4634517	4634846	-	0	ID=cds5061	0	93,5	STM14_5261	yjfO	putative lipoprotein	13,35741556	-1,928482877
NC_01685(	RefSeq	CDS	4635030	4635779	+	0	ID=cds5062	0	1	STM14_5262	yjfP	esterase	0,142100166	-0,623264422
NC_01685(	RefSeq	CDS	4635776	4636531	-	0	ID=cds5063	0	4	STM14_5263	yjfQ	transcriptional repressor UlaR	0,568400662	-1,710438693
NC_01685(	RefSeq	CDS	4636636	4637700	-	0	ID=cds5064	0	1	STM14_5264	yjfR	putative L-ascorbate 6-phosphate lactonase	0,142100166	-0,623264422
NC_01685(	RefSeq	CDS	4638063	4639460	+	0	ID=cds5065	0	3	STM14_5265	ulaA_3	PTS system ascorbate-specific transporter subunit IIC	0,426300497	-1,498211557
NC_01685(	RefSeq	CDS	4639476	4639781	+	0	ID=cds5066	0	1	STM14_5266	sgaB	L-ascorbate-specific enzyme IIB component of PTS	0,142100166	-0,623264422
NC_01685(	RefSeq	CDS	4639791	4640255	+	0	ID=cds5067	0	1,5	STM14_5267	ptxA	L-ascorbate-specific enzyme IIA component of PTS	0,284200331	-1,182723423
NC_01685(	RefSeq	CDS	4640269	4640919	+	0	ID=cds5068	0	4,5	STM14_5268	ulaD	3-keto-L-gulonate-6-phosphate decarboxylase	0,568400662	-1,710438693
NC_01685(	RefSeq	CDS	4640929	4641783	+	0	ID=cds5069	0	0	STM14_5269	sgaU	L-xylulose 5-phosphate 3-epimerase	0	NA
NC_01685(	RefSeq	CDS	4641783	4642469	+	0	ID=cds5070	0	2,5	STM14_5270	sgaE	L-ribulose-5-phosphate 4-epimerase	0,284200331	-1,182723423
NC_01685(	RefSeq	CDS	4642598	4642873	-	0	ID=cds5071	0	1	STM14_5271	yjfY	putative outer membrane protein	0,142100166	-0,623264422
NC_01685(	RefSeq	CDS	4642797	4642994	-	0	ID=cds5072	0	0	STM14_5272	STM14_5272	hypothetical protein	0	NA
NC_01685(	RefSeq	CDS	4642921	4643028	+	0	ID=cds5073	0	0	STM14_5273	STM14_5273	hypothetical protein	0	NA
NC_01685(	RefSeq	CDS	4643067	4643273	+	0	ID=cds5074	0	5,5	STM14_5274	STM14_5274	putative cytoplasmic protein	0,852600993	-1,98056697
NC_01685(	RefSeq	CDS	4643344	4643739	+	0	ID=cds5075	0	62	STM14_5275	rpsF	30S ribosomal protein S6	8,810210263	-2,091539774
NC_01685(	RefSeq	CDS	4643746	4644060	+	0	ID=cds5076	0	49	STM14_5276	priB	primosomal replication protein N	6,962908111	-2,178239812
NC_01685(	RefSeq	CDS	4644065	4644292	+	0	ID=cds5077	4	35,5	STM14_5277	rpsR	30S ribosomal protein S18	12,15289544	0,31489869
NC_01685(	RefSeq	CDS	4644334	4644783	+	0	ID=cds5078	0	44,5	STM14_5278	rplI	50S ribosomal protein L9	6,252407284	-2,216733793
NC_01685(	RefSeq	CDS	4644931	4645857	+	0	ID=cds5079	0	4	STM14_5279	yifZ	putative permease	0,568400662	-1,710438693
NC_01685(	RefSeq	CDS	4645907	4646542	-	0	ID=cds5080	0	9	STM14_5280	ytfB	putative cell envelope opacity-associated protein A	1,27890149	-2,207741391
NC_01685(	RefSeq	CDS	4646651	4647382	+	0	ID=cds5081	0	27,5	STM14_5281	fklB	peptidyl-prolyl cis-trans isomerase	3,978804635	-2,448045729
NC_01685(	RefSeq	CDS	4647678	4649087	+	0	ID=cds5082	0	13,5	STM14_5282	cycA	D-alanine%2FD-serine%2Fglycine permease	1,989402318	-2,376535557
NC_01685(	RefSeq	CDS	4649199	4649861	-	0	ID=cds5083	0	1	STM14_5283	ytfE	cell morphogenesis%2Fcell wall metabolism regulator	0,142100166	-0,623264422
NC_01685(	RefSeq	CDS	4649964	4650929	-	0	ID=cds5084	0	2,5	STM14_5284	ytfF	putative cationic amino acid transporter	0,284200331	-1,182723423
NC_01685(	RefSeq	CDS	4651010	4651858	-	0	ID=cds5085	0	1,5	STM14_5285	ytfG	putative reductase	0,284200331	-1,182723423
NC_01685(	RefSeq	CDS	4651946	4652332	+	0	ID=cds5086	0	0	STM14_5286	ytfH	putative transcriptional regulator	0	NA
NC_01685(	RefSeq	CDS	4652390	4654333	-	0	ID=cds5087	0	20,5	STM14_5287	cpdB	bifunctional 2%27%2C3%27-cyclic nucleotide 2%27-phosp	2,842003311	-2,427810709
NC_01685(	RefSeq	CDS	4654346	4654567	-	0	ID=cds5088	0	3	STM14_5288	STM14_5288	hypothetical protein	0,426300497	-1,498211557
NC_01685(	RefSeq	CDS	4654601	4655341	+	0	ID=cds5089	0	7,75	STM14_5289	cysQ	adenosine 3%27-phosphate 5%27-phosphosulfate 3%27%	1,136801324	-2,147403465
NC_01685(	RefSeq	CDS	4655331	4655888	-	0	ID=cds5090	0	1,5	STM14_5290	ytfJ	putative transcriptional regulator	0,284200331	-1,182723423
NC_01685(	RefSeq	CDS	4655902	4656081	-	0	ID=cds5091	0	0	STM14_5291	STM14_5291	hypothetical protein	0	NA
NC_01685(	RefSeq	CDS	4656215	4656421	+	0	ID=cds5092	0	18	STM14_5292	ytfK	putative cytoplasmic protein	2,55780298	-2,419166771
NC_01685(	RefSeq	CDS	4656506	4657849	-	0	ID=cds5093	0	29	STM14_5293	ytfL	putative hemolysin-like protein	4,120904801	-2,455089955

NC_01685(	RefSeq	CDS	4658032	4658670	-	0	ID=cds5094	2	4	STM14_5294	msrA	methionine sulfoxide reductase A	4,087045402	1,631390028
NC_01685(	RefSeq	CDS	4658725	4658841	+	0	ID=cds5095	1	1,5	STM14_5295	STM14_5295	hypothetical protein	2,043522701	1,824239109
NC_01685(	RefSeq	CDS	4658883	4660616	+	0	ID=cds5096	1	22	STM14_5296	ytfM	putative outer membrane protein	4,885526012	-0,515045314
NC_01685(	RefSeq	CDS	4660613	4664392	+	0	ID=cds5097	5	43	STM14_5297	ytfN	hypothetical protein	14,90691897	0,365256911
NC_01685(	RefSeq	CDS	4664395	4664739	+	0	ID=cds5098	0	12,5	STM14_5298	ytfP	putative cytoplasmic protein	1,705201986	-2,329925387
NC_01685(	RefSeq	CDS	4664793	4666001	-	0	ID=cds5099	0	0	STM14_5299	STM14_5299	putative permease	0	NA
NC_01685(	RefSeq	CDS	4665998	4667161	-	0	ID=cds5100	0	0	STM14_5300	STM14_5300	putative metallo-dependent hydrolase	0	NA
NC_01685(	RefSeq	CDS	4667571	4668101	-	0	ID=cds5101	0	59	STM14_5301	ppa	inorganic pyrophosphatase	8,383909767	-2,110782333
NC_01685(	RefSeq	CDS	4668328	4669326	-	0	ID=cds5102	0,5	83,75	STM14_5302	fbp	fructose-1%2C6-bisphosphatase	11,93641391	-1,973338016
NC_01685(	RefSeq	CDS	4669323	4669508	-	0	ID=cds5103	0,5	3,75	STM14_5303	STM14_5303	hypothetical protein	0,568400662	-1,710438693
NC_01685(	RefSeq	CDS	4669501	4670880	+	0	ID=cds5104	0	21	STM14_5304	mpl	UDP-N-acetylmuramate%2FL-alanyl-gamma-D-glutamyl- r	2,984103476	-2,430148754
NC_01685(	RefSeq	CDS	4670877	4671137	-	0	ID=cds5105	0	0,5	STM14_5305	STM14_5305	hypothetical protein	0	NA
NC_01685(	RefSeq	CDS	4671075	4671302	+	0	ID=cds5106	0	5	STM14_5306	STM14_5306	hypothetical protein	0,710500828	-1,864806942
NC_01685(	RefSeq	CDS	4671371	4672204	+	0	ID=cds5107	0	17	STM14_5307	STM14_5307	putative transcriptional regulator	2,415702814	-2,412509737
NC_01685(	RefSeq	CDS	4672255	4673688	-	0	ID=cds5108	0	0	STM14_5308	STM14_5308	sugar transporter	0	NA
NC_01685(	RefSeq	CDS	4673931	4674110	-	0	ID=cds5109	0	0	STM14_5309	STM14_5309	hypothetical protein	0	NA
NC_01685(	RefSeq	CDS	4674147	4675583	+	0	ID=cds5110	0	4,5	STM14_5310	STM14_5310	sugar transporter	0,568400662	-1,710438693
NC_01685(	RefSeq	CDS	4675936	4676745	-	0	ID=cds5111	0	7,5	STM14_5311	STM14_5311	putative inner membrane protein	1,136801324	-2,147403465
NC_01685(	RefSeq	CDS	4676770	4678275	-	0	ID=cds5112	0	4,5	STM14_5312	STM14_5312	putative NAD-dependent aldehyde dehydrogenase	0,568400662	-1,710438693
NC_01685(	RefSeq	CDS	4678291	4678593	-	0	ID=cds5113	0	0	STM14_5313	STM14_5313	putative cytoplasmic protein	0	NA
NC_01685(	RefSeq	CDS	4678716	4679540	+	0	ID=cds5114	0	1	STM14_5314	STM14_5314	putative DNA-binding protein	0,142100166	-0,623264422
NC_01685(	RefSeq	CDS	4679544	4679654	+	0	ID=cds5115	0	0	STM14_5315	STM14_5315	hypothetical protein	0	NA
NC_01685(	RefSeq	CDS	4679815	4680720	+	0	ID=cds5116	0	1,5	STM14_5316	STM14_5316	putative endonuclease	0,284200331	-1,182723423
NC_01685(	RefSeq	CDS	4680739	4681749	+	0	ID=cds5117	0	2	STM14_5317	STM14_5317	putative dehydrogenase	0,284200331	-1,182723423
NC_01685(	RefSeq	CDS	4681846	4683189	+	0	ID=cds5118	0	0	STM14_5318	srfJ	lysosomal glucosyl ceramidase-like protein	0	NA
NC_01685(	RefSeq	CDS	4683190	4684023	+	0	ID=cds5119	0	3	STM14_5319	STM14_5319	putative endonuclease	0,426300497	-1,498211557
NC_01685(	RefSeq	CDS	4684020	4685186	-	0	ID=cds5120	0	0	STM14_5320	STM14_5320	hypothetical protein	0	NA
NC_01685(	RefSeq	CDS	4685247	4687178	-	0	ID=cds5121	0	4	STM14_5321	STM14_5321	putative carbohydrate kinase	0,568400662	-1,710438693
NC_01685(	RefSeq	CDS	4689572	4689691	+	0	ID=cds5122	0	0	STM14_5323	STM14_5323	hypothetical protein	0	NA
NC_01685(	RefSeq	CDS	4689722	4690744	+	0	ID=cds5123	0	0	STM14_5324	STM14_5324	myo-inositol 2-dehydrogenase	0	NA
NC_01685(	RefSeq	CDS	4690817	4692043	+	0	ID=cds5124	0	0	STM14_5325	STM14_5325	putative permease	0	NA
NC_01685(	RefSeq	CDS	4692204	4693019	+	0	ID=cds5125	0	1,5	STM14_5326	STM14_5326	putative cytoplasmic protein	0,284200331	-1,182723423
NC_01685(	RefSeq	CDS	4693072	4693956	+	0	ID=cds5126	0	2	STM14_5327	STM14_5327	putative endonuclease	0,284200331	-1,182723423
NC_01685(	RefSeq	CDS	4694012	4694563	-	0	ID=cds5127	0	18,5	STM14_5328	yjgA	hypothetical protein	2,55780298	-2,419166771
NC_01685(	RefSeq	CDS	4694659	4696011	+	0	ID=cds5128	0	21,5	STM14_5329	pmbA	peptidase PmbA	3,126203642	-2,431590384
NC_01685(	RefSeq	CDS	4696110	4696496	+	0	ID=cds5129	0	2,5	STM14_5330	cybC	cytochrome b562	0,284200331	-1,182723423
NC_01685(	RefSeq	CDS	4696774	4697112	+	0	ID=cds5130	0	0	STM14_5331	STM14_5331	putative cytoplasmic protein	0	NA
NC_01685(	RefSeq	CDS	4697123	4697485	+	0	ID=cds5131	0	0	STM14_5332	STM14_5332	putative cytoplasmic protein	0	NA
NC_01685(	RefSeq	CDS	4697485	4697787	+	0	ID=cds5132	0	0	STM14_5333	STM14_5333	putative cytoplasmic protein	0	NA
NC_01685(	RefSeq	CDS	4697810	4698586	+	0	ID=cds5133	0	1	STM14_5334	STM14_5334	putative inner membrane protein	0,142100166	-0,623264422
NC_01685(	RefSeq	CDS	4698598	4699242	+	0	ID=cds5134	0	1	STM14_5335	STM14_5335	putative inner membrane protein	0,142100166	-0,623264422
NC_01685(	RefSeq	CDS	4699301	4700434	+	0	ID=cds5135	0	0	STM14_5336	STM14_5336	dihydroorotase	0	NA
NC_01685(	RefSeq	CDS	4700418	4701536	+	0	ID=cds5136	0	5	STM14_5337	STM14_5337	putative selenocysteine synthase	0,710500828	-1,864806942
NC_01685(	RefSeq	CDS	4701533	4702273	+	0	ID=cds5137	0	1	STM14_5338	STM14_5338	4-hydroxy-2-oxoglutarate aldolase	0,142100166	-0,623264422
NC_01685(	RefSeq	CDS	4702290	4704203	+	0	ID=cds5138	0	4,5	STM14_5339	STM14_5339	phosphotransferase system mannitol%2Ffructose-specific	0,568400662	-1,710438693
NC_01685(	RefSeq	CDS	4704281	4704523	+	0	ID=cds5139	0	4,5	STM14_5340	STM14_5340	bifunctional antitoxin%2Ftranscriptional repressor RelB	0,568400662	-1,710438693
NC_01685(	RefSeq	CDS	4704513	4704797	+	0	ID=cds5140	0	10,5	STM14_5341	STM14_5341	putative inner membrane protein	1,421001655	-2,256999172
NC_01685(	RefSeq	CDS	4704801	4705265	-	0	ID=cds5141	0	19,5	STM14_5342	nrdG	anaerobic ribonucleotide reductase-activating protein	2,842003311	-2,427810709
NC_01685(	RefSeq	CDS	4705386	4707524	-	0	ID=cds5142	0	170	STM14_5343	nrdD	anaerobic ribonucleoside triphosphate reductase	24,15702814	-1,887389583
NC_01685(	RefSeq	CDS	4707729	4707899	+	0	ID=cds5143	0	0	STM14_5344	STM14_5344	hypothetical protein	0	NA
NC_01685(	RefSeq	CDS	4707933	4709585	-	0	ID=cds5144	0	6,5	STM14_5345	treC	trehalose-6-phosphate hydrolase	0,852600993	-1,98056697
NC_01685(	RefSeq	CDS	4711078	4711185	+	0	ID=cds5145	0	0	STM14_5347	STM14_5347	hypothetical protein	0	NA
NC_01685(	RefSeq	CDS	4711190	4712137	-	0	ID=cds5146	0	3	STM14_5348	treR	trehalose repressor	0,426300497	-1,498211557
NC_01685(	RefSeq	CDS	4712521	4715229	+	0	ID=cds5147	5	11	STM14_5349	mgtA	magnesium-transporting ATPase MgtA	10,35971367	1,647620804
NC_01685(	RefSeq	CDS	4715345	4715791	-	0	ID=cds5148	0	19,83333333	STM14_5350	STM14_5350	putative transposase	2,842003311	-2,427810709
NC_01685(	RefSeq	CDS	4715866	4716252	-	0	ID=cds5149	0	73,5	STM14_5351	yjgF	putative translation initiation inhibitor	10,51541225	-2,024396986
NC_01685(	RefSeq	CDS	4716329	4716790	-	0	ID=cds5150	0	0,5	STM14_5352	pyrI	aspartate carbamoyltransferase regulatory subunit	0	NA
NC_01685(	RefSeq	CDS	4716803	4717738	-	0	ID=cds5151	0	3,5	STM14_5353	pyrB	aspartate carbamoyltransferase catalytic subunit	0,568400662	-1,710438693
NC_01685(	RefSeq	CDS	4717774	4717875	-	0	ID=cds5152	0	14,5	STM14_5354	pyrL	pyrBI operon leader peptide	1,989402318	-2,376535557
NC_01685(	RefSeq	CDS	4717965	4718453	-	0	ID=cds5153	0	61,5	STM14_5355	STM14_5355	putative arginine repressor	8,810210263	-2,091539774
NC_01685(	RefSeq	CDS	4718450	4718566	-	0	ID=cds5154	0	12	STM14_5356	STM14_5356	hypothetical protein	1,705201986	-2,329925387
NC_01685(	RefSeq	CDS	4718640	4720043	-	0	ID=cds5155	0	252,3333333	STM14_5357	STM14_5357	putative arginine repressor	35,80924172	-1,916734799
NC_01685(	RefSeq	CDS	4720099	4721103	-	0	ID=cds5156	0	338,9166667	STM14_5358	STM14_5358	ornithine carbamoyltransferase	48,17195612	-1,933394402
NC_01685(	RefSeq	CDS	4721103	4721225	-	0	ID=cds5157	0	53,5	STM14_5359	STM14_5359	hypothetical protein	7,673408939	-2,144619715
NC_01685(	RefSeq	CDS	4721215	4722147	-	0	ID=cds5158	0	222,5833333	STM14_5360	STM14_5360	carbamate kinase	31,68833691	-1,908582718
NC_01685(	RefSeq	CDS	4722158	4723378	-	0	ID=cds5159	0	306,5	STM14_5361	STM14_5361	arginine deiminase	43,48265065	-1,928105601
NC_01685(	RefSeq	CDS	4723479	4723670	+	0	ID=cds5160	0	0	STM14_5362	STM14_5362	hypothetical protein	0	NA
NC_01685(	RefSeq	CDS	4724054	4724506	+	0	ID=cds5161	0	1	STM14_5363	yjgK	putative cytoplasmic protein	0,142100166	-0,623264422
NC_01685(	RefSeq	CDS	4724580	4725584	-	0	ID=cds5162	0	1	STM14_5364	argI	ornithine carbamoyltransferase subunit I	0,142100166	-0,623264422
NC_01685(	RefSeq	CDS	4725750	4726166	+	0	ID=cds5163	0	13,5	STM14_5365	yjgD	hypothetical protein	1,989402318	-2,376535557
NC_01685(	RefSeq	CDS	4726178	4726990	+	0	ID=cds5164	0	6,5	STM14_5366	miaE	hydroxylase	0,852600993	-1,98056697
NC_01685(	RefSeq	CDS	4727023	4727145	-	0	ID=cds5165	0	0	STM14_5367	STM14_5367	hypothetical protein	0	NA
NC_01685(	RefSeq	CDS	4727224	4727712	-	0	ID=cds5166	0	0	STM14_5368	ytgA	putative inner membrane protein	0	NA
NC_01685(	RefSeq	CDS	4727819	4728322	-	0	ID=cds5167	0	4	STM14_5369	yjgM	putative acetyltransferase	0,568400662	-1,710438693
NC_01685(	RefSeq	CDS	4728517	4729704	+	0	ID=cds5168	0	1	STM14_5370	yjgN	putative inner membrane protein	0,142100166	-0,623264422
NC_01685(	RefSeq	CDS	4729846	4732701	-	0	ID=cds5169	0	87,75	STM14_5371	valS	valyl-tRNA synthetase	12,50481457	-1,954585237
NC_01685(	RefSeq	CDS	4732701	4733144	-	0	ID=cds5170	0	12,25	STM14_5372	holC	DNA polymerase III subunit chi	1,705201986	-2,329925387
NC_01685(	RefSeq	CDS	4733281	4734792	-	0	ID=cds5171	0	46,25	STM14_5373	pepA	leucyl aminopeptidase	6,536607615	-2,201017198
NC_01685(	RefSeq	CDS	4734836	4734955	-	0	ID=cds5172	0	7,25	STM14_5374	STM14_5374	putative cytoplasmic protein	0,994701159	-2,072978628
NC_01685(	RefSeq	CDS	4735187	4736287	+	0	ID=cds5173	0	20,25	STM14_5375	yjgP	putative permease	2,842003311	-2,427810709
NC_01685(	RefSeq	CDS	4736287	4737369	+	0	ID=cds5174	0	23,25	STM14_5376	yjgQ	putative permease	3,268303807	-2,432747446
NC_01685(	RefSeq	CDS	4737564	4738562	-	0	ID=cds5175	0	3,5	STM14_5377	idnR	L-idonate regulator	0,568400662	-1,710438693
NC_01685(	RefSeq	CDS	4738626	4739945	-	0	ID=cds5176	0	12	STM14_5378	idnT	L-idonate transport protein	1,705201986	-2,329925387
NC_01685(	RefSeq	CDS	4740007	4740771	-	0	ID=cds5177	0	1	STM14_5379	idnO	gluconate 5-dehydrogenase	0,142100166	-0,623264422
NC_01685(	RefSeq	CDS	4740796	4741827	-	0	ID=cds5178	0	3	STM14_5380	idnD	L-idonate 5-dehydrogenase	0,426300497	-1,498211557
NC_01685(	RefSeq	CDS	4741810	4741944	+	0	ID=cds5179	0	0,5	STM14_5381	STM14_5381	hypothetical protein	0	NA
NC_01685(	RefSeq	CDS	4742044	4742574	+	0	ID=cds5180	0	0,5	STM14_5382	idnK	D-gluconate kinase	0	NA
NC_01685(	RefSeq	CDS	4742602	4743621	-	0	ID=cds5181	0	1	STM14_5383	yjgB	putative alcohol dehydrogenase	0,142100166	-0,623264422
NC_01685(	RefSeq	CDS	4744147	4744434	+	0	ID=cds5182	0	1	STM14_5385	STM14_5385	putative integrase	0,142100166	-0,623264422
NC_01685(	RefSeq	CDS	4744796	4748311	+	0	ID=cds5183	0	27,5	STM14_5386	STM14_5386	putative DNA helicase	3,978804635	-2,448045729
NC_01685(	RefSeq	CDS	4748408	4749397	+	0	ID=cds5184	0	9,5	STM14_5387	STM14_5387	putative restriction endonuclease	1,421001655	-2,256999172
NC_01685(	RefSeq	CDS	4749510	4751594	-	0	ID=cds5185	0	26,75	STM14_5388	STM14_5388	putative ATP-dependent Lon protease	3,836704469	-2,44262576
NC_01685(	RefSeq	CDS	4751605	4754202	-	0	ID=cds5186	0	63,75	STM14_5389	STM14_5389	putative cytoplasmic protein	9,094410594	-2,079502768
NC_01685(	RefSeq	CDS	4754205	4755026	-	0	ID=cds5187	0	36,75	STM14_5390	STM14_5390	putative cytoplasmic protein	5,257706125	-2,272984196
NC_01685(	RefSeq	CDS	4755004	4756095	-	0	ID=cds5188	0	26,25	STM14_5391	STM14_5391	putative ABC-type sugar%2Fspermidine%2Fputrescine tra	3,694604304	-2,438835558
NC_01685(	RefSeq	CDS	4756095	4759772	-	0	ID=cds5189	0	58	STM14_5392	STM14_5392	putative type II restriction enzyme methylase subunit	8,241809601	-2,117369507
NC_01685(	RefSeq	CDS	4759818	4763459	-	0	ID=cds5190	3	69,33333333	STM14_5393	STM14_5393	putative DNA repair ATPase	15,08287853	-0,615362437
NC_01685(	RefSeq	CDS	4763471	4764073	-	0	ID=cds5191	0	14	STM14_5394	STM14_5394	putative cytoplasmic protein	1,989402318	-2,376535557
NC_01685(	RefSeq	CDS	4764070	4764672	-	0	ID=cds5192	0	24	STM14_5395	STM14_5395	putative inner membrane protein	3,410403973	-2,434086198
NC_01685(	RefSeq	CDS	4764865	4765110	-	0	ID=cds5193	0	0	STM14_5396	STM14_5396	molybdopterin-guanine dinucleotide biosynthesis protein	0	NA
NC_01685(	RefSeq	CDS	4765498	4766214	-	0	ID=cds5194	0	30	STM14_5398	yeeN	hypothetical protein	4,263004966	-2,464060063
NC_01685(	RefSeq	CDS	4766523	4766714	+	0	ID=cds5195	0	0	STM14_5399	STM14_5399	hypothetical protein	0	NA
NC_01685(	RefSeq	CDS	4766774	4768405	-	0	ID=cds5196	0	22,5	STM14_5400	yjhP	putative SAM-dependent methyltransferase	3,126203642	-2,431590384
NC_01685(	RefSeq	CDS	4768410	4768667	-	0	ID=cds5197	0	3,5	STM14_5401	STM14_5401	hypothetical protein	0,568400662	-1,710438693
NC_01685(	RefSeq	CDS	4768806	4769102	-	0	ID=cds5198	0	44	STM14_5402	STM14_5402	hypothetical protein	6,252407284	-2,216733793
NC_01685(	RefSeq	CDS	4769156	4769764	+	0	ID=cds5199	0	2,5	STM14_5403	STM14_5403	putative cytoplasmic protein	0,284200331	-1,182723423
NC_01685(	RefSeq	CDS	4770016	4771011	+	0	ID=cds5200	0	81,5	STM14_5404	STM14_5404	putative inner membrane protein	11,65221357	-1,983056535
NC_01685(	RefSeq	CDS	4771287	4772198	+	0	ID=cds5201	0	9,333333333	STM14_5405	STM14_5405	putative cytoplasmic protein	1,27890149	-2,207741391
NC_01685(	RefSeq	CDS	4772201	4772314	+	0	ID=cds5202	0	0,833333333	STM14_5406	STM14_5406	hypothetical protein	0,142100166	-0,623264422
NC_01685(	RefSeq	CDS	4772241	4772357	-	0	ID=cds5203	0	0	STM14_5407	STM14_5407	hypothetical protein	0	NA
NC_01685(	RefSeq	CDS	4772403	4772864	+	0	ID=cds5204	0	3	STM14_5408	STM14_5408	hypothetical protein	0,426300497	-1,498211557
NC_01685(	RefSeq	CDS	4772861	4773535	-	0	ID=cds5205	0	1,5	STM14_5409	STM14_5409	putative dienelactone hydrolase	0,284200331	-1,182723423
NC_01685(	RefSeq	CDS	4773638	4773850	+	0	ID=cds5206	0	0	STM14_5410	STM14_5410	hypothetical protein	0	NA
NC_01685(	RefSeq	CDS	4773885	4774658	+	0	ID=cds5207	1	9	STM14_5411	uxuR	DNA-binding transcriptional repressor UxuR	3,03822386	0,303276411
NC_01685(	RefSeq	CDS	4774659	4775672	-	0	ID=cds5208	0	4	STM14_5412	trpS2	tryptophanyl-tRNA synthetase II	0,568400662	-1,710438693
NC_01685(	RefSeq	CDS	4775969	4776121	+	0	ID=cds5209	0	1	STM14_5413	STM14_5413	hypothetical protein	0,142100166	-0,623264422
NC_01685(	RefSeq	CDS	4776390	4776920	+	0	ID=cds5210	0	14,5	STM14_5414	STM14_5414	putative hyperinvasive locus E	1,989402318	-2,376535557
NC_01685(	RefSeq	CDS	4776954	4777076	+	0	ID=cds5211	0	0	STM14_5415	STM14_5415	hypothetical protein	0	NA
NC_01685(	RefSeq	CDS	4776976	4777107	-	0	ID=cds5212	0	0	STM14_5416	STM14_5416	hypothetical protein	0	NA
NC_01685(	RefSeq	CDS	4777159	4777893	+	0	ID=cds5213	0	12,5	STM14_5417	STM14_5417	putative aspartate racemase	1,705201986	-2,329925387
NC_01685(	RefSeq	CDS	4777899	4778807	-	0	ID=cds5214	0	77,33333333	STM14_5418	yjiE	putative DNA-binding transcriptional regulator	10,94171275	-2,008412955
NC_01685(	RefSeq	CDS	4778926	4780098	-	0	ID=cds5215	0	121,5	STM14_5419	iadA	isoaspartyl dipeptidase	17,3362202	-1,854848636
NC_01685(	RefSeq	CDS	4780111	4780572	-	0	ID=cds5216	0	18,5	STM14_5420	yjiG	hypothetical protein	2,55780298	-2,419166771
NC_01685(	RefSeq	CDS	4780569	4781453	-	0	ID=cds5217	0	110,5	STM14_5421	yjiH	putative inner membrane protein	15,63101821	-1,86872757

NC_016856	RefSeq	CDS	4782113	4783297	-	0	ID=cds5218	0	0,25	STM14_5422	yjiJ	putative sugar transporter	0	NA
NC_016856	RefSeq	CDS	4783149	4783325	-	0	ID=cds5219	0	0,25	STM14_5423	STM14_5423	hypothetical protein	0	NA
NC_016856	RefSeq	CDS	4783517	4784788	-	0	ID=cds5220	0	5,5	STM14_5424	yjiN	putative inner membrane protein	0,852600993	-1,98056697
NC_016856	RefSeq	CDS	4784875	4786116	-	0	ID=cds5221	0	18	STM14_5425	yjiO	putative transport protein	2,55780298	-2,419166771
NC_016856	RefSeq	CDS	4786173	4786316	-	0	ID=cds5222	0	2	STM14_5426	STM14_5426	hypothetical protein	0,284200331	-1,182723423
NC_016856	RefSeq	CDS	4786386	4786523	+	0	ID=cds5223	0	0	STM14_5427	STM14_5427	hypothetical protein	0	NA
NC_016856	RefSeq	CDS	4786595	4787110	+	0	ID=cds5224	0	1	STM14_5428	STM14_5428	putative inner membrane protein	0,142100166	-0,623264422
NC_016856	RefSeq	CDS	4787107	4787220	-	0	ID=cds5225	0	1	STM14_5429	STM14_5429	hypothetical protein	0,142100166	-0,623264422
NC_016856	RefSeq	CDS	4787291	4788661	+	0	ID=cds5226	0	4,5	STM14_5430	STM14_5430	putative NAD-dependent aldehyde dehydrogenase	0,568400662	-1,710438693
NC_016856	RefSeq	CDS	4788756	4788899	-	0	ID=cds5227	0	0	STM14_5431	STM14_5431	putative cytoplasmic protein	0	NA
NC_016856	RefSeq	CDS	4788866	4789030	+	0	ID=cds5228	1	0	STM14_5432	yjiS	putative cytoplasmic protein	1,75932237	1,868512432
NC_016856	RefSeq	CDS	4789105	4789806	-	0	ID=cds5229	0	1,5	STM14_5433	STM14_5433	putative inner membrane protein	0,284200331	-1,182723423
NC_016856	RefSeq	CDS	4789880	4790212	-	0	ID=cds5230	0	0	STM14_5434	yjiW	endoribonuclease SymE	0	NA
NC_016856	RefSeq	CDS	4790281	4790412	-	0	ID=cds5231	0	0	STM14_5435	STM14_5435	hypothetical protein	0	NA
NC_016856	RefSeq	CDS	4790439	4791848	-	0	ID=cds5232	0	8,5	STM14_5436	hsdS	type I restriction enzyme specificity protein	1,136801324	-2,147403465
NC_016856	RefSeq	CDS	4791845	4793434	-	0	ID=cds5233	0	35	STM14_5437	hsdM	DNA methylase M	4,973505794	-2,290637485
NC_016856	RefSeq	CDS	4793592	4797101	-	0	ID=cds5234	0	26	STM14_5438	hsdR	type I restriction enzyme EcoKI subunit R	3,694604304	-2,438835558
NC_016856	RefSeq	CDS	4797299	4798213	+	0	ID=cds5235	0	10	STM14_5439	mrr	restriction endonuclease	1,421001655	-2,256999172
NC_016856	RefSeq	CDS	4798305	4798442	+	0	ID=cds5236	0	0	STM14_5440	STM14_5440	hypothetical protein	0	NA
NC_016856	RefSeq	CDS	4798482	4798769	+	0	ID=cds5237	0	3	STM14_5441	STM14_5441	putative inner membrane protein	0,426300497	-1,498211557
NC_016856	RefSeq	CDS	4798756	4799058	+	0	ID=cds5238	0	4	STM14_5442	STM14_5442	putative cytoplasmic protein	0,568400662	-1,710438693
NC_016856	RefSeq	CDS	4799133	4800089	-	0	ID=cds5239	0	6	STM14_5443	yjiA	putative GTP-binding protein YjiA	0,852600993	-1,98056697
NC_016856	RefSeq	CDS	4800100	4800303	-	0	ID=cds5240	0	0	STM14_5444	yjiX	putative cytoplasmic protein	0	NA
NC_016856	RefSeq	CDS	4800398	4802548	-	0	ID=cds5241	0	2	STM14_5445	yjiY	putative carbon starvation protein	0,284200331	-1,182723423
NC_016856	RefSeq	CDS	4802915	4804576	+	0	ID=cds5242	1	29	STM14_5446	tsr	methyl-accepting chemotaxis protein I	5,88022717	-0,769427562
NC_016856	RefSeq	CDS	4804689	4804838	-	0	ID=cds5243	0	1	STM14_5447	STM14_5447	hypothetical protein	0,142100166	-0,623264422
NC_016856	RefSeq	CDS	4804900	4807665	+	0	ID=cds5244	0	37,5	STM14_5448	STM14_5448	putative transcriptional regulator	5,39980629	-2,264331336
NC_016856	RefSeq	CDS	4807767	4808189	+	0	ID=cds5245	0	7,5	STM14_5449	STM14_5449	putative PTS permease	1,136801324	-2,147403465
NC_016856	RefSeq	CDS	4808204	4808665	+	0	ID=cds5246	0	12	STM14_5450	STM14_5450	putative PTS permease	1,705201986	-2,329925387
NC_016856	RefSeq	CDS	4808691	4809470	+	0	ID=cds5247	0	8,5	STM14_5451	STM14_5451	putative PTS permease	1,136801324	-2,147403465
NC_016856	RefSeq	CDS	4809460	4810296	+	0	ID=cds5248	0	19	STM14_5452	STM14_5452	putative PTS permease	2,699903145	-2,42419778
NC_016856	RefSeq	CDS	4810309	4811370	+	0	ID=cds5249	0	34,5	STM14_5453	STM14_5453	putative glucosamine-fructose-6-phosphate aminotransfe	4,831405628	-2,523251848
NC_016856	RefSeq	CDS	4811388	4812398	+	0	ID=cds5250	0	53	STM14_5454	STM14_5454	putative glucosamine-fructose-6-phosphate aminotransfe	7,531308773	-2,151665531
NC_016856	RefSeq	CDS	4812431	4812535	+	0	ID=cds5251	0	2,5	STM14_5455	STM14_5455	hypothetical protein	0,284200331	-1,182723423
NC_016856	RefSeq	CDS	4812532	4814823	-	0	ID=cds5252	0	36,5	STM14_5456	mdoB	phosphoglycerol transferase I	5,115605959	-2,281753063
NC_016856	RefSeq	CDS	4815106	4815567	-	0	ID=cds5253	1	3,5	STM14_5457	yjjA	hypothetical protein	2,327723032	1,125268706
NC_016856	RefSeq	CDS	4815625	4816362	-	0	ID=cds5254	0	7	STM14_5458	dnaC	DNA replication protein DnaC	0,994701159	-2,072978628
NC_016856	RefSeq	CDS	4816365	4816904	-	0	ID=cds5255	0	15	STM14_5459	dnaT	primosomal protein I	2,131502483	-2,392032191
NC_016856	RefSeq	CDS	4816997	4817470	-	0	ID=cds5256	0	0,5	STM14_5460	STM14_5460	hypothetical protein	0	NA
NC_016856	RefSeq	CDS	4817461	4818372	-	0	ID=cds5257	0	10,5	STM14_5461	yjjP	hypothetical protein	1,421001655	-2,256999172
NC_016856	RefSeq	CDS	4818419	4818574	-	0	ID=cds5258	0	0	STM14_5462	STM14_5462	hypothetical protein	0	NA
NC_016856	RefSeq	CDS	4818950	4819675	+	0	ID=cds5259	0	0	STM14_5463	yjjQ	putative transcriptional regulator	0	NA
NC_016856	RefSeq	CDS	4819687	4820310	+	0	ID=cds5260	0	0	STM14_5464	bglJ	DNA-binding transcriptional activator BglJ	0	NA
NC_016856	RefSeq	CDS	4820361	4820819	-	0	ID=cds5261	0	6	STM14_5465	STM14_5465	putative cytoplasmic protein	0,852600993	-1,98056697
NC_016856	RefSeq	CDS	4820970	4821758	-	0	ID=cds5262	0	5,5	STM14_5466	fhuF	ferric hydroximate transport ferric iron reductase	0,852600993	-1,98056697
NC_016856	RefSeq	CDS	4821879	4822943	-	0	ID=cds5263	0	11,5	STM14_5467	STM14_5467	hypothetical protein	1,705201986	-2,329925387
NC_016856	RefSeq	CDS	4823006	4823134	+	0	ID=cds5264	0	1	STM14_5468	STM14_5468	hypothetical protein	0,142100166	-0,623264422
NC_016856	RefSeq	CDS	4823194	4823430	+	0	ID=cds5265	0	1	STM14_5469	STM14_5469	putative inner membrane protein	0,142100166	-0,623264422
NC_016856	RefSeq	CDS	4823971	4824999	-	0	ID=cds5266	0	6	STM14_5473	rsmC	16S ribosomal RNA m2G1207 methyltransferase	0,852600993	-1,98056697
NC_016856	RefSeq	CDS	4824998	4825105	+	0	ID=cds5267	0	0	STM14_5474	STM14_5474	hypothetical protein	0	NA
NC_016856	RefSeq	CDS	4825102	4825539	+	0	ID=cds5268	0	4,5	STM14_5475	holD	DNA polymerase III subunit psi	0,568400662	-1,710438693
NC_016856	RefSeq	CDS	4825484	4825930	+	0	ID=cds5269	0	8,5	STM14_5476	rimI	ribosomal-protein-alanine N-acetyltransferase	1,136801324	-2,147403465
NC_016856	RefSeq	CDS	4825952	4826629	+	0	ID=cds5270	0	10,5	STM14_5477	yjjG	nucleotidase	1,421001655	-2,256999172
NC_016856	RefSeq	CDS	4826721	4828310	+	0	ID=cds5271	1	24	STM14_5478	prfC	peptide chain release factor 3	5,169726343	-0,59430973
NC_016856	RefSeq	CDS	4828502	4828636	+	0	ID=cds5272	0	4	STM14_5479	STM14_5479	hypothetical protein	0,568400662	-1,710438693
NC_016856	RefSeq	CDS	4828712	4829329	+	0	ID=cds5273	0	21	STM14_5480	osmY	hypothetical protein	2,984103476	-2,430148754
NC_016856	RefSeq	CDS	4829440	4829619	+	0	ID=cds5274	0	1	STM14_5481	STM14_5481	hypothetical protein	0,142100166	-0,623264422
NC_016856	RefSeq	CDS	4829743	4830816	+	0	ID=cds5275	0	3,5	STM14_5482	yjjU	putative phosphoesterase	0,568400662	-1,710438693
NC_016856	RefSeq	CDS	4830813	4831586	+	0	ID=cds5276	0	2	STM14_5483	yjjV	putative deoxyribonuclease YjjV	0,284200331	-1,182723423
NC_016856	RefSeq	CDS	4831619	4832482	-	0	ID=cds5277	0	18,75	STM14_5484	yjjW	pyruvate formate lyase-activating enzyme	2,699903145	-2,42419778
NC_016856	RefSeq	CDS	4832454	4834004	-	0	ID=cds5278	0	52,75	STM14_5485	yjjI	hypothetical protein	7,531308773	-2,151665531
NC_016856	RefSeq	CDS	4833992	4834120	+	0	ID=cds5279	0	1	STM14_5486	STM14_5486	hypothetical protein	0,142100166	-0,623264422
NC_016856	RefSeq	CDS	4834246	4835043	+	0	ID=cds5280	0	11,5	STM14_5487	deoC	deoxyribose-phosphate aldolase	1,705201986	-2,329925387
NC_016856	RefSeq	CDS	4835059	4836489	+	0	ID=cds5281	0	10	STM14_5488	deoA	thymidine phosphorylase	1,421001655	-2,256999172
NC_016856	RefSeq	CDS	4836541	4837764	+	0	ID=cds5282	0	50,5	STM14_5489	deoB	phosphopentomutase	7,105008277	-2,173389122
NC_016856	RefSeq	CDS	4837974	4838693	+	0	ID=cds5283	0	32	STM14_5490	deoD	purine nucleoside phosphorylase	4,547205297	-2,489878815
NC_016856	RefSeq	CDS	4838731	4839303	-	0	ID=cds5284	0	1	STM14_5491	STM14_5491	putative outer membrane protein	0,142100166	-0,623264422
NC_016856	RefSeq	CDS	4839300	4841708	-	0	ID=cds5285	0	3	STM14_5492	stjB	putative fimbrial usher protein	0,426300497	-1,498211557
NC_016856	RefSeq	CDS	4841722	4842429	-	0	ID=cds5286	0	0	STM14_5493	stjC	putative periplasmic chaperone protein	0	NA
NC_016856	RefSeq	CDS	4842507	4843196	-	0	ID=cds5287	0	0	STM14_5494	STM14_5494	putative outer membrane protein	0	NA
NC_016856	RefSeq	CDS	4843244	4843933	-	0	ID=cds5288	0	0	STM14_5495	STM14_5495	putative outer membrane protein	0	NA
NC_016856	RefSeq	CDS	4843995	4844144	+	0	ID=cds5289	0	0	STM14_5496	STM14_5496	hypothetical protein	0	NA
NC_016856	RefSeq	CDS	4844246	4845262	-	0	ID=cds5290	0	7	STM14_5497	lplA	lipoate-protein ligase A	0,994701159	-2,072978628
NC_016856	RefSeq	CDS	4845296	4845940	-	0	ID=cds5291	0	1	STM14_5498	smp	hypothetical protein	0,142100166	-0,623264422
NC_016856	RefSeq	CDS	4846057	4847025	+	0	ID=cds5292	0	3,5	STM14_5499	serB	phosphoserine phosphatase	0,568400662	-1,710438693
NC_016856	RefSeq	CDS	4847109	4848491	+	0	ID=cds5293	0	7	STM14_5500	radA	DNA repair protein RadA	0,994701159	-2,072978628
NC_016856	RefSeq	CDS	4848561	4849874	+	0	ID=cds5294	0	16,5	STM14_5501	nadR	nicotinamide-nucleotide adenylyltransferase	2,273602649	-2,403729808
NC_016856	RefSeq	CDS	4849992	4851659	-	0	ID=cds5295	2	57,5	STM14_5502	yjjK	putative ABC transporter ATP-binding protein	11,76045434	-0,823245194
NC_016856	RefSeq	CDS	4851868	4853805	+	0	ID=cds5296	0	9	STM14_5503	slt	lytic murein transglycosylase	1,27890149	-2,207741391
NC_016856	RefSeq	CDS	4853863	4854189	+	0	ID=cds5297	3	3	STM14_5504	trpR	Trp operon repressor	5,704267606	2,218275778
NC_016856	RefSeq	CDS	4854291	4854806	-	0	ID=cds5298	0	3	STM14_5505	yjjX	NTPase	0,426300497	-1,498211557
NC_016856	RefSeq	CDS	4854855	4855502	+	0	ID=cds5299	0	7,5	STM14_5506	gpmB	phosphoglycerate mutase	1,136801324	-2,147403465
NC_016856	RefSeq	CDS	4855499	4856368	-	0	ID=cds5300	0	65,75	STM14_5507	rob	transcriptional regulator	9,378610925	-2,069476383
NC_016856	RefSeq	CDS	4856580	4857053	+	0	ID=cds5301	0	7,25	STM14_5508	creA	hypothetical protein	0,994701159	-2,072978628
NC_016856	RefSeq	CDS	4857066	4857755	+	0	ID=cds5302	0	12,75	STM14_5509	creB	DNA-binding response regulator CreB	1,847302152	-2,356210635
NC_016856	RefSeq	CDS	4857755	4859179	+	0	ID=cds5303	2	15,5	STM14_5510	creC	sensory histidine kinase CreC	5,792247388	0,405774795
NC_016856	RefSeq	CDS	4859237	4860586	+	0	ID=cds5304	0	1	STM14_5511	creD	hypothetical protein	0,142100166	-0,623264422
NC_016856	RefSeq	CDS	4860644	4861729	-	0	ID=cds5305	0	0	STM14_5512	sthE	putative major fimbrial subunit	0	NA
NC_016856	RefSeq	CDS	4861770	4862327	-	0	ID=cds5306	0	0	STM14_5513	sthD	putative fimbrial subunit	0	NA
NC_016856	RefSeq	CDS	4862345	4864882	-	0	ID=cds5307	0	1	STM14_5514	sthB	putative fimbrial usher protein	0,142100166	-0,623264422
NC_016856	RefSeq	CDS	4864928	4865611	-	0	ID=cds5308	0	1	STM14_5515	sthA	putative fimbrial chaperone	0,142100166	-0,623264422
NC_016856	RefSeq	CDS	4865682	4866227	-	0	ID=cds5309	0	0	STM14_5516	STM14_5516	putative fimbrial chaperone	0	NA
NC_016856	RefSeq	CDS	4866566	4867237	-	0	ID=cds5310	0	2,5	STM14_5517	STM14_5517	putative inner membrane protein	0,284200331	-1,182723423
NC_016856	RefSeq	CDS	4867622	4867936	-	0	ID=cds5311	0	1	STM14_5518	STM14_5518	hypothetical protein	0,142100166	-0,623264422
NC_016856	RefSeq	CDS	4868203	4868919	-	0	ID=cds5312	0	122	STM14_5519	arcA	two-component response regulator	17,3362202	-1,854848636
NC_016856	RefSeq	CDS	4869015	4869155	+	0	ID=cds5313	0	0	STM14_5520	yjjY	putative inner membrane protein	0	NA
NC_016856	RefSeq	CDS	4869555	4870241	+	0	ID=cds5314	0	1,5	STM14_5521	lasT	putative RNA methyltransferase	0,284200331	-1,182723423
